# Diastereoselective Self‐Assembly of Low‐Symmetry Pd_*n*_L_2*n*_ Nanocages through Coordination‐Sphere Engineering**

**DOI:** 10.1002/anie.202315451

**Published:** 2023-11-15

**Authors:** Paulina Molinska, Andrew Tarzia, Louise Male, Kim E. Jelfs, James E. M. Lewis

**Affiliations:** ^1^ School of Chemistry University of Birmingham Edgbaston Birmingham B15 2TT UK; ^2^ Department of Applied Science and Technology Politecnico di Torino Corso Duca degli Abruzzi 24 10129 Torino Italy; ^3^ Department of Chemistry Imperial College London, Molecular Sciences Research Hub White City Campus Wood Lane London W12 0BZ UK

**Keywords:** Coordination Cages, Coordination-Sphere, Low-Symmetry, Metallosupramolecular Chemistry, Self-Assembly

## Abstract

Metal‐organic cages (MOCs) are popular host architectures assembled from ligands and metal ions/nodes. Assembling structurally complex, low‐symmetry MOCs with anisotropic cavities can be limited by the formation of statistical isomer libraries. We set out to investigate the use of primary coordination‐sphere engineering (CSE) to bias isomer selectivity within homo‐ and heteroleptic Pd_*n*_L_2*n*_ cages. Unexpected differences in selectivities between alternative donor groups led us to recognise the significant impact of the second coordination sphere on isomer stabilities. From this, molecular‐level insight into the origins of selectivity between *cis* and *trans* diastereoisomers was gained, highlighting the importance of both host–guest and host‐solvent interactions, in addition to ligand design. This detailed understanding allows precision engineering of low‐symmetry MOC assemblies without wholesale redesign of the ligand framework, and fundamentally provides a theoretical scaffold for the development of stimuli‐responsive, shape‐shifting MOCs.

## Graphical abstract

Often overlooked second coordination sphere effects are shown to be of significant importance in isomer selectivity with self‐assembled, low‐symmetry metal‐organic cages. In some instances, these effects override primary structural factors that are often the only consideration when designing such systems.

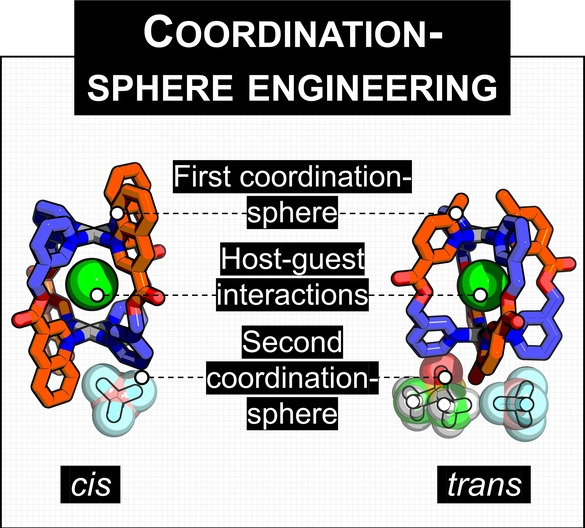

## Introduction

Metal‐organic cages (MOCs) are discrete, porous supramolecular architectures assembled from metal ions/nodes and coordinating ligands.^[1]^ The ability to encapsulate guest molecules within the cavities of MOCs has led to investigations for their use in catalysis,^[2]^ sensing,^[3]^ drug delivery^[4]^ and stabilising reactive species.^[5]^

**Figure 1:**
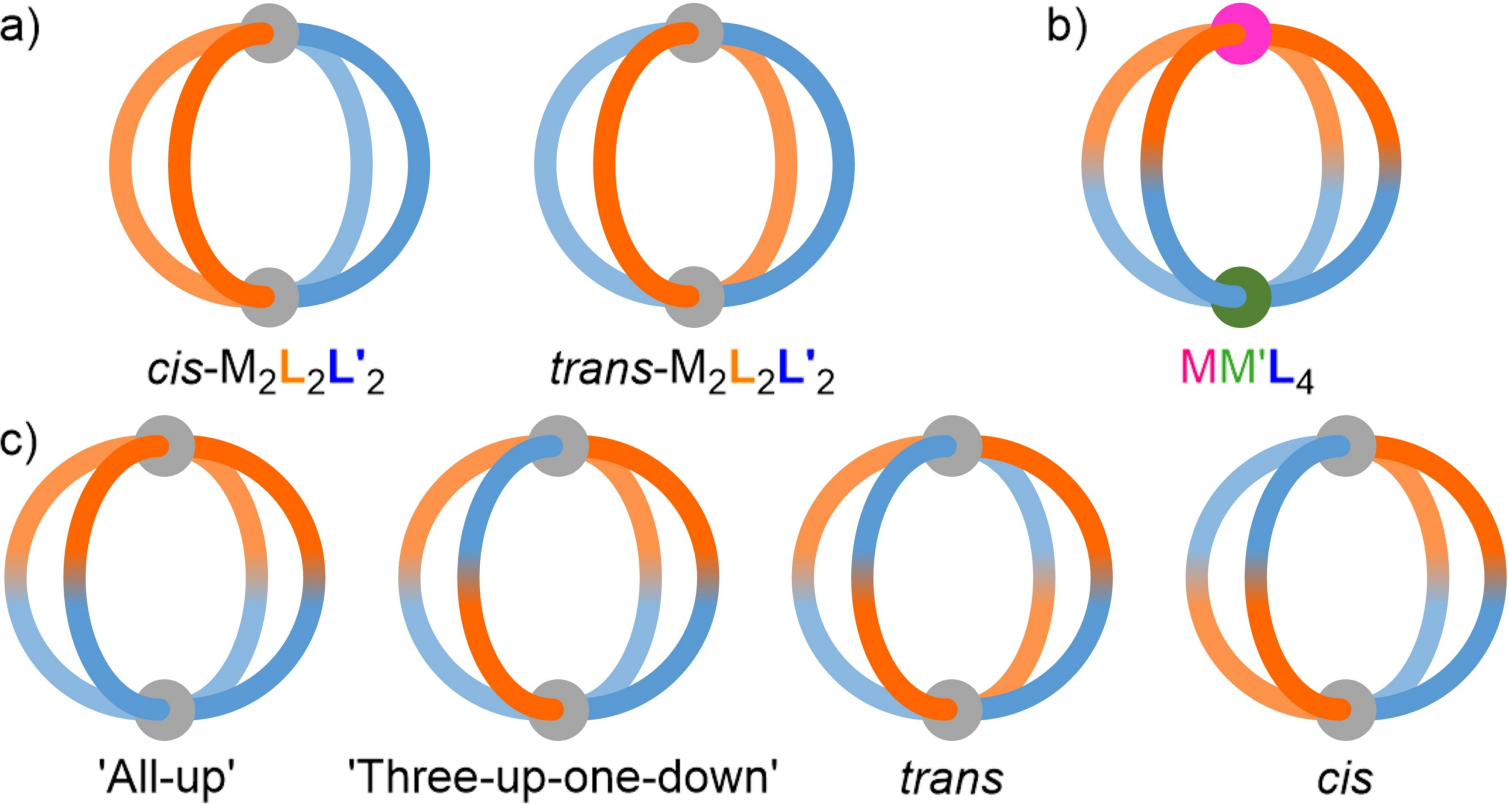
Schematic representations of different unsymmetrical MOCs: a) *cis*‐ and *trans*‐M_2_L_2_L′_2_ heteroleptic cages, b) MM′L_4_ heteronuclear cage, and c) potential isomers of M_2_L_4_ cages assembled from an unsymmetrical ditopic ligand.

Detailed principles behind the self‐assembly of high‐symmetry MOCs have been elucidated over the last four decades. To generate more sophisticated systems^[6]^ with advanced functionality,^[7]^ attention has recently turned to the development of methodologies to access lower symmetry cages.^[8]^ These include the design of mixed‐ligand (heteroleptic)^[9]^ (Figure 1a) and mixed‐metal (heteronuclear) MOCs (Figure 1b),^[10]^ as well as those assembled from low‐symmetry ligands (Figure 1c).^[11]^ Using these approaches, low‐symmetry MOCs have been realised that exhibit shape‐^[12]^ and orientation‐selective^[13]^ guest binding.

The inherent directionality of unsymmetrical ligands gives rise to multiple possible constitutional isomers of their metal‐organic assemblies (Figure 1c). Various strategies have been investigated towards the high‐fidelity, isomer‐selective self‐assembly of low‐symmetry ligands.^[11]^ Aside from developing ligands with mixed‐denticity donors,^[14]^ these include geometric design parameters,[^15^, ^16^] use of non‐covalent interactions within the ligand backbone,^[17]^ and coordination‐sphere engineering (CSE; also known as side‐chain directing).^[18]^ CSE strategies can be subdivided into two further categories: those that use attractive interactions, such as hydrogen‐bonding,^[19]^ and those that use repulsive interactions, such as steric hindrance.

The use of CSE approaches in the metal‐organic self‐assembly of low‐symmetry ligands has been limited,^[20]^ despite its success in directing the self‐assembly of heteroleptic MOCs.[^21^, ^22^, ^23^, ^24^] We have previously reported preliminary findings of two systems that use steric parameters, by themselves and in combination with geometric designs,^[15]^ whilst Crowley and co‐workers have used hydrogen‐bonding interactions to direct formation of a *cis*‐Pd_2_L_4_ cage.^[19]^ Aside from these individual examples, CSE strategies to direct the isomer‐selective assembly of homoleptic MOCs remains a significant, and under‐investigated, challenge.

It was envisaged that combinations of unsubstituted and sterically bulky coordinating groups in unsymmetric ditopic ligands would bias self‐assembly with Pd(II) ions towards specific isomers of M_*n*_L_2*n*_ cages. Given their prior utility in directing the self‐assembly of heteroleptic MOCs, picoline^[22]^ (**L^P^**) and quinoline^[24]^ (**L^Q^**) coordinating groups were chosen for investigation.

In this work, the successful use of CSE in the selective synthesis of Pd_*n*_L_2*n*_ MOC isomers is reported. For both quinoline and picoline ligands, biasing towards assemblies with a 2 : 2 stoichiometry of donors at the metal nodes was observed. Intriguingly, the different coordinating groups were selective for alternative donor arrangements around the metal ions,^[25]^ namely *cis* (**L^Q^**) and *trans* (**L^P^**).

Through careful investigation, the diastereoselectivities between *cis* and *trans* cages were rationalised, and molecular origins for this effect identified, demonstrating the importance of considering the combined effects of both first and second coordination sphere interactions in the design of these supramolecular systems. This detailed understanding has ramifications for the future design of MOCs, particularly those of low‐symmetry, and also stimuli‐responsive systems using CSE approaches.

CSE strategies allow the targeted assembly of MOCs with different symmetries whilst maintaining the structural formulation resulting from the design of the core ligand scaffold. As such, this nuanced approach provides a route for precision engineering the shape of low‐symmetry MOCs, and the cavity spaces within, towards the development of more sophisticated, functional supramolecular hosts.

## Results and Discussion

### Pd_2_L_4_ cages

**Figure 2:**
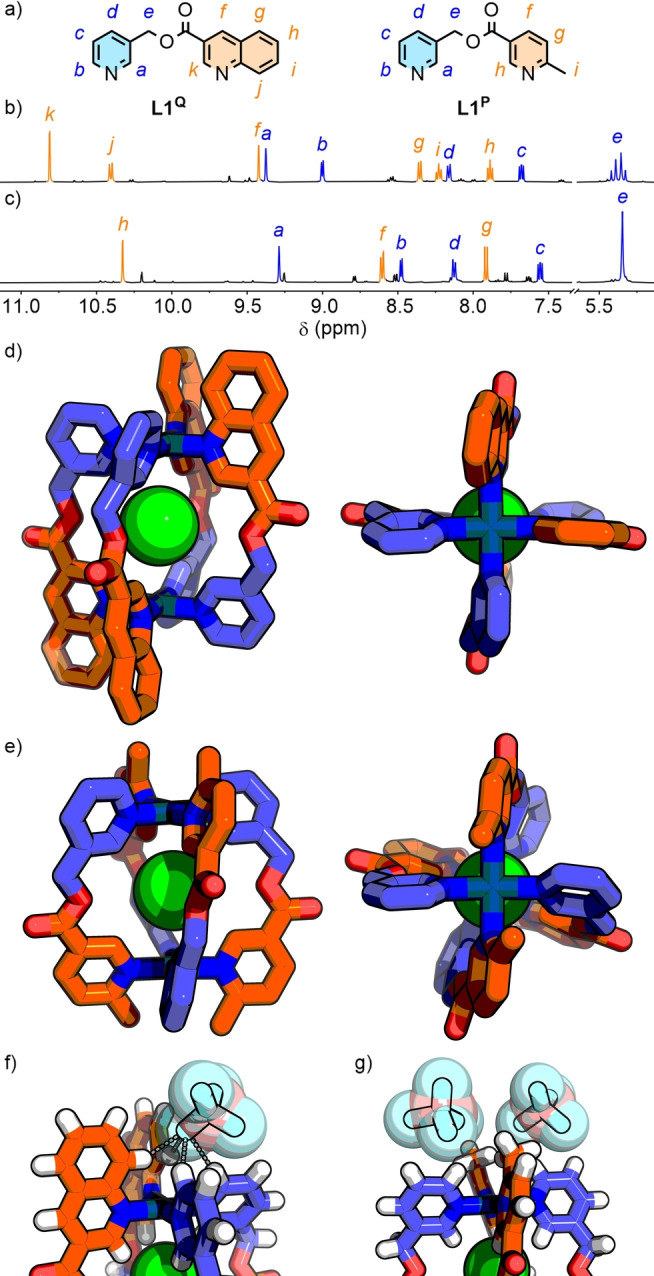
a) Ligands **L1^Q^** and **L1^P^**. Partial ^1^H NMR spectra (500 MHz, *d*_6_‐DMSO, 298 K) of b) **C1^Q^**, and c) **C1^P^**. SCXRD structures of d) *cis*‐**C1^Q^** ⊃Cl (only one crystallographically independent conformer shown), and e) *trans*‐**C1^P^** ⊃Cl. Exohedral interactions of BF_4_^−^ counteranions with the Pd(II) coordination sphere in f) **C1^Q^** (F⋅⋅⋅H 2.31–2.45 Å, C−H⋅⋅⋅F 139–156°), and g) **C1^P^**.

Based on a dipyridyl ligand motif originally reported by Chand and co‐workers,^[26]^ **L1^Q^** and **L1^P^** (Figure 2a) were synthesised by ester condensation between commercially available 3‐(hydroxymethyl)pyridine and the appropriate carboxylic acid. Each ligand was then combined with Pd(NO_3_)_2_ ⋅ 2H_2_O in a 2 : 1 ratio in *d*_6_‐DMSO ([**L1**]=40 mM) and heated at 50 °C for 24 h; no further changes were observed by ^1^H NMR with prolonged heating.

Analysis by electrospray ionisation mass spectrometry (ESI‐MS) indicated formation of Pd_2_L_4_ assemblies (**C1**) for both systems (Figure S22–25 and S53–56). Diffusion‐orientated spectroscopy (DOSY) further supported this, with each system displaying diffusion coefficients (*D*=9.51×10^−11^ and 11.0×10^−11^ m^s^ s^−1^ for **C1^Q^** and **C1^P^**, respectively) consistent with related systems.^[26]^

In both cases a mixture of isomers of **C1** was observed to form by ^1^H NMR (Figure 2b and 2c), but with a major component arising at a compositional fraction greater than expected from a statistical library (i.e. 25 %), demonstrating successful induction of isomer selectivity. The percentage composition of the predominant species was estimated through a comparison of integrals between methylene signals (H_*e*_) and isolated signals in the aromatic region of the ^1^H NMR spectra, giving values of approximately 70 % and 50 % for **C1^Q^** and **C1^P^**, respectively (Figure S21 and S52).

For both major species of **C1**, nuclear Overhauser effect spectroscopy (NOESY) revealed through‐space interactions between signals assigned to the different coordinating groups of **L1** (Figure S17 and S50), identifying these as either the *cis* or *trans* isomers (Figure 1c). In the case of **C1^Q^**, apparent diastereotopic splitting of the CH_2_ signal (*J*≈14 Hz; Figure 2b) was consistent with formation of *cis*‐**C1^Q^** as the major species. The absence of diastereotopic splitting for the major isomer of **C1^P^** led to the conclusion that this was most likely *trans*‐**C1^P^**.

It was also possible to observe second, minor isomers for **C1^Q^** (≈9 %) and **C1^P^** (≈14 %) that were identified as the alternative diastereoisomers *trans*‐**C1^Q^** (ESI section S2.3) and *cis*‐**C1^P^** (ESI section S2.8), respectively.

Related Pd(II) cages are known to encapsulate an NO_3_^−^ anion that can be exchanged for stronger binding halide anions.^[27]^ Upon addition of 1 eq. of Bu_4_NCl to **C1^Q^** and **C1^P^**, encapsulation of Cl^−^ was evidenced by notable downfield shifts of signals assigned to the endohedral protons of the cage (e.g. H_*a*_ and H_*h*_ Δδ=0.49 and 0.65, respectively, for **C1^P^**; Figure S30 and S60).

**C1**⊃Cl^−^ were subsequently prepared and isolated through self‐assembly of **L1** with [Pd(CH_3_CN)_4_](BF_4_)_2_ in the presence of 1 eq. of Bu_4_NCl. Unexpectedly, although the switch to BF_4_^−^ counterions and encapsulation of a Cl^−^ guest in place of NO_3_^−^ anions did not change the identity of the major host isomer, the selectivity values were altered:^[28]^ for **C1^Q^** the *cis* isomer fell to ≈40 % of the mixture (Figure S32), whilst the *trans* isomer of **C1^P^** increased to ≈70 % (Figure S68).

The solid‐state structures of **C1**⊃Cl^−^ were determined by single‐crystal X‐ray diffraction (SCXRD) analysis, which revealed the anticipated *cis*‐ and *trans*‐[Pd_2_(**L1**)_4_⊃Cl]^3+^ assemblies for **C1^Q^** (Figure 2d) and **C1^P^** (Figure 2e), respectively.^[29]^ For **C1^P^**, steric clash of the methyl groups was avoided through induction of a helical twist, with an azimuthal angle (α) of ≈27°. Whilst **C1^Q^** displayed no significant helical twist (α≈1–3°), resulting in a slightly larger Pd⋅⋅⋅Pd distance (6.94–7.05 Å compared to 6.81 Å for **C1^P^**), the planes of pyridine and quinoline units *trans* to each other were rotated to reduce interactions (Θ=3–28°; Figure S250 and Table S2).

### Molecular origins of diastereoselectivity

Interestingly, the relative energies of the *cis* and *trans* isomers of both **C1^Q^** and **C1^P^**, computed using density functional theory (DFT) calculations (HSE06 functional and def2‐SVP basis set with implicit DMSO solvation), suggested the *trans* isomer should be most stable for both systems (ESI section S3). This implied that additional external influences, beyond inherent structural factors, were responsible for the observed speciation. Thus, the question arose: why did **C1^Q^** exhibit selectivity towards the *cis* isomer?

The SCXRD structures showed exohedral BF_4_^−^ anions located in proximity to the Pd(II) ions^[30]^ for both assemblies (Figure 2f and 2g). For **C1^P^** the steric bulk of the methyl groups resulted in a greater F⋅⋅⋅Pd distance compared to **C1^Q^**. Indeed, for **C1^Q^**, interactions between the counterions and C−H of both pyridine and quinoline donors were observed (F⋅⋅⋅H 2.3–2.8 Å, C−H⋅⋅⋅F 139–166°, Figure 2f). This initially led us to consider that the different diastereoselectivities resulted from differences in interactions between cage and counteranions. Specifically, non‐covalent interactions between the BF_4_^−^ anions and **C1^Q^** stabilised the *cis* isomer to such an extent as to make it lower in energy than the more sterically favourable *trans* isomer.

**Figure 3:**
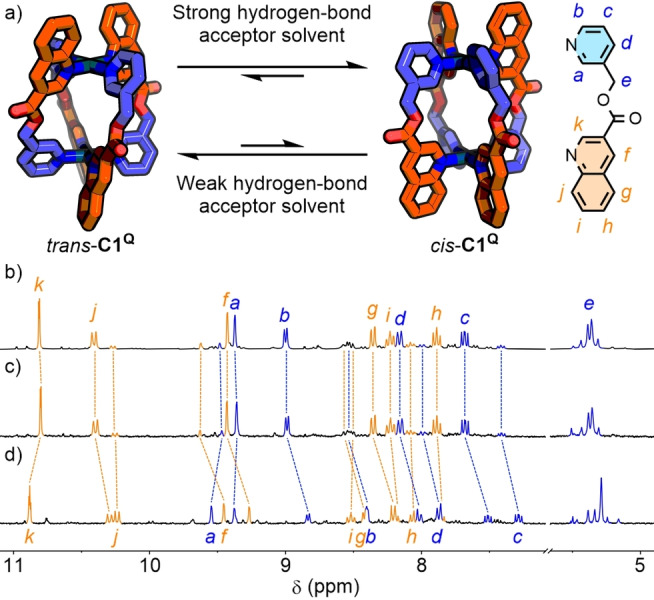
a) Second coordination sphere effects with solvent molecules alter the equilibrium between *cis*‐ and *trans*‐**C1^Q^**. Partial ^1^H NMR spectra (300 MHz, 298 K) of **C1^Q^** ⊃NO_3_ b) 10 mM (*d*_6_‐DMSO) with *cis* isomer peaks labelled, c) 1 mM (*d*_6_‐DMSO), and d) 1 mM (9 : 1 CD_3_CN/*d*_6_‐DMSO) with *trans* isomer peaks labelled.

This hypothesis was probed on two fronts: through dilution ([**C1**] 10–1 mM) in *d*_6_‐DMSO (Figure 3b, 3c, S75 and S76) to investigate the effect of reducing the anion concentration, and the synthesis of **C1^Q^** ⊃NO_3_ in the presence of excess BF_4_^−^ or ^−^OTf to monitor the effect of increased anion concentration and stoichiometry (Figure S71). No impact on the diastereoselectivity was observed from either study (ESI section 2.11).

The thought occurred that, whilst the BF_4_^−^ anions were located around the Pd(II) nodes in the solid‐state, in solution the concentration of strongly hydrogen‐bond accepting DMSO molecules would be orders of magnitude higher. The idea that solvent molecules, rather than anions, interacting with the coordination sphere of the cages were responsible for stabilisation of the *cis* assemblies was thus examined experimentally.

The dilution studies were revisited using CD_3_CN—a weaker hydrogen bond acceptor—as titrant (DMSO and MeCN have hydrogen bond acceptor parameters, *β*, of 8.9 and 4.7, respectively^[31]^). In this instance, the proportion of the *trans* isomer of **C1^P^** increased slightly with dilution (Figure S81 and S82). Meanwhile, for **C1^Q^**, the percentage of the *cis* cage decreased; as the proportion of CD_3_CN increased, the minor *trans* isomer became more prominent (Figure S77 and S78). At a solvent ratio of 9 : 1 CD_3_CN/*d*_6_‐DMSO, the percentage composition of *trans*‐**C1^Q^** actually superceded that of the *cis* isomer (Figure 3d). Similarly, addition of D_2_O (*β*=4.5)^[31]^ to a *d*_6_‐DMSO solution of **C1^Q^** also resulted in enhancement of the *trans* isomer at the expense of the *cis* (Figure S72 and S74).

It was concluded that, in the absence of additional effects, the *trans* isomer is favoured for both **C1^P^** and **C1^Q^** purely on grounds of steric hindrance. The *cis* isomers, however, provide a suitable site around the coordination sphere for interacting with hydrogen bond acceptors (Figure 3a). Consequently, employing as solvent DMSO—a strong hydrogen bond acceptor—led to a reduction in the relative energy of the *cis* isomer. For **C1^Q^** this effect was more pronounced due to the four polarised aromatic C−H bonds (H_*b*_ and H_*j*_) around each Pd(II) ion (compared to just two for **C1^P^**−H_*b*_).

The difference in isomer selectivity between **C1**⊃NO_3_^−^ and **C1**⊃Cl^−^ is proposed to arise partly from a reduction in Pd⋅⋅⋅Pd distance, with the smaller guest inducing an increased helical pitch.^[32]^ This increased the offset of bulky donor groups in the *trans* isomer, further reducing its relative energy, observed as an increase in diastereoselectivity towards *trans*‐**C1**. For **C1^Q^**, the conformational changes upon guest exchange may also impact the complementarity between the external binding pocket and solvent molecules, reducing effective stabilisation of the *cis* isomer. This was suggested by a lack of shift in the quinoline resonance H_*j*_ for *trans*‐**C1^Q^** (Δδ=0.01 ppm), in contrast to *cis*‐**C1^Q^** (Δδ=0.11 ppm), upon increasing CD_3_CN composition (Figure 3b–d), indicative of H_*j*_ being incapable of significant interactions with solvent molecules in the *trans* cage isomer.

From the combined data, we have constructed a molecular‐level picture of the multiple interactions that influence the observed diastereoselectivities between *cis* and *trans* cages. The ligand design, endohedral interactions between host and guest, and exohedral interactions with solvent molecules all contribute to the relative energies of the diastereomers. Thus, both first and second coordination spheres^[33]^ play an important role in directing the self‐assembly process. Modulation of these factors enables control over isomer selectivity, and opens up the possibility for stimuli‐responsive switching of the equilibrium position within isomer libraries.^[34]^ More detailed investigations into this effect are underway and will be reported in due course.

### Larger Pd_2_L_4_ cages

**Figure 4:**
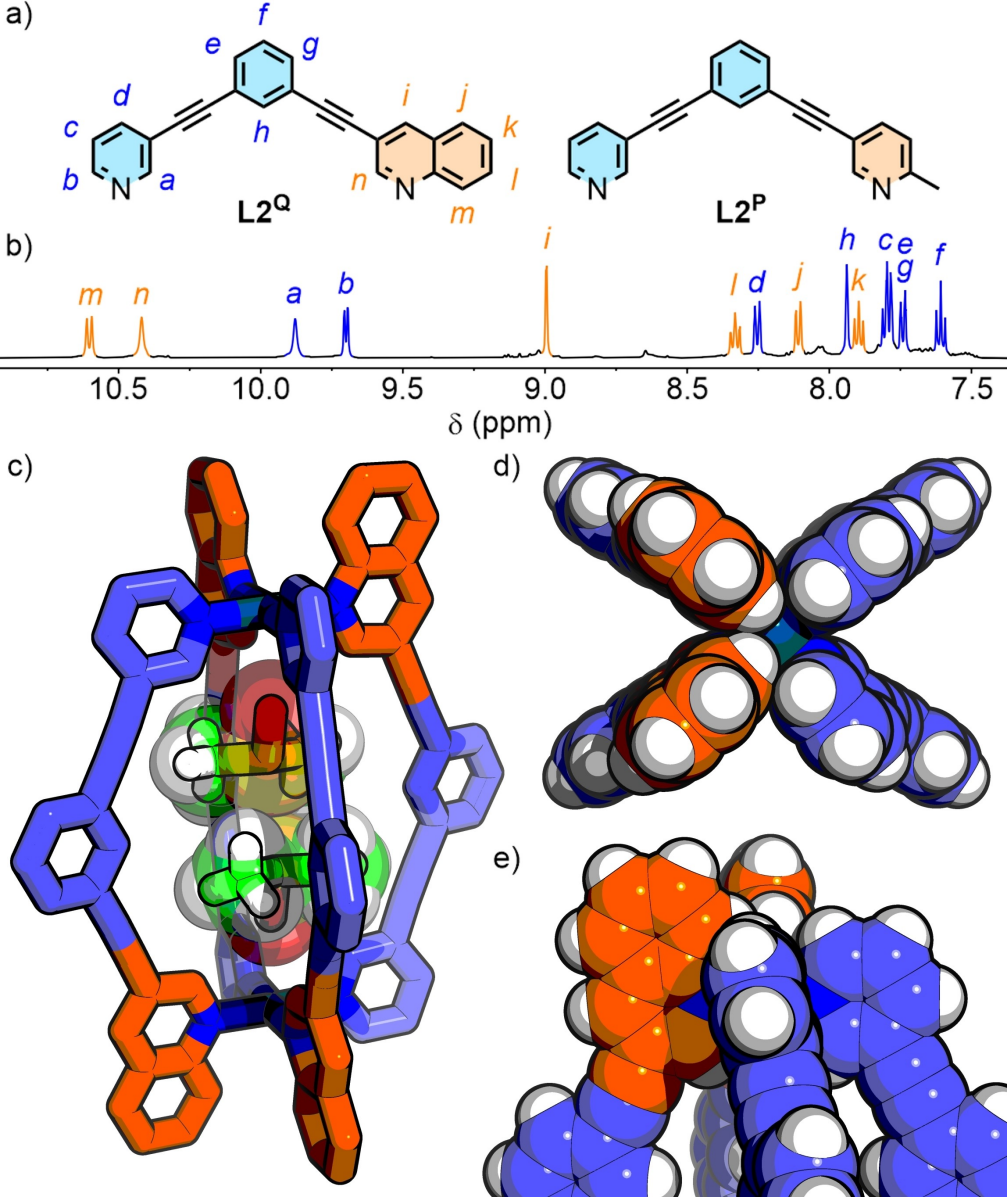
a) Ligands **L2^Q^** and **L2^P^**; b) Partial ^1^H NMR spectrum (500 MHz, *d*_6_‐DMSO, 298 K) of **C2^Q^**; SCXRD structure of *cis*‐**C2^Q^** showing c) *cis*‐**C2^Q^** ⊃DMSO_2_, d) view down the Pd⋅⋅⋅Pd axis, and e) *cis* coordination environment around a Pd(II) ion.

To probe the utility of these designs with alternative ligand scaffolds, ligand **L2^Q^** (Figure 4a) was investigated. We have previously reported the self‐assembly of ligand **L2^P^**; in *d*_6_‐DMSO a mixture of the *cis‐* and *trans*‐Pd_2_L_4_ cage (**C2**) isomers formed, whilst in CD_3_CN *trans*‐**C2^P^** formed essentially exclusively.^[15]^ It had been suggested that this behaviour arose from the higher polarity DMSO solvent stabilising the more polar *cis* isomer,^[35]^ without being able to provide a more detailed explanation. In light of the new investigations with **C1**, it is now proposed that the stronger hydrogen bond acceptor nature of DMSO, compared to CH_3_CN, leads to enhanced stabilisation of the *cis*‐**C2^P^** isomer specifically through hydrogen bonding interactions between solvent molecules and the exohedral face of the Pd(II) coordination sphere. These interactions are less favoured with the *trans* isomer which, excluding other factors, provides the least sterically hindered primary coordination sphere.

Self‐assembly of **L2^Q^** with Pd(II) (as the BF_4_^−^ salt) in *d*_6_‐DMSO resulted in what, superficially, appeared to be near‐quantitative (estimated at 70 % by NMR integration; Figure S114) formation of a single species, **C2^Q^** (Figure 4b). In combination, the high‐symmetry NMR spectra (Figure 4b and S104–106), ESI‐MS (Figure S115–118) and NOESY (H_*a*_⋅⋅⋅H_*n*_, H_*b*_⋅⋅⋅H_*m*_; Figure S112) data identified **C2^Q^** as either *cis*‐ or *trans*‐Pd_2_(**L2^Q^**)_4_. The absence of prochiral units within the ligand structure prevented the use of diastereotopic splitting (or lack thereof) as a diagnostic tool to differentiate the two isomers in solution.

The solid‐state structure of **C2^Q^** was determined by SCXRD and revealed, as expected, a *cis* arrangement of ligands within the assembly (Figure 4c–e).^[29]^ Consequently, it seemed the preference under these conditions for *cis* and *trans* coordination environments when pairing pyridine with quinoline and picoline, respectively, holds for different ligand scaffolds.

### Pd_3_L_6_ ‘double‐walled’ triangles

**Figure 5:**
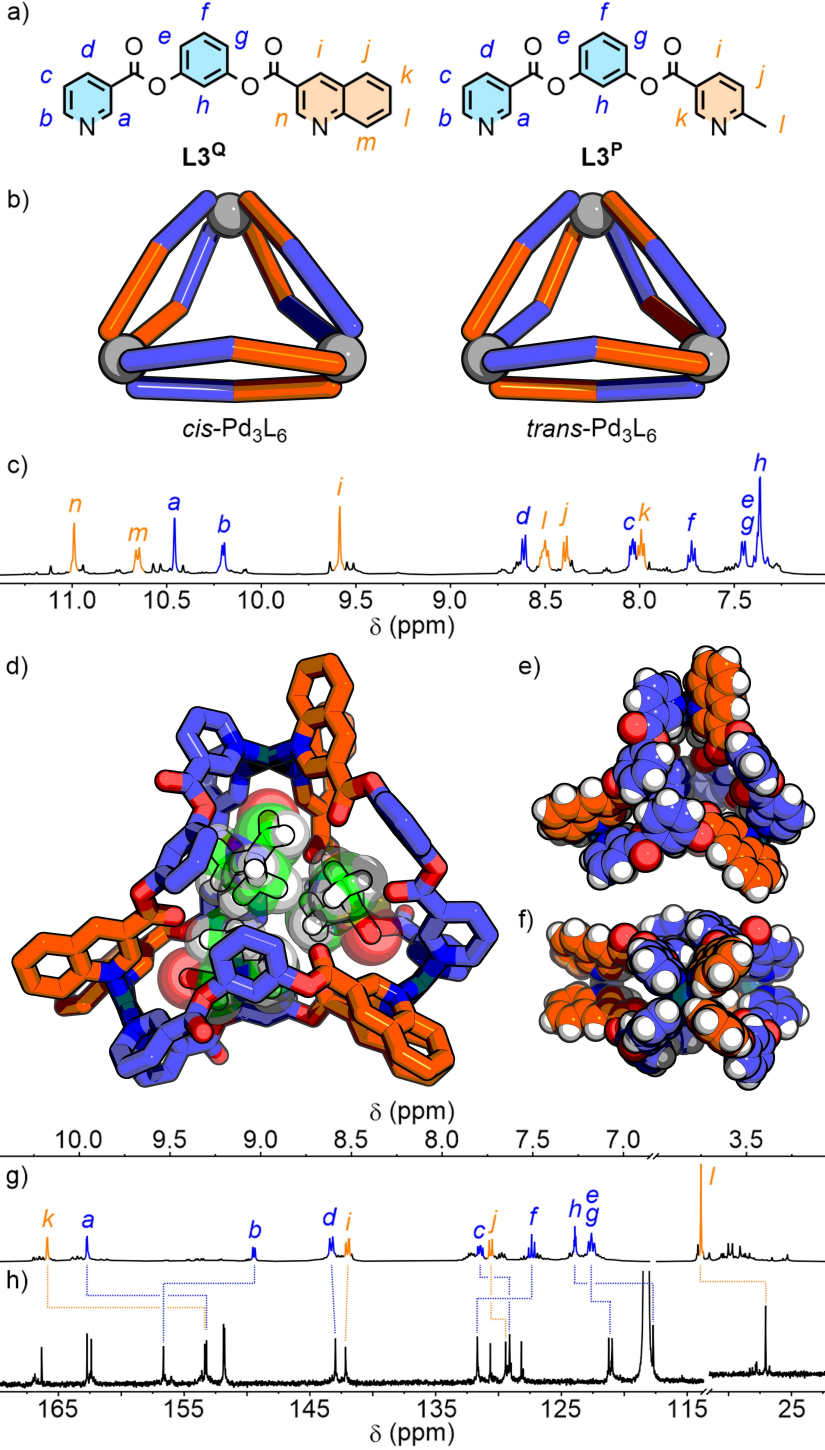
a) Ligands **L3^Q^** and **L3^P^**; b) schematic representations of Pd_3_L_6_ ‘double‐walled’ triangle isomers with all‐*cis* and all‐*trans* donor arrangements; c) ^1^H NMR spectrum (500 MHz, *d*_6_‐DMSO) of **C3^Q^**; SCXRD structure of *cis*‐**C3^Q^** showing d) *cis*‐**C3^Q^** ⊃DMF_2_DMSO, and space filling representation e) from the top, and f) from the side; g) ^1^H NMR spectrum (500 MHz, CD_3_CN) and h) ^13^C NMR spectrum (126 MHz, CD_3_CN) of **C3^P^**.

To explore higher nuclearity systems, **L3^Q^** and **L3^P^** (Figure 5a) were synthesised. Symmetric, dipyridyl analogues of these ditopic ligands^[26]^ have been shown to assemble into ‘double‐walled’ Pd_3_L_6_ triangles (Figure 5b).[^36^, ^37^] For such structures assembled from an unsymmetrical ligand, one instance of which has been reported by Chand and co‐workers,^[38]^ there are 9 possible isomers. Two of these provide all‐*cis* or all‐*trans* arrangements of donors at the three metal nodes (Figure 5b). From our understanding of isomer selectivities induced by CSE, it was hypothesised that self‐assembly of **L3^Q^** with Pd(II) in DMSO would favour formation of the *cis*‐Pd_3_L_6_ isomer of **C3**, whilst **L3^P^** would be biased towards the *trans* assembly.

**C3^Q^** exhibited a set of dominant signals in the ^1^H NMR spectrum (Figure 5c). The diffusion coefficient of this major species, derived from DOSY, corresponded to a solvodynamic radius of 15 Å, whilst isotopic patterns observed by ESI‐MS were consistent with assemblies possessing the anticipated Pd_3_L_6_ formulation (Figure S139–146). This major species was estimated by integration of ^1^H NMR signals to constitute ≈35 % of the isomeric mixture (Figure S138), over three‐fold that of a statistical library (11 %). As with the previous systems, the high symmetry of the NMR spectra and through‐space interactions observed by NOESY (H_*a*_⋅⋅⋅H_*n*_ and H_*b*_⋅⋅⋅H_*m*_) were consistent with only the all‐*cis* or all‐*trans* isomers (Figure 5b).

Weakly diffracting crystals were grown that required the use of synchrotron radiation to obtain satisfactory SCXRD data.^[29]^ The solid‐state structure (Figure 5d–f), however, unambiguously revealed each pair of ligands along the three “walls” to be aligned parallel, with a ‘head‐to‐tail’ arrangement of the ligands on each face of the triangle. This gave, at each of the three metal nodes, the anticipated *cis* arrangement of quinoline and pyridine donors.

In contrast to **L3^Q^**, the equilibrated self‐assembly mixture of **L3^P^** and Pd(II) in *d*_6_‐DMSO resulted in a ^1^H NMR spectrum that appeared to show formation of a mixture of assemblies, with no clear dominant species. Given the established impact of solvent on the equilibrium between *cis* and *trans* isomers, self‐assembly with **L3^P^** was re‐examined in CD_3_CN. This yielded a much simpler ^1^H NMR spectrum (Figure 5g), with a clear dominant species (≈25 % of mixture, Figure S175).

The major product was of high symmetry, as observed by both ^1^H (Figure 5g) and ^13^C NMR (Figure 5h) which, combined with NOE interactions between the pyridyl and picolyl donor groups (H_*b*_⋅⋅⋅H_*l*_; Figure S174) and ESI‐MS data (Figure S176–186) again indicated formation of either the *cis*‐ or *trans*‐Pd_3_L_6_ assembly as the major species. Despite multiple attempts, single crystals suitable for X‐ray diffraction could not be obtained in our hands and the lack of prochiral units prevented the use of NMR spectroscopy to distinguish between the two possible isomers. Consequently, we turned to DFT to investigate the relative energies of the *cis* and *trans* assemblies.

Unexpectedly, use of different functionals for the geometry optimisations resulted in a switching of the *cis* (HSE) or *trans* (PBE0) **C3^P^** isomer being lower in energy (ESI section S3). It has been demonstrated how environmental perturbations significantly impact the stability of the individual isomers. Thus, without the suitable inclusion of explicit encapsulated and exohedral solvent molecules and anions within these models, the balance of calculated energies between isomers can be easily swayed. Based on the experimental data obtained for **C1**, and the DFT calculations previously performed on the more rigid **C2** systems, it is tentatively suggested that the most likely identity of the major isomer of **C3^P^** is the *trans* assembly.

### Heteroleptic self‐assembly

**Figure 6:**
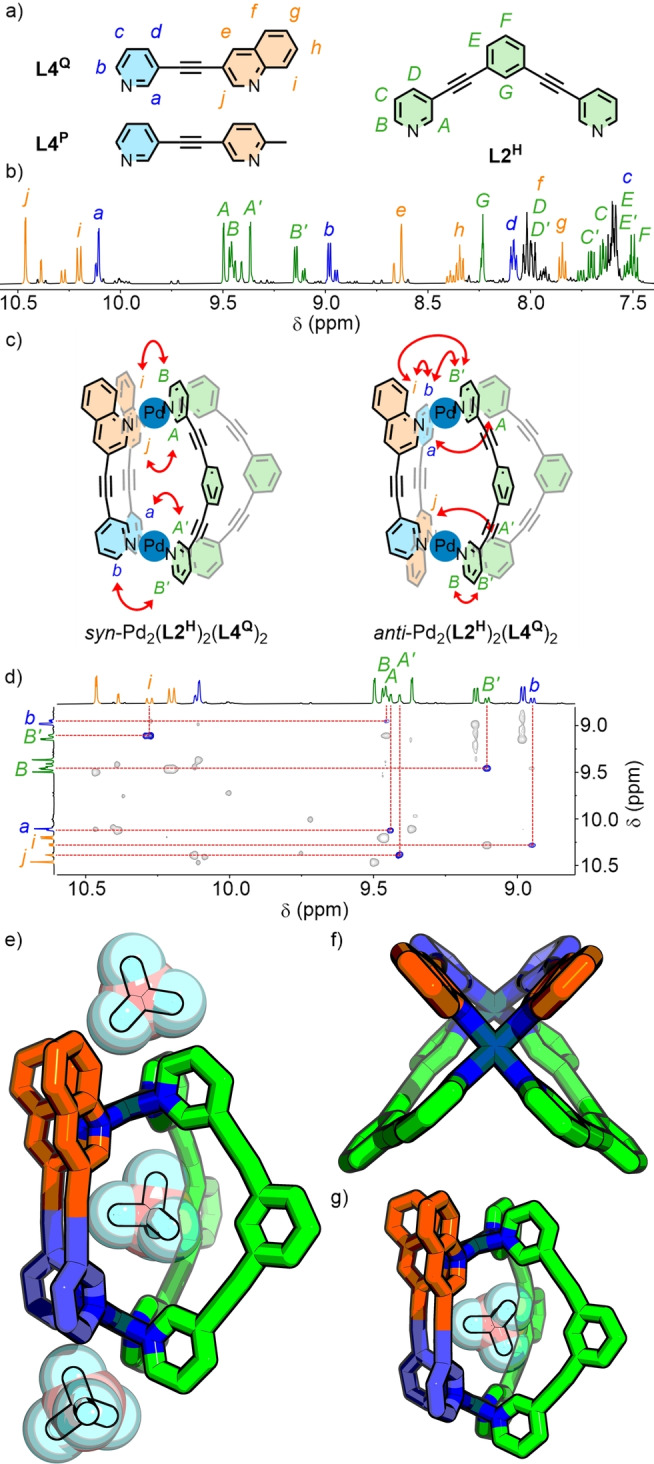
a) Ligands **L4^Q^**, **L4^P^** and **L2^H^**; b) ^1^H NMR spectrum (500 MHz, CD_3_CN) of [Pd_2_(**L2^H^**)_2_(**L4^Q^**)_2_](BF_4_)_4_ with major *syn* isomer peaks labelled; c) through‐space interactions observed by NOESY in the two heteroleptic isomers; d) NOESY spectrum (500 MHz, CD_3_CN) with minor *anti* isomer peaks labelled. SCXRD structure of *syn*‐Pd_2_(**L2^H^**)_2_(**L4^Q^**)_2_ e) with endo‐ and exohedral BF_4_^−^ counteranions, f) viewed down the Pd⋅⋅⋅Pd axis, and g) second crystallographically independent molecule with alternative co‐conformation of internal BF_4_^−^ anion.

Preliminary investigations to extend these CSE designs to Pd_4_L_8_ ‘double‐walled’ tetrahedra[^37^, ^39^, ^40^] assembled from ligands **L4** (Figure 6a)—synthesised through copper‐free Sonogashira couplings^[41]^—proved prohibitively difficult, with complex NMR spectra obtained from equilibrated mixtures with Pd(II) (ESI Section S2.22 and S2.24). This is perhaps unsurprising. In a statistical library, each of the 35 possible isomers^[42]^ would constitute <3 % of the mixture, with even the most symmetrical cages (i.e. *cis* and *trans*) possessing two ligand environments. Without near quantitative selectivity, identification of NMR signals for a particular isomer would likely be an insurmountable challenge. Consequently, further exploration of these systems was not attempted.

Inspired by a recent report of a heteroleptic *cis*‐Pd_2_ **L**_2_ **L′**_2_ MOC,^[40]^ there was motivation to investigate the potential integrative self‐assembly between **L4** and symmetric ligand **L2^H^**. From such a mixed‐ligand assembly there would be two possible isomers: the *syn* and the (chiral) *anti* isomer, with the pair of **L4** ligands arranged in the same or opposite directions, respectively (Figure 6c).

After equilibrating a stoichiometric mixture of **L2^H^** and **L4^Q^** with Pd(II) in CD_3_CN, DOSY (Figure S224) and ESI‐MS (Figure S226–230) confirmed formation of heteroleptic assemblies with the anticipated Pd_2_(**L2^H^**)_2_(**L4^Q^**)_2_ formulation. Meanwhile, NMR analysis demonstrated the presence of two spectroscopically similar species in an approximately 3 : 1 ratio (Figure 6b and S225).

The lack of bilateral symmetry in **L4^Q^** induced a lowering of the symmetry of **L2^H^** in both cage isomers, resulting in distinct signals for all 12 protons, corroborated by the ^13^C NMR spectrum (Figure S211). Whilst analysis of the ^1^H NMR spectrum was made challenging by significant signal overlap, 2D NMR techniques (COSY, HMBC and TOCSY) enabled assignment of all peaks for both isomers (Figure S205).

NOESY and ROESY were employed to determine the identities of the two isomers (Figure 6c). Observation of particular through‐space interactions (H_*B*_⋅⋅⋅H_*B’*_ and H_*b*_⋅⋅⋅H_*i*_⋅⋅⋅H_*B’*_) for the minor species (Figure 6d) led to the conclusion that this was the less sterically congested *anti* isomer. The more limited NOE interactions observed for the major assembly demonstrated that this was the *syn* isomer, with both **L4^Q^** ligands arranged parallel. This result further supported the conclusion that, rather than selectivity towards the *cis* isomers of **C1−C3** being purely driven by repulsive steric effects, additional stabilising interactions promoted this ligand arrangement. In this instance, presumably the two different second coordination sphere sites around the Pd(II) ions in the *syn* isomer provide more favourable interactions with solvent molecules compared to those of the *anti* cage.

The anticipated structure of *syn*‐Pd_2_(**L2^H^**)_2_(**L4^Q^**)_2_ was confirmed by SCXRD, with both **L4^Q^** ligands arranged parallel to each other (Figure 6e–g).^[29]^ The solid‐state structure of the heteroleptic cage was found with one BF_4_^−^ anion encapsulated within the cavity, and external counterions interacting with the external face of the coordination spheres around the two Pd(II) ions (Figure 6e).

The integrative self‐assembly between **L2^H^** and **L4^P^** was also attempted. Whilst ESI‐MS (Figure S233–241) demonstrated the presence of the heteroleptic assembly (alongside minor signals for the homoleptic Pd_2_(**L2^H^**)_4_), signal resolution in the NMR spectrum was insufficient to enable effective analysis (Figure S232).

We have previously been able to arrange two different unsymmetrical ligand scaffolds in defined relative orientations through covalent tethering, forming *pseudo*‐heteroleptic MOCs.^[43]^ Using CSE, we now demonstrate the ability to assemble truly heteroleptic MOCs, derived through integrative self‐assembly of ligands with the same denticity, incorporating unsymmetrical scaffolds in an orientationally‐selective manner.^[44]^

## Conclusion

We have prepared a range of unsymmetrical ditopic ligands, with varying backbone scaffolds, incorporating a pyridine donor paired with a sterically bulky quinoline or picoline moiety, and investigated their self‐assembly into Pd_*n*_L_2*n*_ architectures. This coordination‐sphere engineering approach was successful in biasing self‐assembly towards specific isomers from a statistical library. Interestingly, quinoline and picoline units promoted *cis* and *trans* arrangements of donors at the metal centres, respectively, resulting in diastereoselectivity of the self‐assembly process towards different isomers of particular architectures. The ability to use this relatively subtle difference to target the formation of specific metal‐organic assembly isomers provides a nuanced approach towards directing the self‐assembly of unsymmetrical ligand scaffolds.

After probing the source of this difference in selectivity, it was concluded that interactions between solvent molecules and the exterior of the cage around the metal nodes play a crucial role in determining the relative stabilities of isomers. Thus, not only is the first coordination sphere important in directing the self‐assembly, but second coordination sphere effects play a critical role and can, in fact, supercede the directing effects of the primary structure. This insight further opens up the potential for designing stimuli‐responsive,^[45]^ shape‐shifting systems^[46]^ that respond to changes in their environment.

The orientationally selective incorporation of an unsymmetrical ligand scaffold into a heteroleptic M_2_L_2_L′_2_ MOC was also demonstrated. Consistent with the homoleptic assemblies, the major isomer that formed had both bulky quinoline donor units coordinating to the same Pd(II) ion. This result further supported the hypothesis that isomer selectivity can be affected by interactions beyond those simply between components within the covalent structure.

This flexible strategy adds a new approach to preparing metal‐organic hosts with increased anisotropy to the metallosupramolecular chemist's toolbox. The continued development of methods to access more structurally sophisticated metal‐organic cages^[6]^ will lead to supramolecular hosts exhibiting higher‐level behaviours,^[7]^ reminiscent of the impressive properties of natural architectures, like enzymes, that have long provided a source of inspiration for chemists.

## Author Contributions

PM—synthesis and characterisation, analysis; LM—SCXRD analysis; AT and KEJ—computational modelling; JEML—synthesis and characterisation, analysis, conceptualisation, supervision, writing—original draft. All authors contributed to reviewing of the final manuscript.

## Conflict of interest

The authors declare no conflict of interest.

1

## Supporting information

As a service to our authors and readers, this journal provides supporting information supplied by the authors. Such materials are peer reviewed and may be re‐organized for online delivery, but are not copy‐edited or typeset. Technical support issues arising from supporting information (other than missing files) should be addressed to the authors.

Supporting Information

Supporting Information

## Data Availability

The data that support the findings of this study are available in the supplementary material of this article.
