# Supplementary material for: Diastereoselective Self‐Assembly of Low‐Symmetry Pd n L2n Nanocages through Coordination‐Sphere Engineering
Source: Angew Chem Int Ed Engl. 2023 Nov 15;62(51):e202315451. doi: 10.1002/anie.202315451 (PMC10952360; doi:10.1002/anie.202315451)
Supplement: Supplementary file 2 — Supporting Information [file ANIE-62-0-s001.pdf]

Angewandte Chemie
Eine Zeitschrift der Gesellschaft Deutscher Chemiker

Supporting Information

# Diastereoselective Self-Assembly of Low-Symmetry $Pd_nL_{2n}$ Nanocages through Coordination-Sphere Engineering

*P. Molinska, A. Tarzia, L. Male, K. E. Jelfs, J. E. M. Lewis**

**Contents**

S1. General Experimental ........ 3
S2. Synthetic Procedures ........ 4
S2.1 Synthesis of **L1**$^{Q}$ ........ 4
S2.2 Synthesis of *cis*-**C1**$^{Q}$ ........ 9
S2.3 Identification of minor *trans*-**C1**$^{Q}$ isomer ........ 19
S2.4 *cis*-**C1**$^{Q}$ + $Bu_4NCl$ ........ 21
S2.5 Synthesis of *cis*-**C1**$^{Q}$⊃Cl ........ 22
S2.6 Synthesis of **L1**$^{P}$ ........ 24
S2.7 Synthesis of *trans*-**C1**$^{P}$ ........ 28
S2.8 Identification of minor *cis*-**C1**$^{P}$ isomer ........ 37
S2.9 *trans*-**C1**$^{P}$ + $Bu_4NCl$ ........ 39
S2.10 Synthesis of *trans*-**C1**$^{P}$⊃Cl ........ 40
S2.11 Investigation of anion and solvent effects on **C1** speciation ........ 46
S2.12 Synthesis of **S1** ........ 54
S2.13 Synthesis of **S2** ........ 59
S2.14 Synthesis of **L2**$^{Q}$ ........ 63
S2.15 Synthesis of *cis*-**C2**$^{Q}$ ........ 68
S2.16 Synthesis of **L3**$^{Q}$ ........ 76
S2.17 Synthesis of *cis*-**C3**$^{Q}$ ........ 82
S2.18 Synthesis of **S3** ........ 92
S2.19 Synthesis of **L3**$^{P}$ ........ 96
S2.20 Synthesis of *trans*-**C3**$^{P}$ ........ 102
S2.21 Synthesis of **L4**$^{Q}$ ........ 114
S2.22 Self-assembly of **L4**$^{Q}$ with $[Pd(CH_3CN)_4](BF_4)_2$ ........ 118
S2.23 Synthesis of **L4**$^{P}$ ........ 120
S2.24 Self-assembly of **L4**$^{P}$ with $[Pd(CH_3CN)_4](BF_4)_2$ ........ 124
S2.25 Synthesis of $[Pd_2($**L2**$^{H})_2($**L4**$^{Q})_2](BF_4)_4$ ........ 126
S2.26 Synthesis of $[Pd_2($**L2**$^{H})_2($**L4**$^{P})_2](BF_4)_4$ ........ 141
S3. Density Functional Theory Calculations ........ 147
Building block construction ........ 147
Cage model construction ........ 147
DFT methodology ........ 148
Geometry-optimised structures ........ 148
Isomer energy comparisons ........ 150
S4. Solvodynamic Radii Calculations ........ 151
S5. X-ray Crystallography ........ 152

S5.1 *cis*-[$Pd_2$(**L1$^Q$**)$_4$⊃Cl]($BF_4$)$_3$ ........ 152
S5.2 *trans*-[$Pd_2$(**L1$^P$**)$_4$⊃Cl]($BF_4$)$_3$ ........ 157
S5.3 *cis*-[$Pd_2$(**L2$^Q$**)$_4$⊃$DMSO_2$]($BF_4$)$_2X_2$ ........ 159
S5.4 *cis*-[$Pd_3$(**L3$^Q$**)$_6$⊃$DMF_2$DMSO]($BF_4$)$_6$ ........ 160
S5.5 [$Pd_2$(**L2$^H$**)$_2$(**L4$^Q$**)$_4$⊃$BF_4$]($BF_4$)$_3$ ........ 162
S6. References ........ 163

## S1. General Experimental

**Synthesis:** Unless otherwise stated, all reagents, including anhydrous solvents, were purchased from commercial sources and used without further purification. All reactions were carried out under an atmosphere of $N_2$ using degassed, anhydrous solvents unless otherwise stated. Petrol refers to the fraction of petroleum ether boiling in the range 40-60 °C. Analytical TLC was performed on pre-coated silica gel plates (0.25 mm thick, 60F254, Merck, Germany) and observed under UV light.

**Analysis:** NMR spectra were recorded on Bruker AV300, AV400 or AV500 instrument, at a constant temperature of 300 K. Chemical shifts (δ) are reported in parts per million (ppm) and referenced to residual solvent peaks ($CDCl_3$: $^1$H δ 7.26 ppm,$^{13}$C δ 77.16 ppm; $CD_3CN$: $^1$H δ 1.94 ppm, $^{13}$C δ 1.32 & 118.26 ppm; $d_6$-DMSO: $^1$H δ 2.50 ppm, $^{13}$C δ 39.52; $d_6$-acetone $^1$H δ 2.05, $^{13}$C δ 206.26 & 29.84). Chemical shifts are reported in parts per million from low to high field and referenced to residual solvent. Standard abbreviations indicating multiplicity were used as follows: m = multiplet, quint = quintet, q = quartet, t = triplet, d = doublet, s = singlet, app. = apparent, br. = broad. Signal assignment was carried out using 2D NMR methods (HSQC, HMBC, COSY, NOESY) where necessary. In the case of some signals absolute assignment was not possible. Here indicative either/or assignments (e.g. $H_A$/$H_B$ for $H_A$ or $H_B$) are provided. Mass spectrometry was carried out by the Mass Spectrometry for Chemistry Research facility at the School of Chemistry, University of Birmingham using an Orbitrap Exploris GC for EI-MS, a Wasters Synapt G2-S for ESI-MS and a Waters Xevo G2-XS for ASAP-MS.

**L2$^H$** was synthesised according to literature procedure.[1]

# S2. Synthetic Procedures

## S2.1 Synthesis of L1$^{Q}$

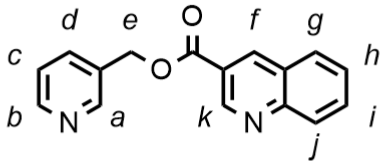


To a stirring solution of 3-quinolinecarboxylic acid (0.173 g, 1.0 mmol, 1.0 eq.) and DMAP (12.7 mg, 0.10 mmol, 0.1 eq.) in $CHCl_3$ (dry, 5 mL) at 0 °C was added EDCI (0.1186 g, 0.97 mmol, 0.97 eq.) as a solid. After 30 minutes, pyridin-3-methanol (0.127 g, 1.2 mmol, 1.2 eq.) in $CHCl_3$ (dry, 1 mL) was added via syringe. The reaction mixture was stirred for 18 h, allowing to warm to rt. The organic phase was washed with sat. aq. $NaHCO_3$ (10 mL) and $H_2O$ (10 mL), dried ($MgSO_4$) and the solvent removed *in vacuo*. After purification by column chromatography on silica gel (step gradient 0% to 17.5% acetone in $CH_2Cl_2$ in 2.5% increments) the product was obtained as a white crystalline solid (0.133 g, 53%).

**$^{1}$H NMR** (500 MHz, $CDCl_3$) δ: 9.46 (d, $J$ = 2.2 Hz, 1H, $H_k$), 8.86 (d, $J$ = 2.2 Hz, 1H, $H_f$), 8.78 (d, $J$ = 2.3 Hz, 1H, $H_a$), 8.64 (dd, $J$ = 4.8, 1.7 Hz, 1H $H_b$), 8.17 (d, $J$ = 8.4 Hz, 1H, $H_g$), 7.93 (d, $J$ = 8.2 Hz, 1H, $H_j$), 7.88-7.83 (m, 2H, $H_d$, $H_h$), 7.63 (app. t, $J$ = 7.5 Hz, 1H, $H_i$), 7.38 (dd, $J$ = 7.9, 4.8 Hz, 1H, $H_c$), 5.48 (s, 2H, $H_e$).

**$^{13}$C NMR** (126 MHz, $CDCl_3$) δ: 165.2, 150.0, 150.0 ($C_k$), 149.7 ($C_b$), 149.6 ($C_a$), 139.2 ($C_f$), 136.7 ($C_d$/$C_h$), 132.3 ($C_d$/$C_h$), 131.6, 129.6 ($C_g$), 129.3 ($C_j$), 127.8 ($C_i$), 126.9, 123.9 ($C_c$), 122.6, 64.7 ($C_e$).

**HR-ESI-MS** $m/z$ = 265.0978 $[M+H]^+$ calc. 265.0977.

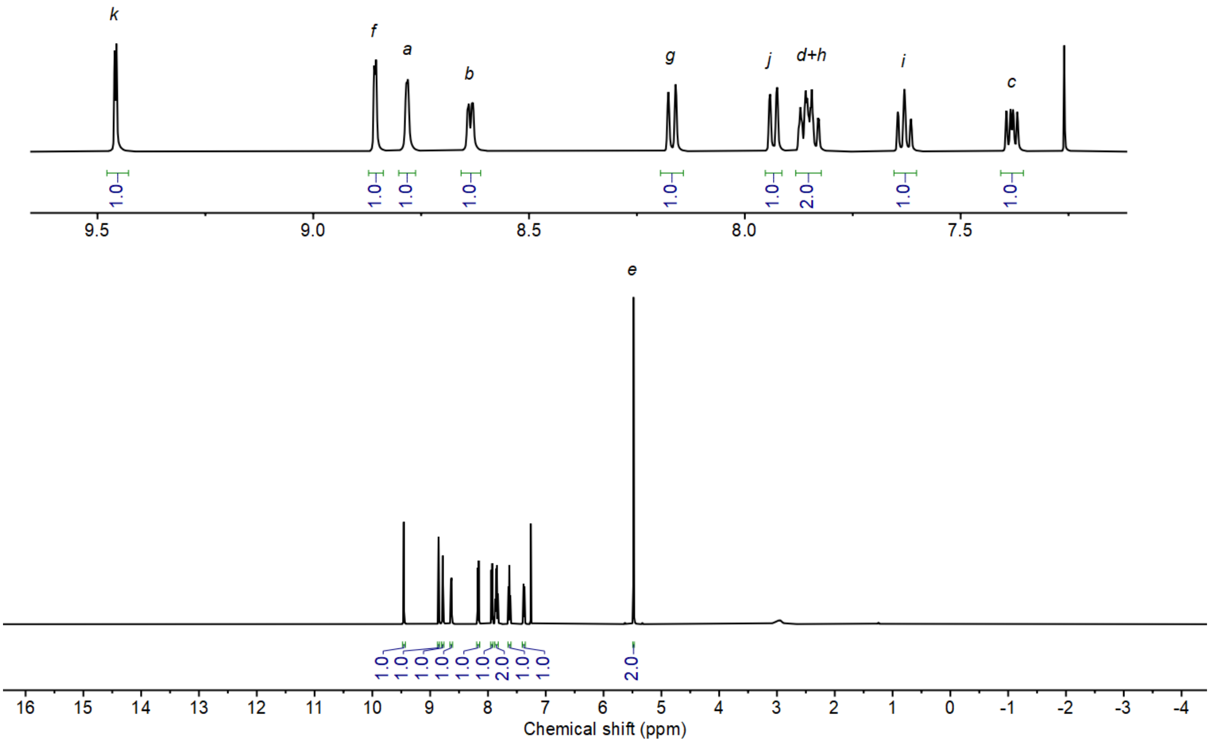


**Figure S1 $^{1}$H NMR (500 MHz, $CDCl_3$) of L1$^{Q}$.**

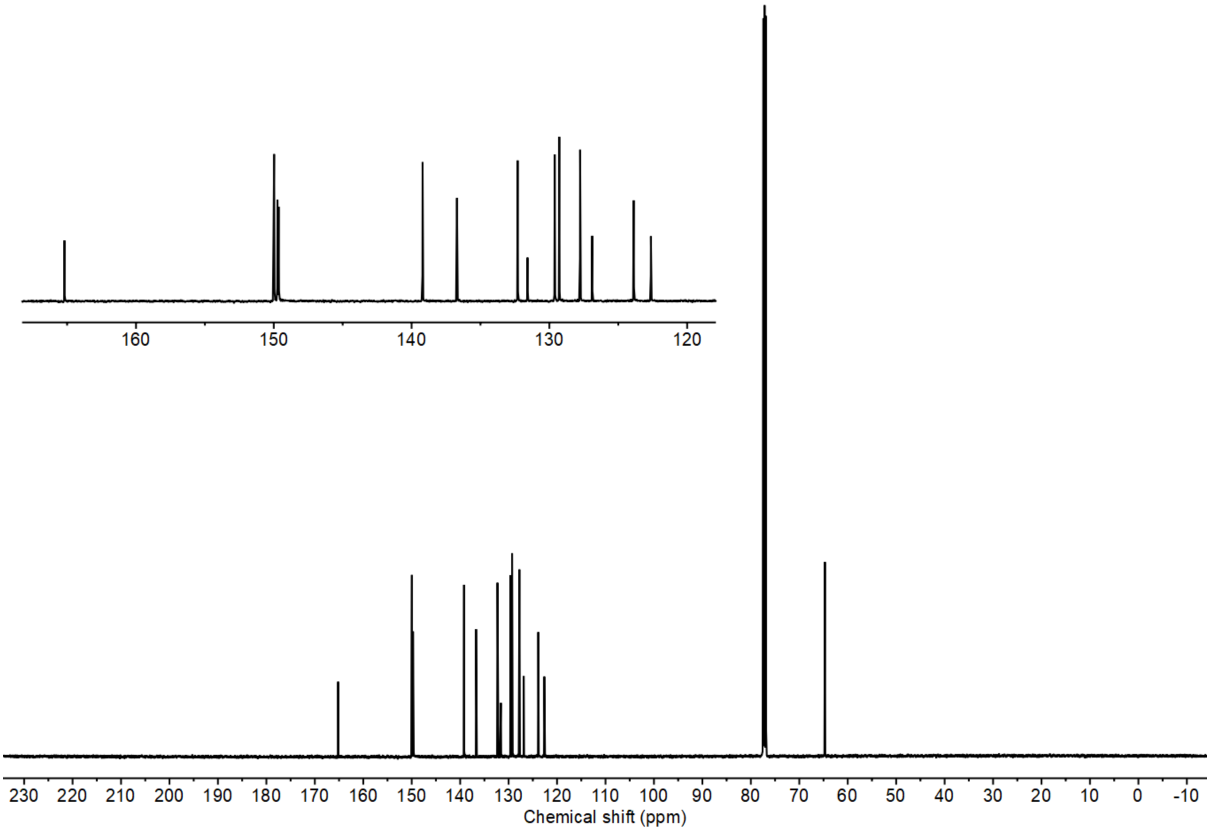


**Figure S2 $^{13}$C NMR (126 MHz, $CDCl_3$) of L1$^{Q}$.**

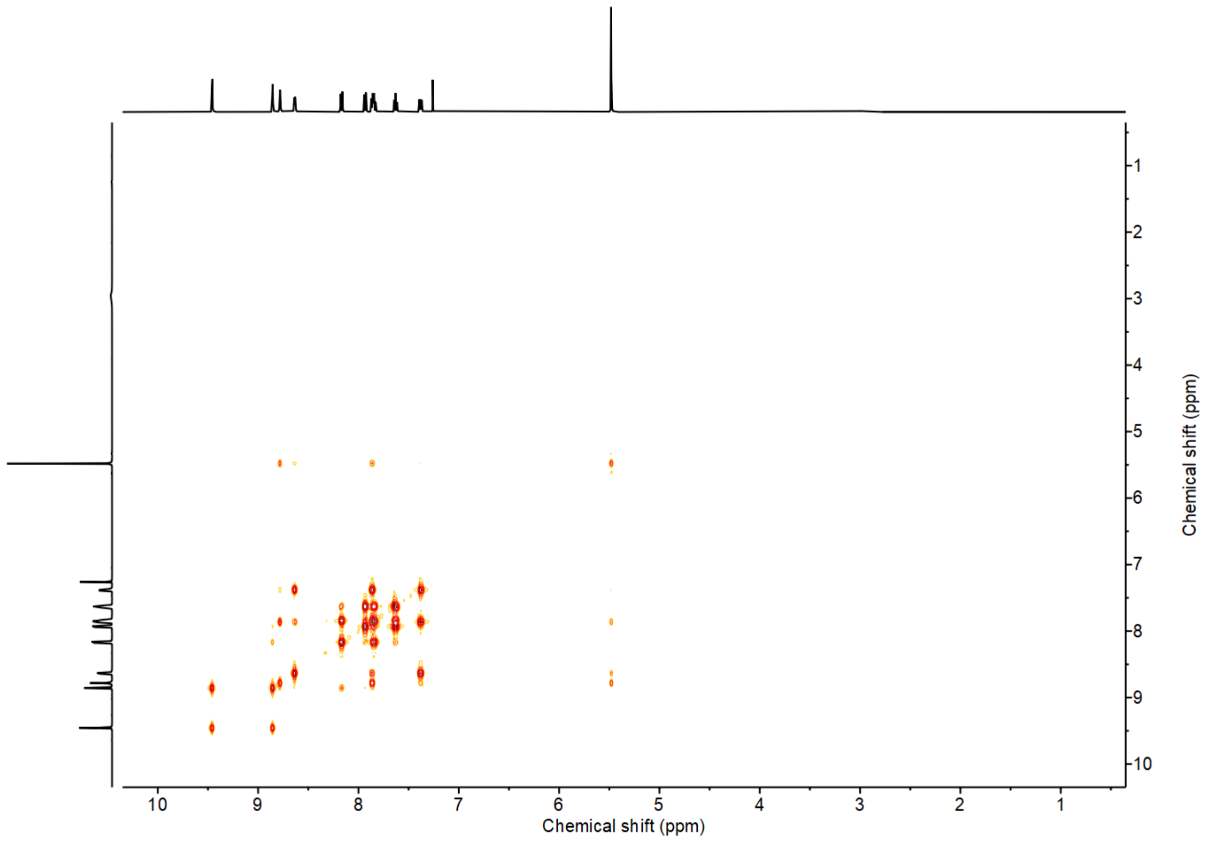


**Figure S3 COSY ($CDCl_3$) of L1[Q].**

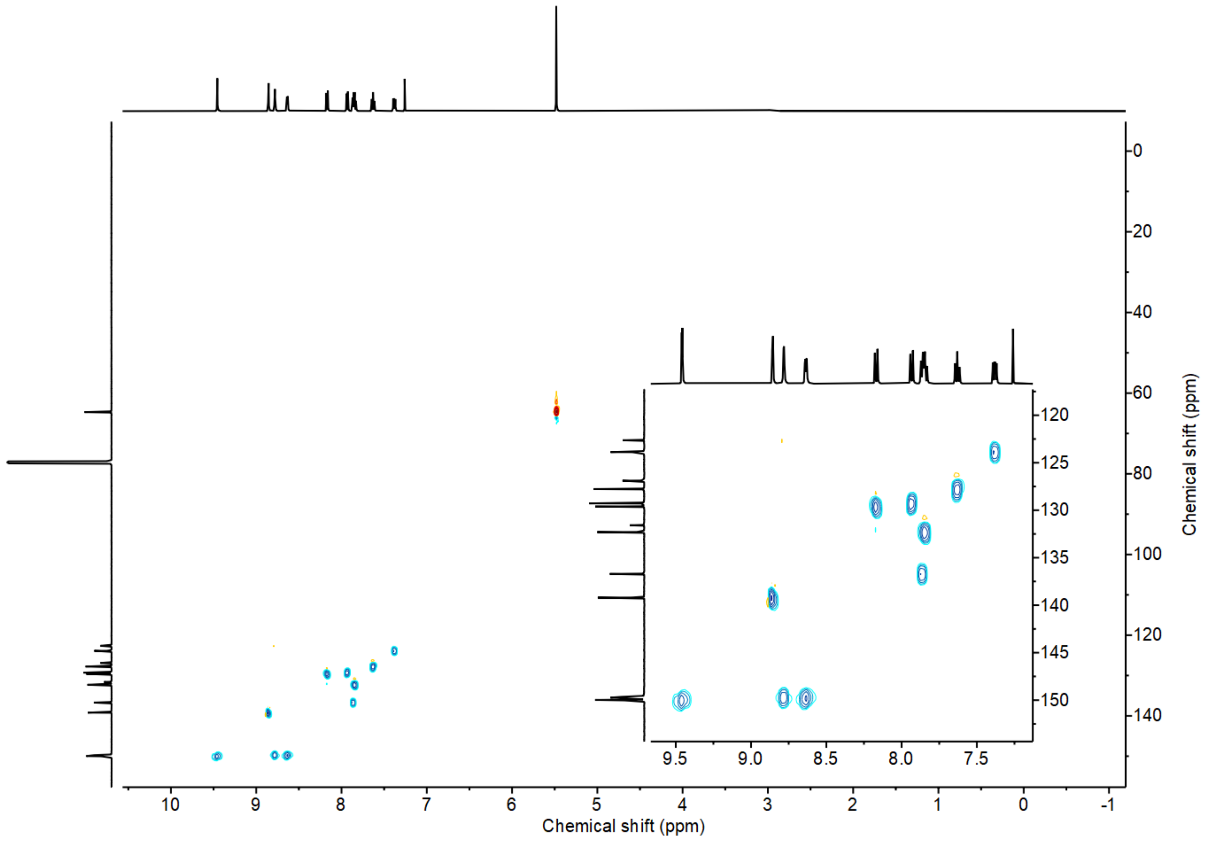


**Figure S4 HSQC ($CDCl_3$) of L1[Q].**

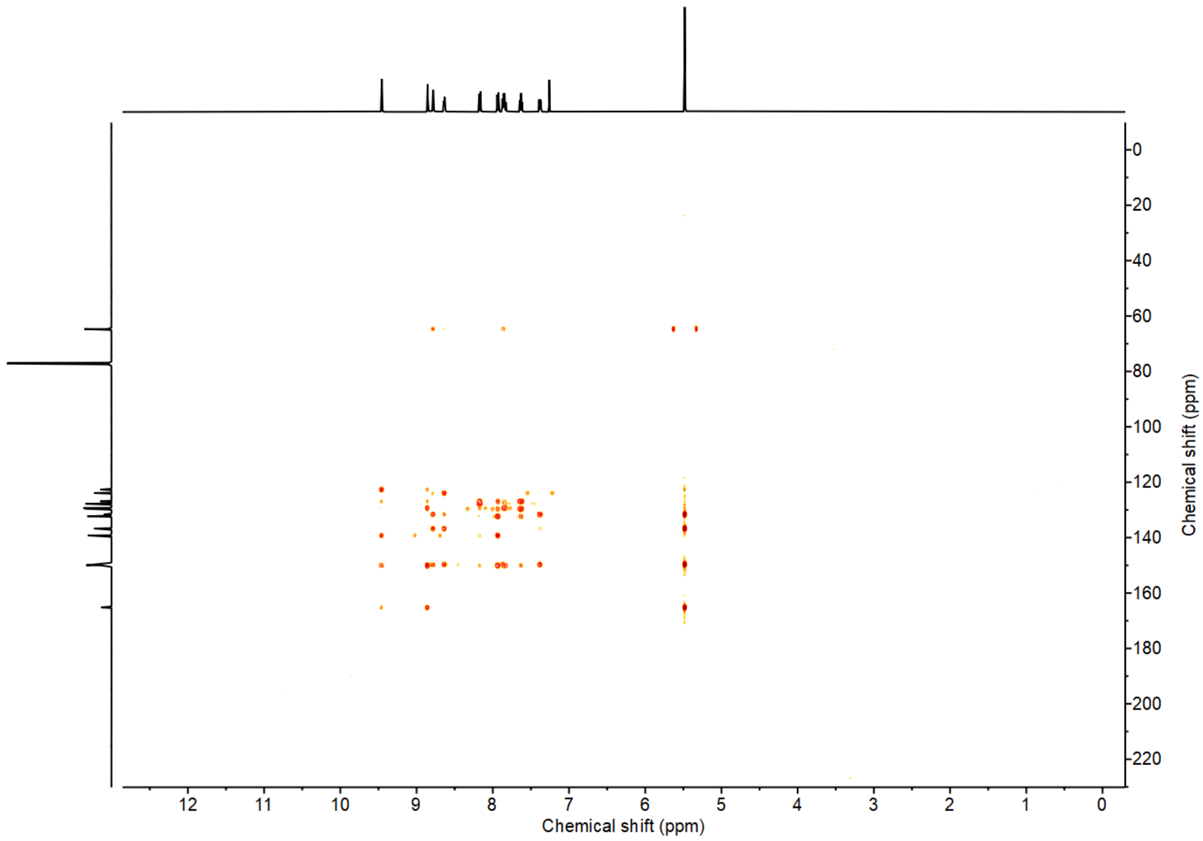


**Figure S5 HMBC ($CDCl_3$) of L1[Q].**

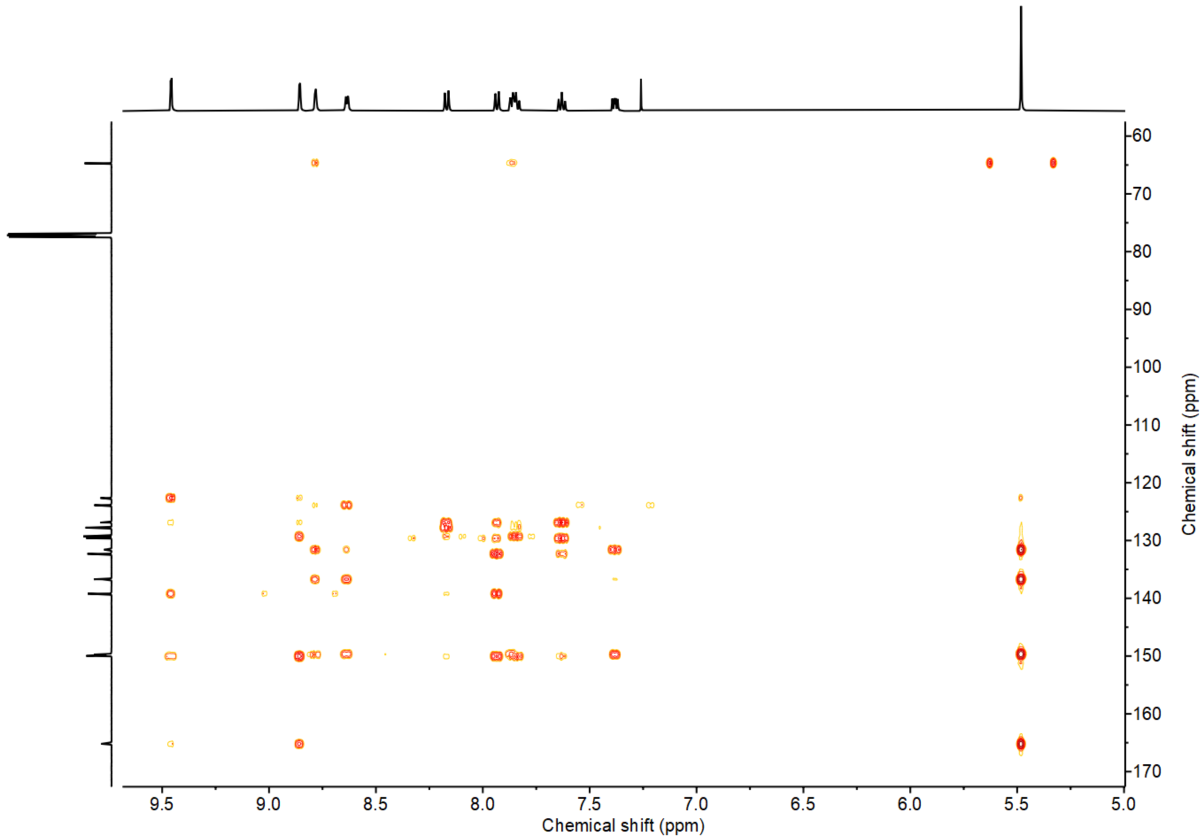


**Figure S6 Partial HMBC ($CDCl_3$) of L1[Q].**

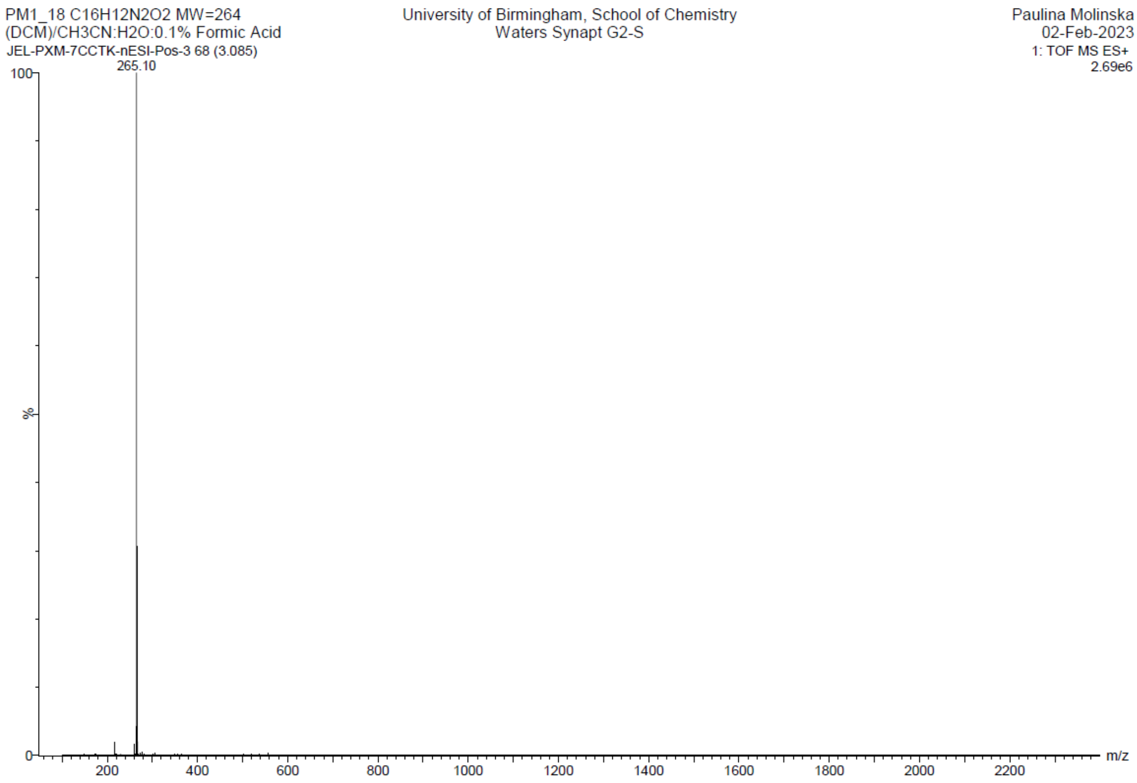


**Figure S7 ESI-MS of L1$^{Q}$.**

## S2.2 Synthesis of *cis*-C1$^{Q}$

**L1$^{Q}$** (10.5 mg, 40 μmol, 2 eq.) and $Pd(NO_3)_2 \cdot 2H_2O$ (5.3 mg, 20 μmol, 1 eq.) were sonicated in $d_6$-DMSO (1.0 mL) until a homogenous solution was obtained. After standing at 50 °C for 24 h, no further changes were observed by $^{1}H$ NMR, and *cis*-**C1$^{Q}$** was formed as the major product.

Only peaks for major species are reported.

**$^{1}$H NMR** (500 MHz, $d_6$-DMSO) δ: 10.81 (d, $J$ = 1.8 Hz, 4H, $H_k$), 10.41 (d, $J$ = 8.9 Hz, 4H, $H_j$), 9.42 (d, $J$ = 1.4 Hz, 4H, $H_f$), 9.38 (s, 4H, $H_a$), 9.00 (d, $J$ = 5.8 Hz, 4H, $H_b$), 8.36 (dd, $J$ = 8.3, 1.5 Hz, 4H, $H_g$), 8.23 (ddd, $J$ = 8.7, 7.0, 1.5 Hz, 4H, $H_i$), 8.16 (d, $J$ = 8.1 Hz, 4H, $H_d$), 7.89 (t, $J$ = 7.6 Hz, 4H, $H_h$), 7.68 (dd, $J$ = 7.9, 5.9 Hz, 4H, $H_c$), 5.41 (d, $J$ = 14.8 Hz, 4H, $H_e$), 5.34 (d, $J$ = 14.5 Hz, 4H, $H_{e'}$).

**$^{13}$C NMR** (126 MHz, $d_6$-DMSO) δ: 162.8, 155.8 ($C_k$), 151.8 ($C_b$), 150.8 ($C_a$), 146.2, 145.0 ($C_f$), 140.4 ($C_d$), 135.1 ($C_i$), 134.4, 131.4 ($C_g$), 129.6 ($C_h$), 128.3, 127.2 ($C_j$), 126.1 ($C_c$), 124.5, 65.1 ($C_e$).

**Diffusion coefficient** (400 MHz, $d_6$-DMSO) $D$: 9.51 × $10^{-11}$ $m^2s^{-1}$.

**HR-ESI-MS** $m/z$ = 444.05 {[$Pd_2$(**L1$^{Q}$**)$_4$]($NO_3$)}$^{3+}$ calc. 444.05; 1456.13 {[$Pd_2$(**L1$^{Q}$**)$_4$]($NO_3$)$_3$}$^{+}$ calc. 1456.13.

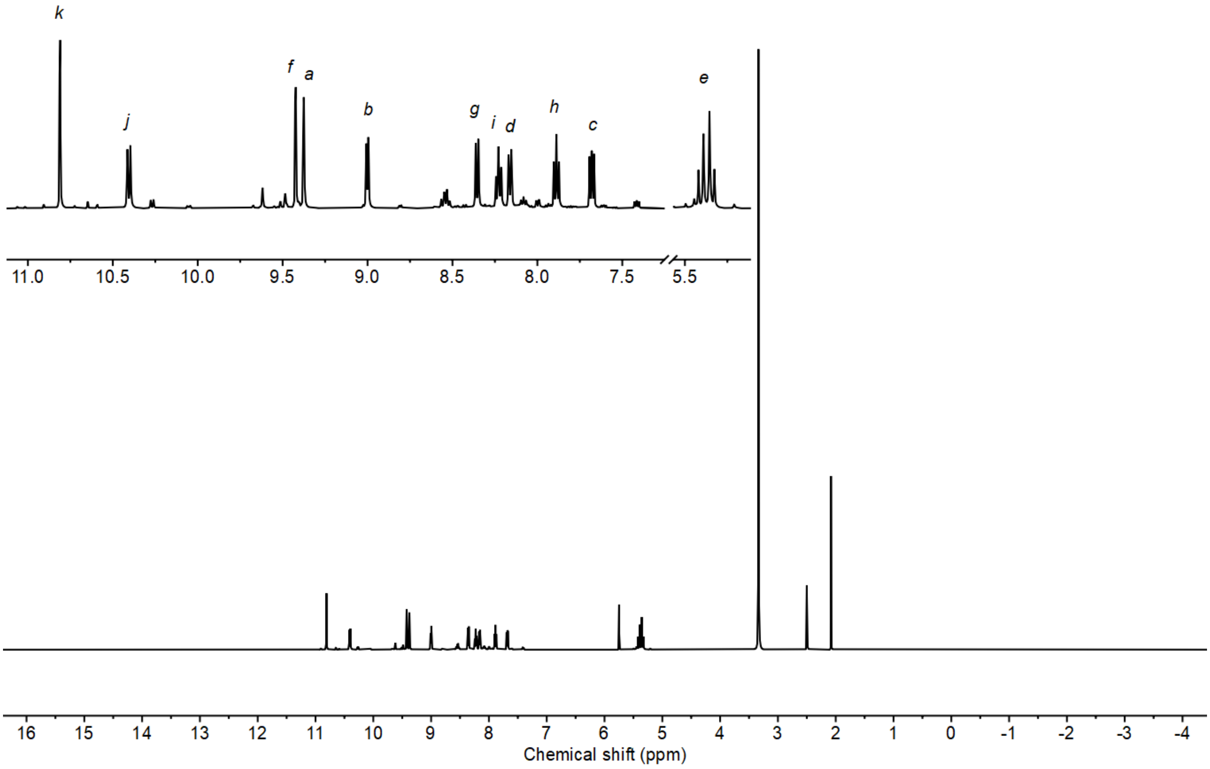


**Figure S8 $^{1}$H NMR (500 MHz, $d_6$-DMSO) of C1$^{Q}$.**

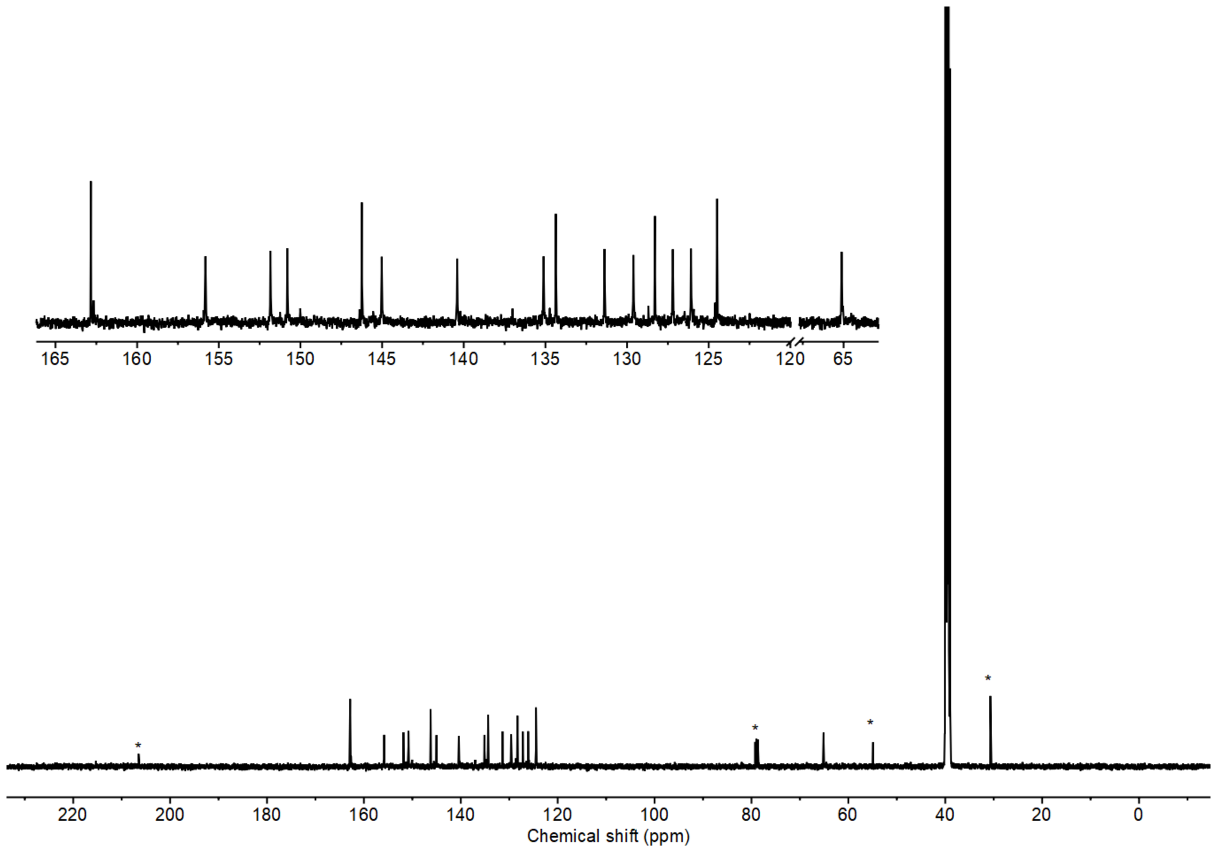


**Figure S9 $^{13}$C NMR (126 MHz, *d*$_6$-DMSO) of C1$^{Q}$.**

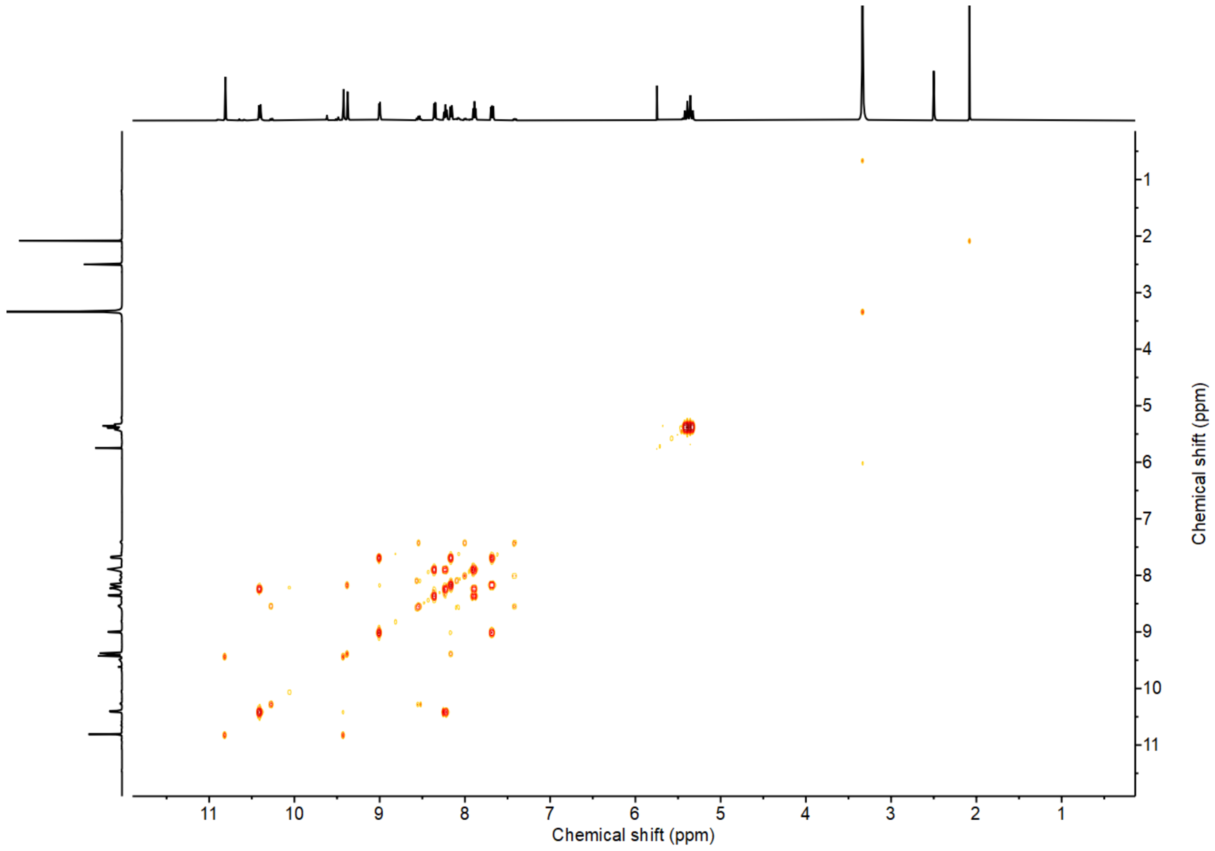


**Figure S10 COSY (*d*$_6$-DMSO) of C1$^{Q}$.**

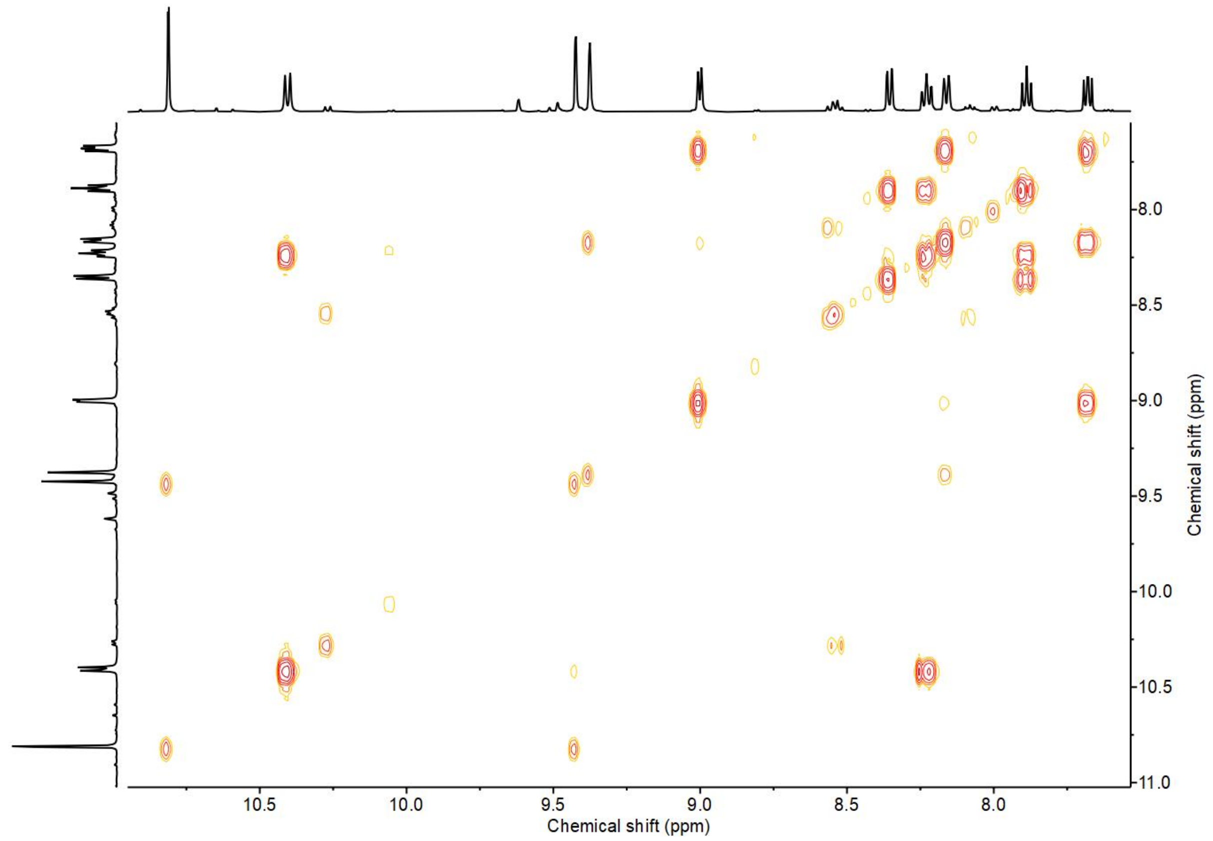


**Figure S11 Partial COSY ($d_6$-DMSO) of C1[Q].**

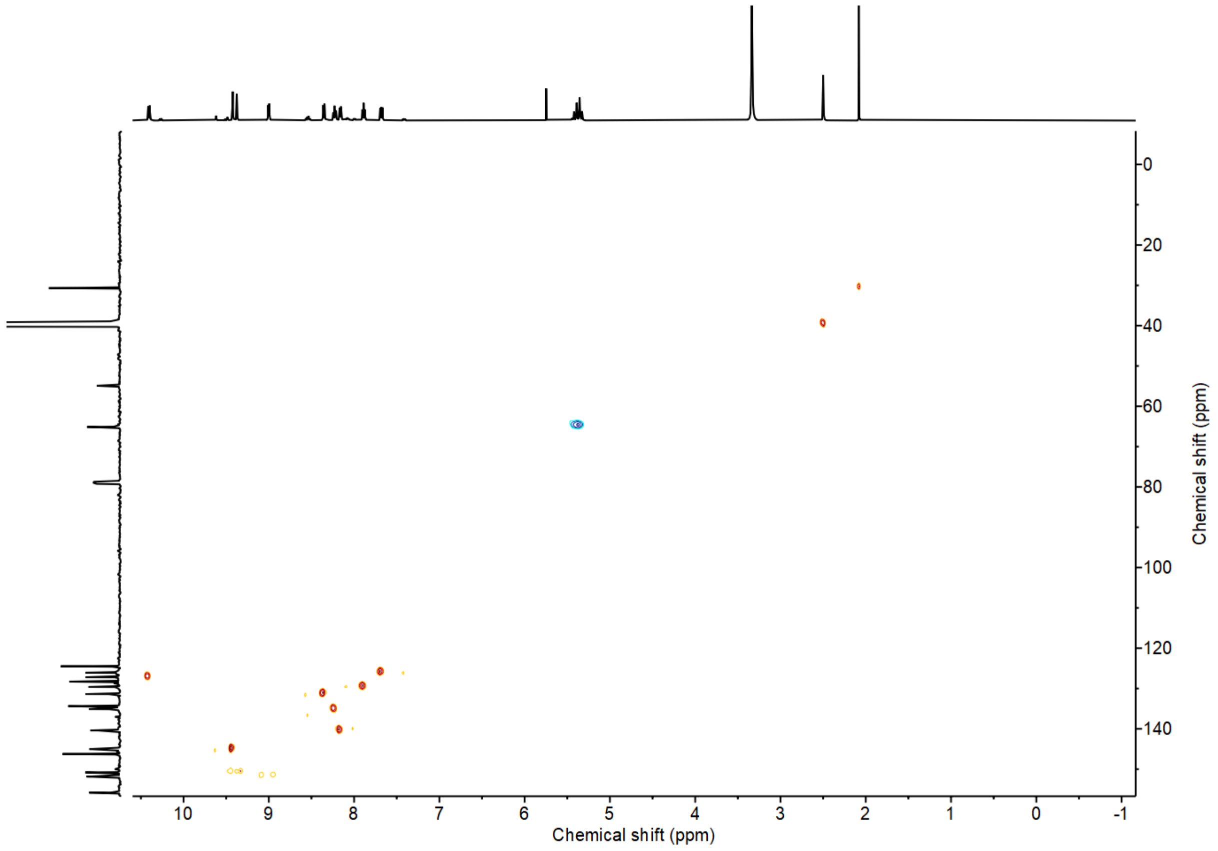


**Figure S12 HSQC ($d_6$-DMSO) of C1[Q].**

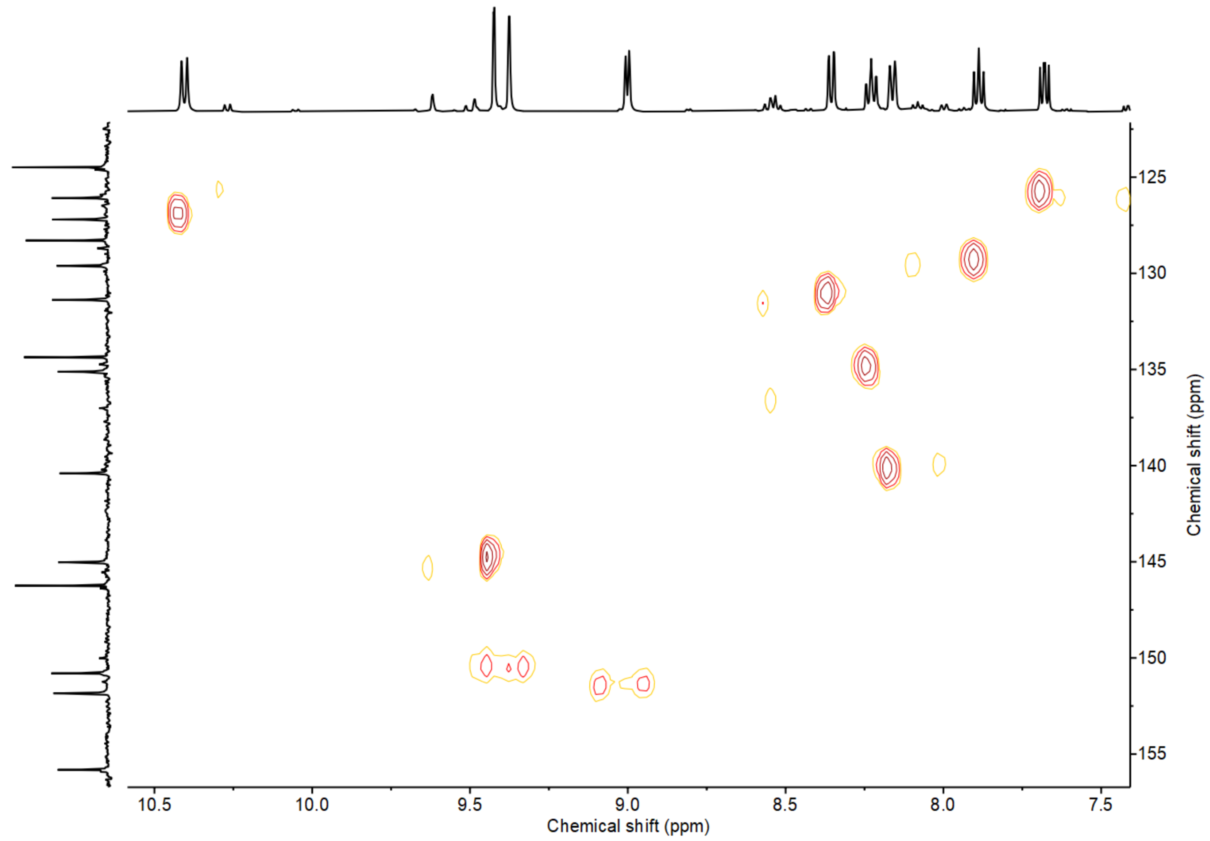


**Figure S13 Partial HSQC ($d_6$-DMSO) of C1$^{Q}$.**

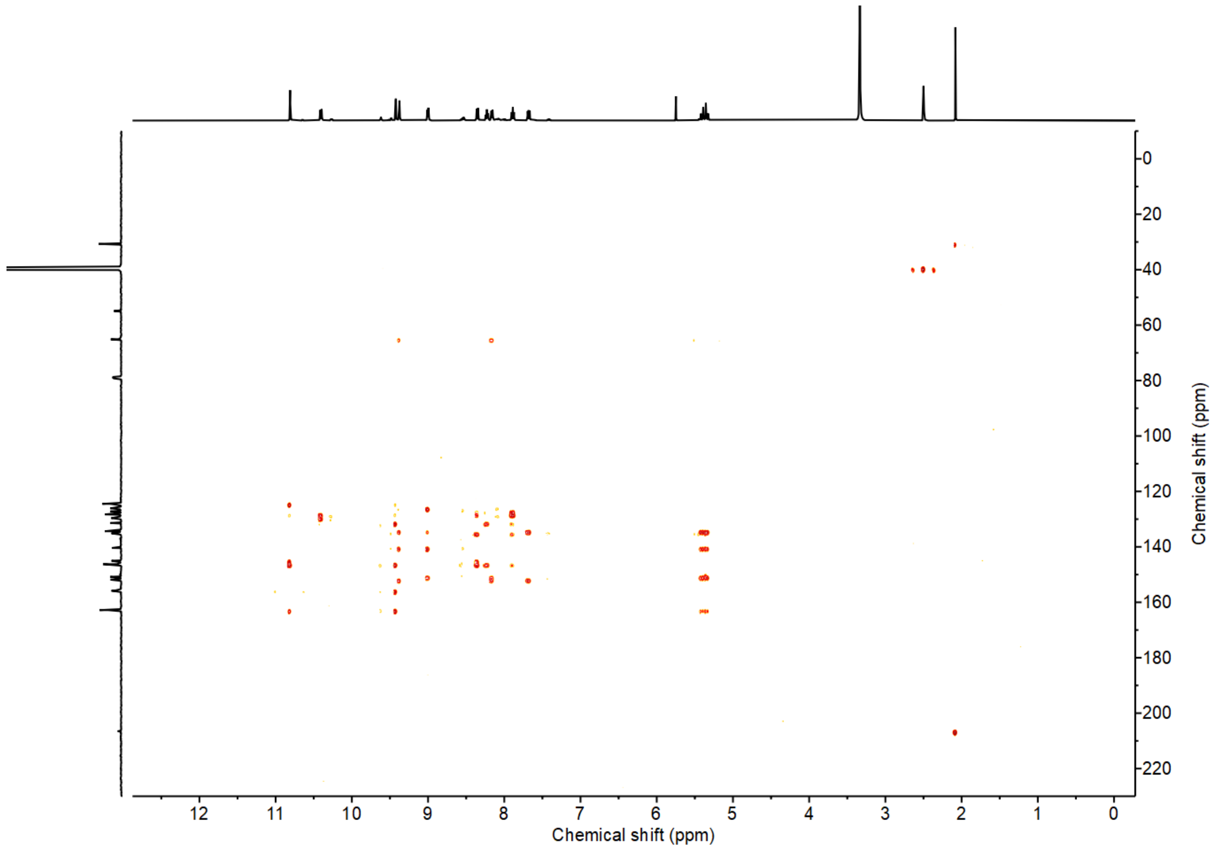


**Figure S14 HMBC ($d_6$-DMSO) of C1$^{Q}$.**

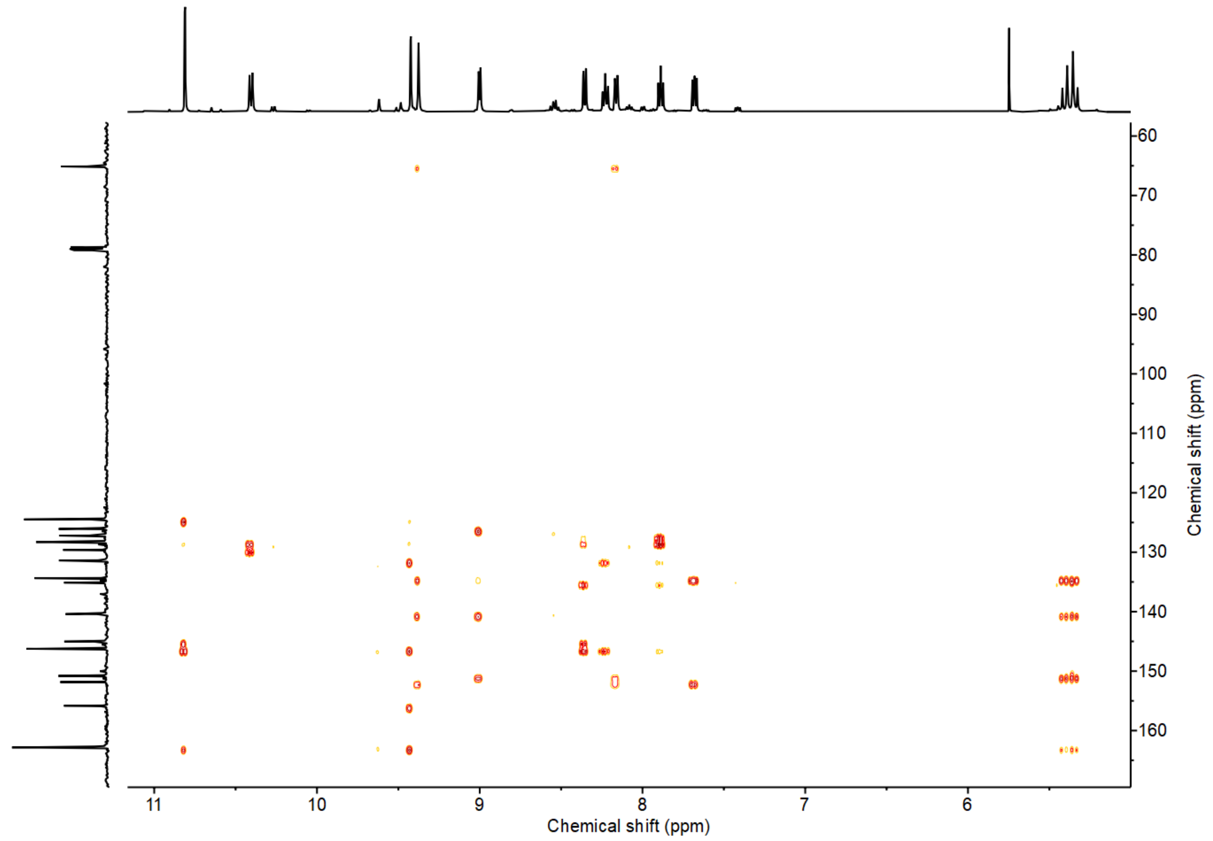


**Figure S15 Partial HMBC (*d*$_6$-DMSO) of C1$^{Q}$.**

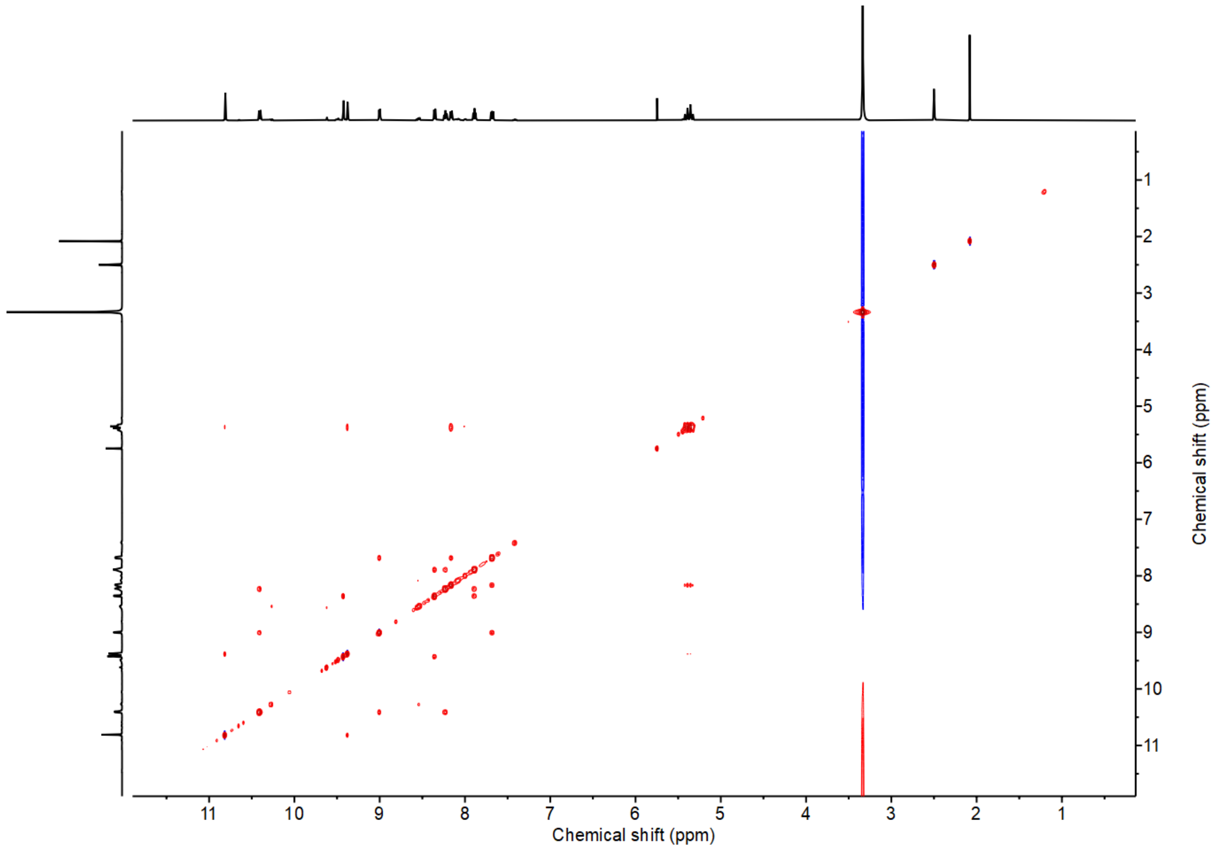


**Figure S16 NOESY (*d*$_6$-DMSO) of C1$^{Q}$.**

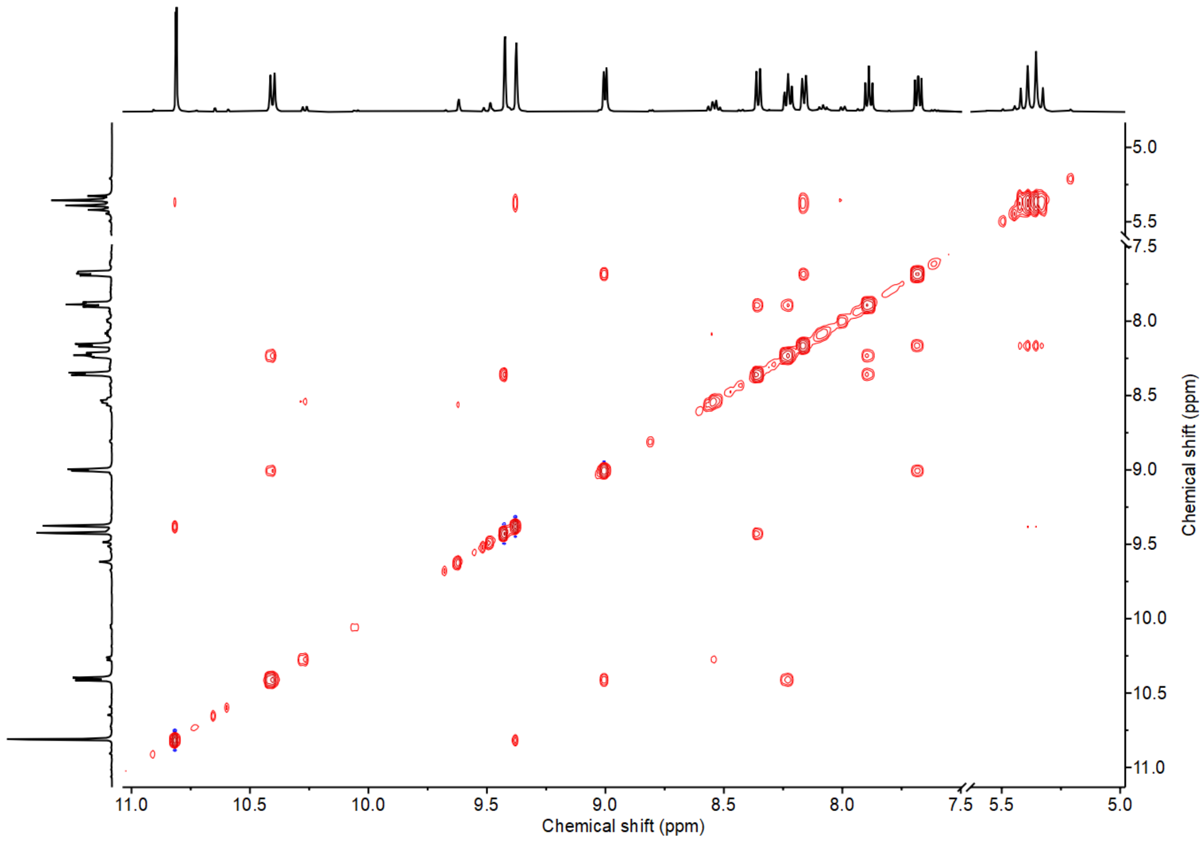


**Figure S17 Partial NOESY ($d_6$-DMSO) of C1$^{Q}$.**

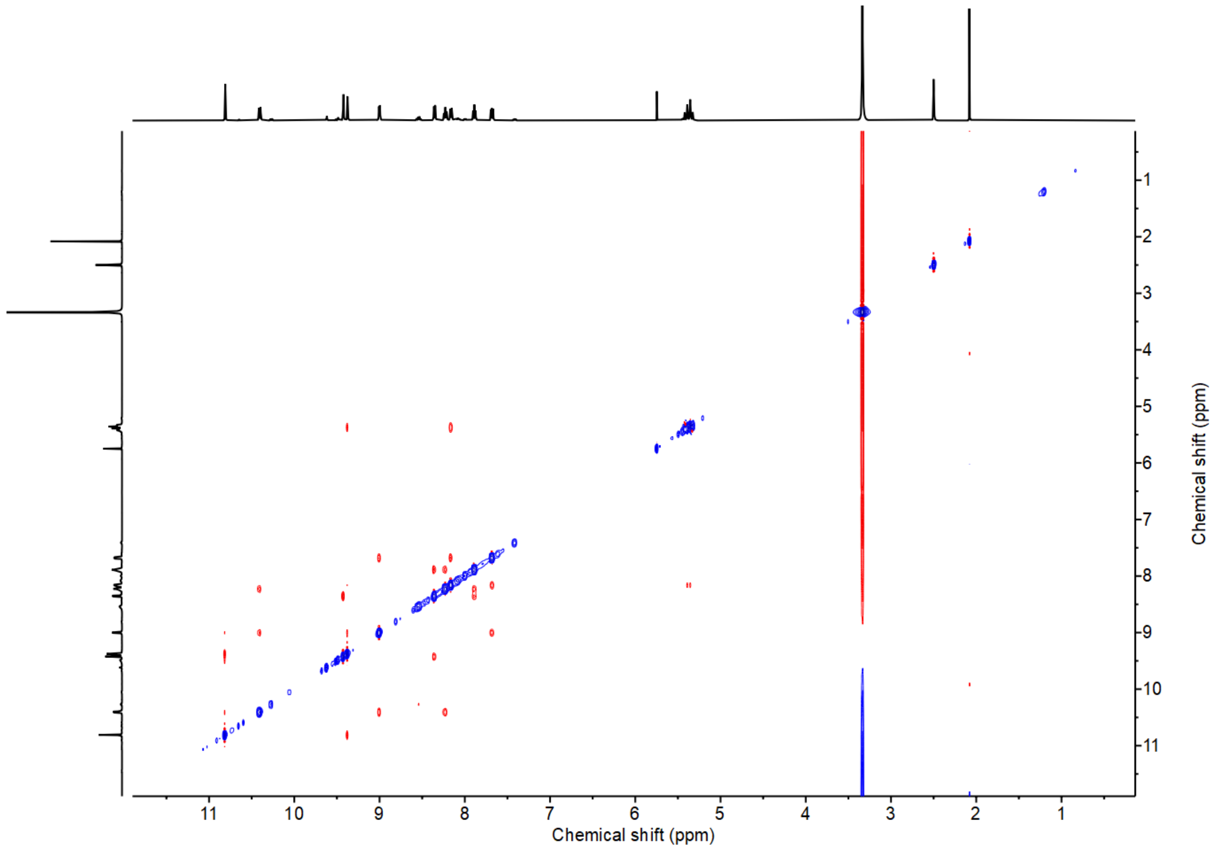


**Figure S18 ROESY ($d_6$-DMSO) of C1$^{Q}$.**

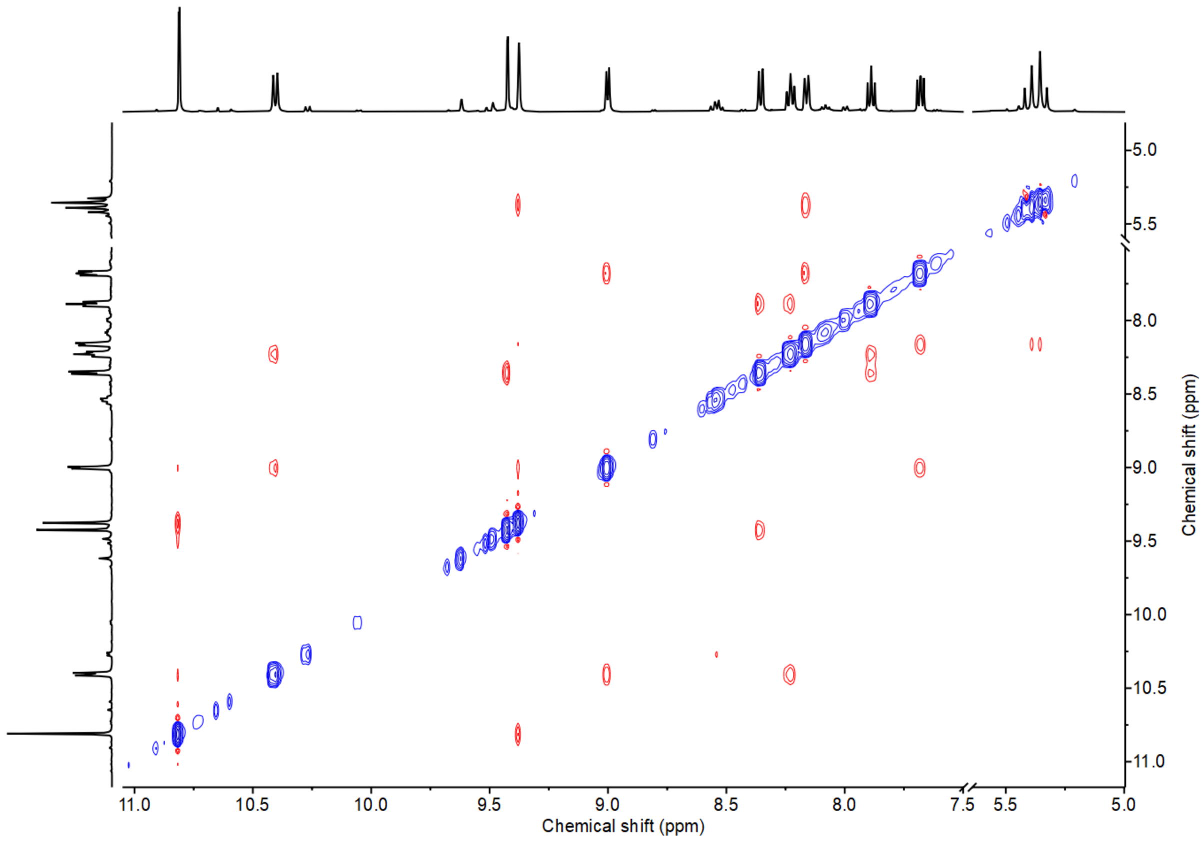


**Figure S19 Partial ROESY ($d_6$-DMSO) of C1$^{Q}$.**

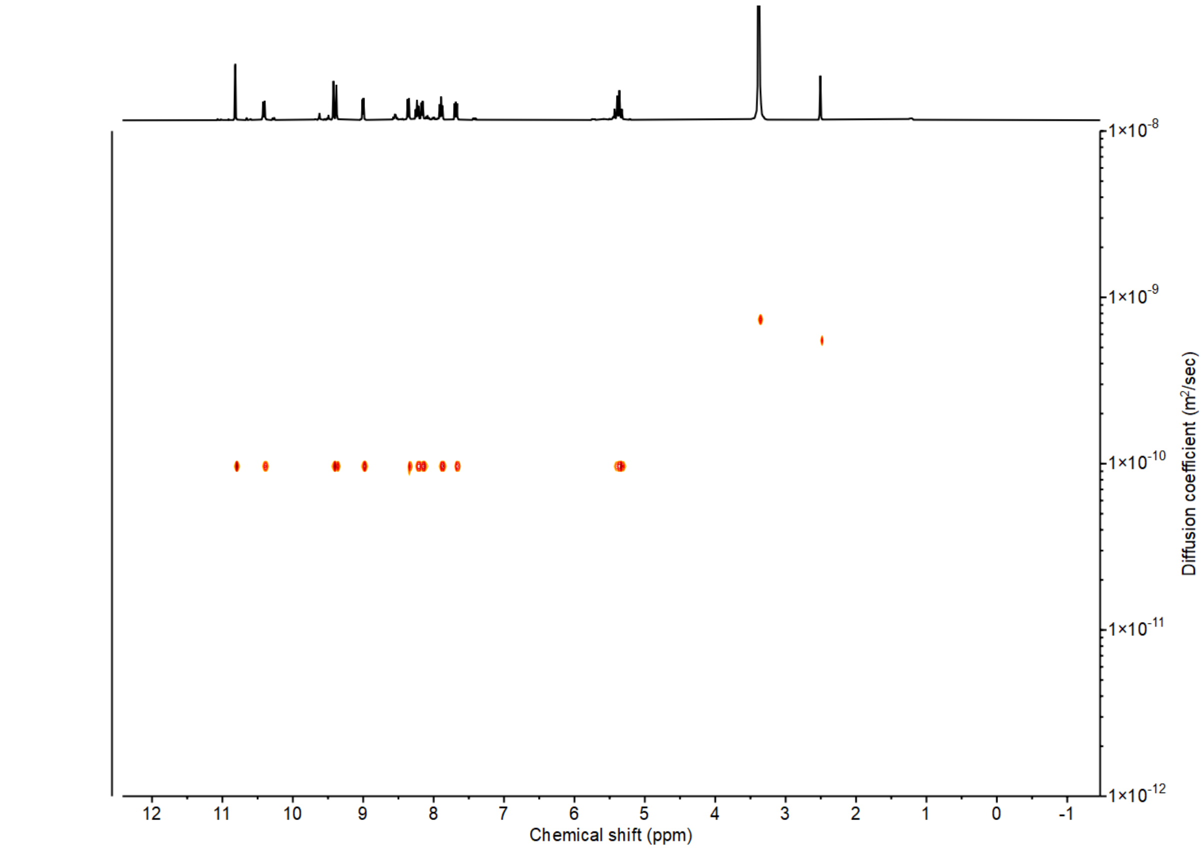


**Figure S20 DOSY ($d_6$-DMSO, 400 MHz) of C1$^{Q}$.**

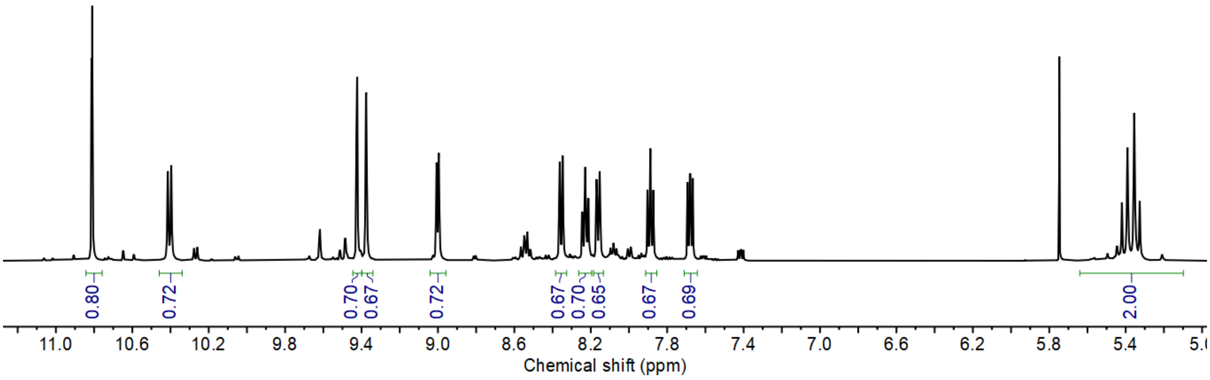


**Figure S21 Partial $^{1}$H NMR (500 MHz, *d*$_6$-DMSO) of C1$^{Q}$ with integrals relative to sum total of $CH_2$ signals.**

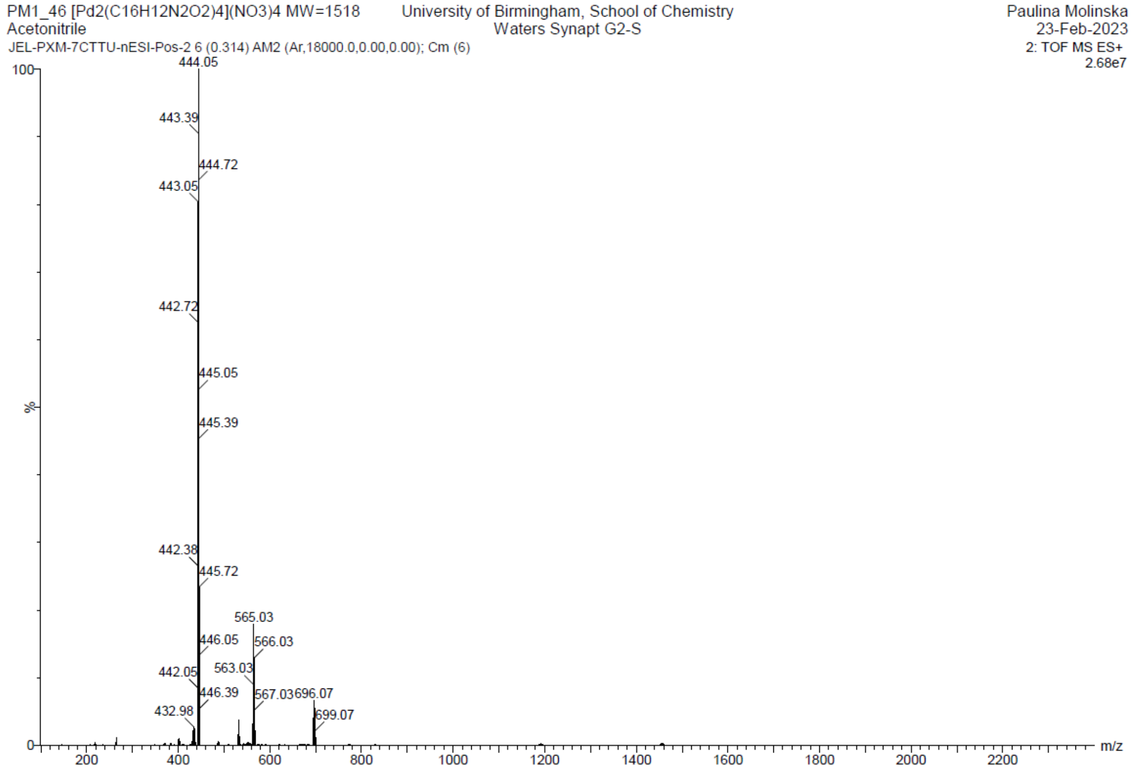


**Figure S22 ESI-MS of C1$^{Q}$.**

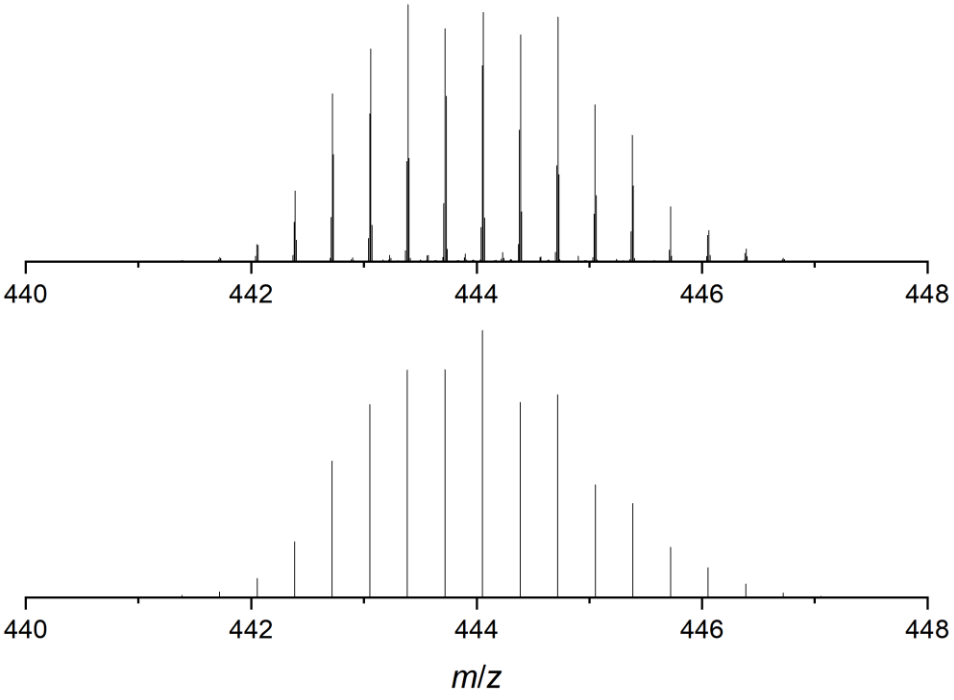


**Figure S23 Observed (top) and calculated (bottom) isotopic patterns for {[$Pd_2(L1^{Q})_4$]($NO_3$)}$^{3+}$.**

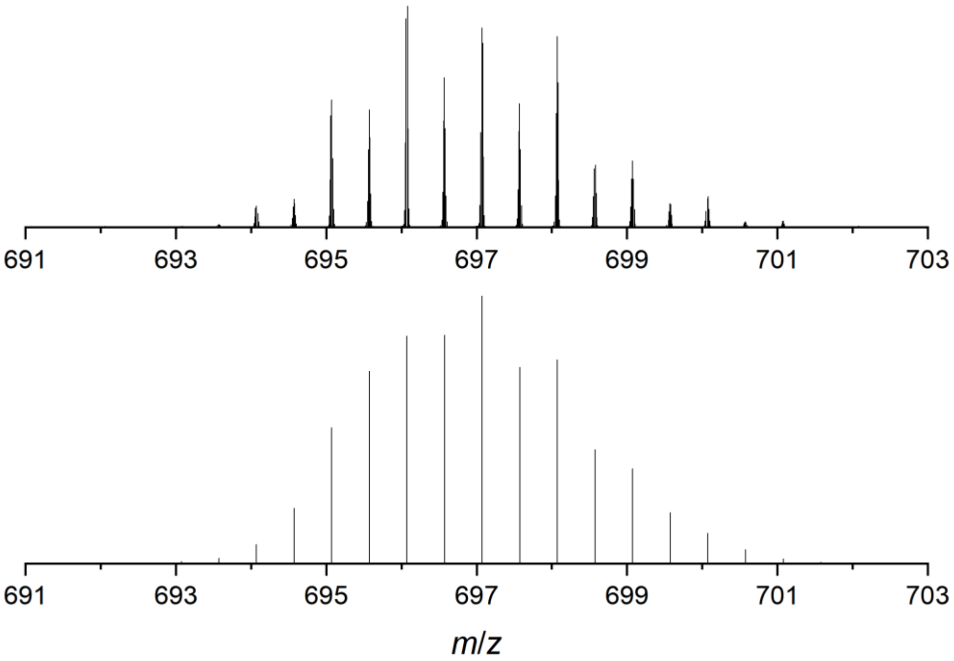


**Figure S24 Observed (top) and calculated (bottom) isotopic patterns for {[$Pd_2(L1^{Q})_4$]$(NO_3)_2$}$^{2+}$.**

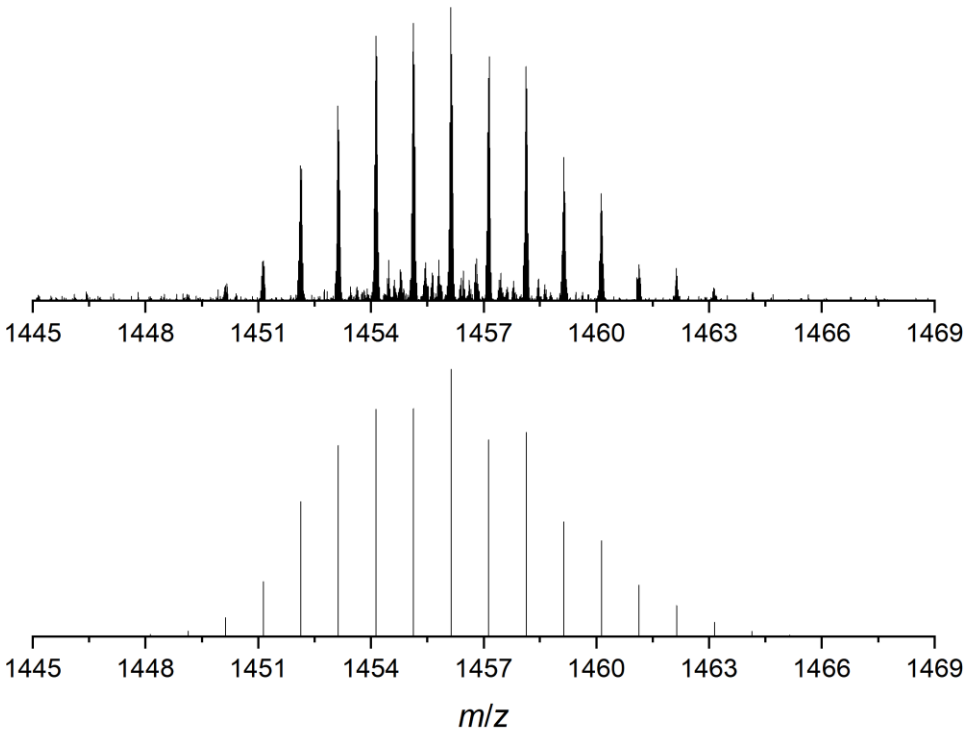


**Figure S25 Observed (top) and calculated (bottom) isotopic patterns for $\{[Pd_2(L1^{Q})_4](NO_3)_3\}^+$.**

## S2.3 Identification of minor *trans*-C1^Q isomer

**$^1$H NMR** (500 MHz, $d_6$-DMSO) δ: 10.81 (4H, $H_k$), 10.27 (d, $J$ = 8.7 Hz, 4H, $H_j$), 9.62 (s, 4H, $H_f$), 9.49 (s, 4H, $H_a$), 8.57-8.51 (m, 12H, $H_b$, $H_g$, $H_i$), 8.08 (app. T, $J$ = 7.6 Hz, 4H, $H_h$), 8.00 (dd, $J$ = 8.1, 1.6 Hz, 4H, $H_d$), 7.41 (dd, $J$ = 7.9, 5.8 Hz, 4H, $H_c$), 5.36 (from COSY; 8H, $H_e$).

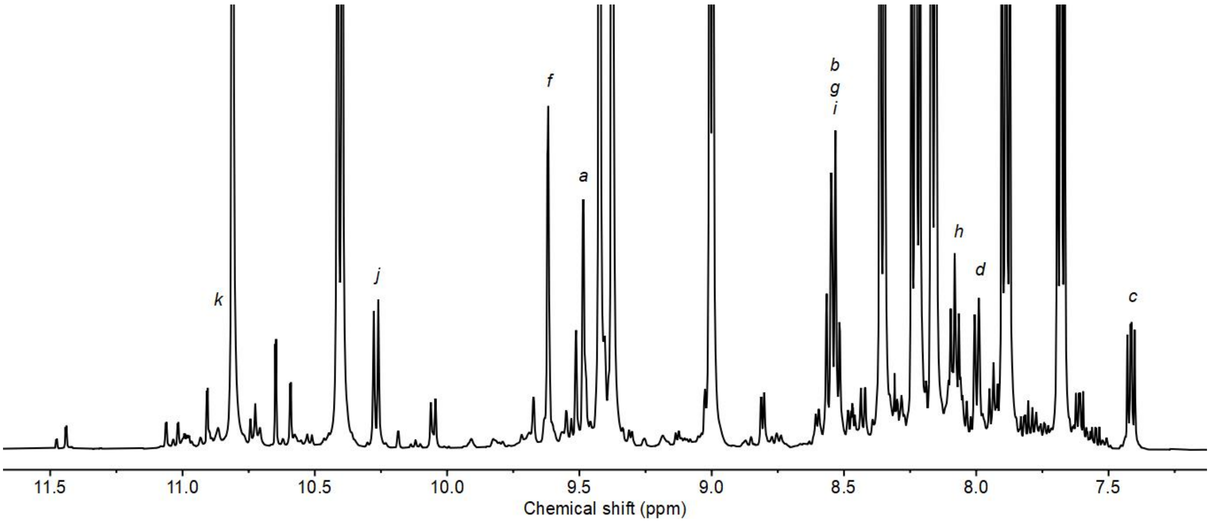


**Figure S26** Partial $^1$H NMR (500 MHz, $d_6$-DMSO) of C1^Q with peaks assigned to minor *trans* isomer labelled.

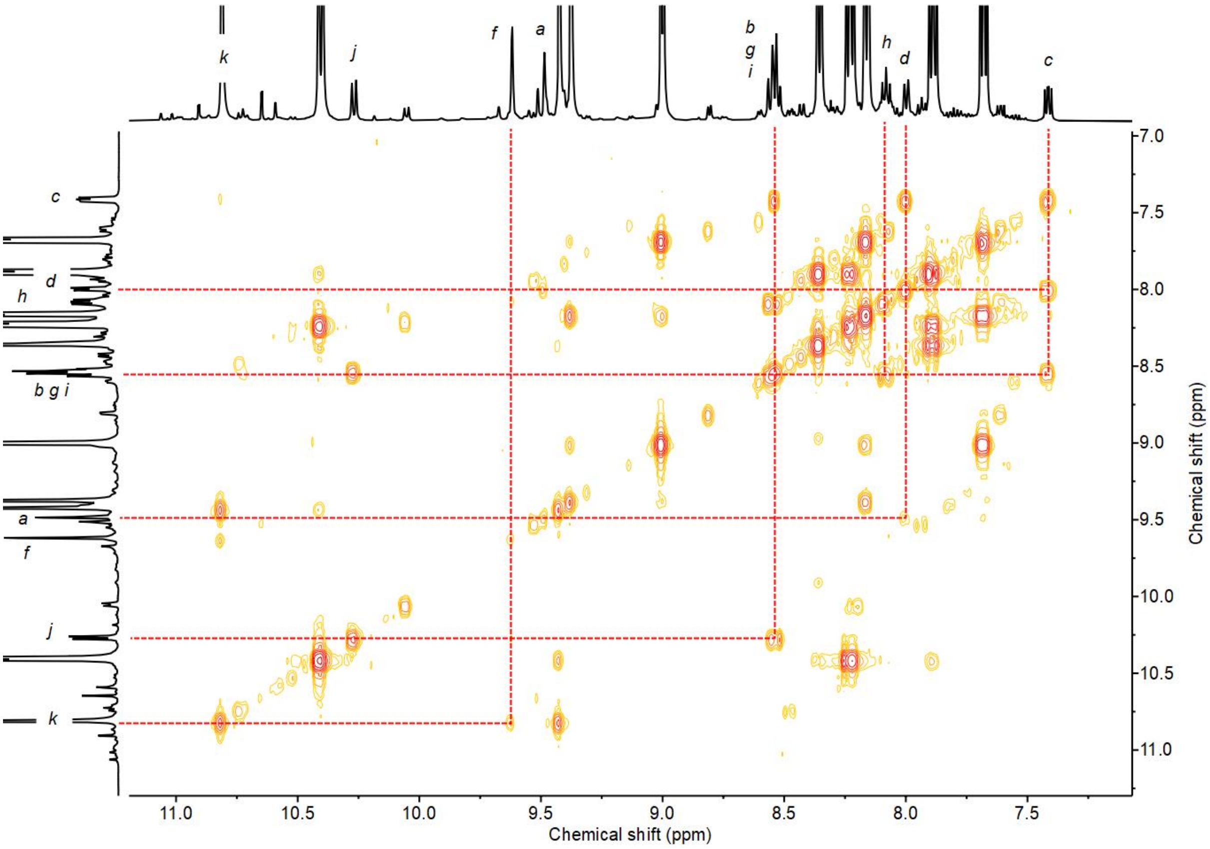


**Figure S27** Partial COSY ($d_6$-DMSO) of C1^Q with peaks assigned to minor *trans* isomer labelled.

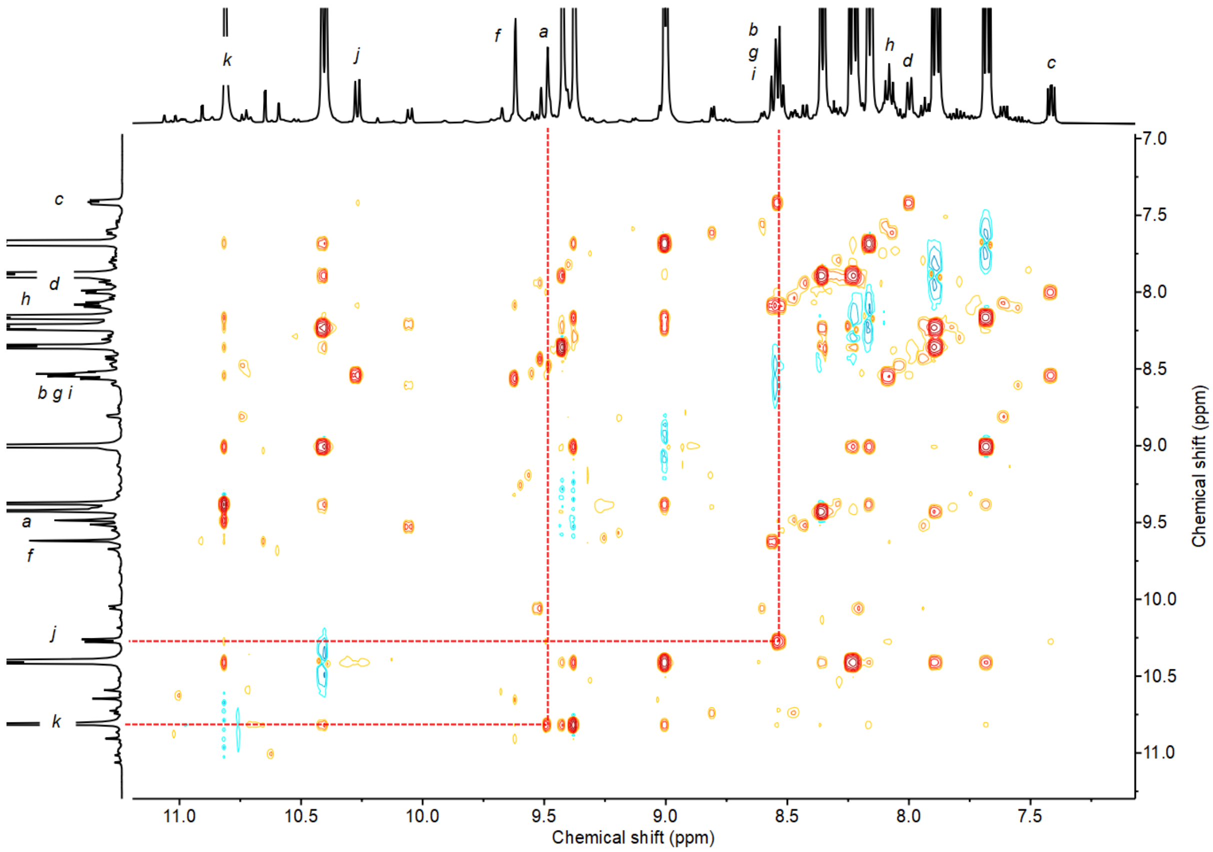


**Figure S28 Partial NOESY ($d_6$-DMSO) with diagonal supression of C1Q with peaks assigned to minor *trans* isomer labelled.**

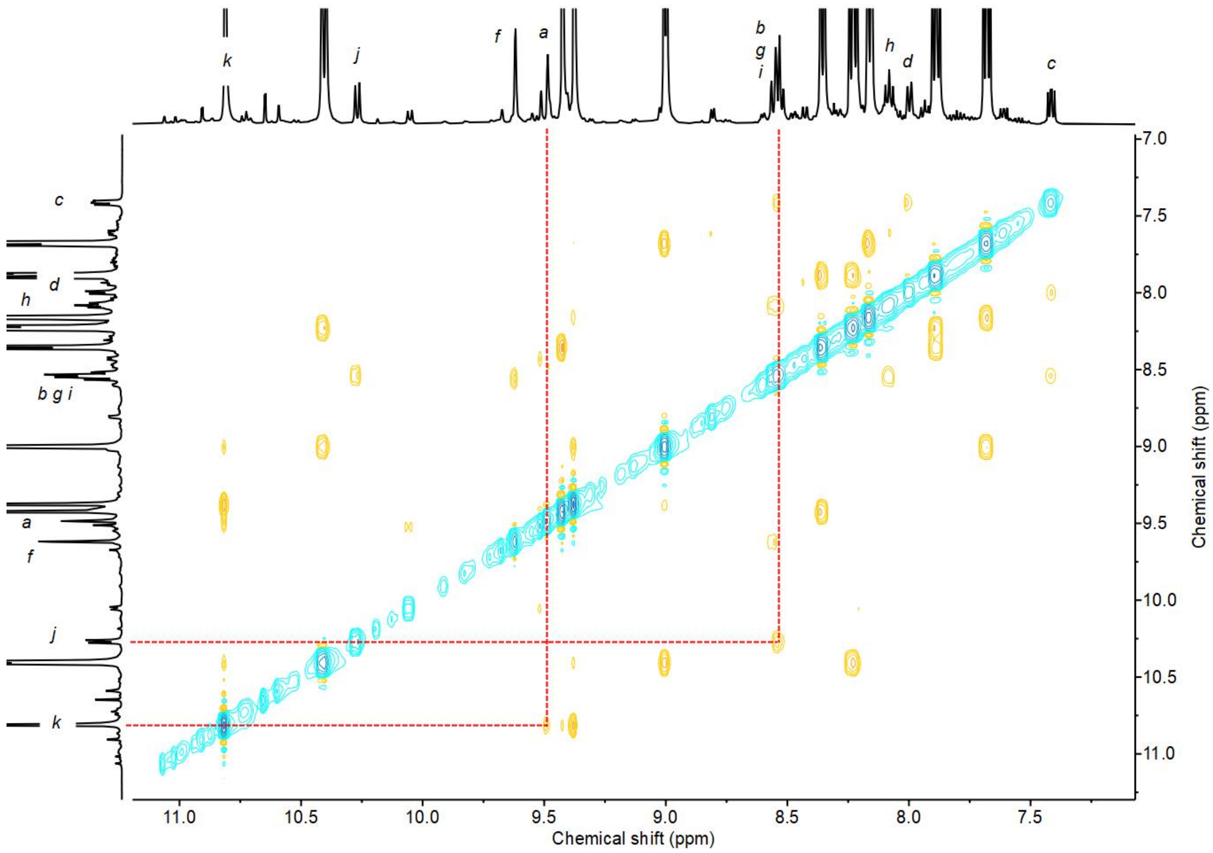


**Figure S29 Partial ROESY ($d_6$-DMSO) of C1Q with peaks assigned to minor *trans* isomer labelled.**

## S2.4 *cis*-C1$^{Q}$ + $Bu_4NCl$

**L1$^{Q}$** (7.9 mg, 30 μmol, 4 eq.) and $Pd(NO_3)_2 \cdot 2H_2O$ (4.0 mg, 15 μmol, 2 eq.) were sonicated in a 40 mM solution of 1,3,5-trimethoxybenzene in $d_6$-DMSO (0.75 mL) until a homogenous solution was obtained After standing at 50 °C for 48 h, a 100 mM solution of $Bu_4NCl$ in $d_6$-DMSO (75 μL, 7.5 μmol, 1 eq.) was added.

Only peaks for major species are reported.

**$^{1}$H NMR** (400 MHz, $CDCl_3$) δ: 11.45 (d, *J* = 1.9 Hz, 4H, $H_k$), 10.53 (d, *J* = 8.8 Hz, 4H, $H_j$), 10.21 (d, *J* = 1.9 Hz, 4H, $H_a$), 9.39 (d, *J* = 2.0 Hz, 4H, $H_f$), 9.32 (d, *J* = 5.7 Hz, 4H, $H_b$), 8.35 (dd, *J* = 8.4, 1.5 Hz, 4H, $H_g$), 8.29 (ddd, *J* = 8.7, 7.0, 1.5 Hz, 4H, $H_i$), 8.10 (dd, *J* = 8.3, 1.7 Hz, 4H, $H_d$), 7.91 (m, 4H, $H_h$), 7.75 (dd, *J* = 8.0, 5.7 Hz, 4H, $H_c$), 5.43 (d, *J* = 15.6 Hz, 4H, $H_e$), 5.34 (d, *J* = 15.7 Hz, 4H, $H_{e'}$).

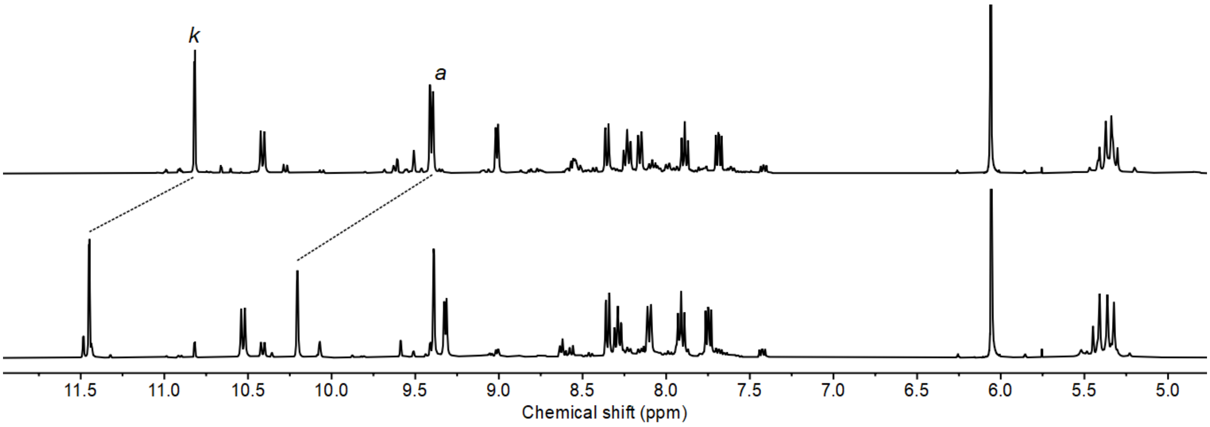


**Figure S30 $^{1}$H NMR (400 MHz, $d_6$-DMSO) of C1$^{Q}$ (top) and C1$^{Q}$ + 1 eq. $Bu_4NCl$ (bottom). Δδ: $H_k$ = 0.63, $H_a$ = 0.82.**

## S2.5 Synthesis of *cis*-$C1^{Q}$⊃Cl

**$L1^{Q}$** (10.6 mg, 40 μmol, 4 eq.), $[Pd(CH_3CN)_4](BF_4)_2$ (8.9 mg, 20 μmol, 2 eq.) and $Bu_4NCl$ (2.8 mg, 10 μmol, 1 eq.) were sonicated in *d*$_6$-DMSO (0.75 mL) until a homogenous solution was obtained. After standing at 60 °C for 4 h, the solution was diluted with DMF (1 mL) and filtered through celite. Vapour diffusion of $Et_2O$ into this solution yielded a precipitate. After the mother liquor was decanted, the solid was washed with $Et_2O$ (× 3) and dried *in vacuo* to give the product as a beige solid (11.8 mg, 75%).

$^{1}$H NMR analysis suggested that, whilst *cis*-$C1^{Q}$ remained the dominant isomer (~40%) with an encapsulated $Cl^{-}$ anion, a second, minor isomer appeared to increase in compositional value (to ~20%). ESI-MS exclusively showed formation of $[Pd_2($**$L1^{Q}$**$)_4$⊃$Cl]^{3+}$.

Only peaks for major species are reported.

**$^{1}$H NMR** (300 MHz, $CDCl_3$) δ: 11.44 (d, *J* = 1.9 Hz, 4H, H$_k$), 10.52 (d, *J* = 8.9 Hz, 4H, H$_j$), 10.18 (s, 4H, H$_a$), 9.41 (d, *J* = 2.0 Hz, 4H, H$_f$), 9.29 (d, *J* = 5.7 Hz, 4H, H$_b$), 8.36 (dd, *J* = 8.3, 1.4 Hz, 4H, H$_g$), 8.29 (ddd, *J* = 8.7, 7.0, 1.5 Hz, 4H, H$_i$), 8.11 (d, *J* = 7.9 Hz, 4H, H$_d$), 7.91 (t, *J* = 7.8 Hz, 4H, H$_h$), 7.74 (dd, *J* = 8.0, 5.7 Hz, 4H, H$_c$). Overlapping peaks prevented assignment of H$_e$ signal.

**ESI-MS** *m/z* = 435.06 $\{[Pd_2($**$L1^{Q}$**$)_4](Cl)\}^{3+}$ calc. 435.05.

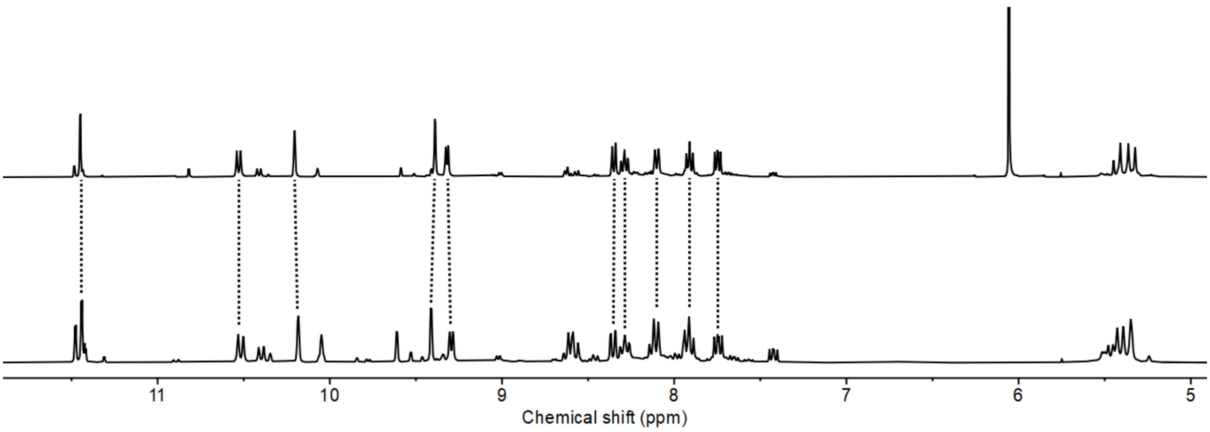


**Figure S31 Partial $^{1}$H NMR (400 MHz, *d*$_6$-DMSO) of $[Pd_2(L1^{Q})$⊃$Cl](NO_3)_3$ (top) and $^{1}$H NMR (300 MHz, *d*$_6$-DMSO) of $[Pd_2(L1^{Q})$⊃$Cl](BF_4)_3$ (bottom).**

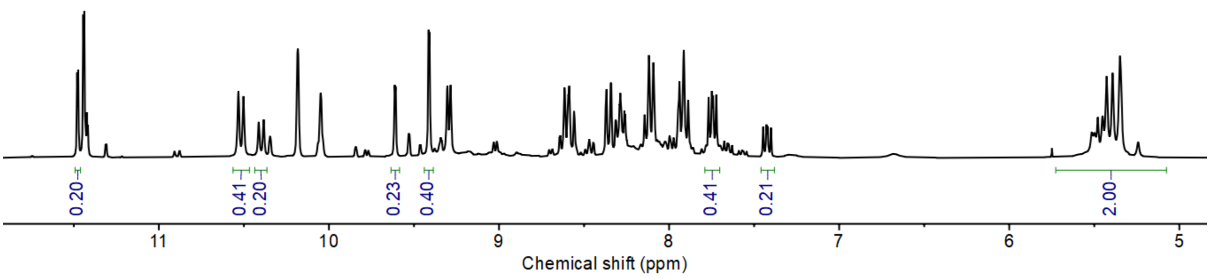


**Figure S32 Partial $^{1}$H NMR (300 MHz, *d*$_6$-DMSO) of $[Pd_2(L1^{Q})$⊃$Cl](BF_4)_3$ with integrals for two major isomers in comparison to total $CH_2$ signal.**

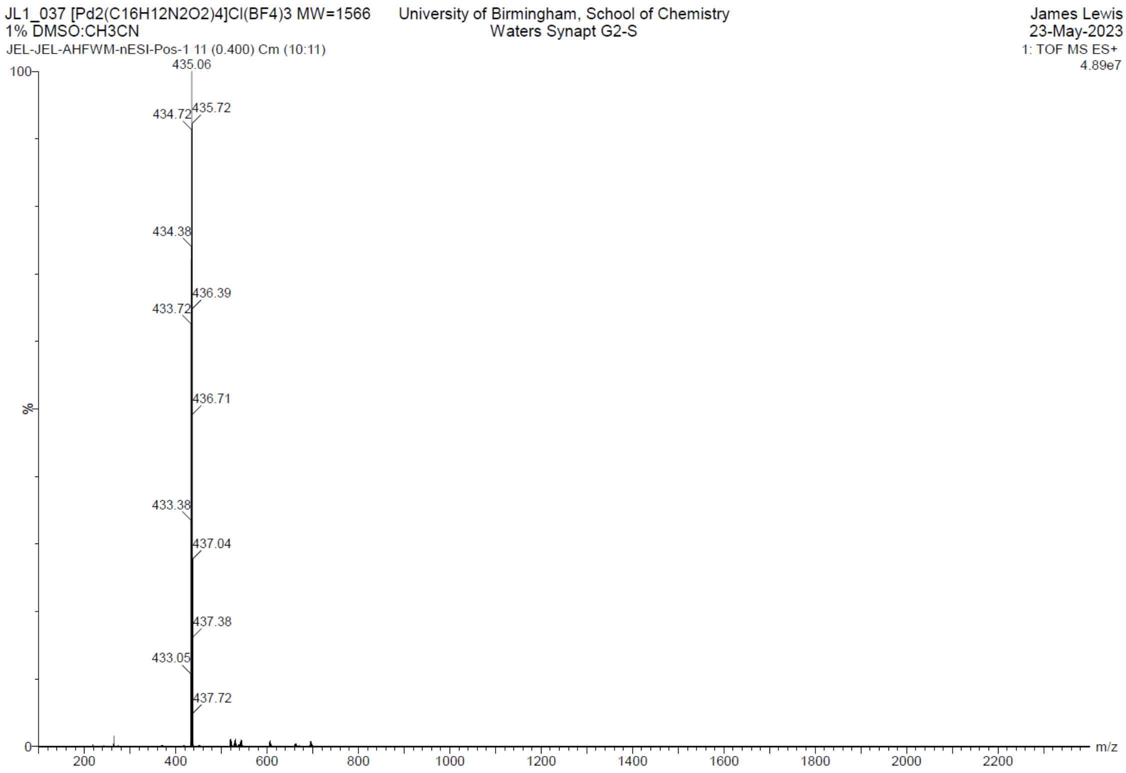


**Figure S33 ESI-MS of C1$^{Q}$⊃Cl.**

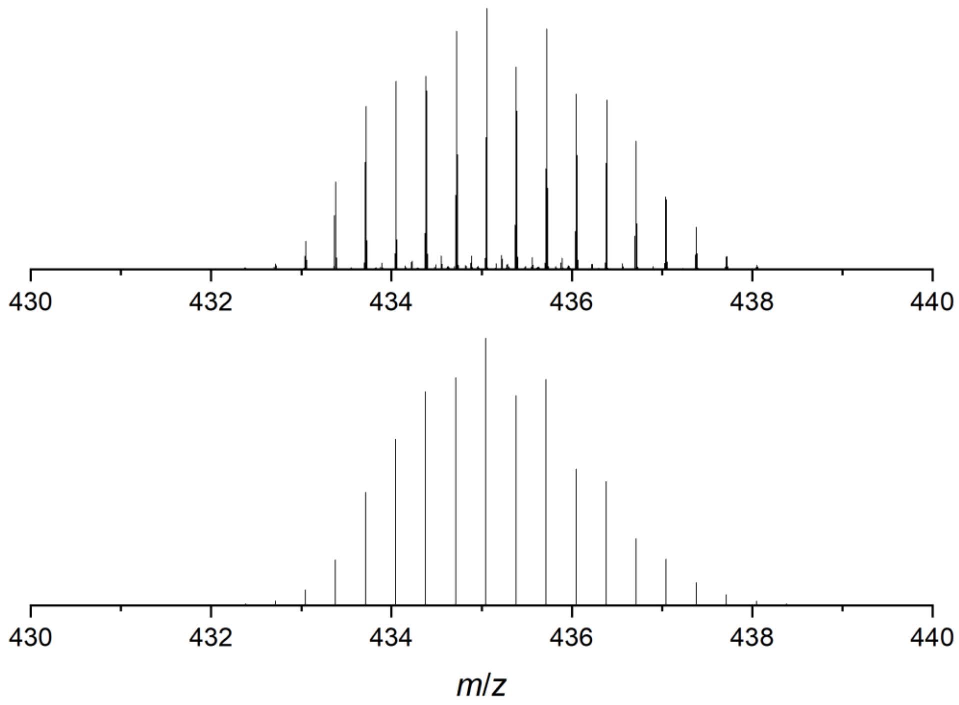


**Figure S34 Observed (top) and calculated (bottom) isotopic pattern for {[$Pd_2$(L1$^{Q}$)$_4$]Cl}$^{3+}$.**

## S2.6 Synthesis of L1$^P$

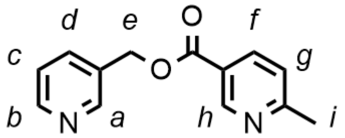


To a stirring solution of 6-methylnicotinic acid (0.301 g, 2.2 mmol, 1.1 eq.) and DMAP (23.9 mg, 0.20 mmol, 0.1 eq.) in $CHCl_3$ (dry, 10 mL) at 0 °C was added EDCl (0.372 g, 1.9 mmol, 0.97 eq.) as a solid. After 30 minutes, pyridin-3-methanol (0.218 g, 2.0 mmol, 1.0 eq.) in $CHCl_3$ (dry, 2 mL) was added via syringe. The reaction mixture was stirred for 18 h, allowing to warm to rt. The organic phase was washed with sat. aq. $NaHCO_3$ (20 mL) and $H_2O$ (20 mL), dried ($MgSO_4$) and the solvent removed in vacuo. After purification by column chromatography on silica gel (step gradient 0% to 60% v/v acetone in $CH_2Cl_2$ in 10% increments) the product was obtained as a yellow crystalline solid (0.391 g, 86%).

**$^1$H NMR** (500 MHz, $d_6$-acetone) δ: 9.04 (d, *J* = 2.3 Hz, 1H, $H_h$), 8.75 (s, 1H, $H_a$), 8.58 (d, *J* = 4.0 Hz 1H, $H_b$), 8.22 (dd, *J* = 8.1, 2.3 Hz, 1H, $H_f$), 7.93 (app. dt, *J* = 7.9, 2.1 Hz, 1H, $H_d$), 7.42 (dd, *J* = 7.8, 4.8 Hz, 1H, $H_c$), 7.39 (d, *J* = 8.2 Hz, 1H, $H_g$), 5.45 (s, 2H, $H_e$), 2.57 (s, 3H, $H_i$).

**$^{13}$C NMR** (126 MHz, $d_6$-acetone) δ: 165.76, 164.41, 150.98 ($C_h$), 150.64 ($C_a$), 150.50 ($C_b$), 137.88 ($C_f$), 136.76 ($C_d$), 132.77, 124.38 ($C_c$), 124.03, 123.80 ($C_g$), 65.06 ($C_e$), 24.71 ($C_i$).

**HR-ESI-MS** *m/z* = 229.0981 $[M+H]^+$ calc. 229.0977.

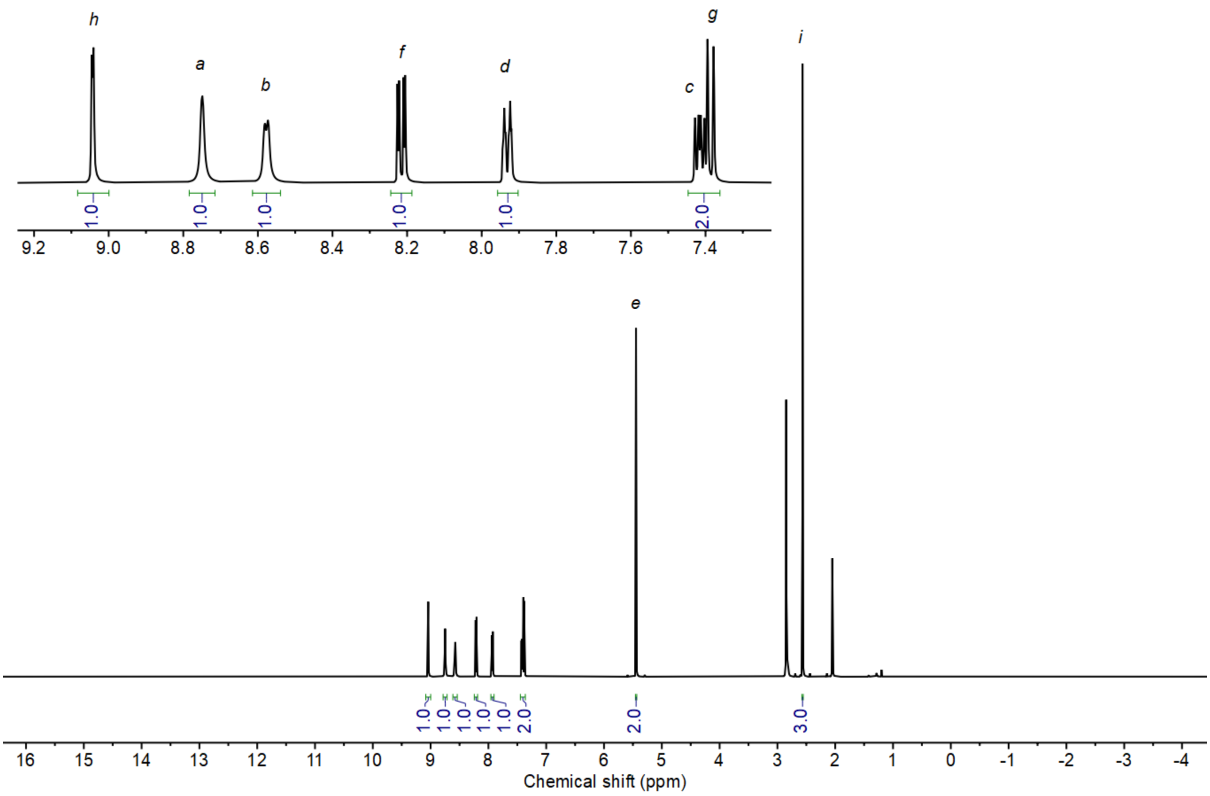


**Figure S35 $^1$H NMR (500 MHz, $d_6$-acetone) of L1$^P$.**

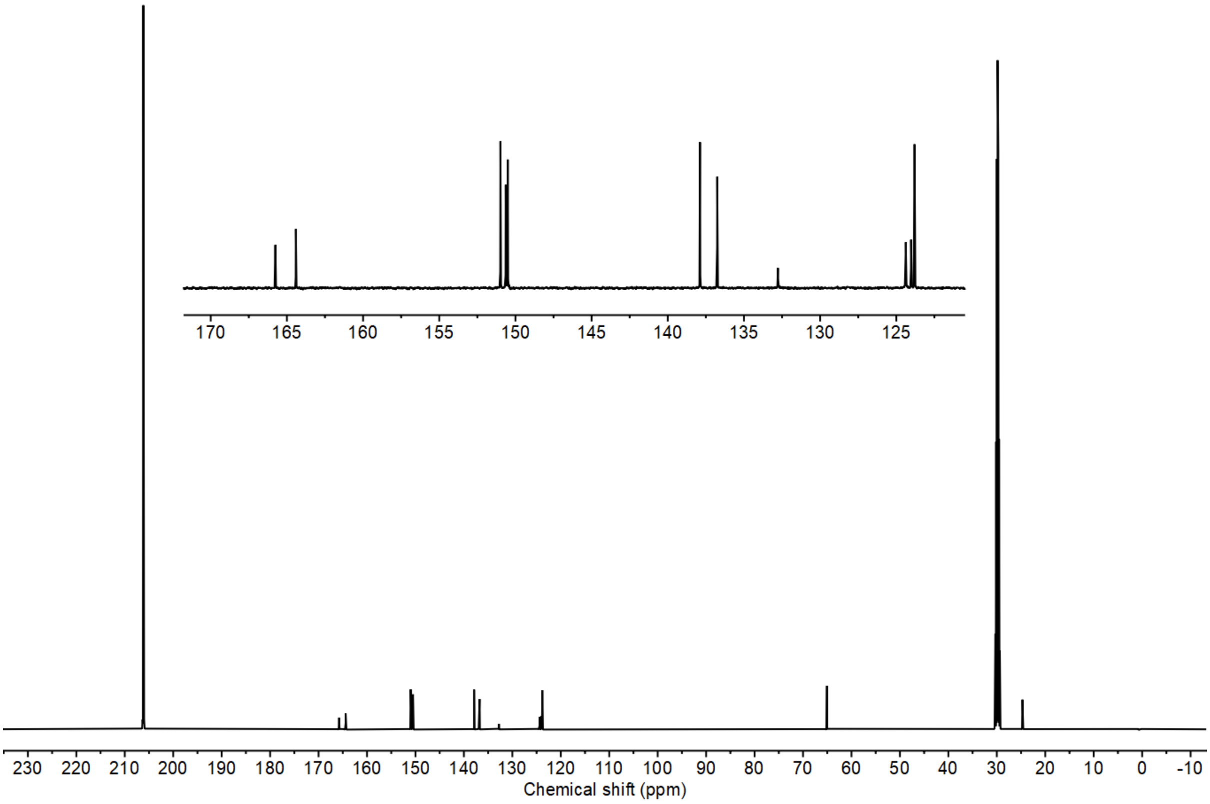


**Figure S36 $^{13}C$ NMR (126 MHz, $d_6$-acetone) of L1$^P$.**

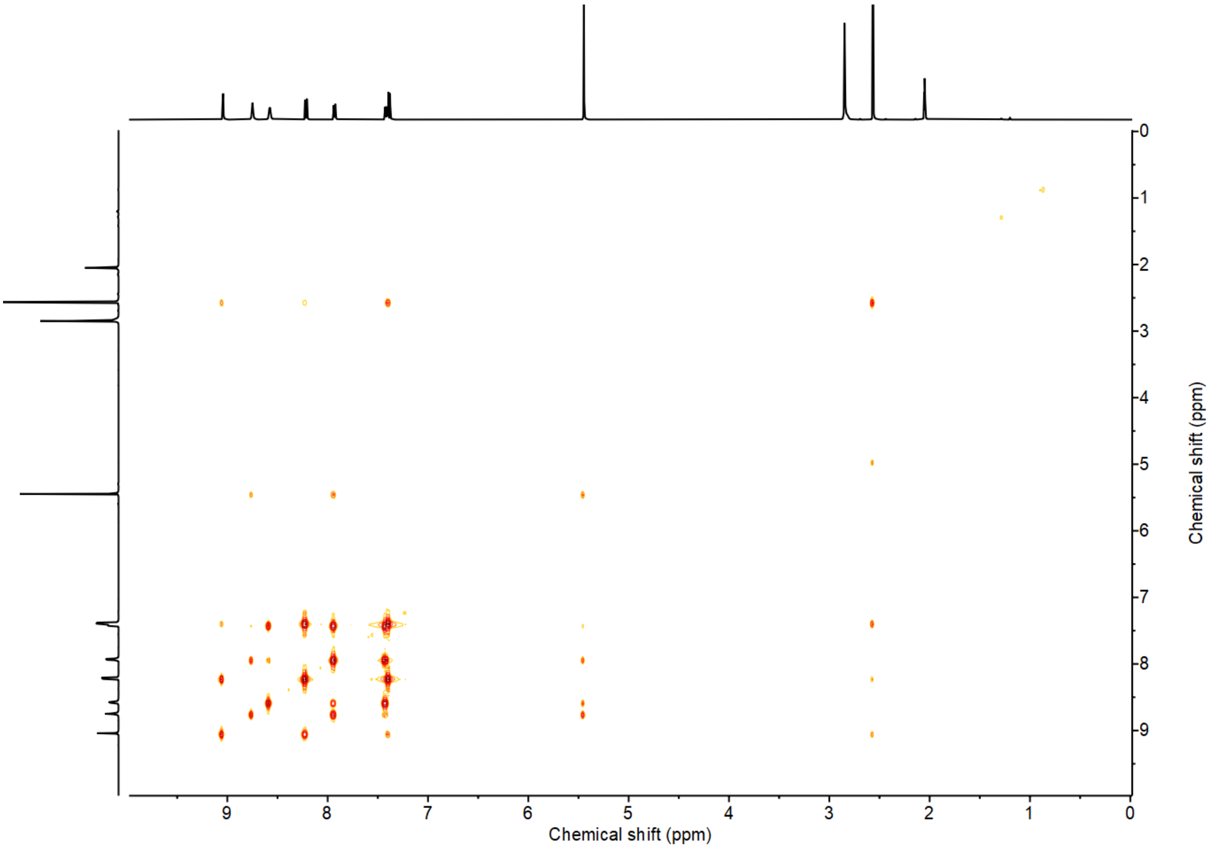


**Figure S37 COSY ($d_6$-acetone) of L1$^P$.**

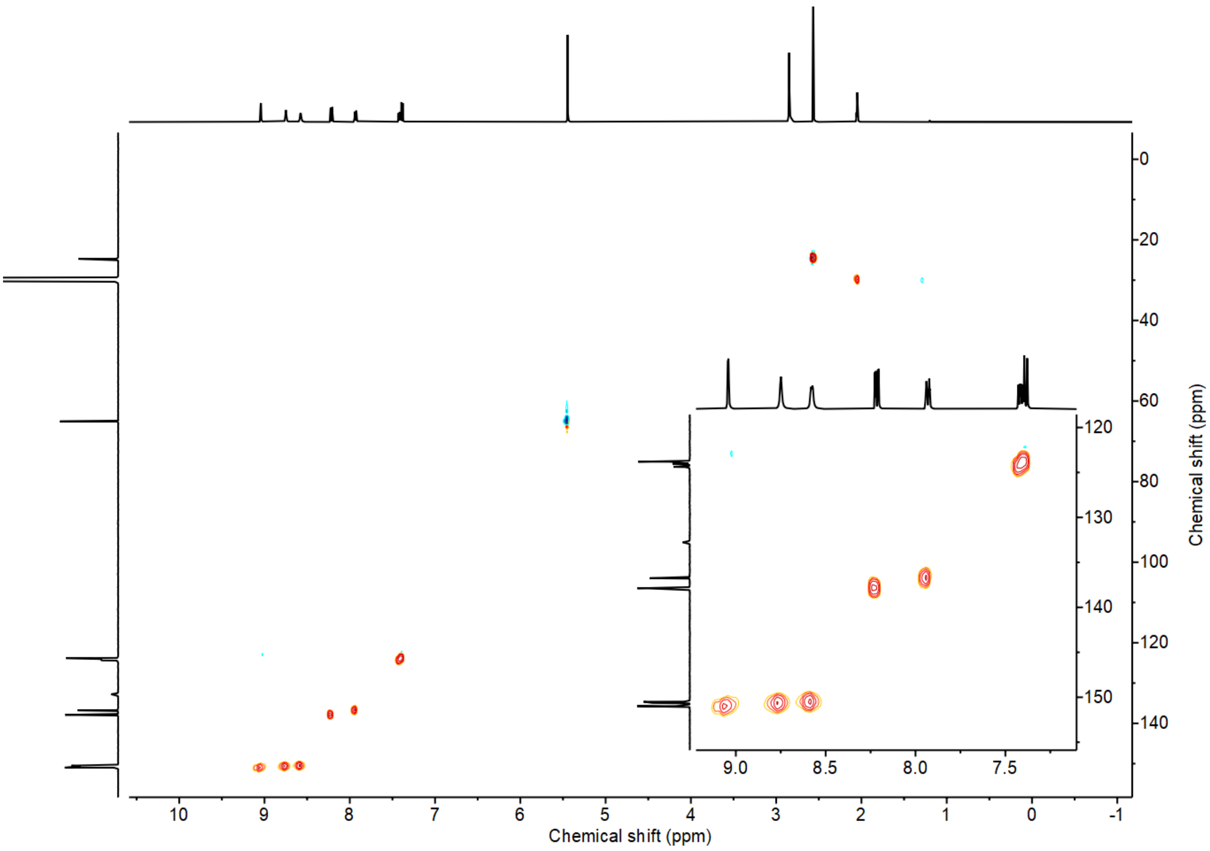


**Figure S38 HSQC ($d_6$-acetone) of L1$^{P}$.**

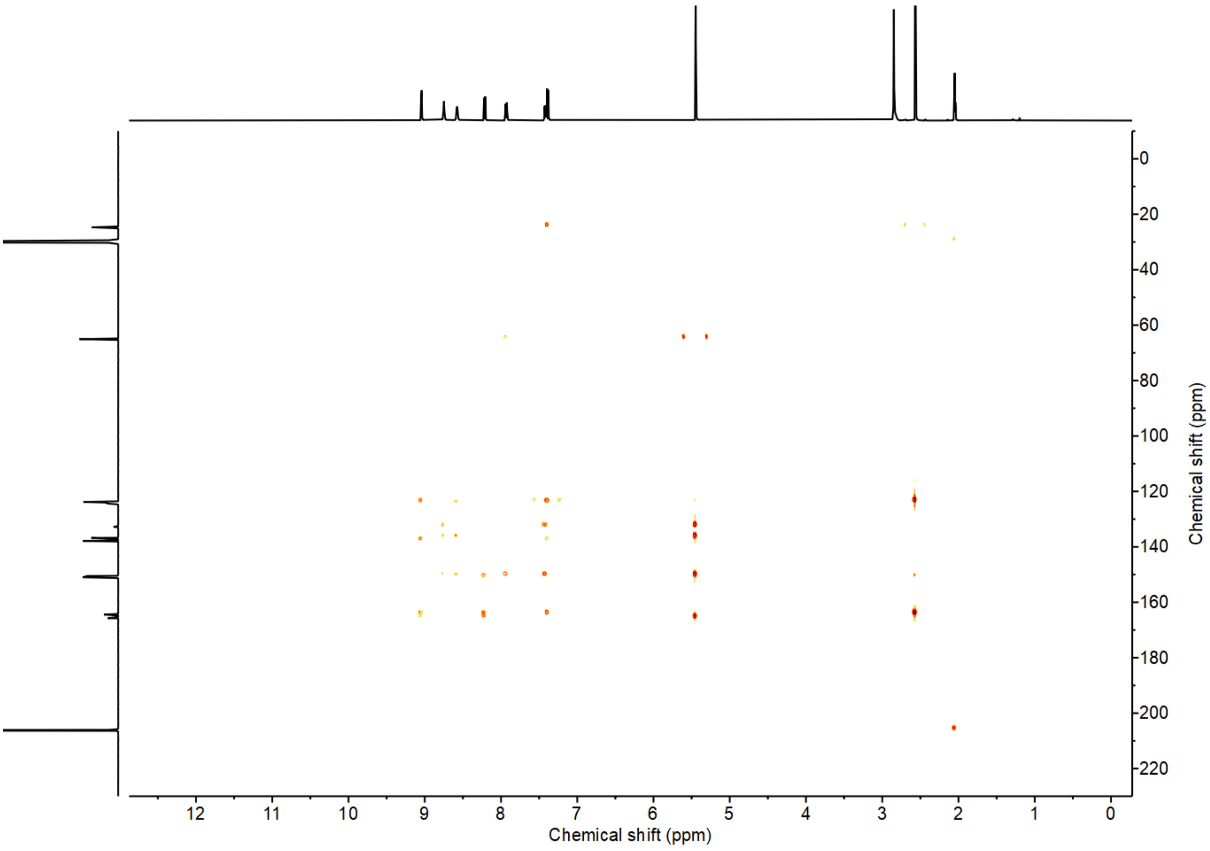


**Figure S39 HMBC ($d_6$-acetone) of L1$^{P}$.**

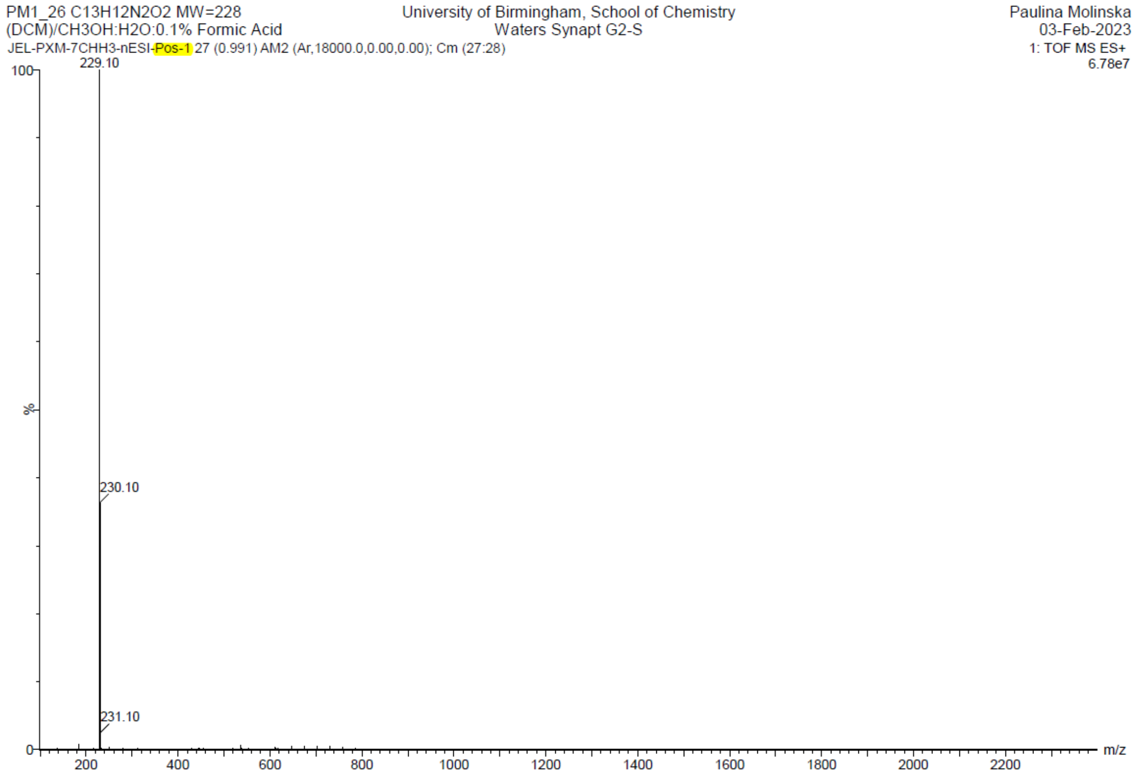


**Figure S40 ESI-MS of L1$^{P}$.**

## S2.7 Synthesis of *trans*-C1$^{P}$

**L1$^{P}$** (6.8 mg, 30 µmol, 1 eq.) and $Pd(NO_3)_2 \cdot 2H_2O$ (4.0 mg, 15 µmol, 0.5 eq.) were sonicated in $d_6$-DMSO (0.75 mL) until a homogenous solution was obtained. After standing at 50 °C for 24 h, no further changes were observed by $^{1}$H NMR, and *trans*-**C1$^{P}$** was formed as the major product.

Only peaks for major species are reported.

**$^{1}$H NMR** (500 MHz, $d_6$-DMSO) δ: 10.33 (d, *J* = 2.0 Hz, 4H, $H_h$), 9.29 (s, 4H, $H_a$), 8.60 (dd, *J* = 8.2, 1.9 Hz, 4H, $H_f$), 8.48 (d, *J* = 5.7 Hz, 4H, $H_b$), 8.13 (dd, *J* = 8.1, 1.6 Hz, 4H, $H_d$), 7.91 (d, *J* = 8.3 Hz, 5H, $H_g$), 7.56 (dd, *J* = 7.9, 5.8 Hz, 4H, $H_c$), 5.35 (s, 8H, $H_e$), 3.54 (s, 12H, $H_i$).

**$^{13}$C NMR** (126 MHz, $d_6$-DMSO) δ: 165.1, 162.6, 153.5 ($C_h$), 151.5 ($C_b$), 150.8 ($C_a$), 142.0 ($C_f$), 140.5 ($C_d$), 134.8, 128.8 ($C_g$), 126.6 ($C_c$), 126.3, 65.0 ($C_e$), 26.5 ($C_i$).

**Diffusion coefficient** (400 MHz, $d_6$-DMSO) *D*: $1.10 \times 10^{-10}$ $m^2s^{-1}$.

**HR-ESI-MS** *m/z* = 396.05 $\{[Pd_2(\mathbf{L1^P})_4](NO_3)\}^{3+}$ calc. 396.05; 625.07 $\{[Pd_2(\mathbf{L1^P})_4](NO_3)_2\}^{2+}$ calc. 625.07; 1312.13 $\{[Pd_2(\mathbf{L1^P})_4](NO_3)_3\}^{+}$ calc. 1312.13.

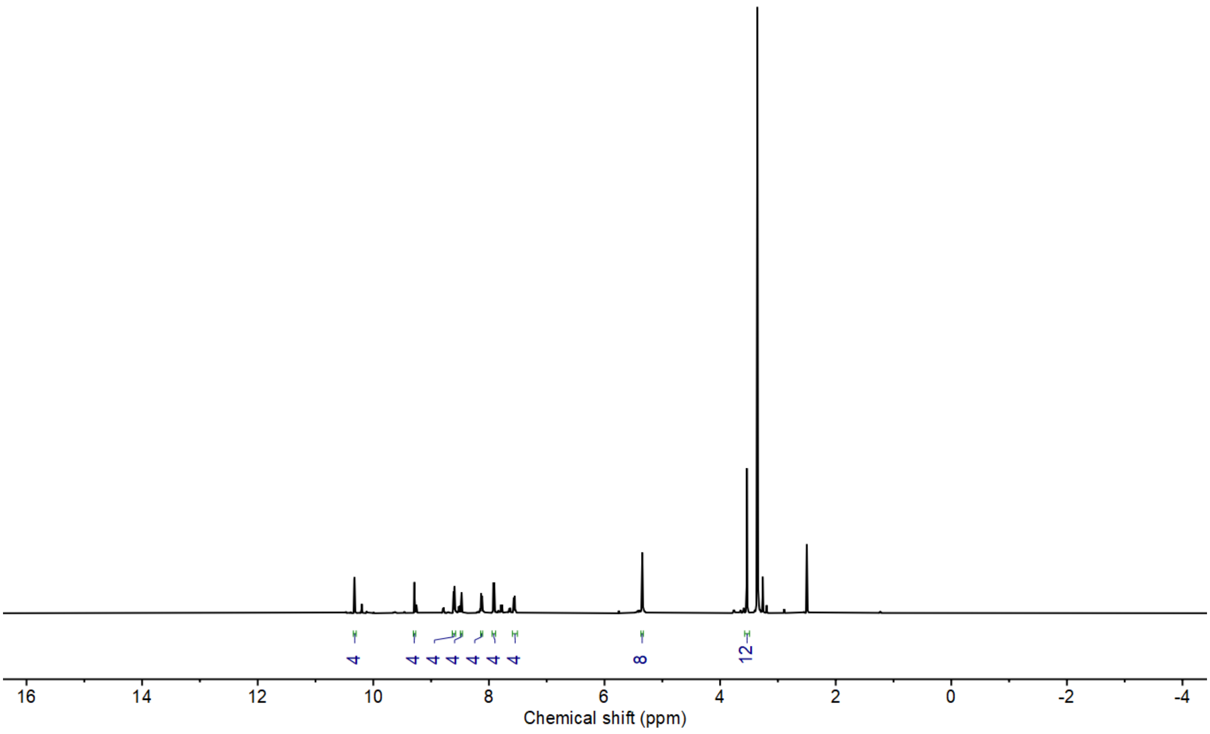


**Figure S41** **$^{1}$H NMR (500 MHz, $d_6$-DMSO) of C1$^{P}$.**

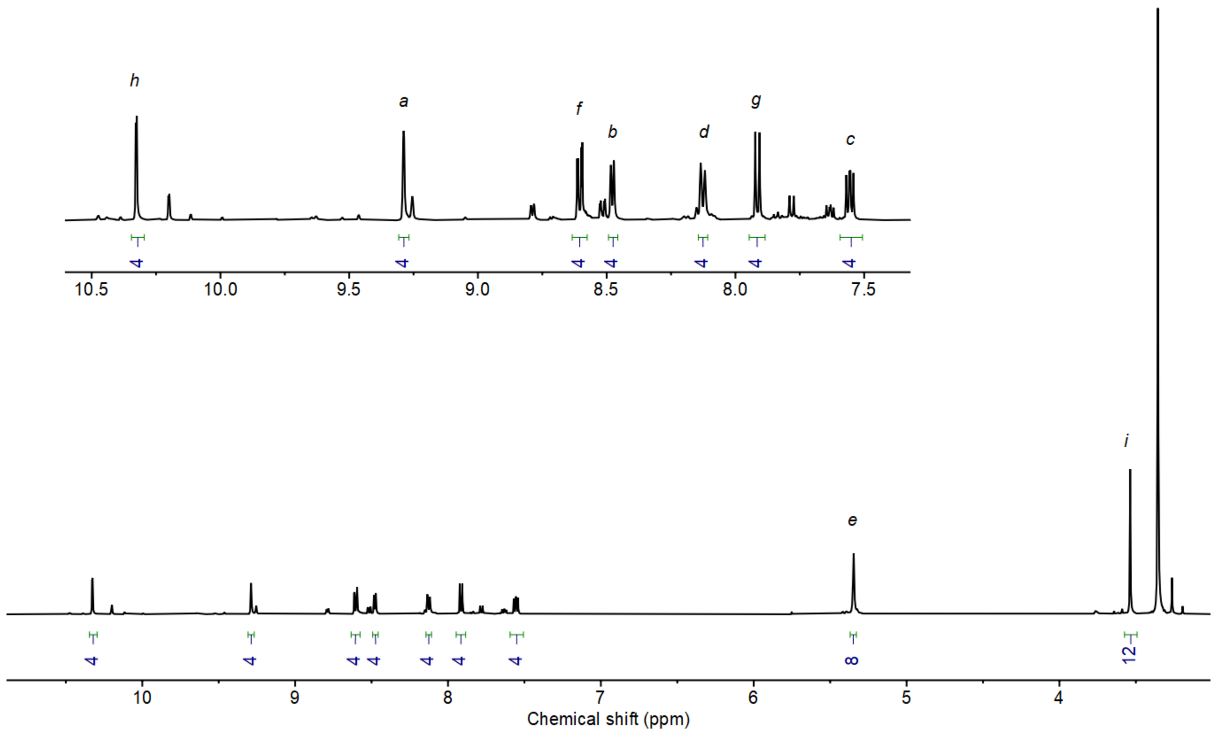


**Figure S42 Partial $^{1}H$ NMR (500 MHz, $d_6$-DMSO) of C1$^{P}$.**

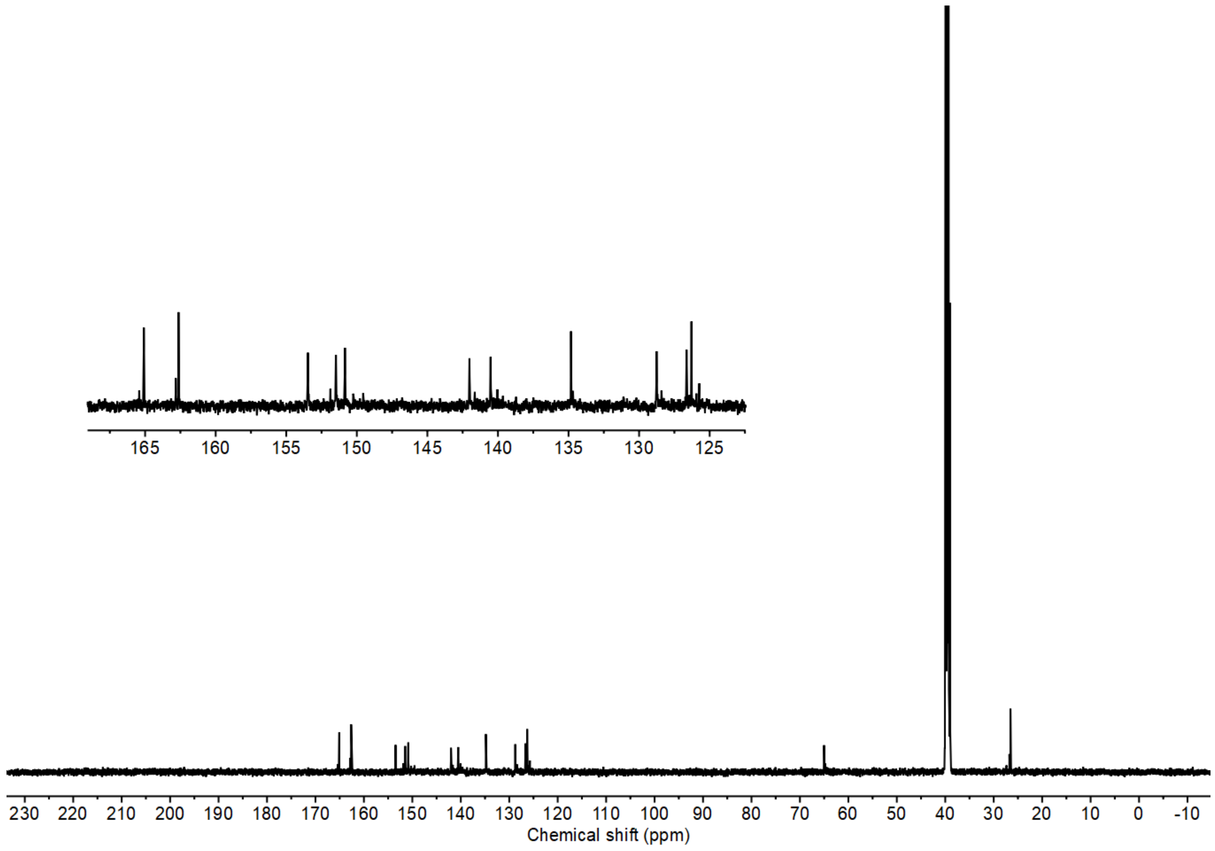


**Figure S43 $^{13}C$ NMR (126 MHz, $d_6$-DMSO) of C1$^{P}$.**

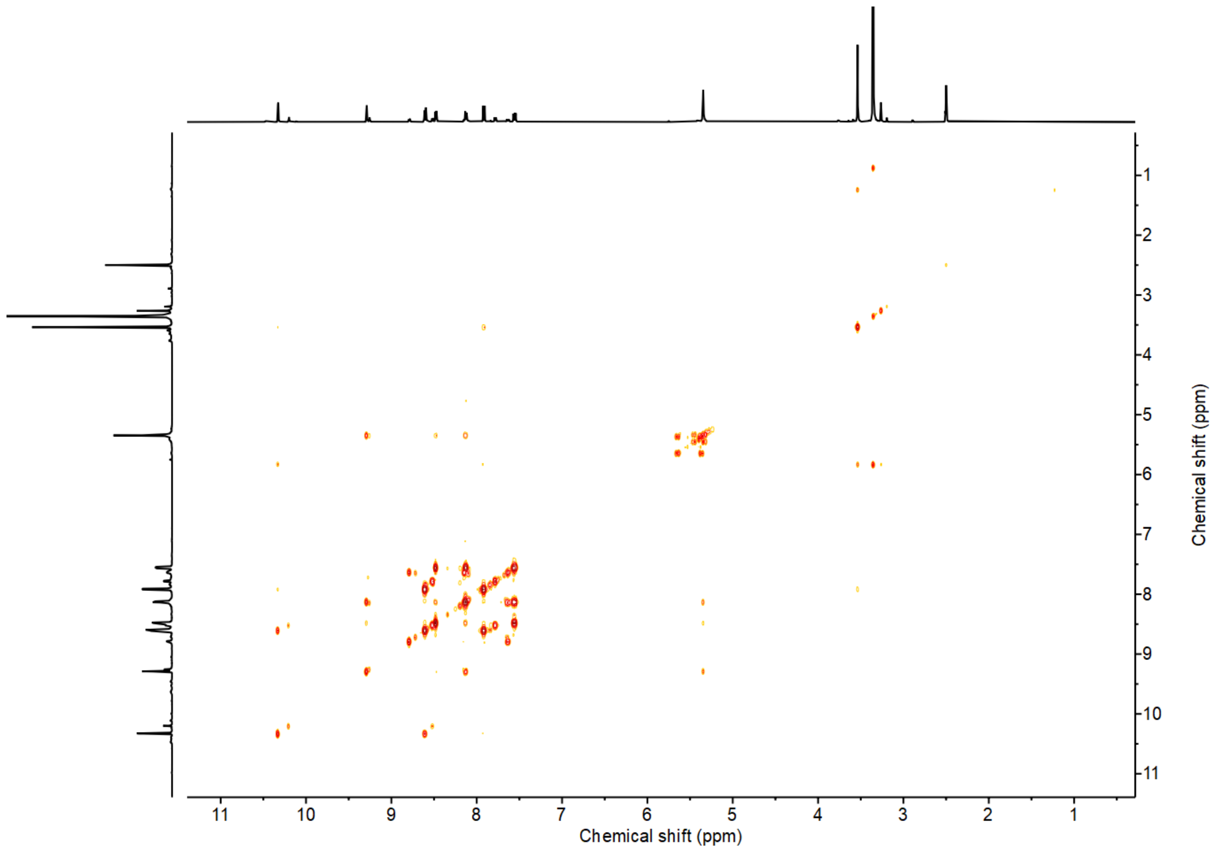


**Figure S44 COSY ($d_6$-DMSO) of C1$^P$.**

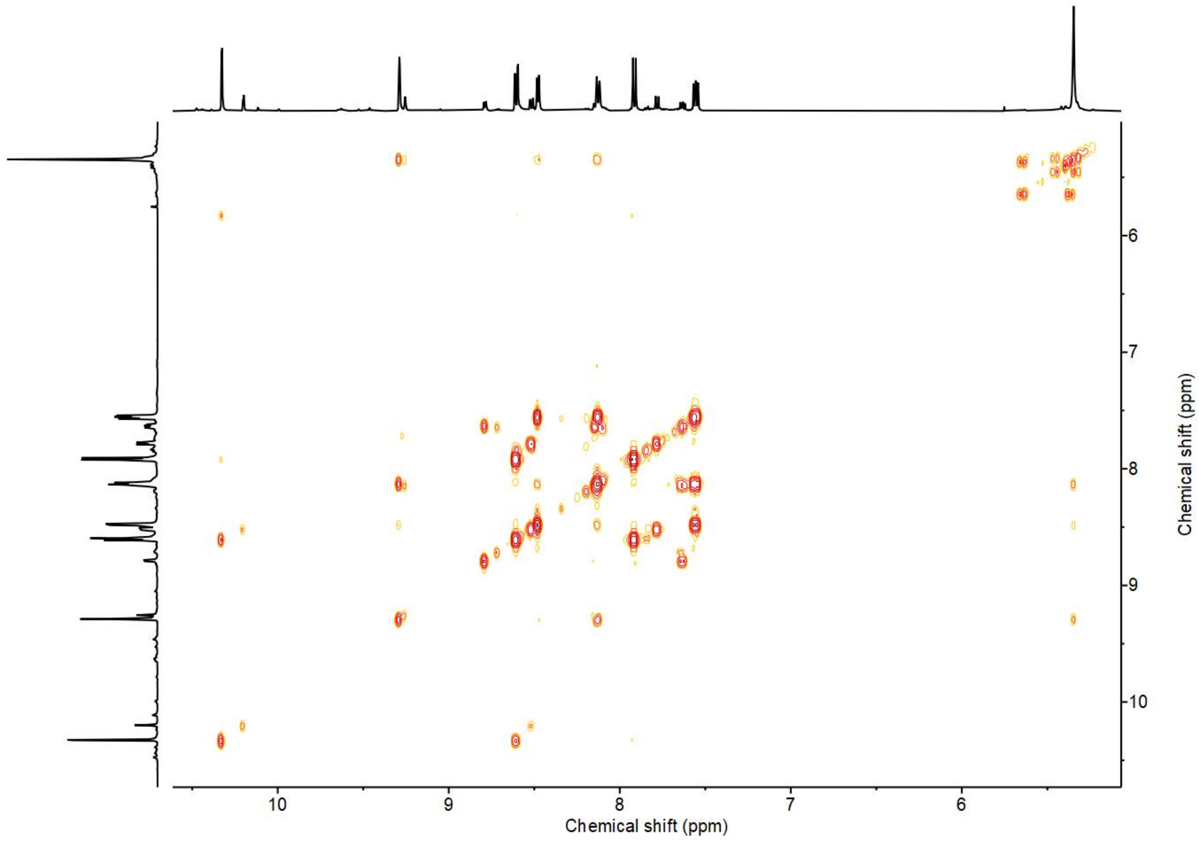


**Figure S45 Partial COSY ($d_6$-DMSO) of C1$^P$.**

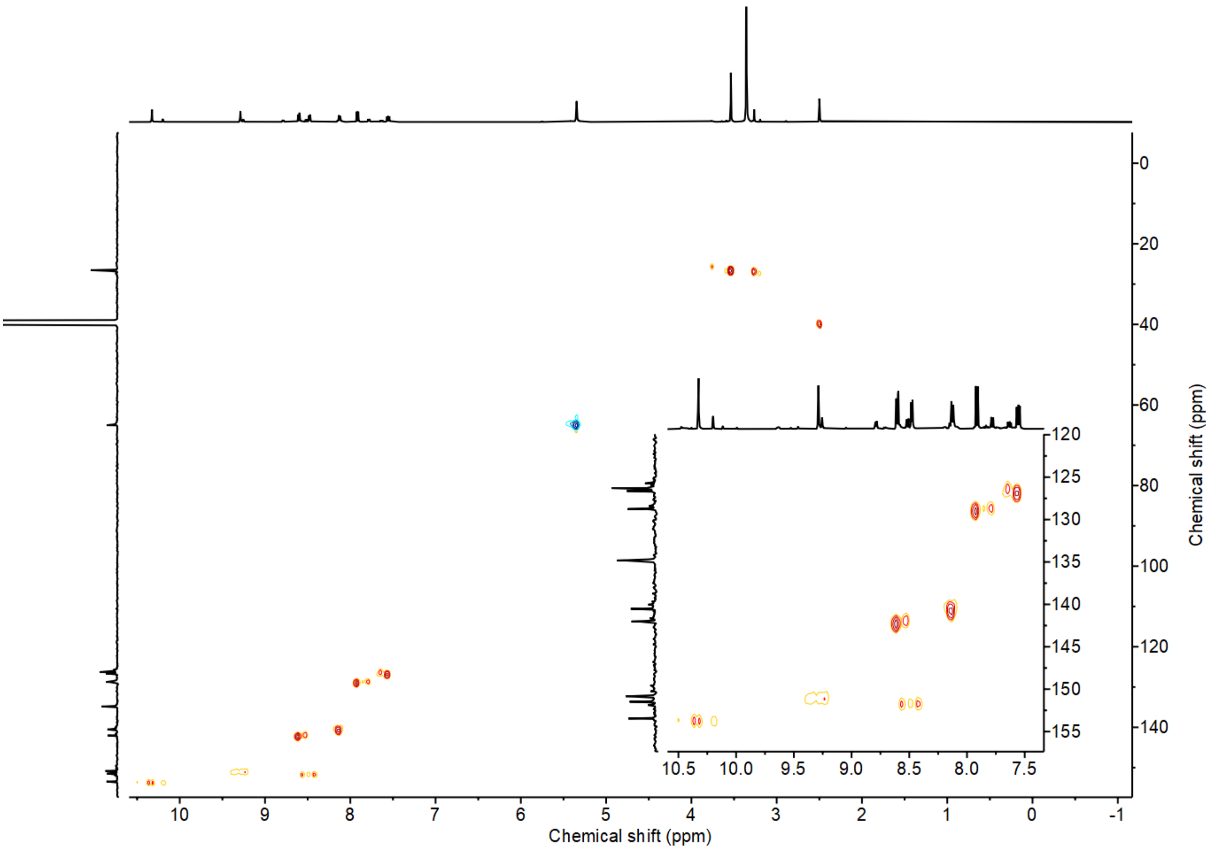


**Figure S46 HSQC ($d_6$-DMSO) of C1^P.**

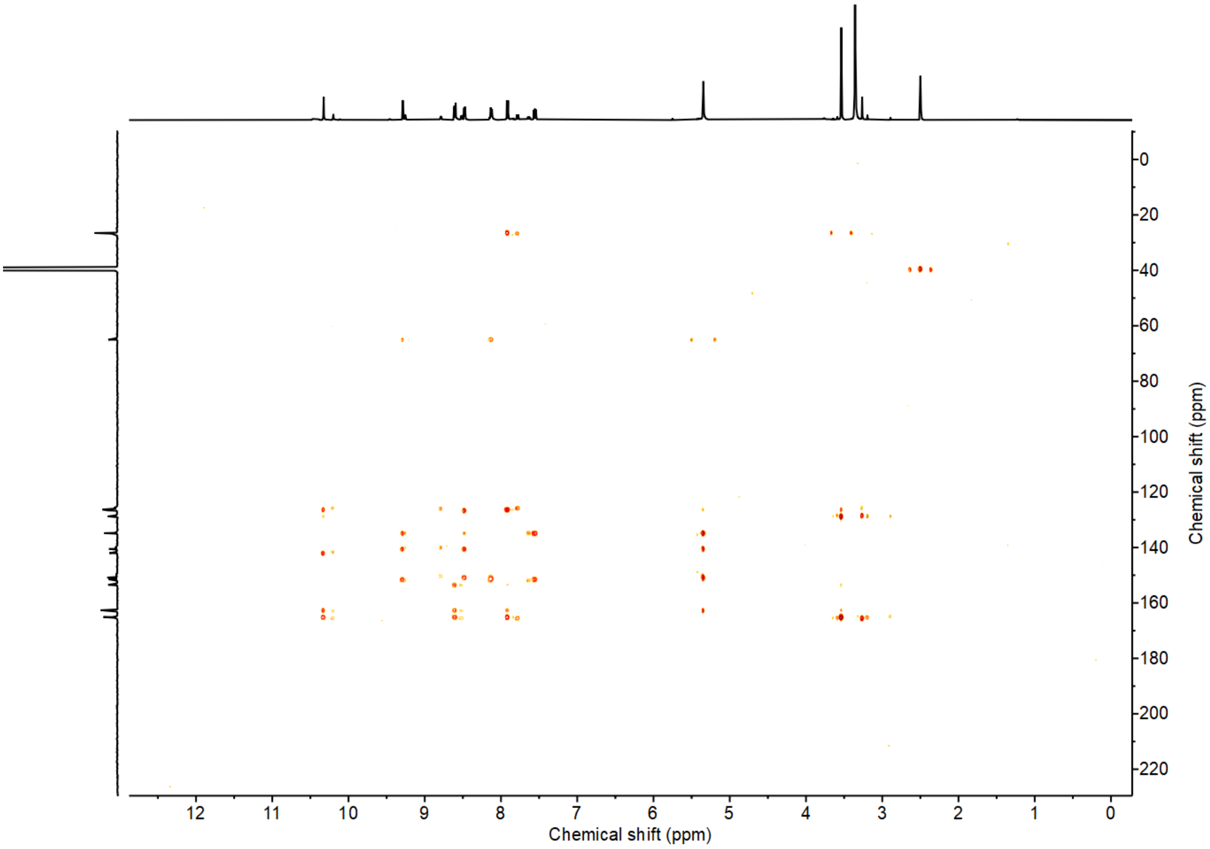


**Figure S47 HMBC ($d_6$-DMSO) of C1^P.**

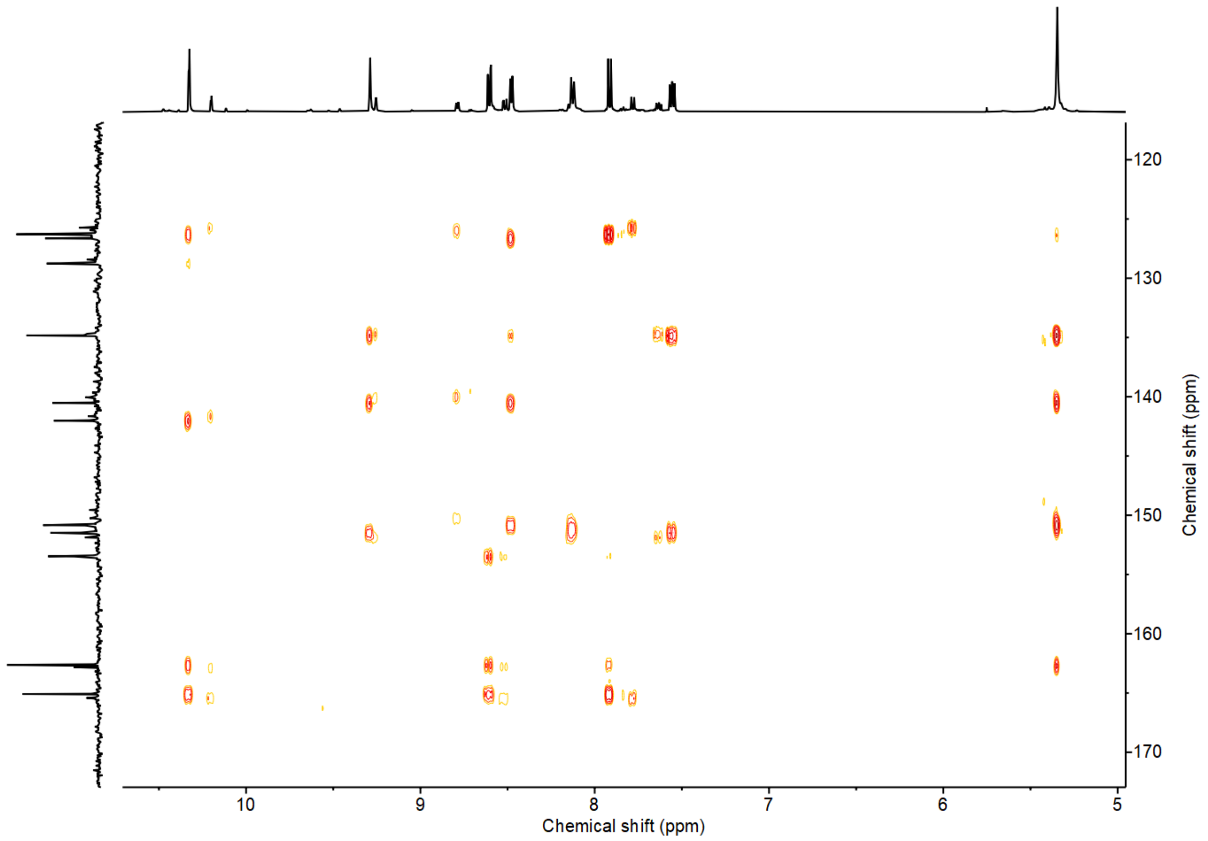


**Figure S48 Partial HMBC ($d_6$-DMSO) of C1^P.**

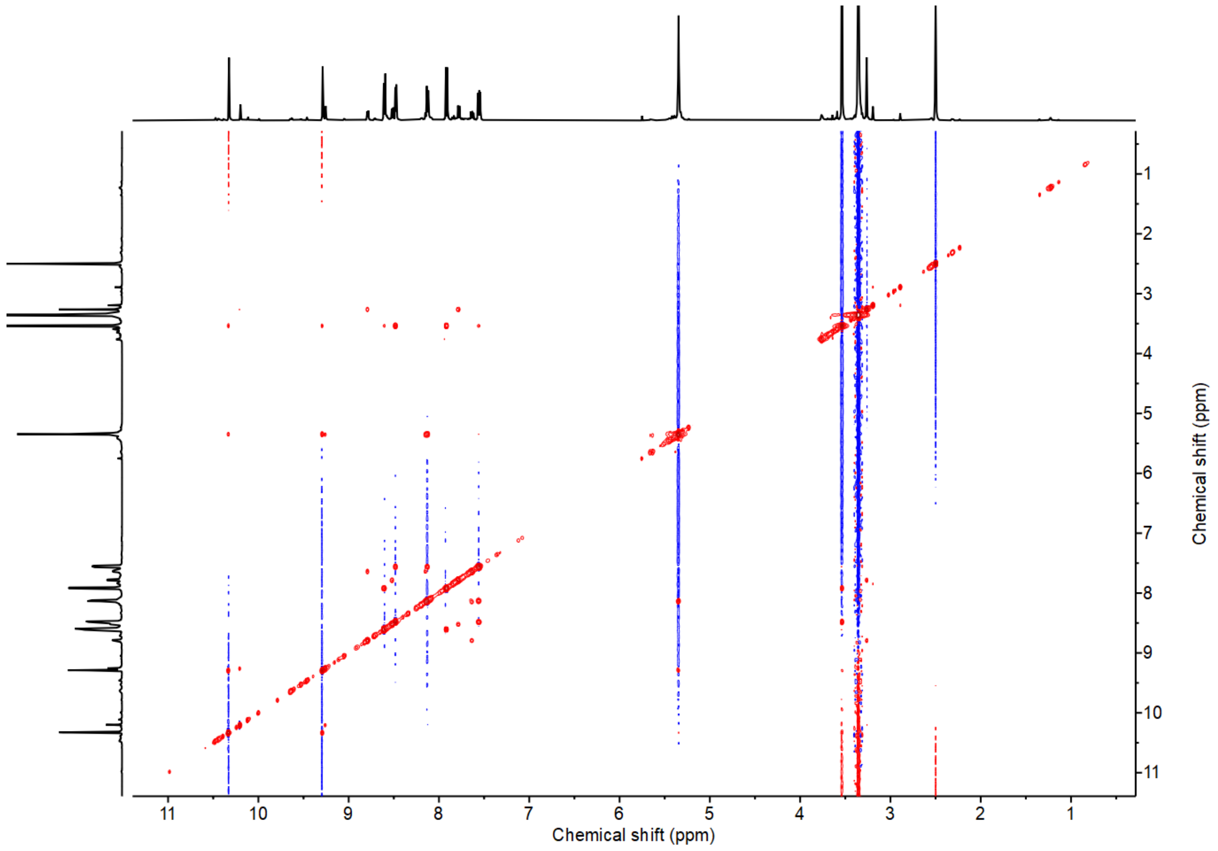


**Figure S49 NOESY ($d_6$-DMSO) of C1^P.**

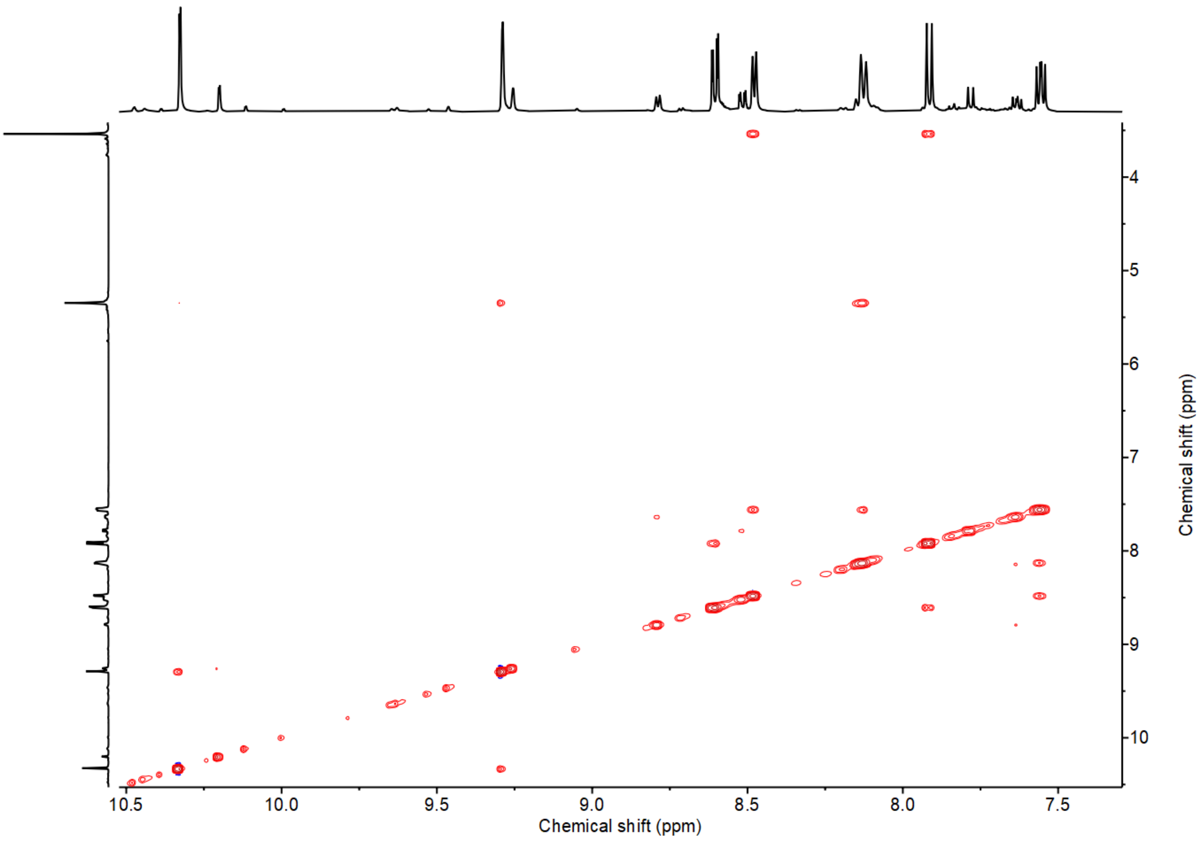


**Figure S50 Partial NOESY ($d_6$-DMSO) of C1$^{P}$.**

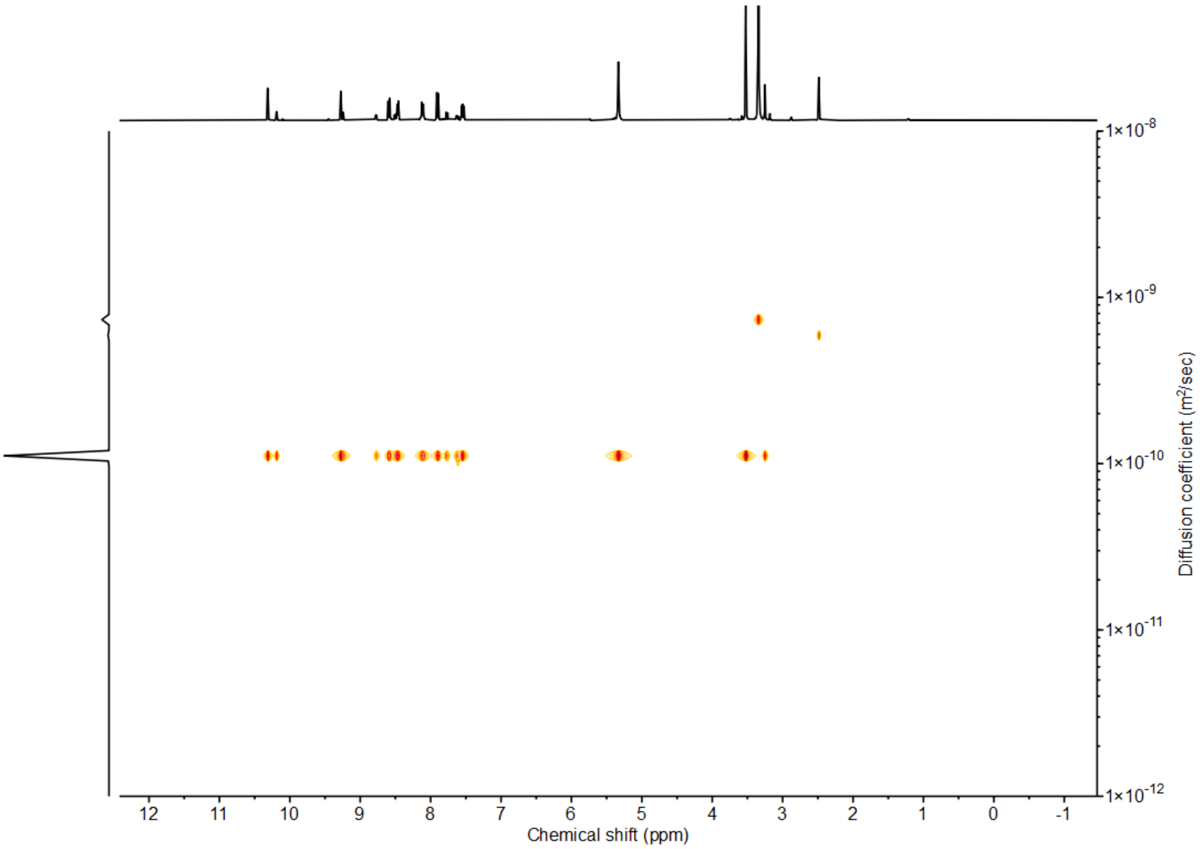


**Figure S51 DOSY (400 MHz, $d_6$-DMSO) of C1$^{P}$.**

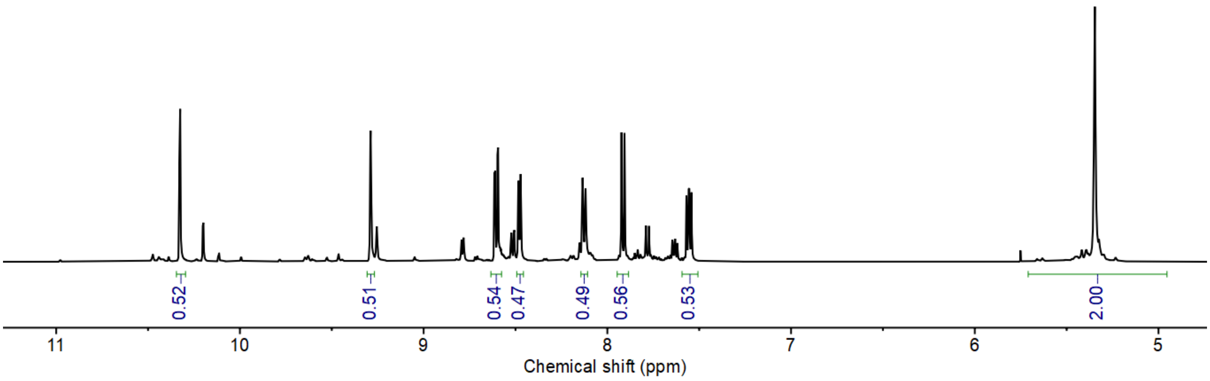


**Figure S52 Partial $^{1}H$ NMR (500 MHz, $d_6$-DMSO) of C1$^{P}$ with integrals relative to sum total of $CH_2$ signals.**

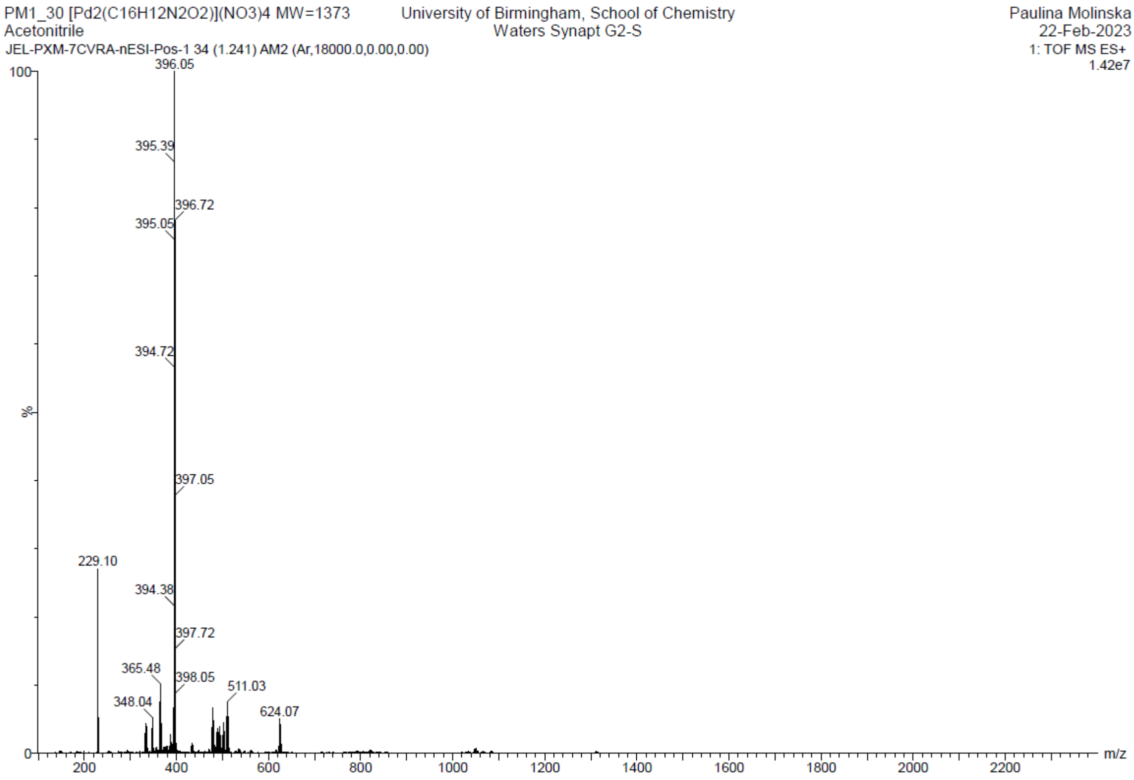


**Figure S53 ESI-MS of C1$^{P}$.**

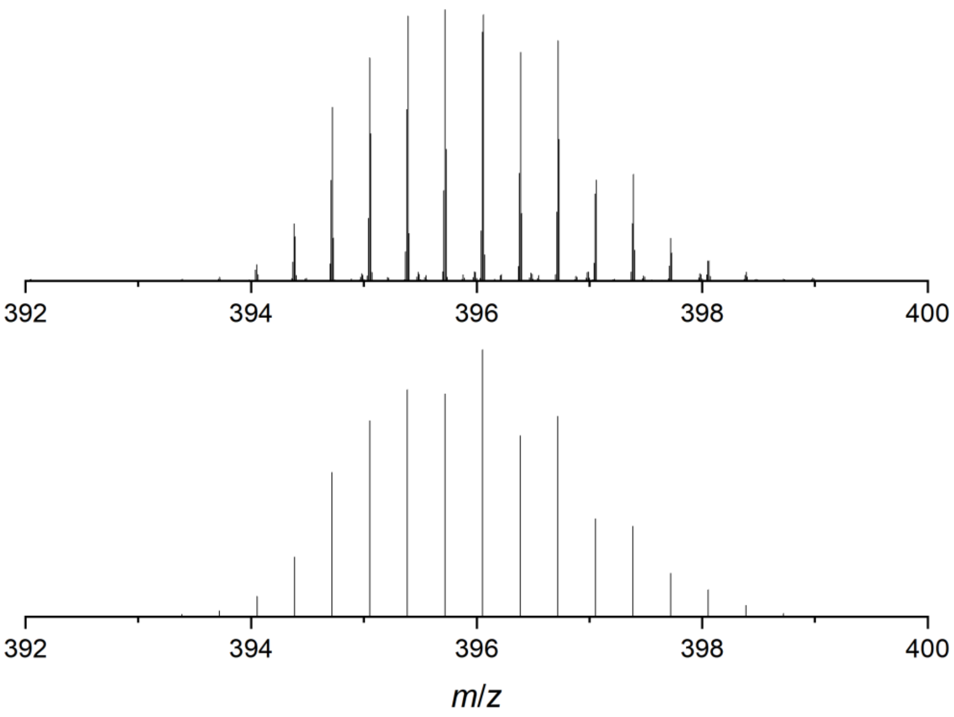


**Figure S54 Observed (top) and calculated (bottom) isotopic pattern for {[$Pd_2$(L1$^P$)$_4$]($NO_3$)}$^{3+}$.**

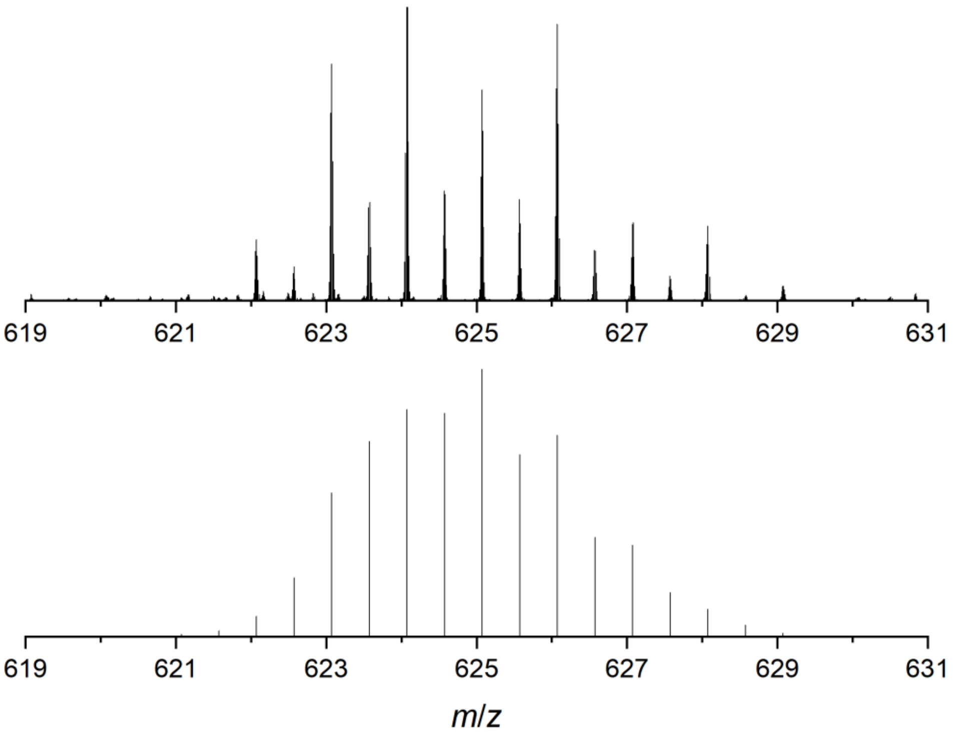


**Figure S55 Observed (top) and calculated (bottom) isotopic pattern for {[$Pd_2$(L1$^P$)$_4$]($NO_3$)$_2$}$^{2+}$.**

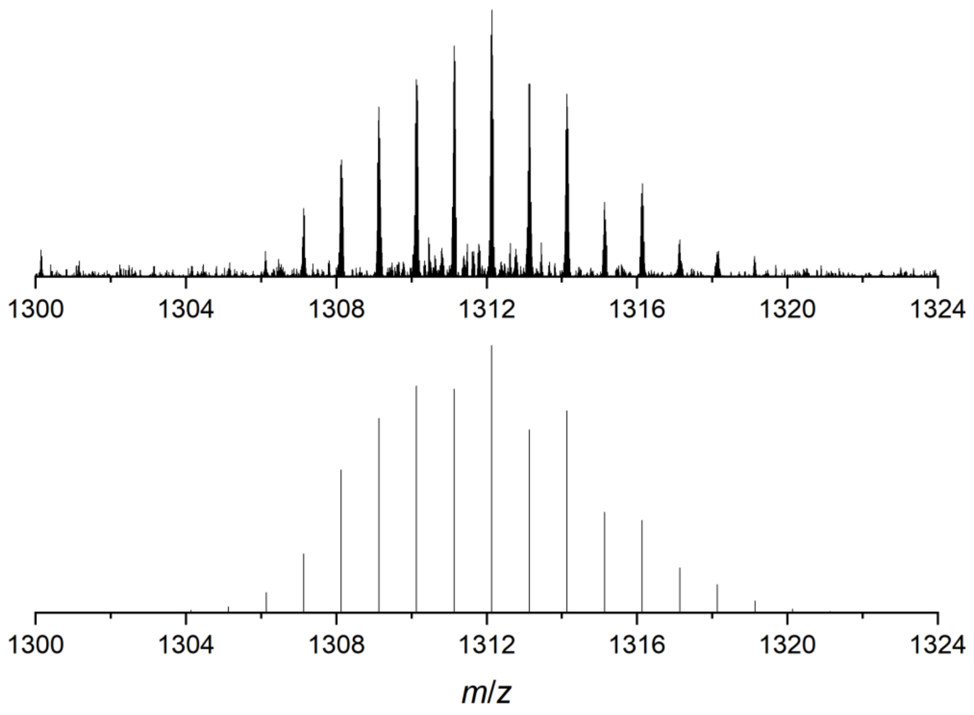


**Figure S56 Observed (top) and calculated (bottom) isotopic pattern for {[$Pd_2(L1^P)_4$]($NO3)_3$}$^+$.**

## S2.8 Identification of minor *cis*-C1$^{P}$ isomer

**$^{1}$H NMR** (500 MHz, $d_6$-DMSO) δ: 10.20 (d, $J$ = 1.9 Hz, 4H, $H_h$), 9.25 (s, 4H, $H_a$), 8.79 (d, $J$ = 5.5 Hz, 4H, $H_b$), 8.52 (dd, $J$ = 8.2, 1.9 Hz, 4H, $H_f$), 8.15 (from COSY; 4H, $H_d$), 7.78 (d, $J$ = 8.3 Hz, 4H, $H_g$), 7.63 (dd, $J$ = 7.9, 5.8 Hz, 4H, $H_c$), 5.36 (from COSY; 8H, $H_e$).

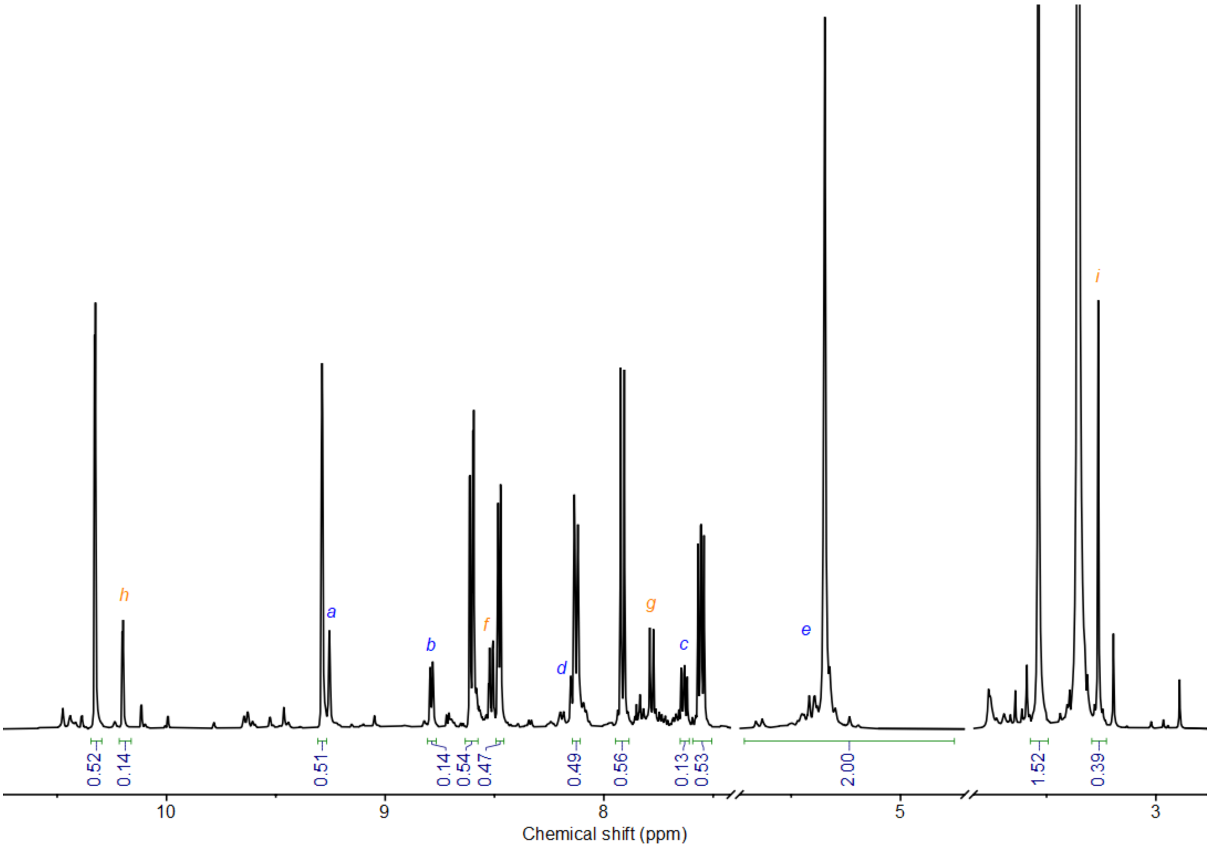


**Figure S57 Partial $^{1}$H NMR (500 MHz, $d_6$-DMSO) of C1$^{P}$ with peaks assigned to minor *cis* isomer labelled.**

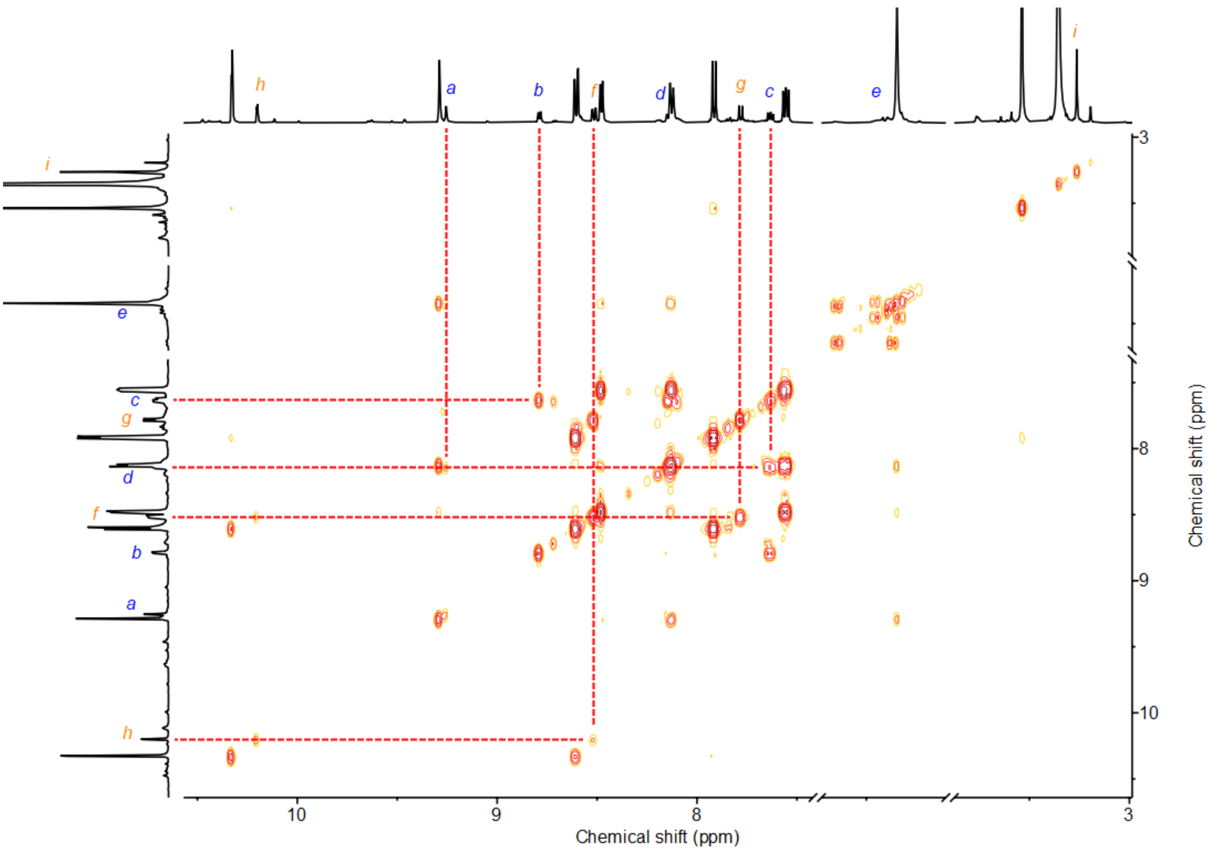


**Figure S58 Partial COSY (500 MHz, $d_6$-DMSO) of C1$^P$ with peaks assigned to minor *cis* isomer labelled.**

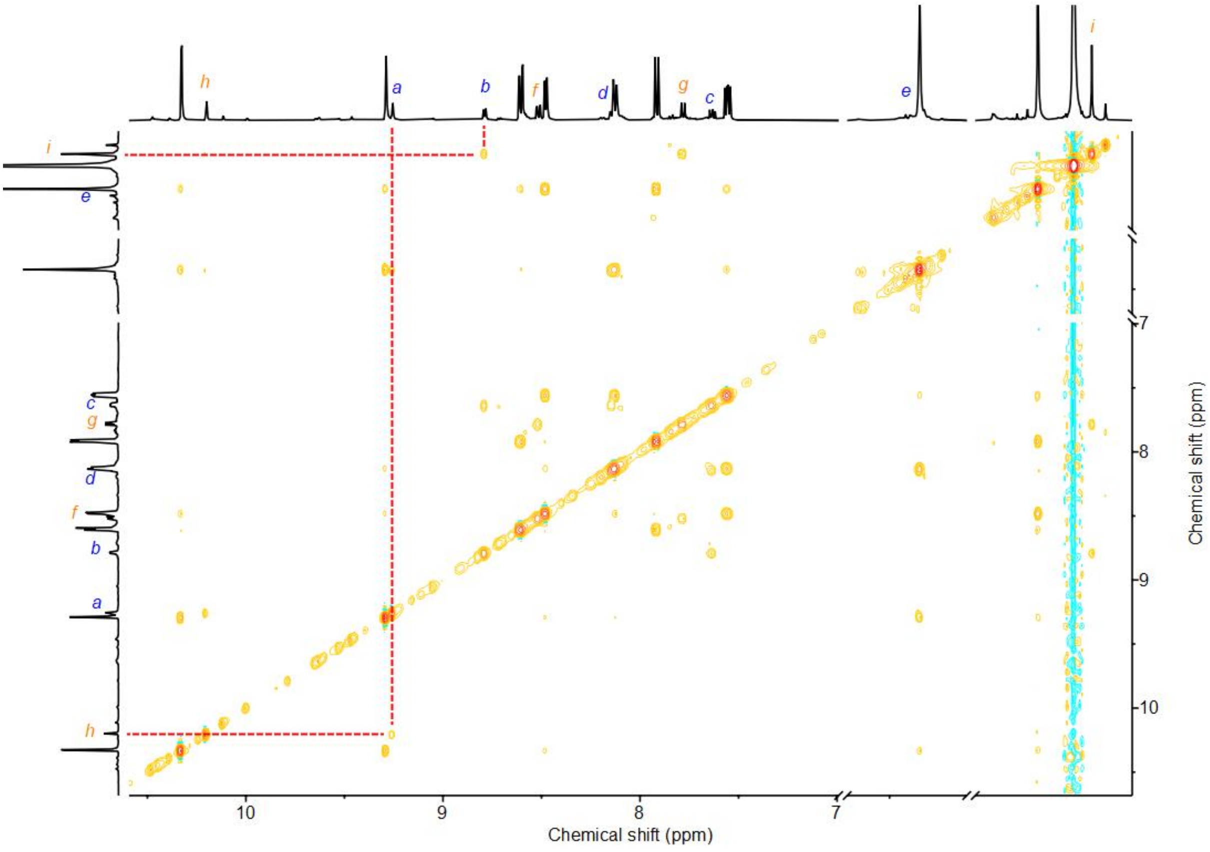


**Figure S59 Partial NOESY (500 MHz, $d_6$-DMSO) of C1$^P$ with peaks assigned to minor *cis* isomer labelled.**

## S2.9 *trans*-C1$^{P}$ + $Bu_4NCl$

**L1$^{P}$** (6.8 mg, 30 μmol, 4 eq.) and $Pd(NO_3)_2 \cdot 2H_2O$ (4.0 mg, 15 μmol, 2 eq.) were sonicated in a 40 mM solution of 1,3,5-trimethoxybenzene in $d_6$-DMSO (0.75 mL) until a homogenous solution was obtained After standing at 50 °C for 48 h, a 100 mM solution of $Bu_4NCl$ in $d_6$-DMSO (75 μL, 7.5 μmol, 1 eq.) was added.

Only peaks for major species are reported.

**$^{1}$H NMR** (400 MHz, $d_6$-DMSO) δ: 10.98 (d, $J$ = 2.0 Hz, 4H, $H_h$), 9.79 (s, 4H, $H_a$), 8.59-8.56 (m, 8H, $H_b$, $H_f$), 8.07 (app. dt, $J$ = 8.2, 1.4 Hz, 4H, $H_d$), 7.91 (d, $J$ = 8.1 Hz, 4H, $H_g$), 7.58 (dd, $J$ = 8.0, 5.7 Hz, 4H, $H_c$), 5.31 (s, 12H).

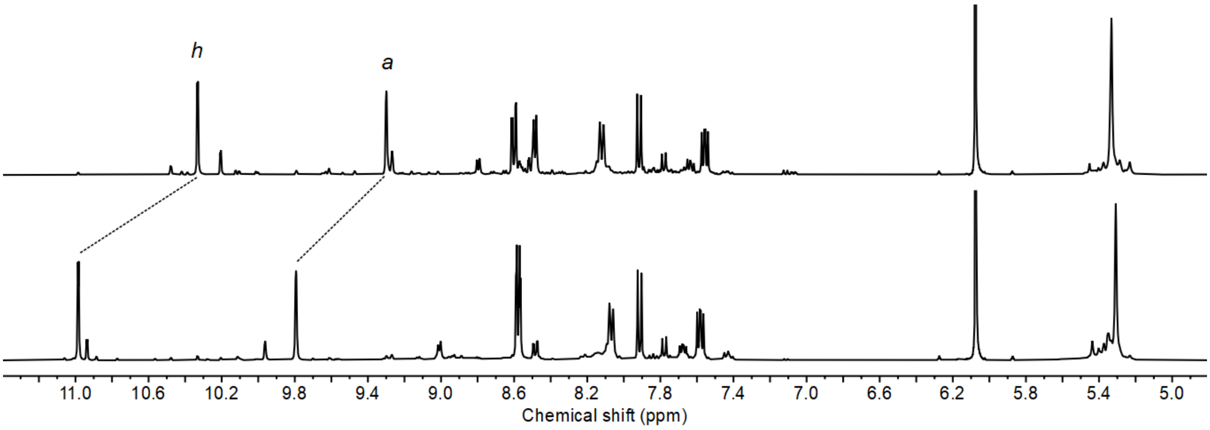


**Figure S60 $^{1}$H NMR (400 MHz, $d_6$-DMSO) of C1$^{P}$ (top) and C1$^{P}$ + 1 eq. $Bu_4NCl$ (bottom) with 1,3,5-trimethoxybenzene as internal standard. Δδ: $H_h$ = 0.65, $H_a$ = 0.49.**

## S2.10 Synthesis of *trans*-C1$^{P}$⊃Cl

**L1$^{P}$** (9.1 mg, 40 μmol, 4 eq.), $[Pd(CH_3CN)_4](BF_4)_2$ (8.9 mg, 20 μmol, 2 eq.) and $Bu_4NCl$ (2.8 mg, 10 μmol, 1 eq.) were sonicated in $d_6$-DMSO (0.75 mL) until a homogenous solution was obtained. After standing at 60 °C for 18 h, the solution was diluted with DMF (1 mL) and filtered through celite. Vapour diffusion of $Et_2O$ into this solution yielded a precipitate. After the mother liquor was decanted, the solid was washed with $Et_2O$ (× 3) and dried *in vacuo* to give the product as a beige solid (7.5 mg, 53%).

Only peaks for major species are reported.

**$^{1}$H NMR** (500 MHz, $d_6$-DMSO) δ: 10.98 (d, $J$ = 2.0 Hz, 4H, $H_h$), 9.78 (s, 4H, $H_a$), 8.59 (dd, $J$ = 8.2, 1.9 Hz, 4H, $H_f$), 8.56 (d, $J$ = 5.7 Hz, 4H, $H_b$), 8.07 (d, $J$ = 8.0 Hz, 4H, $H_d$), 7.91 (d, $J$ = 8.3 Hz, 4H, $H_g$), 7.58 (dd, $J$ = 7.9, 5.8 Hz, 4H, $H_c$), 5.32 (s, 8H, $H_e$), 3.59 (s, 12H, $H_i$).

**$^{13}$C NMR** (126 MHz, $d_6$-DMSO) δ: 165.1, 162.4, 153.1 ($C_h$), 151.4 ($C_b$), 149.9 ($C_a$), 141.7 ($C_f$), 139.5 ($C_d$), 134.7, 128.6 ($C_g$), 126.6 ($C_c$), 126.0, 64.3 ($C_e$), 26.5 ($C_i$).

**HR-ESI-MS** $m/z$ = 387.05 {[Pd$_2$(**L1$^{P}$**)$_4$](Cl)}$^{3+}$ calc. 387.05.

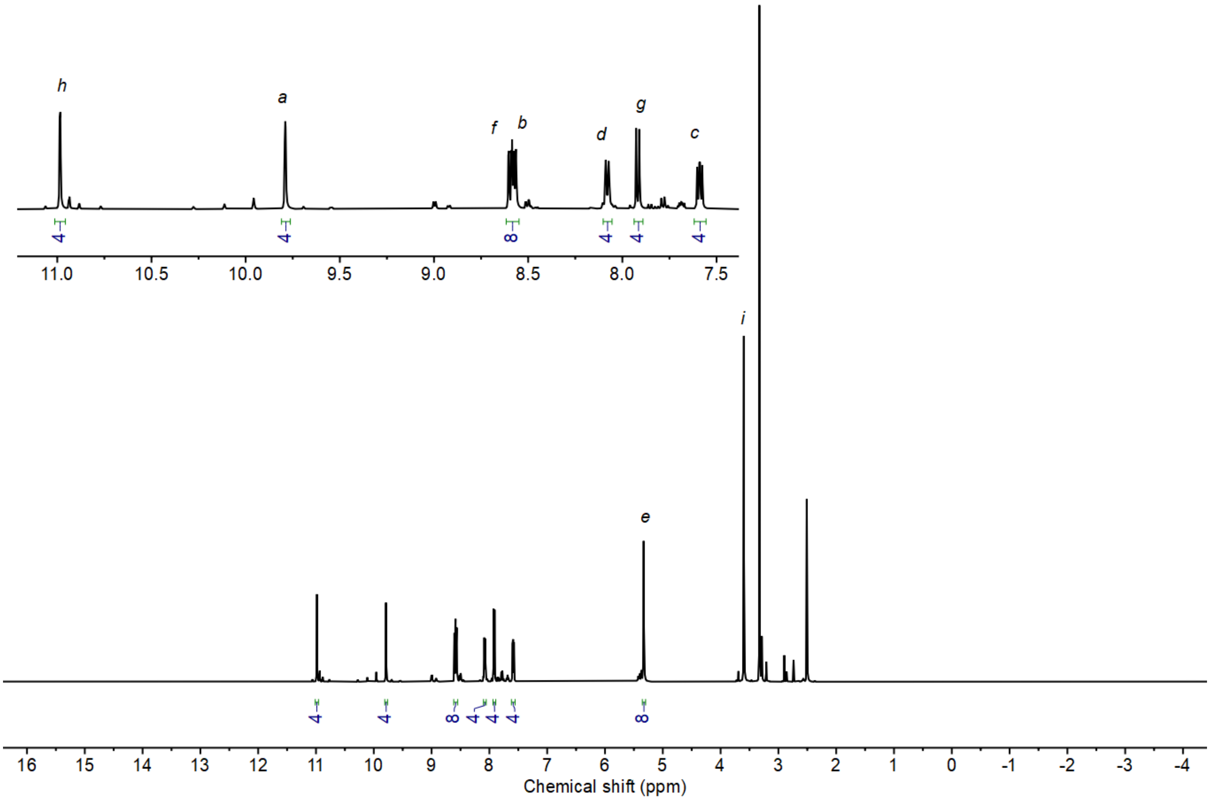


**Figure S61 $^{1}$H NMR (500 MHz, $d_6$-DMSO) of C1$^{P}$⊃Cl.**

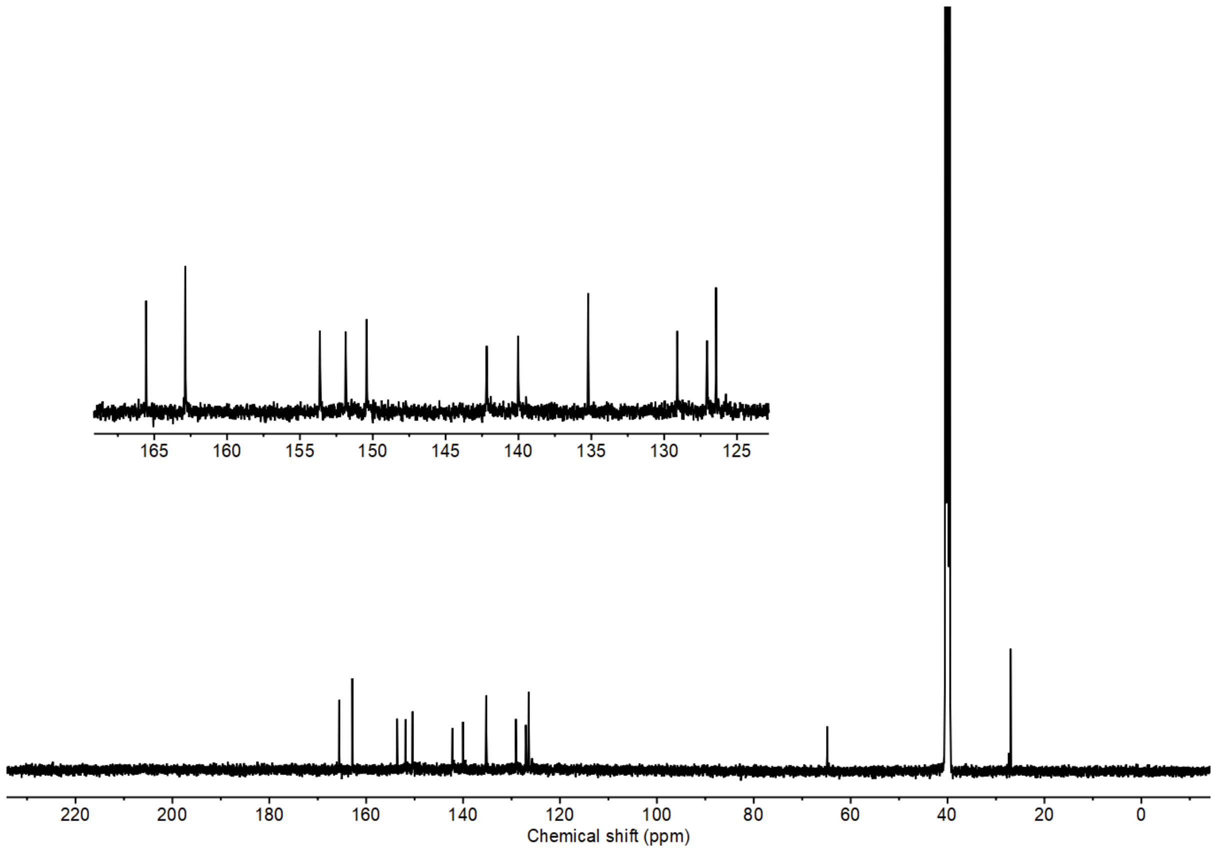


**Figure S62 $^{13}C$ NMR (126 MHz, $d_6$-DMSO) of C1$^{P}$⊃Cl.**

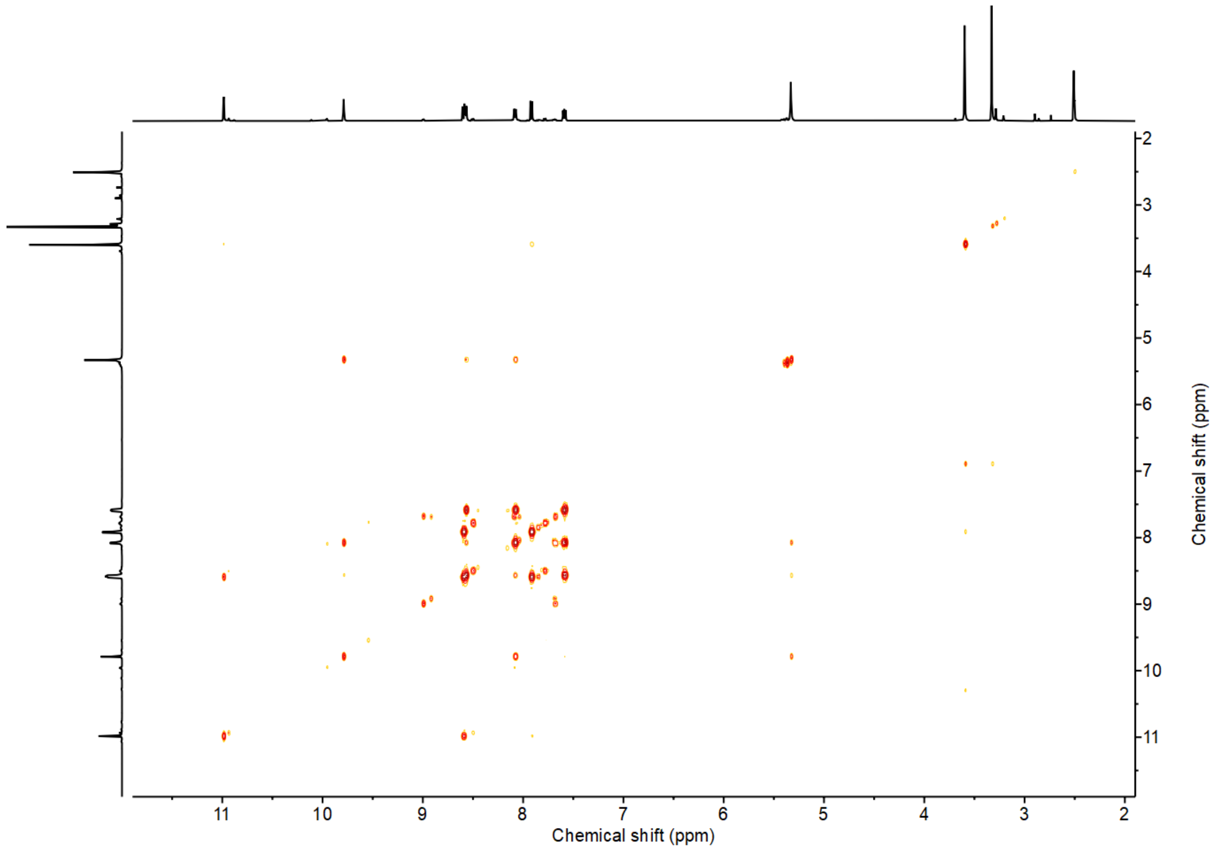


**Figure S63 COSY ($d_6$-DMSO) of C1$^{P}$⊃Cl.**

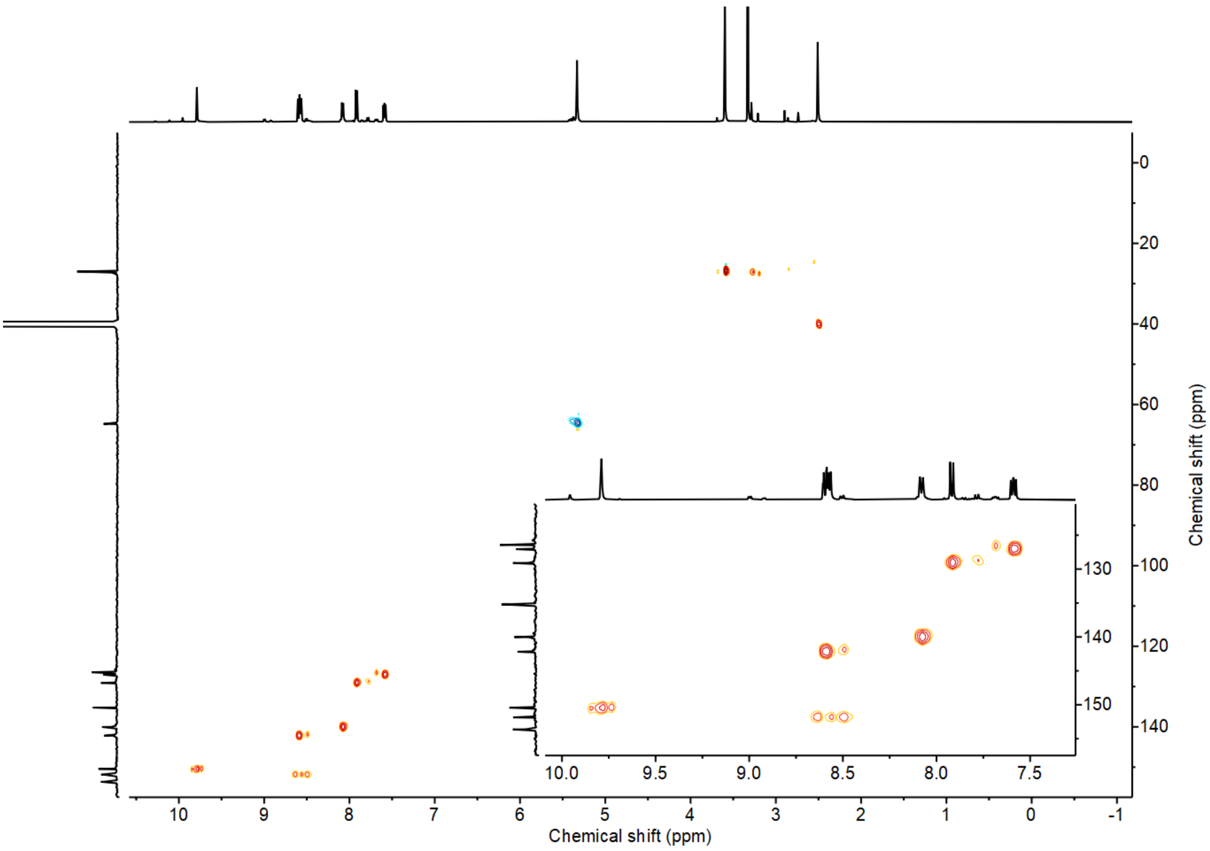


**Figure S64 HSQC ($d_6$-DMSO) of C1$^{P}$⊃Cl.**

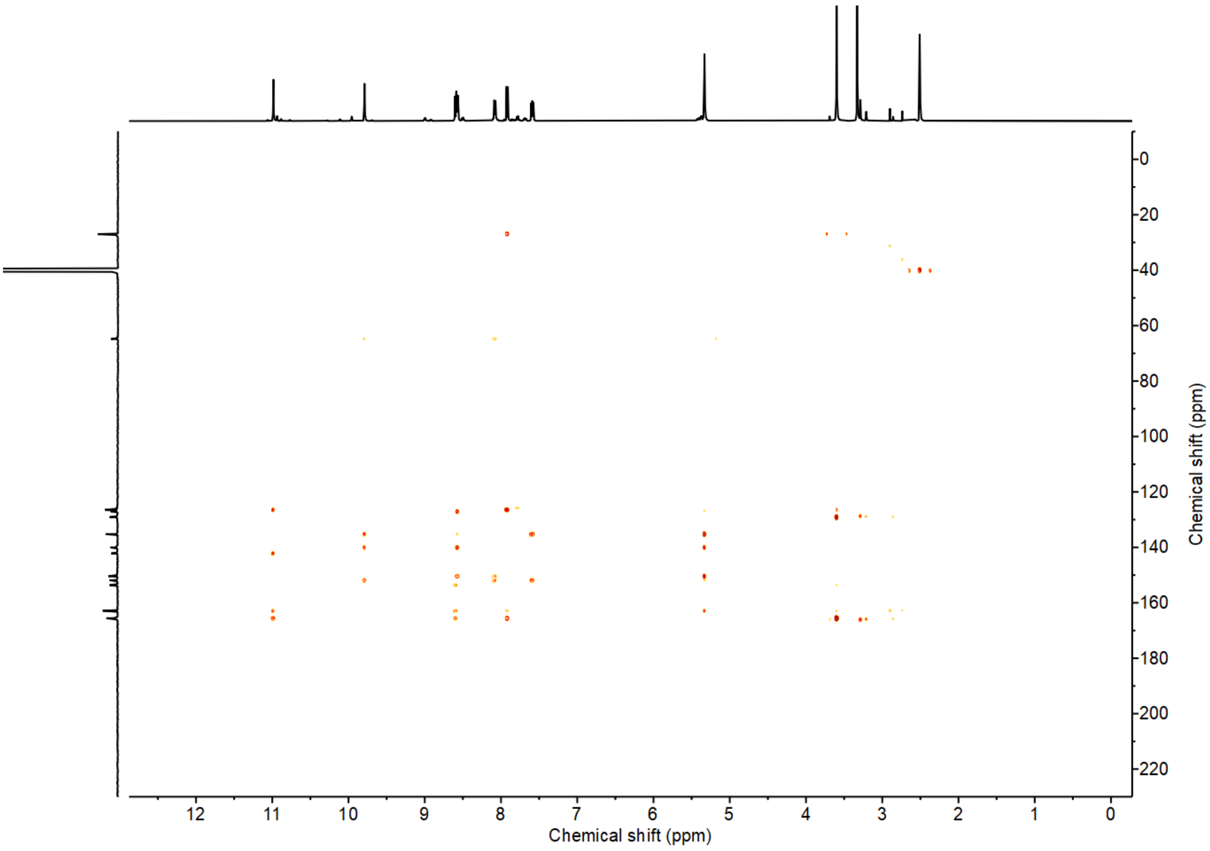


**Figure S65 HMBC ($d_6$-DMSO) of C1$^{P}$⊃Cl.**

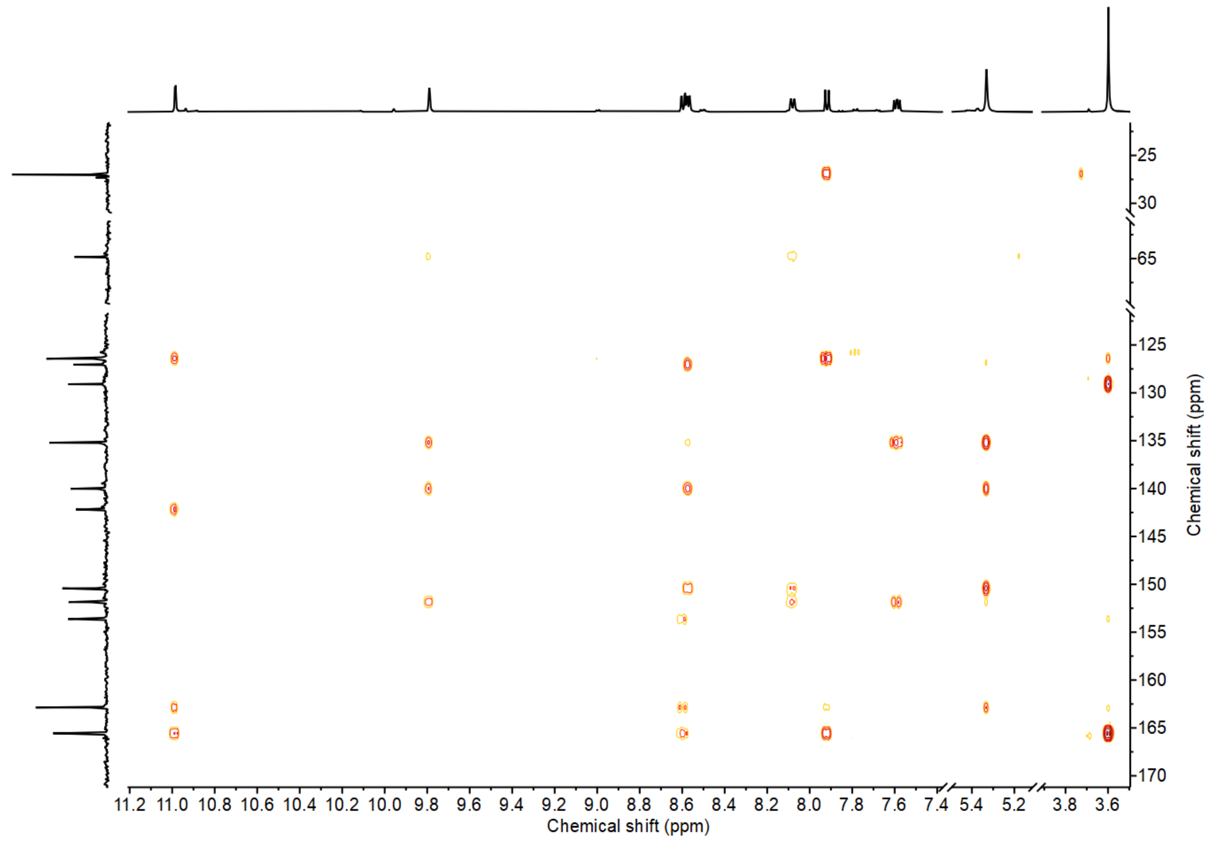


**Figure S66 Partial HMBC ($d_6$-DMSO) of C1$^{P}$⊃Cl.**

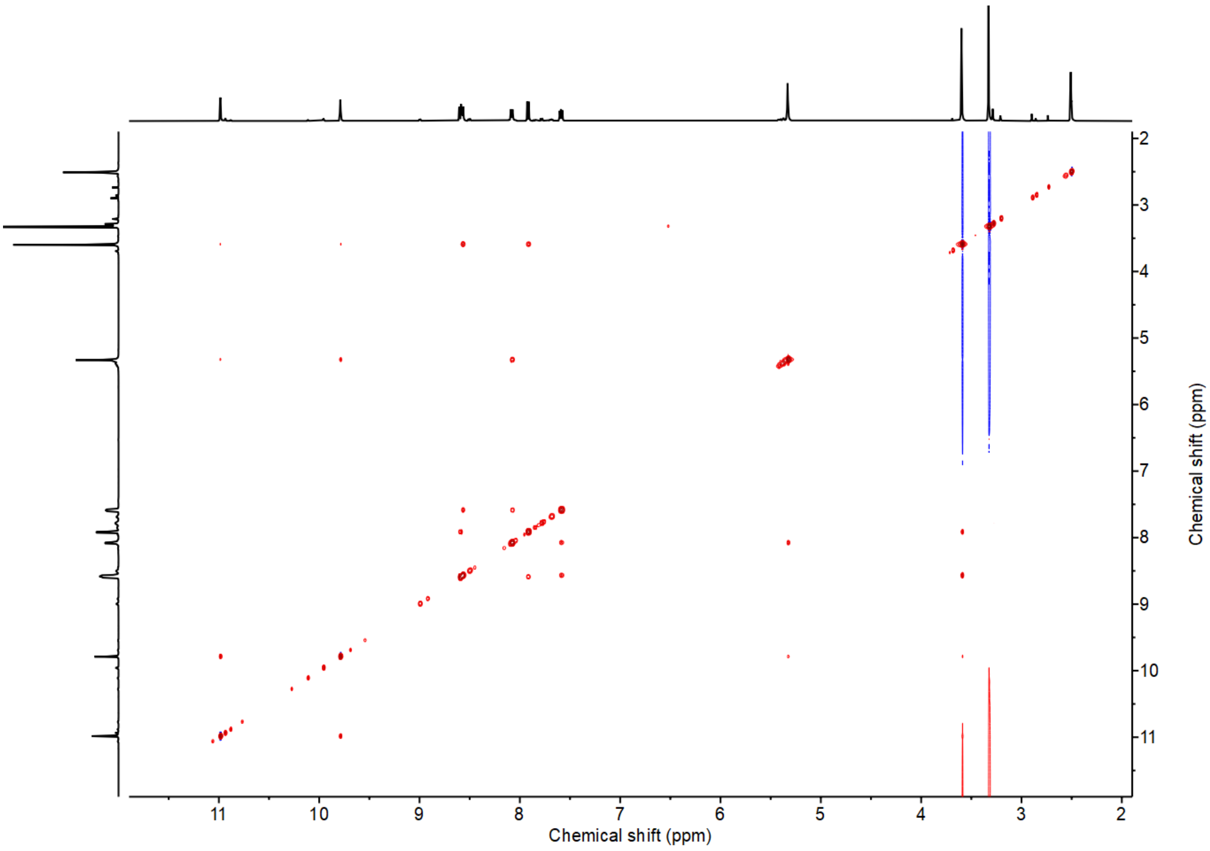


**Figure S67 NOESY ($d_6$-DMSO) of C1$^{P}$⊃Cl.**

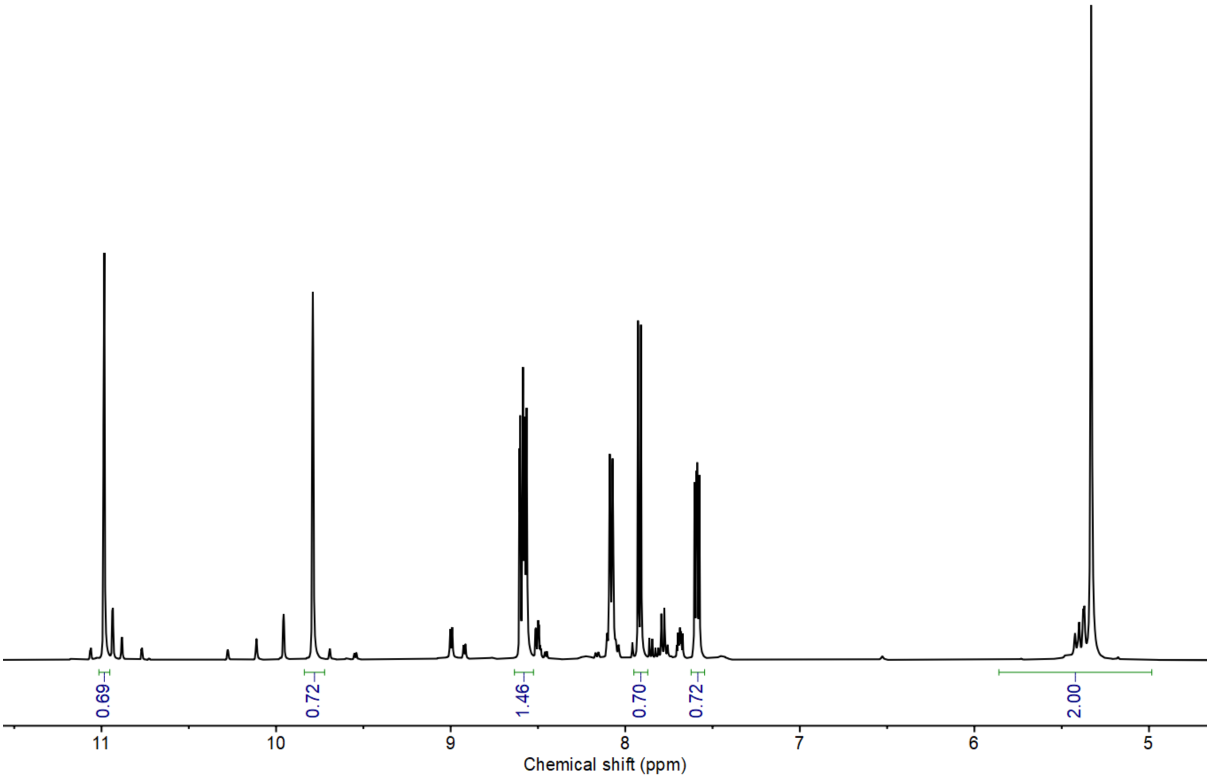


**Figure S68 Partial $^1$H NMR (500 MHz, $d_6$-DMSO) of C1$^P$⊃Cl with integrals relative to sum total of $CH_2$ signals.**

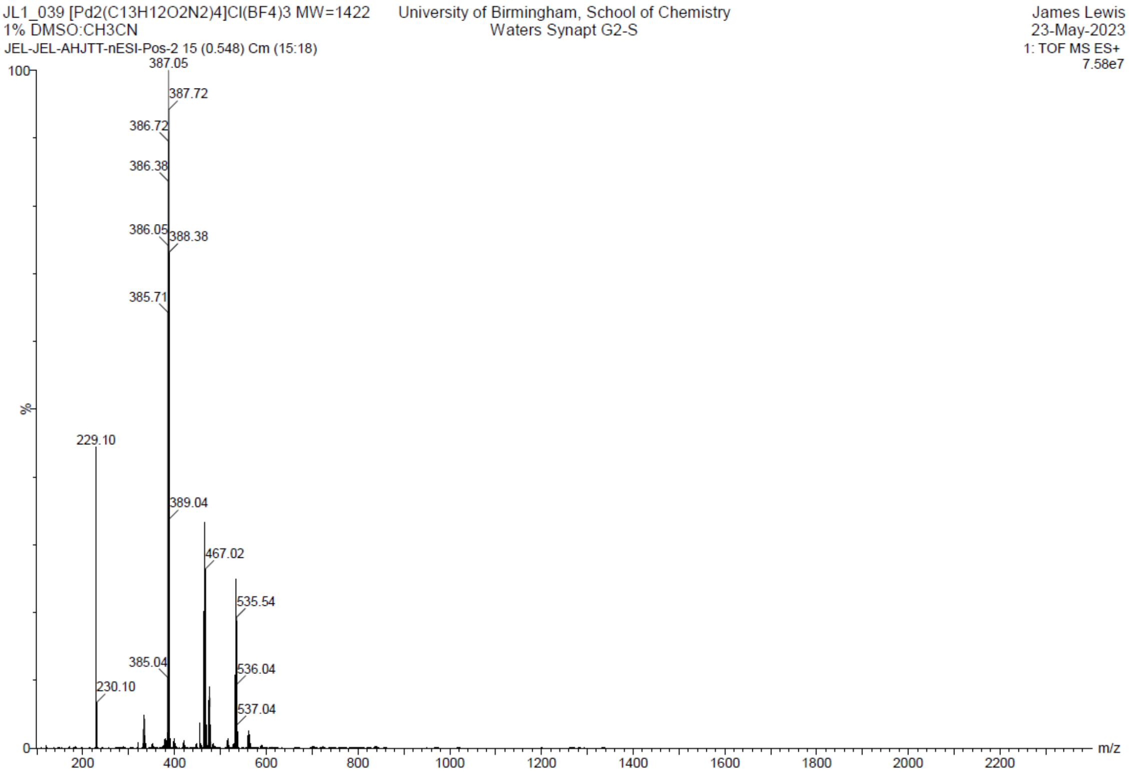


**Figure S69 ESI-MS of C1$^P$⊃Cl.**

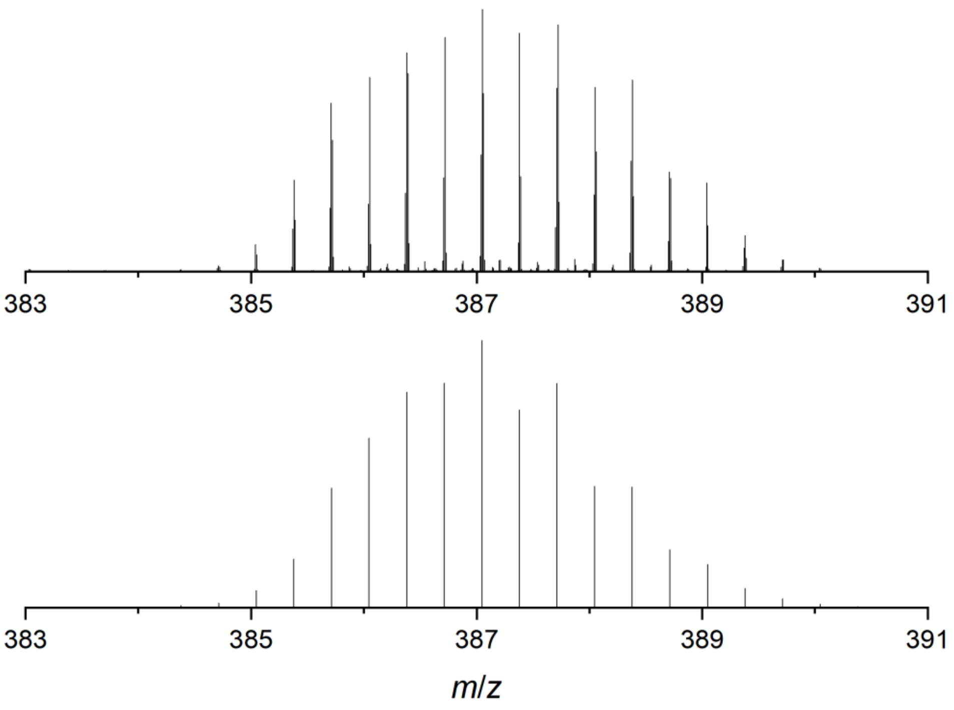


**Figure S70 Observed (top) and calculated (bottom) isotopic patterns for $\{[Pd_2(L1^P)_4]Cl\}^{3+}$.**

## S2.11 Investigation of anion and solvent effects on C1 speciation

### Addition of anions

**L1**$^{Q}$ (21.4 mg, 80 µmol, 1 eq.), $[Pd(CH_3CN)_4](BF_4)_2$ (19.5 mg, 44 µmol, 0.55 eq.) and $Bu_4NNO_3$ (12.2 mg, 40 µmol, 0.5 eq.) were combined in *d*$_6$-DMSO (2000 µL) and sonicated until a homogenous solution was obtained to give a stock solution with ostensible [**C1**$^{Q}$] = 10 mM.

From this stock solution were taken 3 × 650 µL aliquots.

One was transferred directly to a 5 mm NMR tube and stood at 60 °C for 6 h to give [Pd$_2$**L1**$^{Q}_4$⊃NO$_3$](BF$_4$)$_3$.

To one was added $Bu_4NOTf$ (25.4 mg, 65 µmol, 10 eq. per **C1**$^{Q}$), sonicated to give a homogenous solution, and then stood at 60 °C for 6 h.

To one was added $Bu_4NBF_4$ (21.4 mg, 65 µmol, 10 eq. per **C1**$^{Q}$), sonicated to give a homogenous solution, and then stood at 60 °C for 6 h.

All three solutions presented near-identical $^1$H NMR spectra with the same diastereoselectivity values, indicating no detectable impact of the increased anion concentration on the self-assembly.

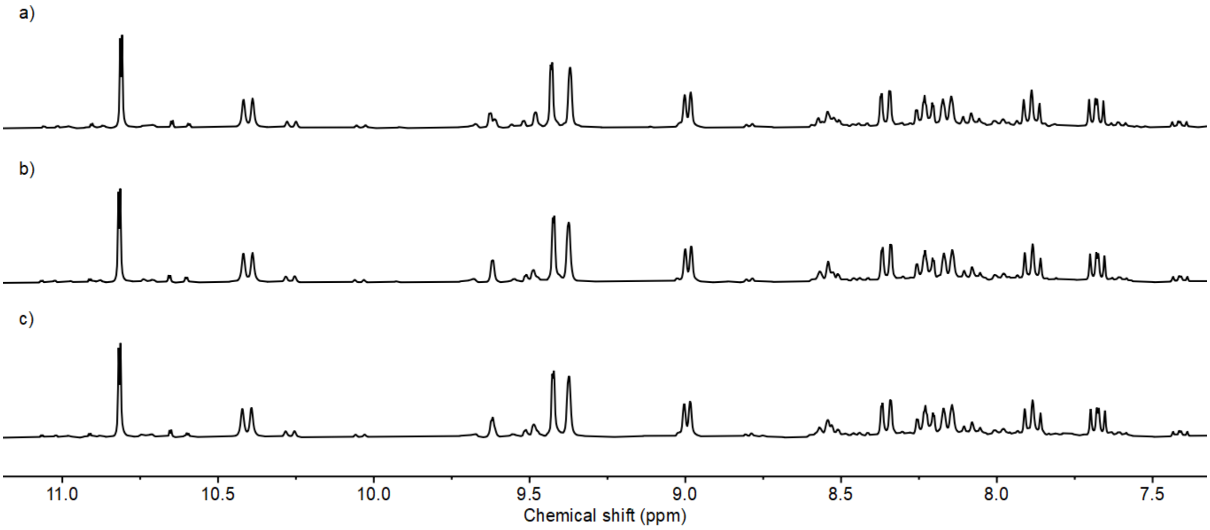


**Figure S71** $^1$**H NMR (300 MHz, *d*$_6$-DMSO) of [C1$^{Q}$⊃NO$_3$](BF$_4$)$_3$ prepared a) in the absence of additional anions, b) with 10 eq. of Bu$_4$NBF$_4$, and c) with 10 eq. Bu$_4$NOTf.**

## Dilution with water

To a 10 mM solution of $[Pd_2\mathbf{L1}^{Q}_4\supset NO_3](BF_4)_3$ in $d_6$-DMSO (650 µL) in a 5 mm NMR tube, prepared as described above, was added aliquots of $D_2O$, mixed thoroughly by a combination of vigorous shaking and sonication, before re-equilibrating at 60 °C for 4 h, and finally standing at rt for at least 12 h. $^1H$ NMR spectra were obtained following each iteration of this procedure.

Volumes of $D_2O$ aliquots added in each iteration were 1) 50 µL, 2) 100 µL, and 3) 150 µL, to give total volumes of 700 µL, 800 µL and 950 µL, respectively.

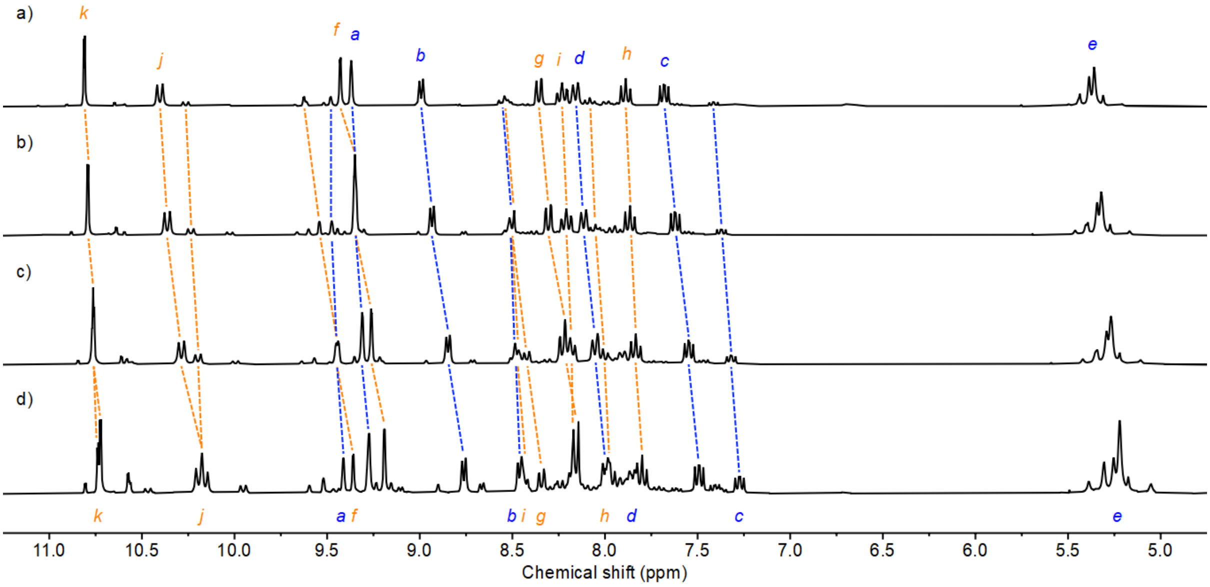


**Figure S72 Partial $^1H$ NMR ($d_6$-DMSO, 300 MHz) of $[Pd_2L1^{Q}_4\supset NO_3](BF_4)_3$ at a) $[C1^{Q}]$ = 10 mM in 650 µL of $d_6$-DMSO as solvent (*cis* isomer peaks labelled), and after addition of b) 50 µL of $D_2O$, c) 150 µL of $D_2O$ and d) 300 µL of $D_2O$ (*trans* isomer peaks labelled).**

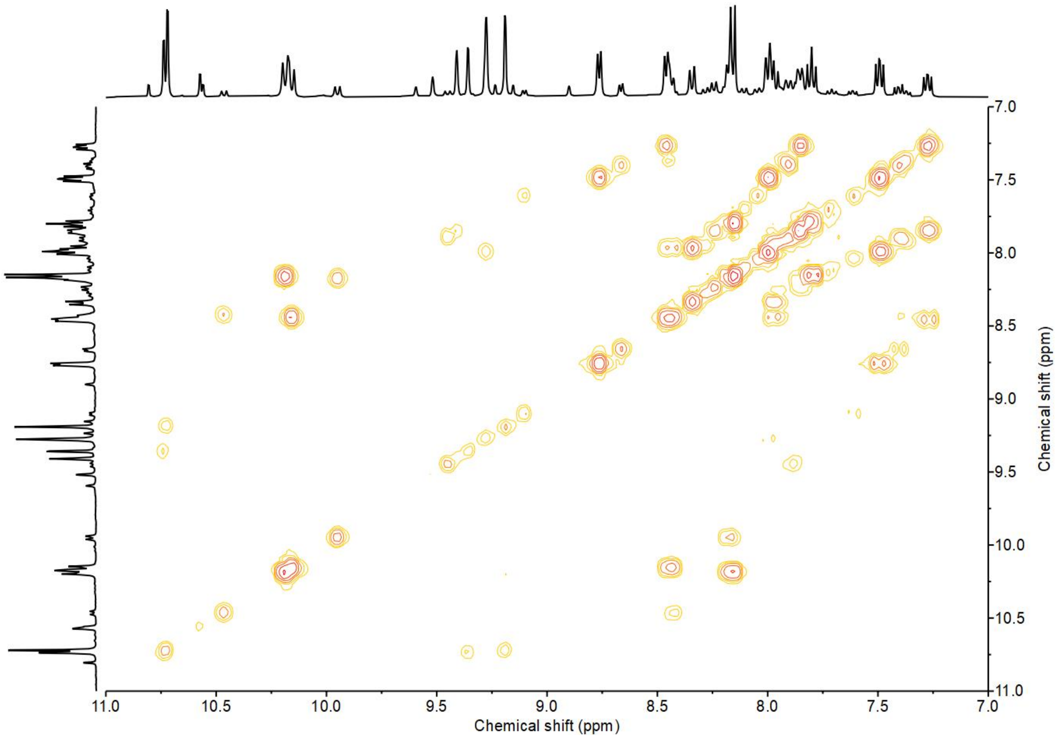


**Figure S73 Partial COSY (400 MHz, 13:6 $d_6$-DMSO/$D_2O$) of $[Pd_2L1^{Q}_4\supset NO_3](BF_4)_3$ at $[C1^{Q}]$ = 6.8 mM.**

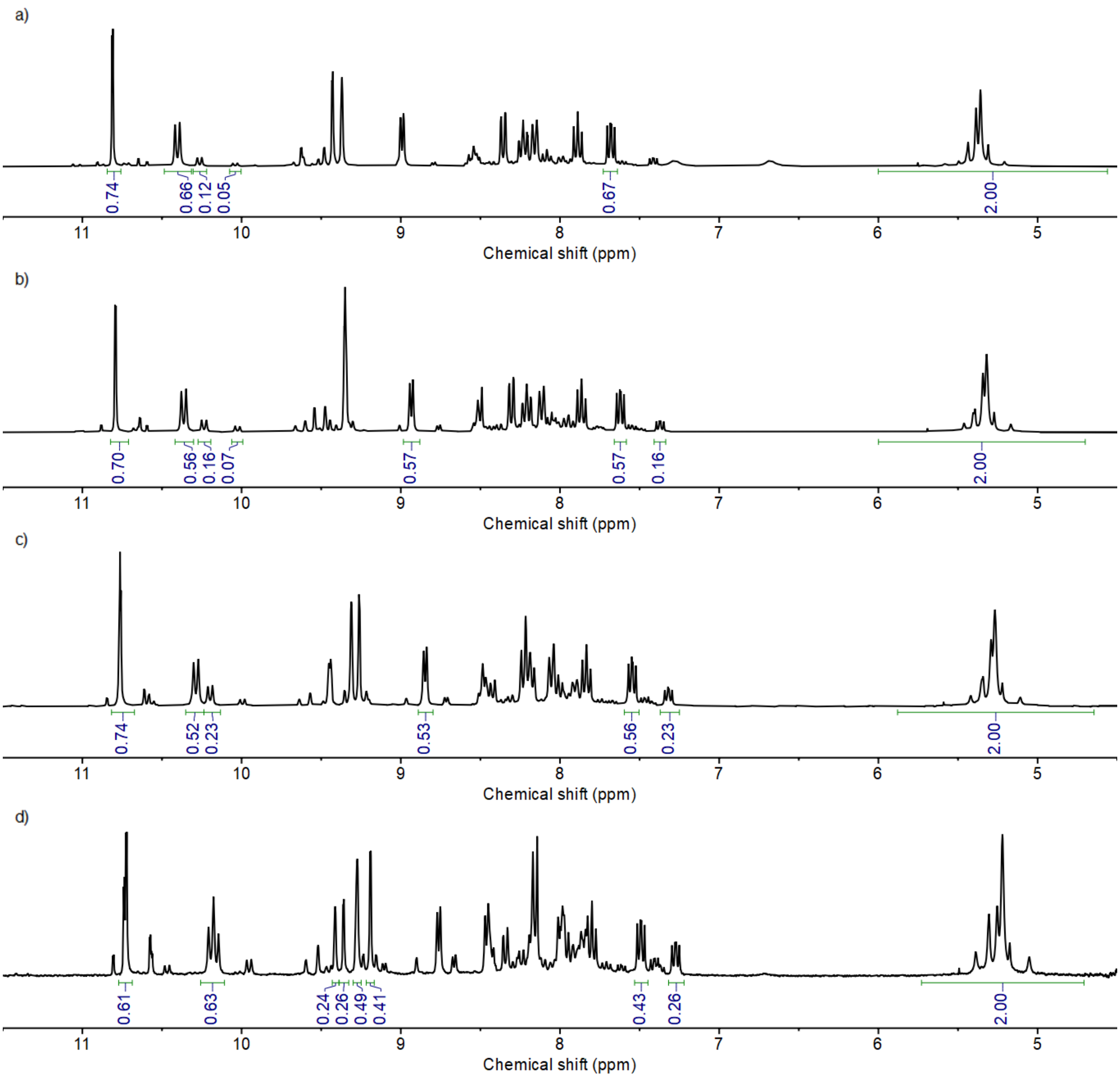


**Figure S74 Partial $^{1}H$ NMR ($d_6$-DMSO, 300 MHz) of $[Pd_2L1^{♀}_4\supset NO_3](BF_4)_3$ at a) $[C1^{♀}]$ = 10 mM in 650 µL of $d_6$-DMSO as solvent, and after addition of b) 50 µL of $D_2O$, c) 150 µL of $D_2O$ and d) 300 µL of $D_2O$.**

**Dilution of $d_6$-DMSO solution**

$Pd(NO_3)_2 \cdot 2H_2O$ (4.0 mg, 15 µmol, eq.) and either **L1$^{Q}$** (7.9 mg, 30 µmol, 2 eq.) or **L1$^{P}$** (6.8 mg, 30 µmol, 2 eq.) were combined in $d_6$-DMSO (0.75 mL), sonicated to give a homogenous solution, and then stood at 60 °C for 5 h to give 10 mM stock solutions of **C1$^{Q}$** and **C1$^{P}$**, respectively.

For both dilution experiments, NMR spectra were referenced against the DMSO solvent signal ($^1H$ δ = 2.50 ppm).

**<u>$d_6$-DMSO</u>**

An aliquot of the stock **C1** solution in $d_6$-DMSO was diluted with $d_6$-DMSO to give a volume of 600 µL, mixed thoroughly by a combination of vigorous shaking and sonication, before re-equilibrating at 60 °C for 4 h, and finally standing at rt for at least 12 h. $^1H$ NMR spectra were obtained following each iteration of this procedure.

For both **C1$^{Q}$** and **C1$^{P}$**, no impact of decreased cage concentration in pure $d_6$-DMSO solution was observed.

**C1$^{Q}$:**

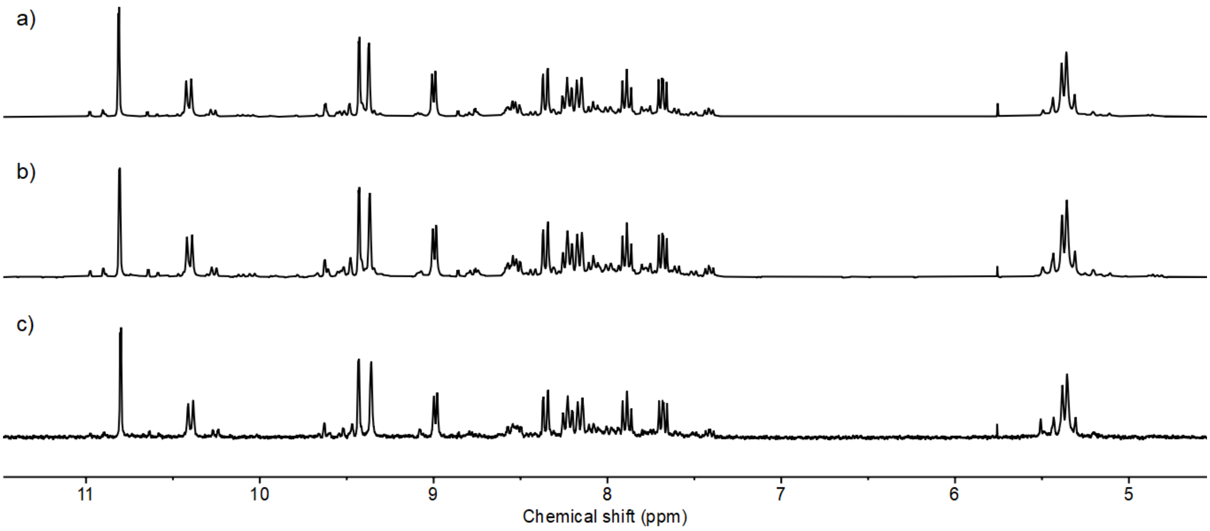


**Figure S75 Partial $^1H$ NMR ($d_6$-DMSO, 300 MHz) of $[Pd_2L1^{Q}_4 \supset NO_3](BF_4)_3$ at [C1$^{Q}$] = a) 10 mM, b) 5 mM, and c) 1 mM.**

**C1$^{P}$:**

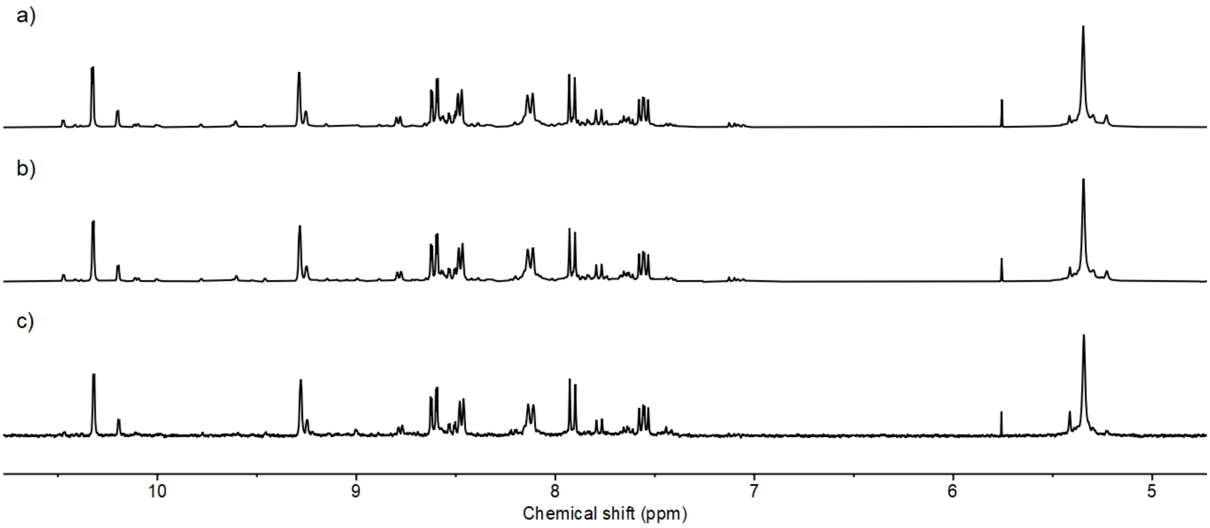


**Figure S76 Partial $^{1}$H NMR ($d_6$-DMSO, 300 MHz) of [$Pd_2L1^{P}_4$⊃$NO_3$]$(BF_4)_3$ at [C1$^{P}$] = a) 10 mM, b) 5 mM, and c) 1 mM.**

<u>$CD_3CN$</u>

An aliquot of the stock **C1** solution in $d_6$-DMSO was diluted with $CD_3CN$ to give a volume of 600 µL, mixed thoroughly by a combination of vigorous shaking and sonication, before re-equilibrating at 60 °C for 4 h, and finally standing at rt for at least 12 h. $^{1}$H NMR spectra were obtained following each iteration of this procedure. All NMRs were referenced to the DMSO solvent peak.

**C1$^{Q}$:**

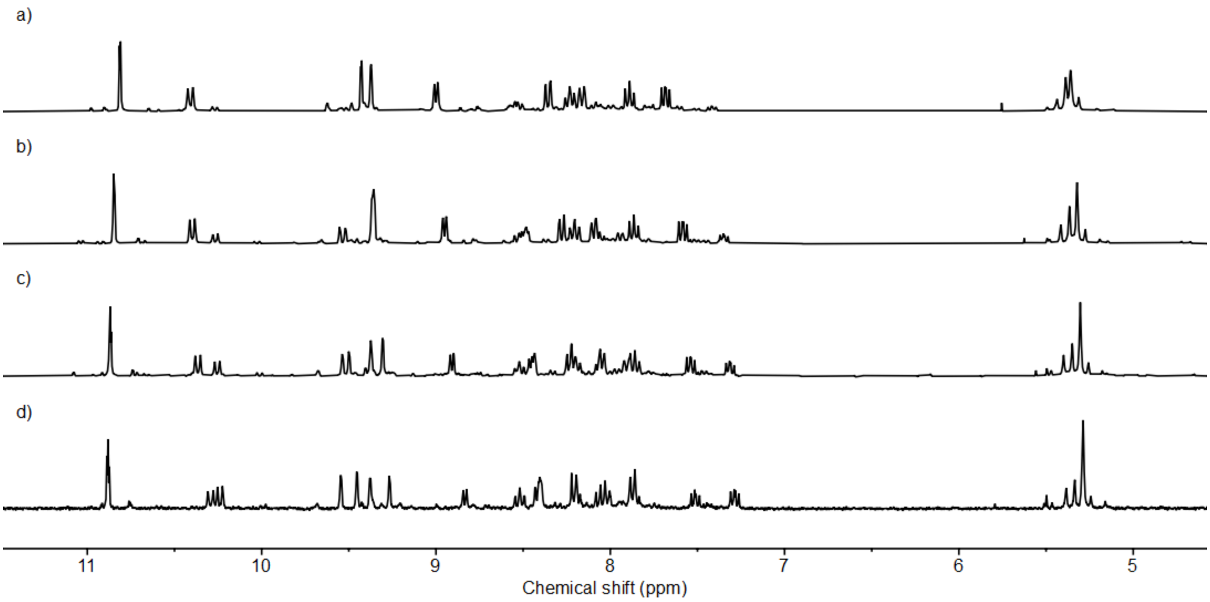


**Figure S77 Partial $^{1}$H NMR (300 MHz) of C1$^{Q}$ upon dilution of $d_6$-DMSO solution with $CD_3CN$: a) [C1$^{Q}$] = 10 mM ($d_6$-DMSO), b) [C1$^{Q}$] = 5 mM (1:1 $d_6$-DMSO/$CD_3CN$), c) [C1$^{Q}$] = 2.5 mM (1:3 $d_6$-DMSO/$CD_3CN$), and d) [C1$^{Q}$] = 1 mM (1:9 $d_6$-DMSO/$CD_3CN$). Intensity normalised to $H_k$ peak.**

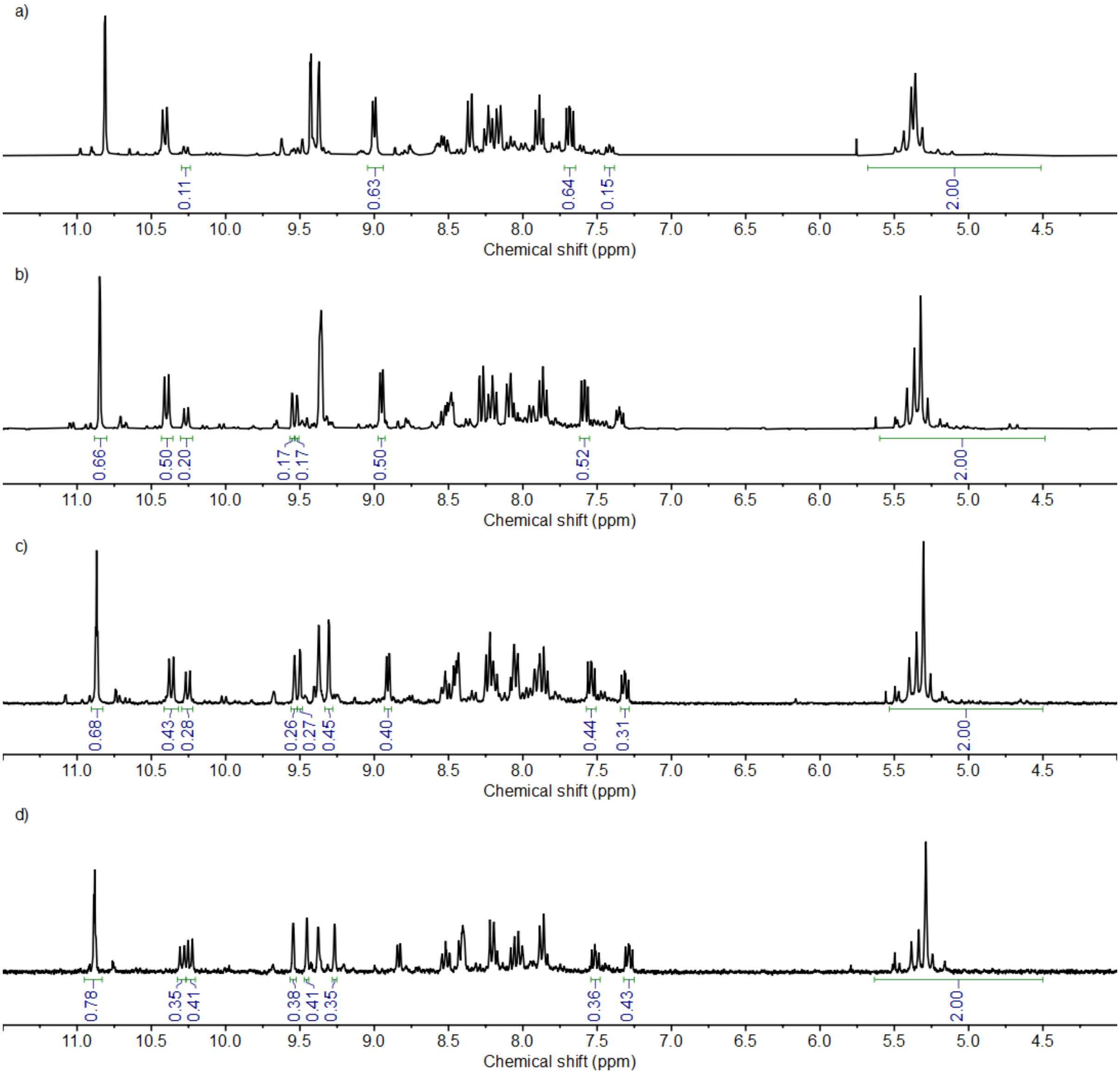


**Figure S78 Partial $^{1}$H NMR (300 MHz) of $C1^{Q}$ upon dilution of $d_6$-DMSO solution with $CD_3CN$ with integrals of peaks for both *cis* and *trans* isomers. a) [$C1^{Q}$] = 10 mM ($d_6$-DMSO), b) [$C1^{Q}$] = 5 mM (1:1 $d_6$-DMSO/$CD_3CN$), c) [$C1^{Q}$] = 2.5 mM (1:3 $d_6$-DMSO/$CD_3CN$), and d) [$C1^{Q}$] = 1 mM (1:9 $d_6$-DMSO/$CD_3CN$). The *cis*/*trans* ratio fell from ~6 to ~3 to ~1.5 and finally ~0.8 with these dilutions.**

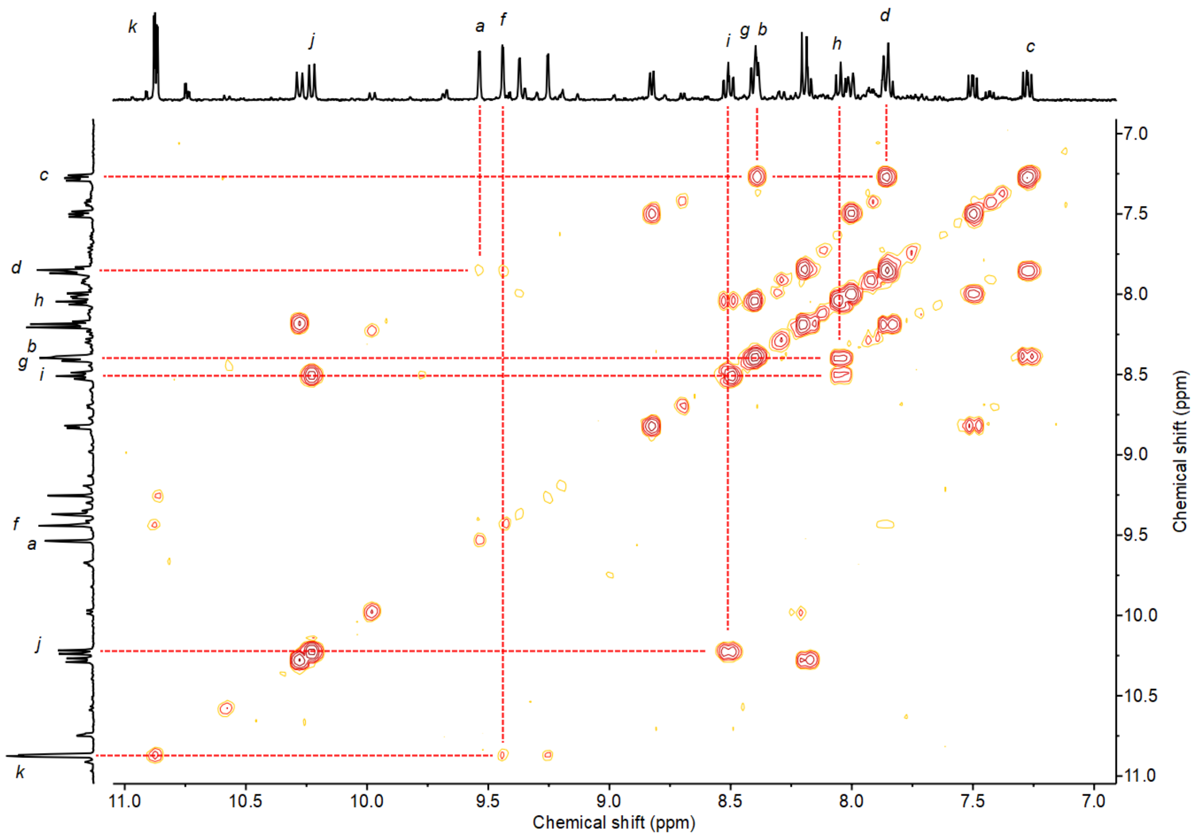


**Figure S79 Partial COSY (400 MHz, 9:1 $CD_3CN$/$d_6$-DMSO) of 1 mM C1$^{Q}$ with peaks for *trans* isomer labelled.**

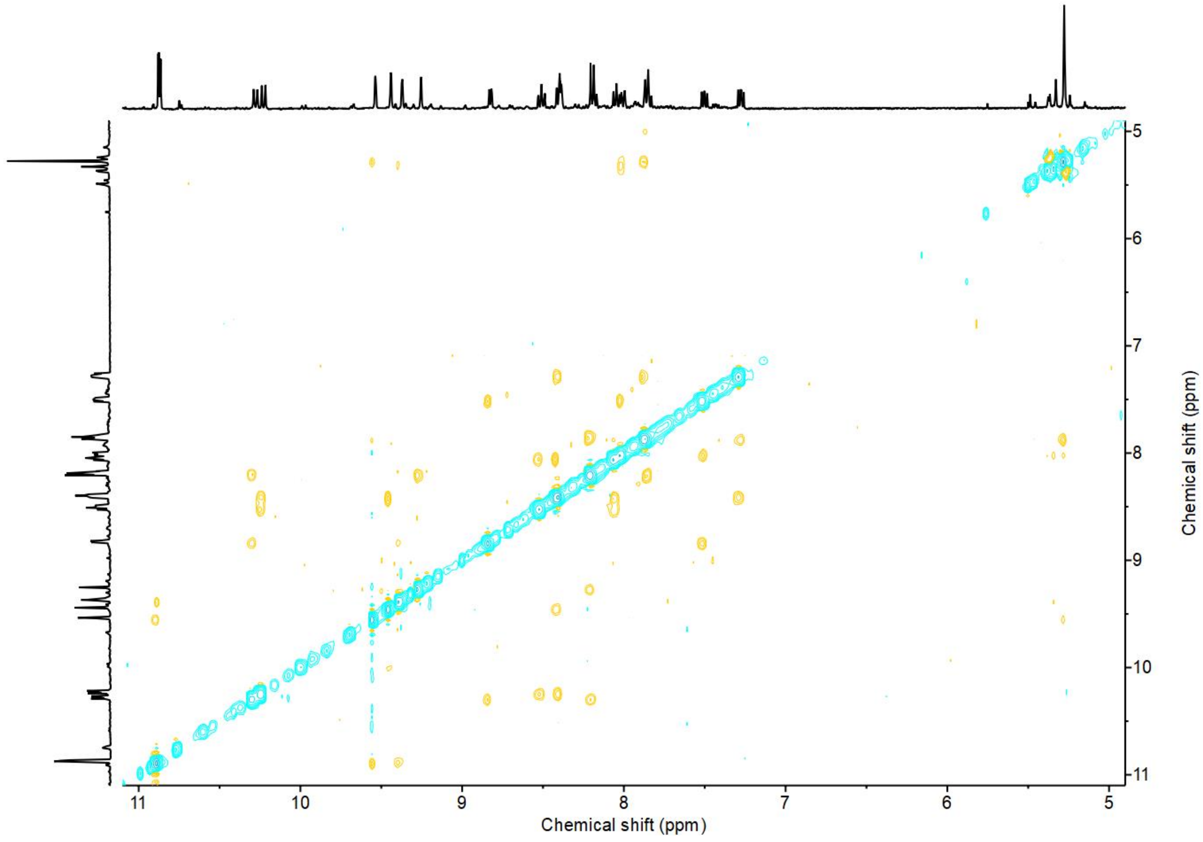


**Figure S80 Partial NOESY (400 MHz, 9:1 $CD_3CN$/$d_6$-DMSO) of 1 mM C1$^{Q}$.**

**C1$^P$:**

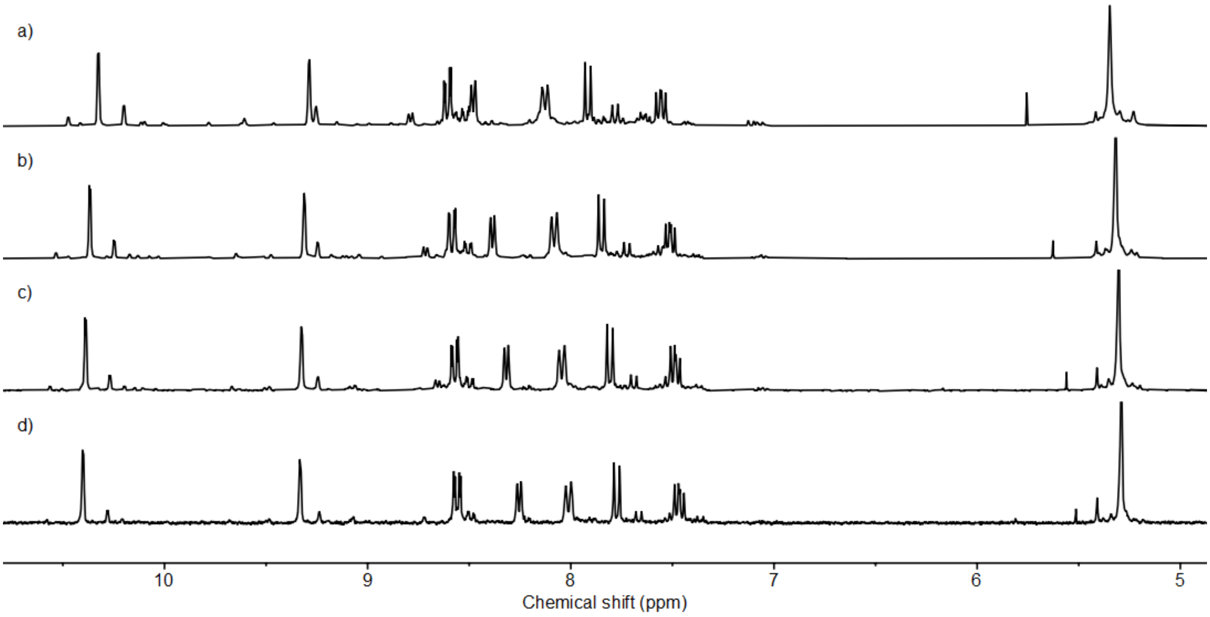


**Figure S81 Partial $^1$H NMR (300 MHz) of C1$^P$ upon dilution of *d*$_6$-DMSO solution with $CD_3CN$: a) [C1$^P$] = 10 mM (*d*$_6$-DMSO), b) [C1$^P$] = 5 mM (1:1 *d*$_6$-DMSO/$CD_3CN$), c) [C1$^P$] = 2.5 mM (1:3 *d*$_6$-DMSO/$CD_3CN$), and d) [C1$^P$] = 1 mM (1:9 *d*$_6$-DMSO/$CD_3CN$). Intensity normalised to *trans*-C1$^P$ $H_k$ peak.**

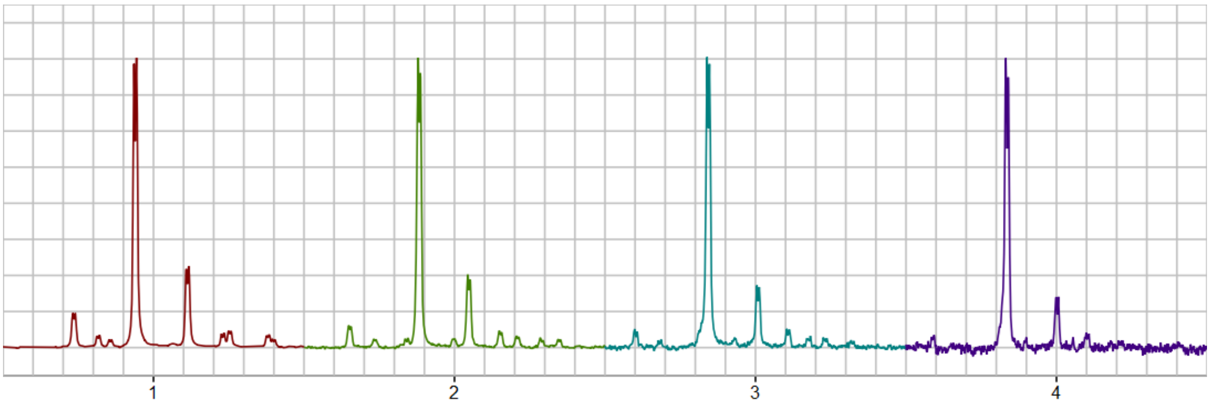


**Figure S82 Partial $^1$H NMR (300 MHz) of C1$^P$ showing the $H_k$ signal region with reduction of non-*trans* peak intensities upon dilution of *d*$_6$-DMSO solution with $CD_3CN$: 1) [C1$^P$] = 10 mM (*d*$_6$-DMSO), 2) [C1$^P$] = 5 mM (1:1 *d*$_6$-DMSO/$CD_3CN$), 3) [C1$^P$] = 2.5 mM (1:3 *d*$_6$-DMSO/$CD_3CN$), and 4) [C1$^P$] = 1 mM (1:9 *d*$_6$-DMSO/$CD_3CN$). Intensity normalised to *trans*-C1$^P$ $H_k$ peak.**

## S2.12 Synthesis of S1

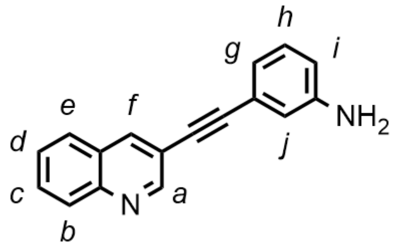


3-Bromoquinoline (0.624 g, 3 mmol, 1 eq.), 3-ethynylaniline (0.387 g, 3.3 mmol, 1.1 eq.), $Pd(PPh_3)_2Cl_2$ (0.053 g, 0.075 mmol, 2.5 mol%) and CuI (0.029 mg, 0.15 mmol, 5 mol%) were stirred at 80 °C in $^i$Pr$_2$NH (12 mL) for 19 h. 0.1 M EDTA$_{(aq)}$ (20 mL) was added, and the aqueous phase extracted with $CH_2Cl_2$ (3 × 20 mL). The combined organic phases were dried ($MgSO_4$) and the solvent removed *in vacuo*. After purification by column chromatography on silica gel (1:19 acetone/$CH_2Cl_2$) the product was obtained as a yellow solid (0.534 g, 73%).

**$^1$H NMR** (500 MHz, $CDCl_3$) δ: 8.99 (d, *J* = 2.1 Hz, 1H, H$_a$), 8.31 (d, *J* = 1.9 Hz, 1H, H$_f$), 8.13 (d, *J* = 8.5 Hz, 1H, H$_b$), 7.80 (d, *J* = 8.2 Hz, 1H, H$_e$), 7.73 (ddd, *J* = 8.4, 6.9, 1.4 Hz, 1H, H$_c$), 7.58 (ddd, *J* = 8.1, 6.8, 1.2 Hz, 1H, H$_d$), 7.16 (app. t, *J* = 7.8 Hz, 1H, H$_h$), 7.00 (app. dt, *J* = 7.5, 1.3 Hz, 1H, H$_g$/H$_i$), 6.92 (app. t, *J* = 2.0 Hz, 1H, H$_j$), 6.71 (ddd, *J* = 8.1, 2.4, 0.8 Hz, 1H, H$_g$/H$_i$), 4.04 (s, 2H H$_{NH}$).

**$^{13}$C NMR** (126 MHz, $CDCl_3$) δ: 151.9 (C$_a$), 146.4, 146.4 (C$_f$), 138.7 (C$_c$), 130.4 (C$_h$), 129.6 (C$_b$), 129.1, 127.8 (C$_e$), 127.6 (C$_d$), 127.5, 123.3, 122.4, 118.0 (C$_g$/C$_i$), 117.8 (C$_j$), 116.1 (C$_g$/C$_i$), 93.3, 85.9.

**HR-EI-MS** *m/z* = 244.0995 $[M]^+$ calc. 244.0995.

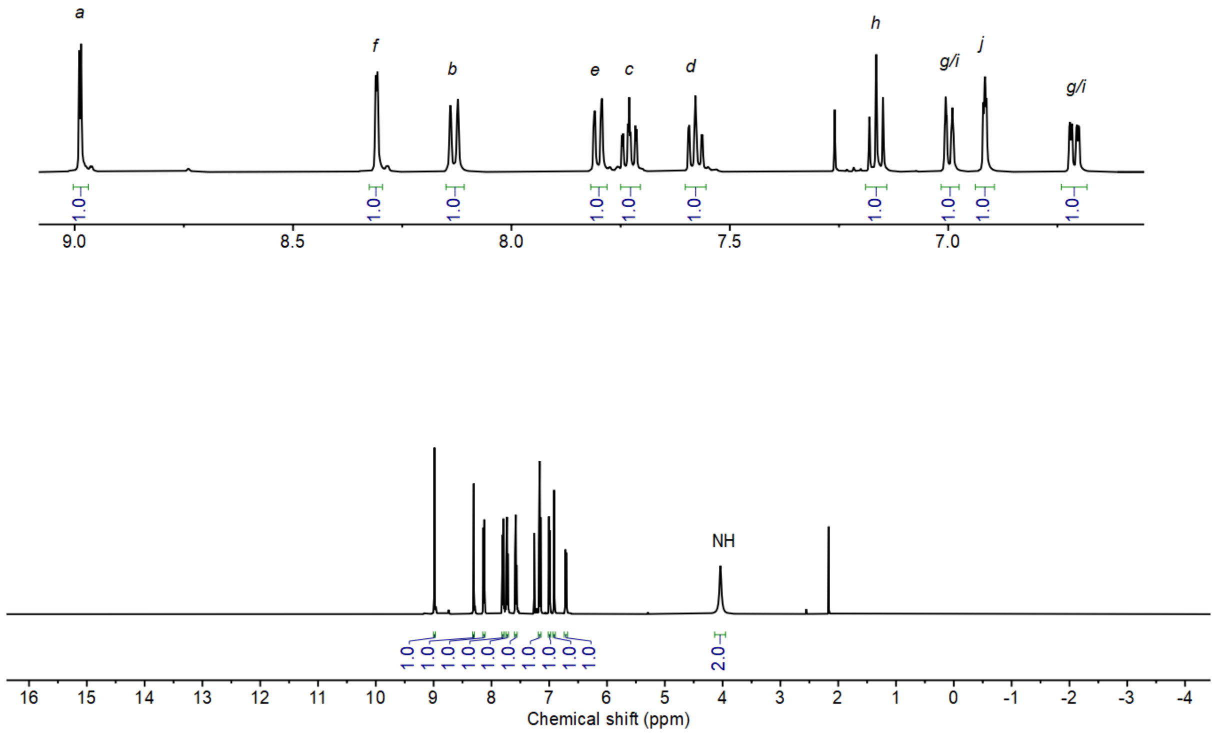


**Figure S83 $^{1}H$ NMR (500 MHz, $CDCl_3$) of S1.**

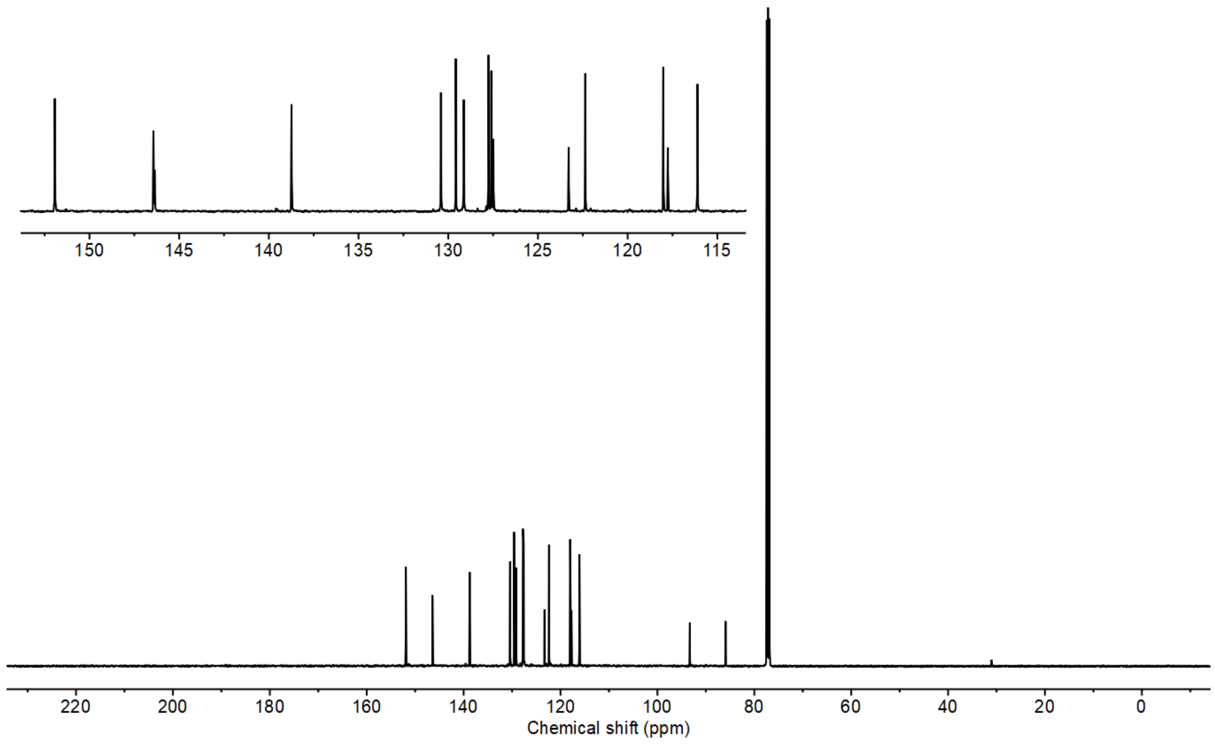


**Figure S84 $^{13}C$ NMR (126 MHz, $CDCl_3$) of S1.**

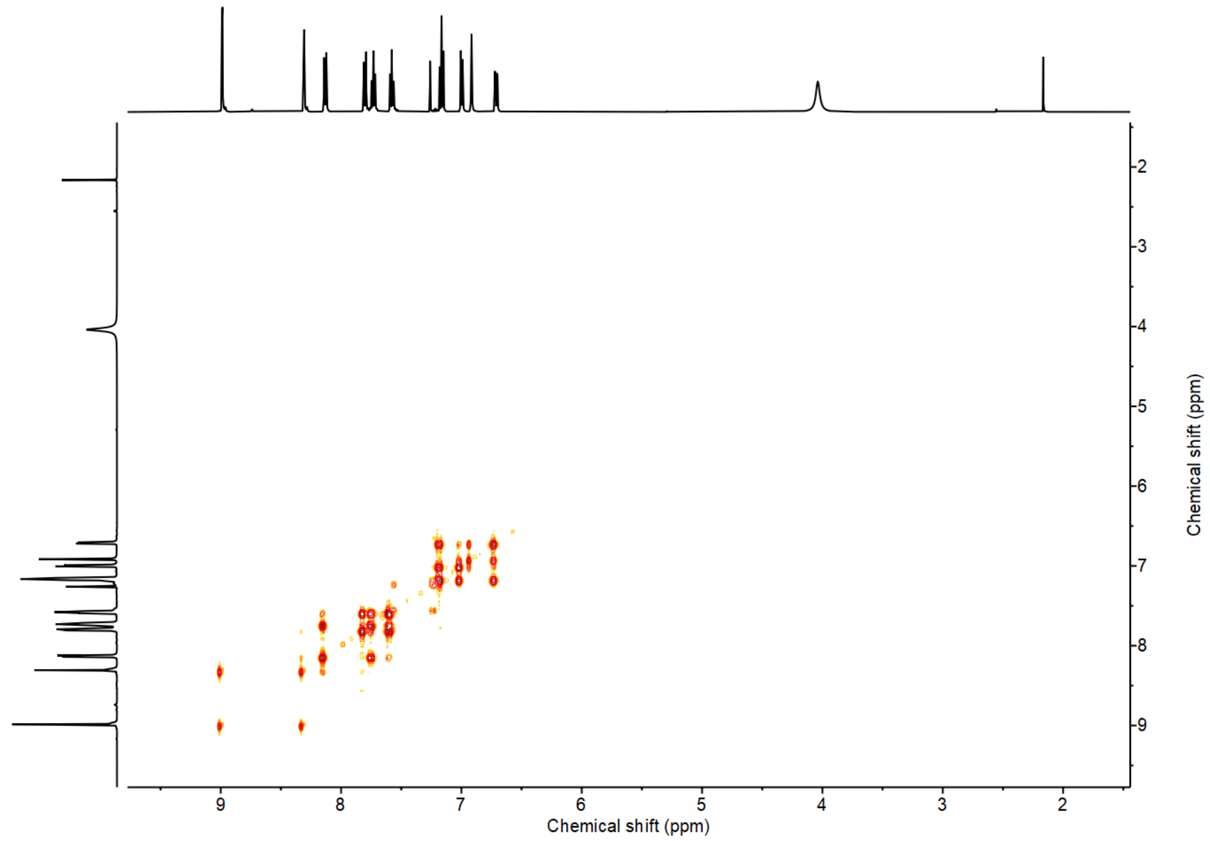


**Figure S85 COSY ($CDCl_3$) of S1.**

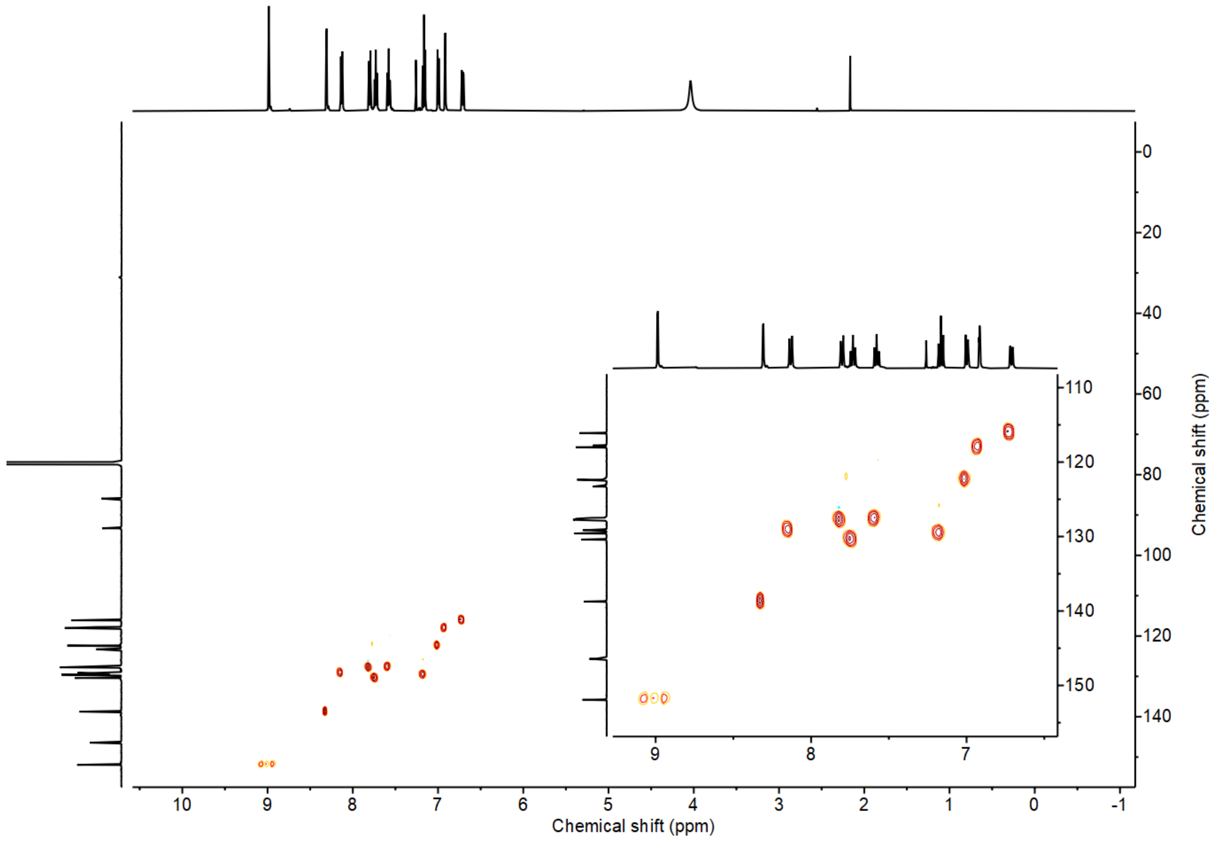


**Figure S86 HSQC ($CDCl_3$) of S1.**

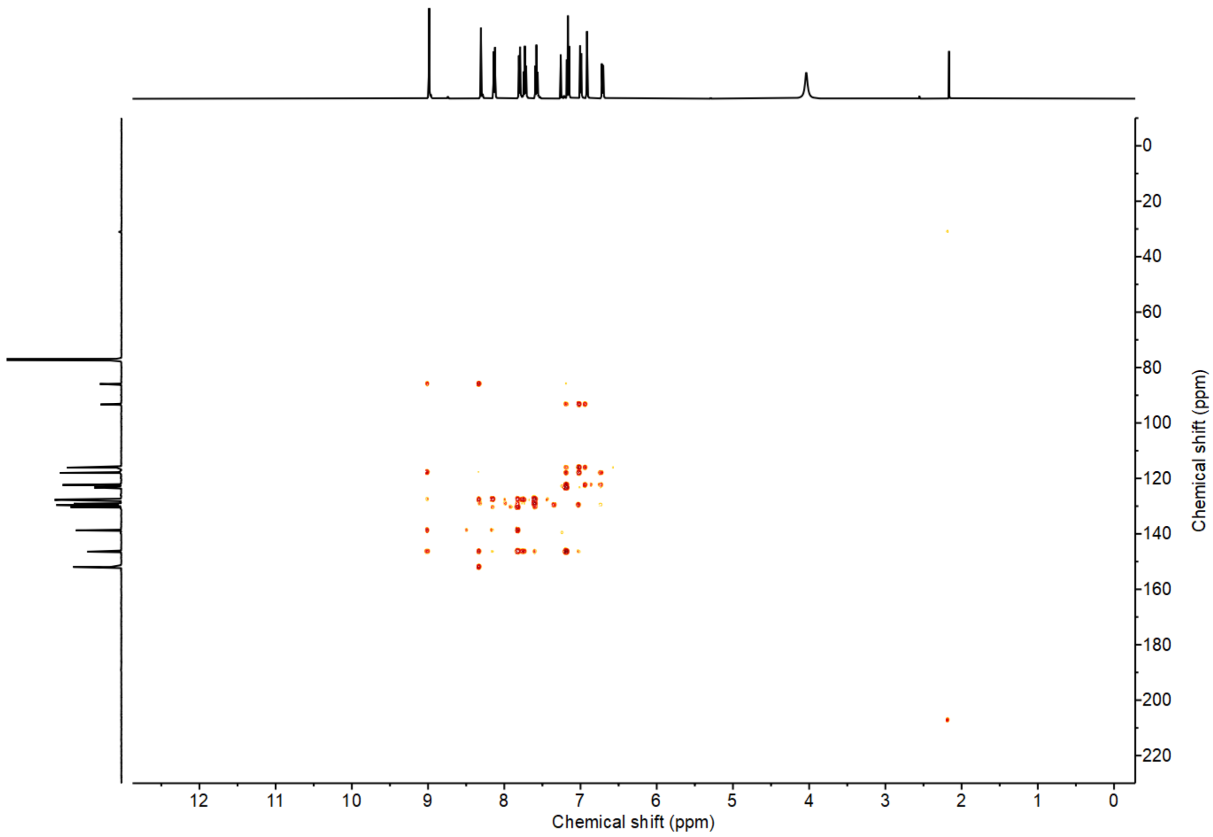


**Figure S87 HMBC ($CDCl_3$) of S1.**

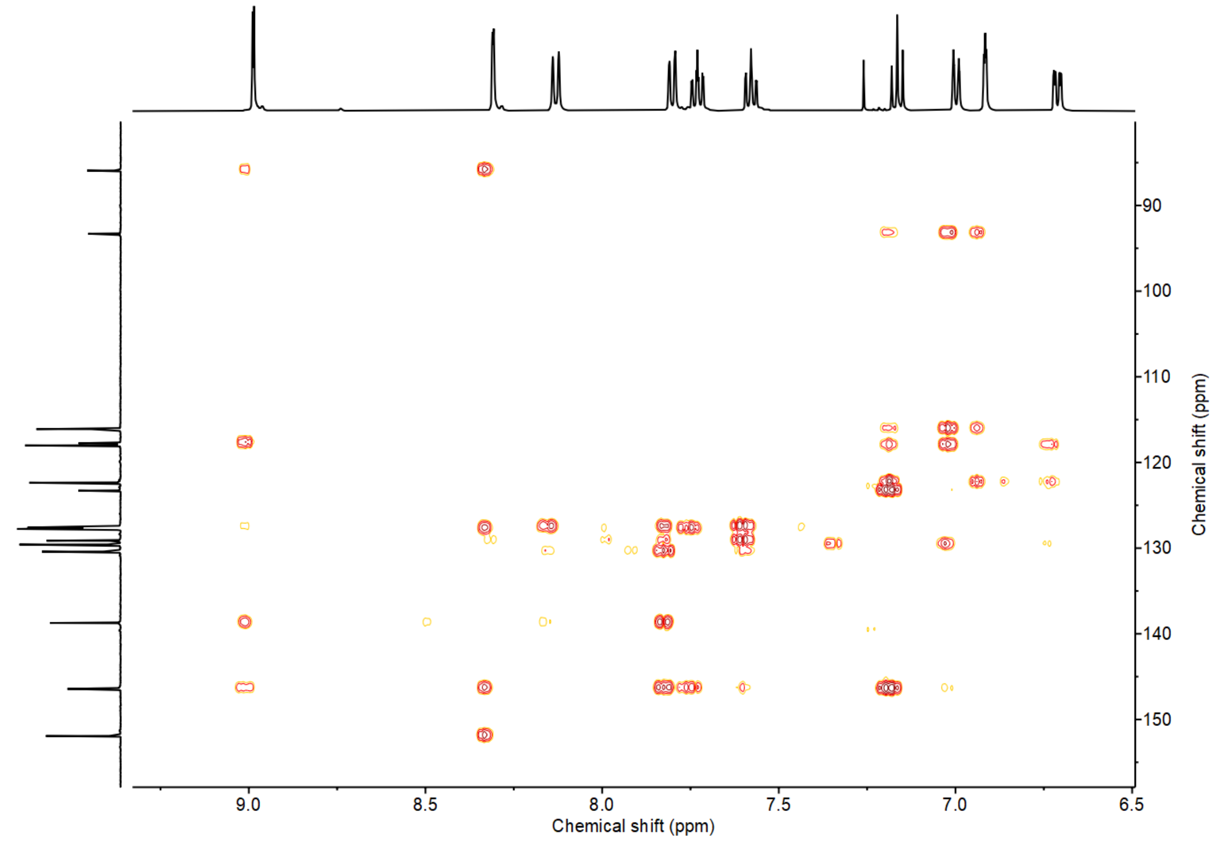


**Figure S88 Partial HMBC ($CDCl_3$) of S1.**

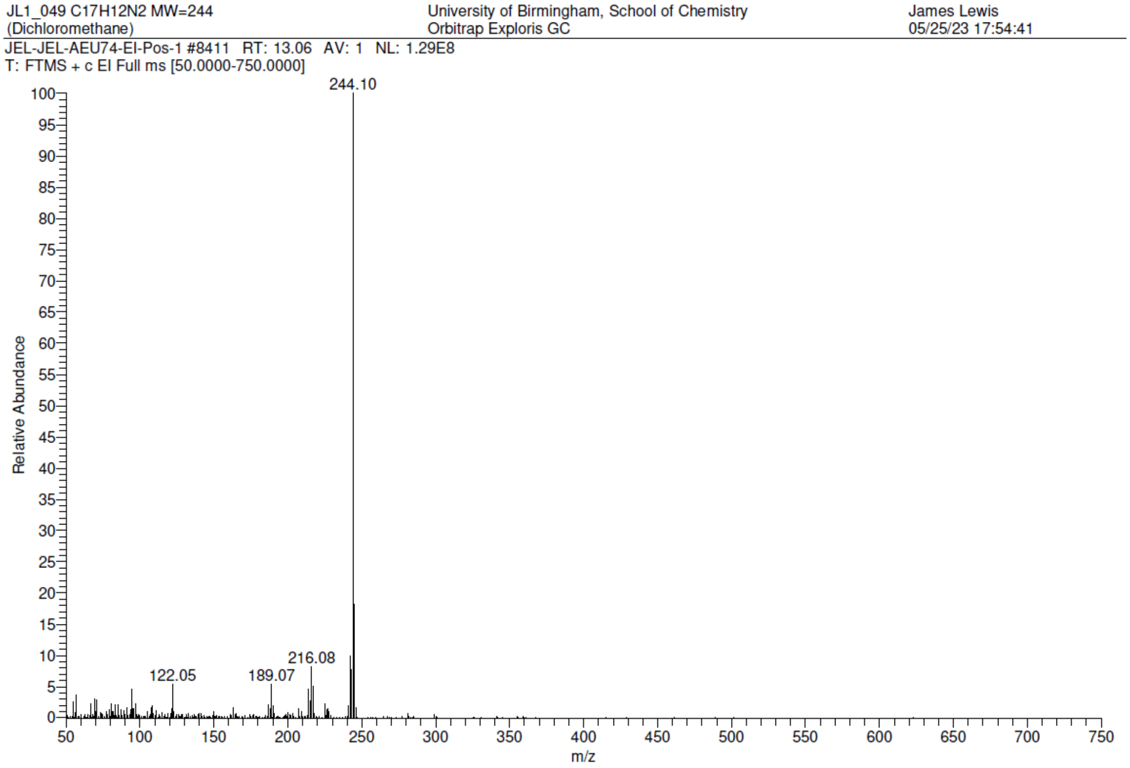


**Figure S89 EI-MS of S1.**

## S2.13 Synthesis of S2

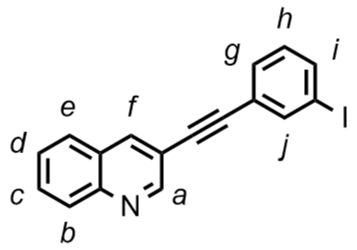

To a stirring suspension of ** (0.534 g, 2.19 mmol, 1 eq.) in MeCN (10 mL) at 0 °C in air was added TsOH·$H_2O$ (1.25 g, 6.56 mmol, 3 eq.) as a solid, portionwise. To this was added dropwise a solution of $NaNO_2$ (0.302 g, 4.57 mmol, 2 eq.) and KI (0.907 g, 5.46 mmol, 2.5 eq.) in $H_2O$ (1 mL). The reaction was stirred, allowing to warm to rt, for 17 h. $H_2O$ (50 mL), sat. aq. $NaHCO_3$ (50 mL) and 0.5 M $Na_2S_2O_{3(aq)}$ (25 mL) were added sequentially. The reaction mixture was extracted with EtOAc (3 × 25 mL). The combined organic phases were washed with brine (25 mL), dried ($MgSO_4$) and the solvent removed *in vacuo*. After purification by column chromatography on silica gel (5% v/v acetone in 1:1 pentane/$CH_2Cl_2$) the product was obtained as a yellow solid (0.530 g, 68%).

**$^1$H NMR** (500 MHz, $CDCl_3$) δ: 8.99 (d, *J* = 2.1 Hz, 1H, $H_a$), 8.31 (d, *J* = 1.8 Hz, 1H, $H_f$), 8.11 (d, *J* = 8.5 Hz, 1H, $H_e$), 7.96 (app. t, *J* = 1.5 Hz, 1H, $H_j$), 7.81 (d, *J* = 8.2 Hz, 1H, $H_b$), 7.76-7.71 (m, 2H, $H_d$, $H_i$), 7.59 (app. td, *J* = 7.5, 7.0, 1.1 Hz, 1H, $H_c$), 7.55 (app. dt, *J* = 7.7, 1.2 Hz, 1H, $H_g$), 7.13 (app. t, *J* = 7.8 Hz, 1H, $H_h$).

**$^{13}$C NMR** (126 MHz, $CDCl_3$) δ: 152.1 ($C_a$), 147.1, 140.4 ($C_j$), 138.6 ($C_f$), 137.9 ($C_i$), 131.0 ($C_g$), 130.5 ($C_d$), 130.1 ($C_h$), 129.6 ($C_e$), 127.8 ($C_b$), 127.6 ($C_c$), 127.4, 124.8, 117.1, 93.9, 90.9, 88.1.

**HR-EI-MS** *m/z* = 354.9853 $[M]^+$ calc. 354.9852.

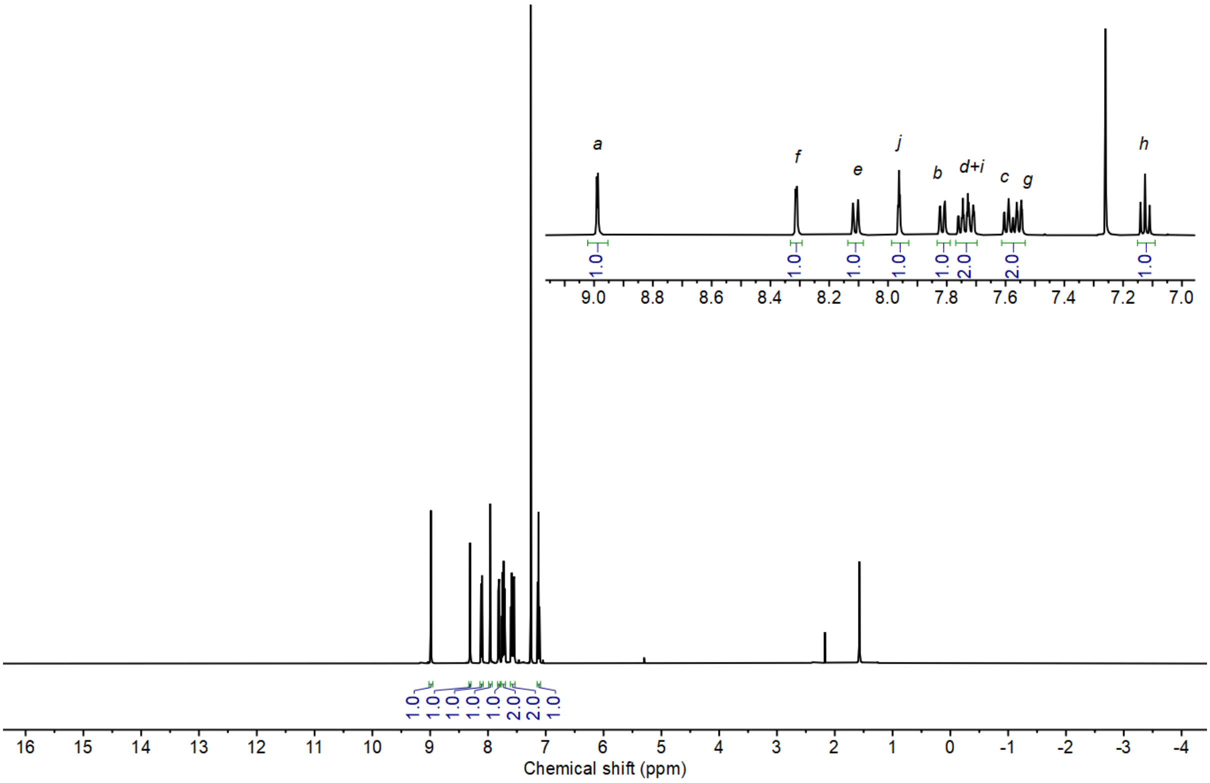

**Figure S90 $^1$H NMR (500 MHz, $CDCl_3$) of S2.**

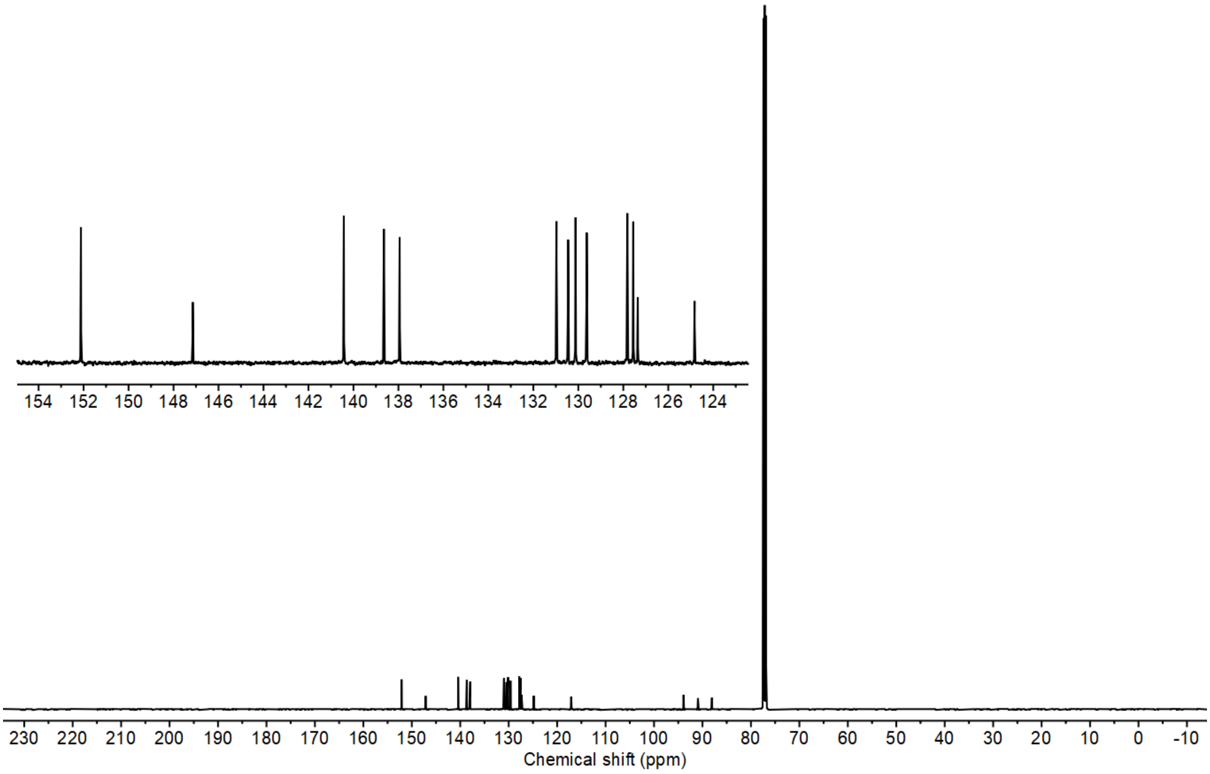

**Figure S91 $^{13}$C NMR (126 MHz, $CDCl_3$) of S2.**

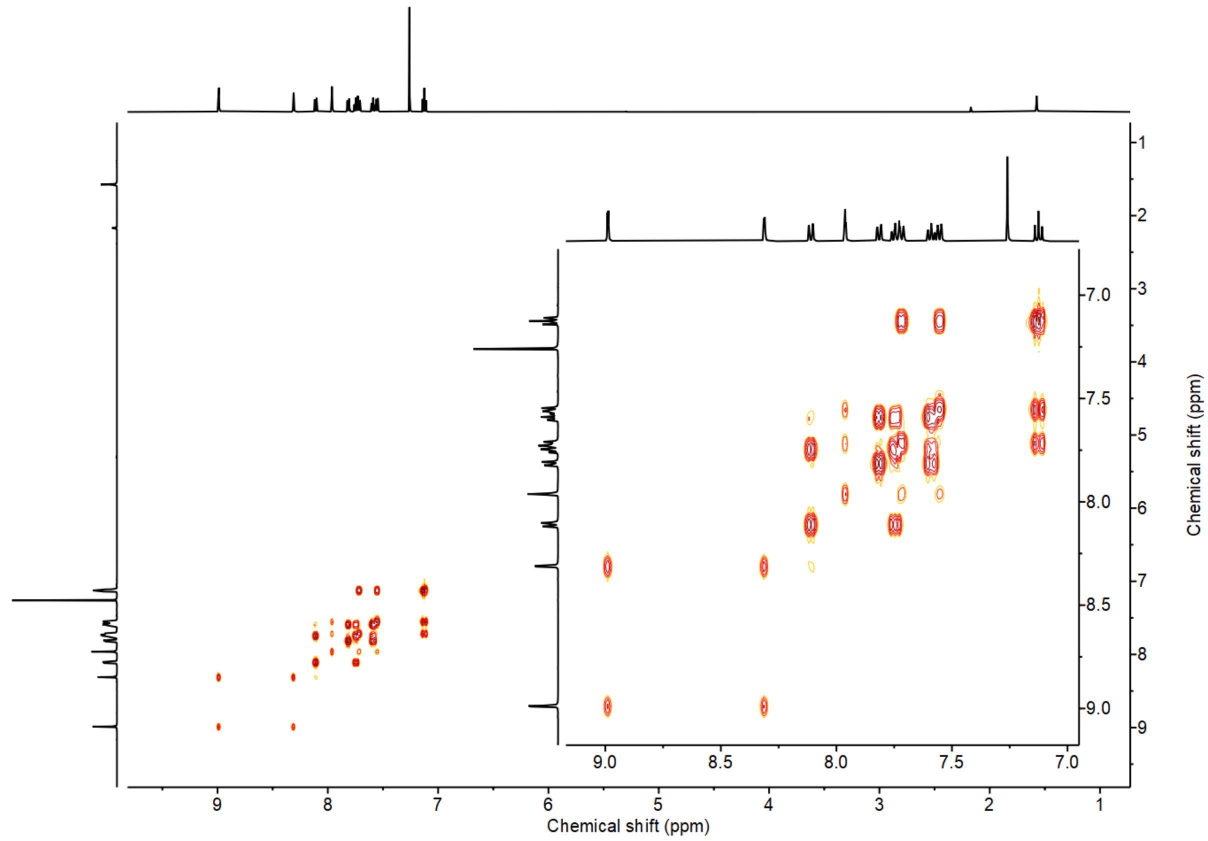


**Figure S92 COSY NMR ($CDCl_3$) of S2.**

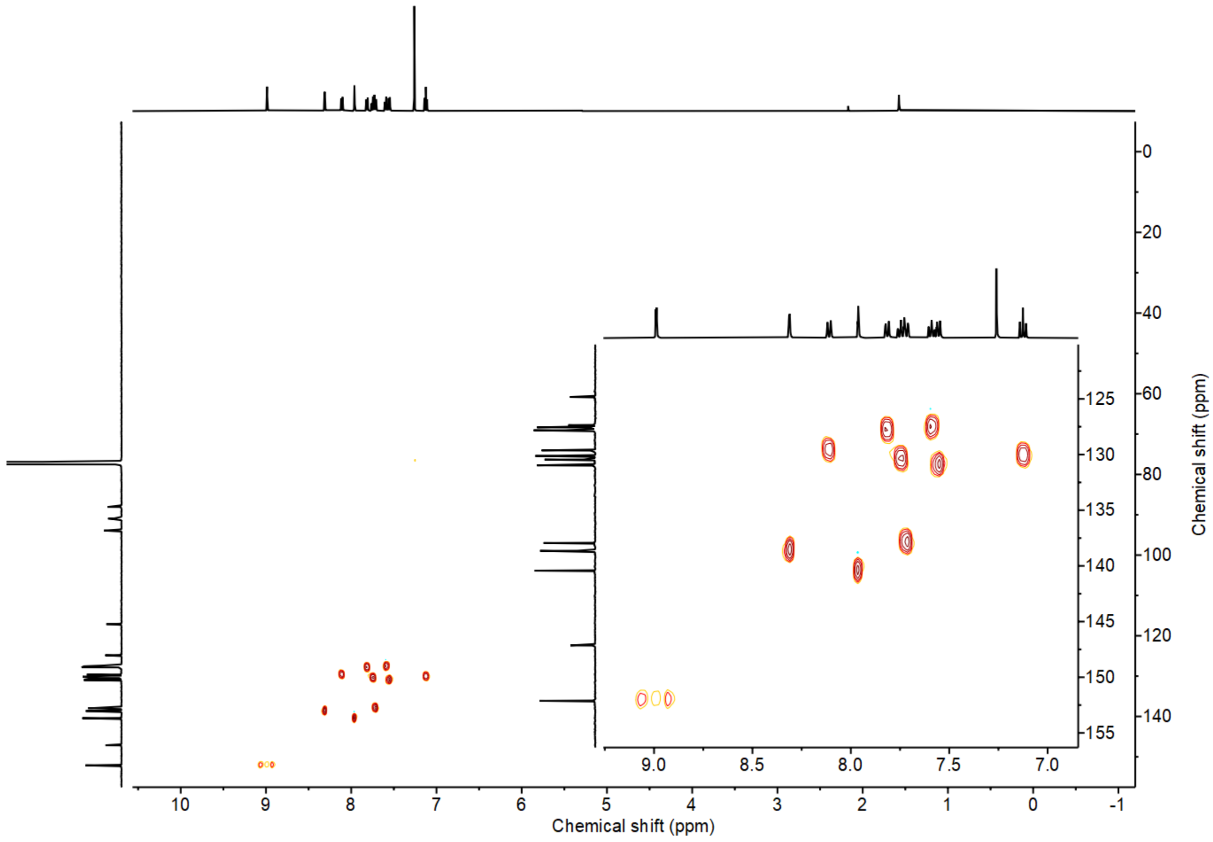


**Figure S93 HSQC NMR ($CDCl_3$) of S2.**

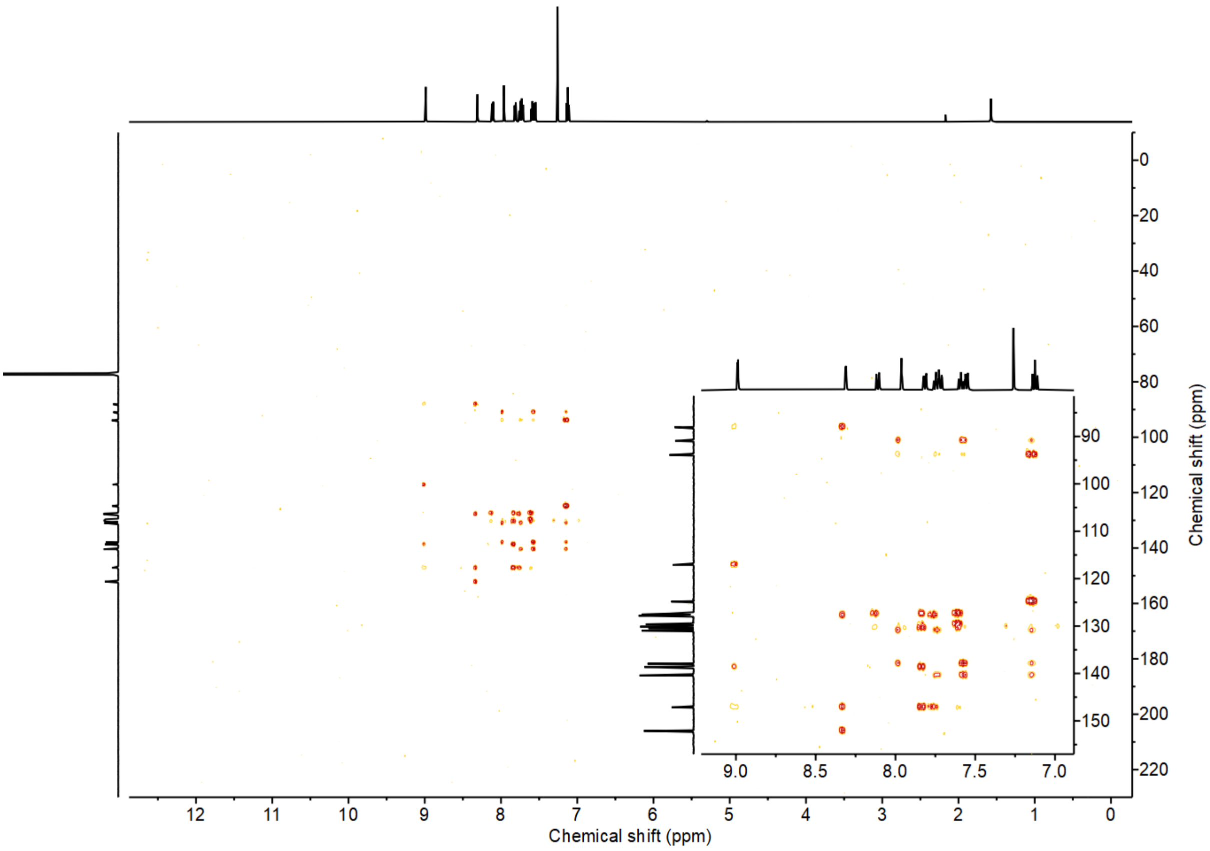


**Figure S94 HMBC NMR ($CDCl_3$) of S2.**

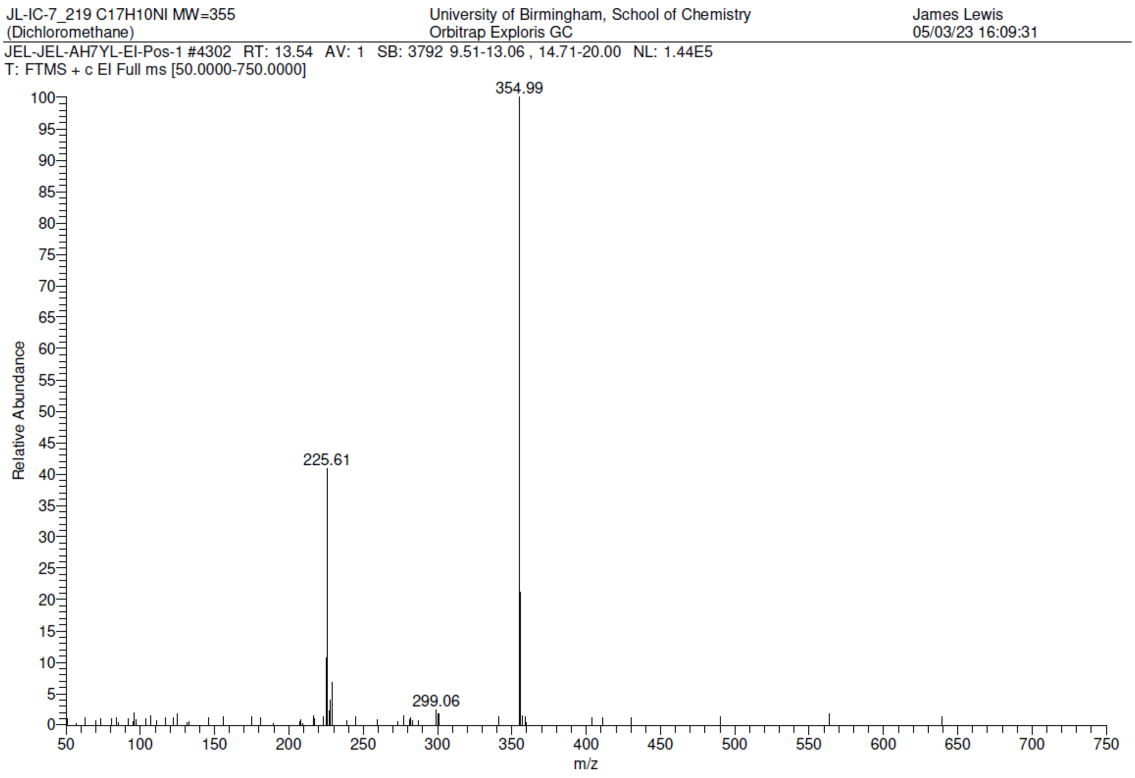


**Figure S95 EI-MS of S2.**

## S2.14 Synthesis of L2$^{Q}$

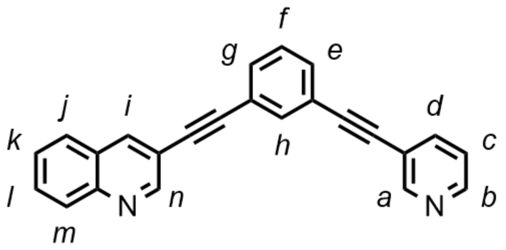


$^{i}Pr_2NH$ (2.5 mL) was added to a solution of **S2** (100 mg, 0.28 mmol, 1 eq.), 3-ethynylpyridine (39.2 mg, 0.38 mmol, 1.4 eq.), $Pd(PPh_3)_4$ (17.4 mg, 15 µmol, 5 mol%) and CuI (2.9 mg, 15 µmol, 5 mol%) in dioxane (2.5 mL) at rt and the reaction mixture stirred for 18 h. 0.1 M $EDTA_{(aq)}$ (10 mL) was added and the aqueous phase extracted with $CH_2Cl_2$ (3 × 10 mL). The combined organic phases were dried ($MgSO_4$) and the solvent removed *in vacuo*. After purification by column chromatography on silica gel (step gradient 0% to 7.5% v/v acetone in $CH_2Cl_2$ in 2.5% increments) the product was obtained as a yellow solid (83.3 mg, 84%).

**$^{1}$H NMR** (500 MHz, $d_6$-acetone) δ: 9.02 (d, *J* = 2.1 Hz, 1H, $H_n$), 8.79 (d, *J* = 1.3 Hz, 1H, $H_a$), 8.60 (dd, *J* = 4.8, 1.7 Hz, 1H, $H_b$), 8.55 (dd, *J* = 2.2, 0.8 Hz, 1H, $H_i$), 8.08 (d, *J* = 8.4 Hz, 1H, $H_m$), 8.01 (m, 1H, $H_j$), 7.97 (app. dt, *J* = 7.8, 2.0 Hz, 1H, $H_d$), 7.85-7.80 (m, 2H, $H_h$, $H_l$), 7.71-7.65 (m, 3H, $H_e$, $H_g$, $H_k$), 7.55 (app. t, *J* = 7.8 Hz, 1H, $H_f$), 7.46 (ddd, *J* = 7.8, 4.8, 0.9 Hz, 1H, $H_c$).

**$^{13}$C NMR** (126 MHz, $d_6$-acetone) δ: 152.9 ($C_a$), 152.6 ($C_n$), 150.1 ($C_b$), 148.1, 139.2 ($C_d$, $C_i$), 135.3 ($C_h$), 132.8 ($C_e$/$C_g$), 132.8 ($C_e$/$C_g$), 131.3 ($C_l$), 130.2 ($C_f$/$C_m$), 130.2 ($C_f$/$C_m$), 128.9 ($C_j$), 128.4 ($C_k$), 128.1, 124.3, 124.2, 124.1, 120.7, 117.7, 91.9, 91.9, 88.3, 87.7.

**HR-ESI-MS** *m/z* = 331.1237 $[M+H]^+$ calc. 331.1235.

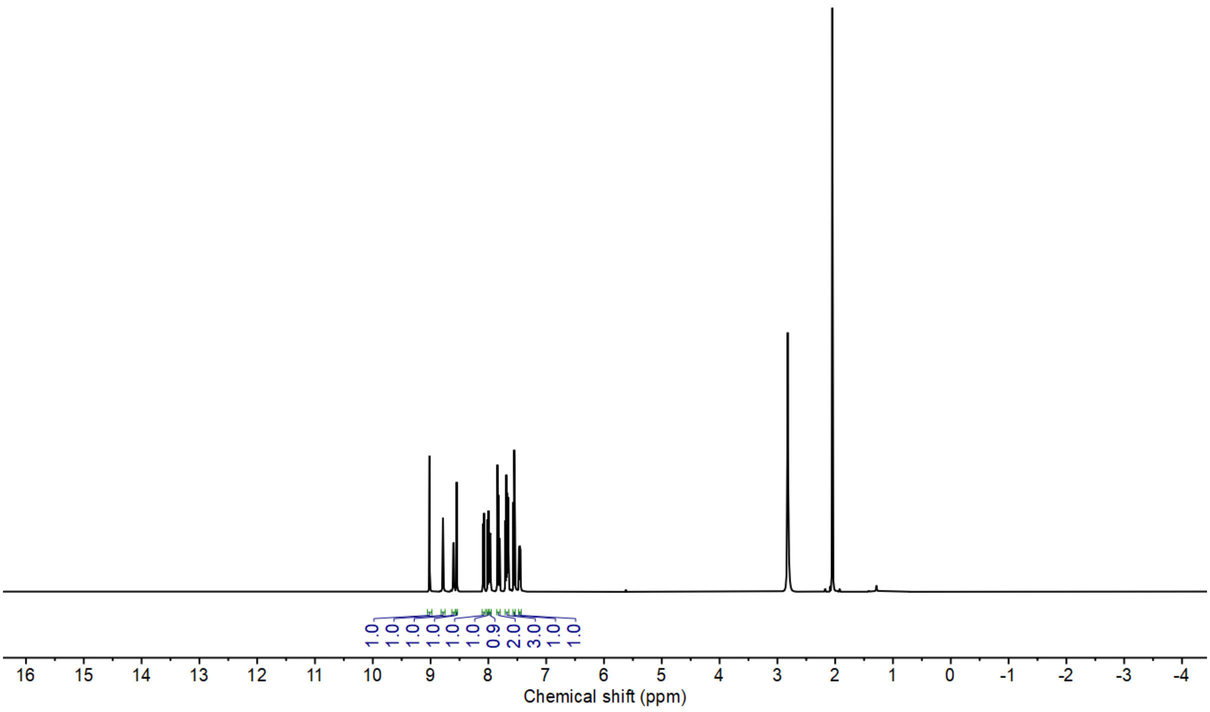


**Figure S96** **$^{1}$H NMR (500 MHz, $d_6$-acetone) of L2$^{Q}$.**

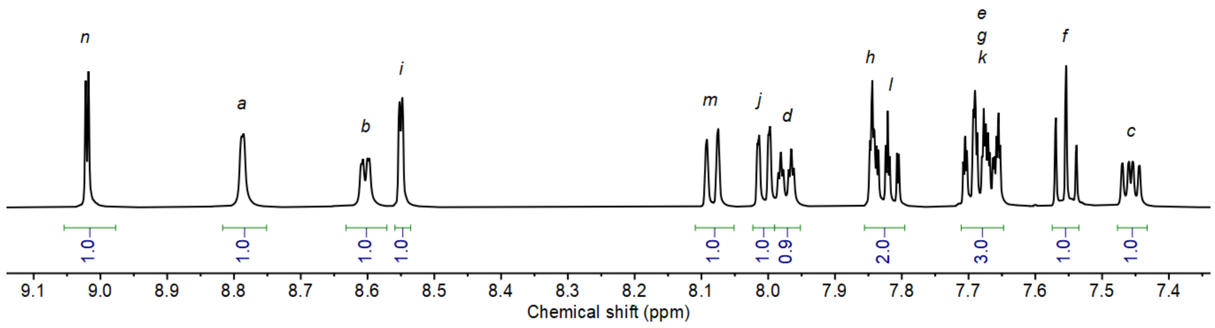


**Figure S97 Partial $^{1}H$ NMR (500 MHz, $d_6$-acetone) of L2$^{Q}$.**

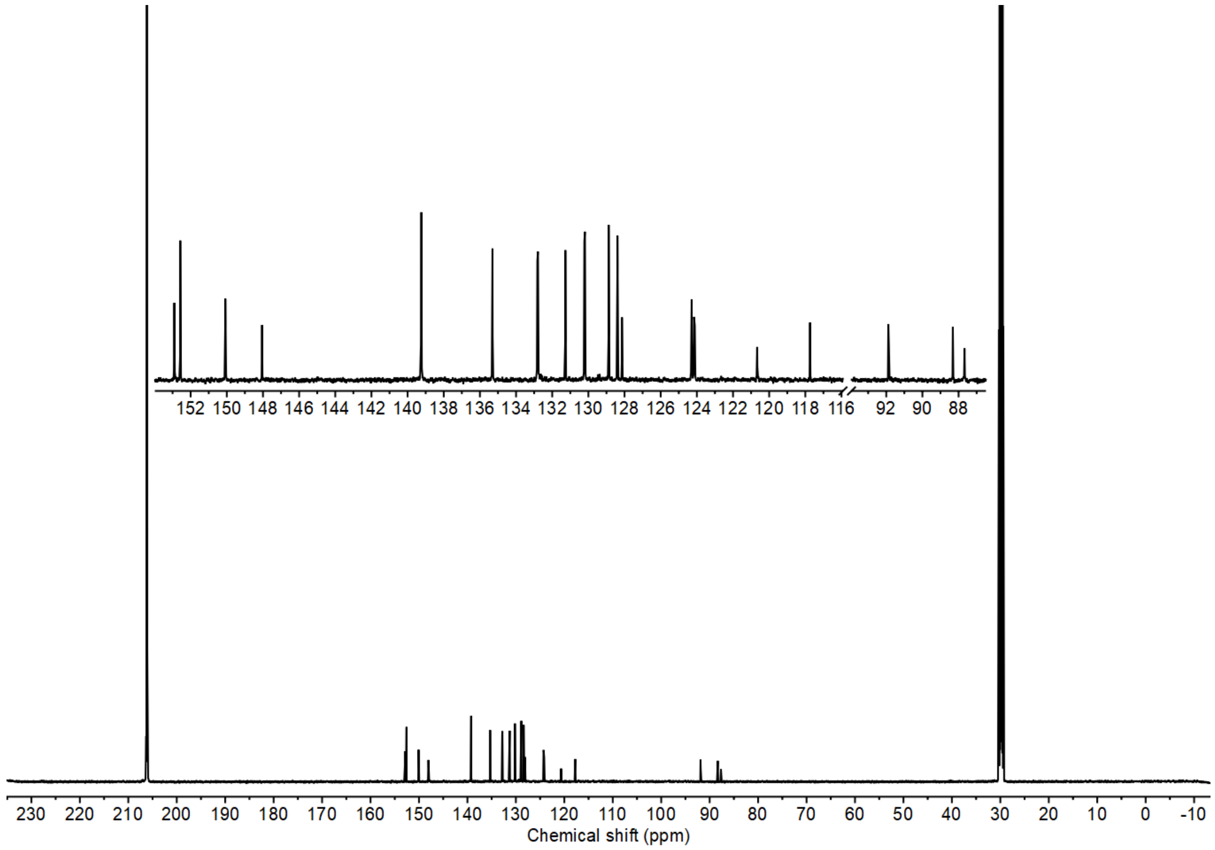


**Figure S98 $^{13}C$ NMR (126 MHz, $d_6$-acetone) of L2$^{Q}$.**

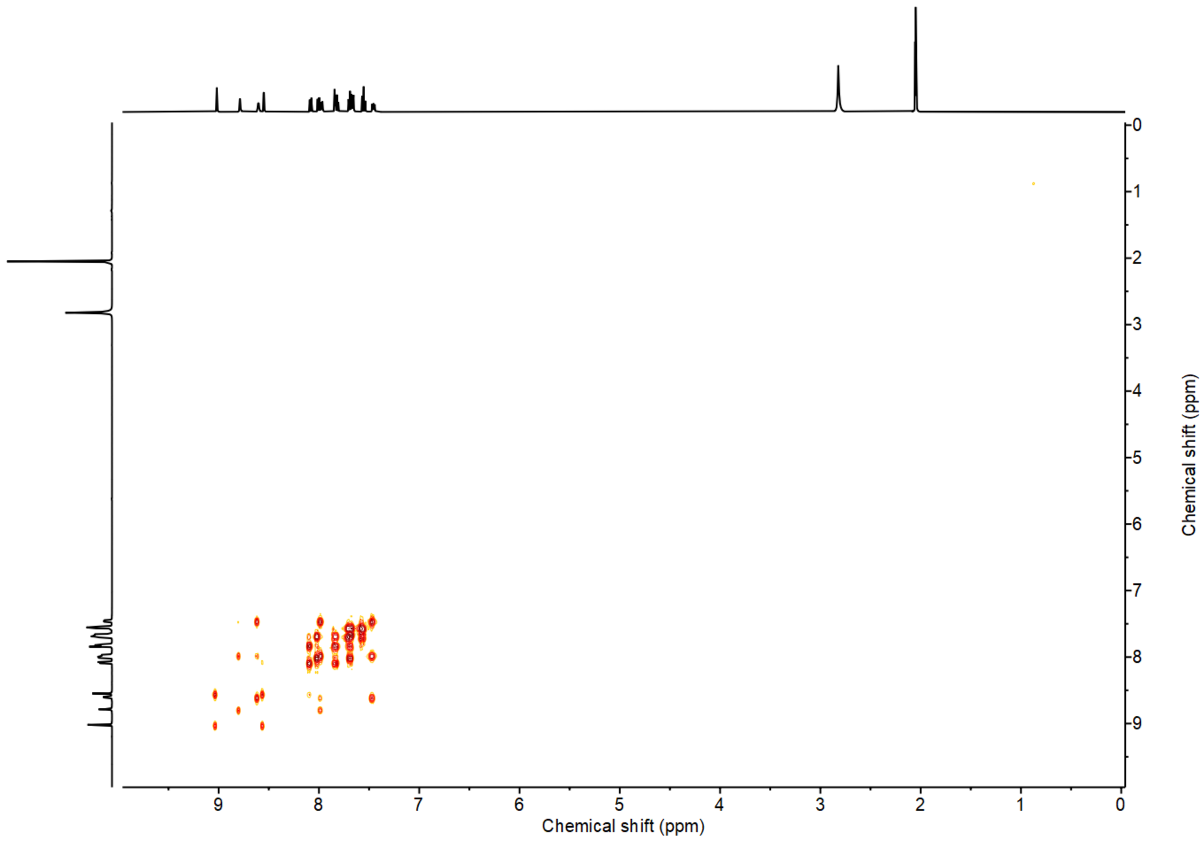


**Figure S99 COSY ($d_6$-DMSO) of L2$^{Q}$.**

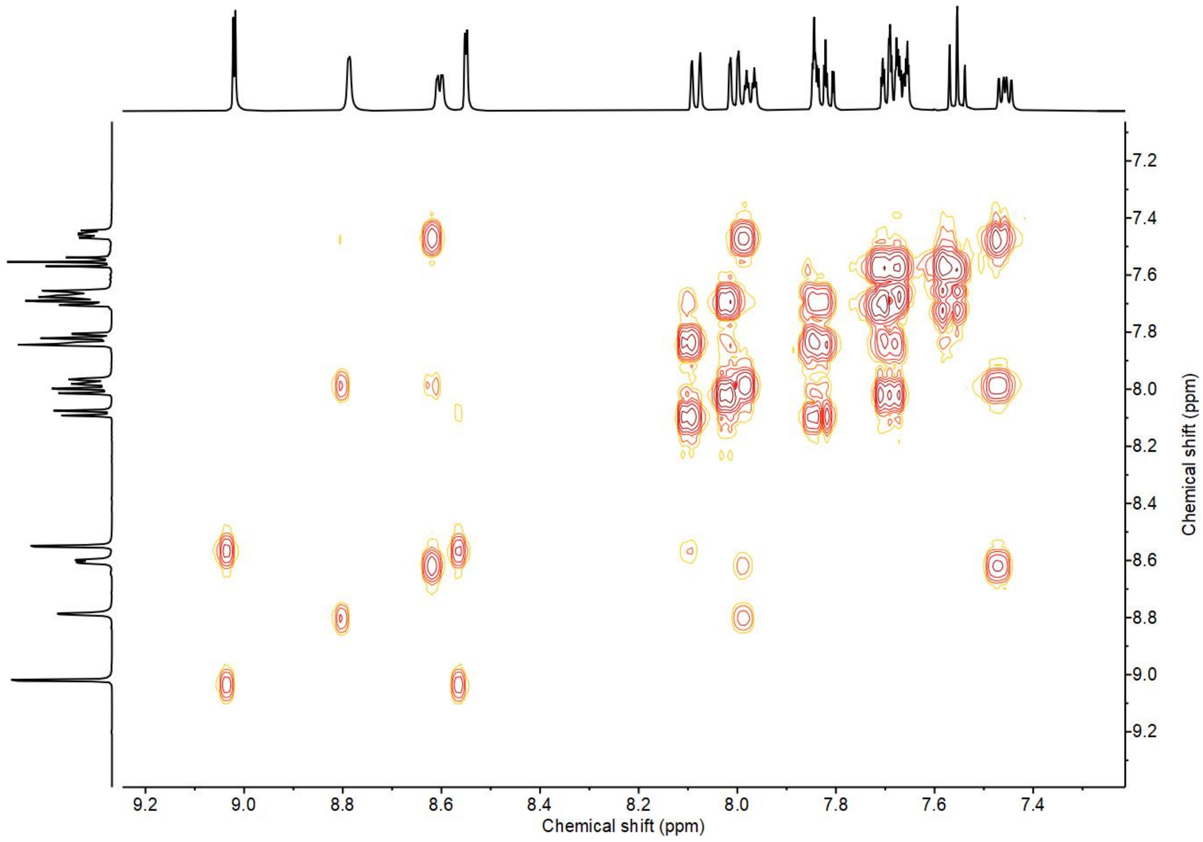


**Figure S100 Partial COSY ($d_6$-DMSO) of L2$^{Q}$.**

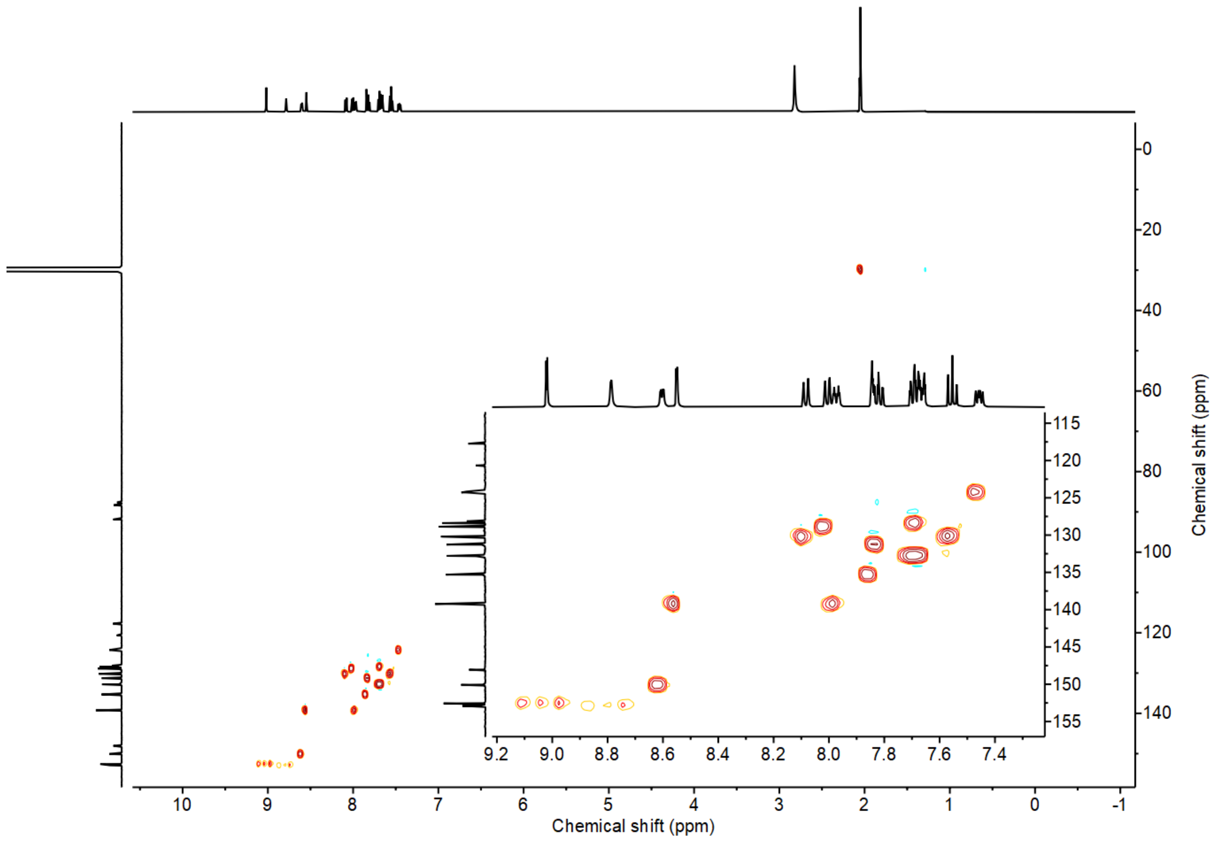


**Figure S101 HSQC ($d_6$-DMSO) of L2$^{Q}$.**

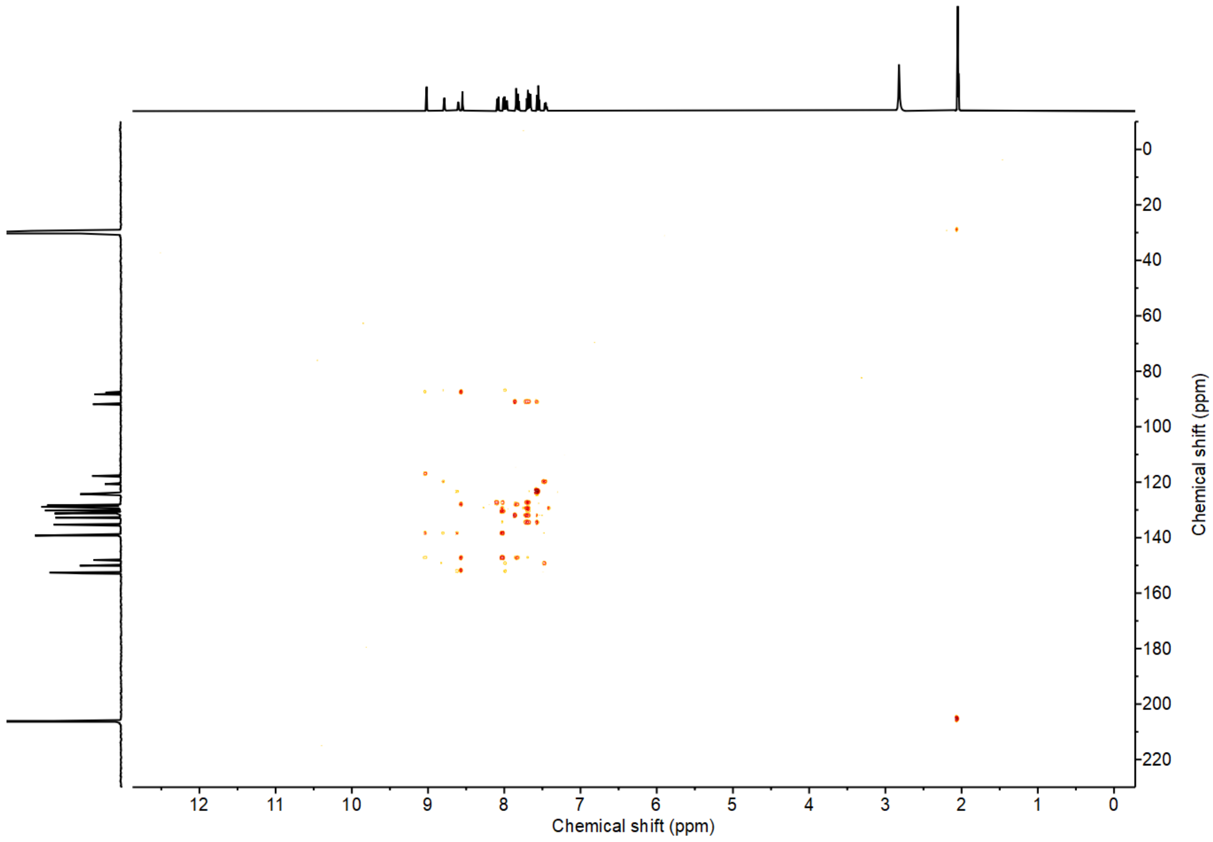


**Figure S102 HMBC ($d_6$-DMSO) of L2$^{Q}$.**

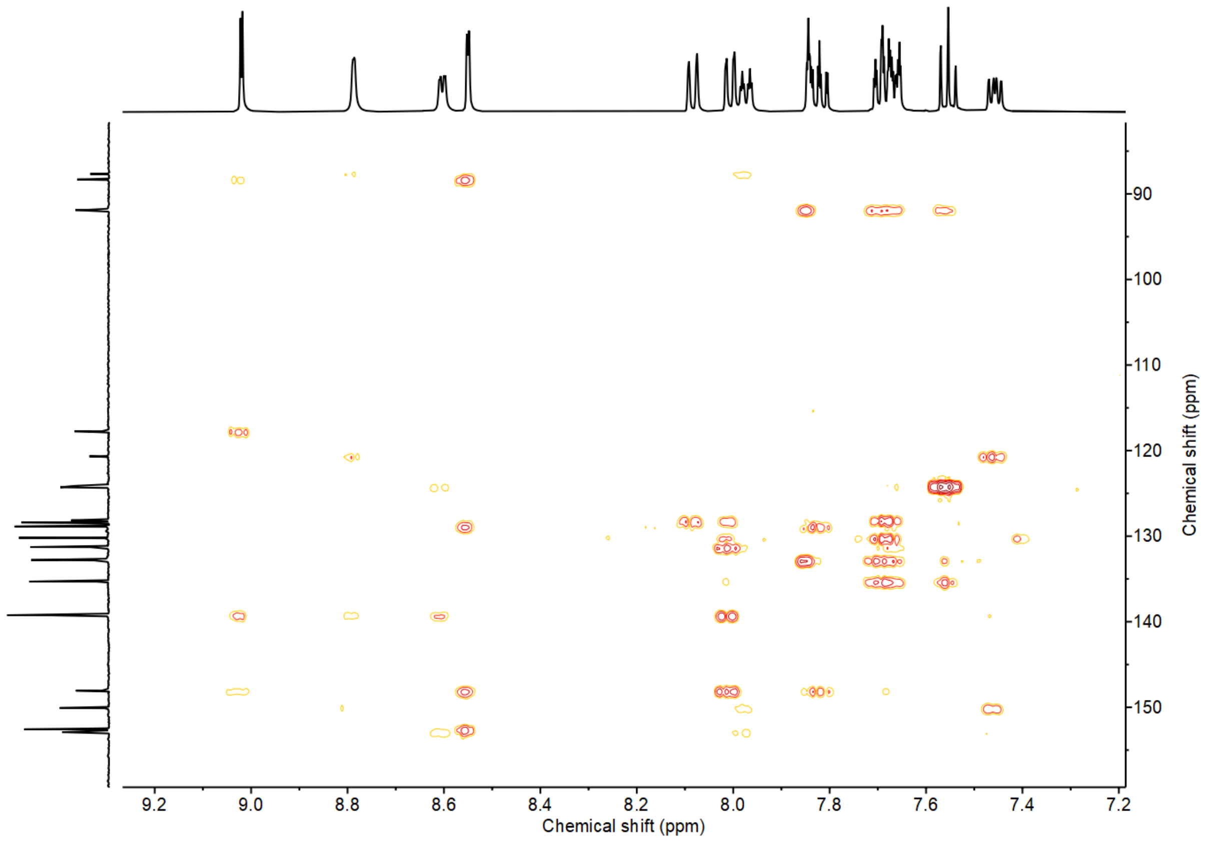


**Figure S103 Partial HMBC ($d_6$-DMSO) of L2[Q].**

## S2.15 Synthesis of *cis*-$C2^Q$

**$L2^Q$** (13.2 mg, 40 µmol, 1 eq.) and $[Pd(CH_3CN)_4](BF_4)_2$ )(8.9 mg, 20 µmol, 0.5 eq.) were sonicated in $d_6$-DMSO (1.0 mL) until a homogenous solution was obtained. After standing at 50 °C for 24 h, no further changes were observed by $^1$H NMR.

Only peaks for major species are reported.

**$^1$H NMR** (500 MHz, $d_6$-DMSO) δ: 10.60 (d, $J$ = 8.8 Hz, 4H, $H_m$), 10.42 (s, 4H, $H_n$), 9.88 (s, 4H, $H_a$), 9.70 (dd, $J$ = 6.0, 1.4 Hz, 4H, $H_b$), 9.00 (d, $J$ = 1.8 Hz, 4H, $H_i$), 8.33 (ddd, $J$ = 8.6, 7.0, 1.4 Hz, 4H, $H_l$), 8.25 (app. dt, $J$ = 8.0, 1.6 Hz, 4H, $H_d$), 8.11 (d, $J$ = 7.5 Hz, 4H, $H_j$), 7.94 (s, 4H, $H_h$), 7.90 (app. t, $J$ = 7.6 Hz, 4H, $H_k$), 7.81-7.78 (m, 8H, $H_c$, $H_e$/$H_g$), 7.74 (app. dt, $J$ = 7.8, 1.3 Hz, 4H, $H_e$/$H_g$), 7.61 (app. t, $J$ = 7.7 Hz, 4H, $H_f$).

**$^{13}$C NMR** (126 MHz, $d_6$-DMSO) δ: 155.5 ($C_n$), 152.9 ($C_a$), 151.3 ($C_b$), 144.6 ($C_i$), 144.1 ($C_m$), 143.3 ($C_d$), 134.1 ($C_e$/$C_g$), 133.8 ($C_e$/$C_g$), 133.7 ($C_l$), 132.3 ($C_h$), 130.2 ($C_f$), 129.7 ($C_j$), 129.7 ($C_k$), 128.5, 127.1 ($C_c$), 122.4, 122.1, 121.9, 118.1, 94.0, 93.1, 86.5, 85.3 (2 quaternary signals missing).

**ESI-MS** $m/z$ = 1659.28 $\{[Pd_2(C_{24}H_{14}N_2)_4](BF_4)F_2\}^+$ calc. 1659.27; 1727.28 $\{[Pd_2(C_{24}H_{14}N_2)_4](BF_4)_2F\}^+$ calc. 1727.28; 1794.28 $\{[Pd_2(C_{24}H_{14}N_2)_4](BF_4)_3\}^+$ calc. 1794.28.

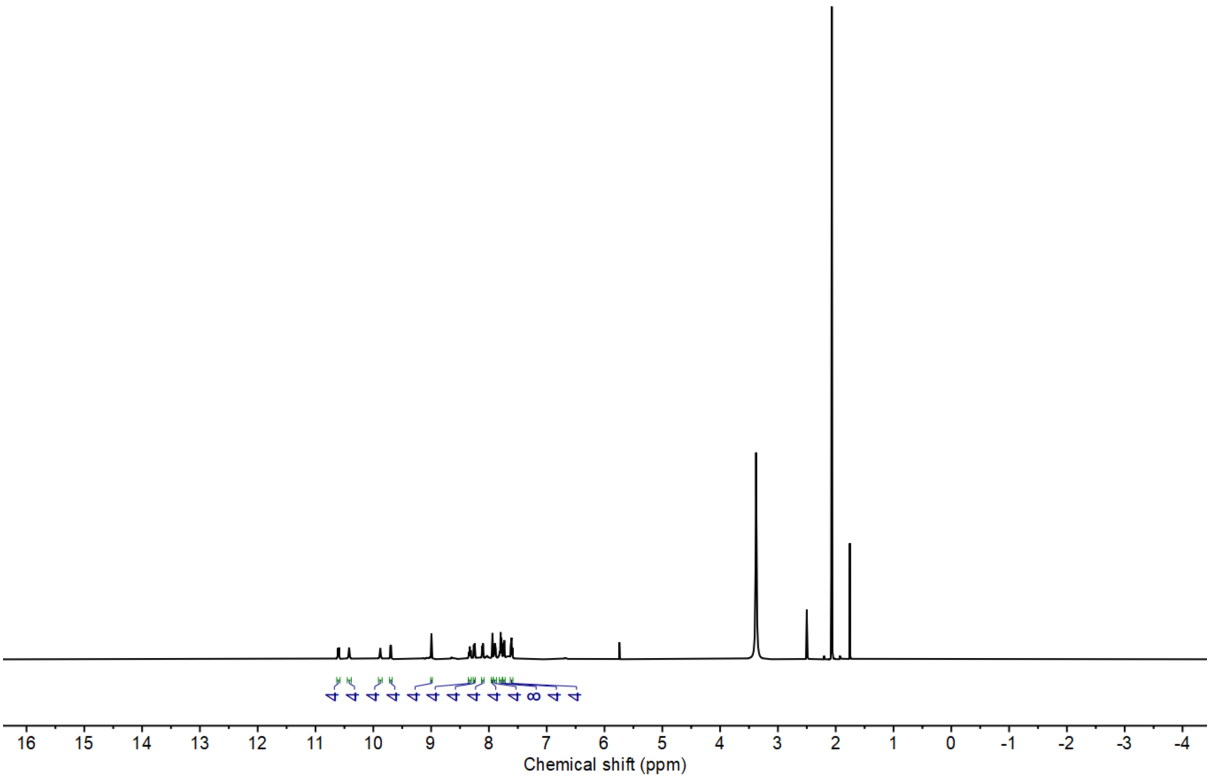


**Figure S104 $^1$H NMR (500 MHz, $d_6$-DMSO) of $C2^Q$.**

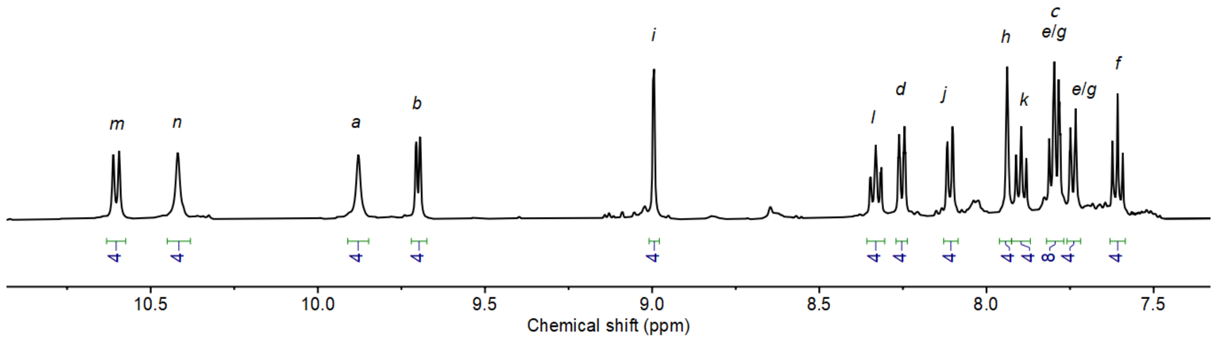


**Figure S105 Partial $^{1}$H NMR (500 MHz, $d_6$-DMSO) of C2$^{Q}$.**

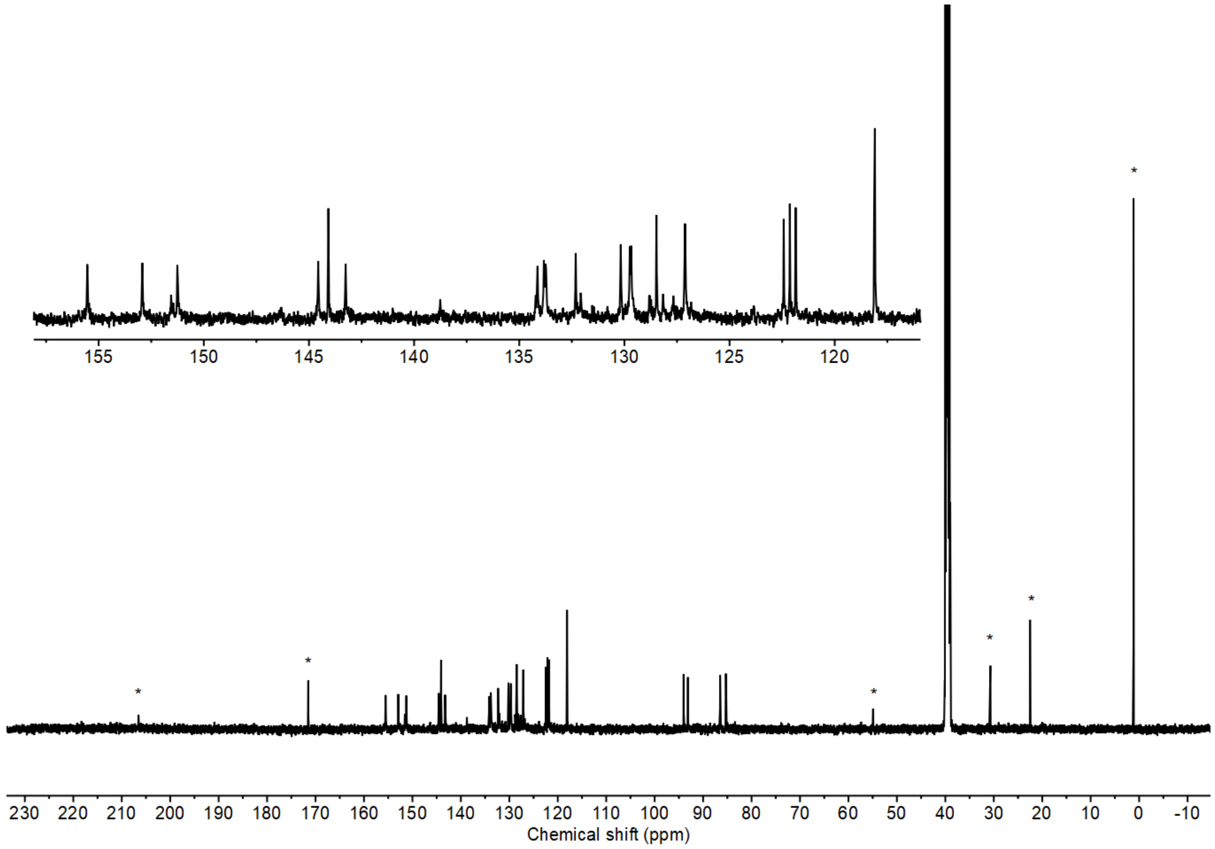


**Figure S106 $^{13}$C NMR (126 MHz, $d_6$-DMSO) of C2$^{Q}$ (*$^{-}$OAc impurity from Pd(II) salt and solvents).**

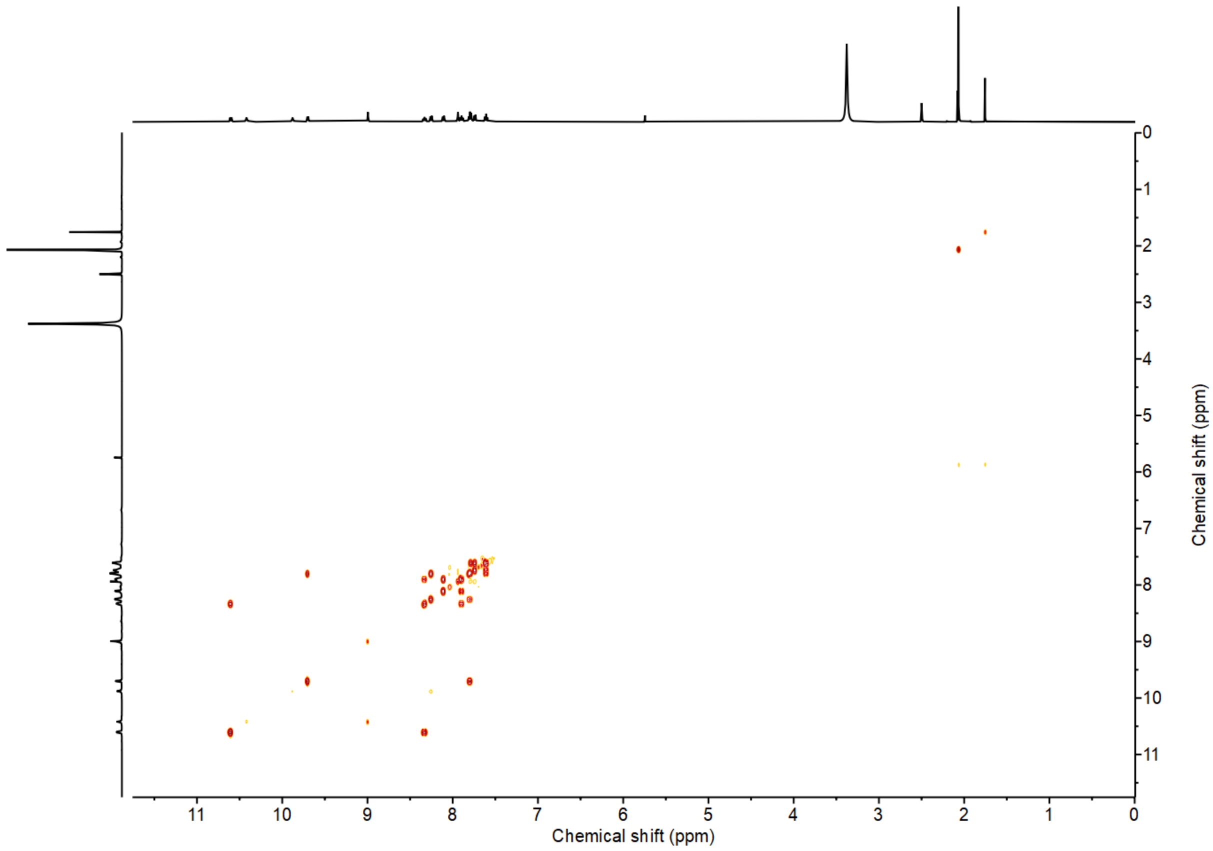


**Figure S107 COSY ($d_6$-DMSO) of C2[Q].**

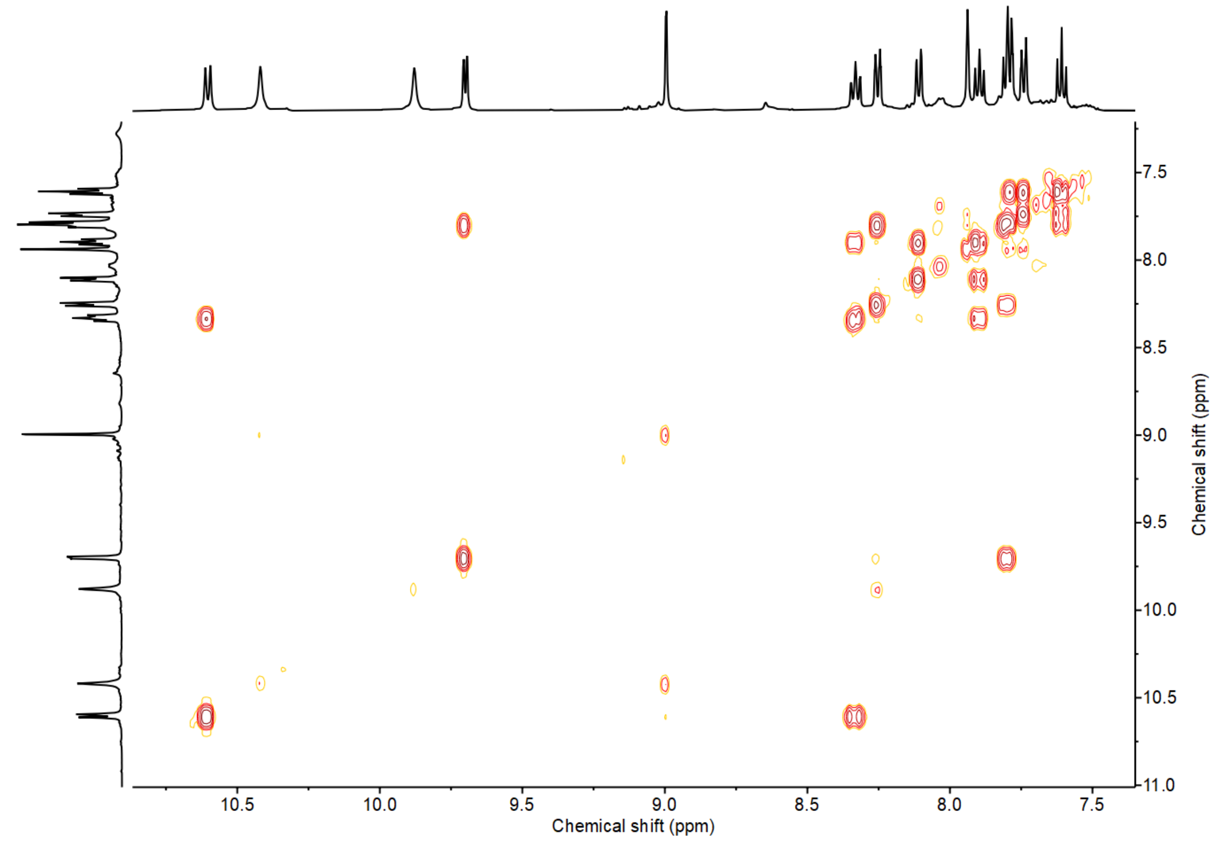


**Figure S108 Partial COSY ($d_6$-DMSO) of C2[Q].**

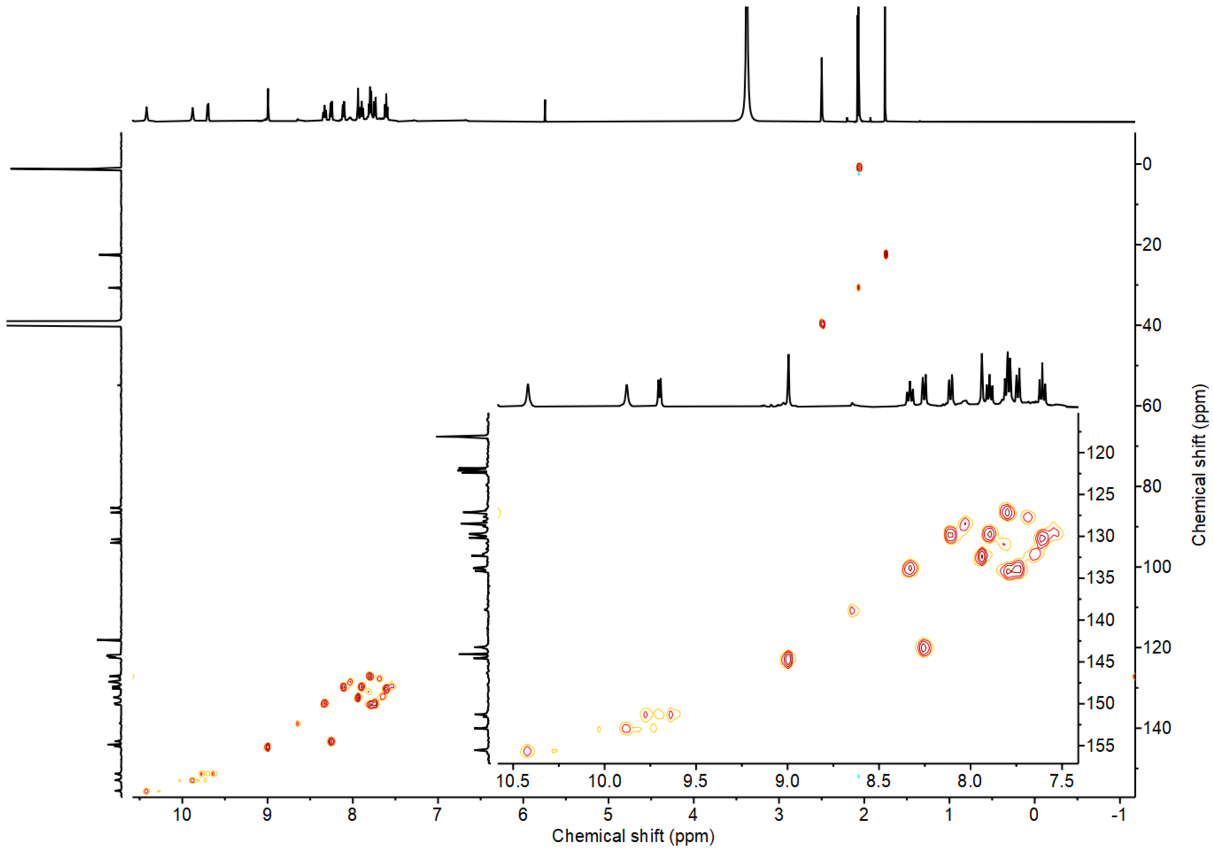


**Figure S109 HSQC ($d_6$-DMSO) of C2[Q].**

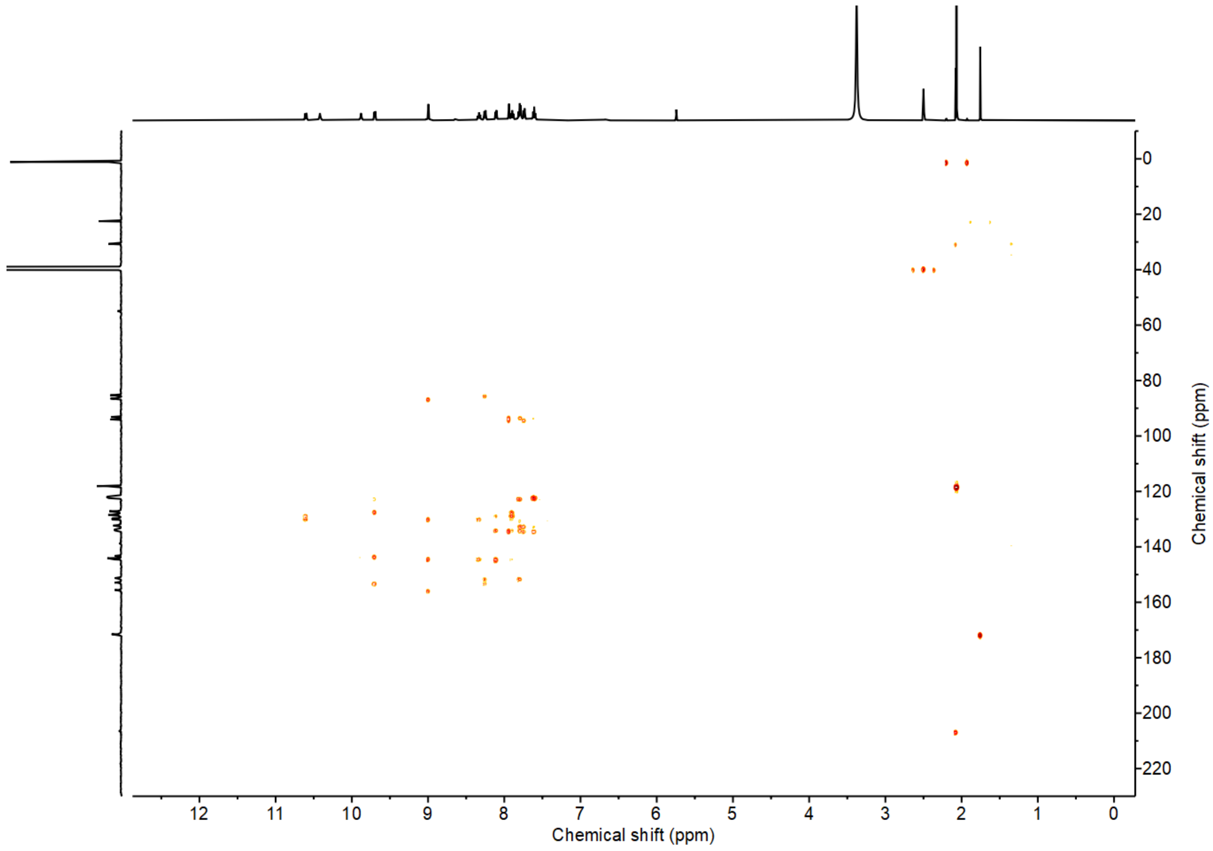


**Figure S110 HMBC ($d_6$-DMSO) of C2[Q].**

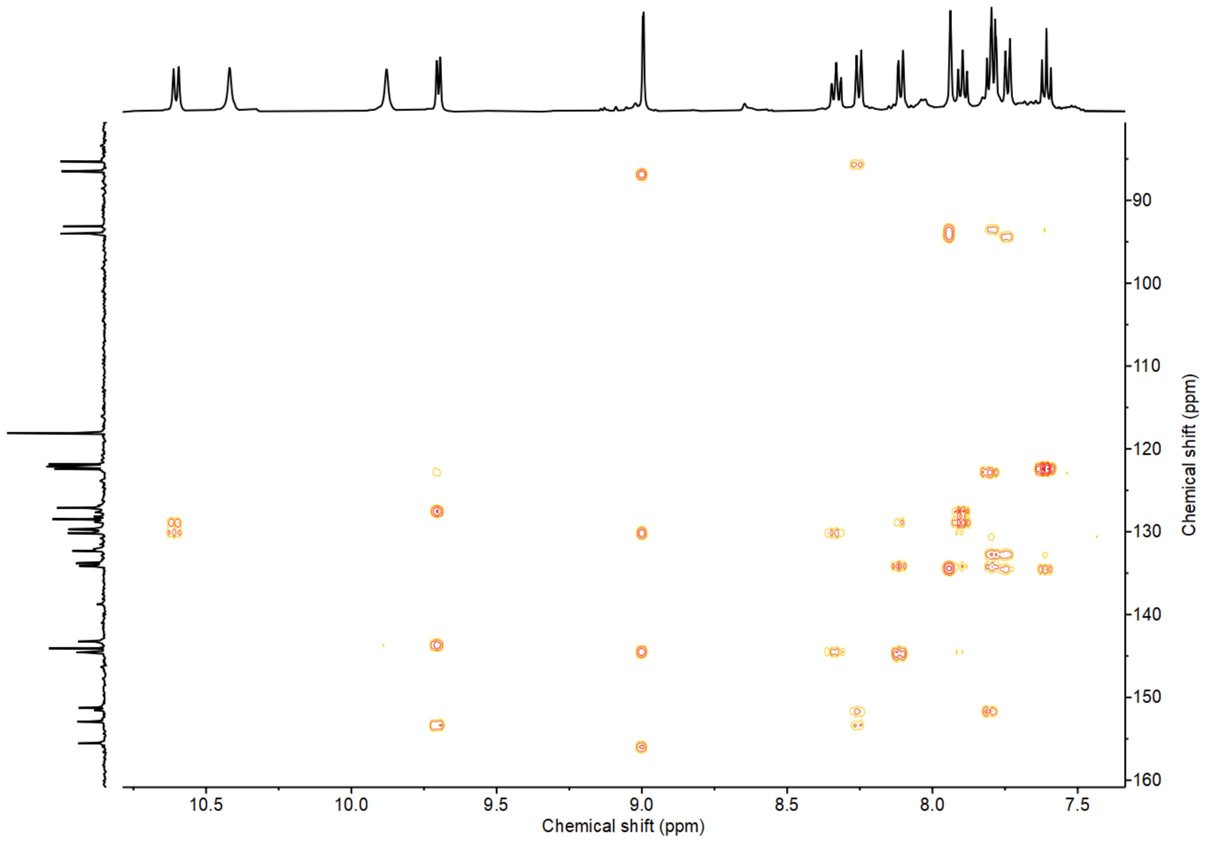


**Figure S111 Partial HMBC (*d*$_6$-DMSO) of C2$^Q$.**

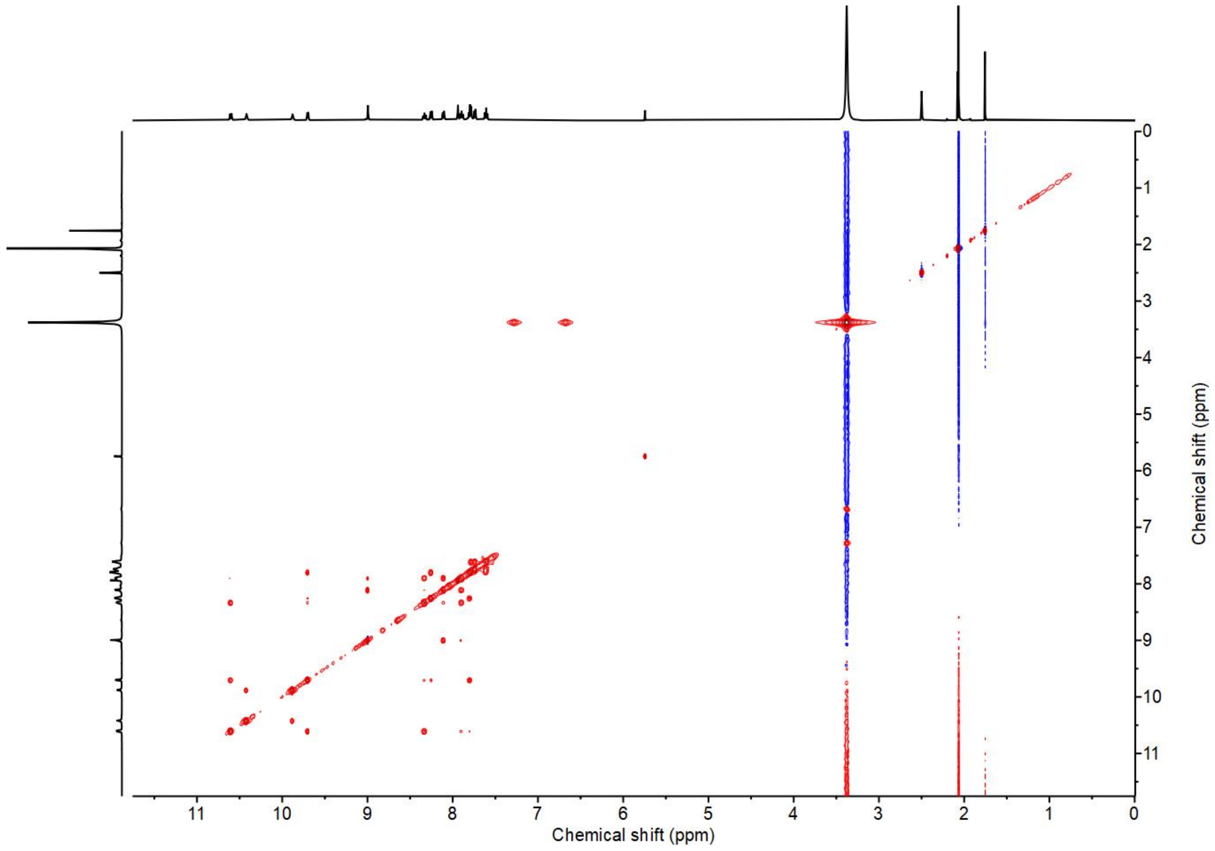


**Figure S112 NOESY (*d*$_6$-DMSO) of C2$^Q$.**

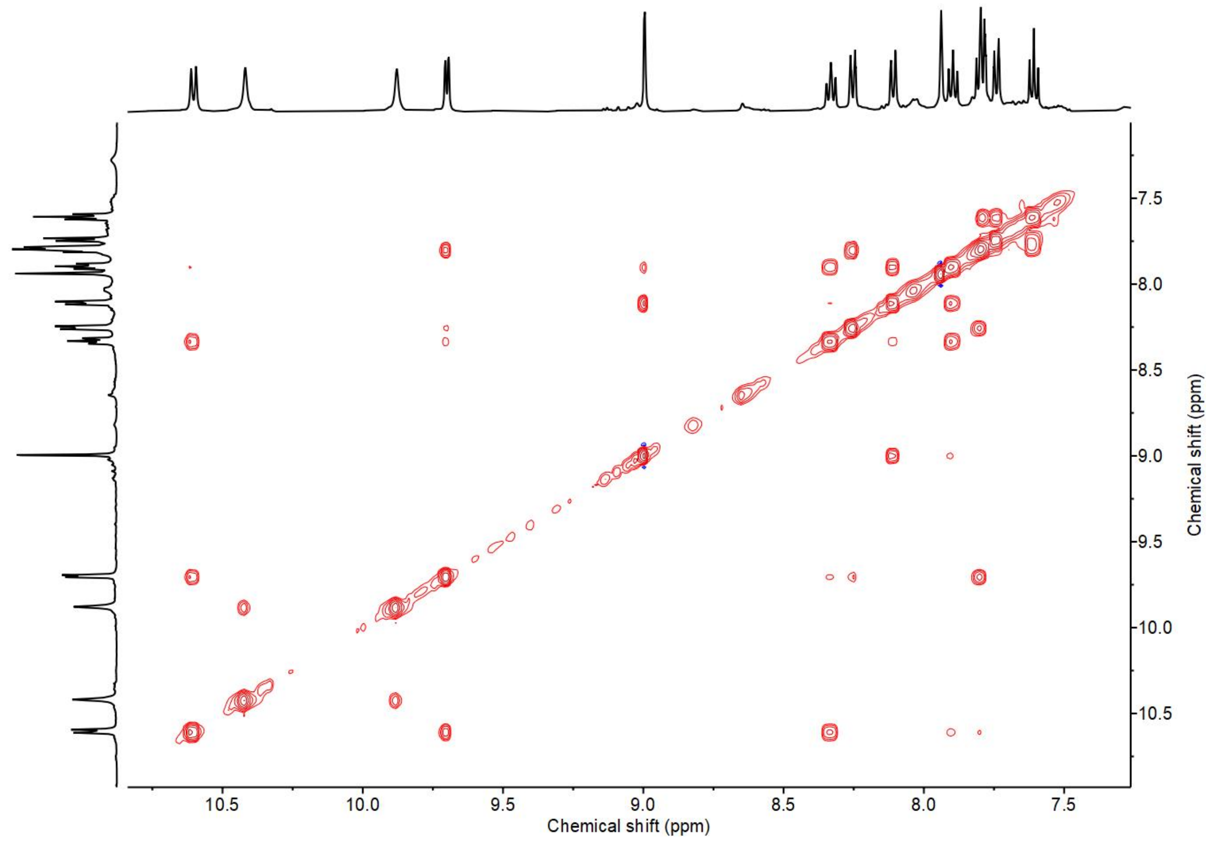


**Figure S113 Partial NOESY ($d_6$-DMSO) of C2$^{Q}$.**

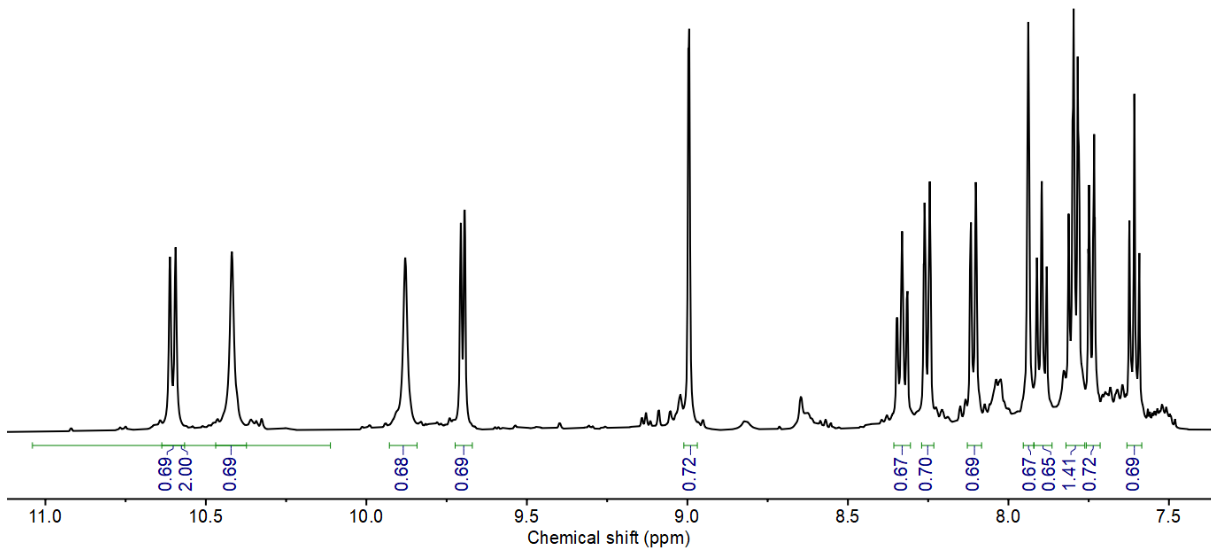


**Figure S114 Partial $^{1}$H NMR ($d_6$-DMSO, 500 MHz) showing estimation of isomer composition through relative integration compared to region containing signals for H$_m$ and H$_n$.**

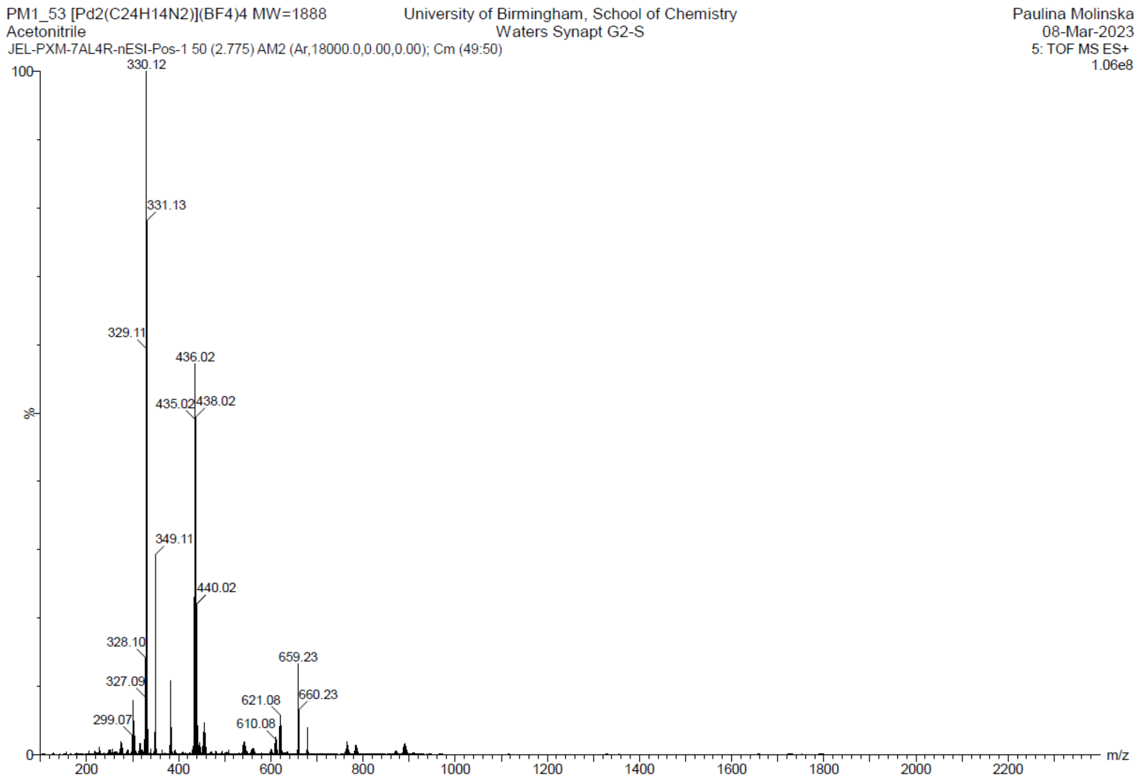


**Figure S115** ESI-MS of **C2^Q^.**

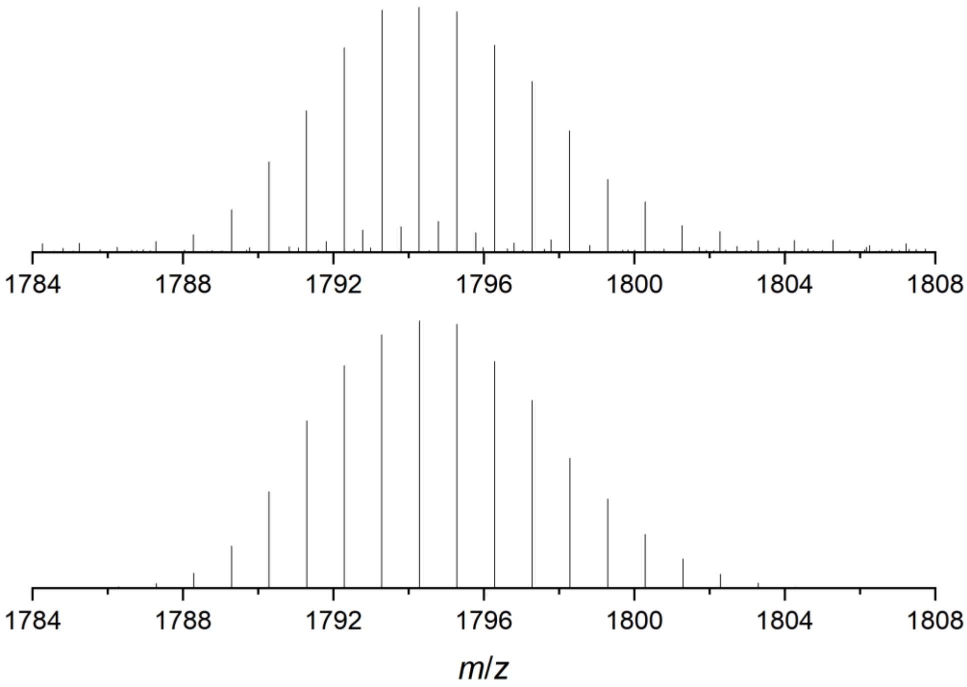


**Figure S116** Observed (top) and calculated (bottom) isotopic patterns for $\{[Pd_2(\mathbf{L2^{Q}})_4](BF_4)_3\}^{+}$.

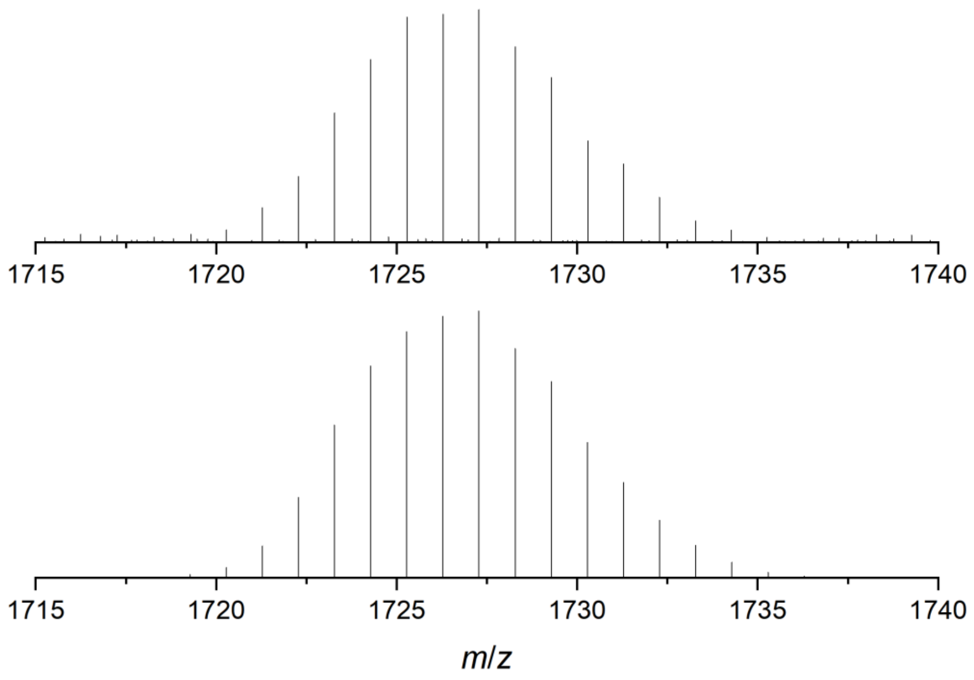


**Figure S117** Observed (top) and calculated (bottom) isotopic patterns for {[$Pd_2$(**L2<sup>Q</sup>**)$_4$]($BF_4$)$_2$F}$^+$.

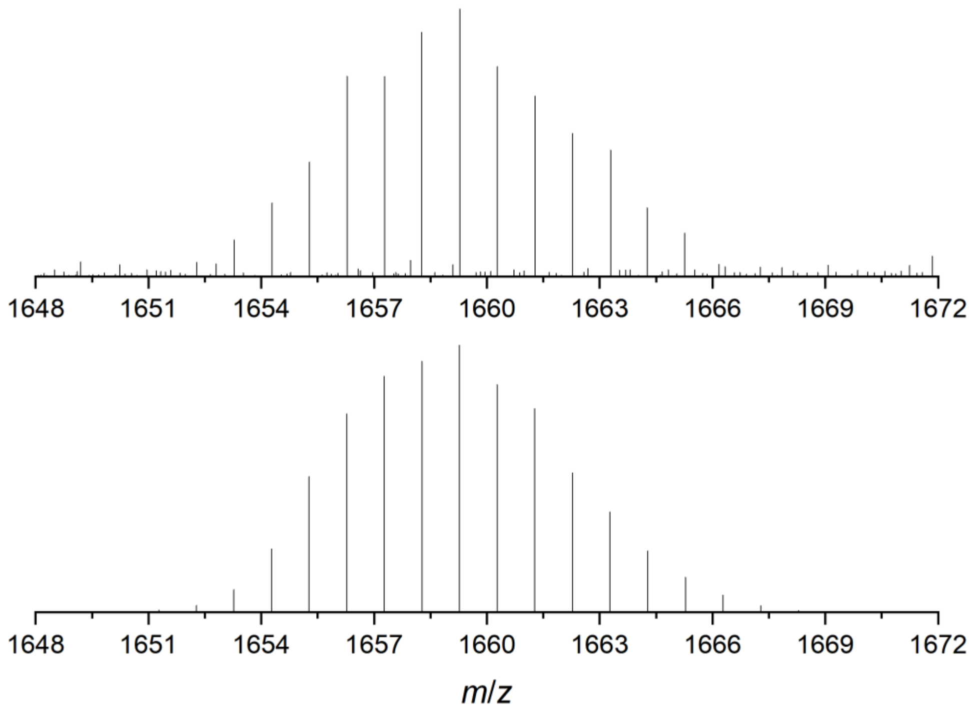


**Figure S118** Observed (top) and calculated (bottom) isotopic patterns for {[$Pd_2$(**L2<sup>Q</sup>**)$_4$]($BF_4$)$F_2$}$^+$.

## S2.16 Synthesis of L3$^{Q}$

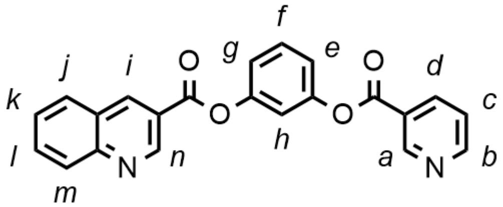


To a stirring suspension of quinoline-3-carboxylic acid (0.346 g, 2.0 mmol, 1.0 eq.), nicotinic acid (0.246 g, 2.0 mmol, 1.0 eq.) and DMAP (0.024 g, 0.20 mmol, 0.1 eq.) in $CHCl_3$ (20 mL) at 0 °C was added EDCI (0.844 g, 4.4 mmol, 2.2 eq.) portionwise as a solid. After 30 minutes, resorcinol (0.220 g, 2.0 mmol, 1.0 eq.) was added as a solid. The reaction was stirred, allowing to warm to rt, for 24 h. The reaction mixture was washed with sat. aq. $NaHCO_3$ (2 × 20 mL), dried ($MgSO_4$) and the solvent removed *in vacuo*. After purification by column chromatography (step gradient 15 to 25% EtOAc in $CH_2Cl_2$ in 5% increments) the product was obtained as a bright white solid (0.125 g, 17%).

**$^{1}$H NMR** (500 MHz, $CDCl_3$) δ: 9.59 (d, $J$ = 2.2 Hz, 1H, $H_n$), 9.41 (dd, $J$ = 2.3, 0.9 Hz, 1H, $H_a$), 9.03 (dd, $J$ = 2.3, 0.8 Hz, 1H, $H_i$), 8.87 (dd, $J$ = 4.9, 1.7 Hz, 1H, $H_b$), 8.46 (app. dt, $J$ = 8.0, 2.0 Hz, 1H, $H_d$), 8.22 (d, $J$ = 8.5 Hz, 1H, $H_m$), 8.01 (dd, $J$ = 8.2, 1.5 Hz, 1H, $H_j$), 7.90 (ddd, $J$ = 8.4, 6.9, 1.4 Hz, 1H, $H_l$), 7.68 (ddd, $J$ = 8.1, 7.0, 1.2 Hz, 1H, $H_k$), 7.55 (app. t, $J$ = 8.2 Hz, 1H, $H_f$), 7.49 (ddd, $J$ = 8.0, 4.8, 0.9 Hz, 1H, $H_c$), 7.30-7.26 (m, 2H, $H_h$, $H_e$/$H_g$), 7.23 (ddd, $J$ = 8.1, 2.2, 0.9 Hz, 1H, $H_e$/$H_g$).

**$^{13}$C NMR** (126 MHz, $CDCl_3$) δ: 163.8, 163.7, 154.4, 151.6, 151.3, 151.2, 150.4, 150.2, 139.9, 137.8, 132.6, 130.3, 129.8, 129.4, 127.9, 126.9, 125.5, 123.7, 122.3, 119.7, 119.6, 115.8.

**HR-ASAP-MS** $m/z$ = 371.1040 $[M+H]^+$ calc. 371.1032.

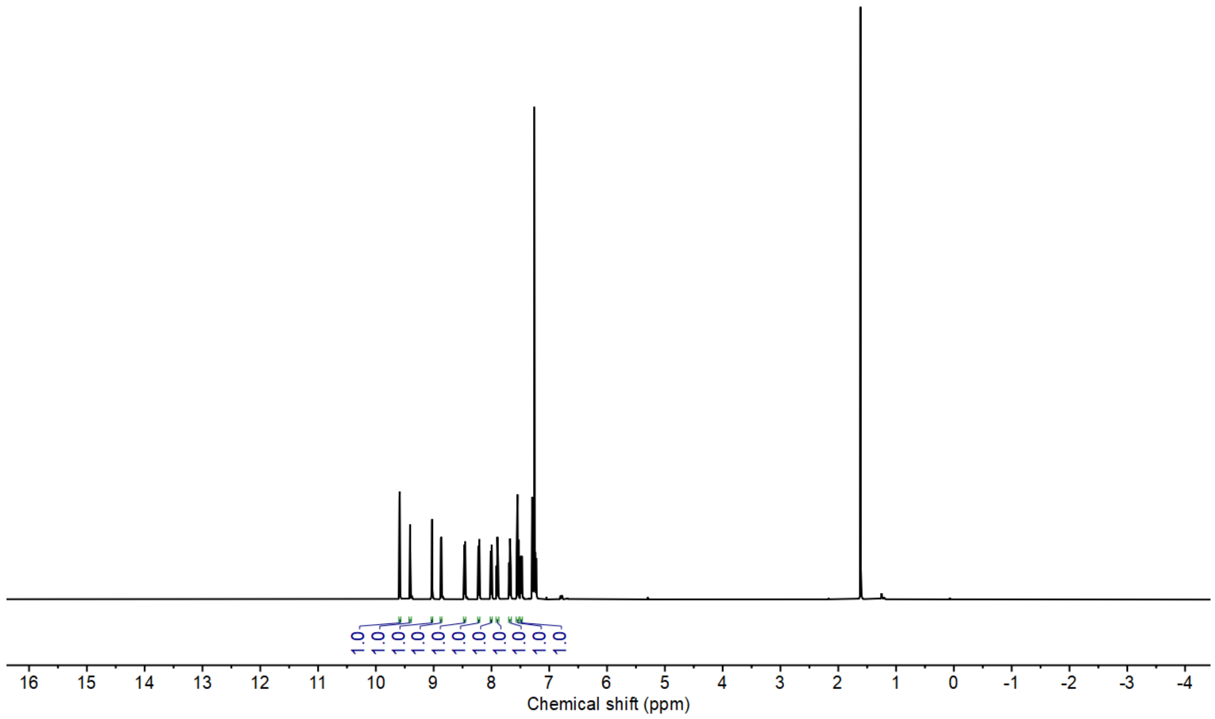


**Figure S119 $^{1}H$ NMR (500 MHz, CDCl₃) of L3$^{Q}$.**

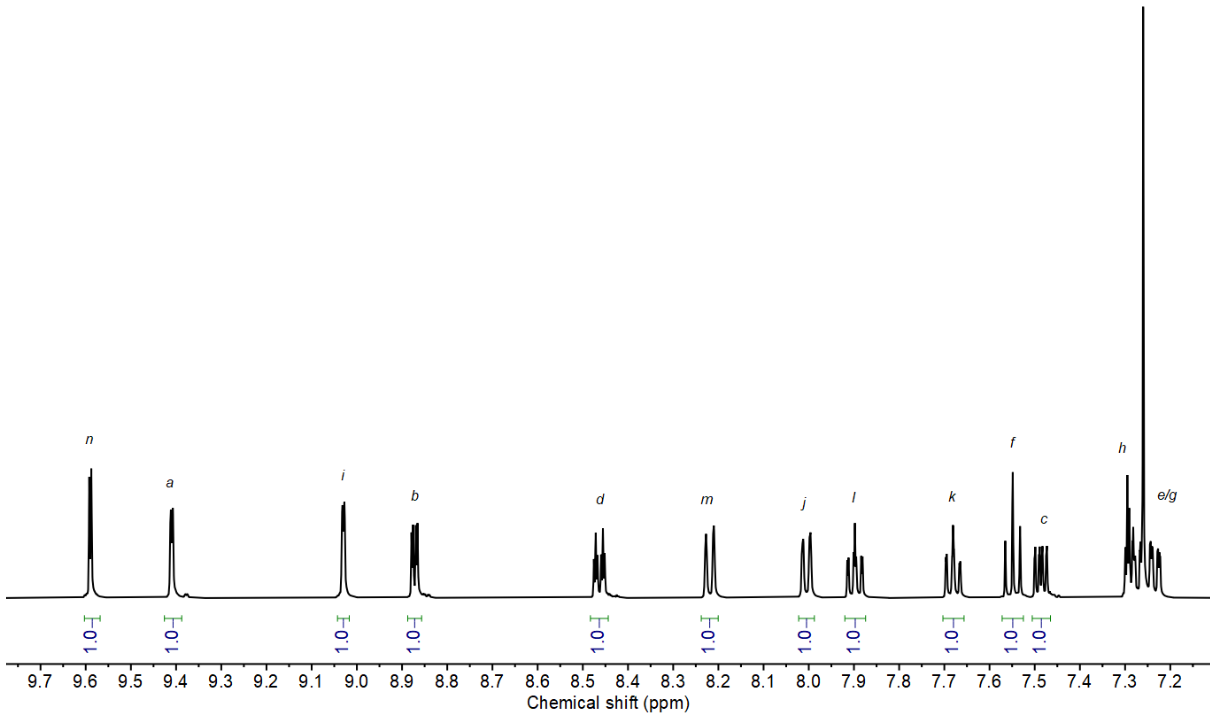


**Figure S120 Partial $^{1}H$ NMR (500 MHz, CDCl₃) of L3$^{Q}$.**

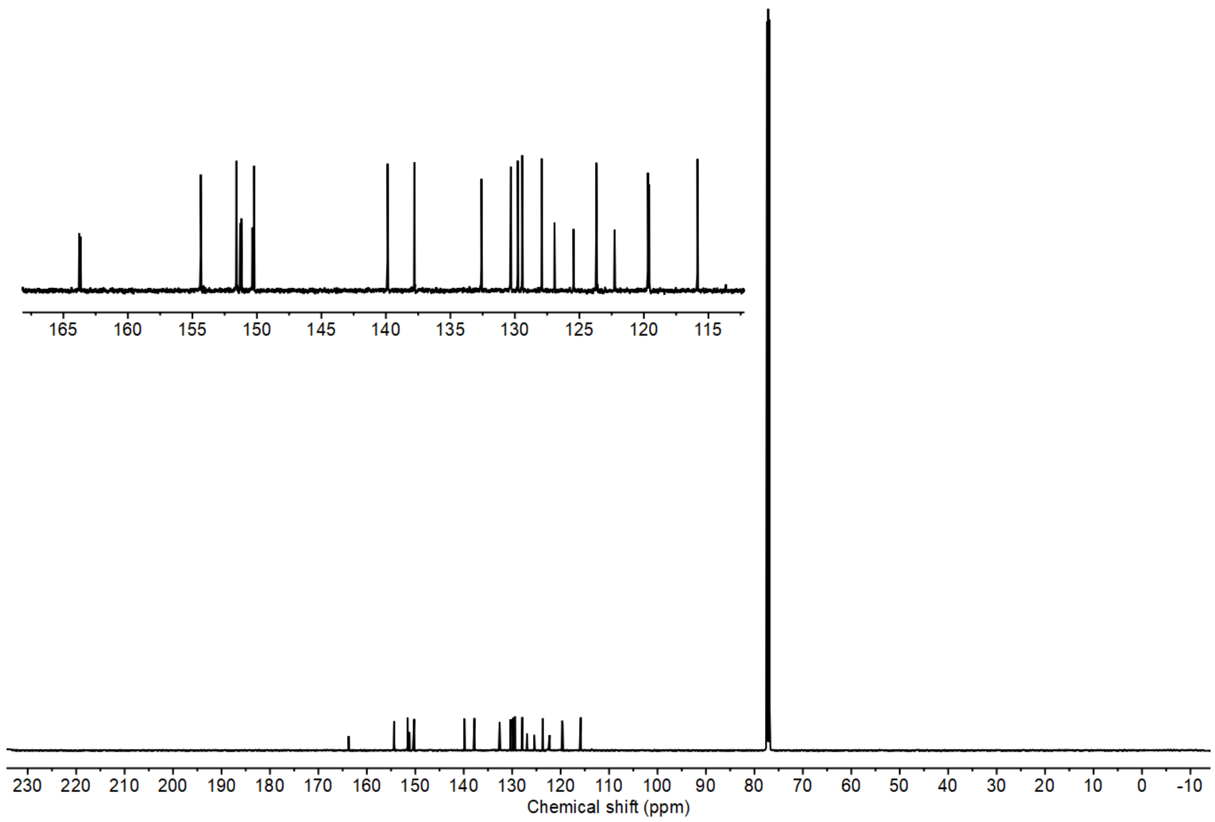


**Figure S121 $^{13}C$ NMR (126 MHz, $CDCl_3$) of L3$^{Q}$.**

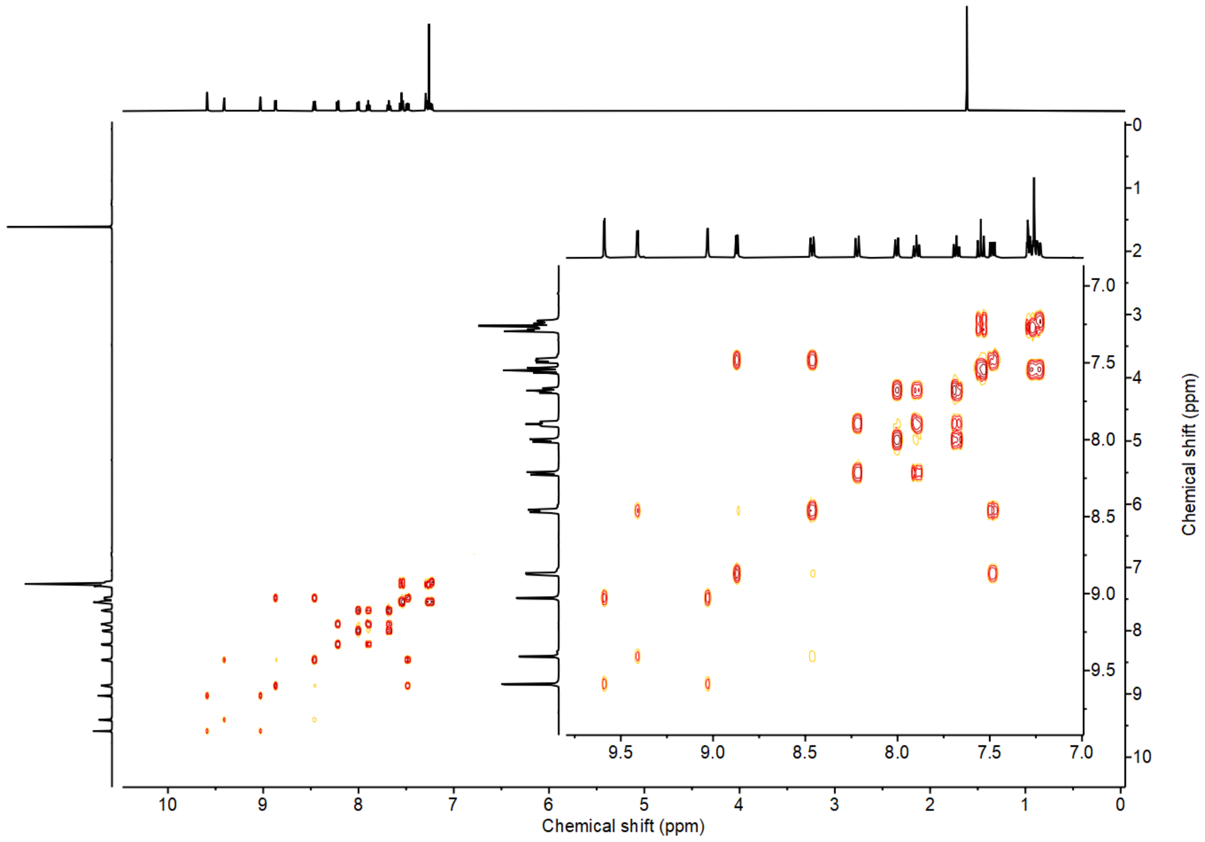


**Figure S122 COSY NMR ($CDCl_3$) of L3$^{Q}$.**

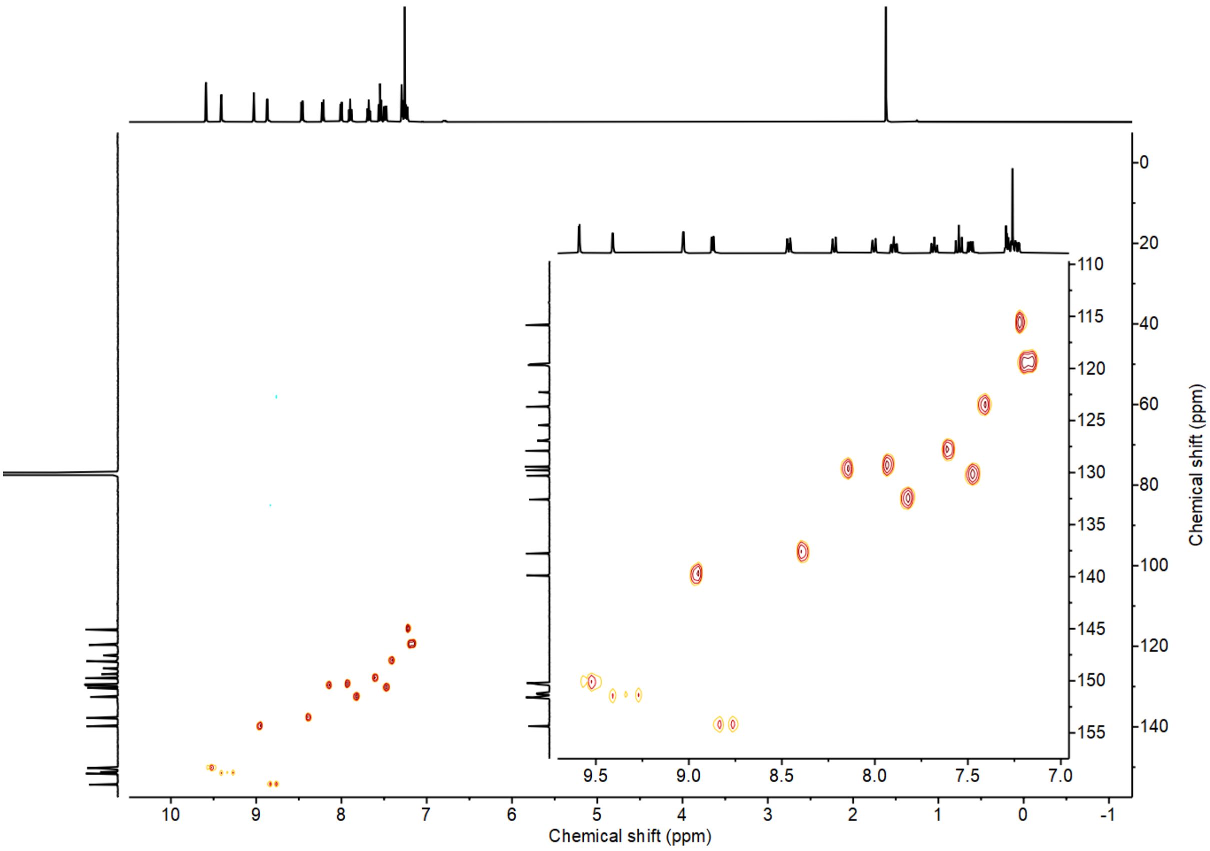


**Figure S123 HSQC NMR ($CDCl_3$) of L3[Q].**

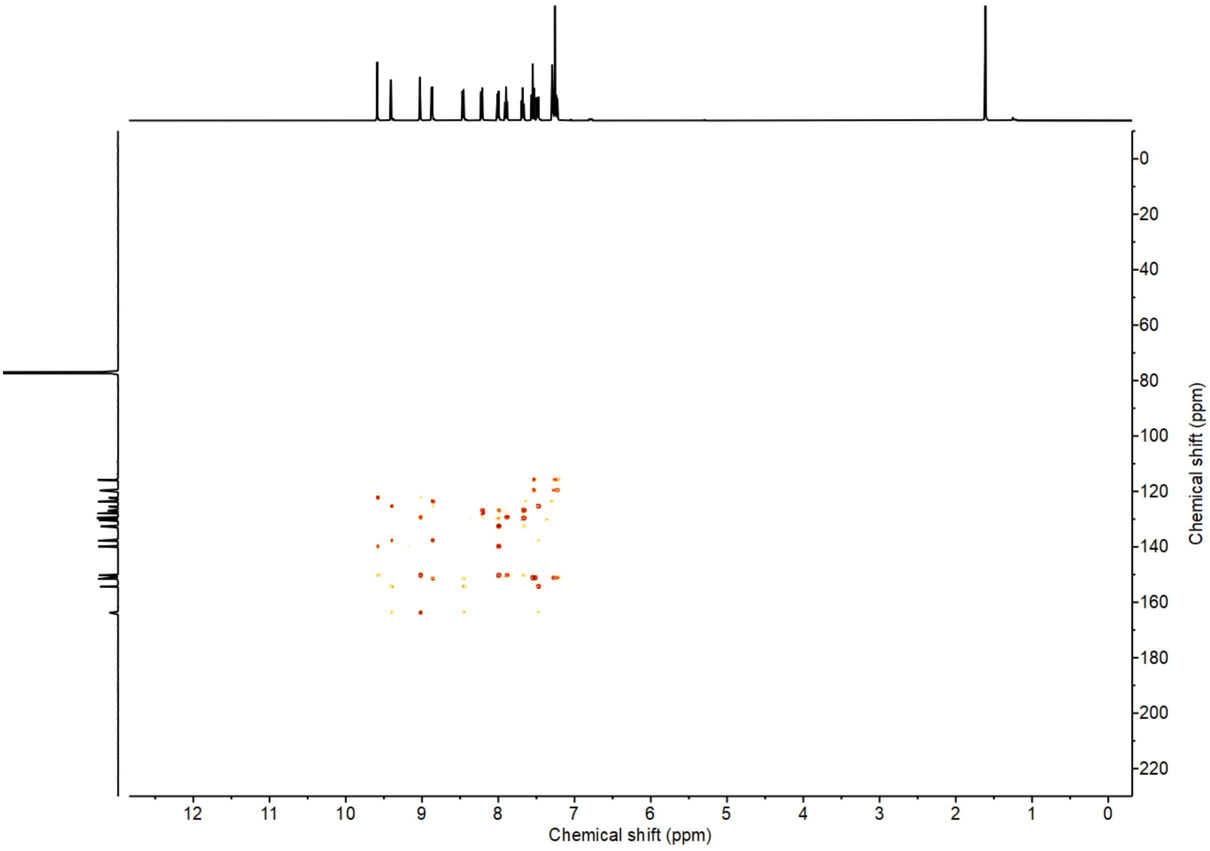


**Figure S124 HMBC NMR ($CDCl_3$) of L3[Q].**

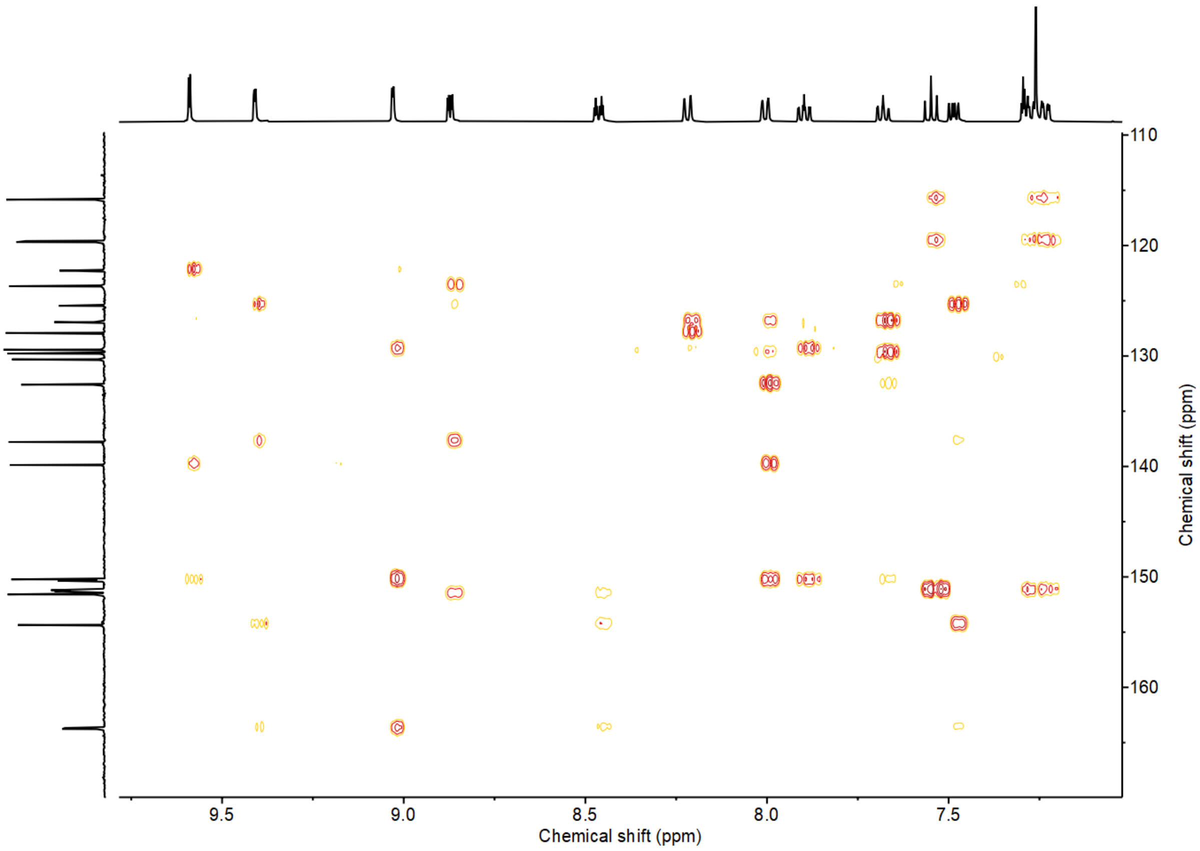


**Figure S125 Partial HMBC NMR (CDCl₃) of L3ᵠ.**

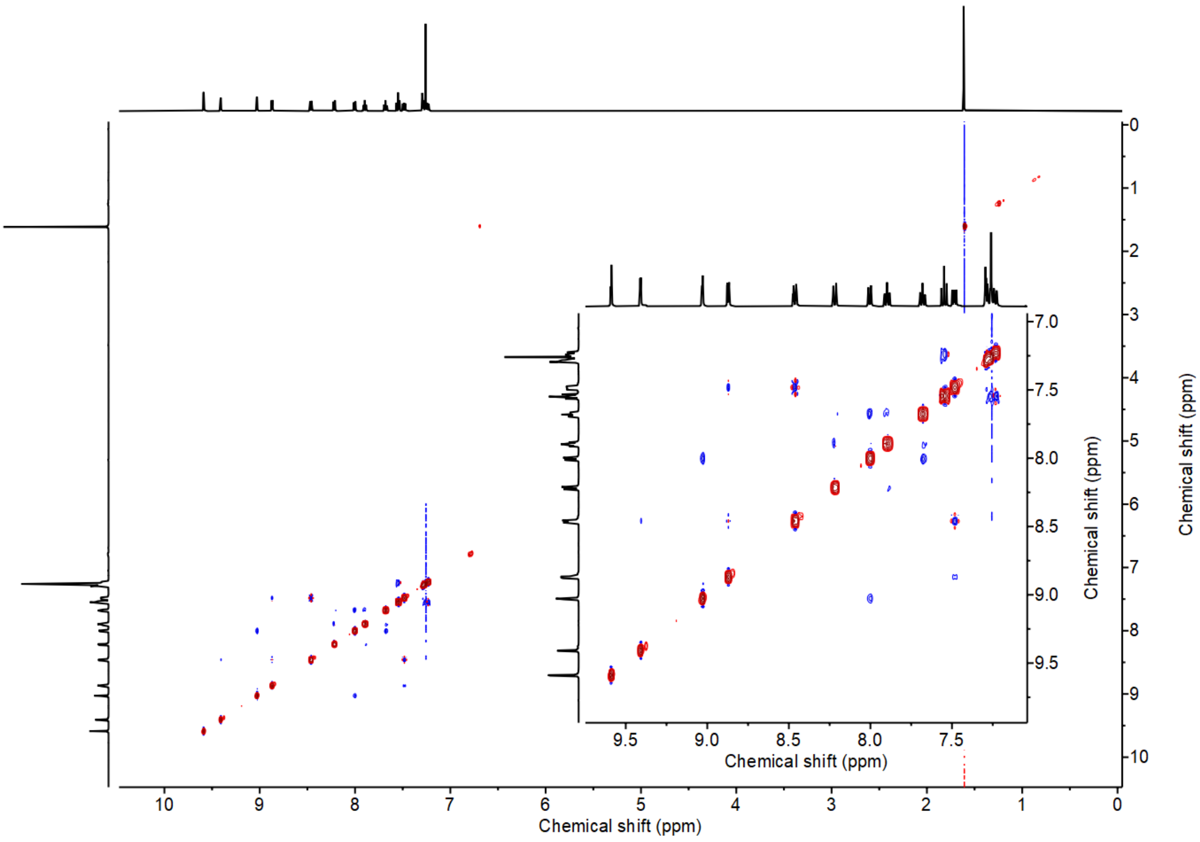


**Figure S126 NOESY ($CDCl_3$) of L3ᵠ.**

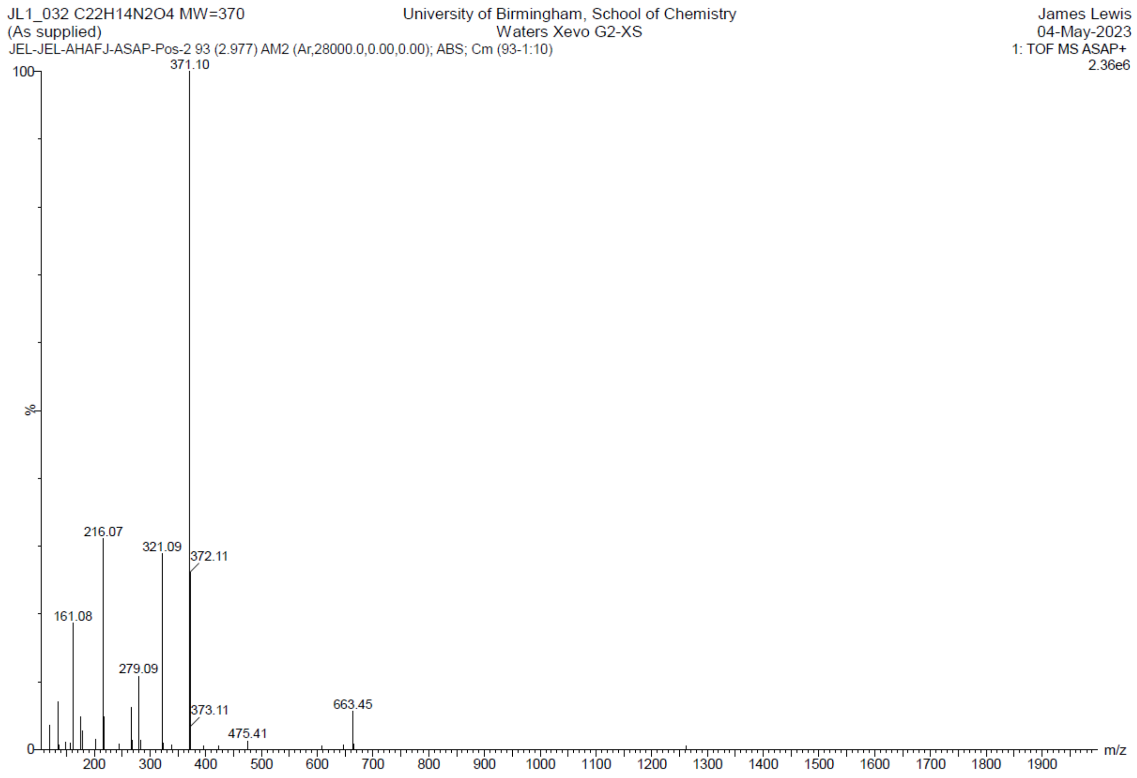


**Figure S127 ASAP-MS of L3$^{Q}$.**

## S2.17 Synthesis of *cis*-C3$^{Q}$

**L3$^{Q}$** (11.1 mg, 30 µmol, 2 eq.) and $[Pd(CH_3CN)_4](BF_4)_2$ (6.7 mg, 15 µmol, 1 eq.) were sonicated in *d*$_6$-DMSO (0.75 mL) until a homogenous solution was obtained. After standing at 60 °C for 4 h, $^{1}$H NMR showed formation of a single dominant species. Continued heating led to no further spectroscopic changes. The solution was then diluted with DMF (1 mL), filtered through celite, and the product precipitated through vapour diffusion of $Et_2O$. After the mother liquor was decanted, the solid was washed with $Et_2O$ (× 3) and dried *in vacuo* to give the product as a beige solid (11.3 mg, 74%).

**$^{1}$H NMR** (500 MHz, *d*$_6$-DMSO) δ: 10.99 (s, 6H, $H_n$), 10.65 (d, $J$ = 8.7 Hz, 6H, $H_m$), 10.46 (s, 6H, $H_a$), 10.20 (d, $J$ = 5.8 Hz, 6H, $H_b$), 9.58 (s, 6H, $H_i$), 8.61 (d, $J$ = 8.1 Hz, 6H, $H_d$), 8.53-8.48 (m, 6H, $H_l$), 8.39 (d, $J$ = 8.0 Hz, 6H, $H_j$), 8.04 (dd, $J$ = 8.1, 5.6 Hz, 6H, $H_c$), 7.99 (app. t, $J$ = 7.6 Hz, 6H, $H_k$), 7.73 (app. t, $J$ = 8.5 Hz, 6H, $H_f$), 7.45 (d, $J$ = 7.5 Hz, 6H, $H_e$/$H_g$), 7.38-7.36 (m, 12H, $H_e$/$H_g$, $H_h$).

**$^{13}$C NMR** (126 MHz, *d*$_6$-DMSO) δ: 162.3, 162.2, 161.3, 155.1 ($C_b$), 154.7, 153.2 ($C_a$), 150.7, 146.2, 144.1 ($C_i$), 141.7 ($C_d$), 135.9 ($C_l$), 131.5 ($C_j$), 130.8 ($C_f$), 130.1 ($C_k$), 128.6, 128.0, 127.8 ($C_c$), 126.8, 124.6, 120.8 ($C_e$/$C_g$), 120.4 ($C_e$/$C_g$), 117.2 ($C_h$).

**Diffusion coefficient** (400 MHz, *d*$_6$-DMSO) $D$: 6.71 × $10^{-11}$ $m^2s^{-1}$.

**HR-ESI-MS** $m/z$ = 423.55 $\{[Pd_2(\mathbf{L3^Q})_4]\}^{4+}$ calc. 423.55; 508.06 $\{[Pd_3(\mathbf{L3^Q})_6]\}^{5+}$ calc. 508.06; 512.06 $\{[Pd_3(\mathbf{L3^Q})_6]F\}^{5+}$ calc. 511.86; 571.06 $\{[Pd_2(\mathbf{L3^Q})_4]F\}^{3+}$ calc. 571.06; 579.73 $\{[Pd_2(\mathbf{L3^Q})_4](HCO_2)\}^{3+}$ calc. 579.73; 644.82 $\{[Pd_3(\mathbf{L3^Q})_6]F_2\}^{4+}$ calc. 644.57; 678.57 $\{[Pd_3(\mathbf{L3^Q})_6](BF_4)_2\}^{4+}$ calc. 678.57; 911.43 $\{[Pd_3(\mathbf{L3^Q})_6](BF_4)_2F\}^{3+}$ calc. 911.10; 934.10 $\{[Pd_3(\mathbf{L3^Q})_6](BF_4)_3\}^{3+}$ calc. 933.77; 1375.64 $\{[Pd_3(\mathbf{L3^Q})_6](BF_4)_2F_2\}^{2+}$ calc. 1376.15; 1410.66 $\{[Pd_3(\mathbf{L3^Q})_6](BF_4)_3F\}^{2+}$ calc. 1410.15; 1443.65 $\{[Pd_3(\mathbf{L3^Q})_6](BF_4)_4\}^{2+}$ calc. 1444.15.

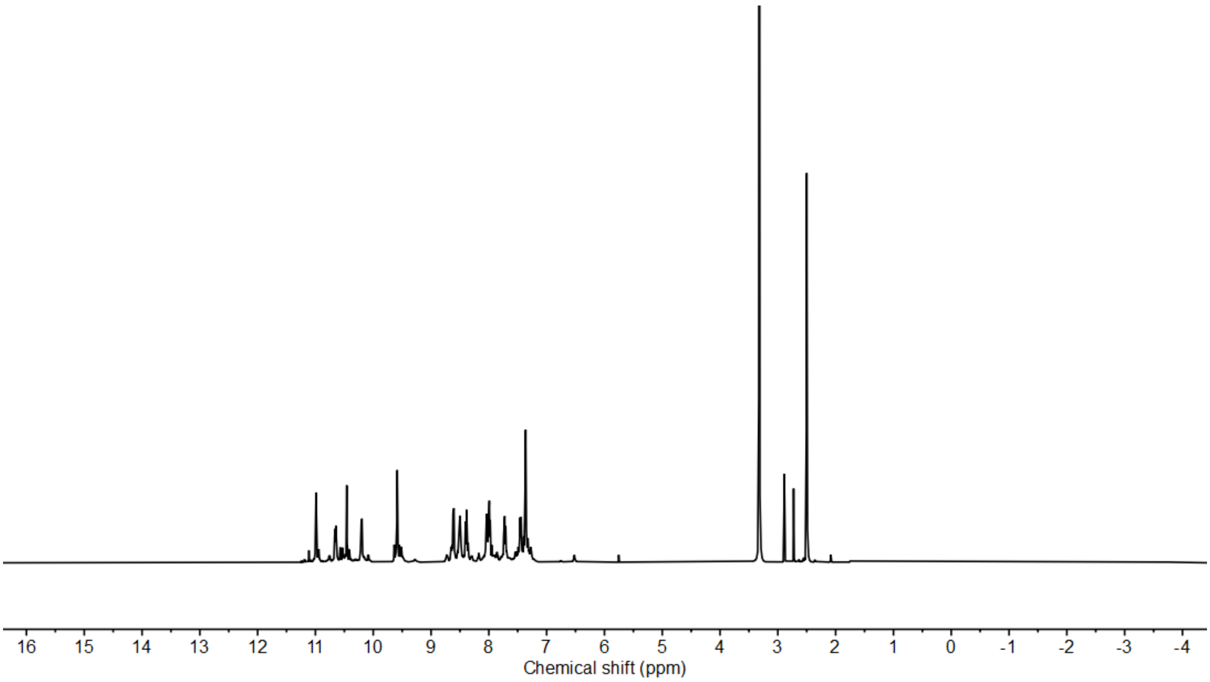


**Figure S128 $^{1}$H NMR (500 MHz, *d*$_6$-DMSO) of C3$^{Q}$.**

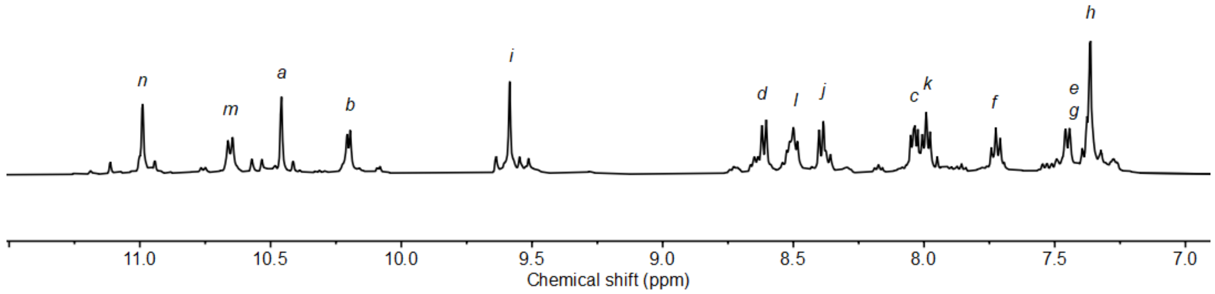


**Figure S129 Partial $^{1}H$ NMR (500 MHz, $d_6$-DMSO) of C3$^{Q}$.**

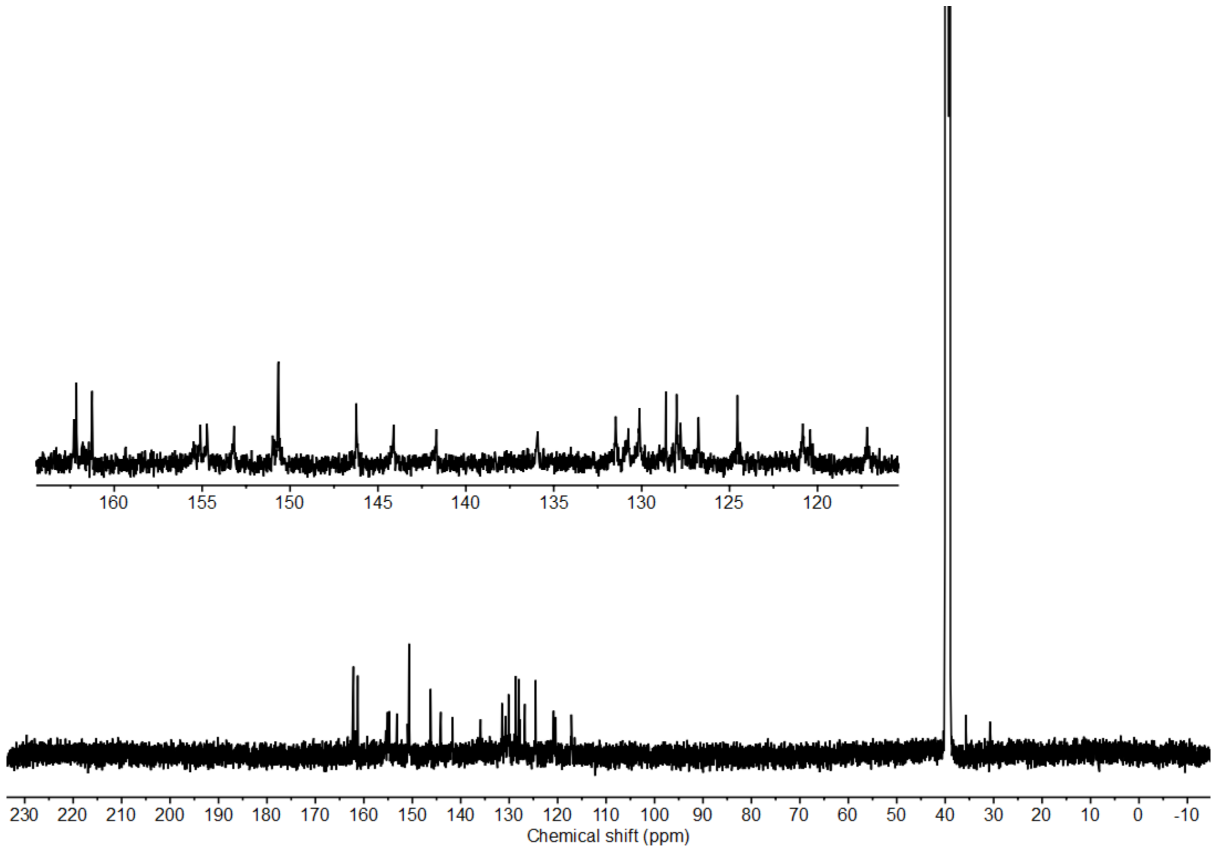


**Figure S130 $^{13}C$ NMR (126 MHz, $d_6$-DMSO) of C3$^{Q}$.**

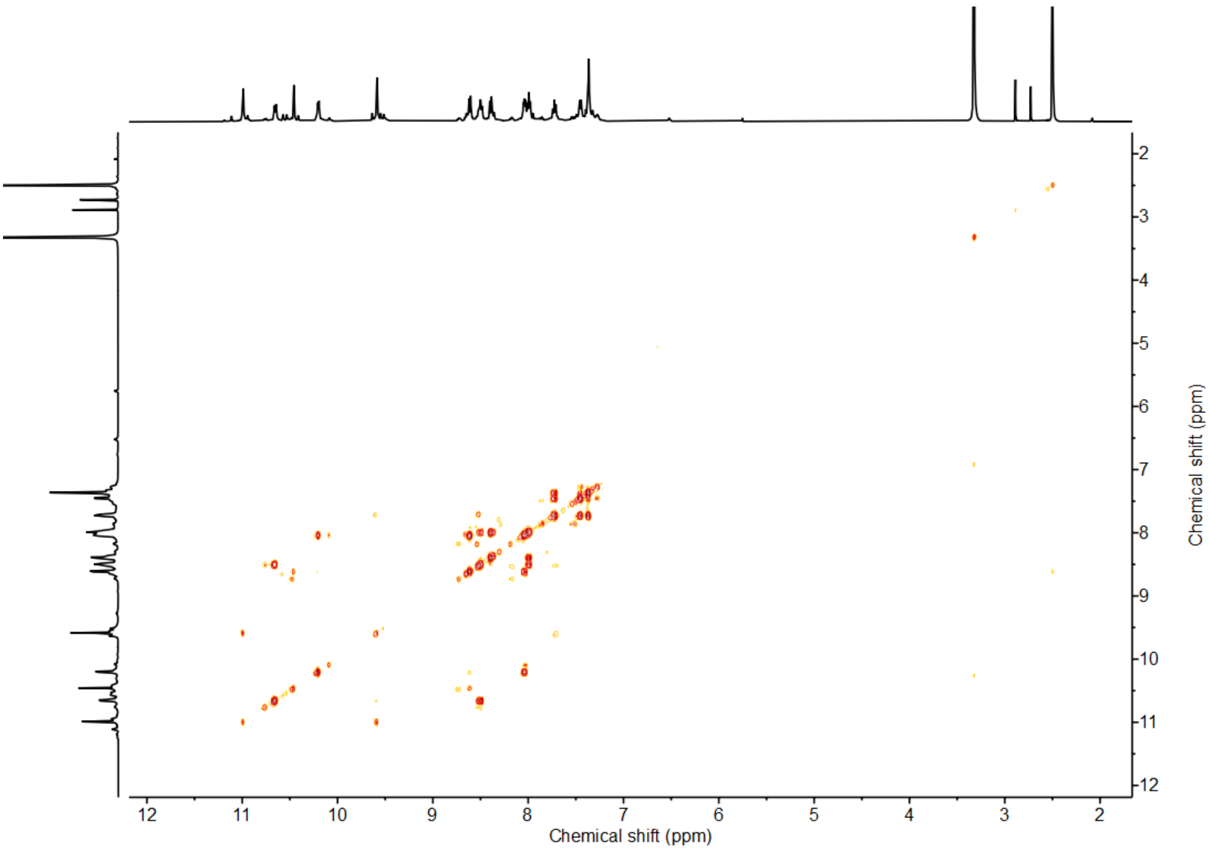


**Figure S131 COSY ($d_6$-DMSO) of C3$^{Q}$.**

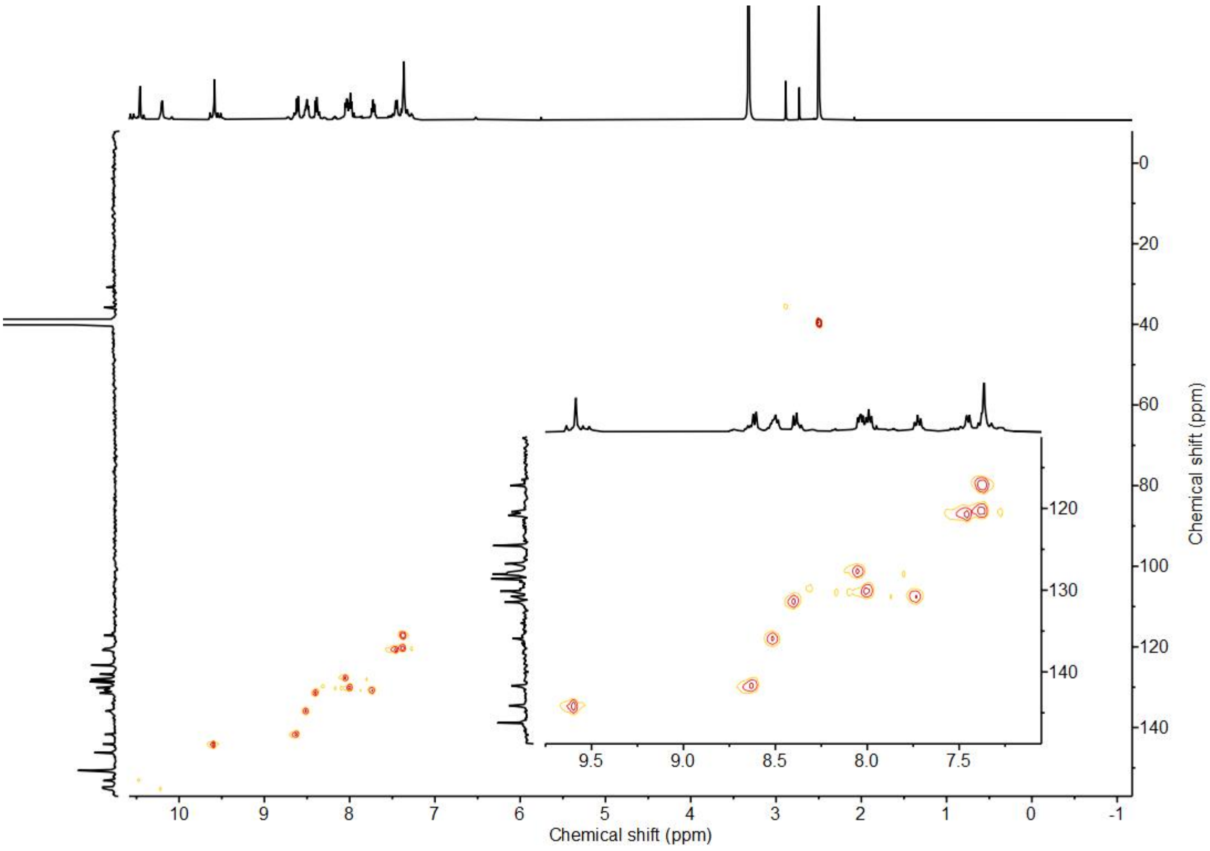


**Figure S132 HSQC ($d_6$-DMSO) of C3$^{Q}$.**

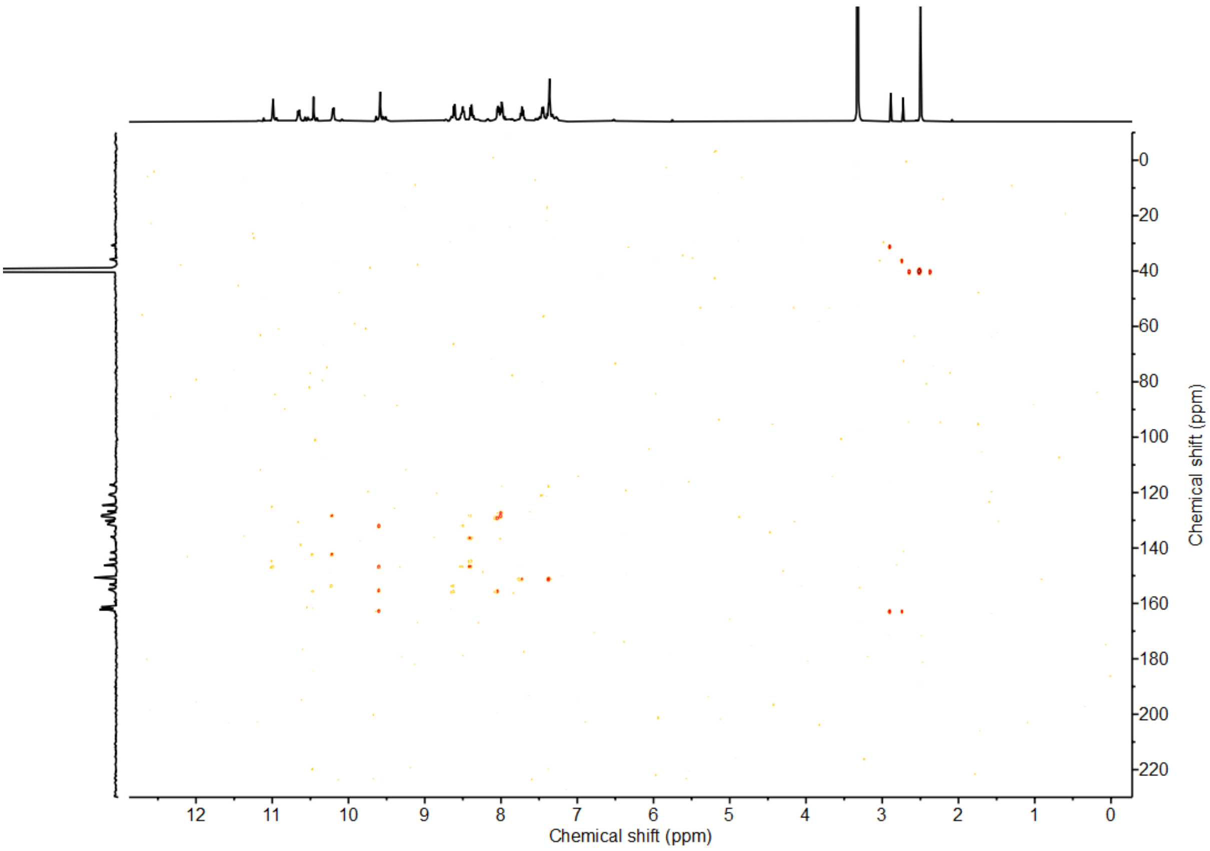


**Figure S133 HMBC ($d_6$-DMSO) of C3$^{Q}$.**

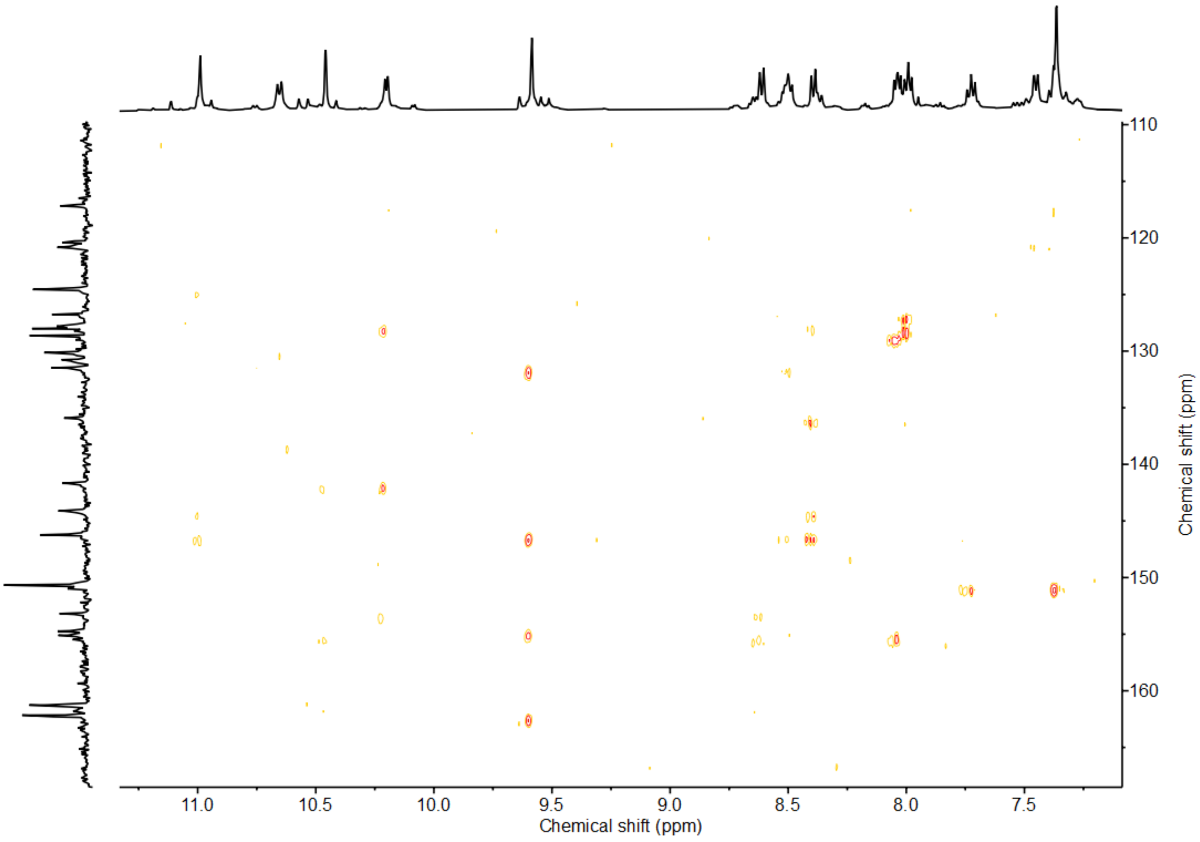


**Figure S134 Partial HMBC ($d_6$-DMSO) of C3$^{Q}$.**

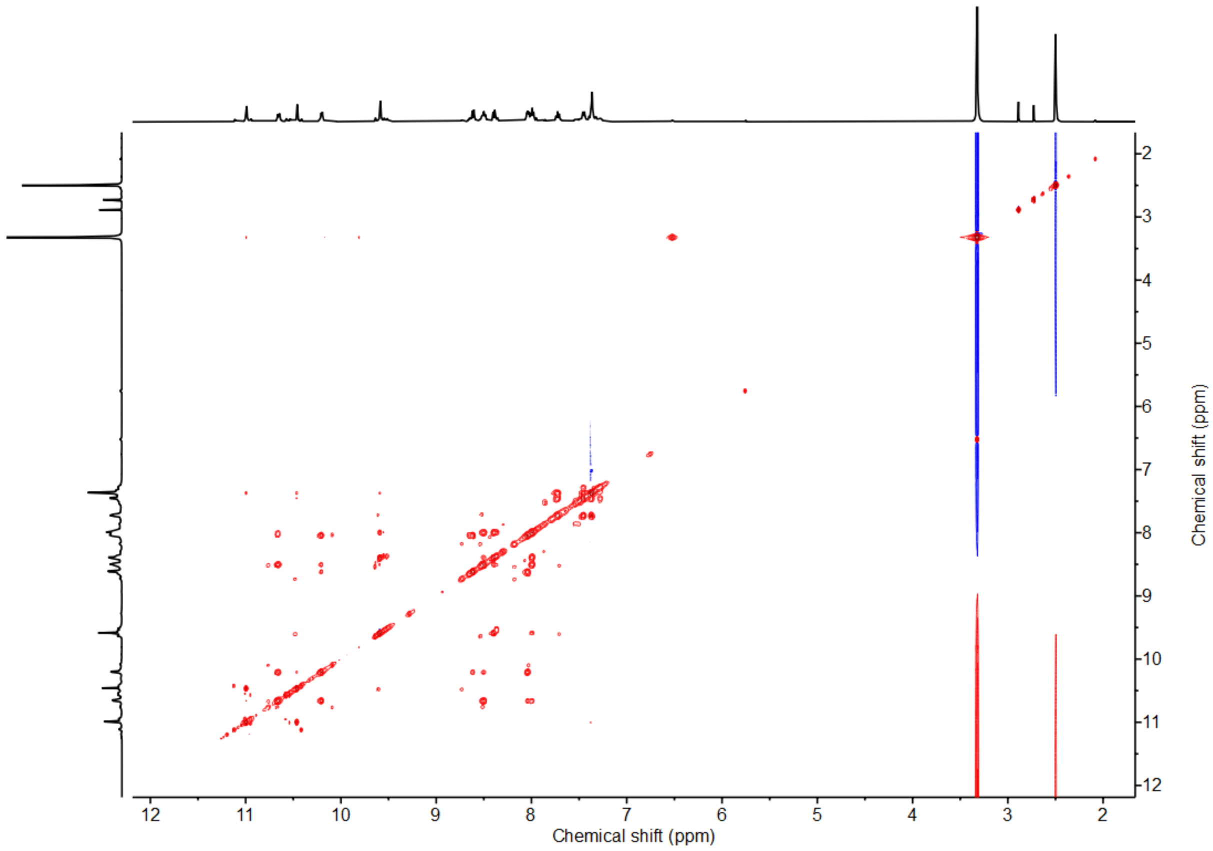


**Figure S135 NOESY ($d_6$-DMSO) of C3$^{Q}$.**

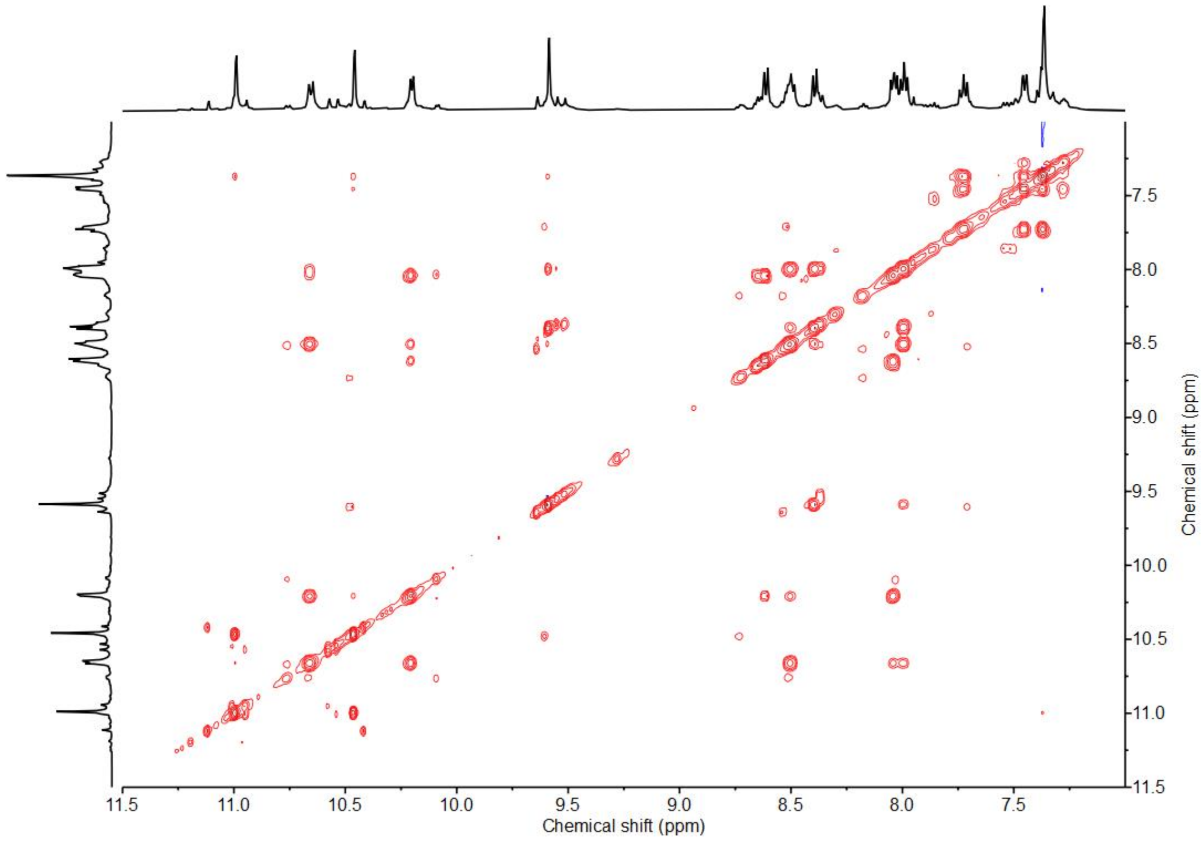


**Figure S136 Partial NOESY ($d_6$-DMSO) of C3$^{Q}$.**

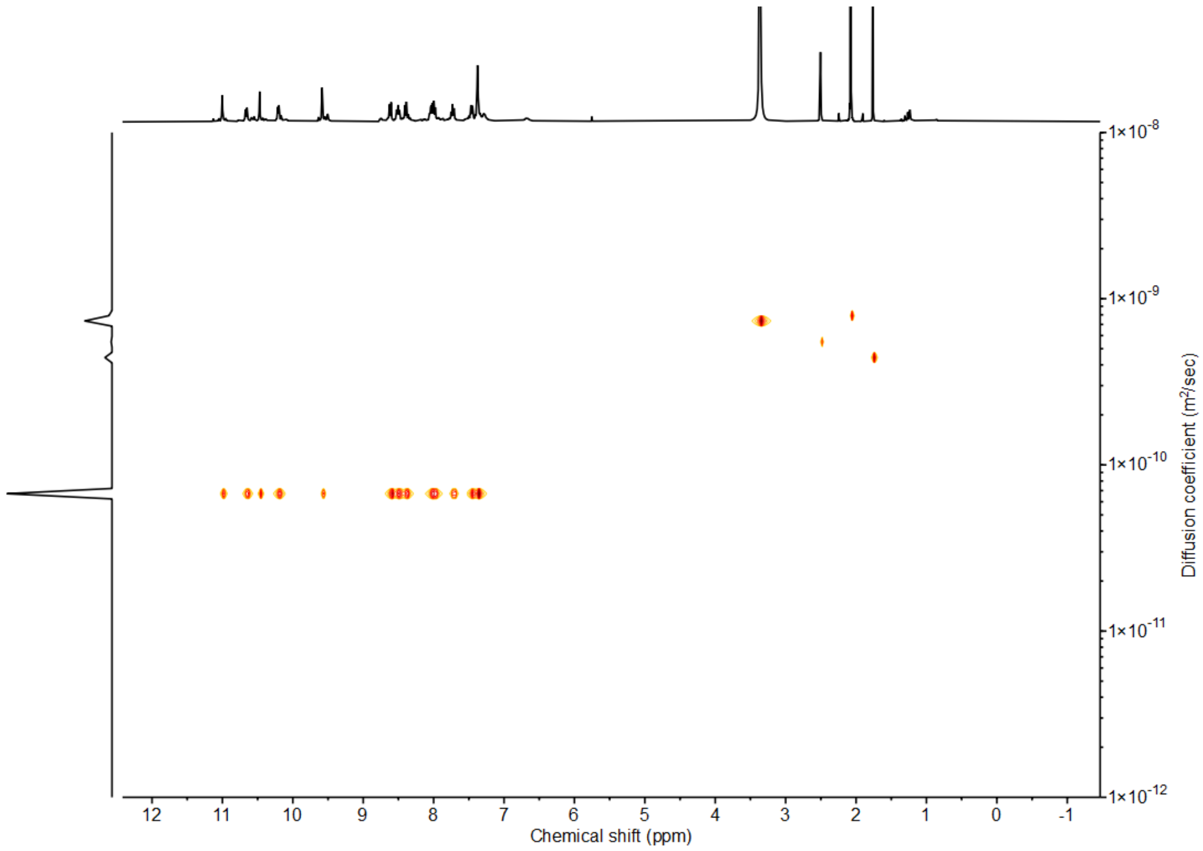


**Figure S137 DOSY (400 MHz, $d_6$-DMSO) of C3$^{Q}$.**

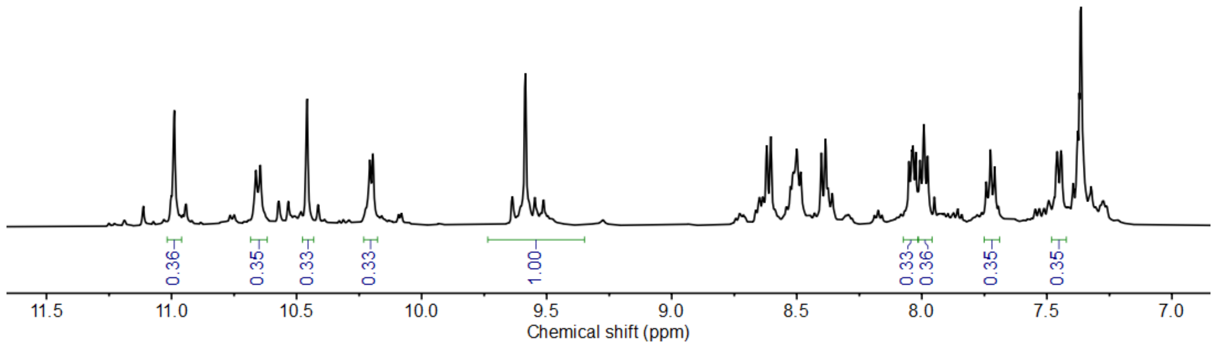


**Figure S138 Partial $^{1}$H NMR (500 MHz, $d_6$-DMSO) of C3$^{Q}$ with integrals relative to sum total of $H_i$ signals.**

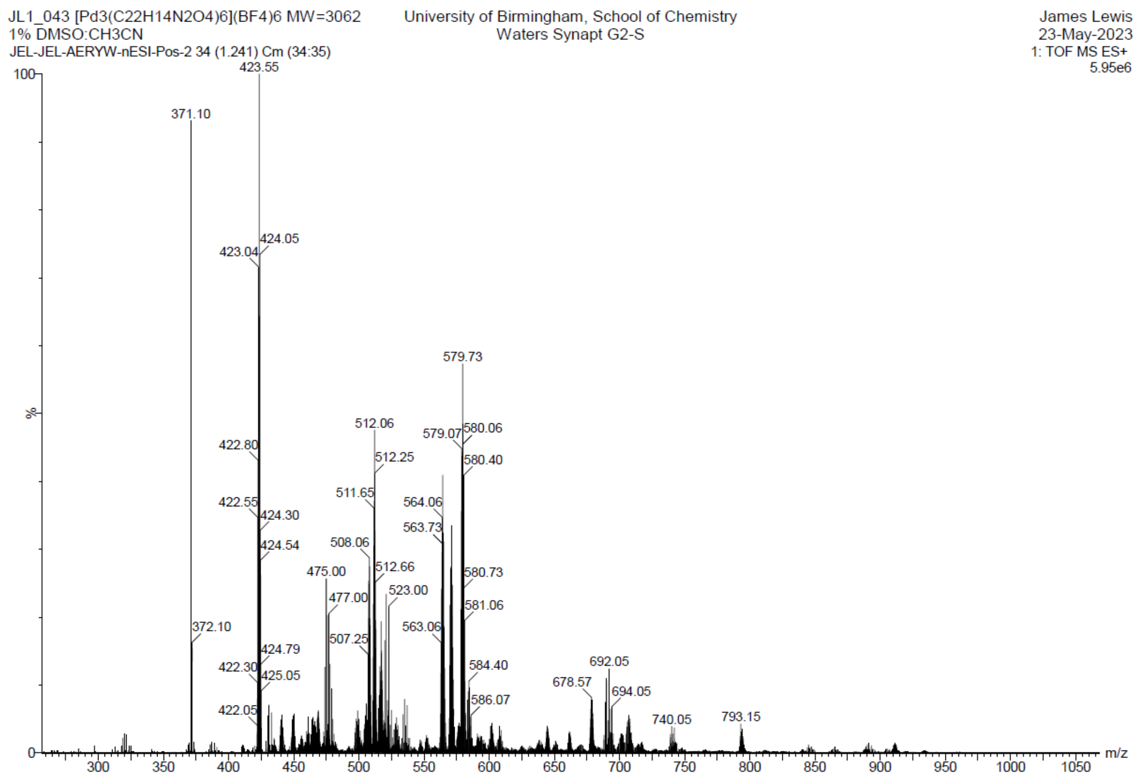


**Figure S139 ESI-MS of C3Q.**

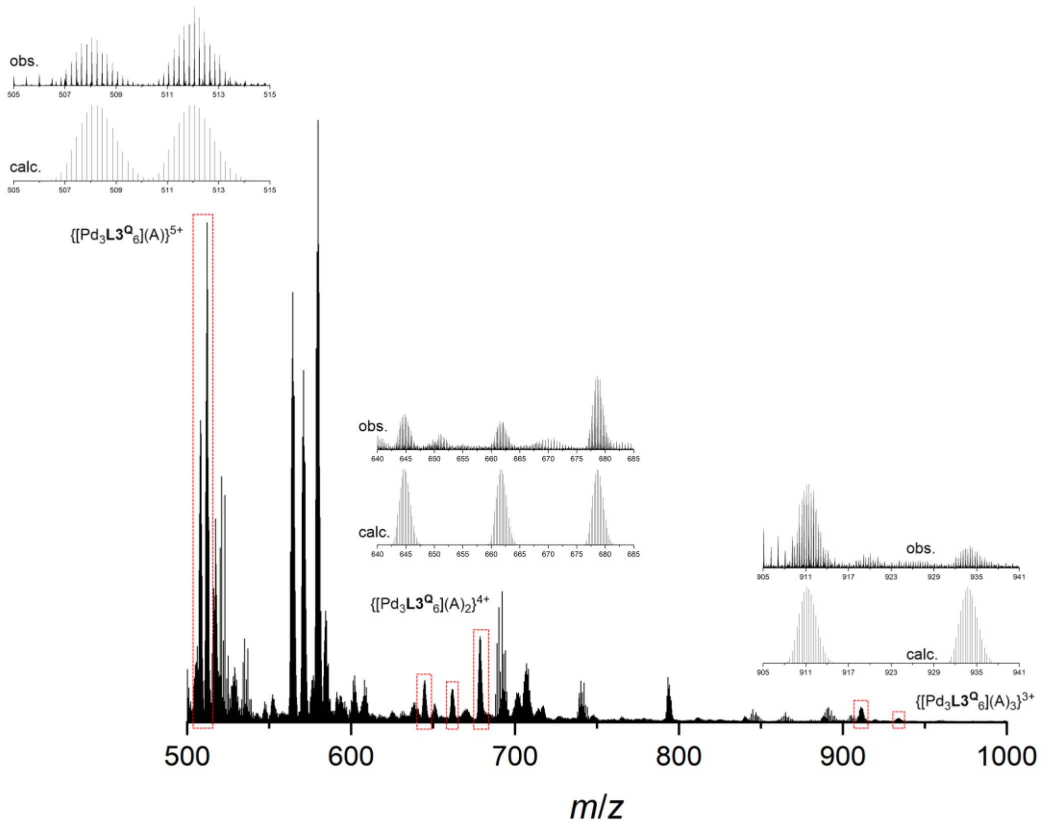


**Figure S140 ESI-MS of C3Q with key peaks labelled.**

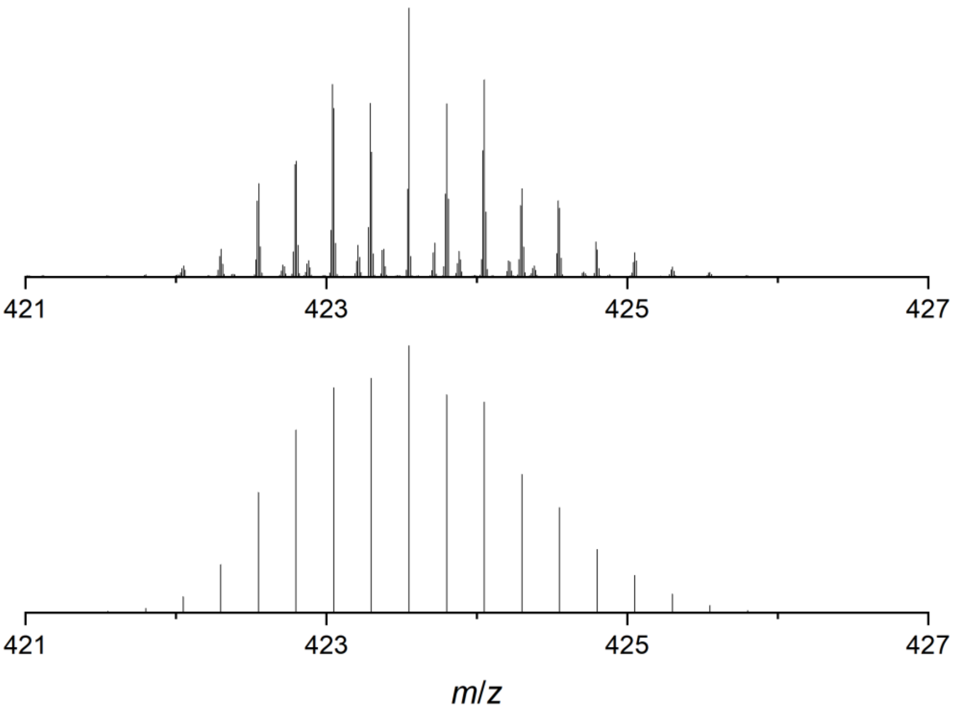


**Figure S141 Observed (top) and calculated (bottom) isotopic patterns for $\{[Pd_2(L3^Q)_4]\}^{4+}$.**

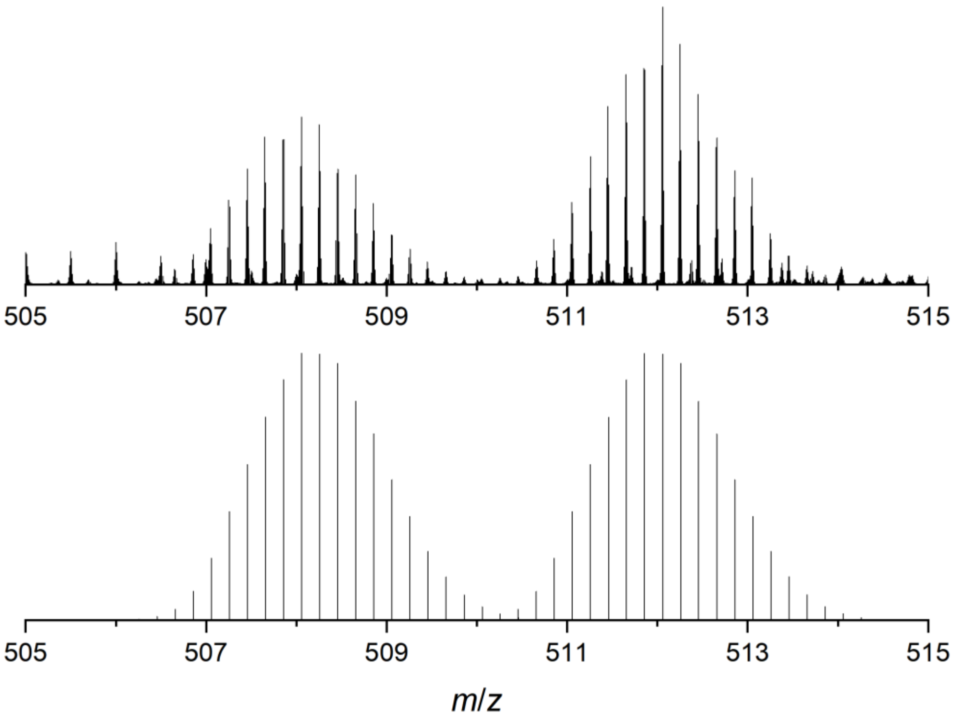


**Figure S142 Observed (top) and calculated (bottom) isotopic patterns for (from left to right) $\{[Pd_3(L3^Q)_6]\}^{5+}$ and $\{[Pd_3(L3^Q)_6]F\}^{5+}$.**

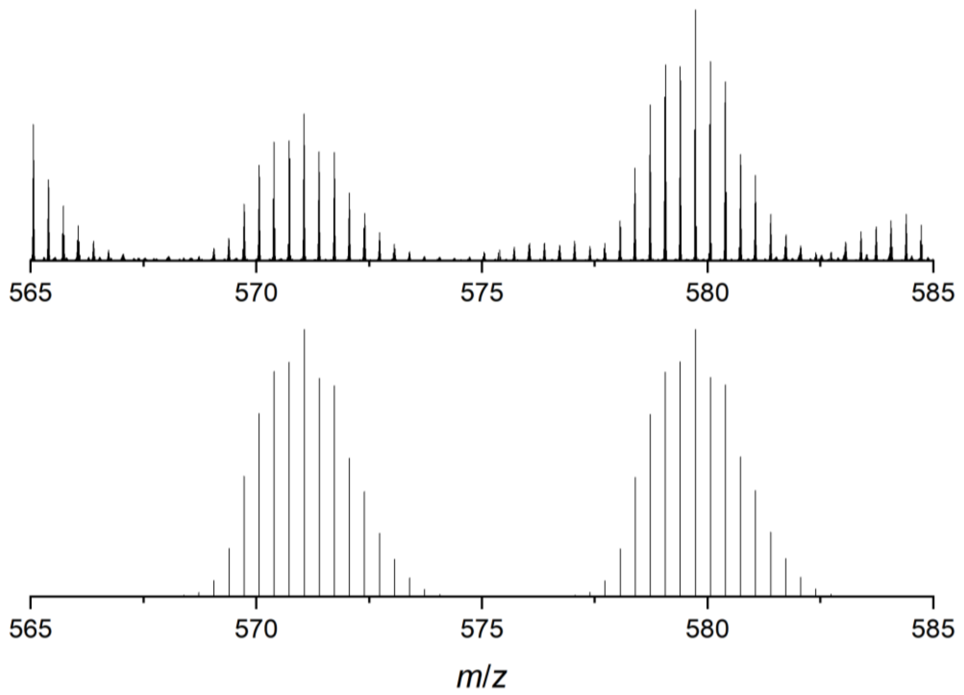


**Figure S143 Observed (top) and calculated (bottom) isotopic patterns for (from left to right) $\{[Pd_2(L3^Q)_4]F\}^{3+}$ and $\{[Pd_2(L3^Q)_4](HCO_2)\}^{3+}$.**

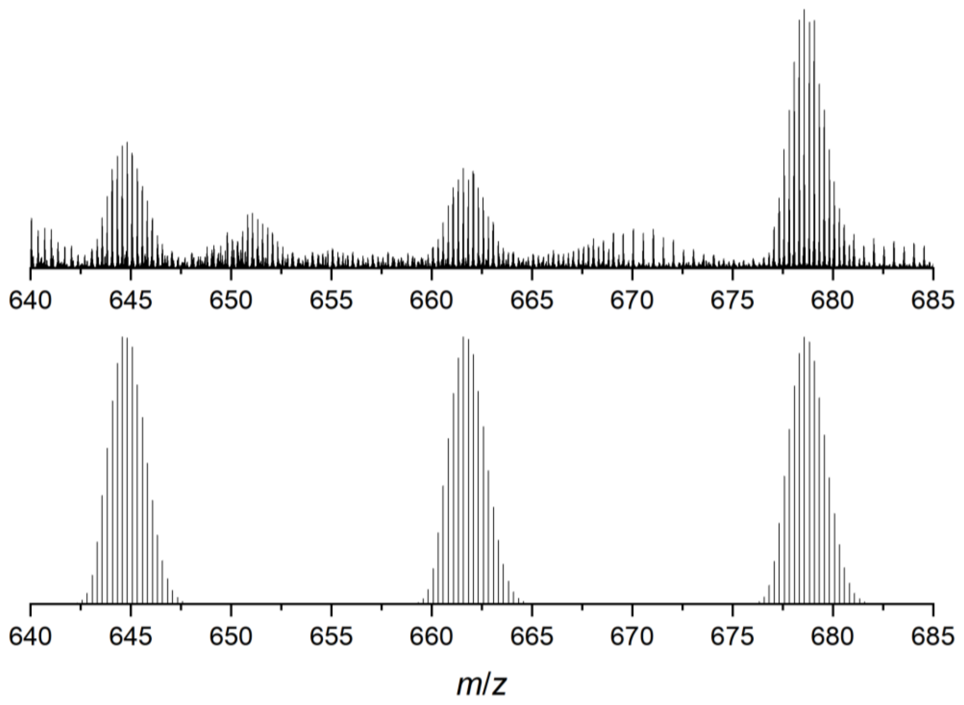


**Figure S144 Observed (top) and calculated (bottom) isotopic patterns for (from left to right) $\{[Pd_3(L3^Q)_6]F_2\}^{4+}$, $\{[Pd_3(L3^Q)_6](BF_4)F\}^{4+}$ and $\{[Pd_3(L3^Q)_6](BF_4)_2\}^{4+}$.**

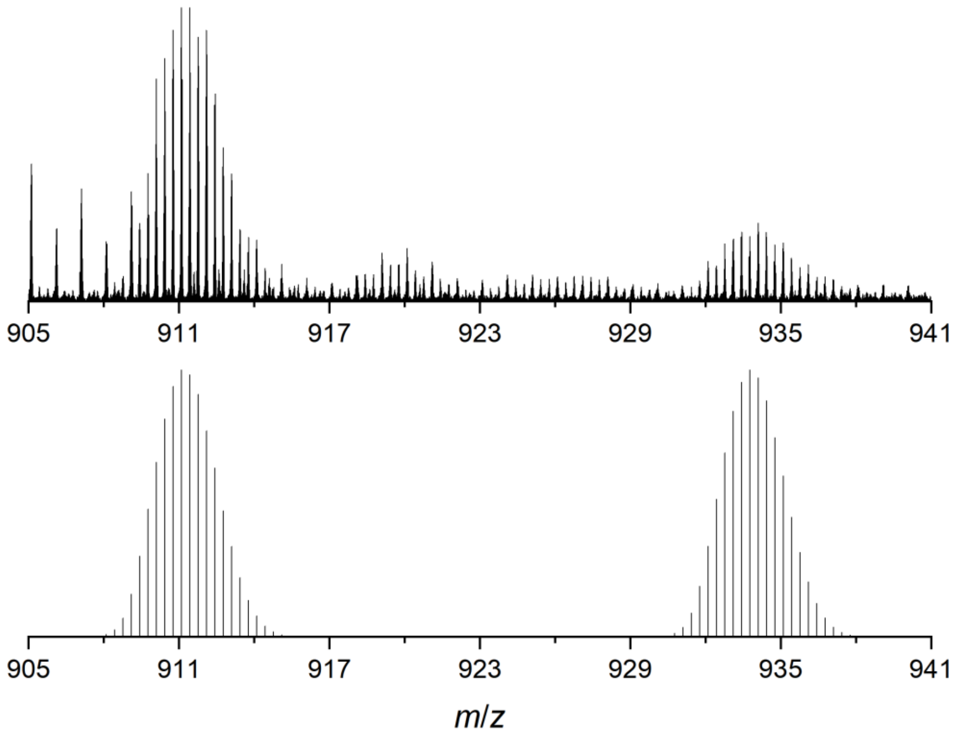


**Figure S145 Observed (top) and calculated (bottom) isotopic patterns for (from left to right) $\{[Pd_3(L3^Q)_6](BF_4)_2F_2\}^{3+}$ and $\{[Pd_3(L3^Q)_6](BF_4)_3\}^{3+}$.**

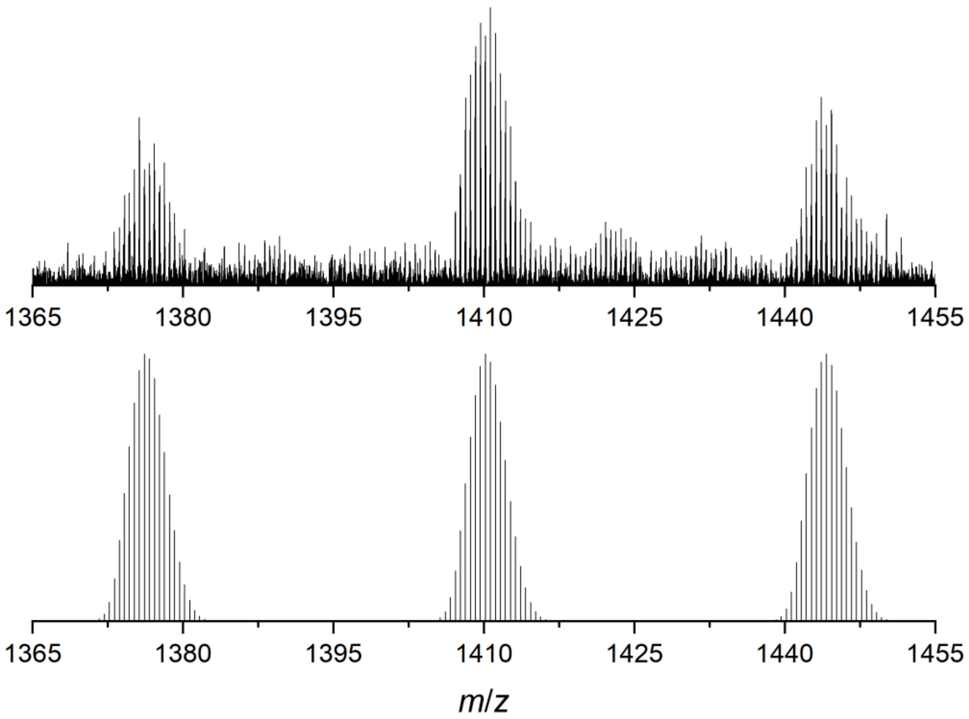


**Figure S146 Observed (top) and calculated (bottom) isotopic patterns for (from left to right) $\{[Pd_3(L3^Q)_6](BF_4)_2F_2\}^{2+}$, $\{[Pd_3(L3^Q)_6](BF_4)_3F\}^{2+}$ and $\{[Pd_3(L3^Q)_6](BF_4)_4\}^{2+}$.**

## S2.18 Synthesis of S3

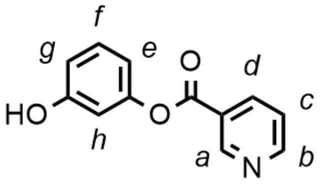


To solution of resorcinol (0.661 g, 6.0 mmol, 2 eq.) and $NEt_3$ (0.84 mL, 6.0 mmol, 2 eq.) in THF (20 mL) at 0 °C was added nicotinoyl chloride hydrochloride (0.534 g, 3.0 mmol, 1 eq.) portionwise as a solid. The reaction was allowed to warm to rt and stirred for 5.5 h. The solvent was removed *in vacuo* and the residue extracted with $CH_2Cl_2$ (3 × 15 mL). Following purification by column chromatography on silica gel (step gradient 0 to 30% EtOAc in $CH_2Cl_2$ in 10% increments) the product was obtained as a white solid (0.149 g, 23%).

**$^1$H NMR** (500 MHz, $d_6$-DMSO) δ: 9.78 (s, 1H, $H_{OH}$), 9.24 (dd, *J* = 2.2, 0.9 Hz, 1H, $H_a$), 8.89 (dd, *J* = 4.8, 1.7 Hz, 1H, $H_b$), 8.45 (dt, *J* = 8.0, 2.1 Hz, 1H, $H_d$), 7.64 (dd, *J* = 8.0, 4.9 Hz, 1H, $H_c$), 7.25 (t, *J* = 8.1 Hz, 1H, $H_f$), 6.74-6.70 (m, 3H, $H_e$, $H_g$, $H_h$).

**$^{13}$C NMR** (126 MHz, $d_6$-DMSO) δ: 163.5, 158.4, 154.2 ($C_b$), 151.2, 150.5 ($C_a$), 137.5 ($C_d$), 130.0 ($C_f$), 125.2, 124.1 ($C_c$), 113.3 ($C_h$), 112.2 ($C_e$), 109.0 ($C_g$).

**HR-ESI-MS** *m/z* = 216.0666 $[M+H]^+$ calc. 216.0661.

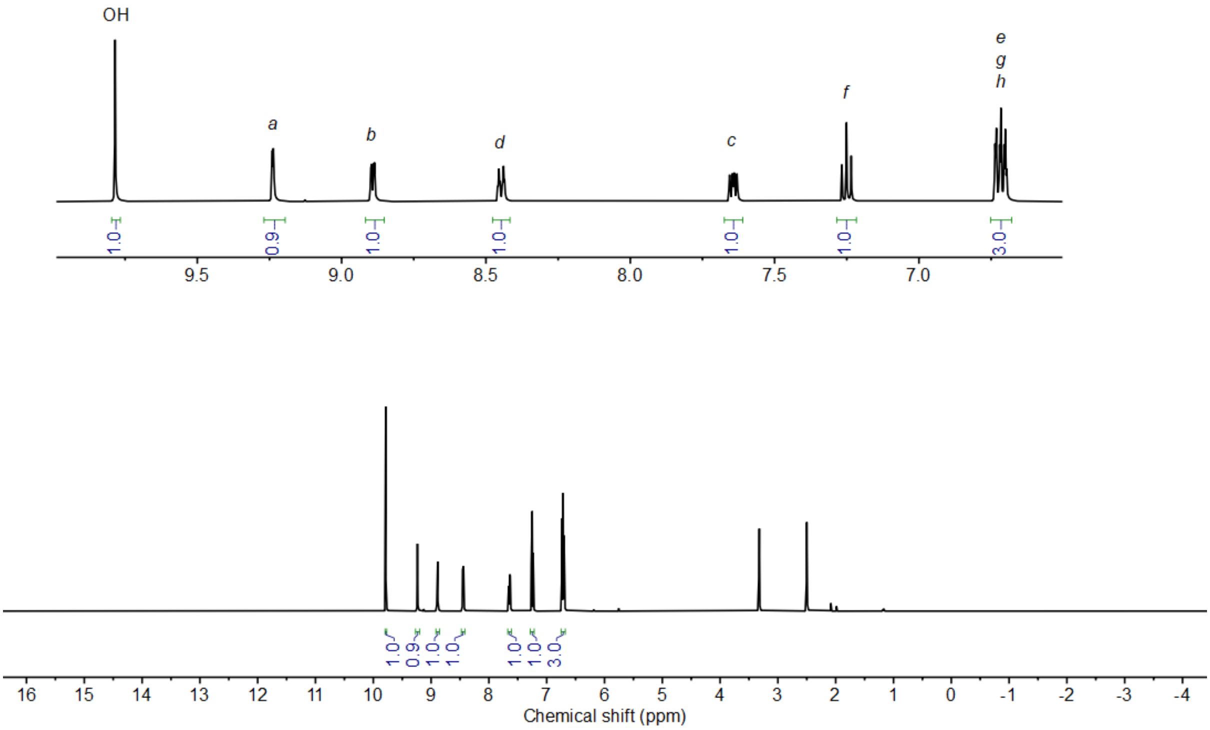


**Figure S147 $^1$H NMR (500 MHz, $d_6$-DMSO) of S3.**

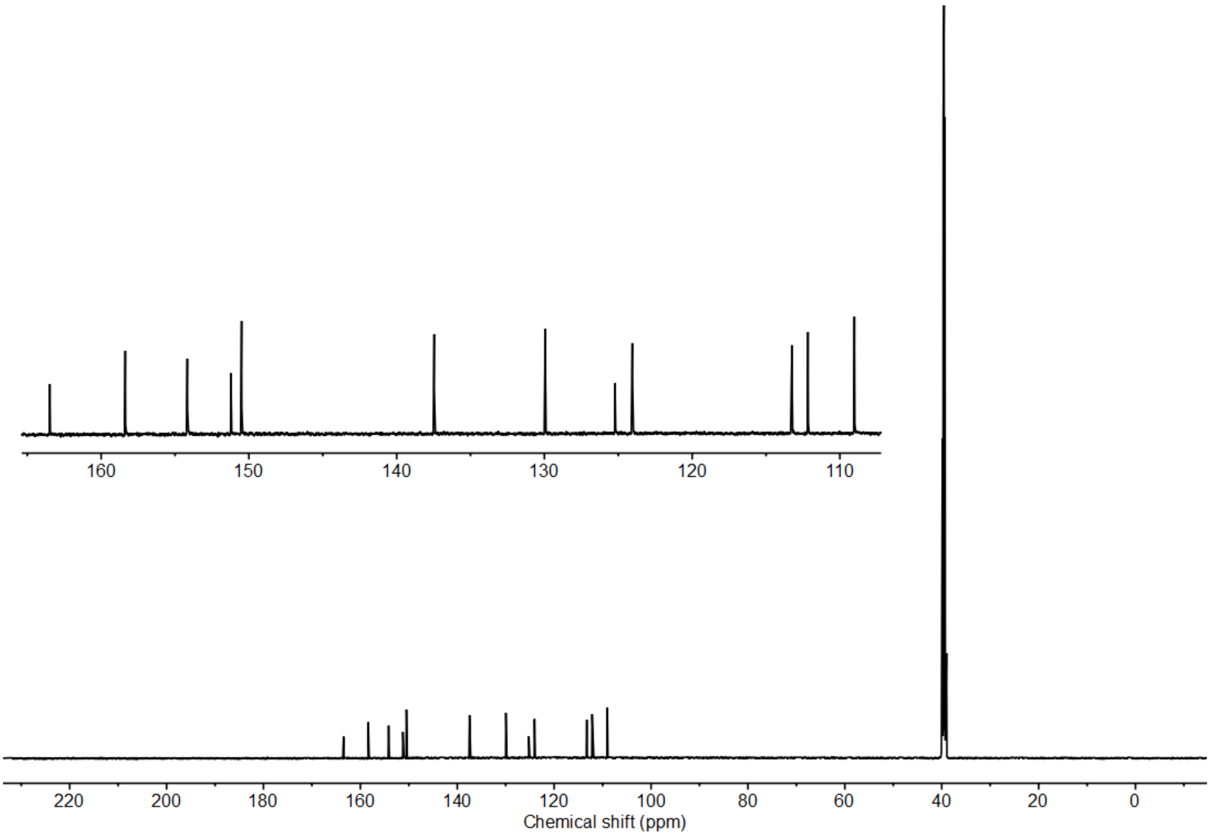


**Figure S148 $^{13}C$ NMR (126 MHz, $d_6$-DMSO) of S3.**

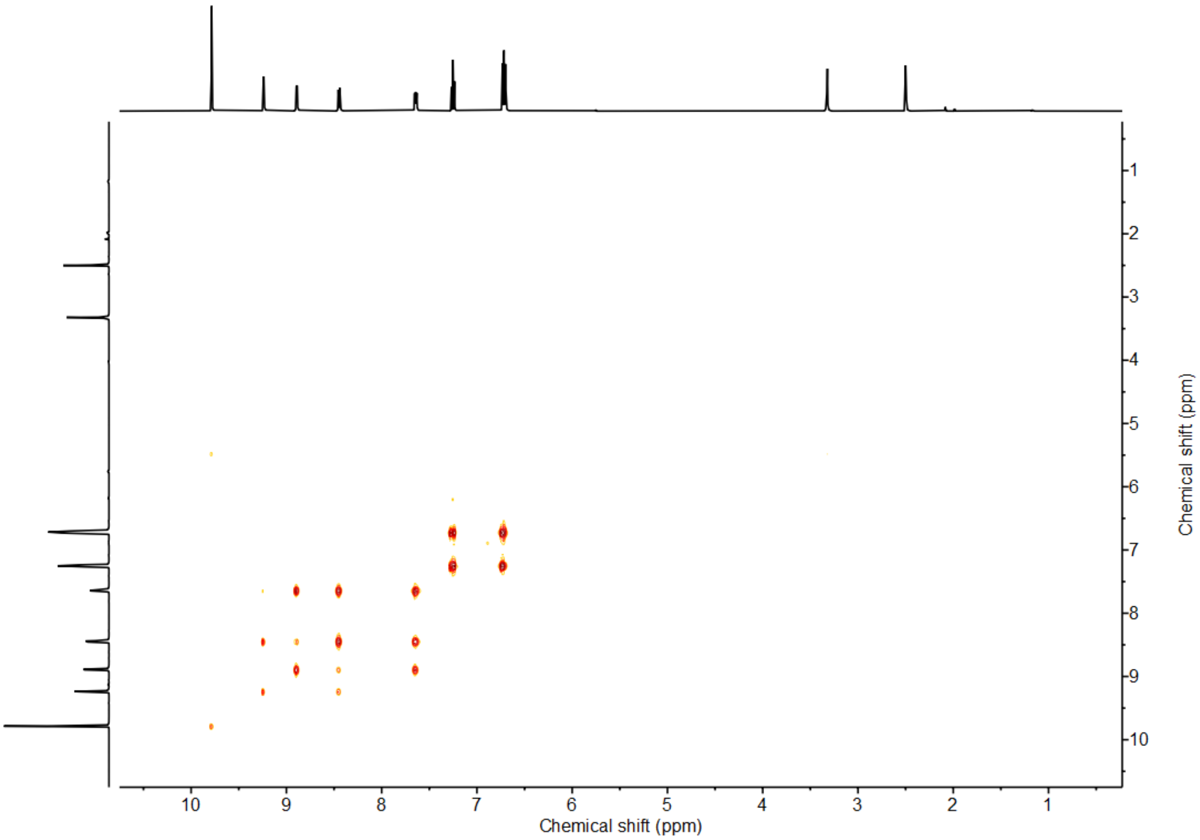


**Figure S149 COSY ($d_6$-DMSO) of S3.**

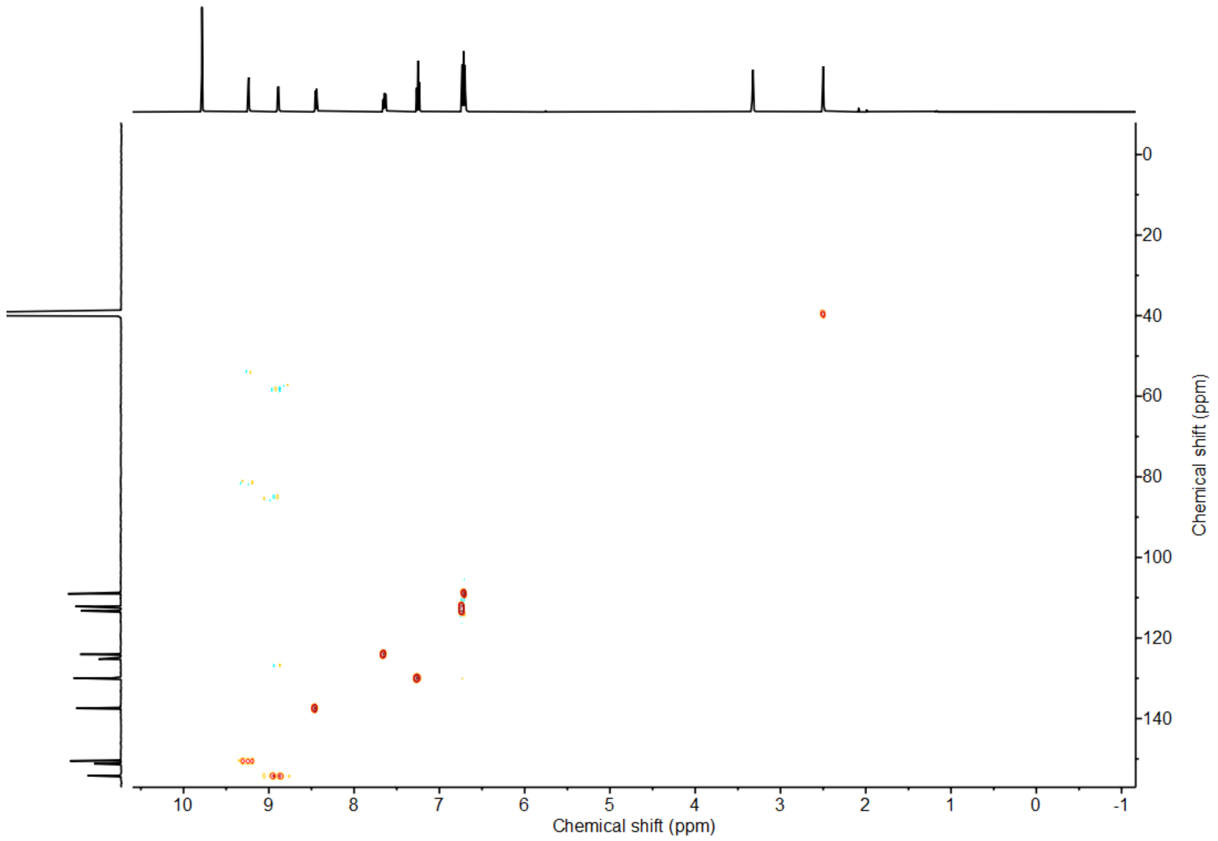


**Figure S150 HSQC ($d_6$-DMSO) of S3.**

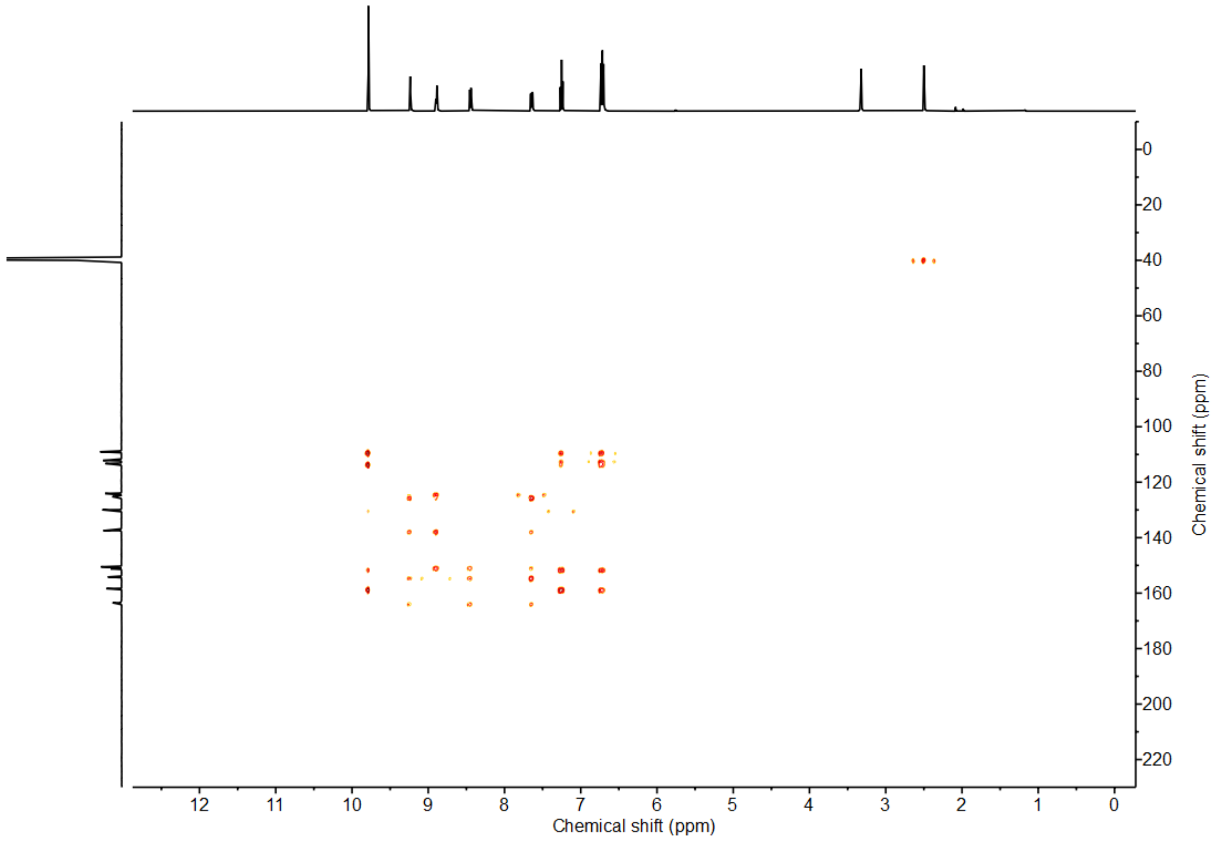


**Figure S151 HMBC ($d_6$-DMSO) of S3.**

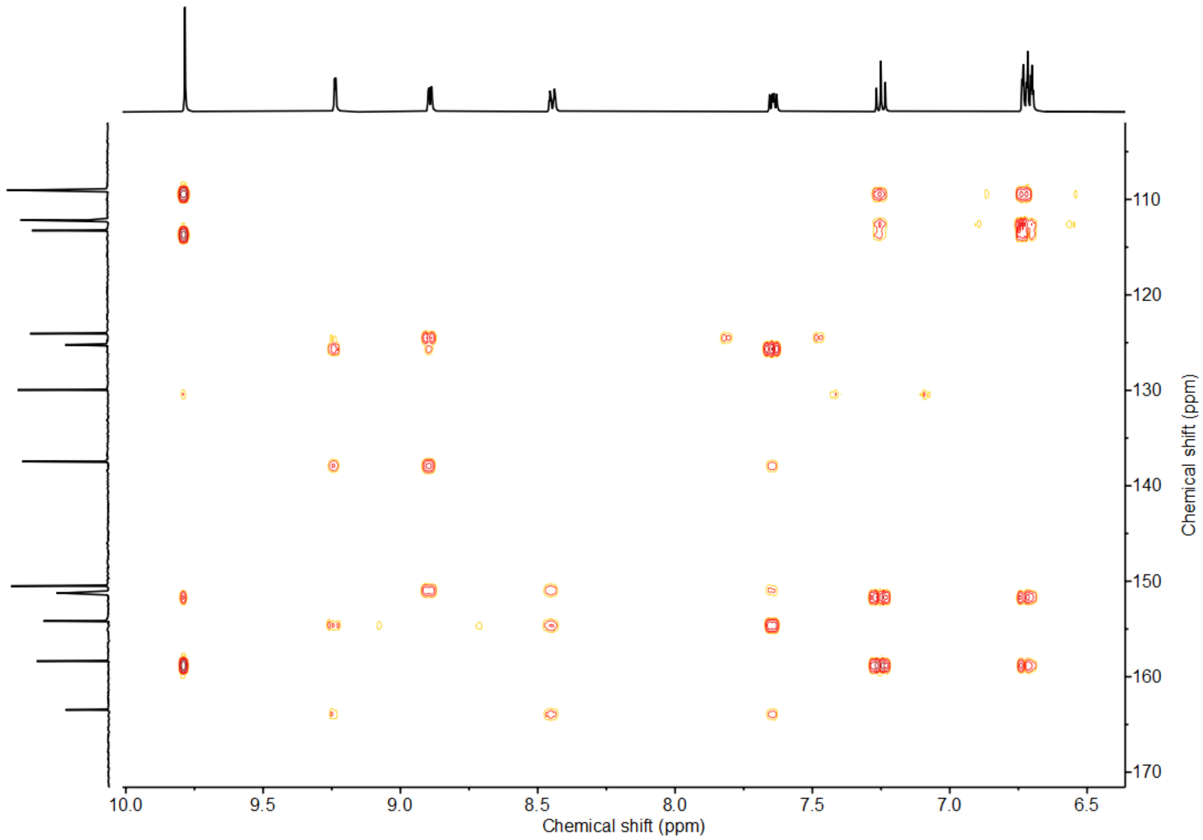


**Figure S152 Partial HMBC ($d_6$-DMSO) of S3.**

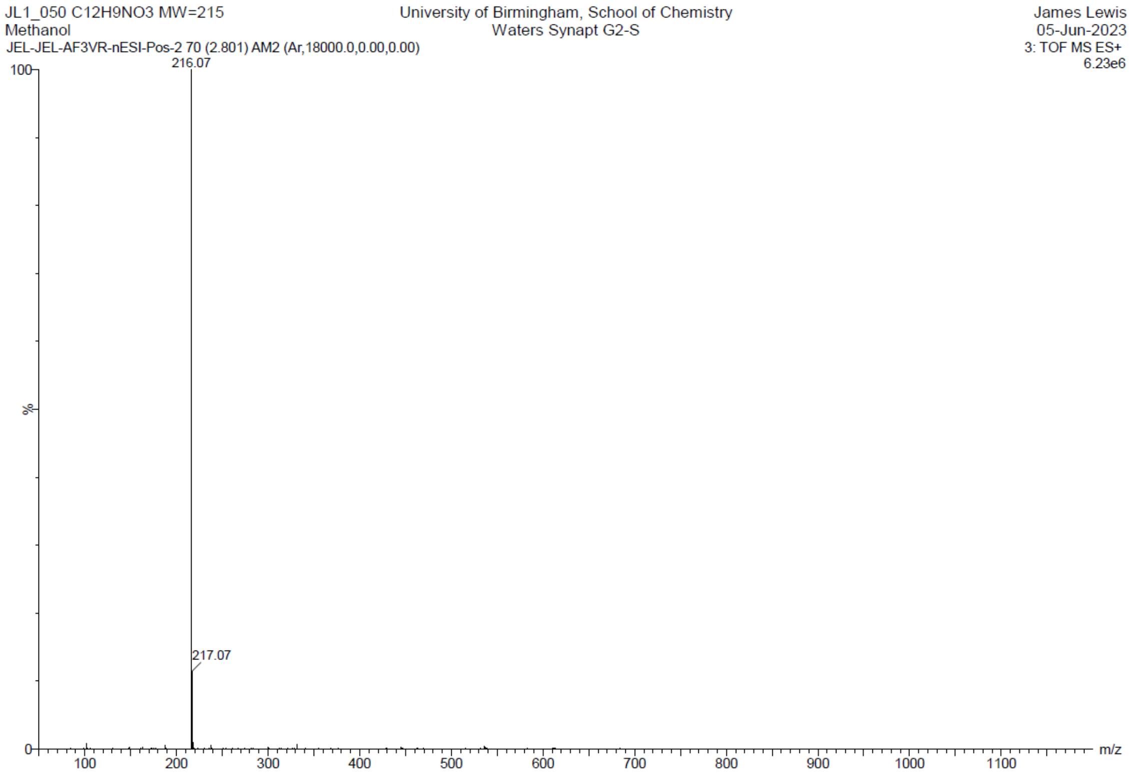


**Figure S153 ESI-MS of S3.**

## S2.19 Synthesis of $L3^P$

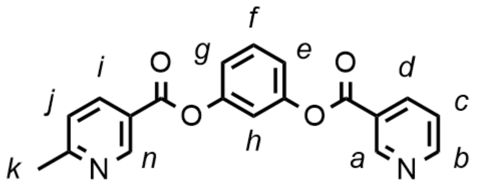


To **S3** (0.108 g, 0.50 mmol, 1 eq.), 6-methylnicotinic acid (0.075 g, 0.55 mmol, 1.1 eq.) and DMAP (0.006 g, 0.05 mmol, 0.1 eq.) in $CHCl_3$ (5 mL) at 0 °C was added EDCI (0.115 g, 0.60 mmol, 1.2 eq.) carefully as a solid. The reaction mixture was stirred, allowing to warm to rt, for 20 h. Sat. aq. $NaHCO_3$ (20 mL) was added, and the aqueous phase extracted with $CH_2Cl_2$ (3 × 15 mL). The combined organic phases were dried ($MgSO_4$) and the solvent removed *in vacuo*. Following purification by column chromatography on silica gel (2:3 EtOAc/$CH_2Cl_2$) the product was obtained as a white solid (0.161 g, 96%).

**$^1$H NMR** (500 MHz, $CDCl_3$) δ: 9.39 (d, $J$ = 2.2 Hz, 1H, $H_a$), 9.27 (d, $J$ = 2.3 Hz, 1H, $H_n$), 8.87 (dd, $J$ = 4.9, 1.8 Hz, 1H, $H_b$), 8.45 (app. dt, $J$ = 8.0, 2.0 Hz, 1H, $H_d$), 8.33 (dd, $J$ = 8.1, 2.2 Hz, 1H, $H_i$), 7.51 (app. t, $J$ = 8.1 Hz, 1H, $H_f$), 7.48 (ddd, $J$ = 7.9, 5.0, 0.9 Hz, 1H, $H_c$), 7.33 (d, $J$ = 8.1 Hz, 1H, $H_j$), 7.24-7.18 (m, 3H, $H_e$, $H_g$, $H_h$), 2.69 (s, 3H, $H_k$).

**$^{13}$C NMR** (126 MHz, $CDCl_3$) δ: 164.2, 163.8, 163.6, 154.3 ($C_b$), 151.6, 151.2, 151.1, 151.0, 138.1 ($C_i$), 137.8 ($C_d$), 130.2 ($C_f$), 125.4, 123.7 ($C_c$), 123.4 ($C_j$), 122.8, 119.7 ($C_e$/$C_g$/$C_h$), 119.5 ($C_e$/$C_g$/$C_h$), 115.8 ($C_e$/$C_g$/$C_h$), 25.0 ($C_k$).

**HR-ESI-MS** *m/z* = 335.1040 $[M+H]^+$ calc. 335.1032.

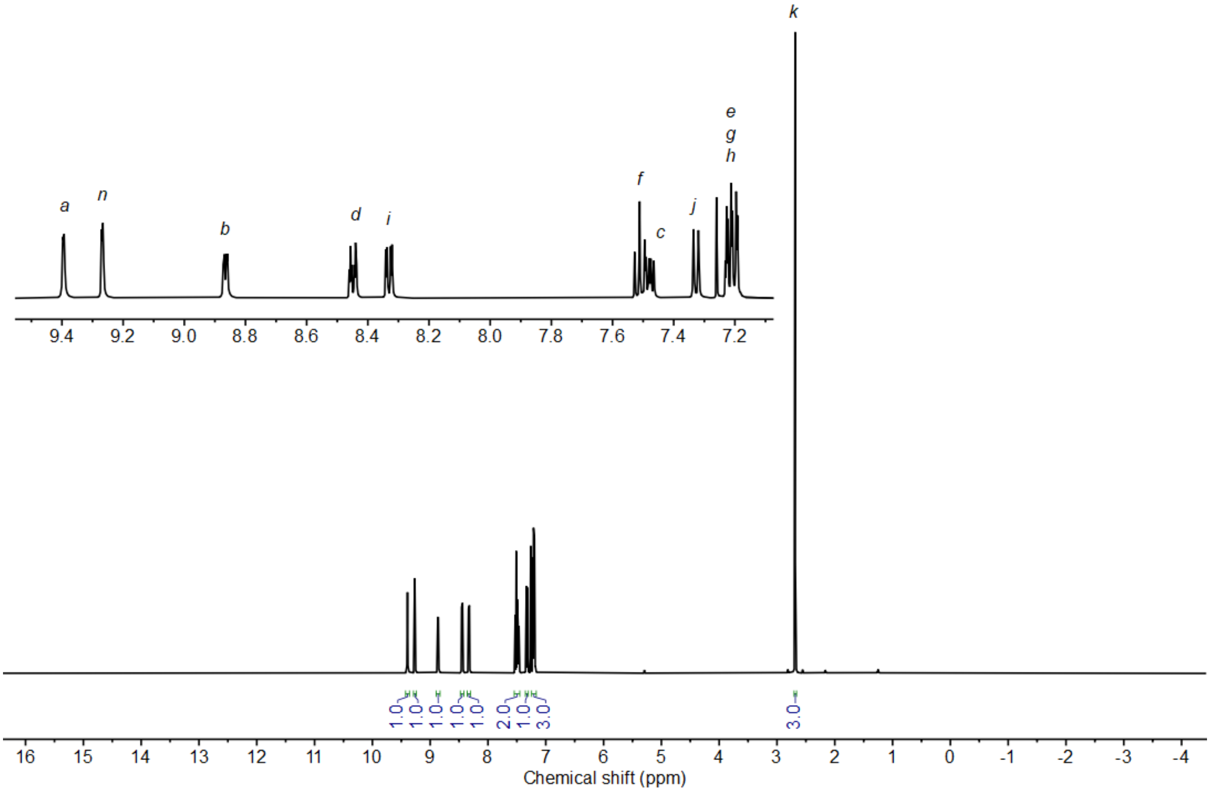


**Figure S154 $^1$H NMR (500 MHz, $CDCl_3$) of $L3^P$.**

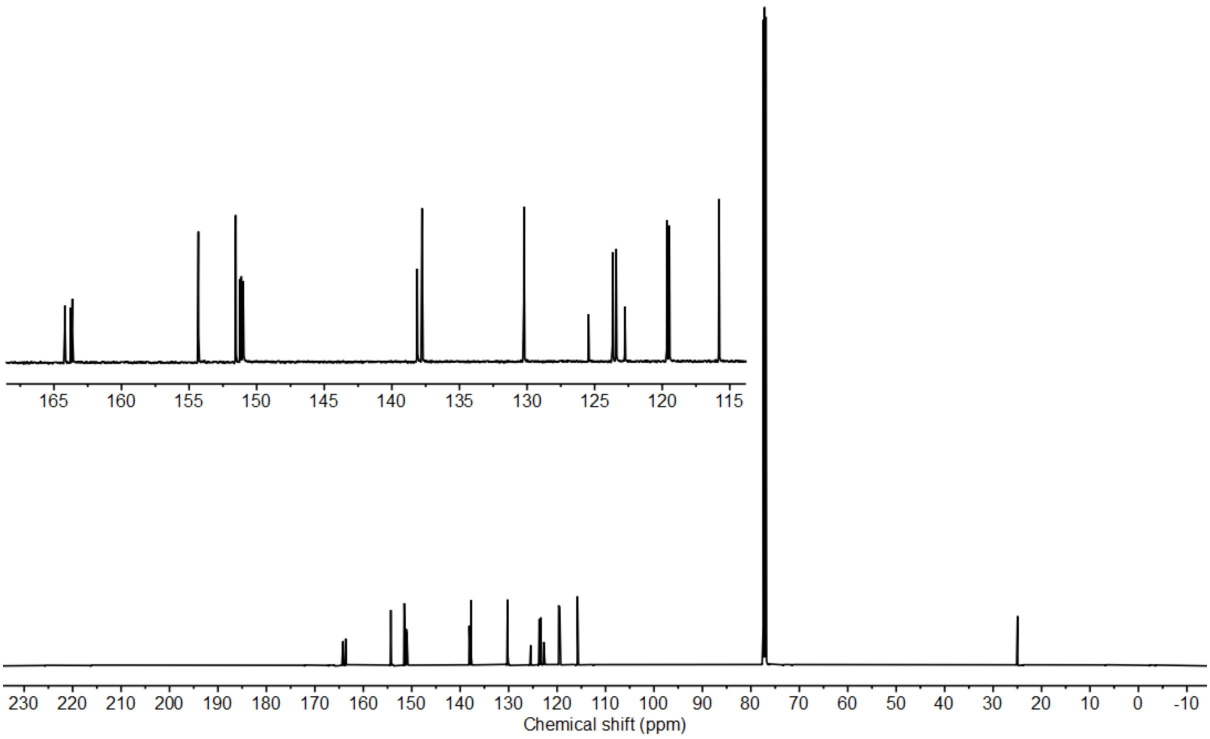


**Figure S155 $^{13}C$ NMR (126 MHz, $CDCl_3$) of L3$^{P}$.**

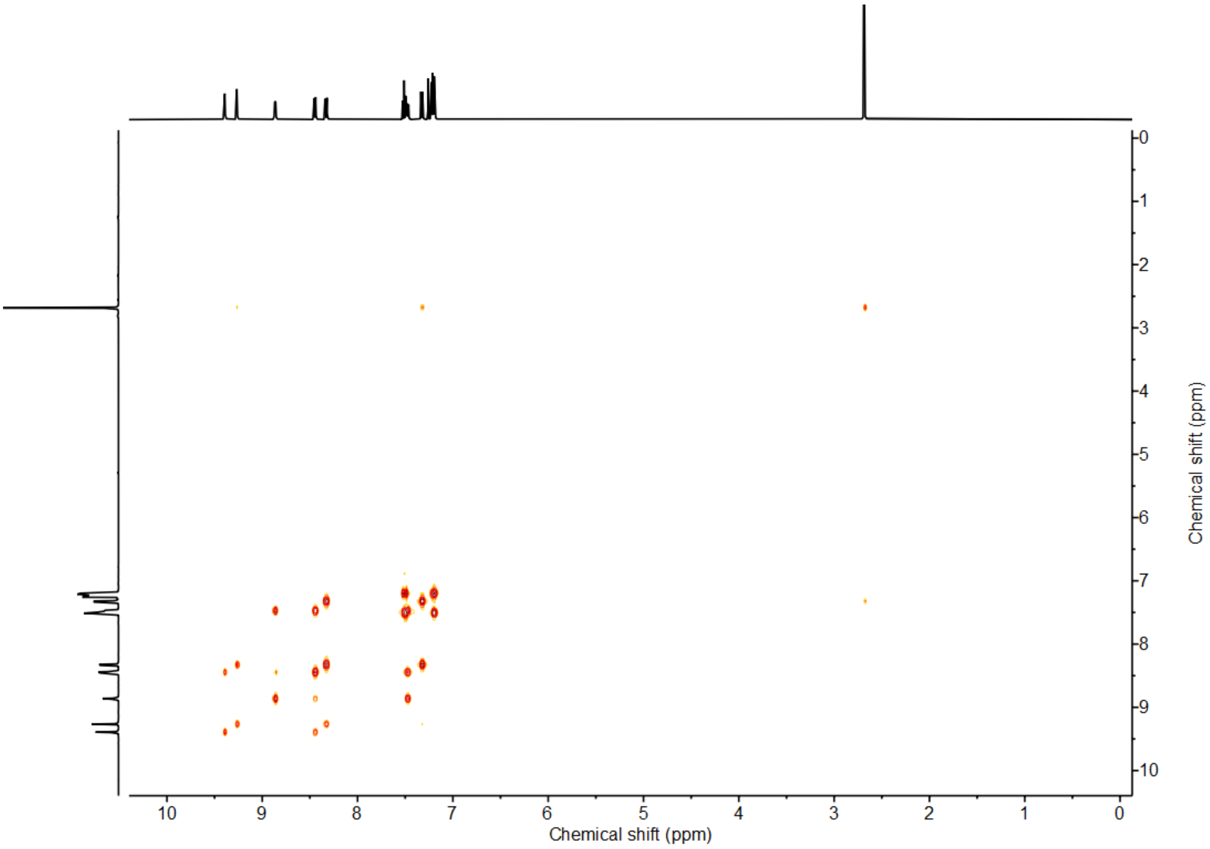


**Figure S156 COSY ($CDCl_3$) of L3$^{P}$.**

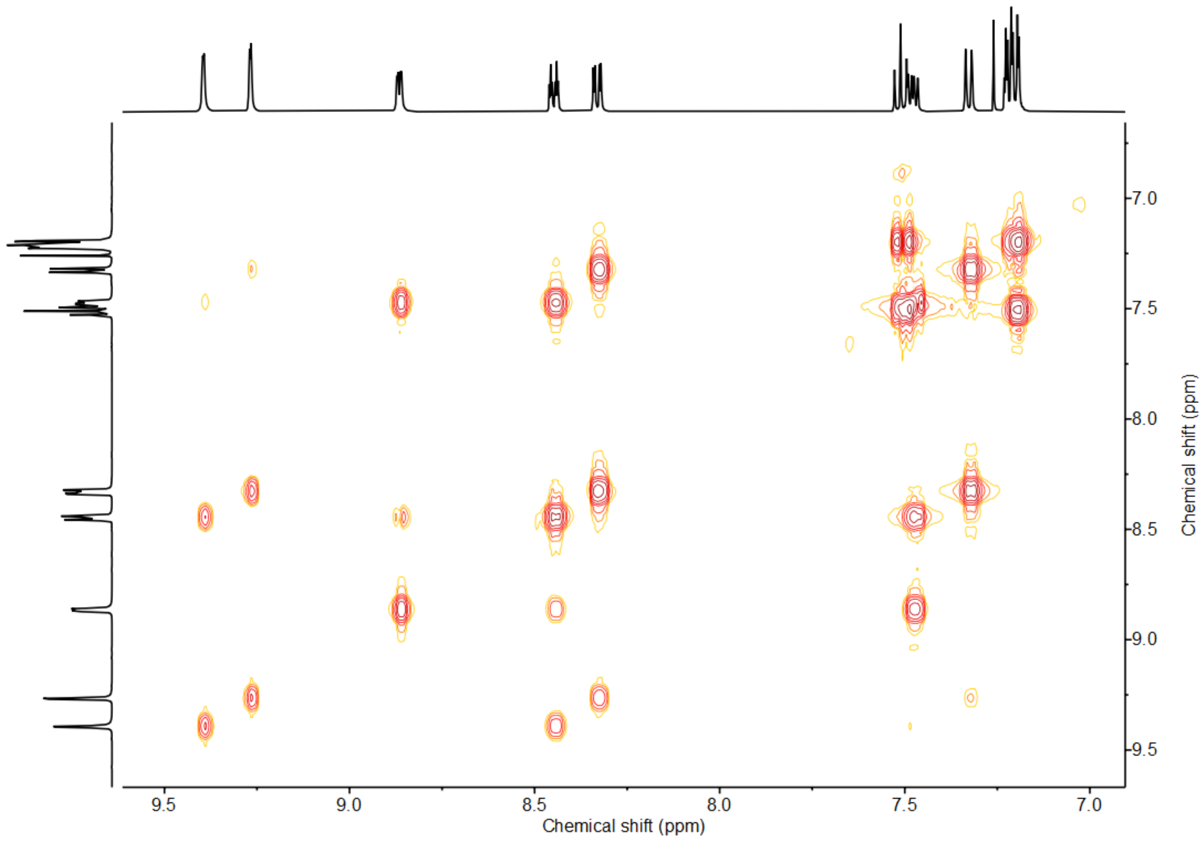


**Figure S157 Partial COSY ($CDCl_3$) of L3[P].**

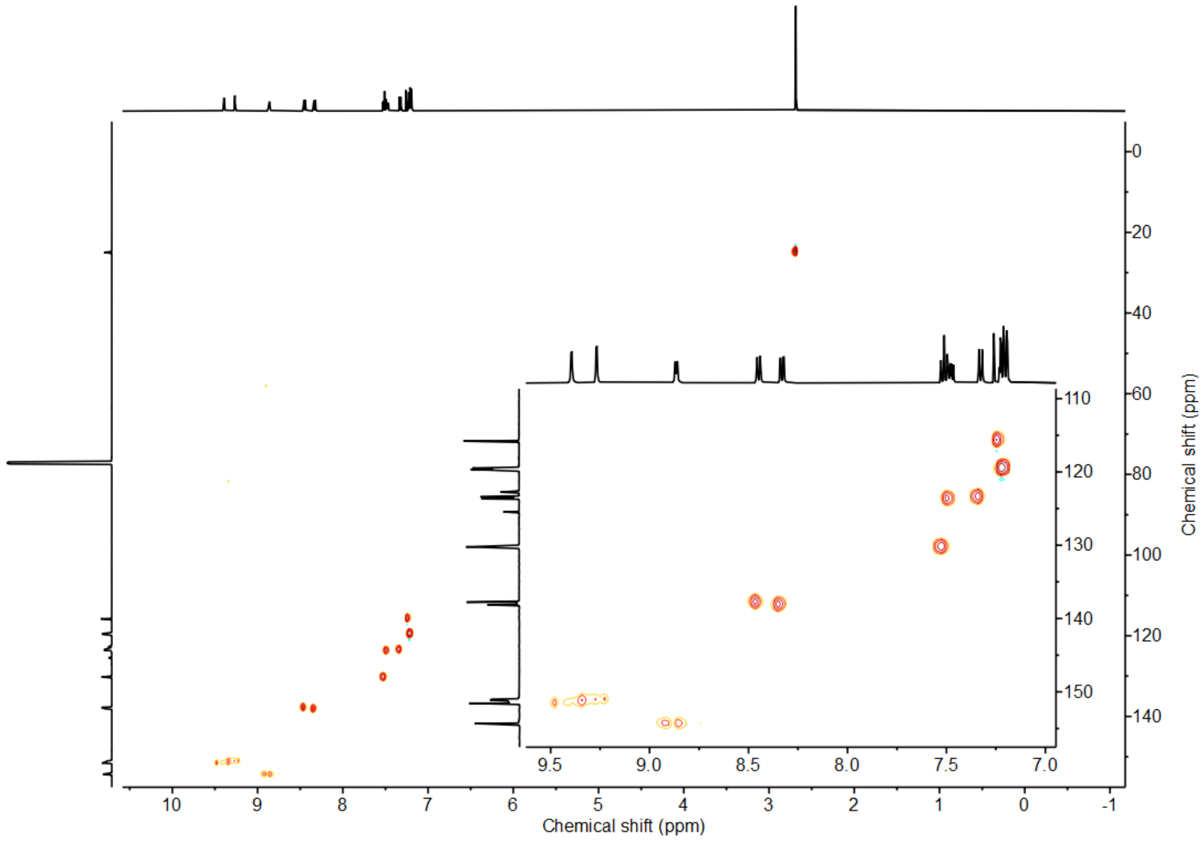


**Figure S158 HSQC ($CDCl_3$) of L3[P].**

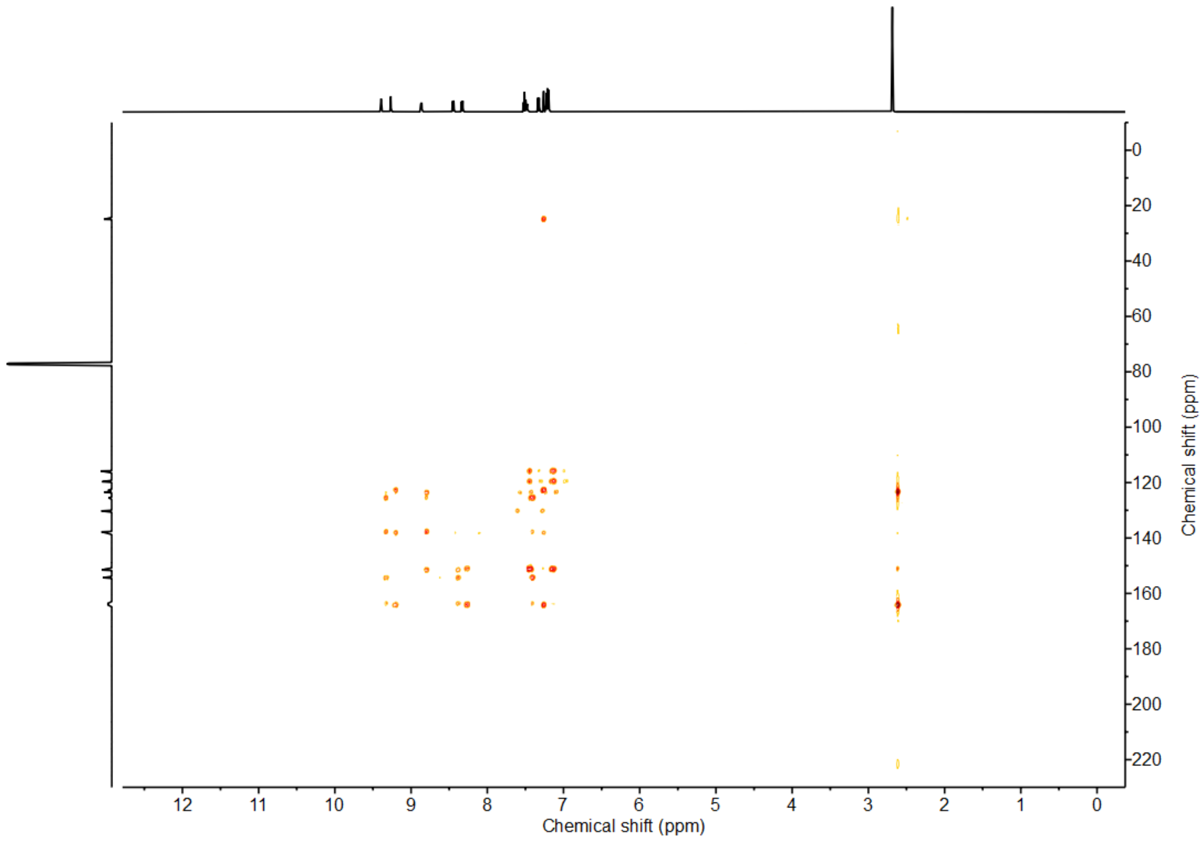


**Figure S159 HMBC ($CDCl_3$) of L3$^P$.**

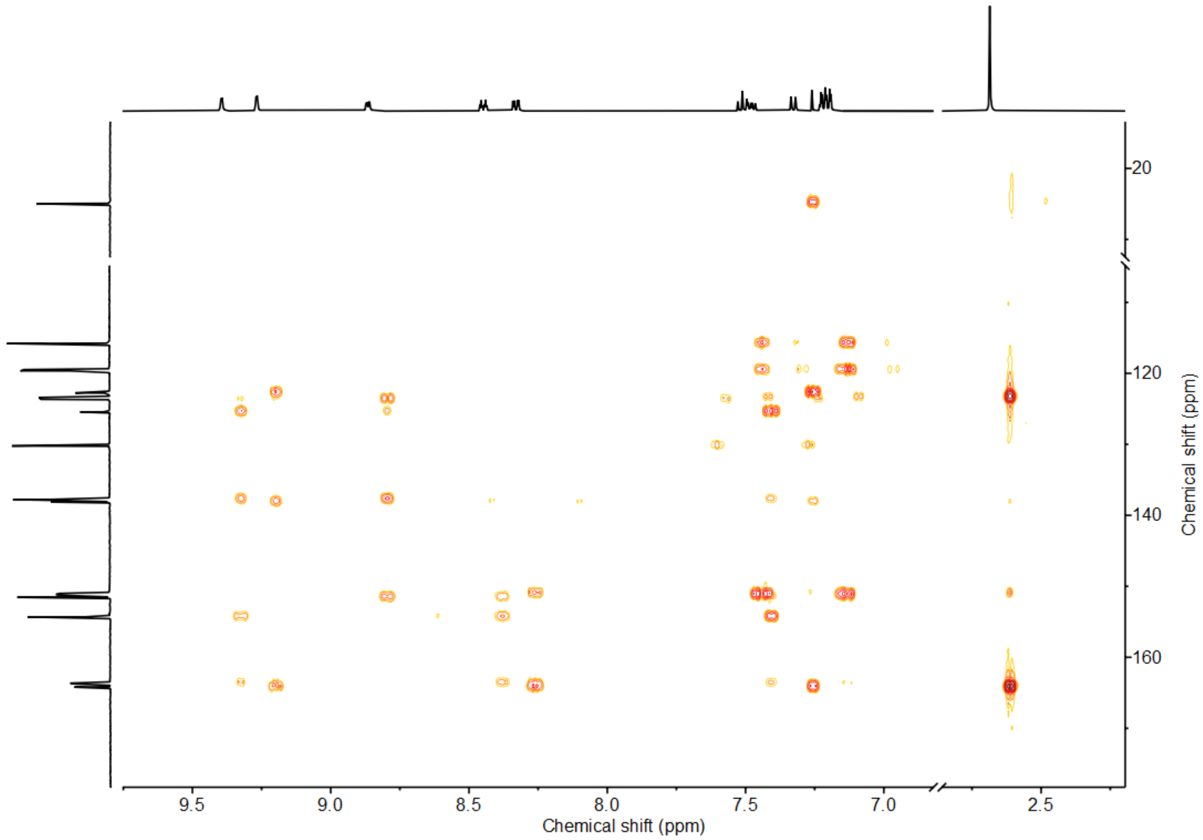


**Figure S160 Partial HMBC ($CDCl_3$) of L3$^P$.**

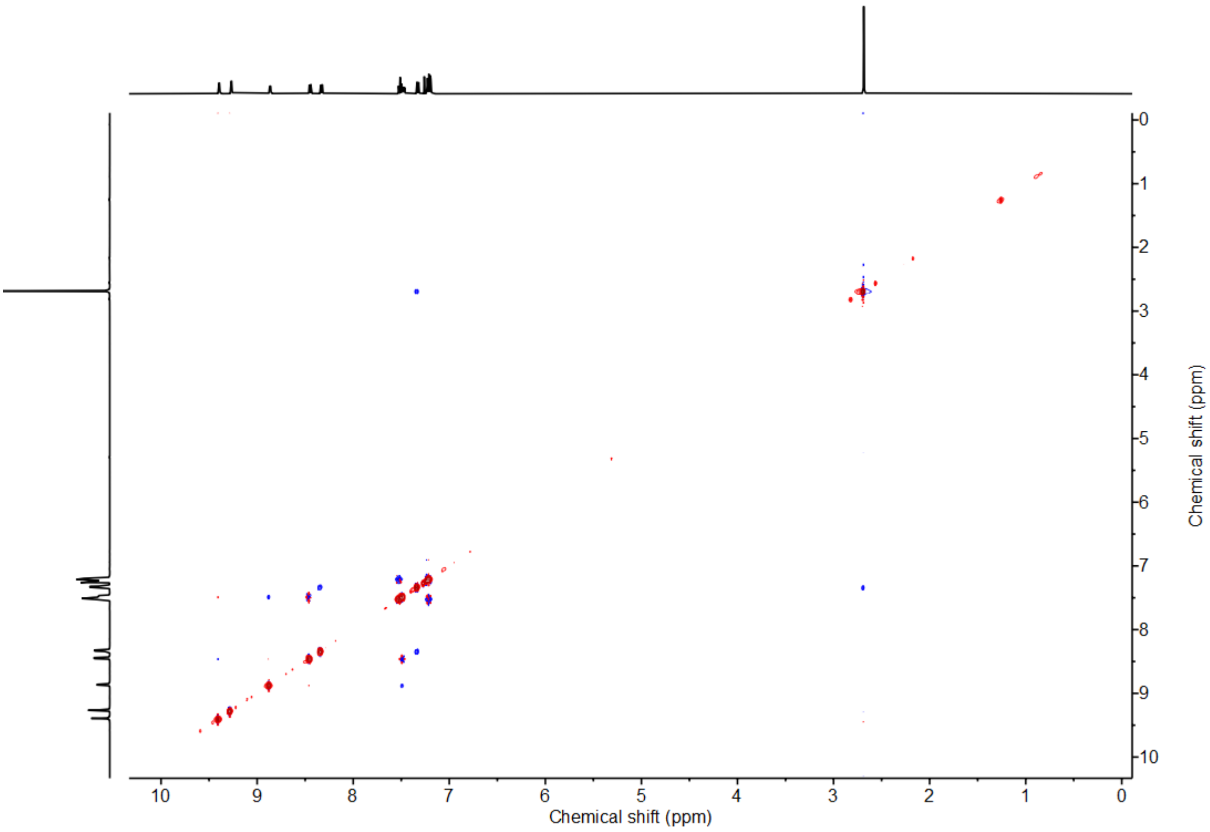


**Figure S161 NOESY ($CDCl_3$) of L3[P].**

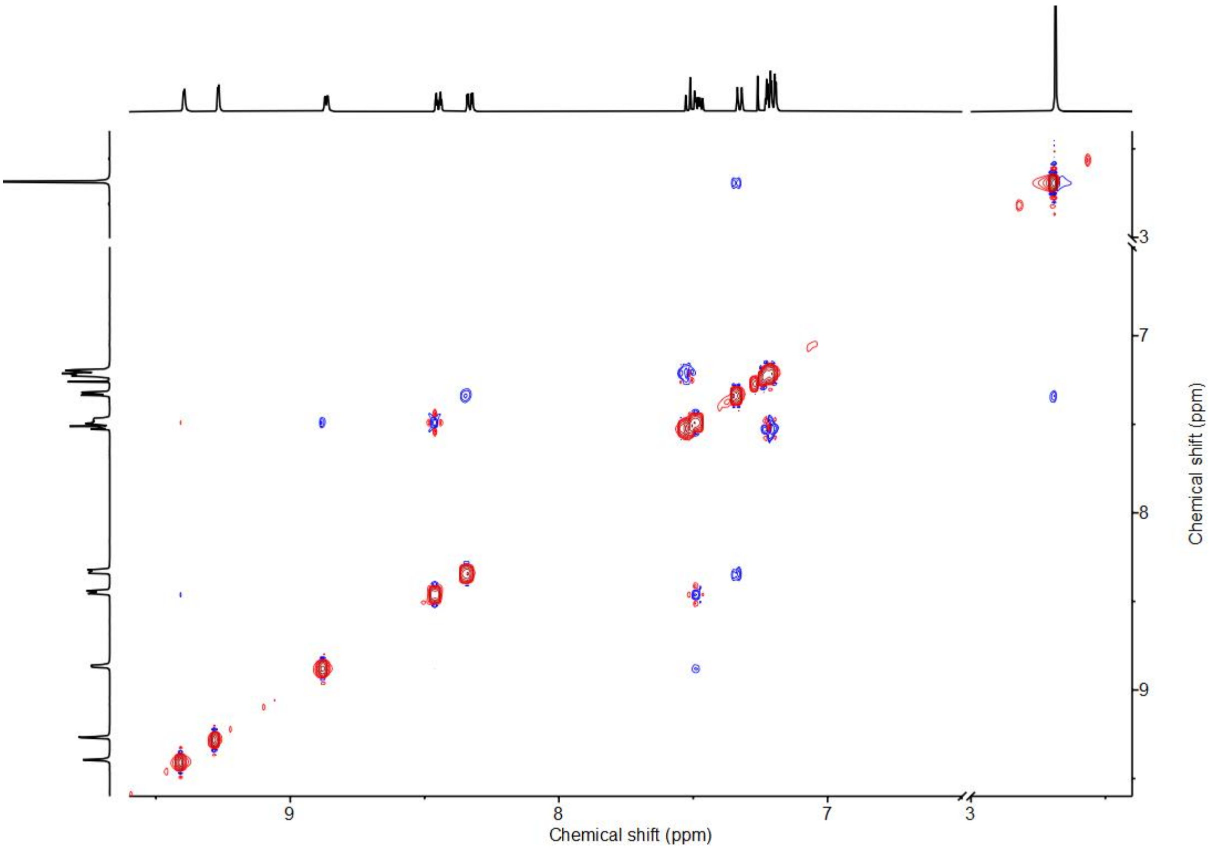


**Figure S162 Partial NOESY ($CDCl_3$) of L3[P].**

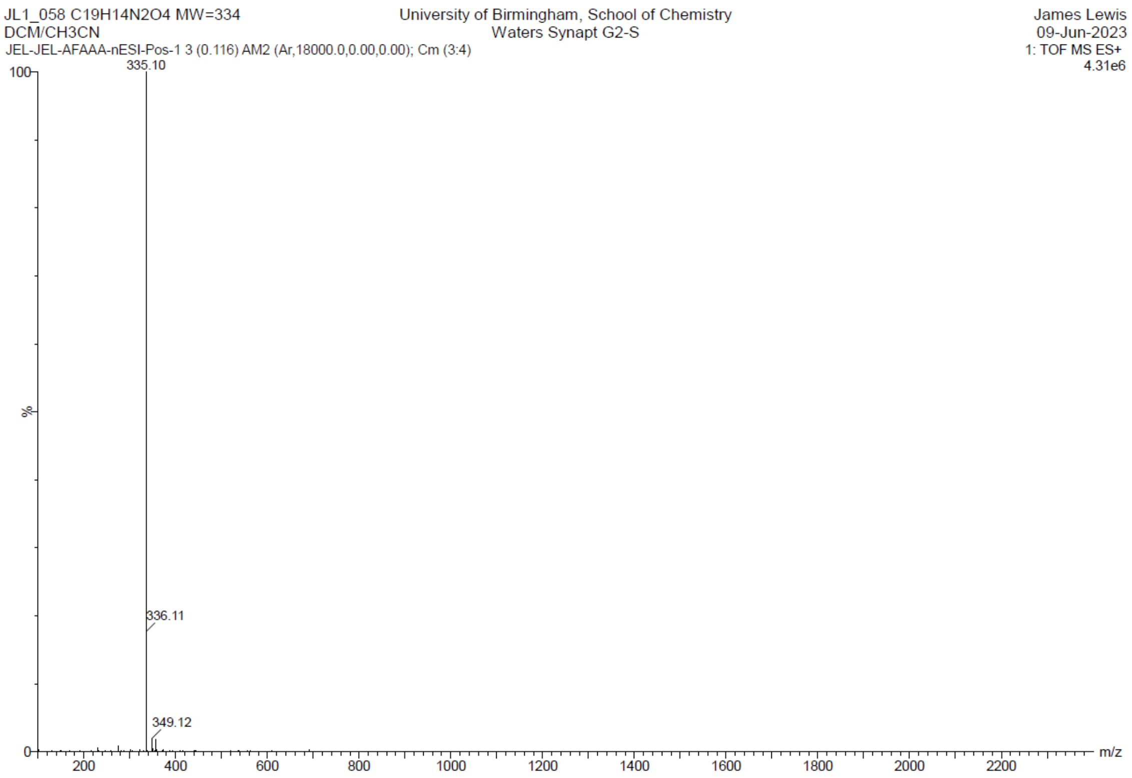


**Figure S163 ESI-MS of L3$^{P}$.**

## S2.20 Synthesis of *trans*-C3$^{P}$

**L3**$^{P}$ (10.0 mg, 30 µmol, 1 eq.) and $[Pd(CH_3CN)_4](BF_4)_2$ (7.3 mg, 16.5 µmol, 0.55 eq.) were sonicated in $CD_3CN$ (0.75 mL) until a homogenous solution was obtained. This was stood at 50 °C for 18 h until no further changes were observed by $^{1}$H NMR.

NMR data for dominant species:

**$^{1}$H NMR** (500 MHz, $CD_3CN$) δ: 10.18 (d, $J$ = 2.0 Hz, 6H, $H_k$), 9.96 (d, $J$ = 1.8 Hz, 6H, $H_a$), 9.04 (m, 6H, $H_b$), 8.61 (dt, $J$ = 8.1, 1.6 Hz, 6H, $H_d$), 8.52 (dd, $J$ = 8.3, 1.9 Hz, 6H, $H_i$), 7.79 (dd, $J$ = 8.1, 5.8 Hz, 6H, $H_c$), 7.73 (d, $J$ = 8.3 Hz, 6H, $H_j$), 7.51 (app. t, $J$ = 8.2 Hz, 6H, $H_f$), 7.27 (app. t, $J$ = 2.2 Hz, 6H, $H_h$), 7.20-7.16 (m, 12H, $H_e$, $H_g$), 3.75 (s, 18H, $H_l$).

**$^{13}$C NMR** (126 MHz, $CD_3CN$) δ: 166.3, 162.7, 162.4, 156.6 ($C_b$), 153.3 ($C_a$/$C_k$), 153.2 ($C_a$/$C_k$), 151.9, 151.8, 143.0 ($C_d$), 142.2 ($C_i$), 131.7 ($C_f$), 130.7, 129.4 ($C_j$), 129.1 ($C_c$), 128.2, 121.2 ($C_e$/$C_g$), 121.0 ($C_e$/$C_g$), 117.7 ($C_h$), 27.1 ($C_l$).

**ESI-MS** *m/z* = 507.04 $\{[Pd_3(\mathbf{L3}^{P})_5]F_2\}^{4+}$ calc. 507.05; 523.07 $\{[Pd_2(\mathbf{L3}^{P})_4]F\}^{3+}$ calc. 523.06; 590.57 $\{[Pd_3(\mathbf{L3}^{P})_6]F_2\}^{4+}$ calc. 590.57; 627.05 $\{[Pd_2(\mathbf{L3}^{P})_3]F_2\}^{2+}$ calc. 627.05; 682.40 $\{[Pd_3(\mathbf{L3}^{P})_5]F_3\}^{3+}$ calc. 682.40; 828.11 $\{[Pd_2(\mathbf{L3}^{P})_4](BF_4)F\}^{2+}$ calc. 828.10; 839.11 $\{[Pd_3(\mathbf{L3}^{P})_6](BF_4)_2F\}^{3+}$ calc. 839.10; 1268.16 $\{[Pd_3(\mathbf{L3}^{P})_6](BF_4)_2F_2\}^{2+}$ calc. 1268.15; 1302.17 $\{[Pd_3(\mathbf{L3}^{P})_6](BF_4)_3F\}^{2+}$ calc. 1302.15; 1341.12 $\{[Pd_2(\mathbf{L3}^{P})_3](BF_4)F_2\}^{+}$ calc. 1341.10.

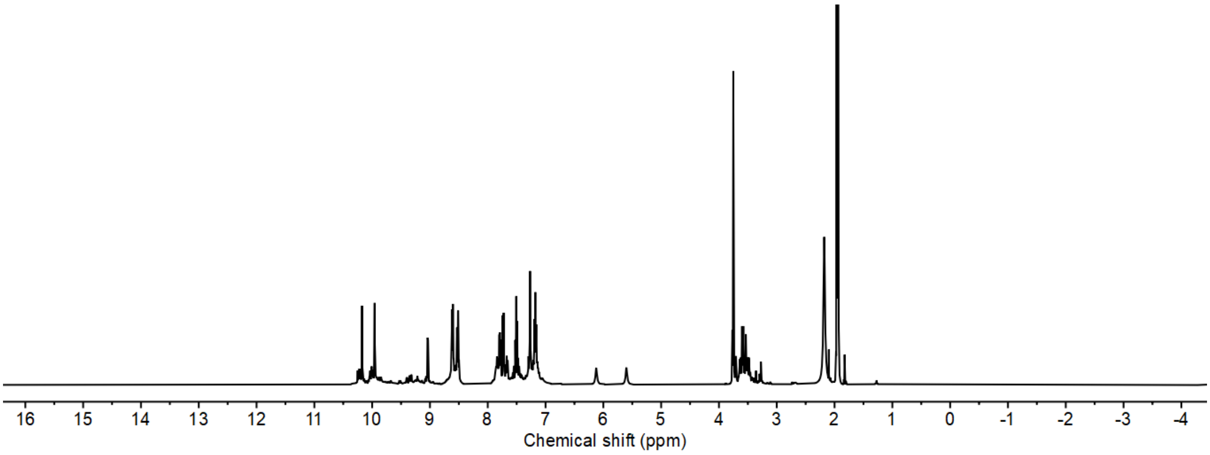


**Figure S164 $^{1}$H NMR (500 MHz, $CD_3CN$) of C3$^{P}$.**

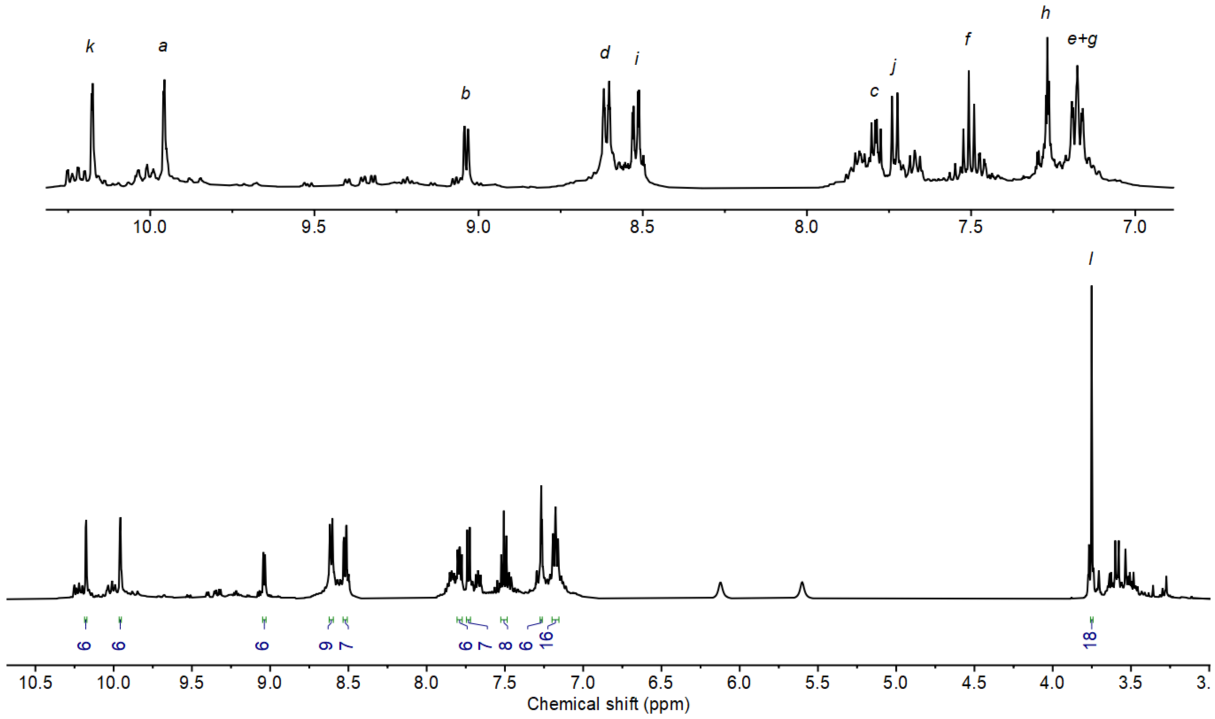


**Figure S165 Partial $^{1}H$ NMR (500 MHz, $CD_3CN$) of C3$^{P}$.**

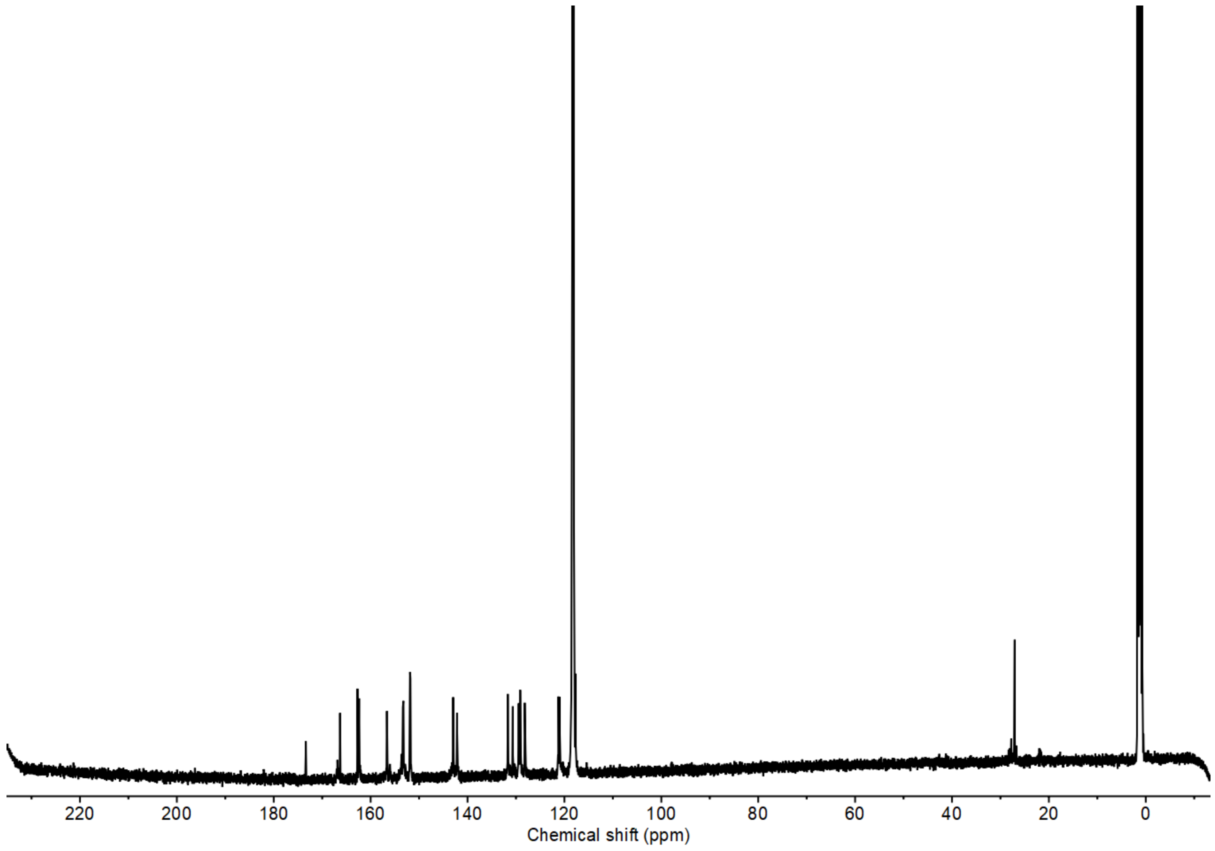


**Figure S166 $^{13}C$ NMR (126 MHz, $CD_3CN$) of C3$^{P}$.**

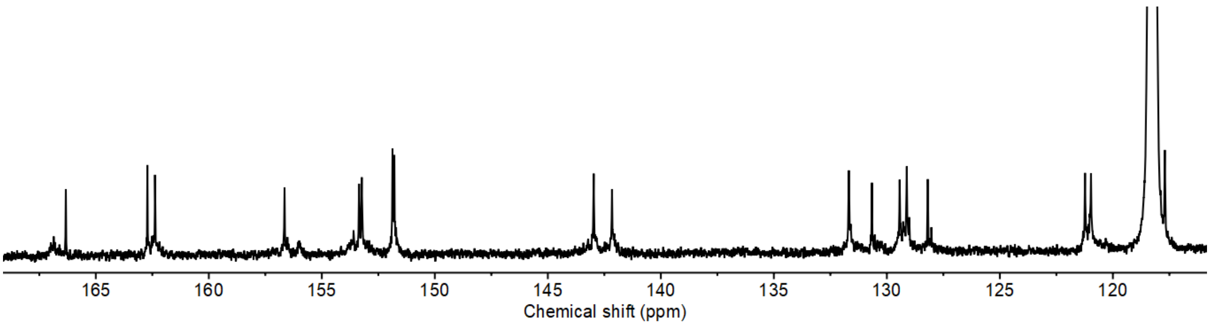


**Figure S167 Partial $^{13}C$ NMR (126 MHz, $CD_3CN$) of C3$^{P}$.**

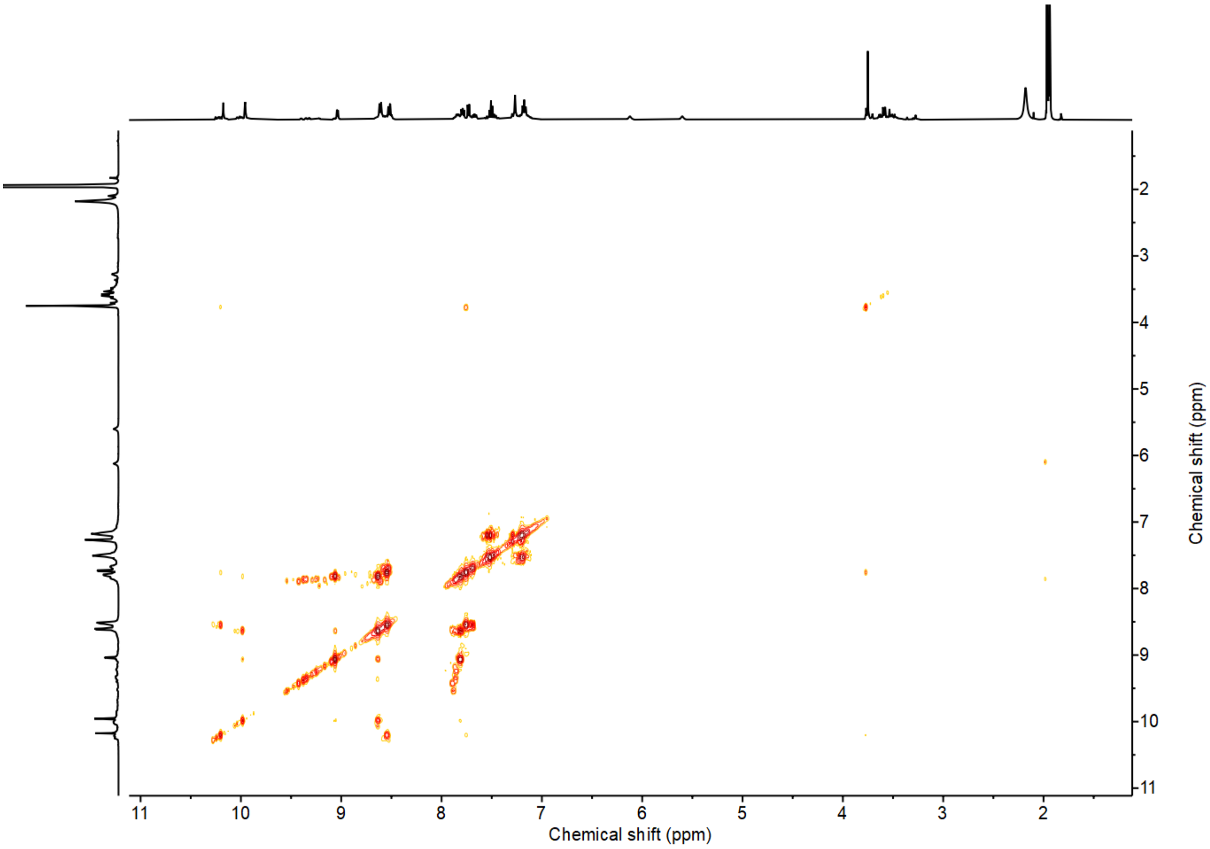


**Figure S168 COSY ($CD_3CN$) of C3$^{P}$.**

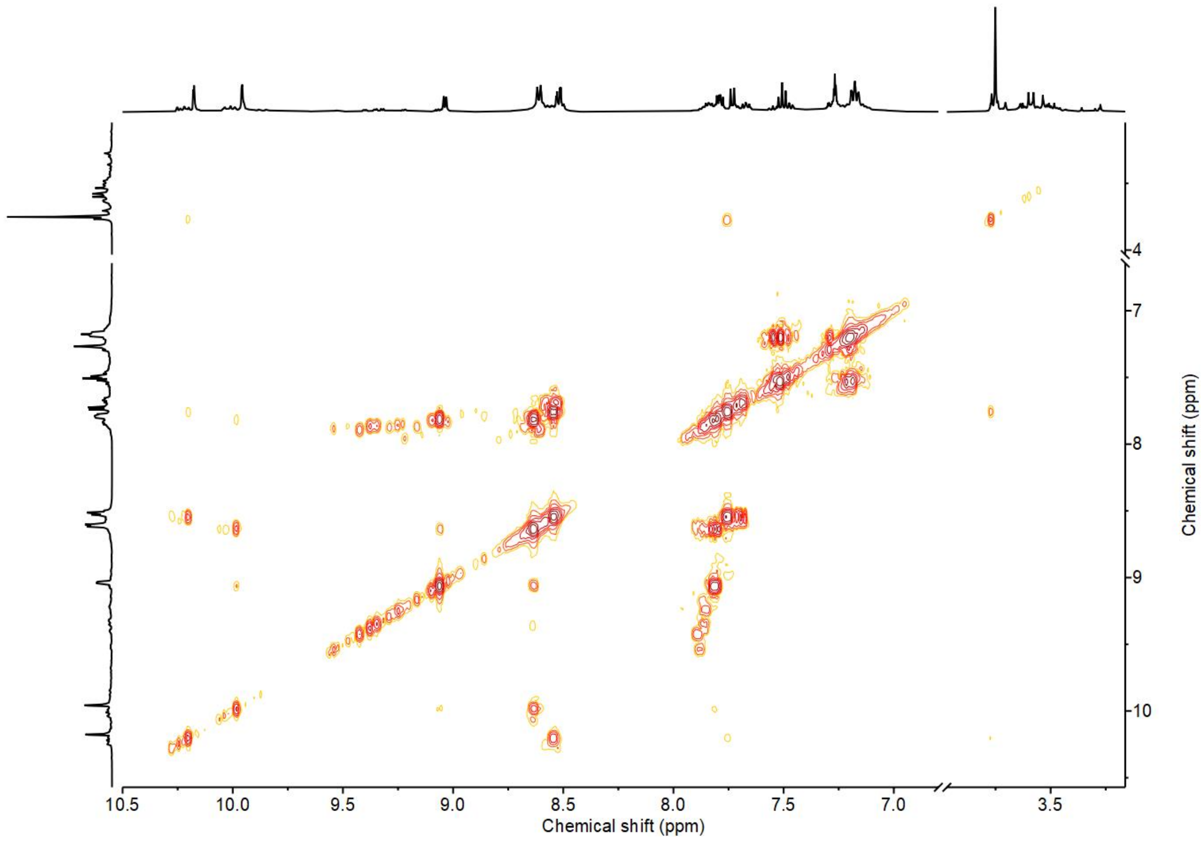


**Figure S169 Partial COSY ($CD_3CN$) of C3$^{P}$.**

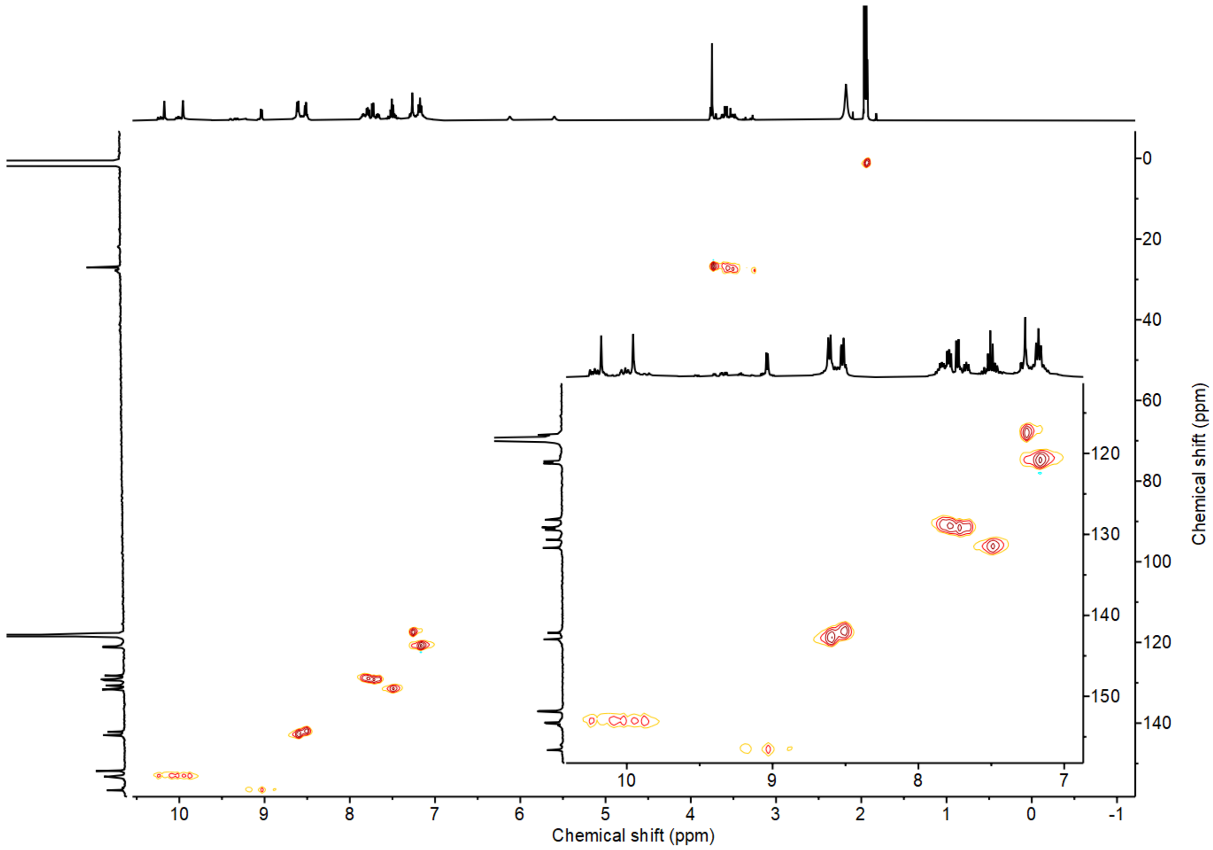


**Figure S170 HSQC ($CD_3CN$) of C3$^{P}$.**

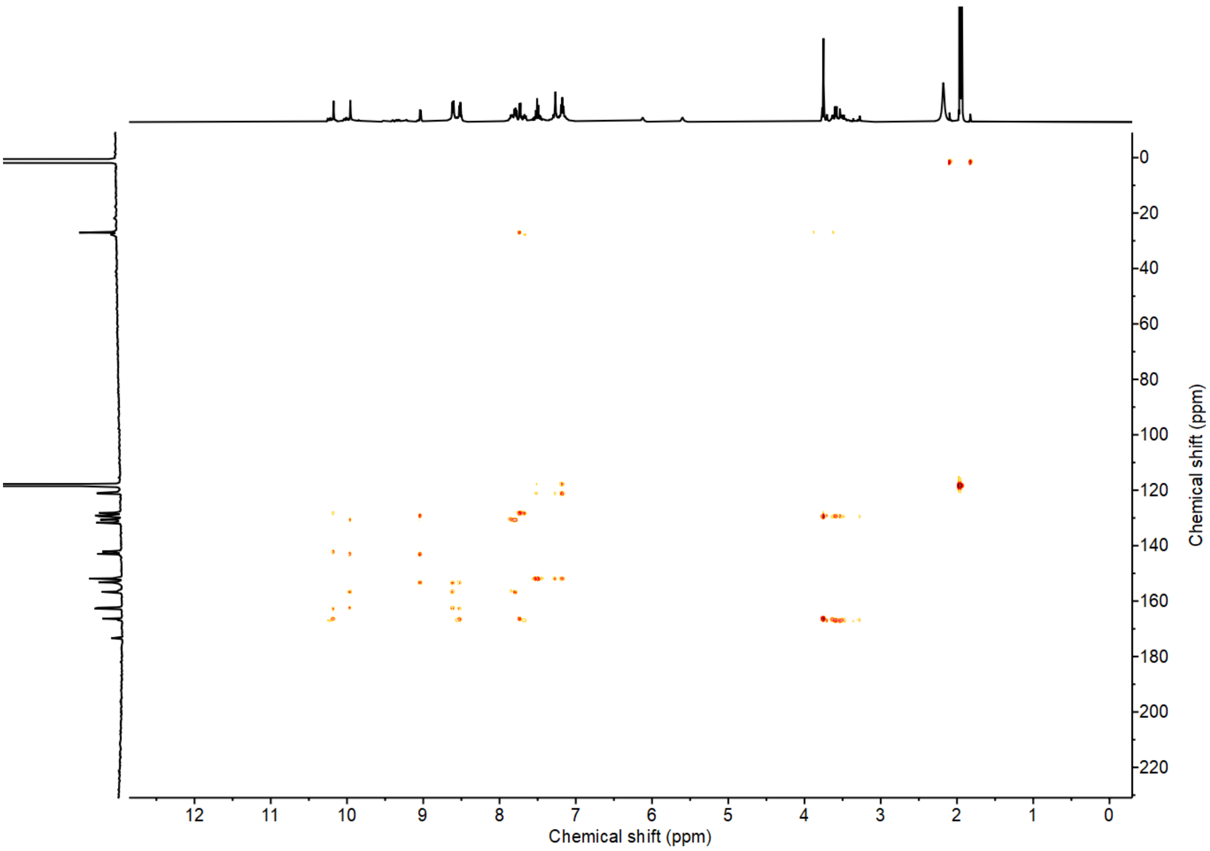


**Figure S171 HMBC ($CD_3CN$) of C3$^P$.**

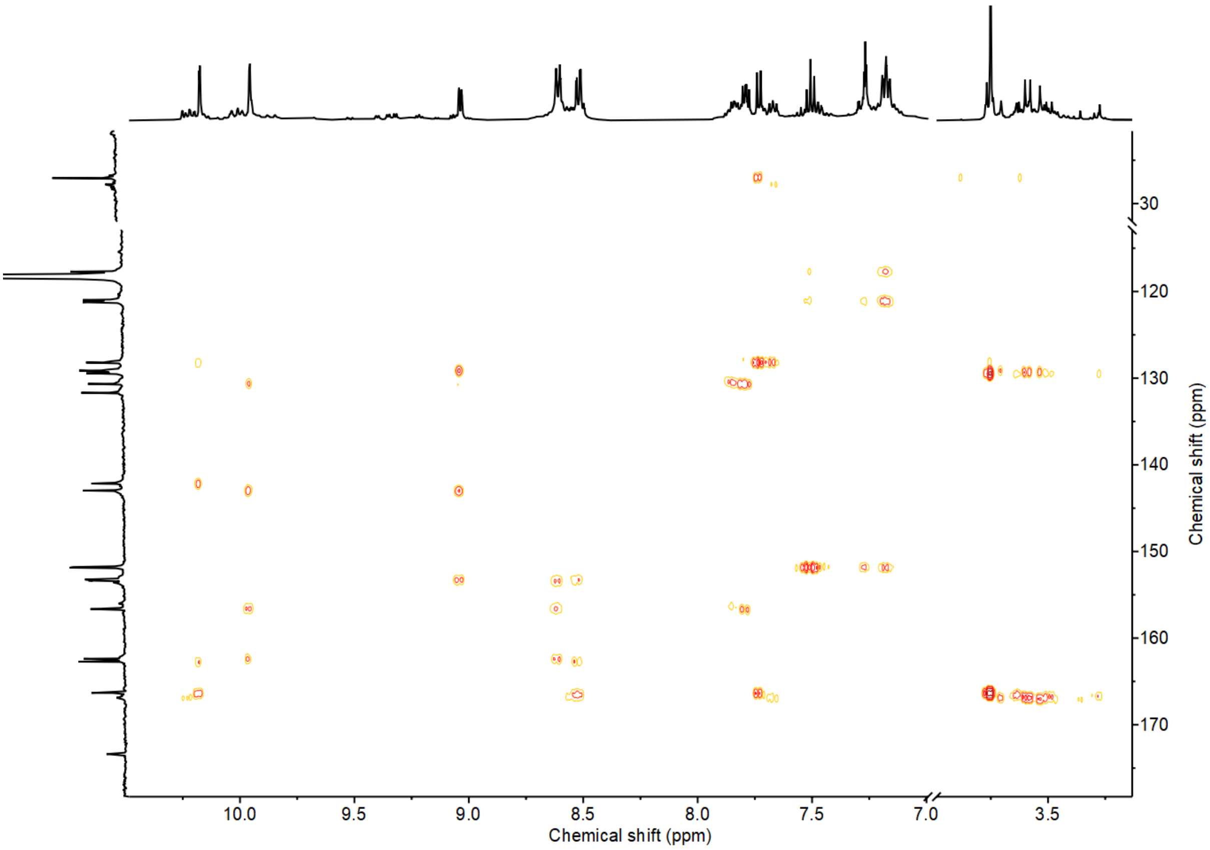


**Figure S172 Partial HMBC ($CD_3CN$) of C3$^P$.**

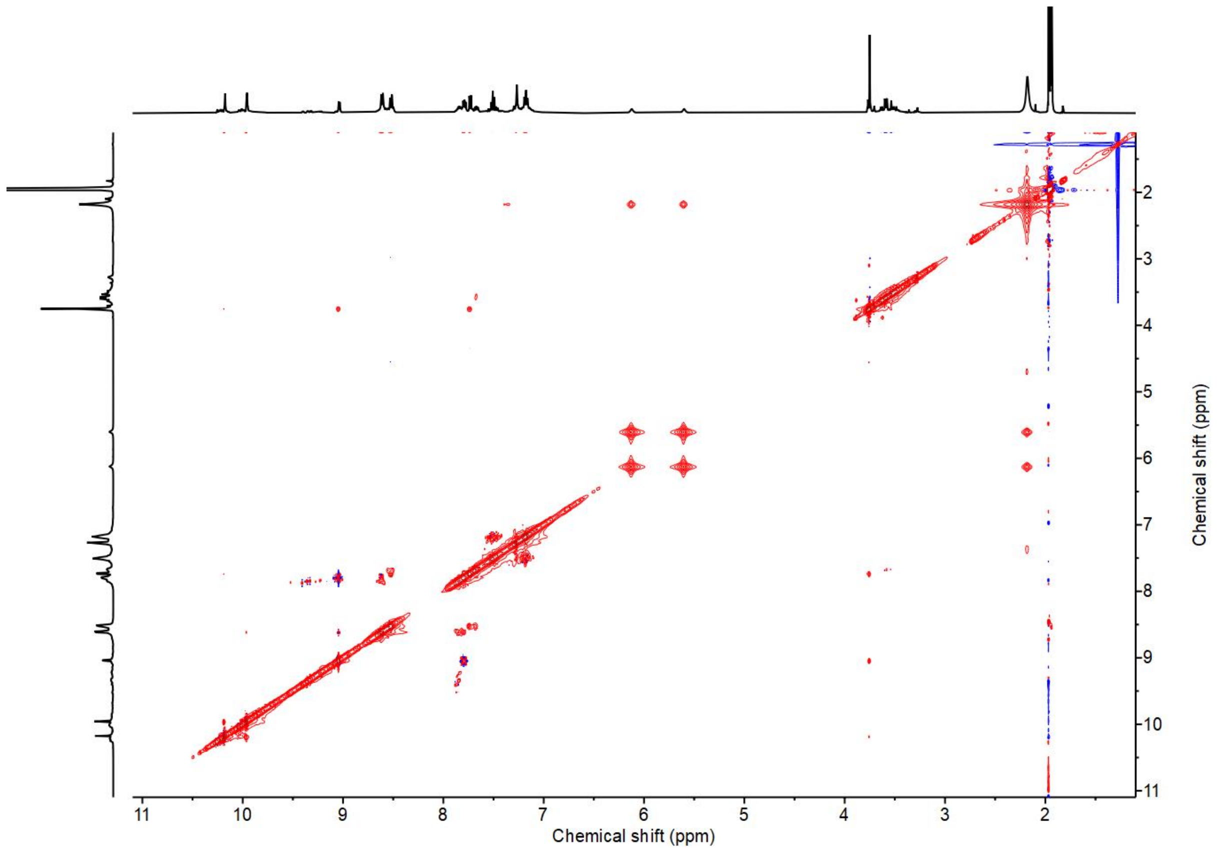


**Figure S173 NOESY ($CD_3CN$) of C3[P].**

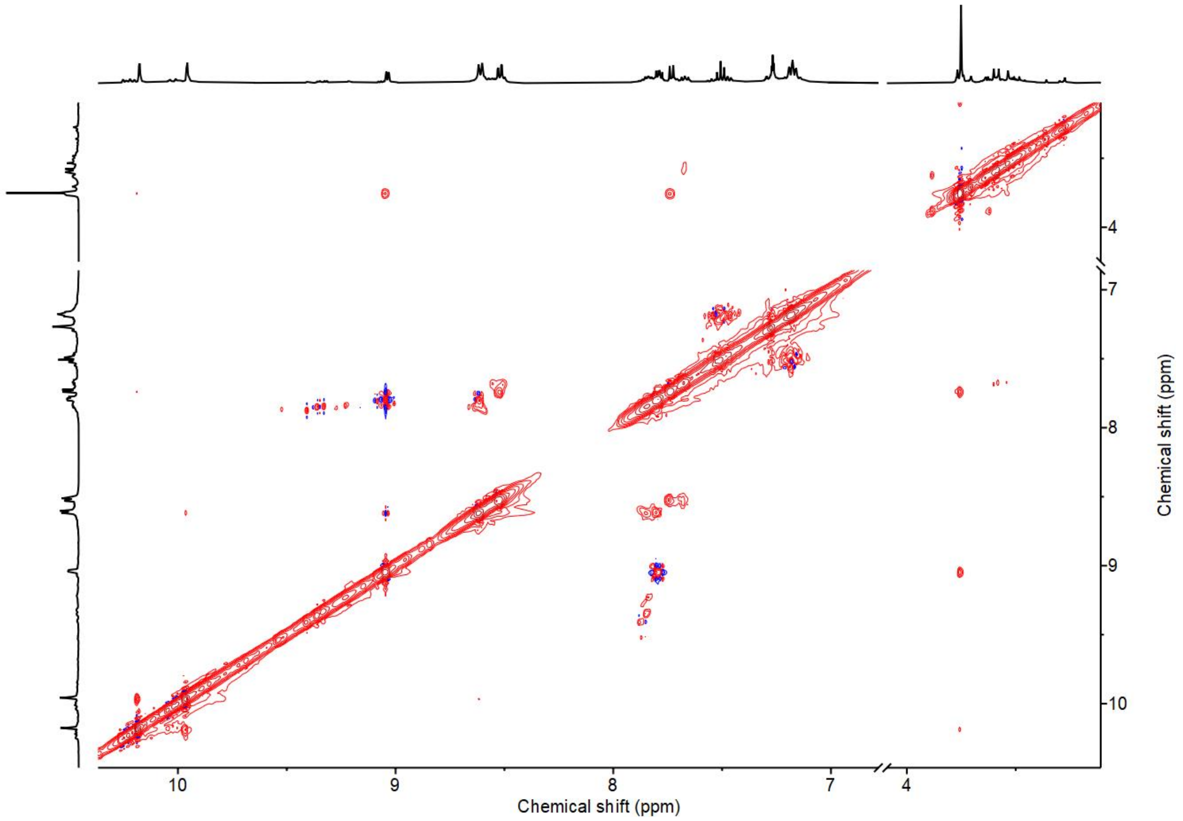


**Figure S174 Partial NOESY ($CD_3CN$) of C3[P].**

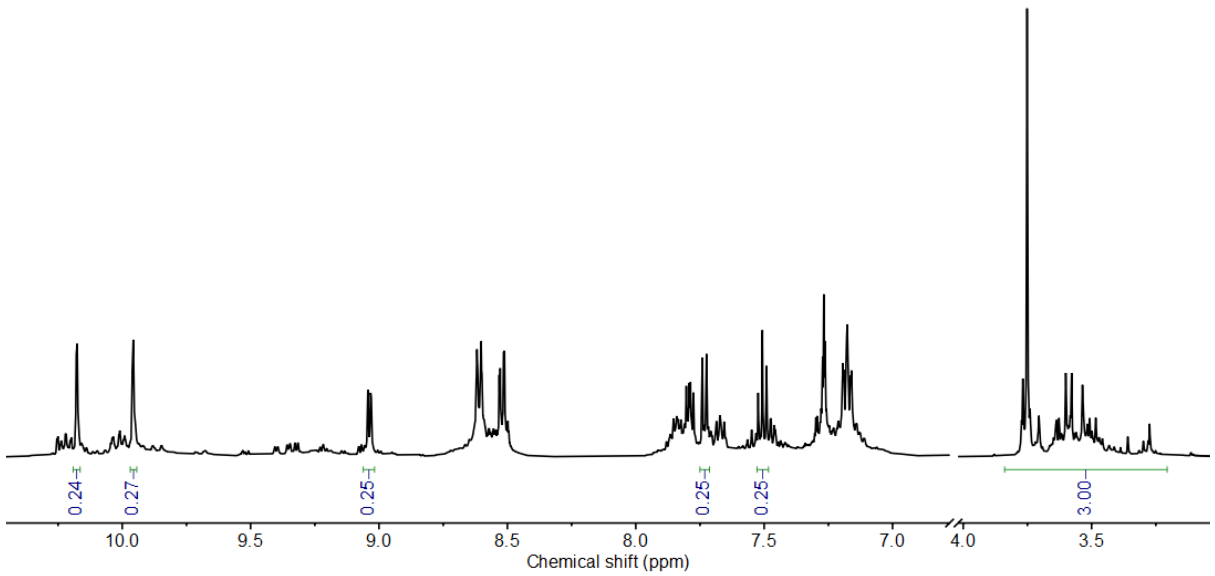


**Figure S175 Partial $^{1}H$ NMR (500 MHz, $CD_3CN$) of $C3^{P}$ with integrals relative to sum total of $CH_3$ signals.**

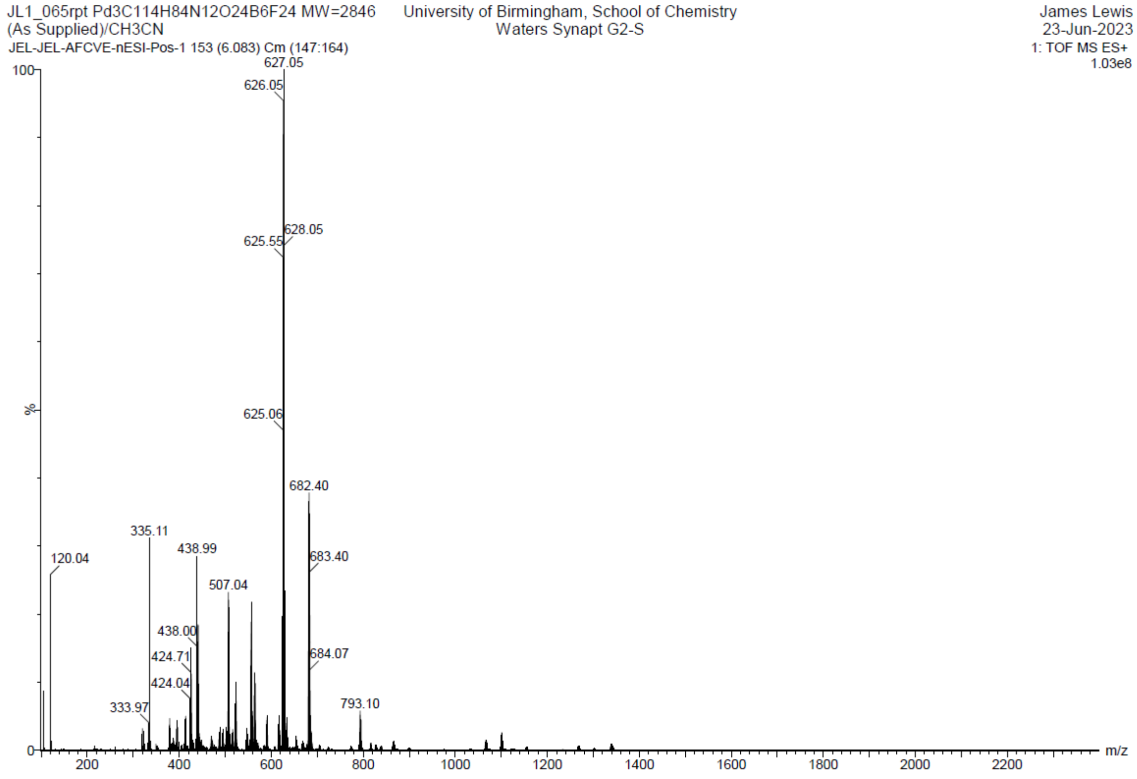


**Figure S176 ESI-MS of $C3^{P}$.**

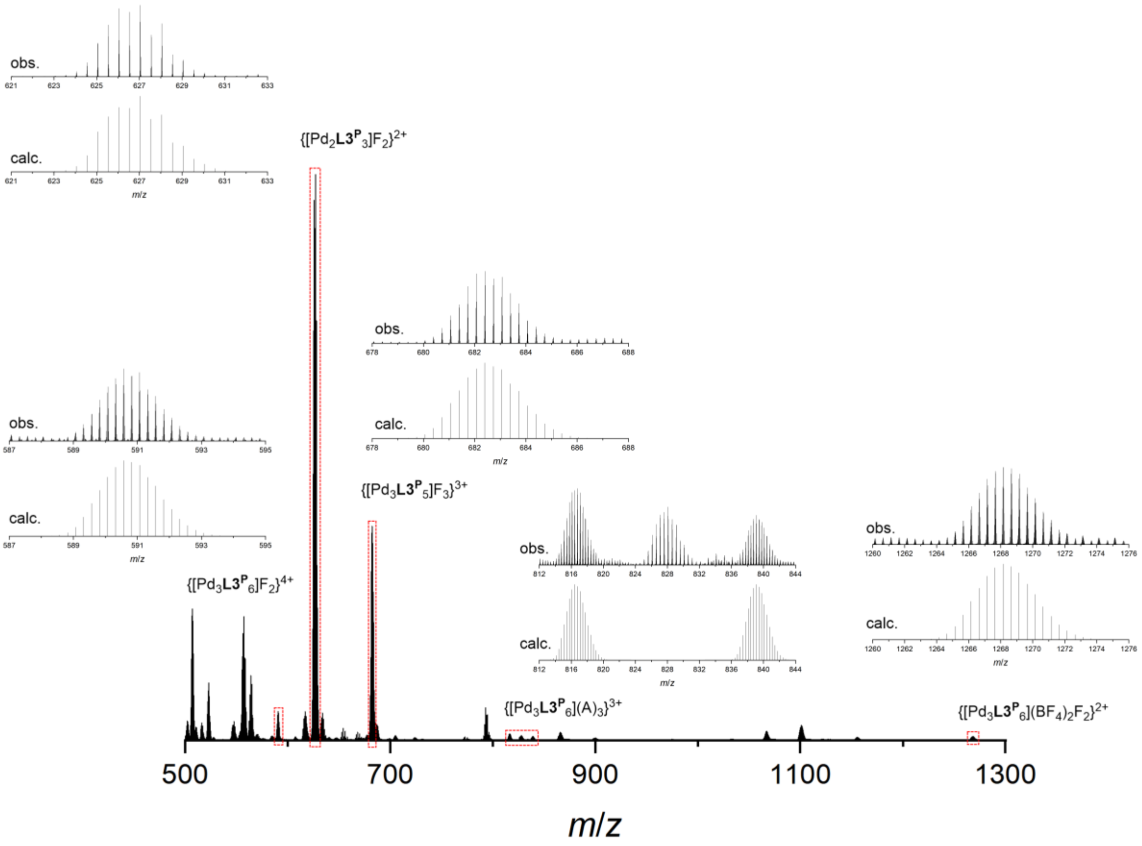


**Figure S177 ESI-MS of C3$^P$ with key peaks labelled.**

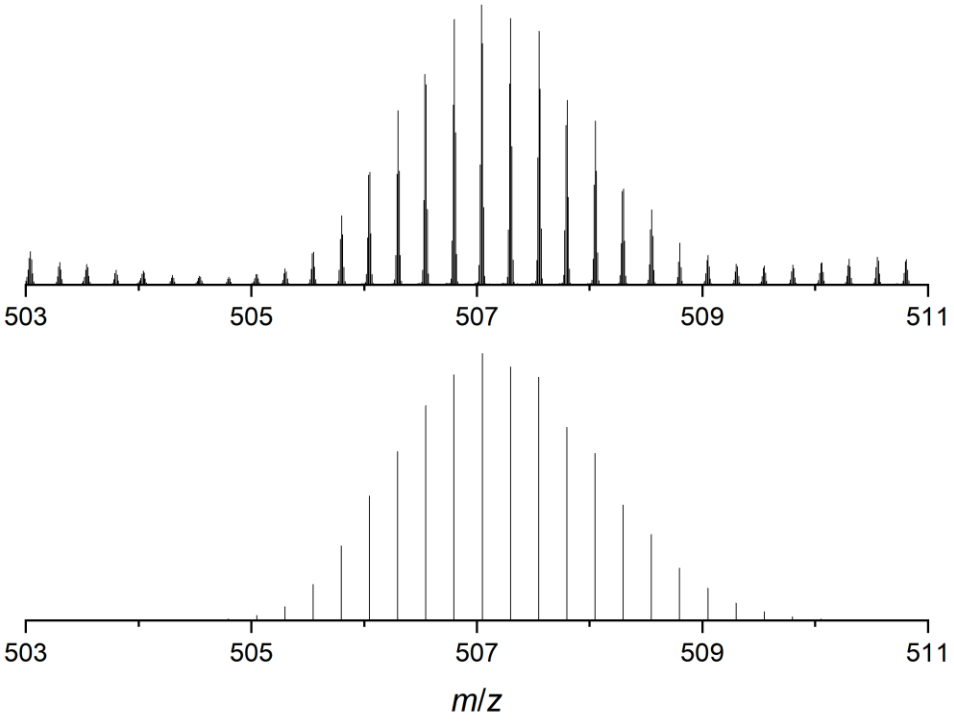


**Figure S178 Observed (top) and calculated (bottom) isotopic patterns for {[Pd$_3$(L3$^P$)$_5$]F$_2$}$^{4+}$.**

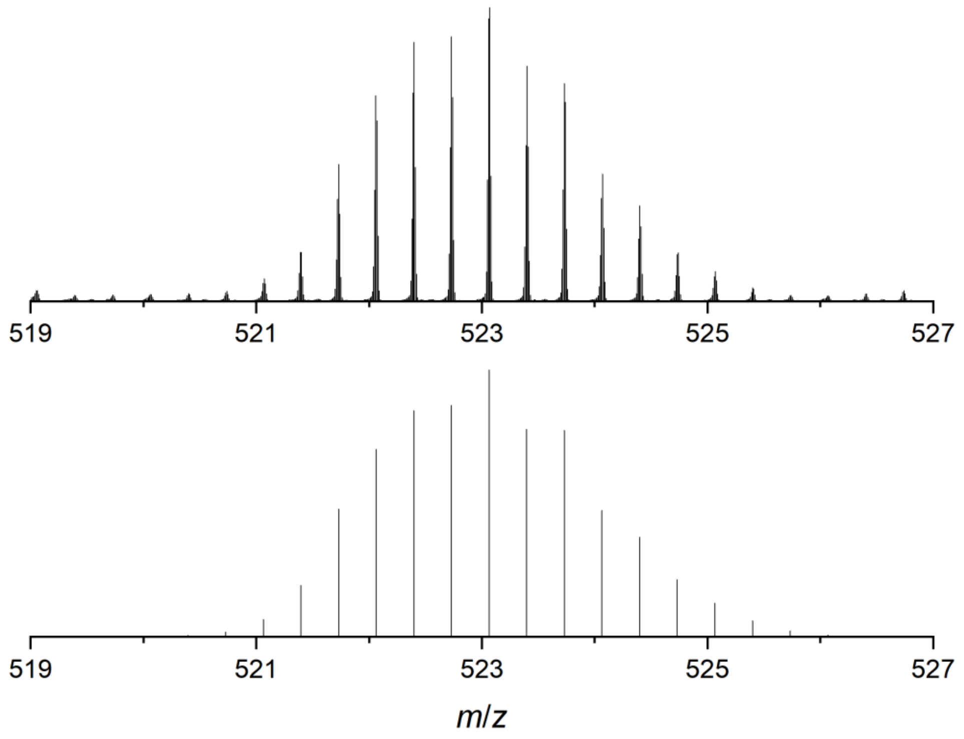


**Figure S179 Observed (top) and calculated (bottom) isotopic patterns for $\{[Pd_2(L3^P)_4]F\}^{3+}$.**

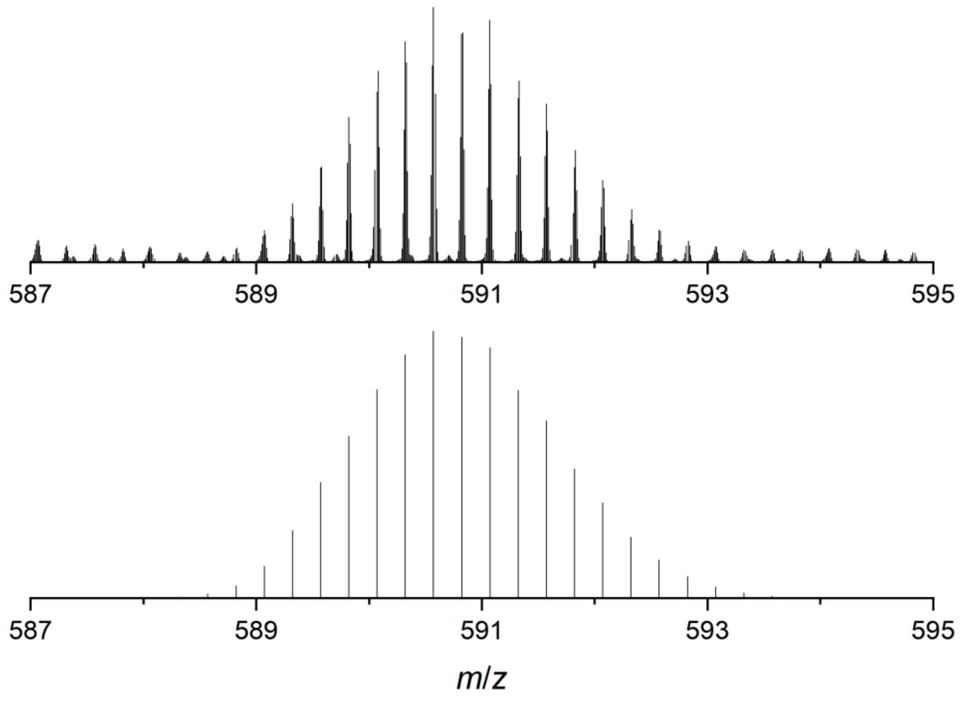


**Figure S180 Observed (top) and calculated (bottom) isotopic patterns for $\{[Pd_3(L3^P)_6]F_2\}^{4+}$.**

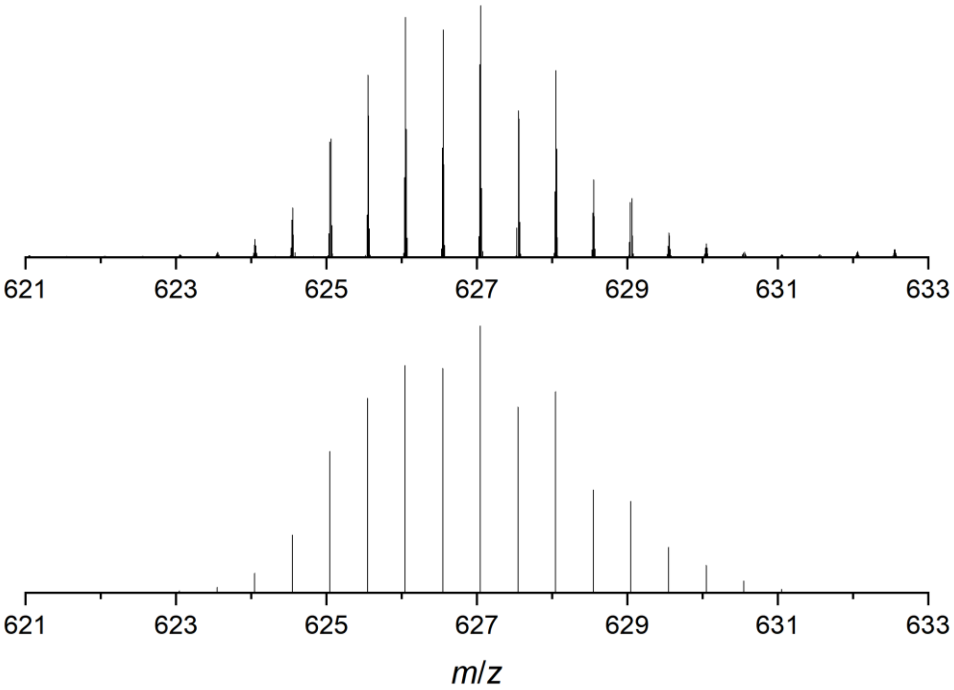


**Figure S181 Observed (top) and calculated (bottom) isotopic patterns for $\{[Pd_2(L3^P)_3]F_2\}^{2+}$.**

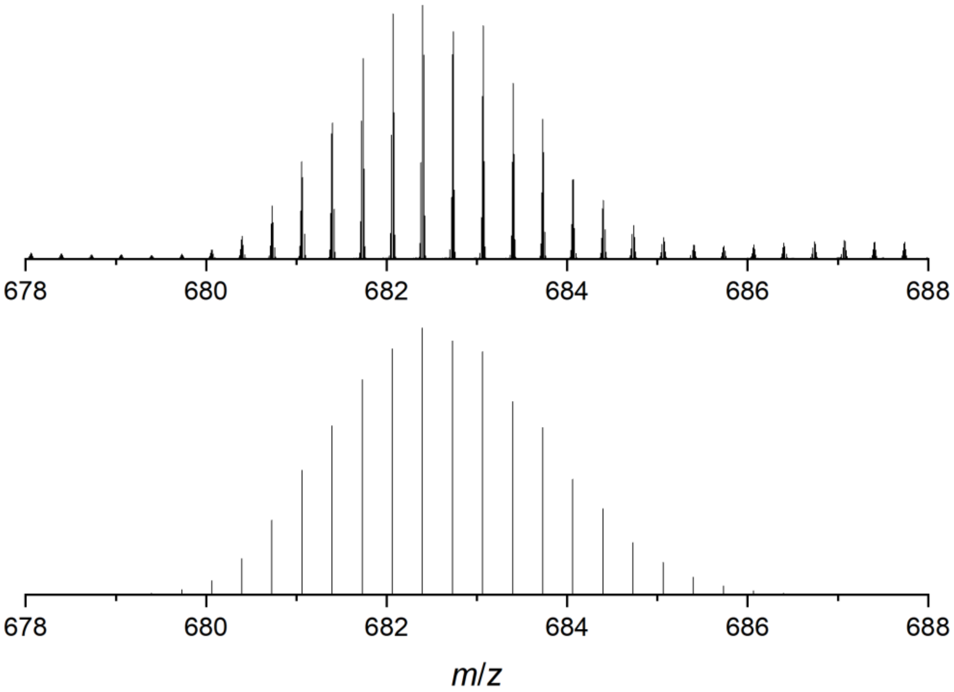


**Figure S182 Observed (top) and calculated (bottom) isotopic patterns for $\{[Pd_3(L3^P)_5]F_3\}^{3+}$.**

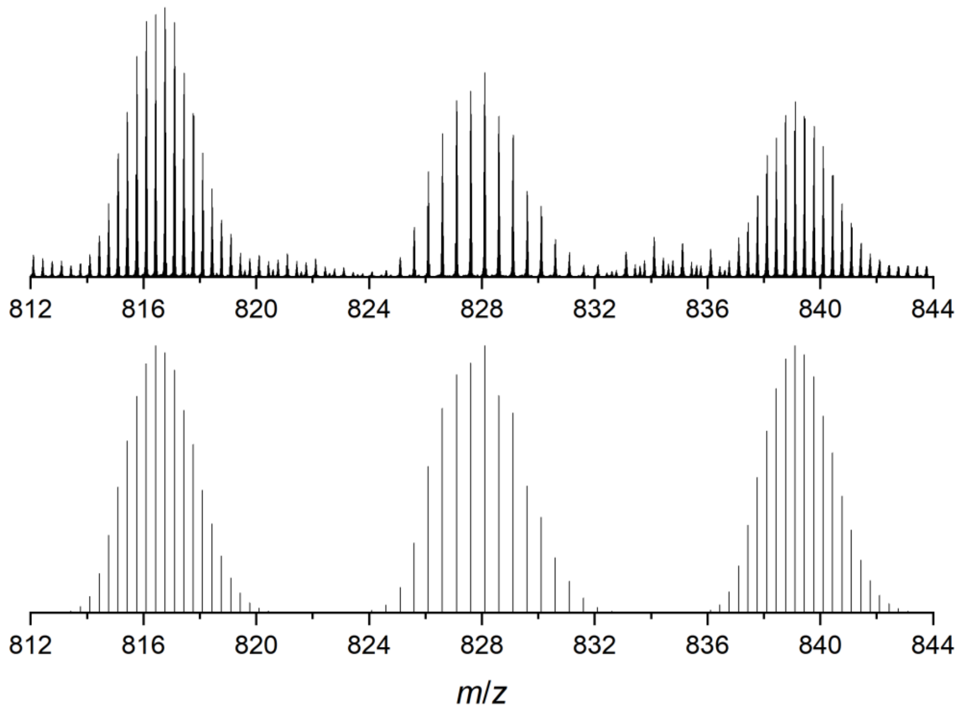


**Figure S183 Observed (top) and calculated (bottom) isotopic patterns for (from left to right) $\{[Pd_3(L3^P)_6](BF_4)F_2\}^{3+}$, $\{[Pd_2(L3^P)_4](BF_4)F\}^{2+}$ and $\{[Pd_3(L3^P)_6](BF_4)_2F\}^{3+}$.**

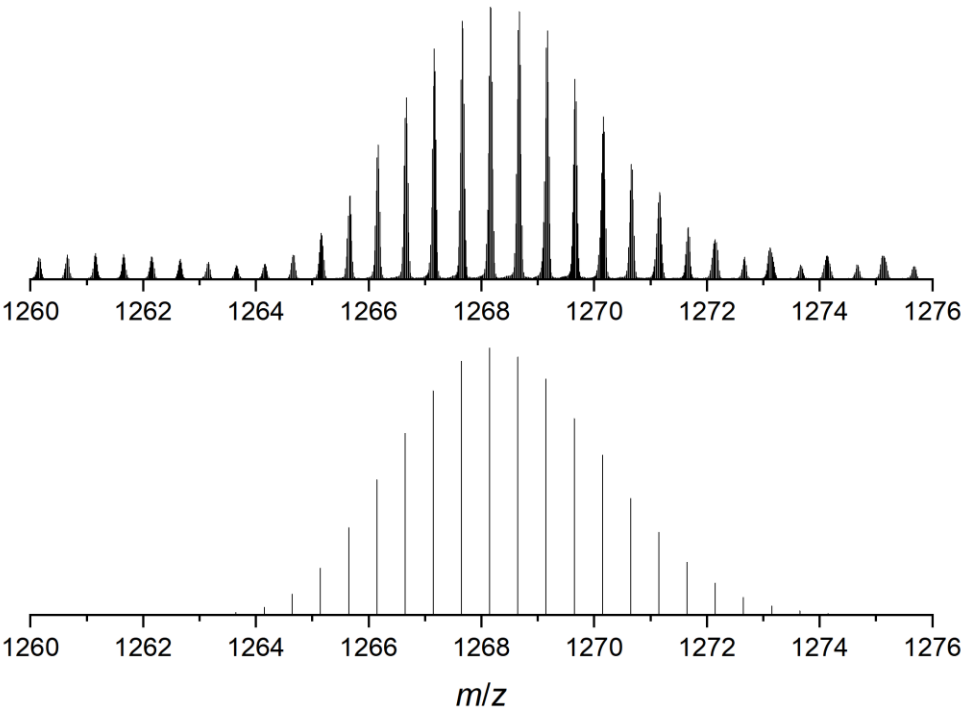


**Figure S184 Observed (top) and calculated (bottom) isotopic patterns for $\{[Pd_3(L3^P)_6](BF4)_2F_2\}^{2+}$.**

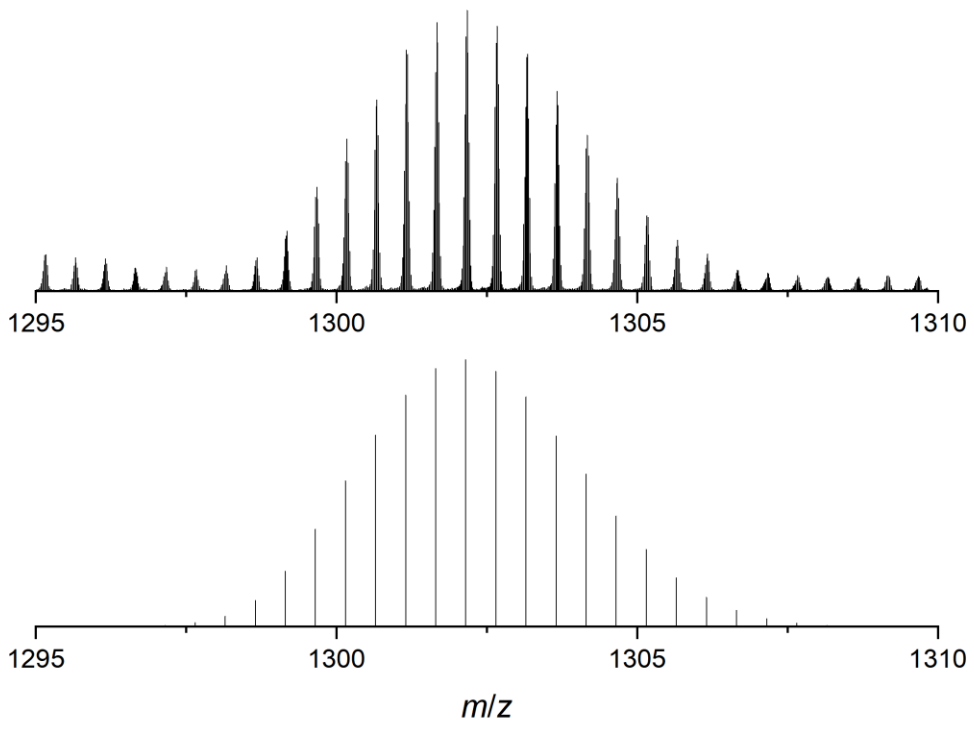


**Figure S185 Observed (top) and calculated (bottom) isotopic patterns for {[$Pd_3(L3^P)_6$]$(BF4)_3F$}$^{2+}$.**

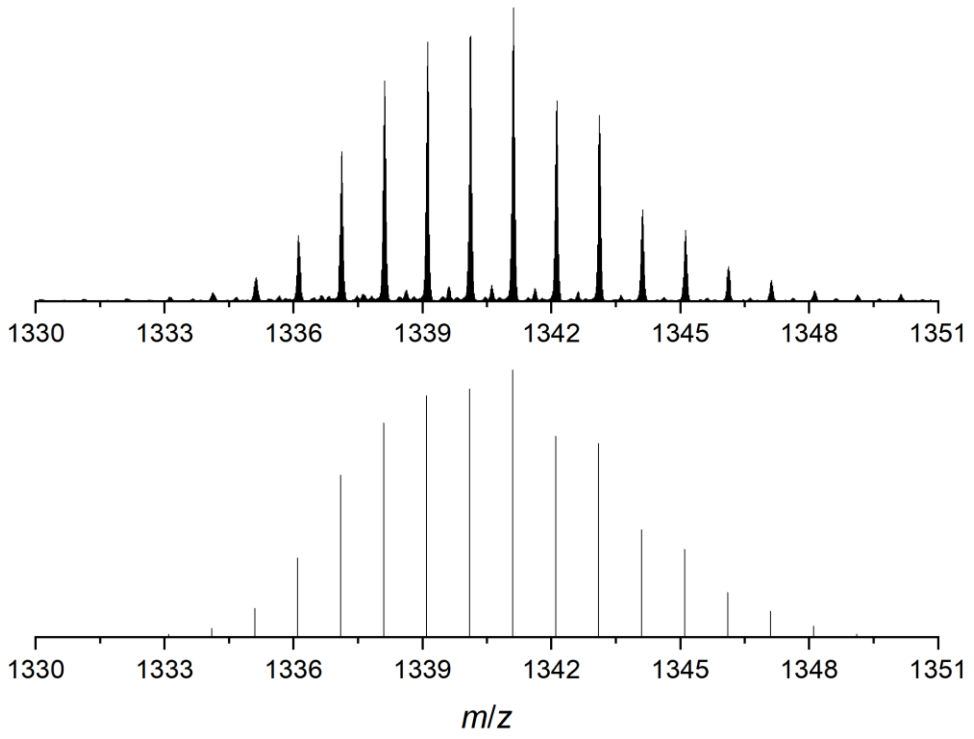


**Figure S186 Observed (top) and calculated (bottom) isotopic patterns for {[$Pd_2(L3^P)_3$]$(BF_4)F_2$}$^{+}$.**

## S2.21 Synthesis of L4$^{Q}$

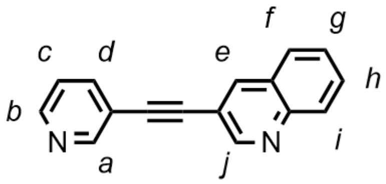


3-Ethynylpyridine (0.103 g, 1.0 mmol, 1.0 eq.), 3-bromoquinoline (0.208 g, 1.0 mmol, 1.0 eq.) and $Pd(PPh_3)_2Cl_2$ (0.035 g, 0.05 mmol, 5 mol%) were stirred at 70 °C in 1.0 M TBAF in THF (5 mL) in a sealed vial for 4 h. $H_2O$ (20 mL) was added to the cooled reaction mixture, and subsequently extracted with $Et_2O$ (3 × 20 mL). The combined organic phases were dried ($MgSO_4$) and the solvent removed *in vacuo*. After purification by column chromatography on silica gel (step gradient 0 to 50% EtOAc in petrol in 10% increments) the product was obtained as a white solid (0.200 g, 87%).

**$^{1}$H NMR** (500 MHz, $CDCl_3$) δ: 9.01 (d, $J$ = 2.1 Hz, 1H, $H_j$), 8.83 (d, $J$ = 1.4 Hz, 1H, $H_a$), 8.60 (dd, $J$ = 4.9, 1.7 Hz, 1H, $H_b$), 8.35 (d, $J$ = 2.0 Hz, 2H, $H_e$), 8.12 (d, $J$ = 8.5 Hz, 1H, $H_i$), 7.87 (app. dt, $J$ = 7.9, 1.9 Hz, 1H, $H_d$), 7.82 (dd, $J$ = 8.1, 1.4 Hz, 1H, $H_f$), 7.75 (ddd, $J$ = 8.4, 6.8, 1.4 Hz, 1H, $H_h$), 7.59 (ddd, $J$ = 8.1, 6.8, 1.2 Hz, 1H, $H_g$), 7.33 (ddd, $J$ = 7.8, 4.9, 0.9 Hz, 1H, $H_c$).

**$^{13}$C NMR** (126 MHz, $CDCl_3$) δ: 152.5 ($C_a$), 152.0 ($C_j$), 149.2 ($C_b$), 147.2, 138.8 ($C_e$/$C_d$), 138.7 ($C_e$/$C_d$), 130.6 ($C_h$), 129.6 ($C_i$), 127.8 ($C_f$), 127.6 ($C_g$), 127.3, 123.3 ($C_c$), 120.0, 116.8, 90.0, 89.3.

**HR-EI-MS** $m/z$ = 230.0840 $[M]^+$ calc. 230.0838.

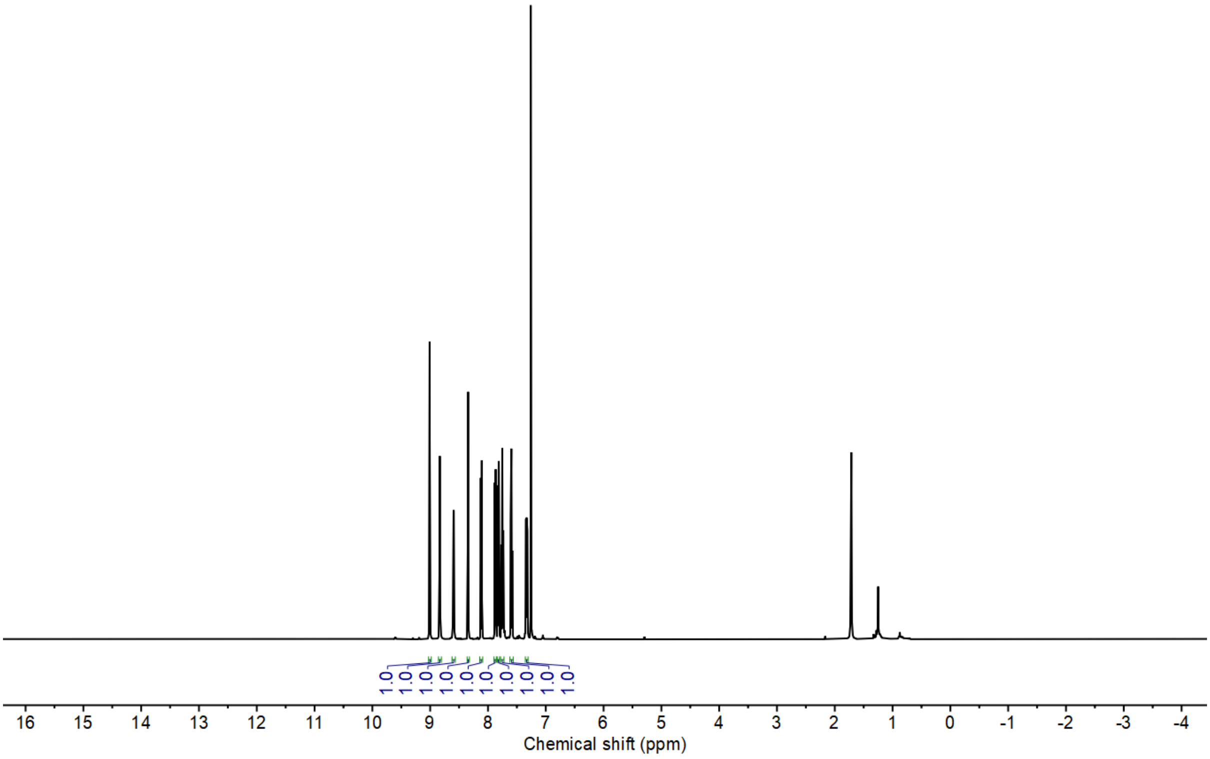


**Figure S187 $^{1}$H NMR (500 MHz, $CDCl_3$) of L4$^{Q}$.**

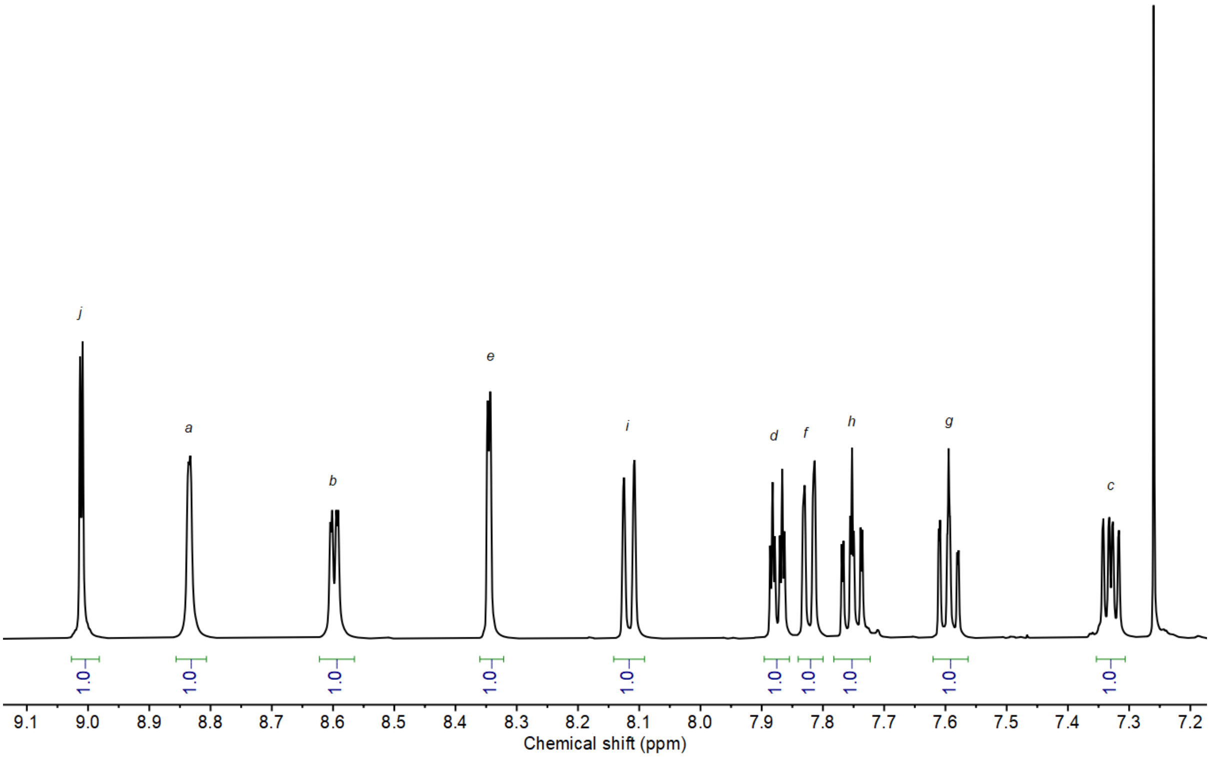


**Figure S188 Partial $^{1}H$ NMR (500 MHz, $CDCl_3$) of L4$^{Q}$.**

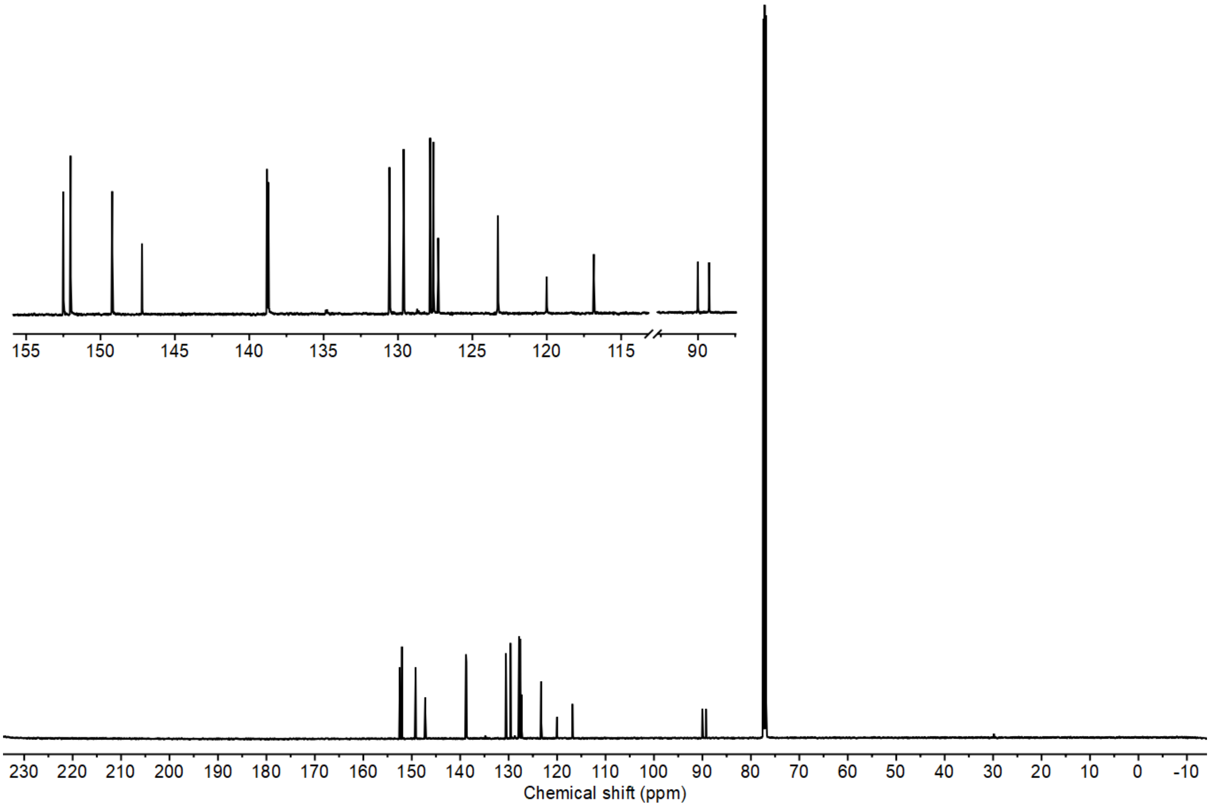


**Figure S189 $^{13}C$ NMR (126 MHz, $CDCl_3$) of L4$^{Q}$.**

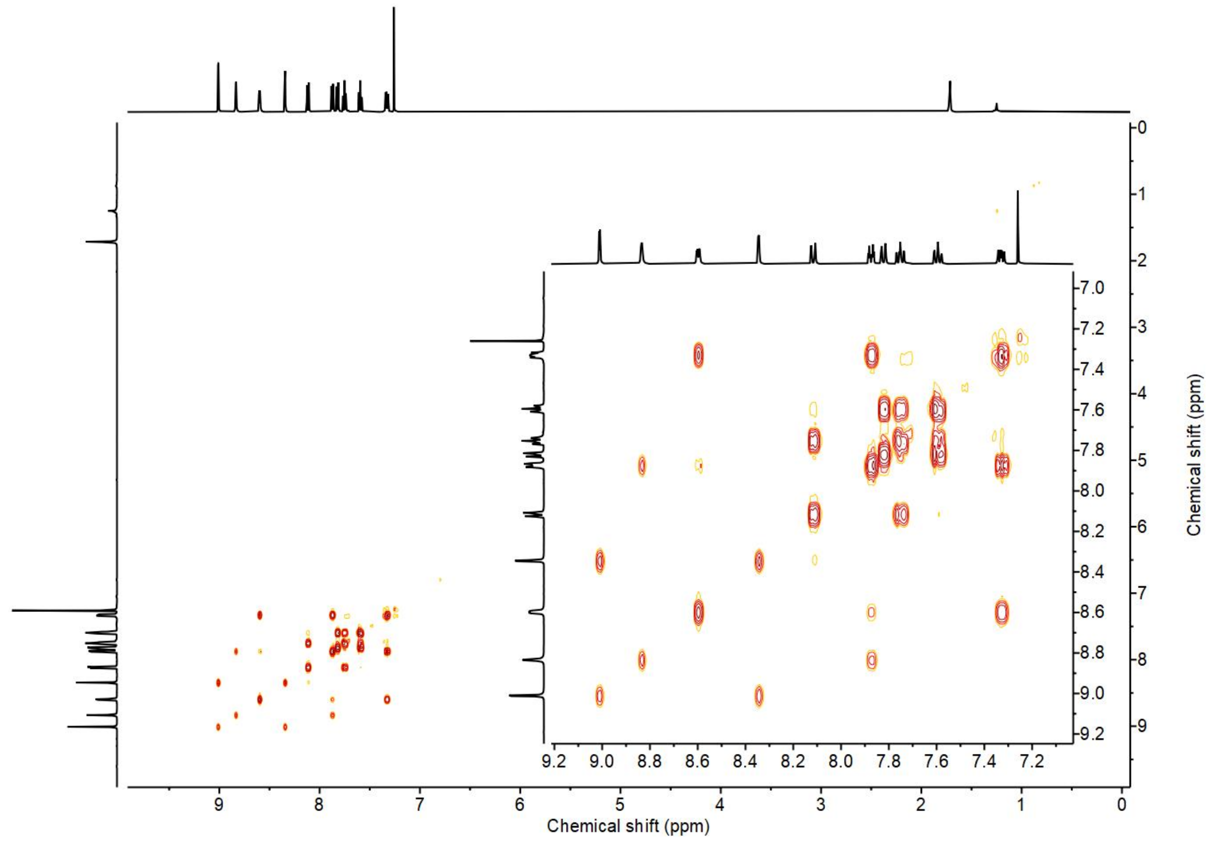


**Figure S190 COSY NMR ($CDCl_3$) of L4$^{Q}$.**

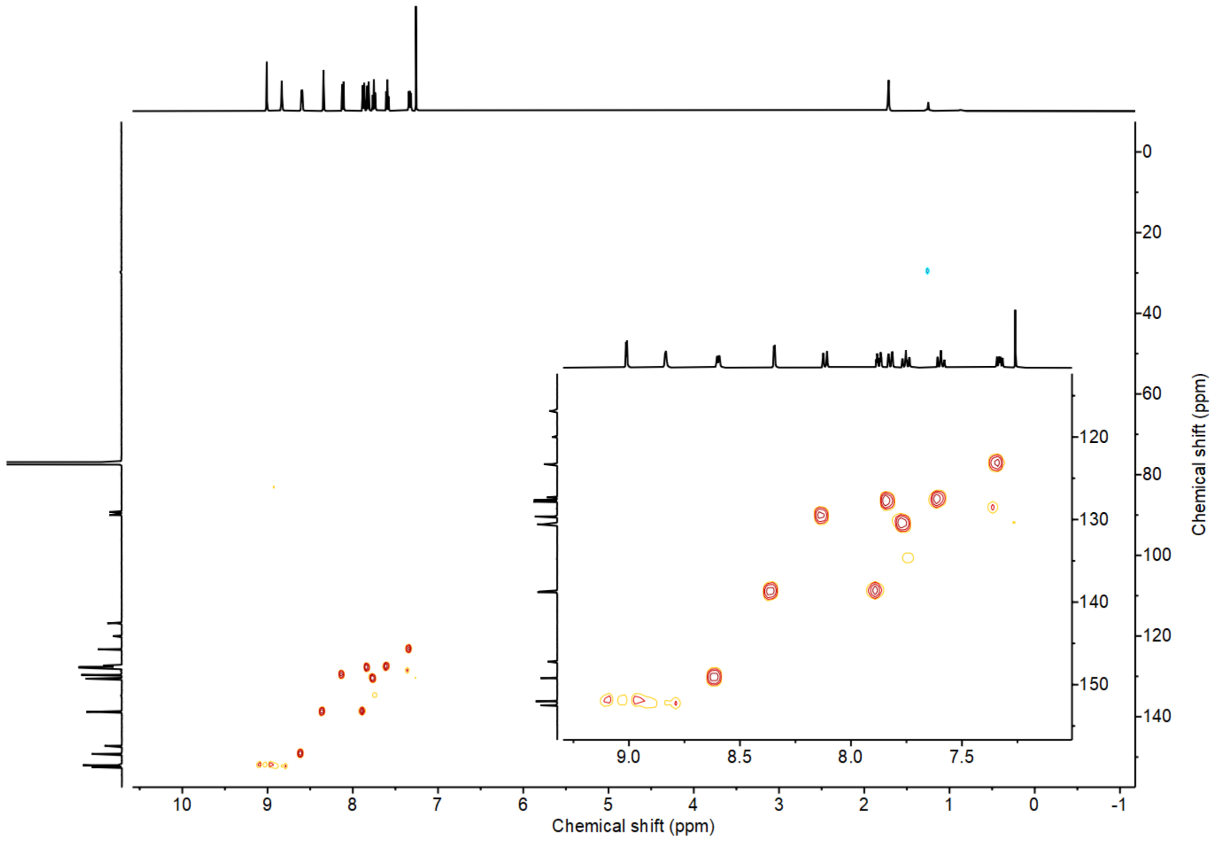


**Figure S191 HSQC NMR ($CDCl_3$) of L4$^{Q}$.**

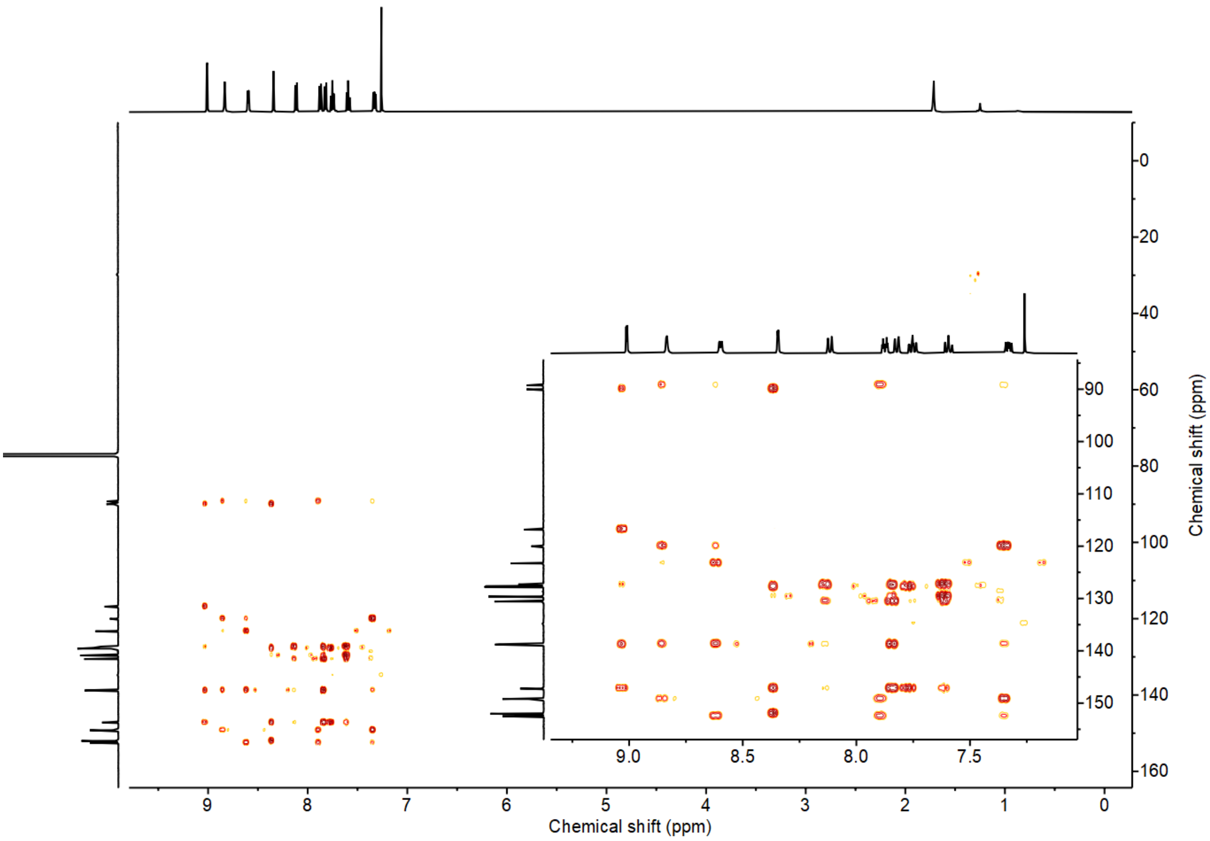


**Figure S192 HMBC NMR (CDCl$_3$) of L4$^{Q}$.**

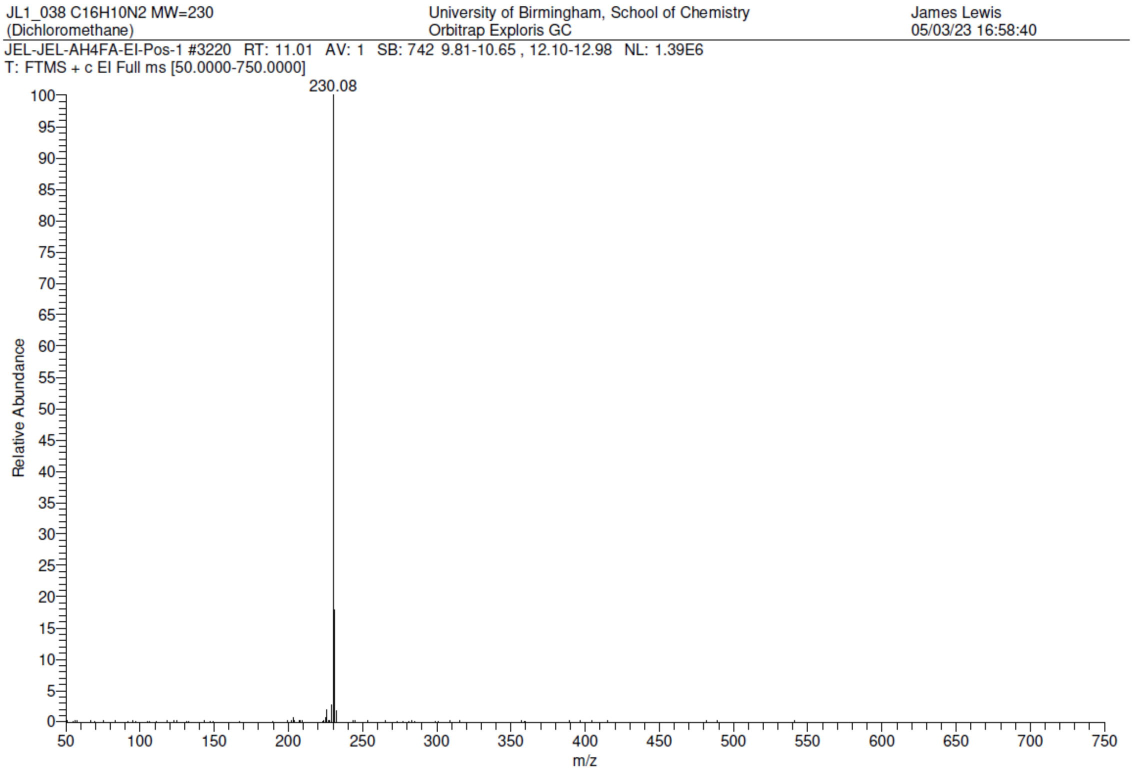


**Figure S193 EI-MS of L4$^{Q}$.**

## S2.22 Self-assembly of $L4^{Q}$ with $[Pd(CH_3CN)_4](BF_4)_2$

**$L4^{Q}$** (6.9 mg, 30 μmol, 1 eq.) and $[Pd(CH_3CN)_4](BF_4)_2$ (7.3 mg, 16.5 μmol, 0.55 eq.) were sonicated in *d*$_6$-DMSO (0.75 mL) until a homogenous solution was obtained. This was then stood at 50 °C for 18 h, with no further changes to the $^1$H NMR spectrum observed with further heating.

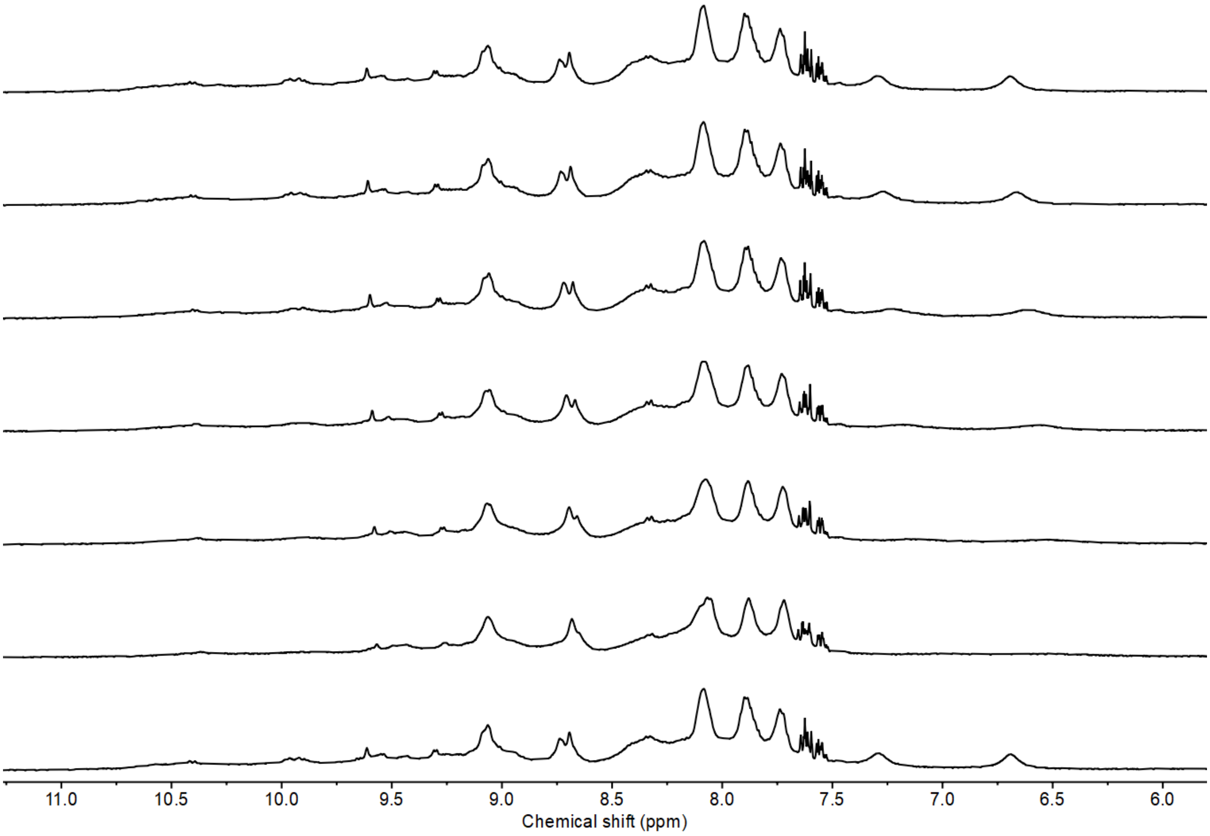


**Figure S194 Partial $^1$H NMR (400 MHz, *d*$_6$-DMSO) of the equilibrated mixture of $L4^{Q}$ and $[Pd(CH_3CN)_4](BF_4)_2$ at 298 K, 303 K, 313 K, 323 K, 333 K, 343, and BACK TO 303 K (from top to bottom).**

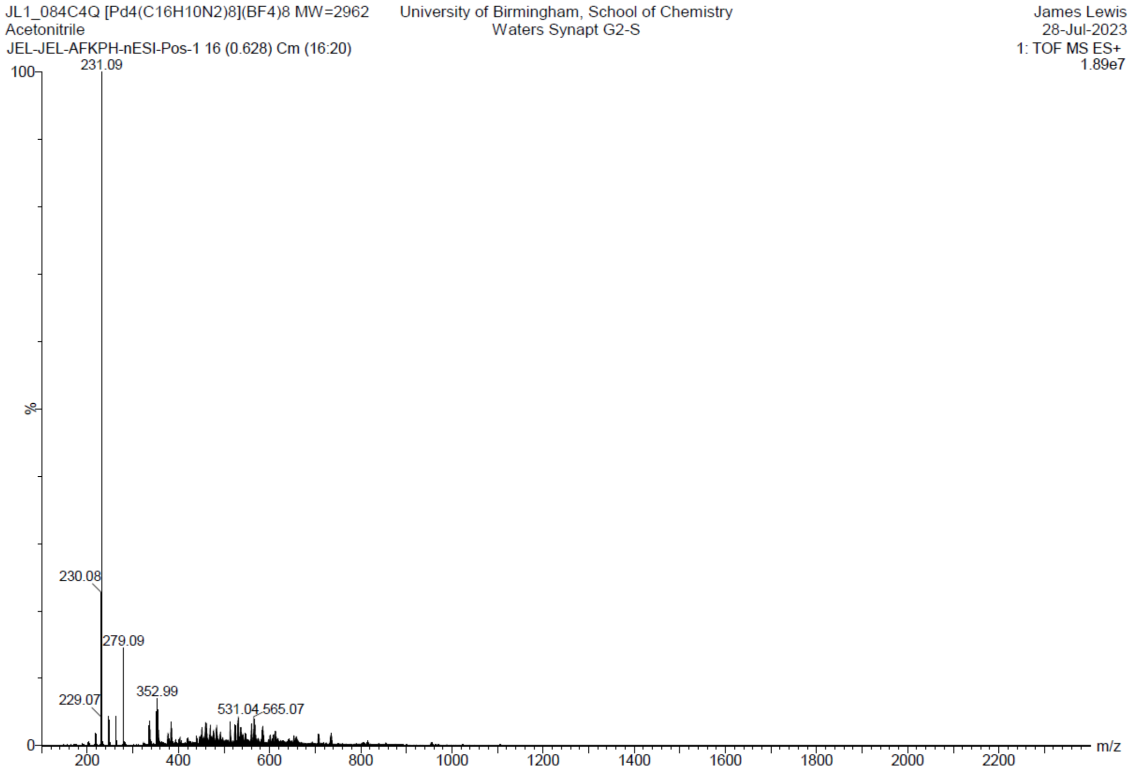


**Figure S195 ESI-MS of the equilibrated mixture of $L4^{Q}$ and $[Pd(CH_3CN)_4](BF_4)_2$.**

## S2.23 Synthesis of L4$^P$

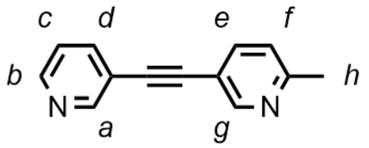


3-Ethynylpyridine (0.103 g, 1.0 mmol, 1.0 eq.), 5-bromo-2-methylpyridine (0.172 g, 1.0 mmol, 1.0 eq.) and $Pd(PPh_3)_2Cl_2$ (0.035 g, 0.05 mmol, 5 mol%) were stirred at 70 °C in 1.0 M TBAF in THF (5 mL) in a sealed vial for 4 h. $H_2O$ (20 mL) and brine (10 mL) were added to the cooled reaction mixture, and the aqueous phase extracted with $Et_2O$ (3 × 20 mL). The combined organic phases were dried ($MgSO_4$) and the solvent removed *in vacuo*. After purification by column chromatography (1:3 followed by 1:1 EtOAc/*n*-hexane) the product was obtained as a white solid (0.0667 g, 34%).

**$^1$H NMR** (500 MHz, $CDCl_3$) δ: 8.78 (s, 1H, $H_a$), 8.69 (d, $J$ = 1.5 Hz, 1H, $H_g$), 8.59 (d, $J$ = 4.2 Hz, 1H, $H_b$), 7.85-7.82 (m, 2H, $H_d$, $H_e$), 7.33 (dd, $J$ = 7.9, 4.9 Hz, 1H, $H_c$), 7.27-7.26 (m, 1H, $H_f$), 2.67 (s, 3H, $H_h$).

**$^{13}$C NMR** (126 MHz, $CDCl_3$) δ: 157.6, 152.2 ($C_a$), 150.1 ($C_g$), 149.0 ($C_b$), 140.4 ($C_e$), 138.9 ($C_d$), 123.9 ($C_f$), 123.4 ($C_c$), 119.9, 117.6, 89.5, 88.8, 23.8 ($C_h$).

**HR-EI-MS** $m/z$ = 194.0840 $[M]^+$ calc. 194.0838.

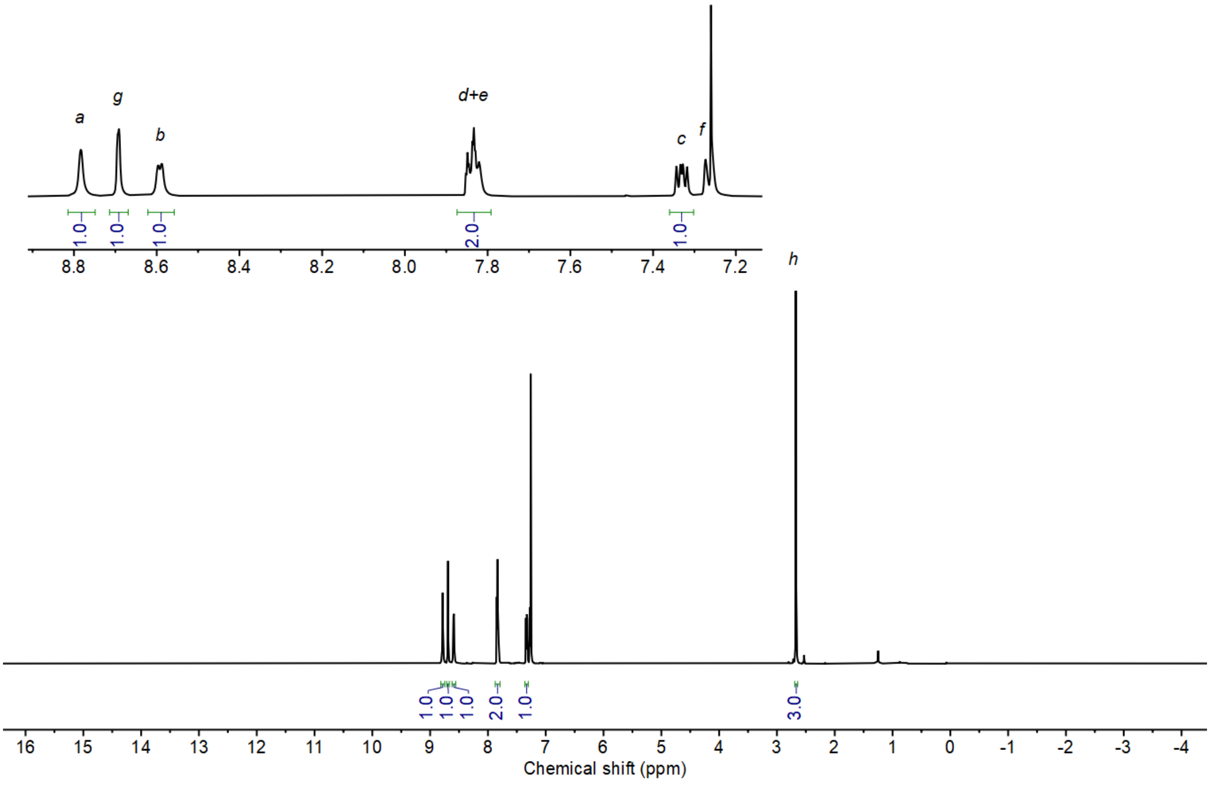


**Figure S196 $^1$H NMR (500 MHz, $CDCl_3$) of L4$^P$.**

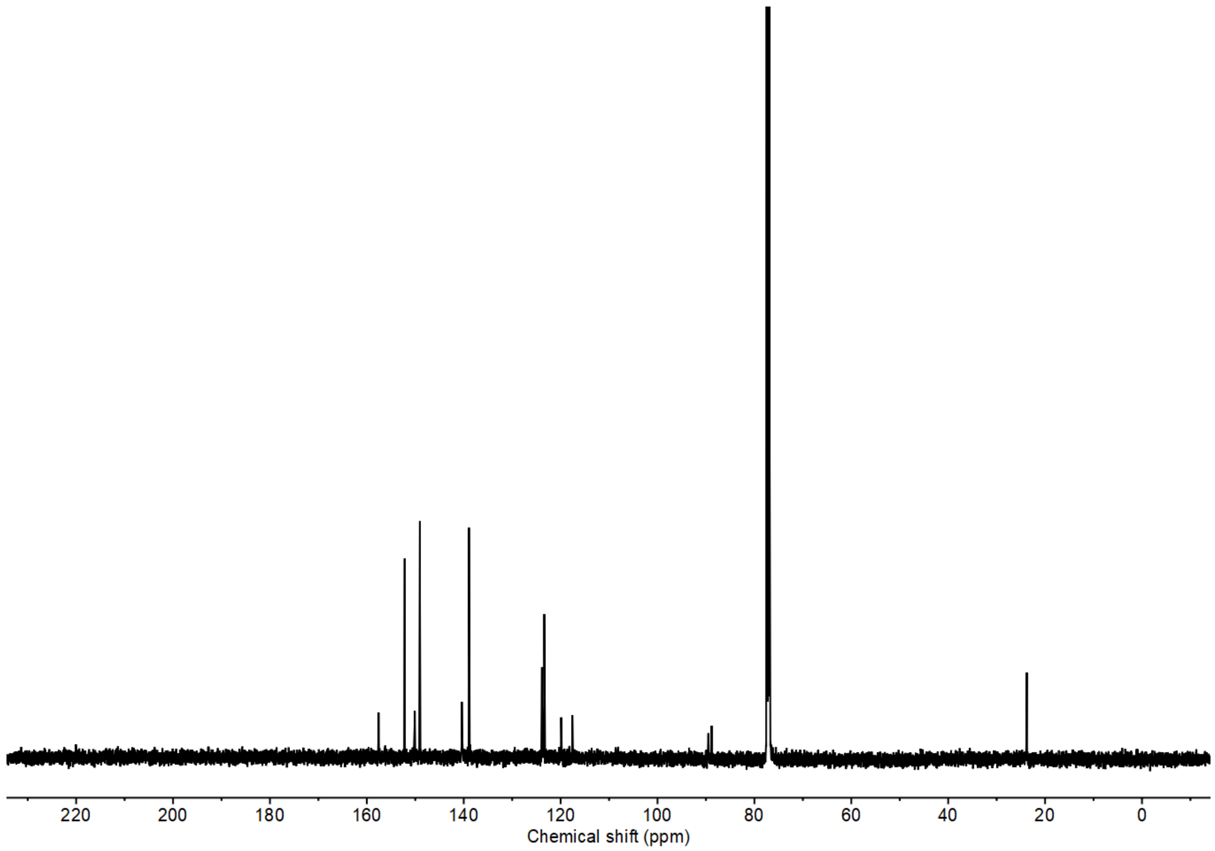


**Figure S197 $^{13}C$ NMR (126 MHz, $CDCl_3$) of L4$^{P}$.**

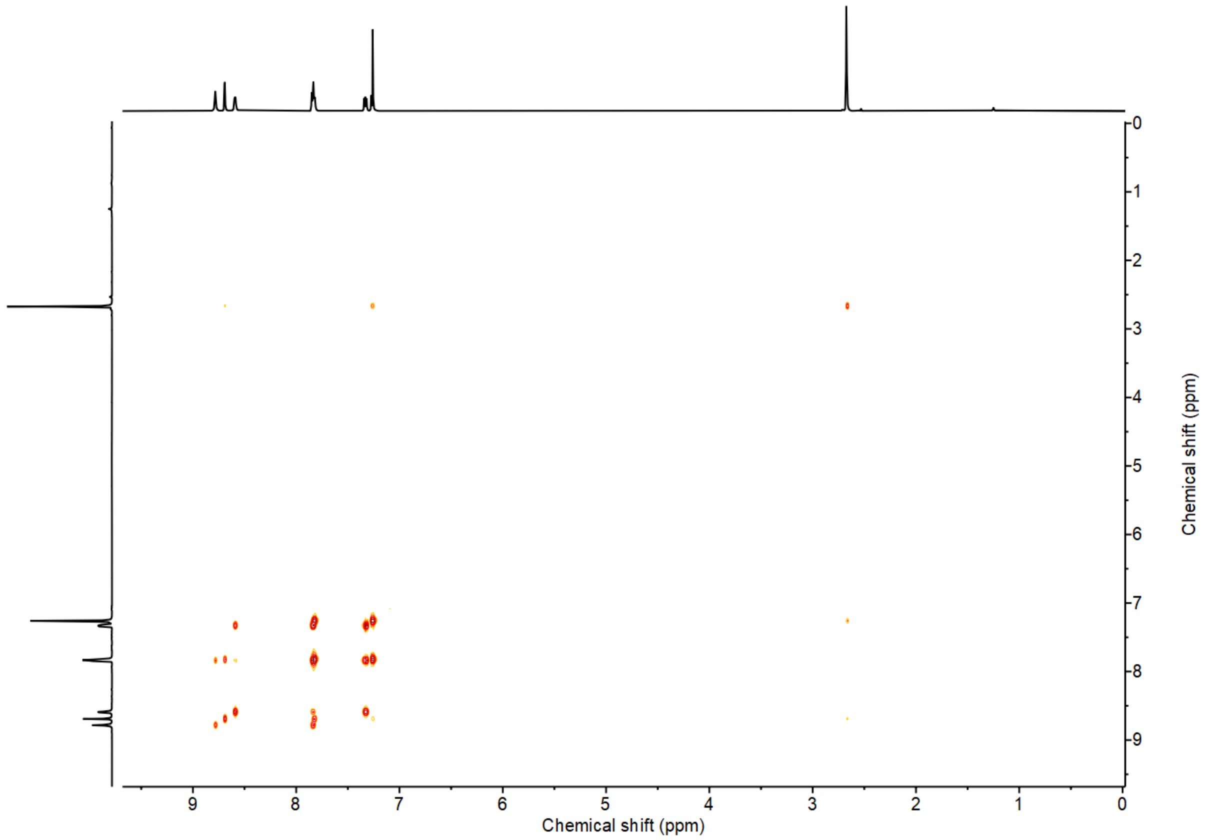


**Figure S198 COSY ($CDCl_3$) of L4$^{P}$.**

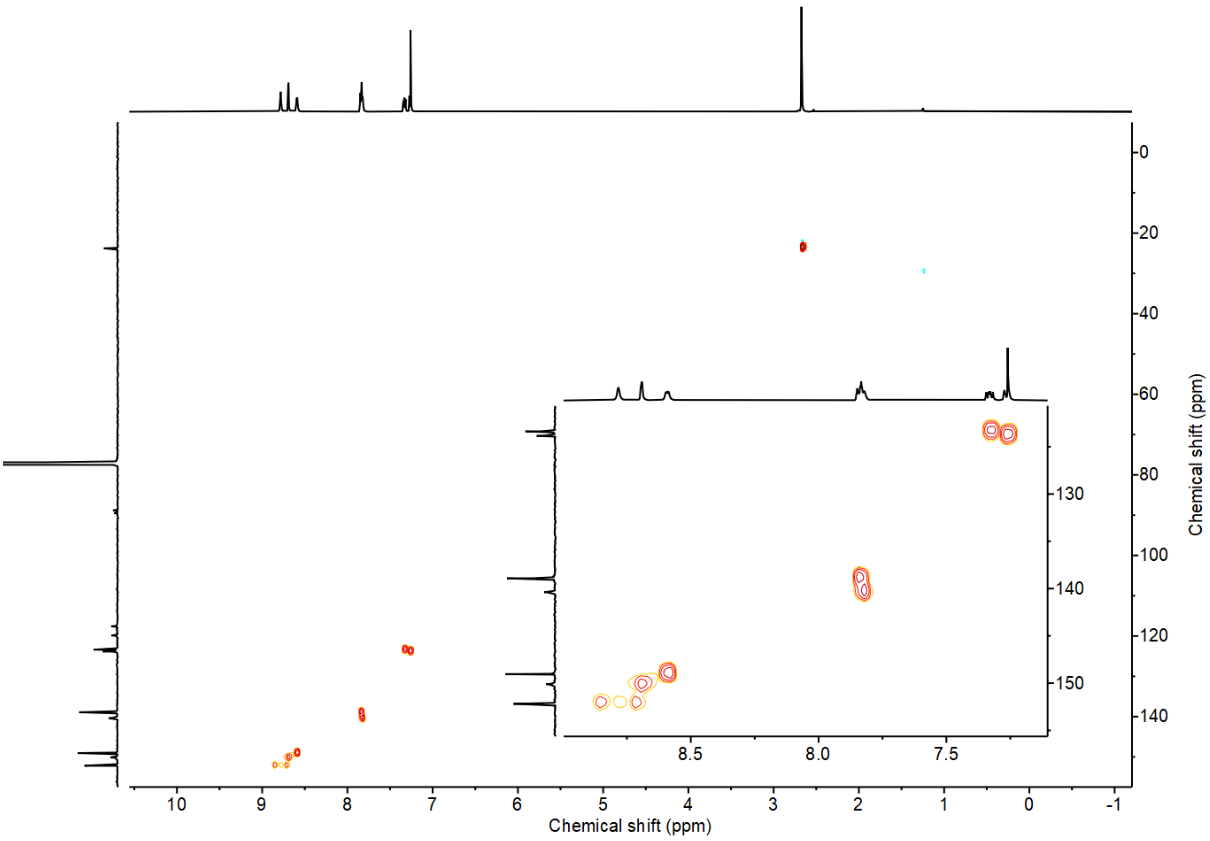


**Figure S199 HSQC ($CDCl_3$) of L4[P].**

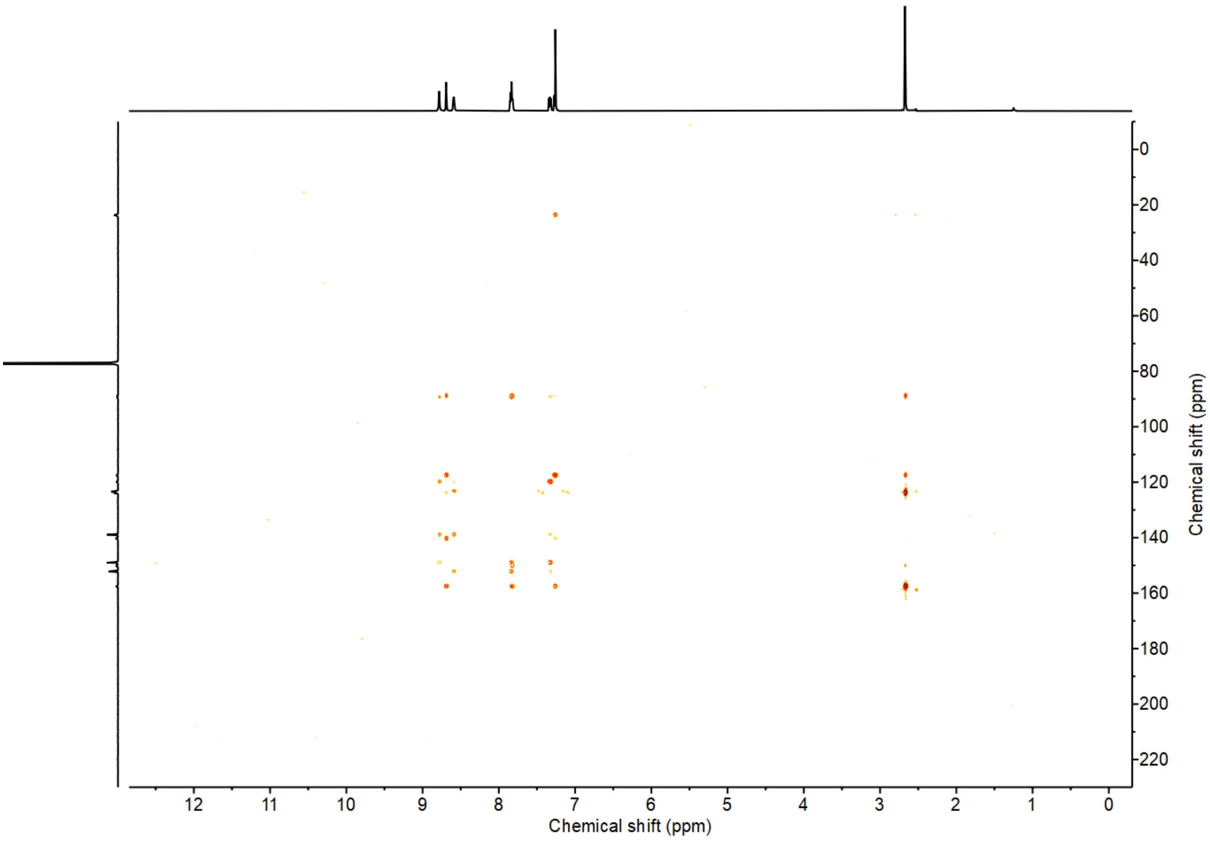


**Figure S200 HMBC ($CDCl_3$) of L4[P].**

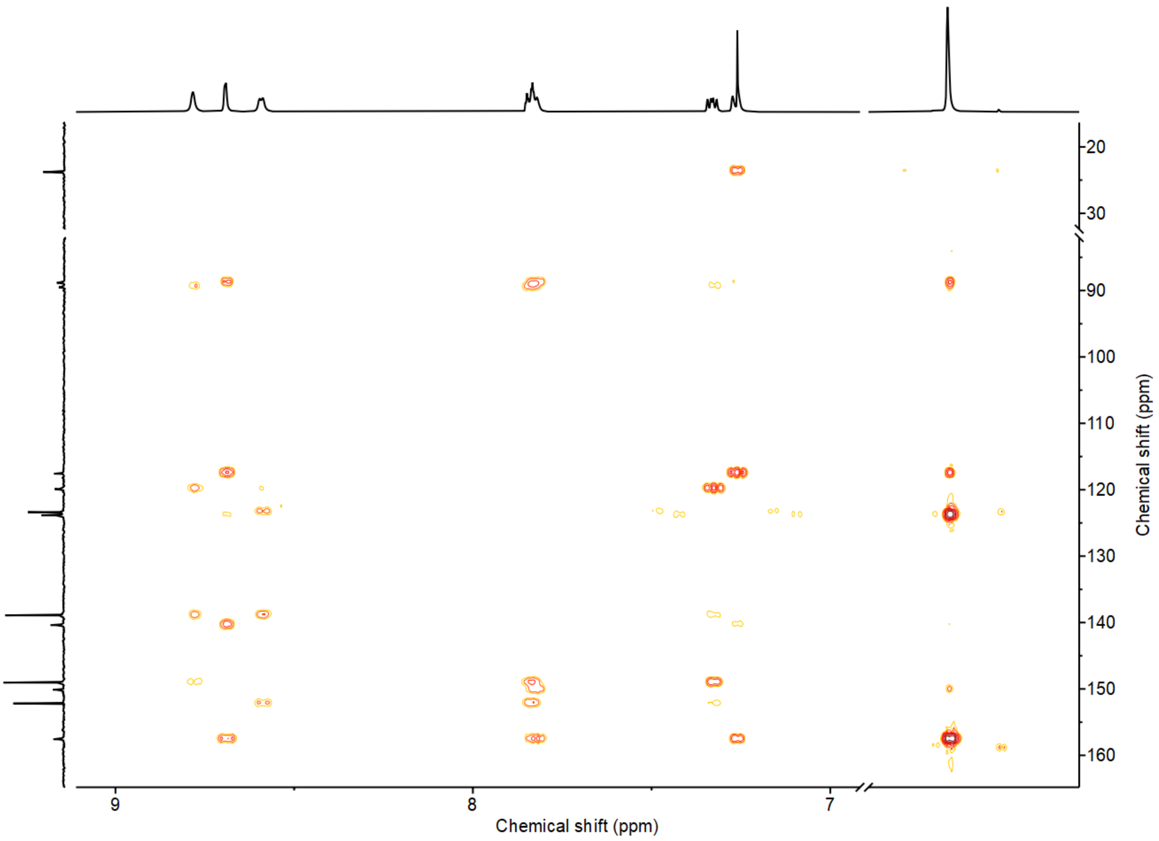


**Figure S201 Partial HMBC ($CDCl_3$) of L4$^{P}$.**

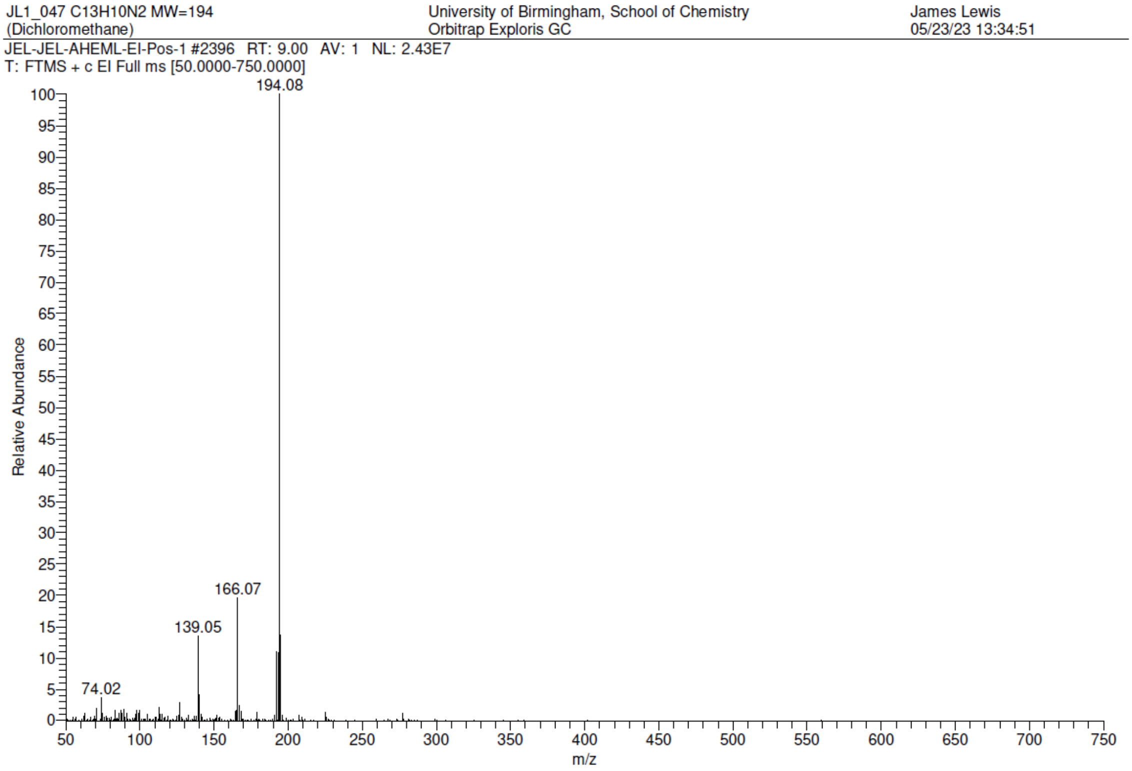


**Figure S202 EI-MS of L4$^{P}$.**

## S2.24 Self-assembly of L4^P with $[Pd(CH_3CN)_4](BF_4)_2$

**L4^P** (5.8 mg, 30 μmol, 1 eq.) and $[Pd(CH_3CN)_4](BF_4)_2$ (7.3 mg, 16.5 μmol, 0.55 eq.) were sonicated in $d_6$-DMSO (0.75 mL) until a homogenous solution was obtained. This was then stood at 60 °C for 20 h, with no further changes to the $^1H$ NMR spectrum observed with further heating.

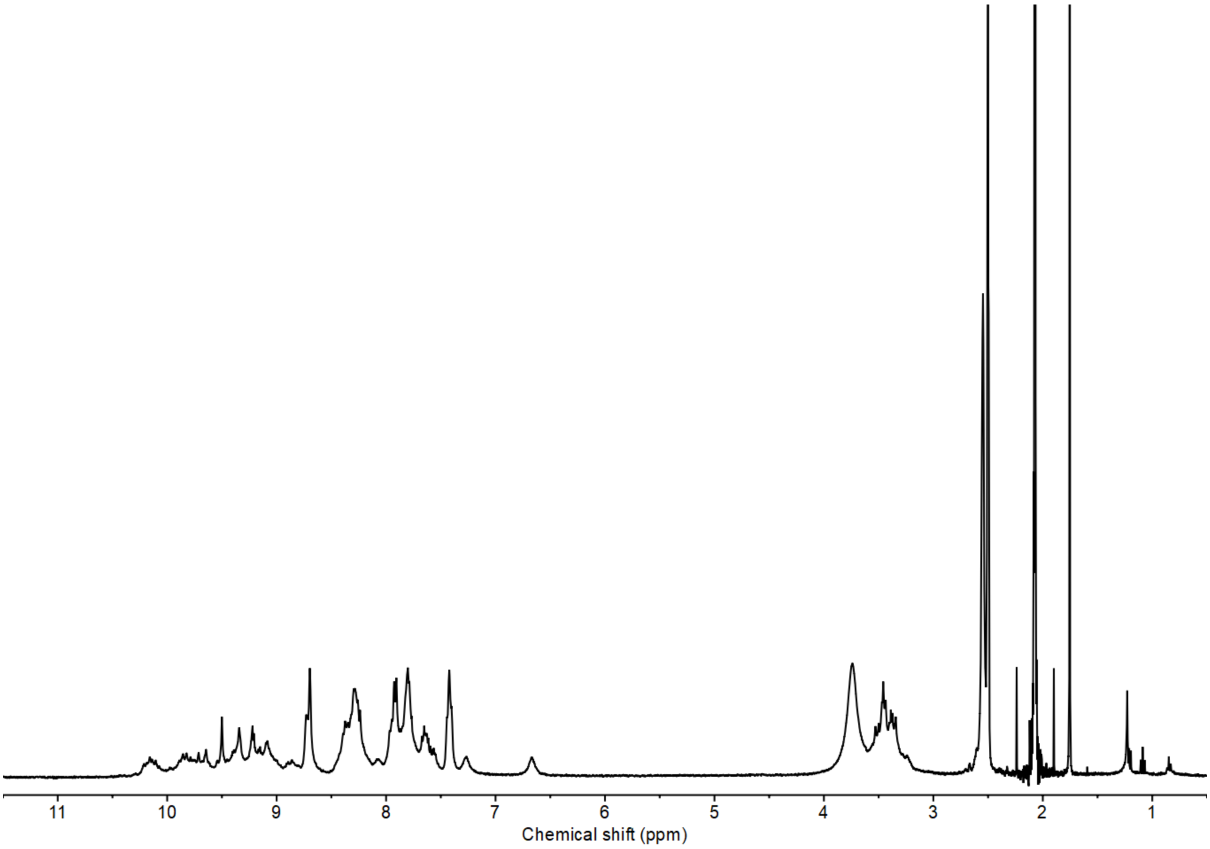


**Figure S203 $^1H$ NMR (400 MHz, $d_6$-DMSO) of the equilibrated mixture of L4^P and $[Pd(CH_3CN)_4](BF_4)_2$.**

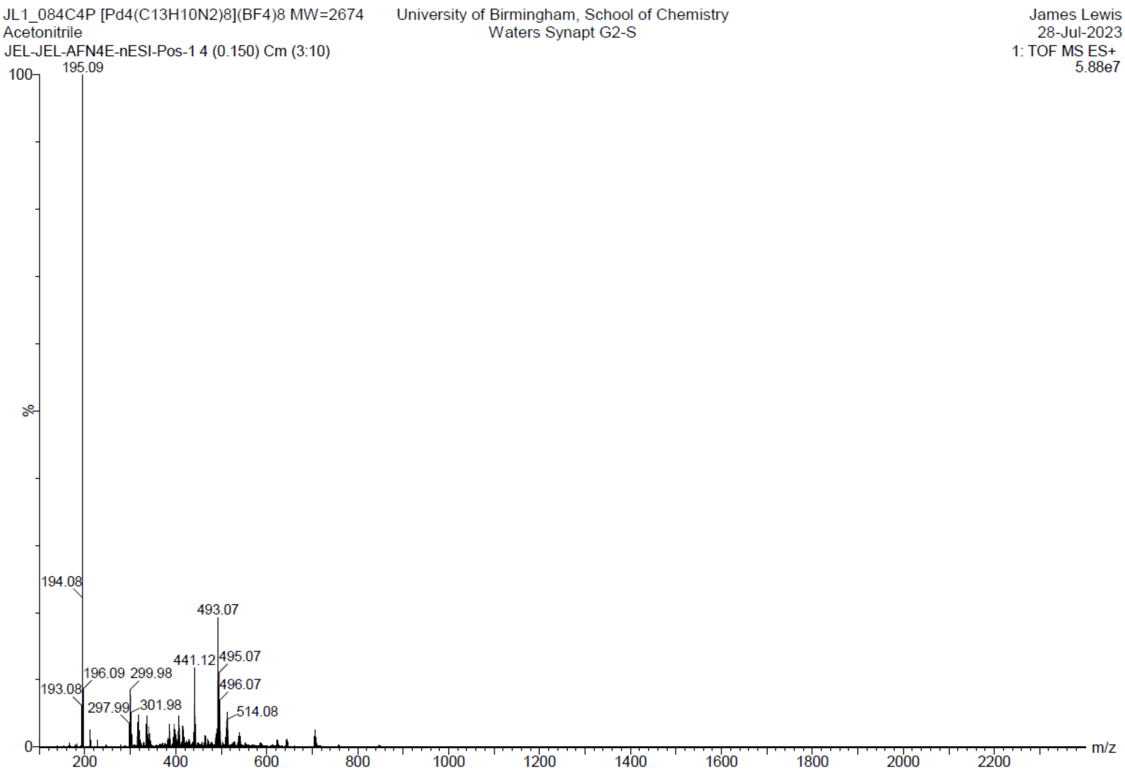


**Figure S204 ESI-MS of the equilibrated mixture of L4$^{P}$ and [Pd($CH_3CN$)$_4$]($BF_4$)$_2$.**

## S2.25 Synthesis of $[Pd_2(L2^H)_2(L4^Q)_2](BF_4)_4$

20 mM stock solutions in $CH_3CN$ of **L2^H** (500 µL, 10 µmol, 1 eq.) and **L4^Q** (500 µL, 10 µmol, 1 eq.) were combined in a vial and the solvent removed *in vacuo*. To the ligand mixture was added an 11 mM stock solution of $[Pd(CH_3CN)_4](BF_4)_2$ in $CD_3CN$ (1000 µL, 11 µmol, 1.1 eq.). A homogenous solution was obtained following sonication which was then transferred to a 5 mm NMR tube. After standing at 70 °C for 19 h the formation of two species in an approximately 3:1 ratio was observed by $^1$H NMR.

**ESI-MS** *m/z* = 440.40 $\{[Pd_2(\mathbf{L2^H})_2(\mathbf{L4^Q})_2](BF_4)\}^{3+}$ calc. 440.39; 670.09 $\{[Pd_2(\mathbf{L2^H})_2(\mathbf{L4^Q})_2](BF_4)F\}^{2+}$ calc. 670.09; 704.09 $\{[Pd_2(\mathbf{L2^H})_2(\mathbf{L4^Q})_2](BF_4)_2\}^{2+}$ calc. 704.09; 1494.19 $\{[Pd_2(\mathbf{L2^H})_2(\mathbf{L4^Q})_2](BF_4)_3\}^{+}$ calc. 1494.19.

**Diffusion coefficient** (400 MHz, $d_6$-DMSO) *D*: $6.35 \times 10^{-10}$ m$^2$s$^{-1}$.

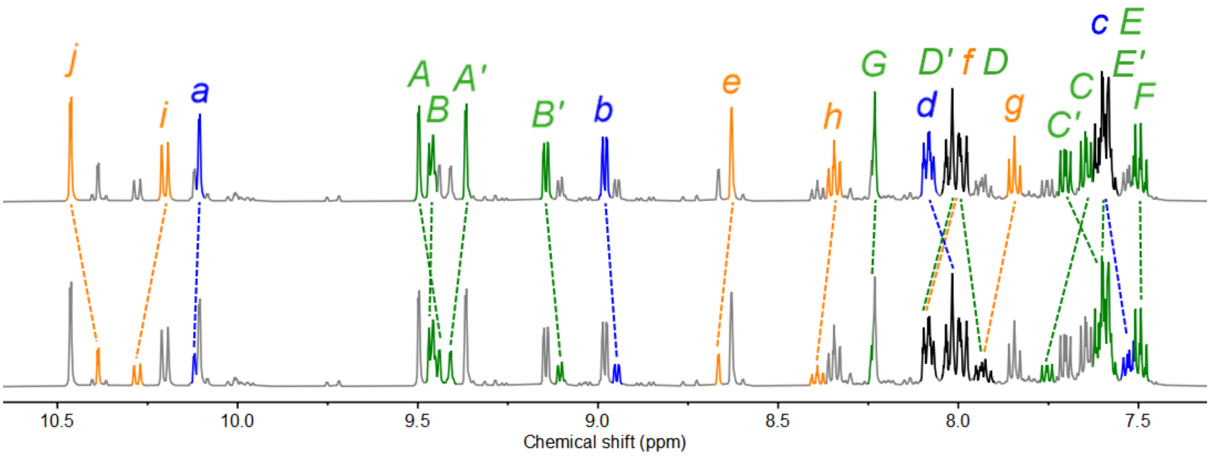


**Figure S205 Partial $^1$H NMR (500 MHz, $CD_3CN$) of $[Pd_2(L2^H)_2(L4^Q)_2](BF_4)_4$ with peaks labelled for the major (top) and minor (bottom) isomers.**

### Major isomer *syn*-$[Pd_2(L2^H)_2(L4^Q)_2](BF_4)_4$:

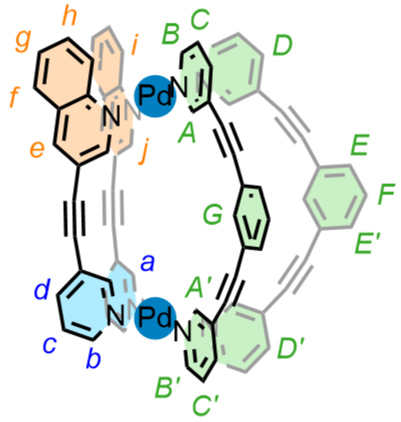


**$^1$H NMR** (500 MHz, $CD_3CN$) δ: 10.46 (d, *J* = 1.8 Hz, 2H, $H_j$), 10.20 (dd, *J* = 8.6, 0.9 Hz, 2H, $H_i$), 10.11 (d, *J* = 1.8 Hz, 2H, $H_a$), 9.50 (d, *J* = 1.8 Hz, 2H, $H_A$), 9.46 (d, *J* = 5.1 Hz, 2H, $H_B$), 9.37 (d, *J* = 1.8 Hz, 2H, $H_{A'}$), 9.14 (dd, *J* = 6.0, 1.4 Hz, 2H, $H_{B'}$), 8.98 (dd, *J* = 5.9, 1.4 Hz, 2H, $H_b$), 8.63 (s, 2H, $H_e$), 8.34 (ddd, *J* = 8.6, 7.1, 1.3 Hz, 2H, $H_h$), 8.23 (m, 2H, $H_G$), 8.08 (m, 2H,

$H_d$), 8.04-7.97 (m, 6H, $H_f$, $H_D$, $H_{D'}$), 7.84 (app. t, $J$ = 7.6 Hz, 2H, $H_G$), 7.70 (dd, $J$ = 8.0, 5.8 Hz, 2H, $H_{C'}$), 7.65 (dd, $J$ = 8.0, 6.0 Hz, 2H, $H_C$), 7.62-7.48 (m, 8H, $H_c$, $H_E$, $H_{E'}$, $H_F$).

**$^{13}$C NMR** (126 MHz, $CD_3CN$) δ: 156.5 ($C_j$), 155.3 ($C_A$/$C_{A'}$), 154.9 ($C_A$/$C_{A'}$), 154.5 ($C_a$), 152.1 ($C_b$), 151.0 ($C_B$), 150.3 ($C_{B'}$), 146.2 (4°), 144.2 ($C_e$), 143.0 ($C_D$/$C_{D'}$), 142.9 ($C_d$), 142.4 ($C_D$/$C_{D'}$), 139.3 ($C_G$), 135.7 ($C_h$), 132.6 ($C_E$/$C_{E'}$), 132.4 ($C_E$/$C_{E'}$), 131.1 (×2, $C_f$, $C_g$), 130.8 ($C_F$), 130.2 (4°), 128.9 ($C_C$/$C_{C'}$), 128.8 ($C_C$/$C_{C'}$), 128.4 ($C_c$), 127.4 ($C_i$), 125.2 (4°), 124.8 (4°), 124.2 (4°), 123.1 (4°), 123.0 (4°), 119.2 (4°), 95.5 (C≡C), 95.4 (C≡C), 91.0 (C≡C), 89.9 (C≡C), 86.1 (C≡C), 85.7 (C≡C).

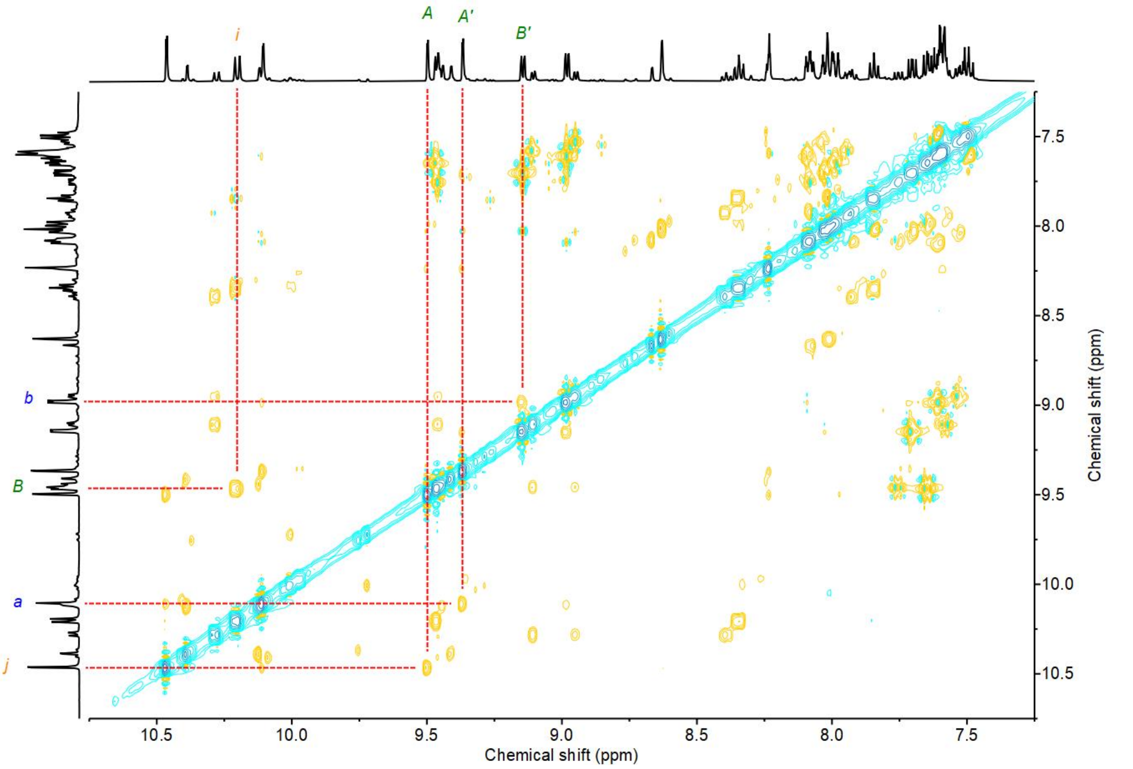


**Figure S206 Partial NOESY (500 MHz, $CD_3CN$) of $[Pd_2(L2^H)_2(L4^Q)_2](BF_4)_4$ labelled for major isomer with through-space interactions used to identify it as the *syn* isomer. The lack of interactions between the two ends of $L2^H$ (i.e. *A*···*A'*, *B*···*B'*), $L4^Q$ (i.e. *b*···*i*) demonstrate that both $L4^Q$ ligands are arranged in the same orientation.**

**Minor isomer *anti*-$[Pd_2(L2^H)_2(L4^Q)_2](BF_4)_4$:**

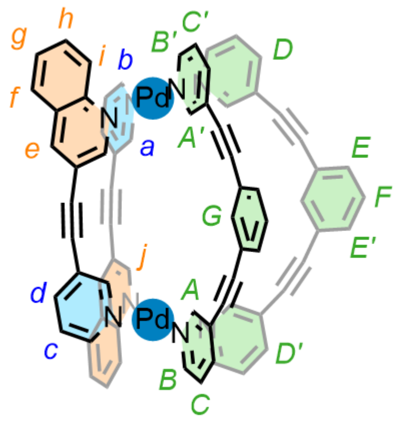


**$^1$H NMR** (500 MHz, $CD_3CN$) δ: 10.39 (d, $J$ = 1.7 Hz, 2H, $H_j$), 10.28 (d, $J$ = 8.6 Hz, 2H, $H_i$), 10.12 (d, $J$ = 1.7 Hz, 2H, $H_a$), 9.46 (m, 2H, $H_B$), 9.44 (d, $J$ = 1.7 Hz, 2H, $H_A$), 9.41 (d, $J$ = 1.7 Hz, 2H, $H_{A'}$), 9.11 (m, 2H, $H_{B'}$), 8.95 (m, 2H, $H_b$), 8.66 (s, 2H, $H_e$), 8.39 (ddd, $J$ = 8.6, 7.1, 1.4 Hz, 2H, $H_h$), 8.24 (m, 2H, $H_G$), 8.10-8.07 (m, 4H, $H_D$, $H_f$), 7.96-7.91 (m, 4H, $H_g$, $H_{D'}$), 7.75 (ddd, $J$ = 8.2, 5.9, 0.7 Hz, 2H, $H_C$), 7.62-7.56 (m, 6H, $H_{C'}$, $H_E$, $H_{E'}$), 7.54-7.48 (m, 4H, $H_c$, $H_F$).

**$^{13}$C NMR** (126 MHz, $CD_3CN$) δ: 156.4, 155.1 ($C_A$/$C_{A'}$), 154.8 ($C_A$/$C_{A'}$), 154.5 ($C_a$), 152.0 ($C_b$), 150.8 ($C_B$), 150.7 ($C_{B'}$), 146.3 (4°), 144.1 ($C_e$), 143.0, 142.9, 142.6, 139.4 ($C_G$), 136.0 ($C_h$), 132.5, 132.4, 131.2, 131.1, 130.1 (4°), 128.9, 128.6, 127.5 ($C_i$), 125.1 (4°), 125.0 (4°), 124.5 (4°), 123.1 (4°), 123.0 (4°), 119.1 (4°), 95.6 (C≡C), 95.4 (C≡C), 91.3 (C≡C), 90.0 (C≡C), 86.0 (C≡C), 85.9 (C≡C). 2 signals missing due to overlap with major isomer peaks.

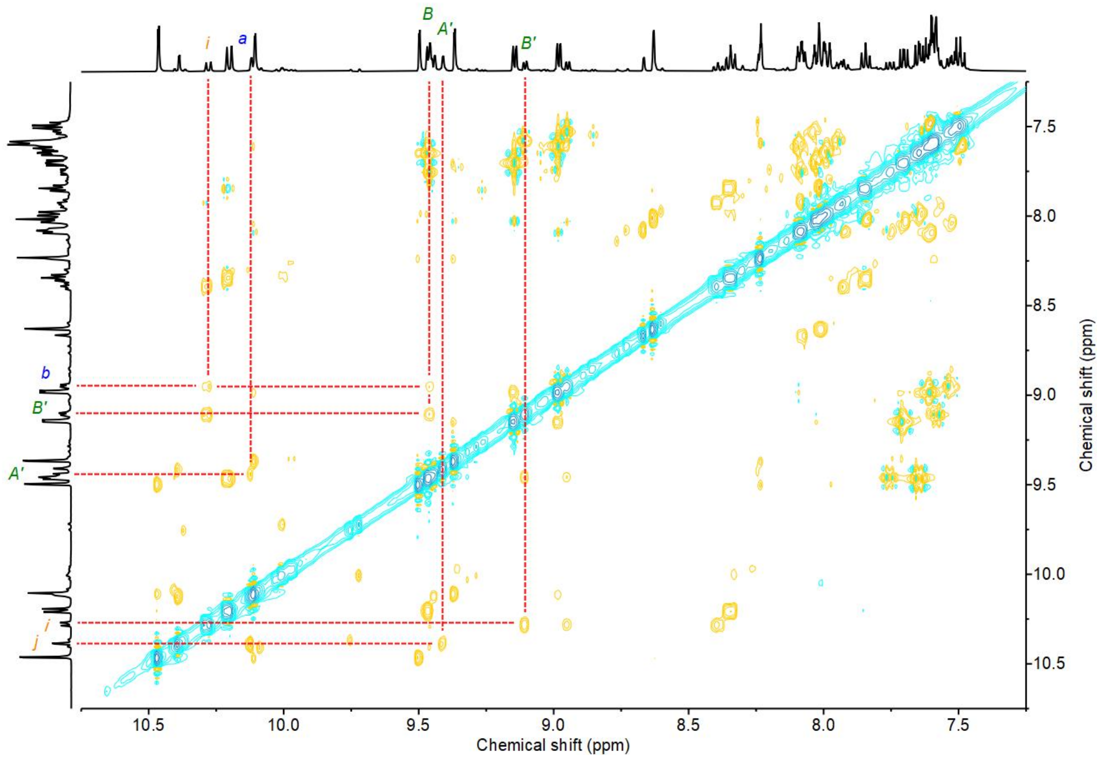


**Figure S207 Partial NOESY (500 MHz, $CD_3CN$) of $[Pd_2(L2^H)_2(L4^Q)_2](BF_4)_4$ labelled for minor isomer with through-space interactions used to identify it as the *anti* isomer. Importantly, interactions *B*···*B'* and *b*···*i* are seen that were not for the major isomer. *A*···*A'* cannot be observed due to the close proximity of these signals (Δδ = 0.03 ppm).**

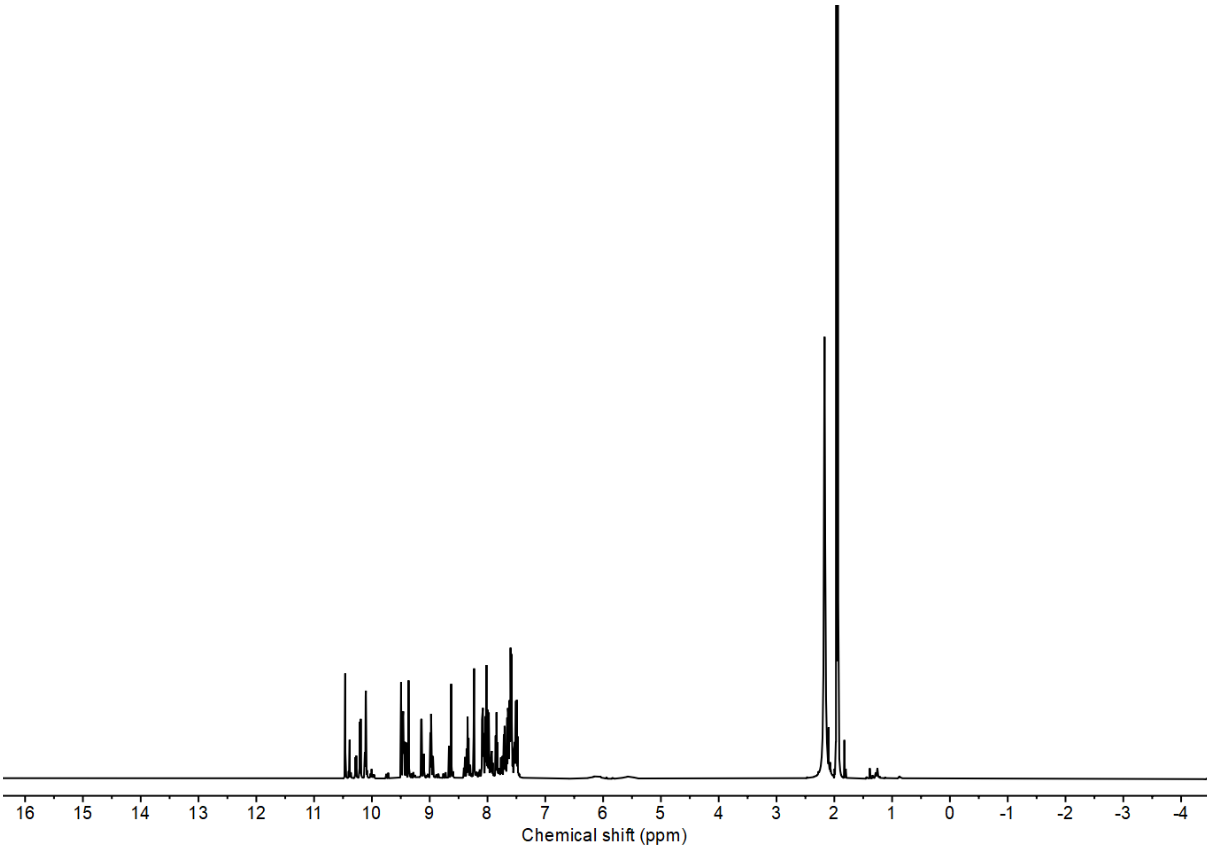


**Figure S208 $^{1}H$ NMR (500 MHz, $d_6$-DMSO) of $[Pd_2(L2^H)_2(L4^Q)_2](BF_4)_4$.**

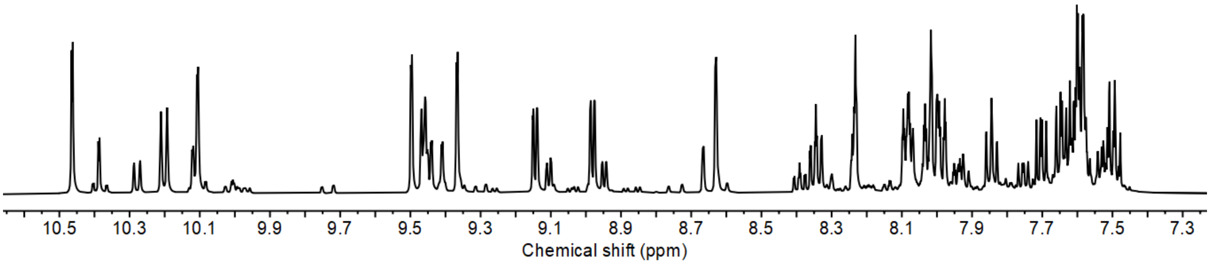


**Figure S209 Partial $^{1}H$ NMR (500 MHz, $d_6$-DMSO) of $[Pd_2(L2^H)_2(L4^Q)_2](BF_4)_4$.**

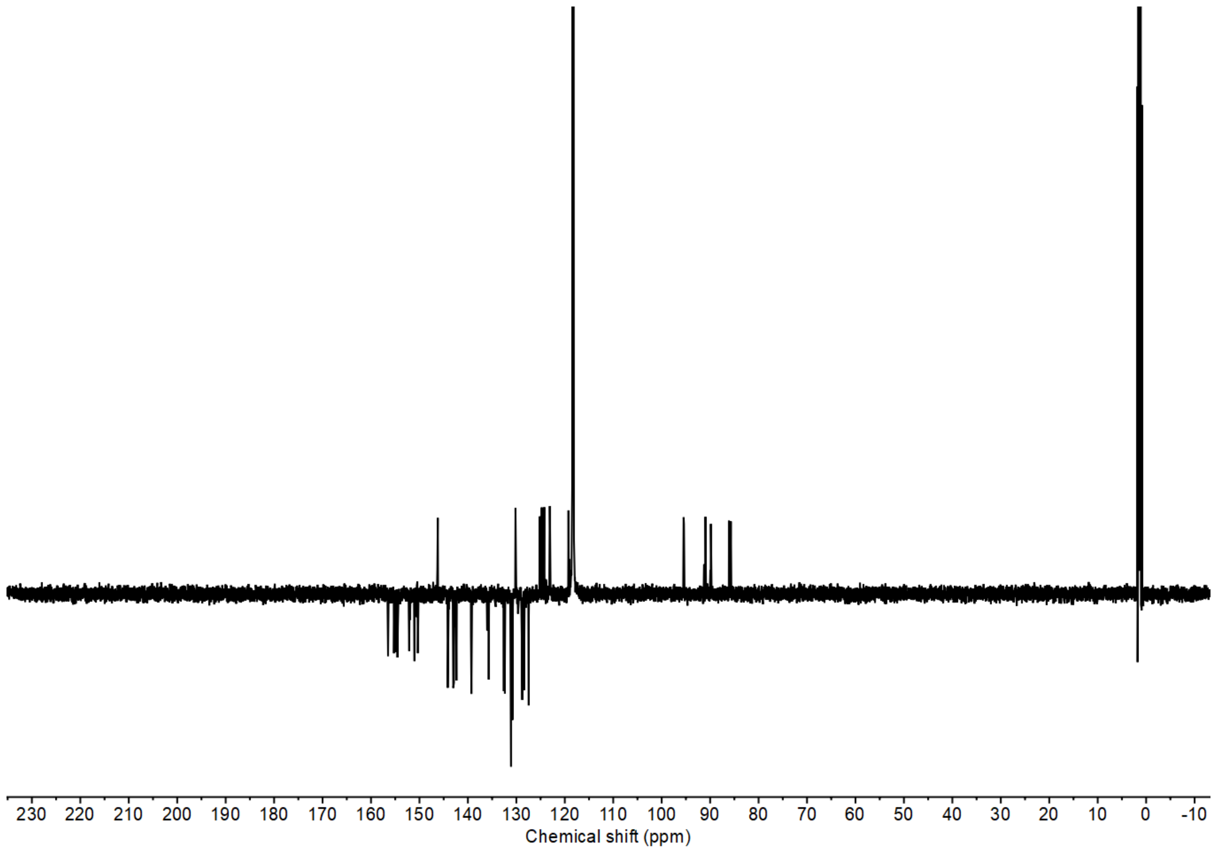


**Figure S210 JMOD $^{13}C$ NMR (126 MHz, $d_6$-DMSO) of $[Pd_2(L2^H)_2(L4^Q)_2](BF_4)_4$.**

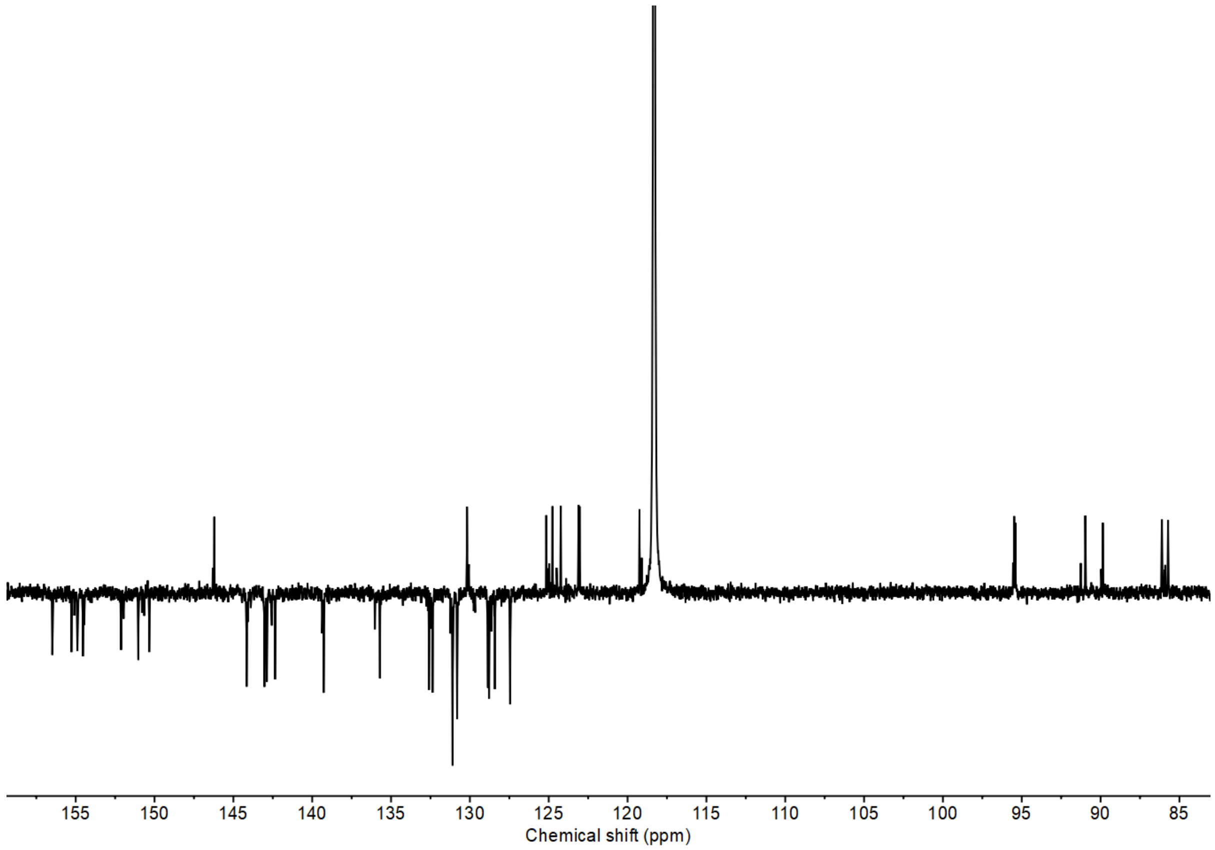


**Figure S211 Partial JMOD $^{13}C$ NMR (126 MHz, $d_6$-DMSO) of $[Pd_2(L2^H)_2(L4^Q)_2](BF_4)_4$.**

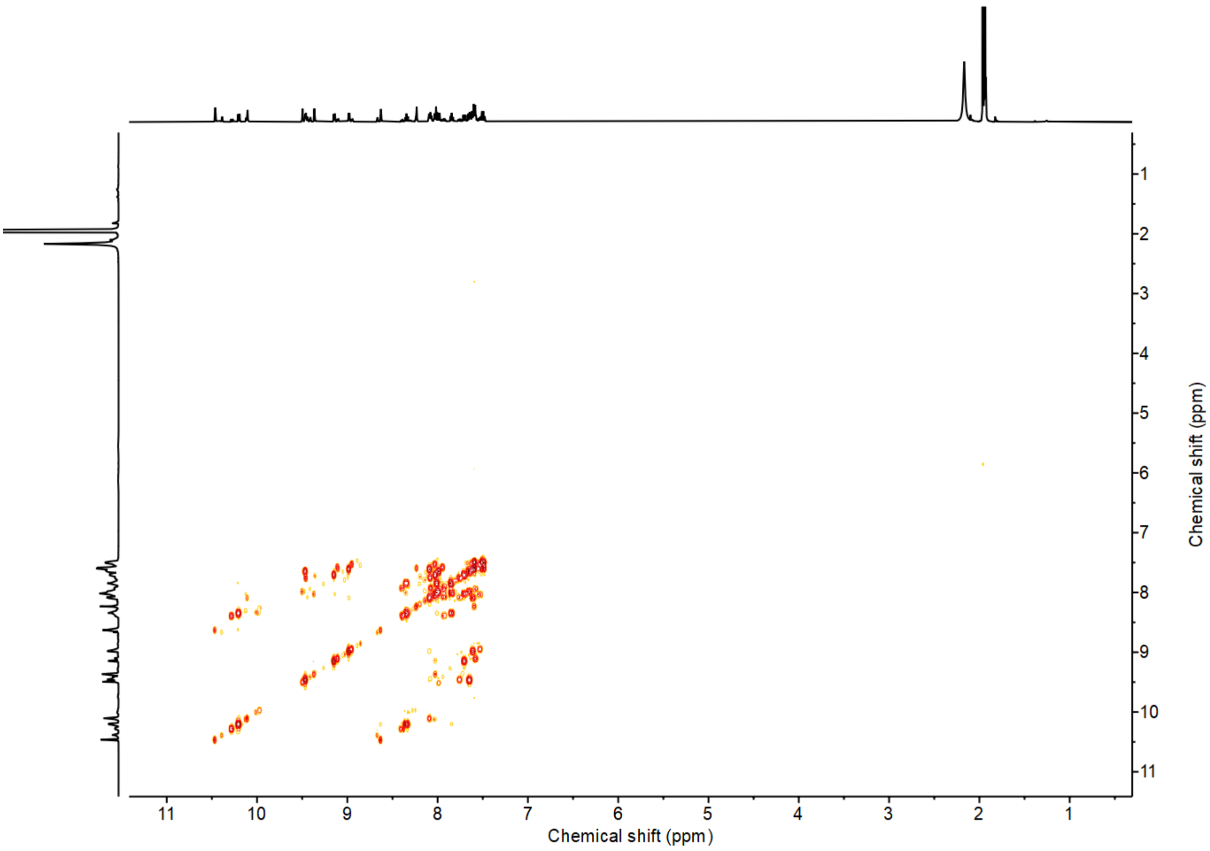


**Figure S212 COSY ($d_6$-DMSO) of [$Pd_2(L2^H)_2(L4^Q)_2$]$(BF_4)_4$.**

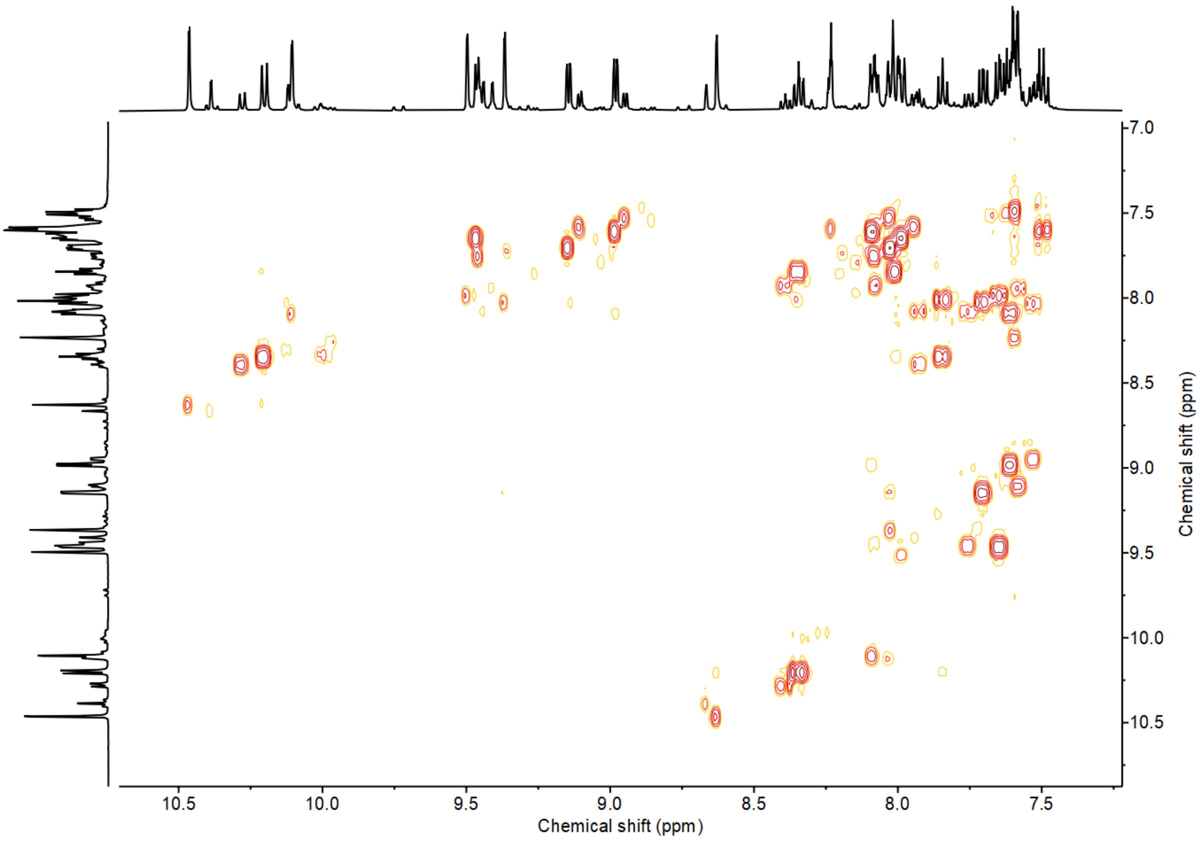


**Figure S213 Partial COSY ($d_6$-DMSO) of [$Pd_2(L2^H)_2(L4^Q)_2$]$(BF_4)_4$ with diagonal suppression.**

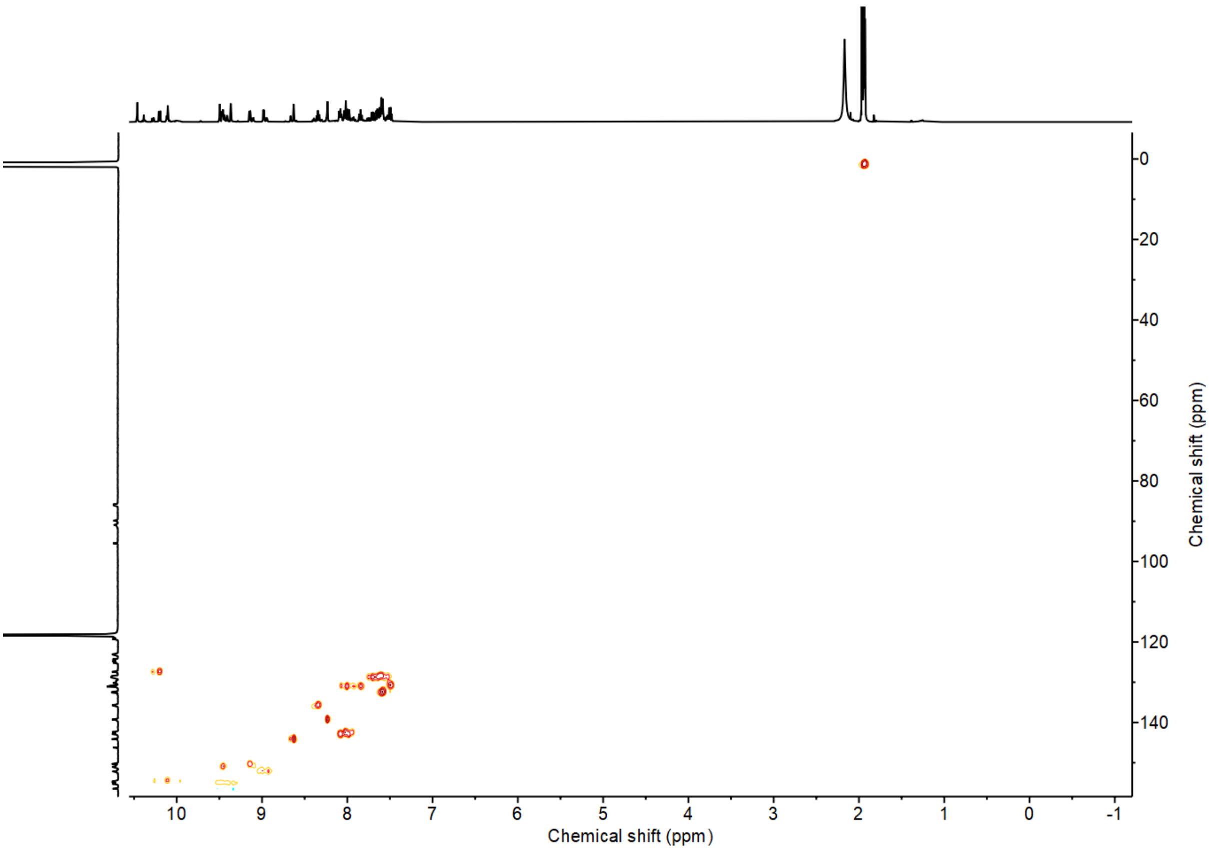


**Figure S214 HSQC ($d_6$-DMSO) of $[Pd_2(L2^H)_2(L4^Q)_2](BF_4)_4$.**

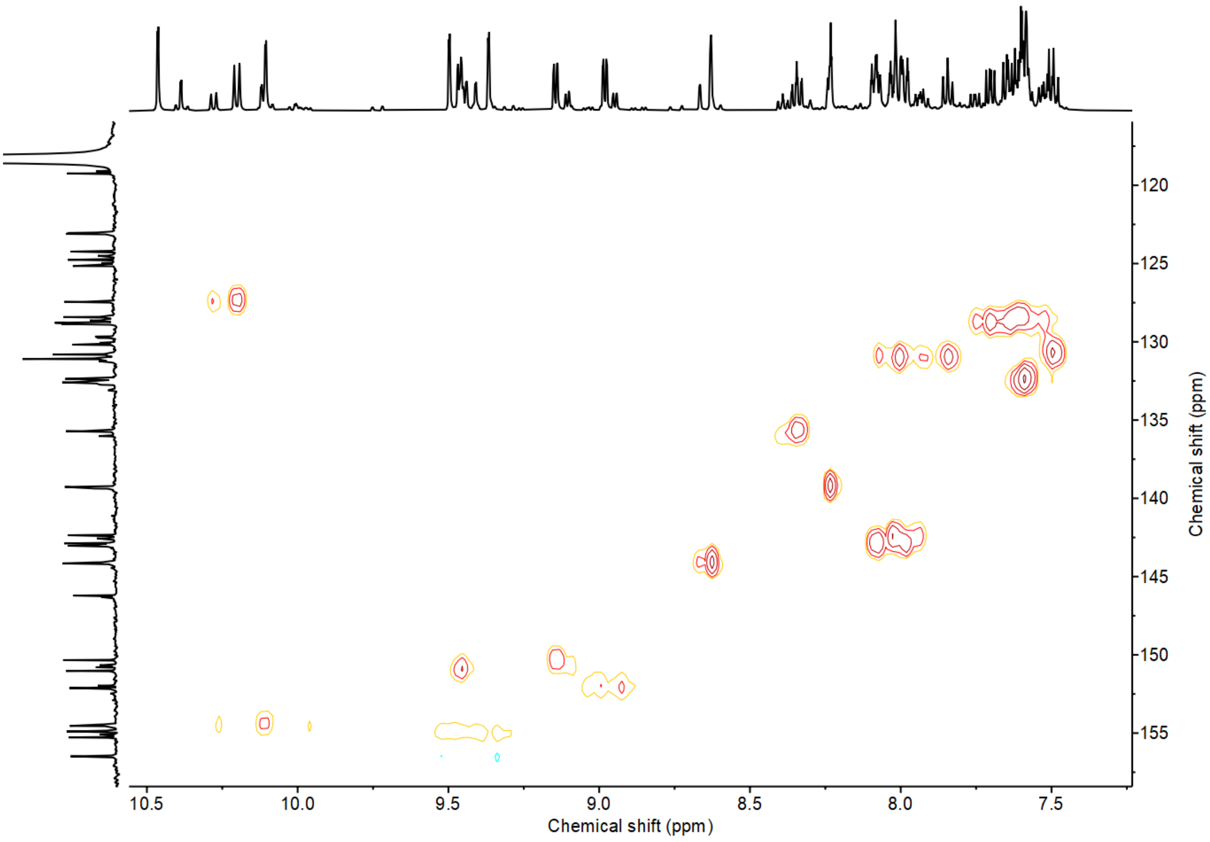


**Figure S215 Partial HSQC ($d_6$-DMSO) of $[Pd_2(L2^H)_2(L4^Q)_2](BF_4)_4$.**

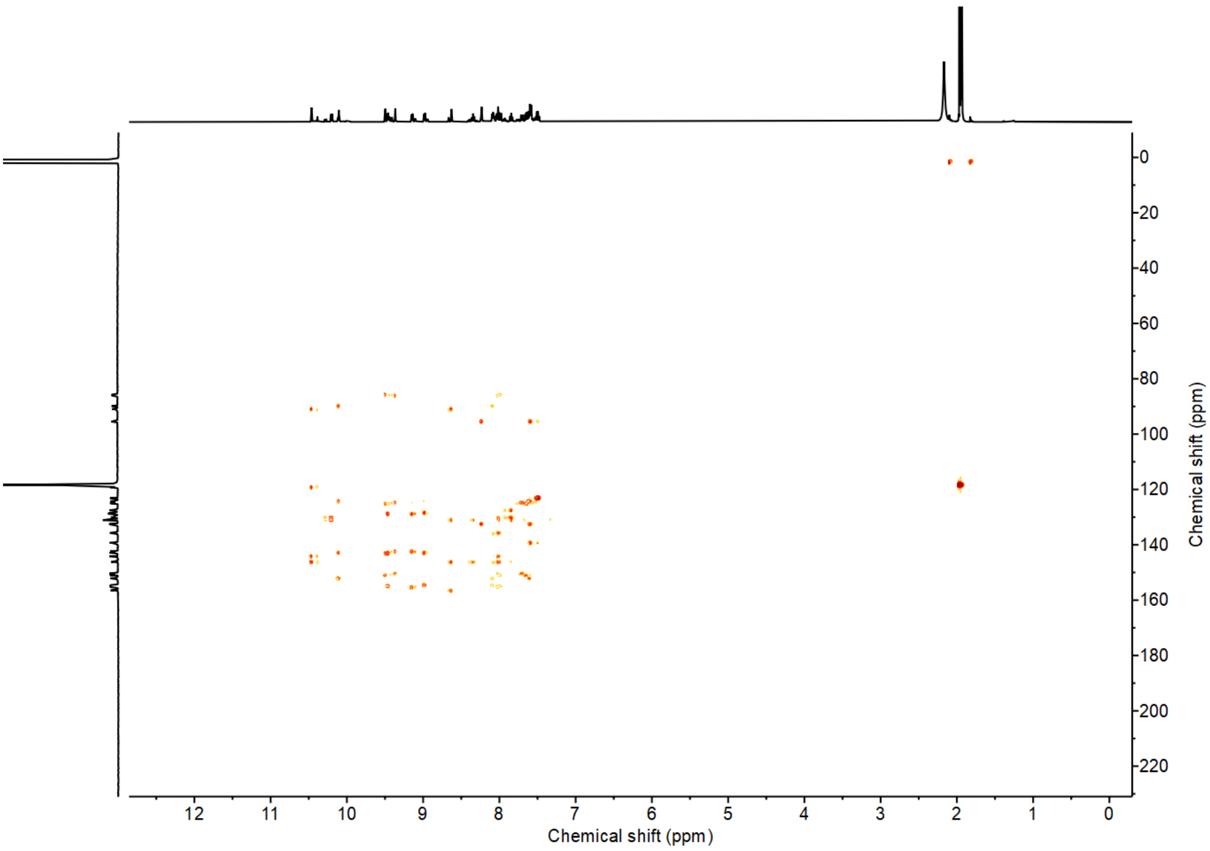


**Figure S216 HMBC ($d_6$-DMSO) of $[Pd_2(L2^H)_2(L4^Q)_2](BF_4)_4$.**

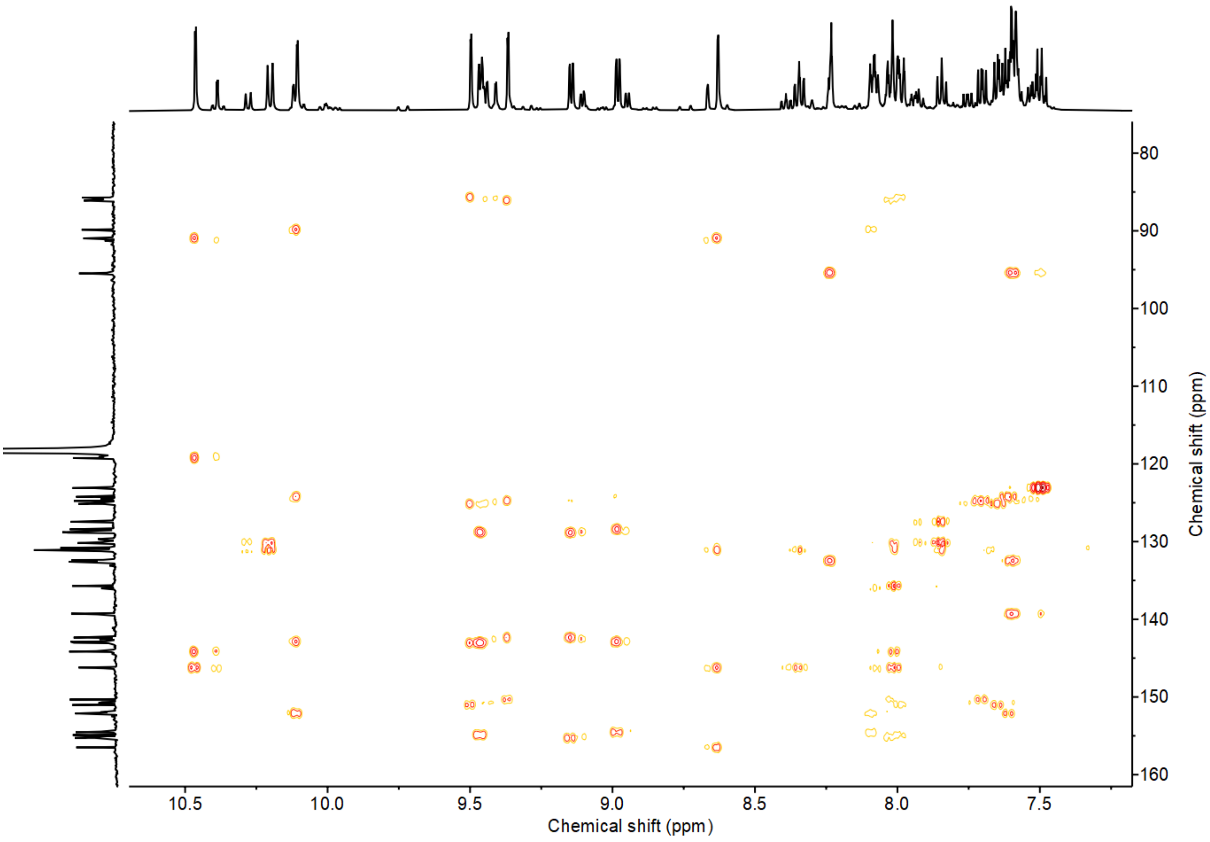


**Figure S217 Partial HMBC ($d_6$-DMSO) of $[Pd_2(L2^H)_2(L4^Q)_2](BF_4)_4$.**

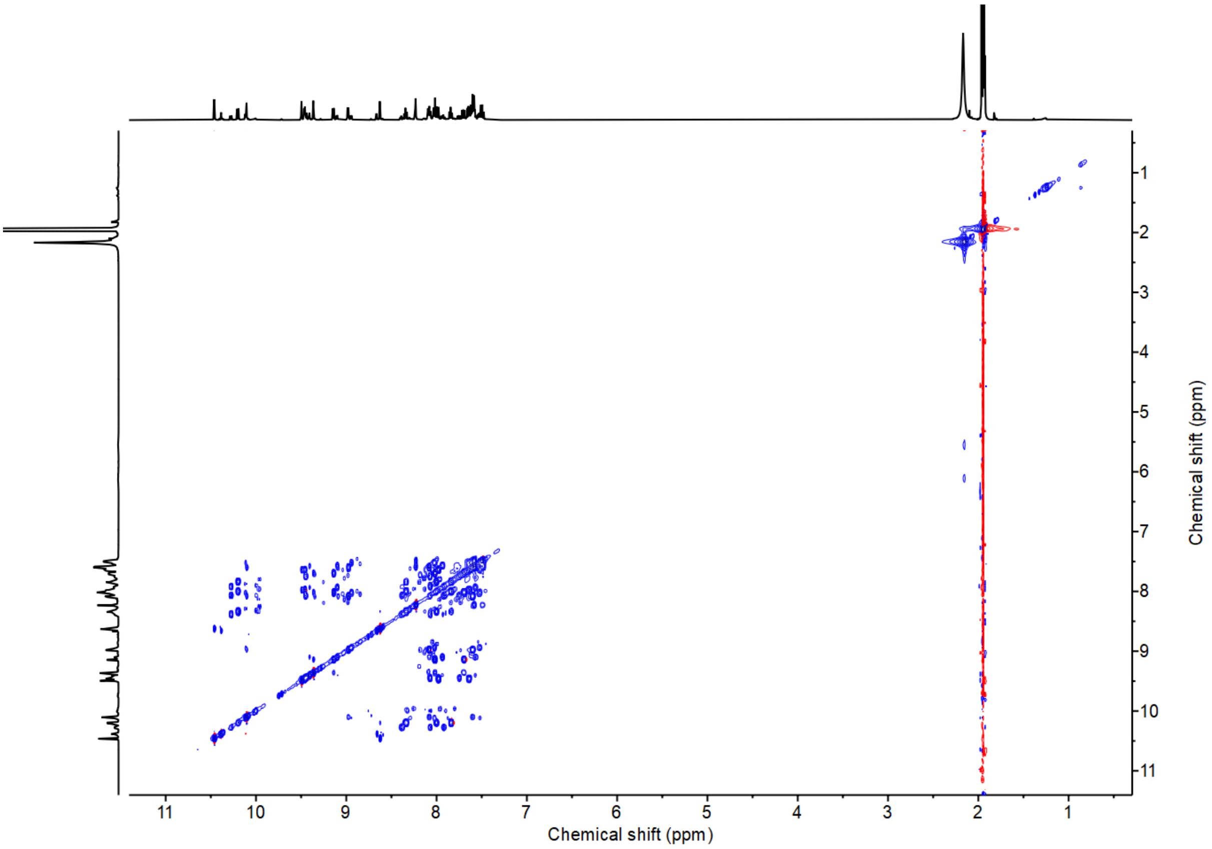


**Figure S218 TOCSY ($d_6$-DMSO) of $[Pd_2(L2^H)_2(L4^Q)_2](BF_4)_4$.**

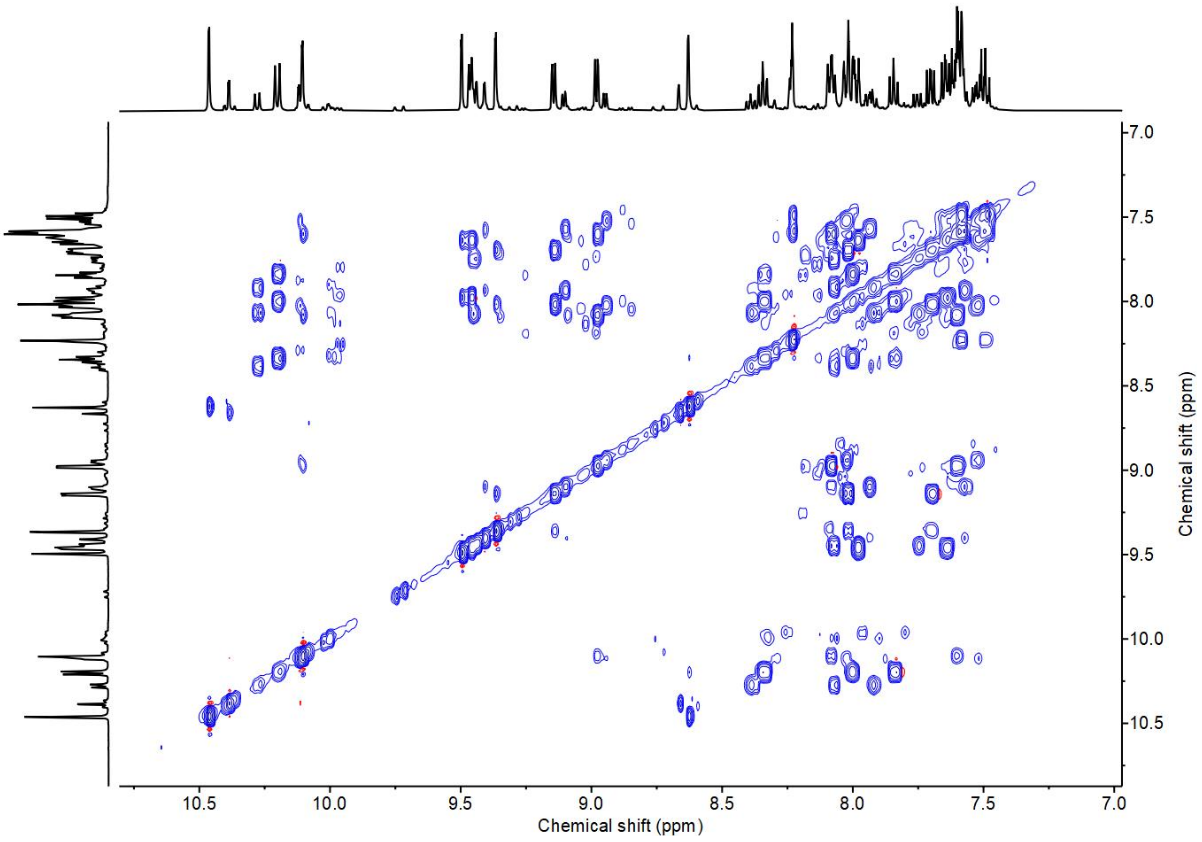


**Figure S219 Partial TOCSY ($d_6$-DMSO) of $[Pd_2(L2^H)_2(L4^Q)_2](BF_4)_4$.**

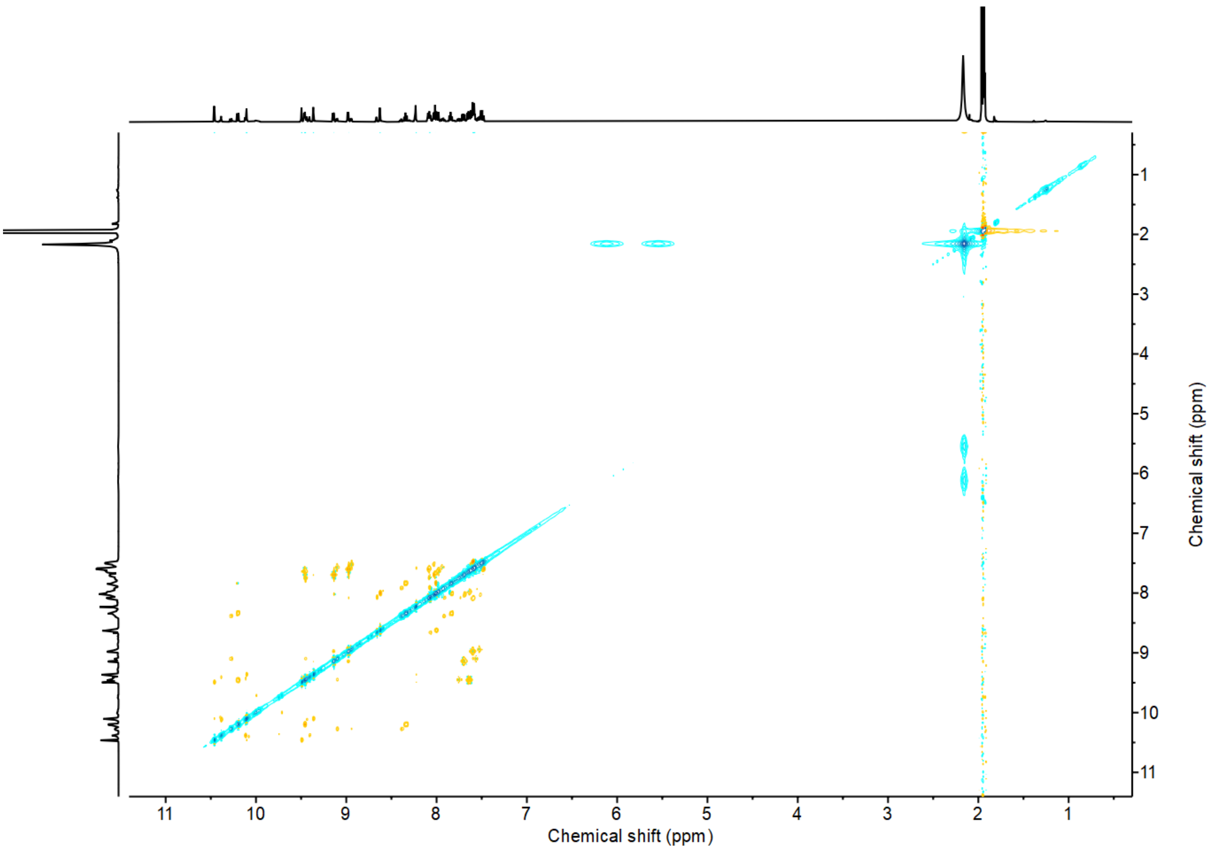


**Figure S220 NOESY ($d_6$-DMSO) of [Pd$_2$(L2$^H$)$_2$(L4$^Q$)$_2$](BF$_4$)$_4$.**

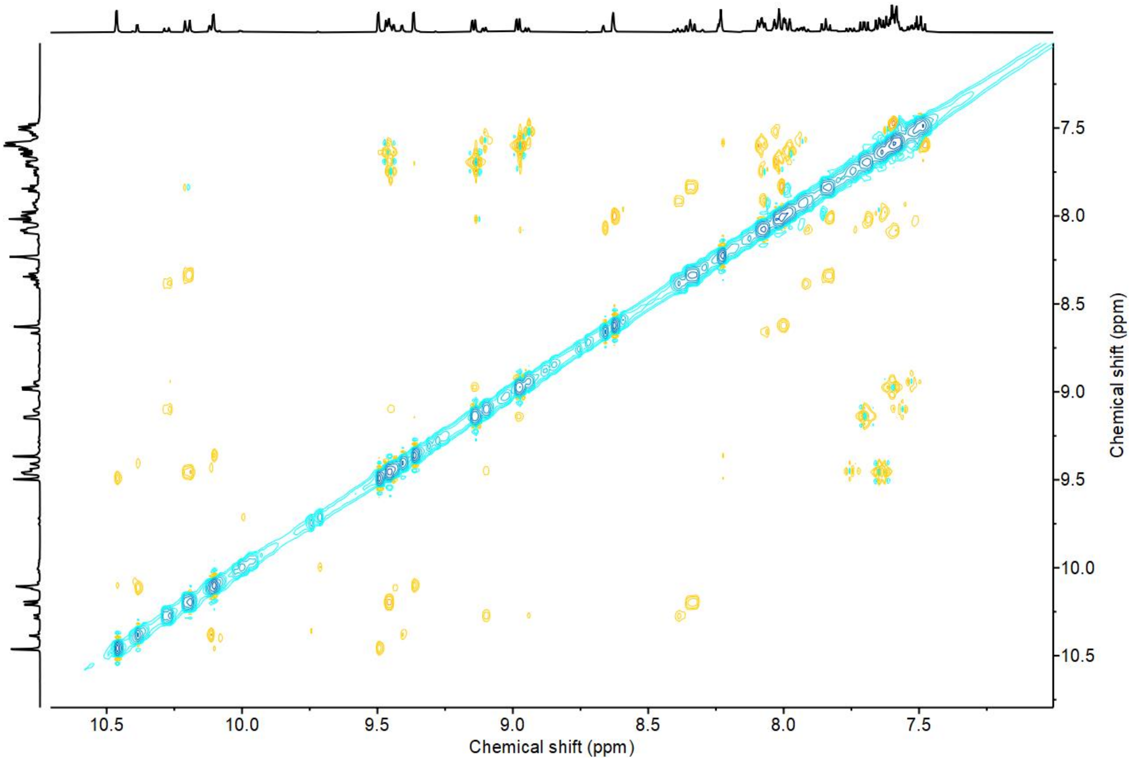


**Figure S221 Partial NOESY ($d_6$-DMSO) of [Pd$_2$(L2$^H$)$_2$(L4$^Q$)$_2$](BF$_4$)$_4$.**

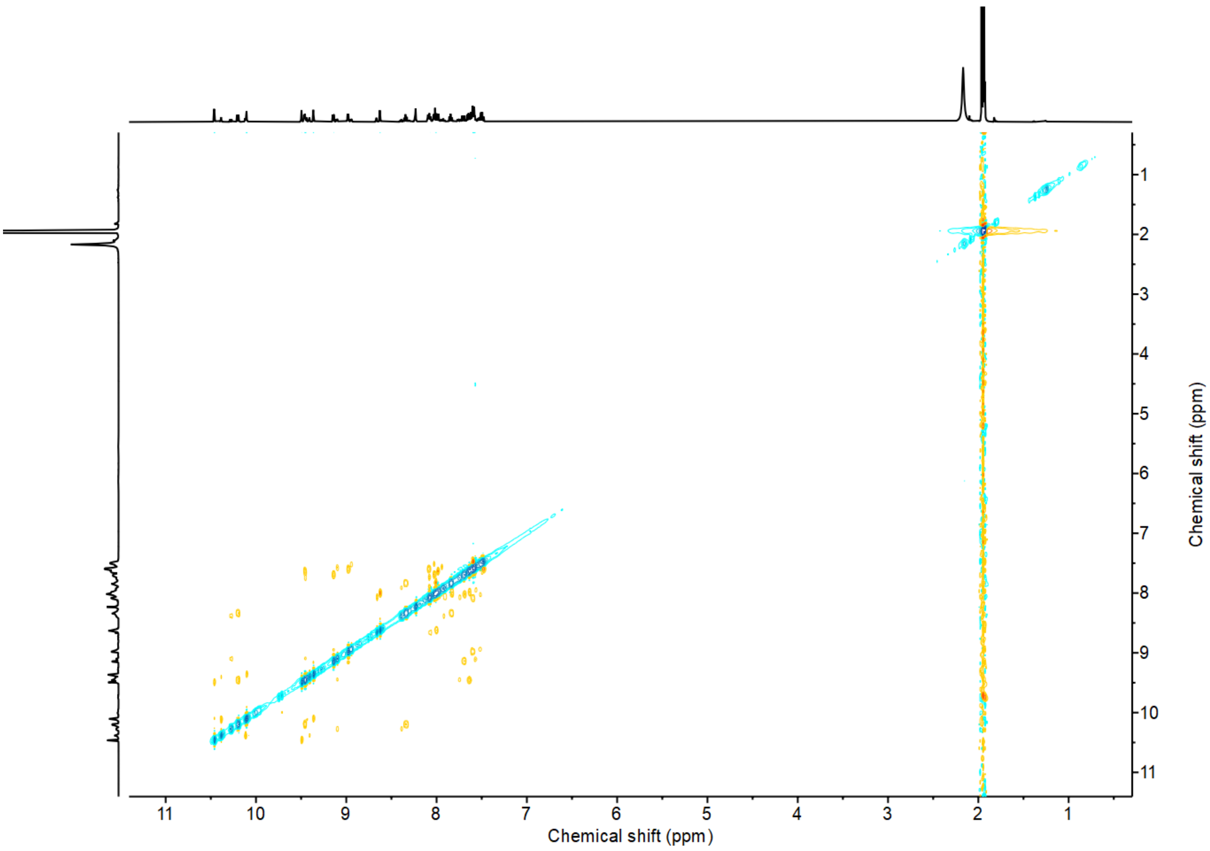


**Figure S222 ROESY ($d_6$-DMSO) of $[Pd_2(L2^H)_2(L4^Q)_2](BF_4)_4$.**

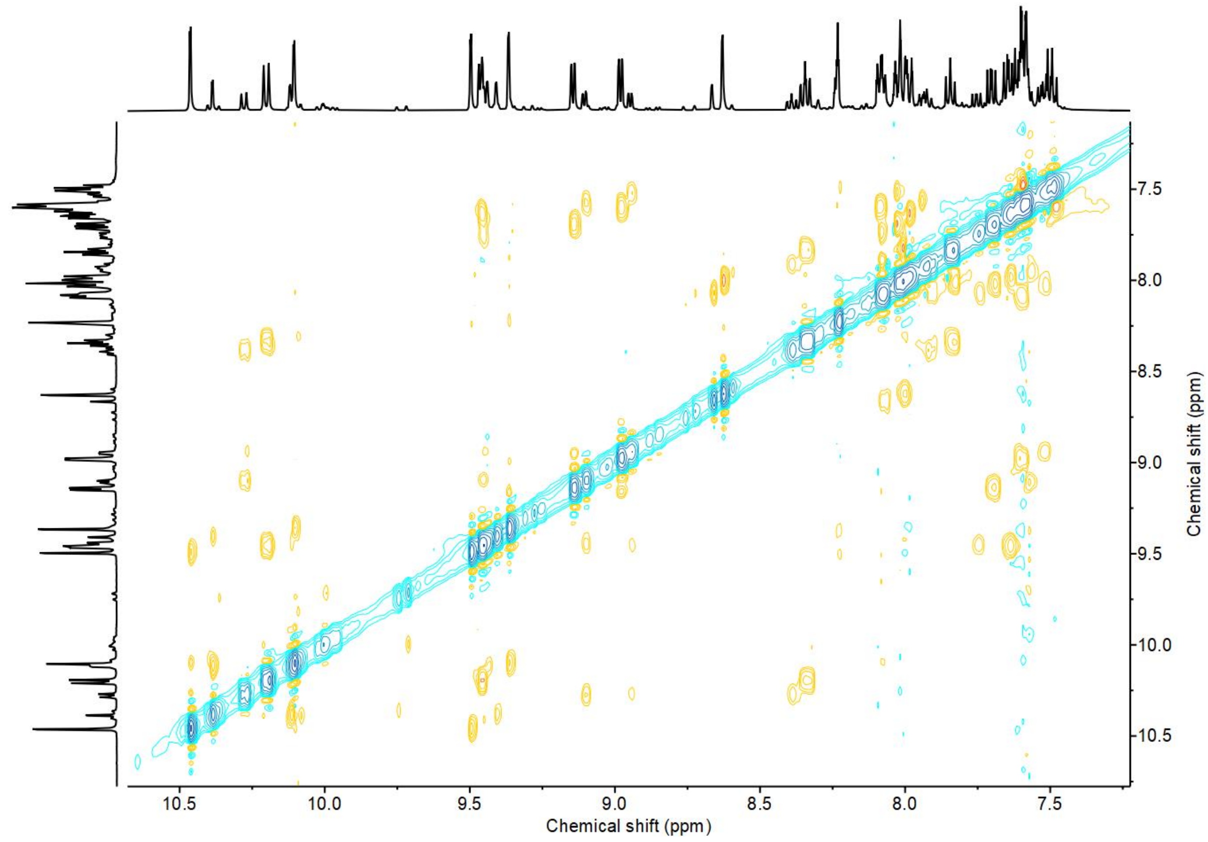


**Figure S223 Partial ROESY ($d_6$-DMSO) of $[Pd_2(L2^H)_2(L4^Q)_2](BF_4)_4$.**

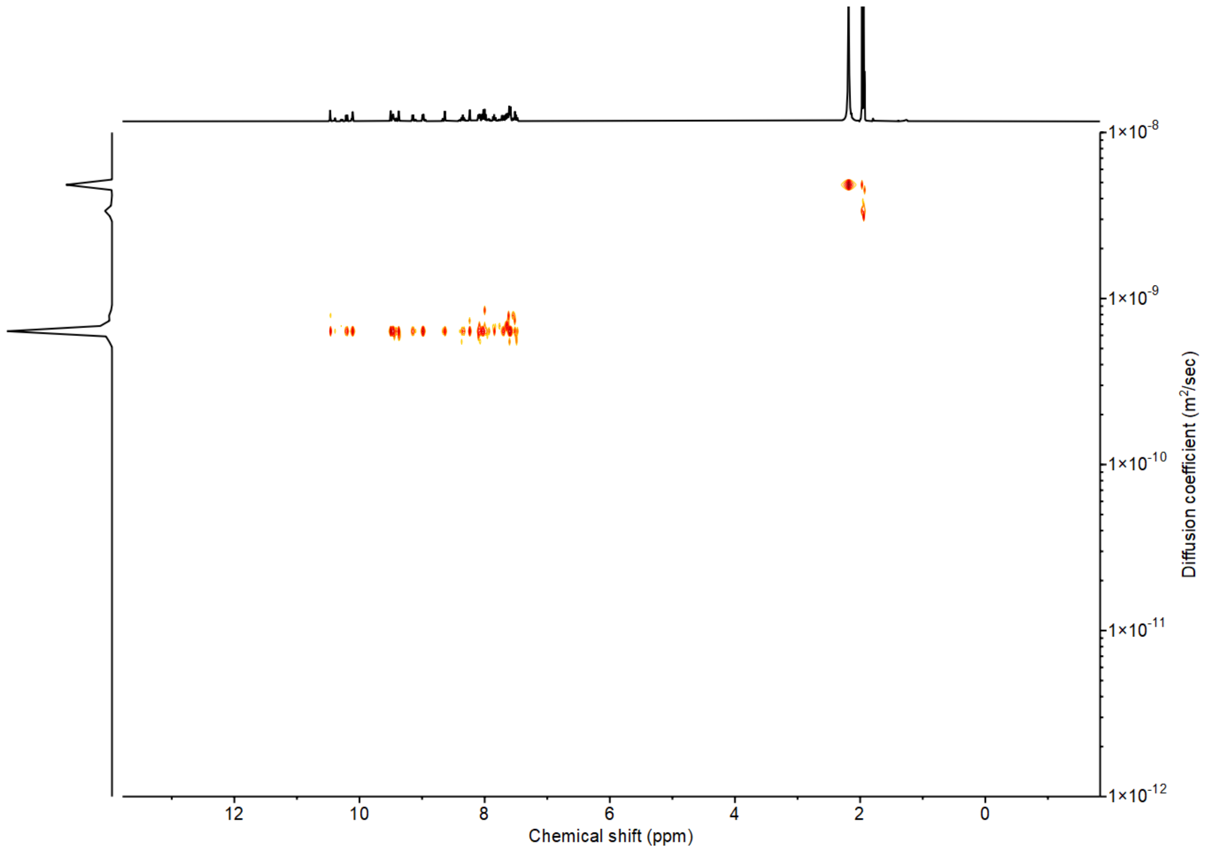


**Figure S224 DOSY (400 MHz, $CD_3CN$) of $[Pd_2(L2^H)_2(L4^Q)_2](BF_4)_4$.**

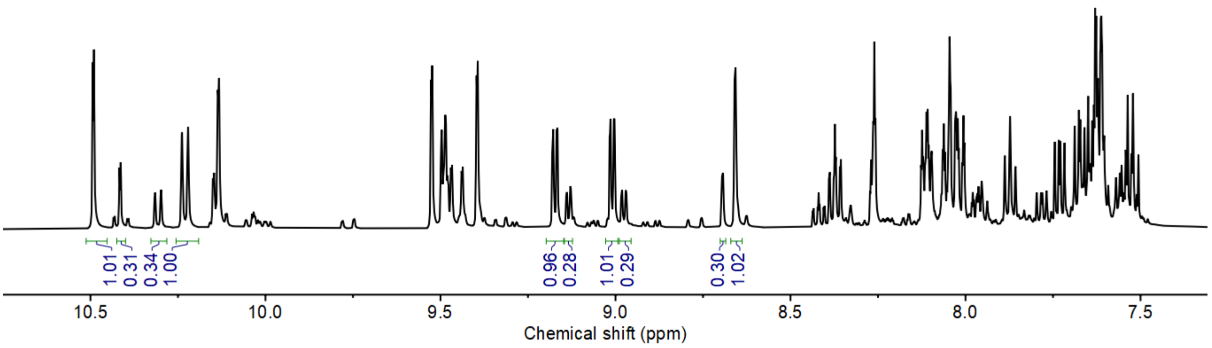


**Figure S225 Partial $^1H$ NMR (500 MHz, $CD_3CN$) showing relative integrations of selected signals for the major and minor isomers of $[Pd_2(L2^H)_2(L4^Q)_2](BF_4)_4$.**

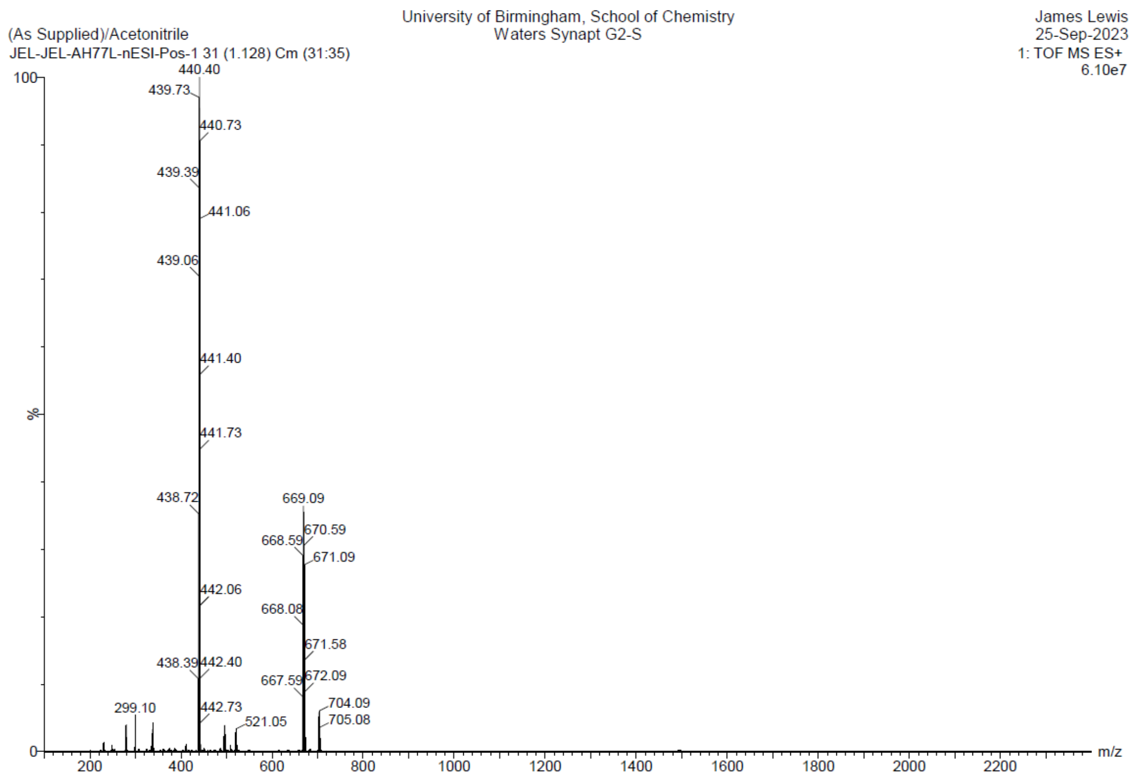


**Figure S226 ESI-MS of $[Pd_2(L2^H)_2(L4^Q)_2](BF_4)_4$.**

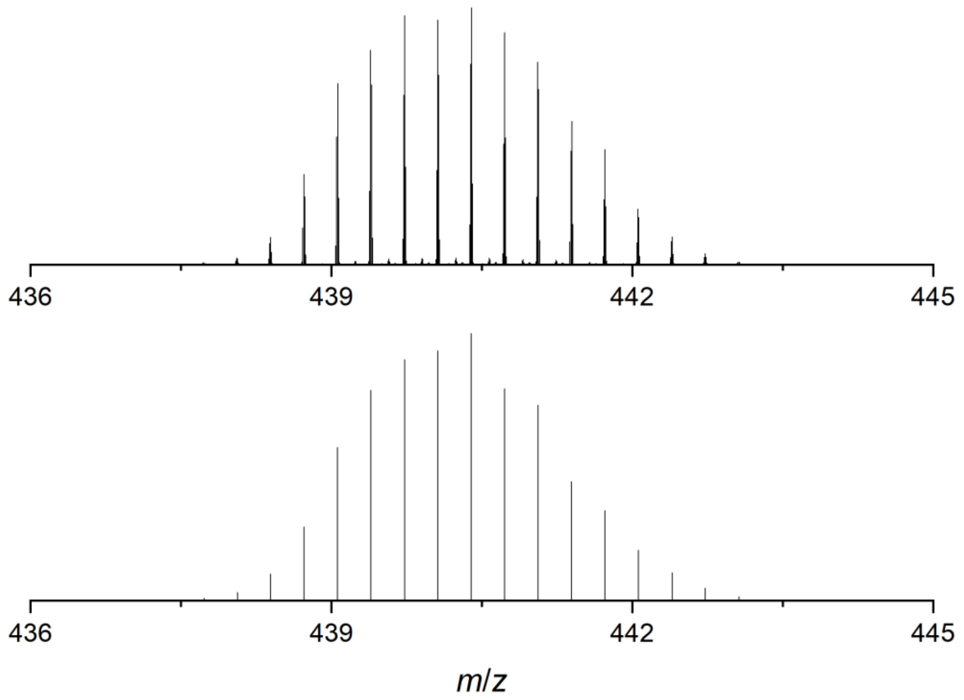


**Figure S227 Observed (top) and calculated (bottom) isotopic patterns for $\{[Pd_2(L2^H)_2(L4^Q)_2](BF_4)\}^{3+}$.**

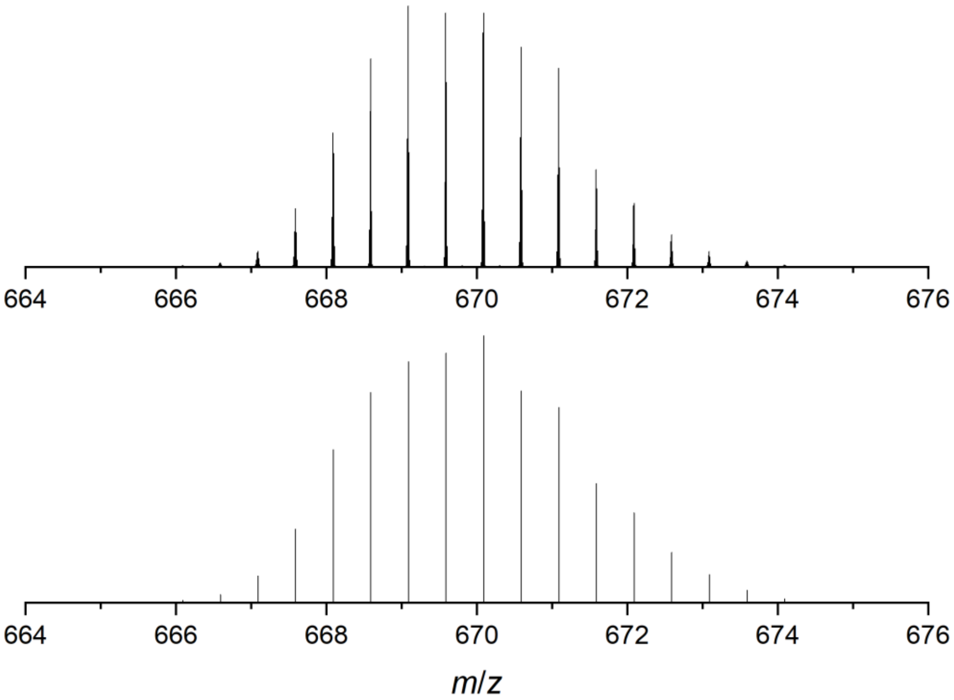


**Figure S228 Observed (top) and calculated (bottom) isotopic patterns for {[$Pd_2(L2^H)_2(L4^Q)_2$]($BF_4$)F}$^{2+}$.**

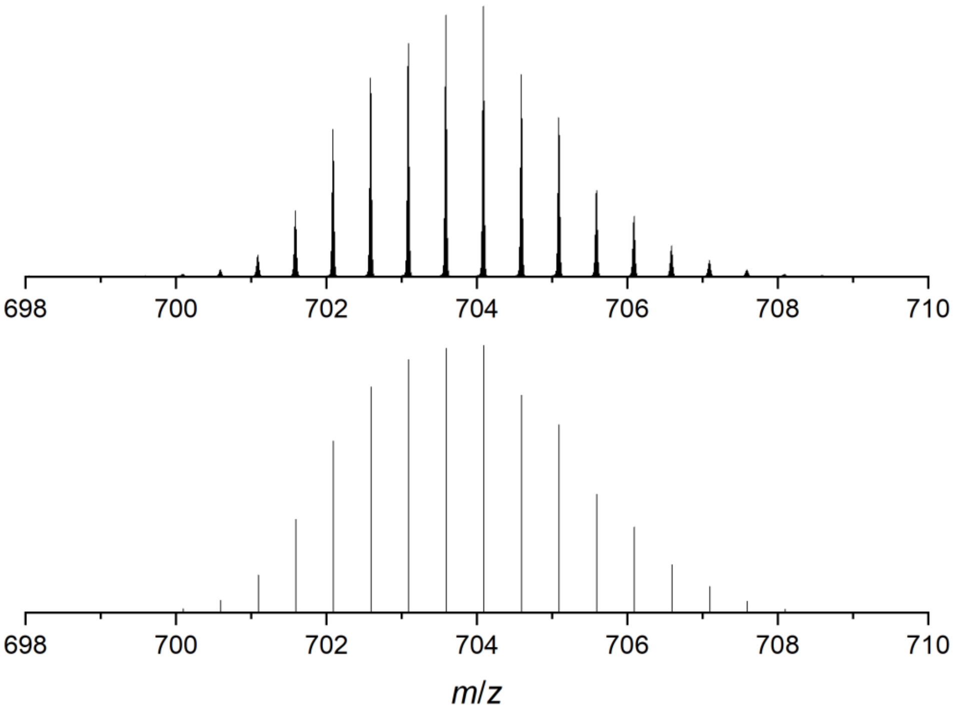


**Figure S229 Observed (top) and calculated (bottom) isotopic patterns for {[$Pd_2(L2^H)_2(L4^Q)_2$]($BF_4$)$_2$}$^{2+}$.**

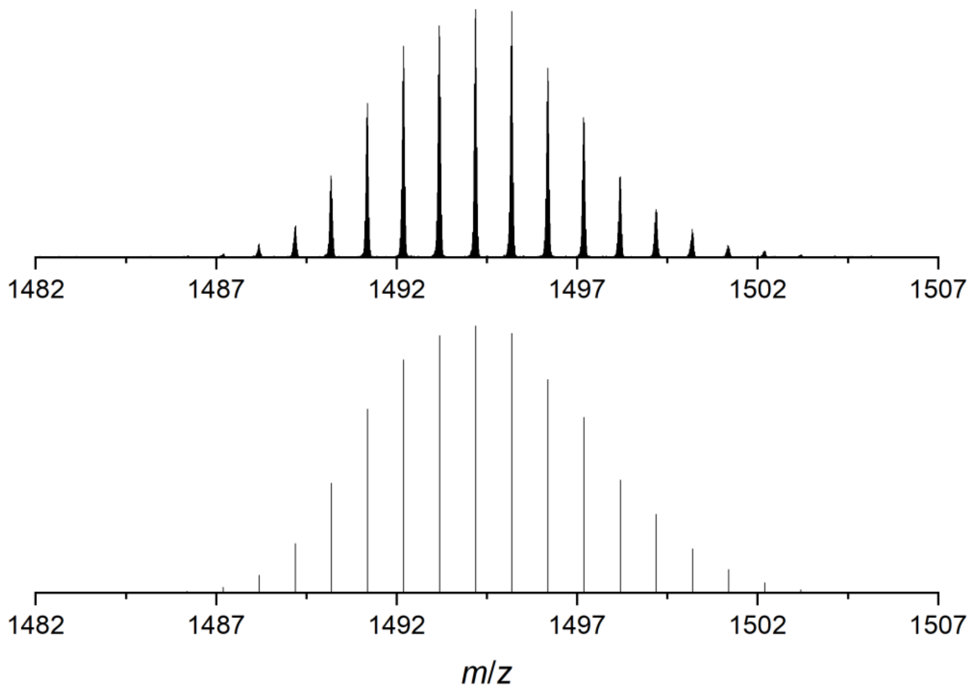


**Figure S230 Observed (top) and calculated (bottom) isotopic patterns for $\{[Pd_2(L2^H)_2(L4^Q)_2](BF_4)_3\}^+$.**

## S2.26 Synthesis of $[Pd_2(L2^H)_2(L4^P)_2](BF_4)_4$

20 mM stock solutions in $CH_3CN$ of **$L2^H$** (500 µL, 10 µmol, 1 eq.) and **$L4^P$** (500 µL, 10 µmol, 1 eq.) were combined in a vial and the solvent removed *in vacuo*. To the ligand mixture was added an 11 mM stock solution of $[Pd(CH_3CN)_4](BF_4)_2$ in $CD_3CN$ (1000 µL, 11 µmol, 1.1 eq.). A homogenous solution was obtained following sonication which was then transferred to a 5 mm NMR tube. After standing at 70 °C no further changes were observed by $^1H$ NMR.

**ESI-MS** *m/z* = 416.39 $\{[Pd_2(\mathbf{L2^H})_2(\mathbf{L4^P})_2](BF_4)\}^{3+}$ calc. 416.39; 634.09 $\{[Pd_2(\mathbf{L2^H})_2(\mathbf{L4^P})_2](BF_4)F\}^{2+}$ calc. 634.09; 668.09 $\{[Pd_2(\mathbf{L2^H})_2(\mathbf{L4^P})_2](BF_4)_2\}^{2+}$ calc. 668.09. Minor signals for the homoleptic $[Pd_2(\mathbf{L2^H})_4]^{4+}$ cage were also observed: 473.74 $\{[Pd_2(\mathbf{L2^H})_4](BF_4)\}^{3+}$ calc. 473.74; 754.10 $\{[Pd_2(\mathbf{L2^H})_4](BF_4)_2\}^{2+}$ calc. 754.11; 720.10 $\{[Pd_2(\mathbf{L2^H})_4](BF_4)F\}^{2+}$ calc. 720.10.

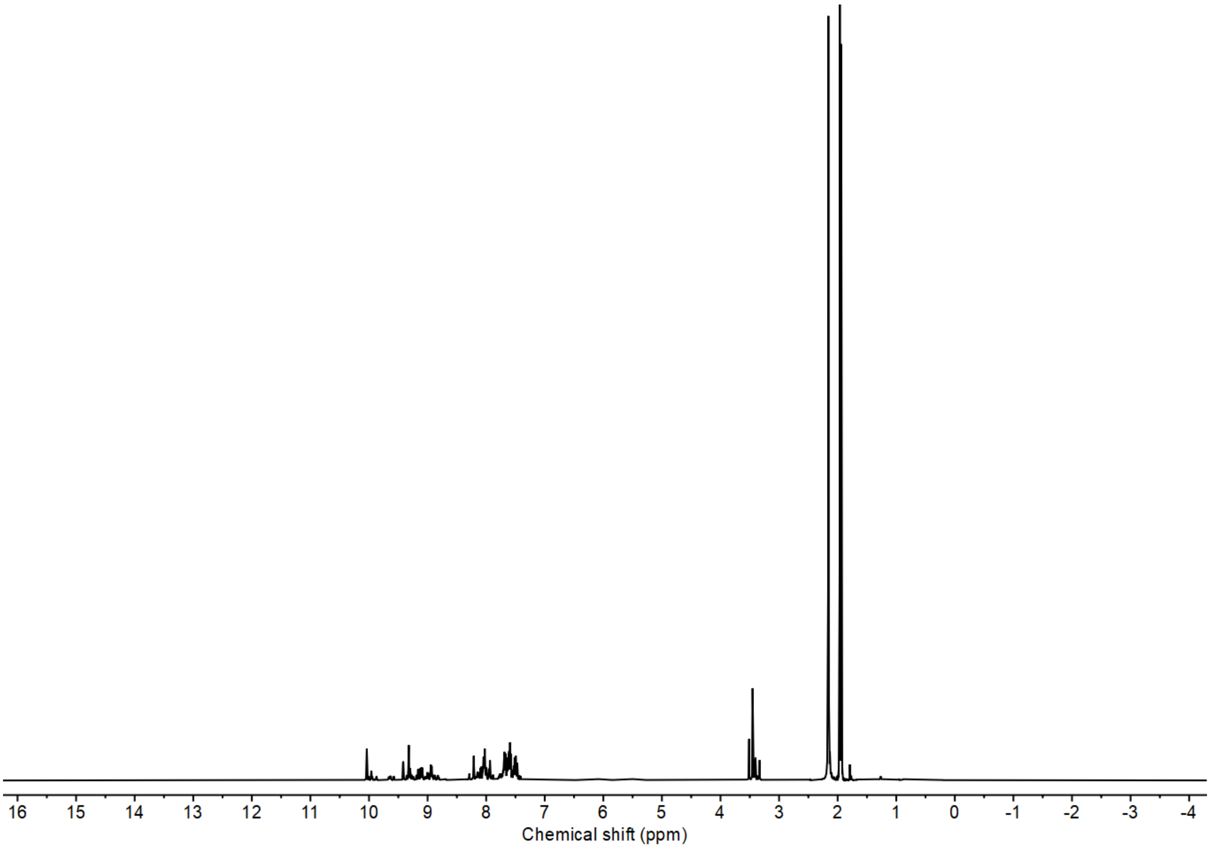


**Figure S231 $^1H$ NMR (400 MHz, $CD_3CN$) of $[Pd_2(L2^H)_2(L4^P)_2](BF_4)_4$.**

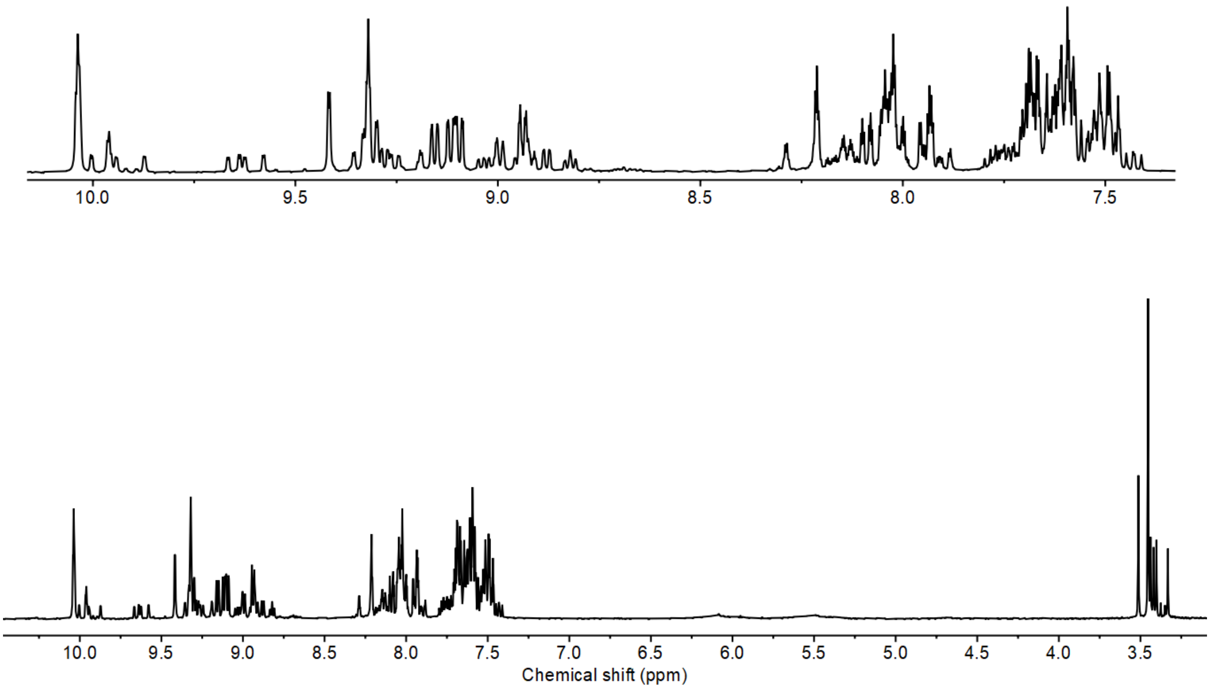


**Figure S232 Partial $^{1}H$ NMR (400 MHz, $CD_3CN$) of $[Pd_2(L2^H)_2(L4^P)_2](BF_4)_4$.**

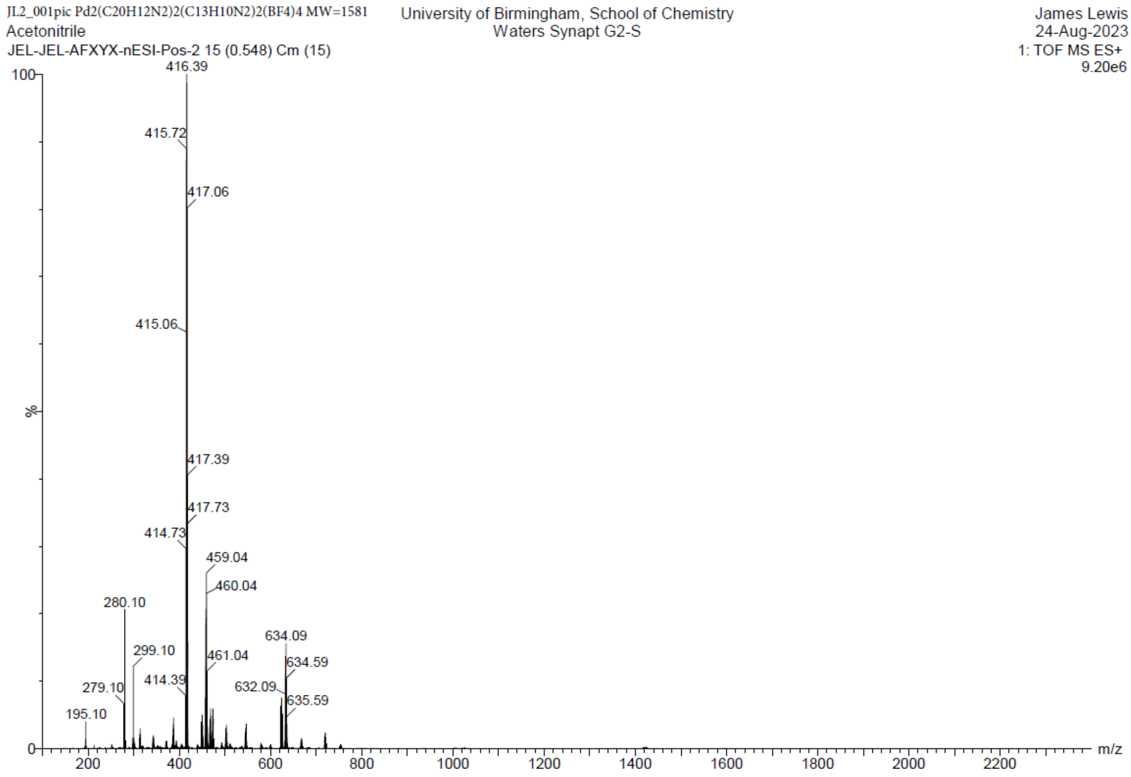


**Figure S233 ESI-MS of $[Pd_2(L2^H)_2(L4^P)_2](BF_4)_4$.**

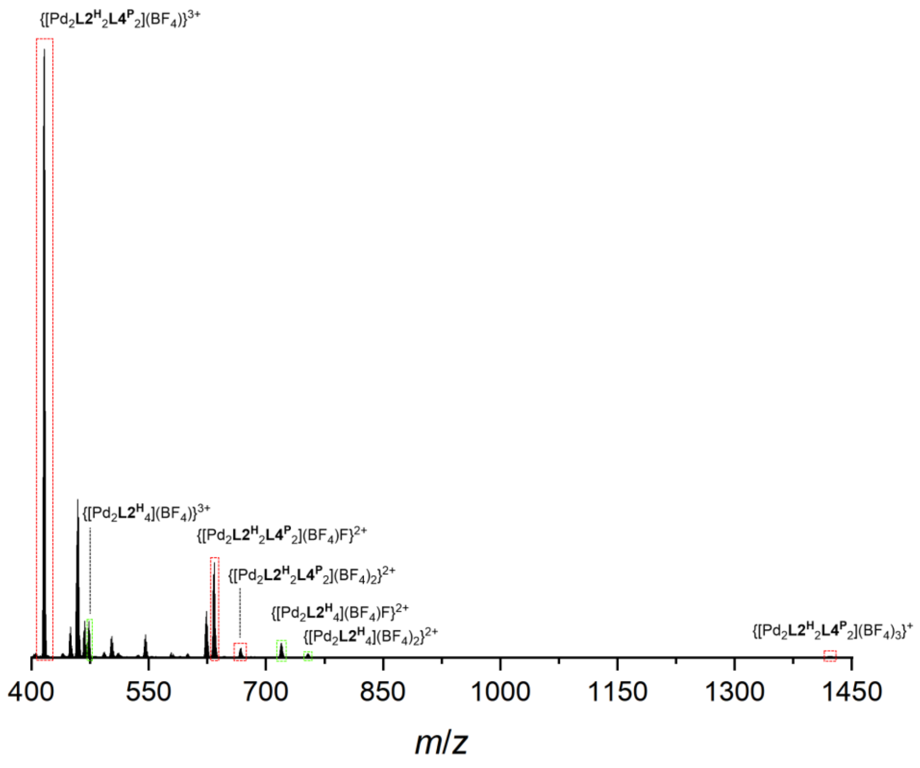


**Figure S234 ESI-MS of $[Pd_2(L2^H)_2(L4^P)_2](BF_4)_4$ with key peaks labelled.**

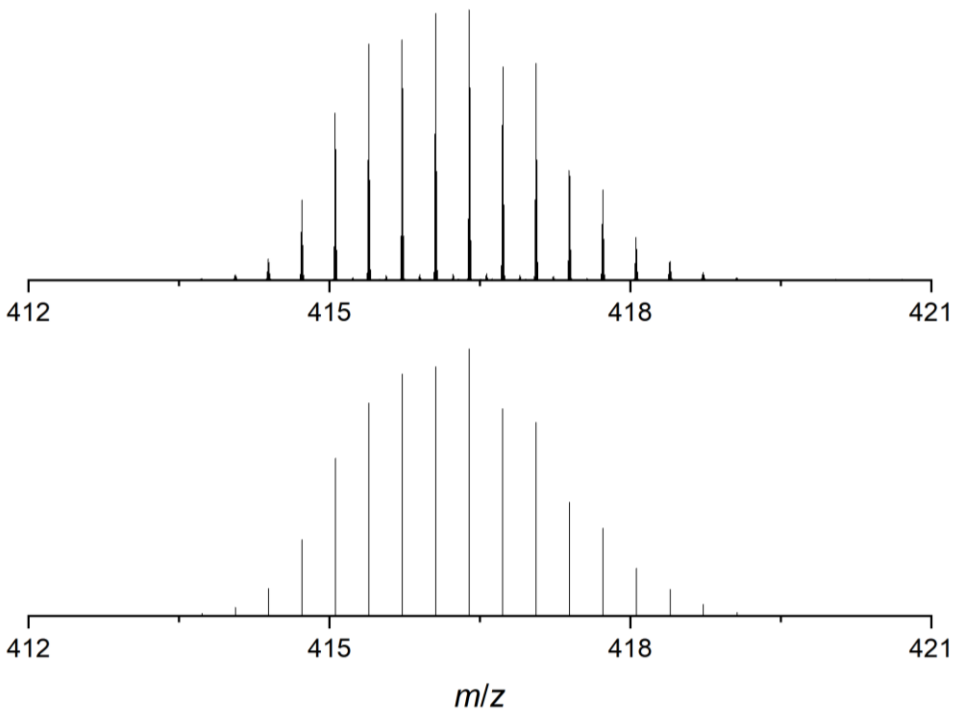


**Figure S235 Observed (top) and calculated (bottom) isotopic patterns for $\{[Pd_2(L2^H)_2(L4^P)_2](BF_4)\}^{3+}$.**

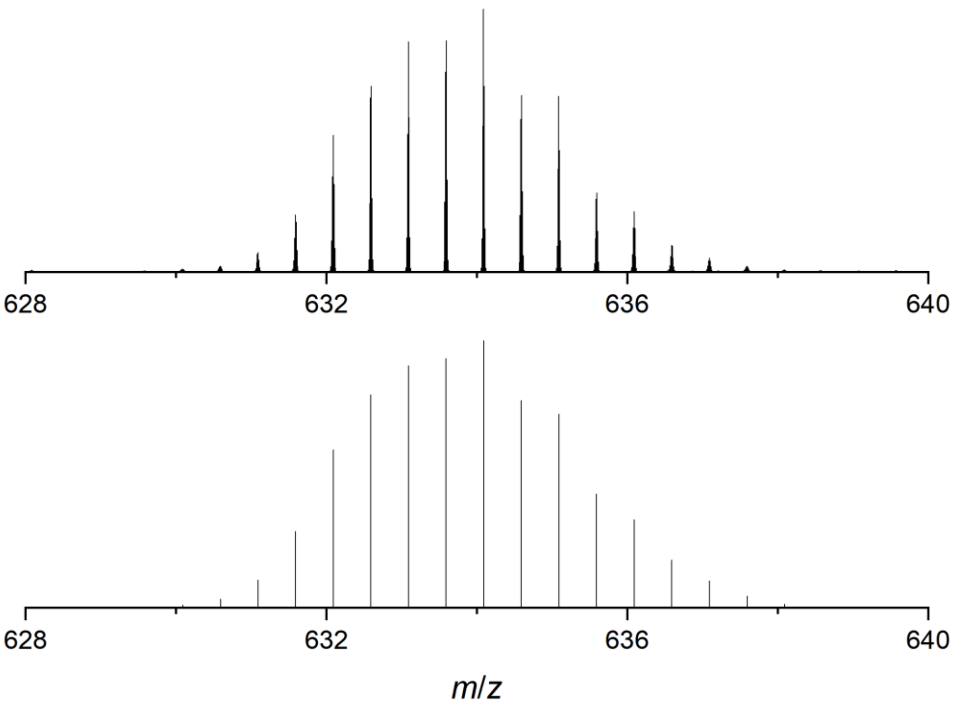


**Figure S236 Observed (top) and calculated (bottom) isotopic patterns for $\{[Pd_2(L2^H)_2(L4^P)_2](BF_4)F\}^{2+}$.**

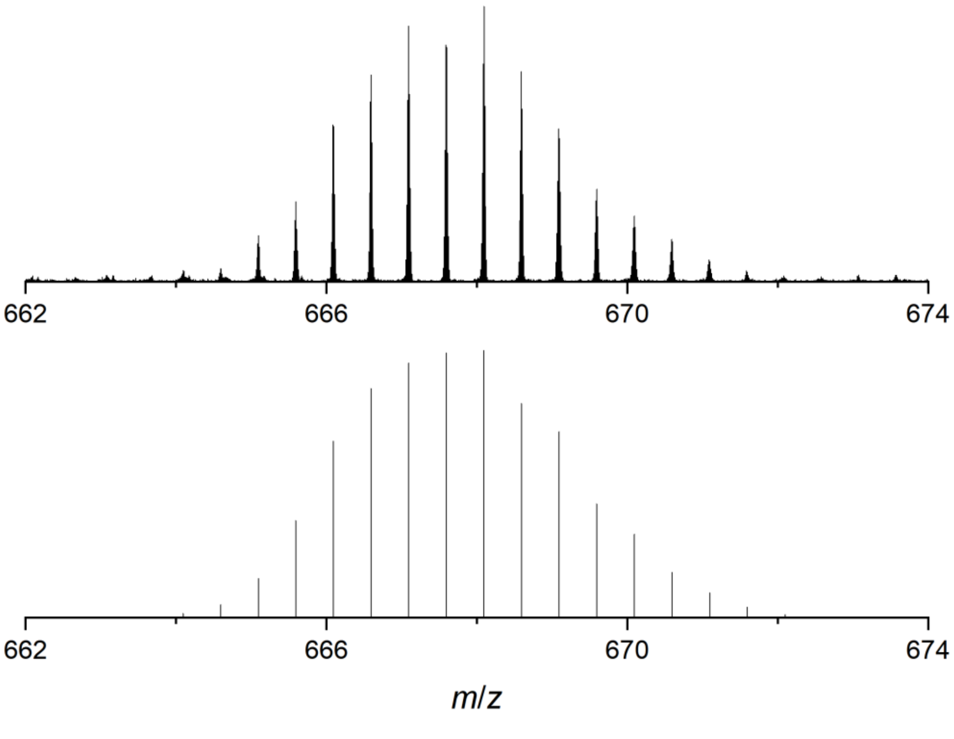


**Figure S237 Observed (top) and calculated (bottom) isotopic patterns for $\{[Pd_2(L2^H)_2(L4^P)_2](BF_4)_2\}^{2+}$.**

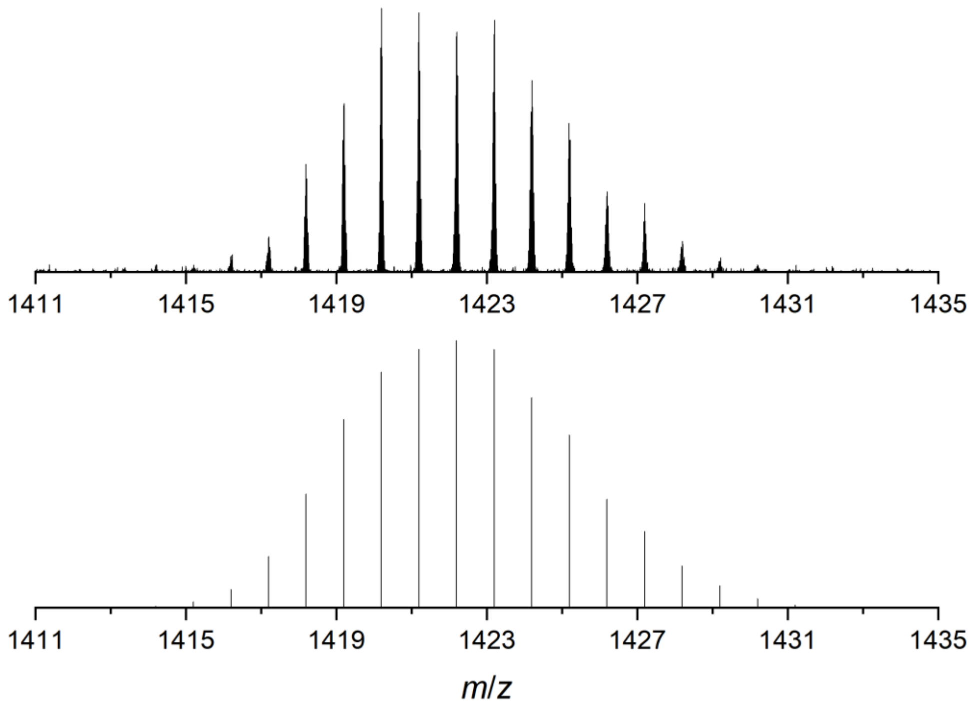


**Figure S238 Observed (top) and calculated (bottom) isotopic patterns for $\{[Pd_2(L2^H)_2(L4^P)_2](BF_4)_3\}^+$.**

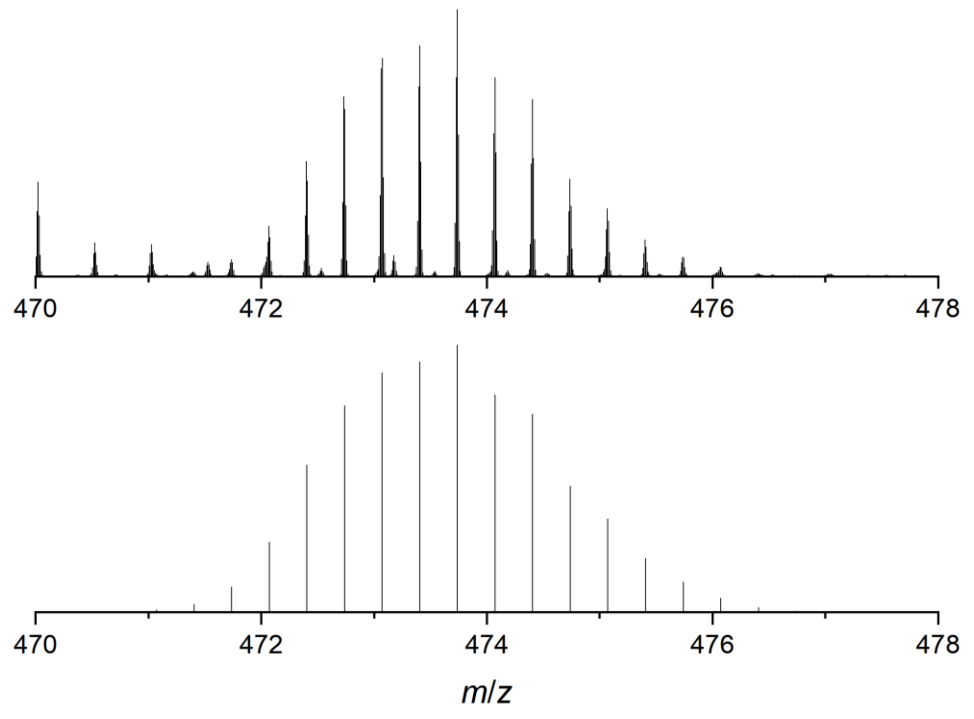


**Figure S239 Observed (top) and calculated (bottom) isotopic patterns for $\{[Pd_2(L2^H)_4](BF_4)\}^{3+}$.**

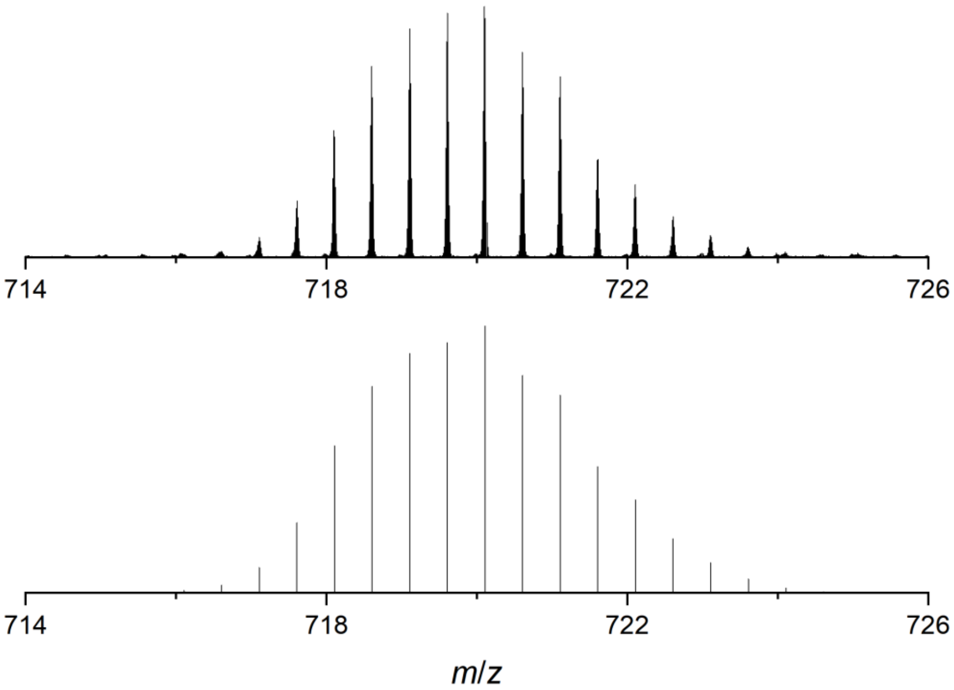


**Figure S240 Observed (top) and calculated (bottom) isotopic patterns for $\{[Pd_2(L2^H)_4](BF_4)F\}^{2+}$.**

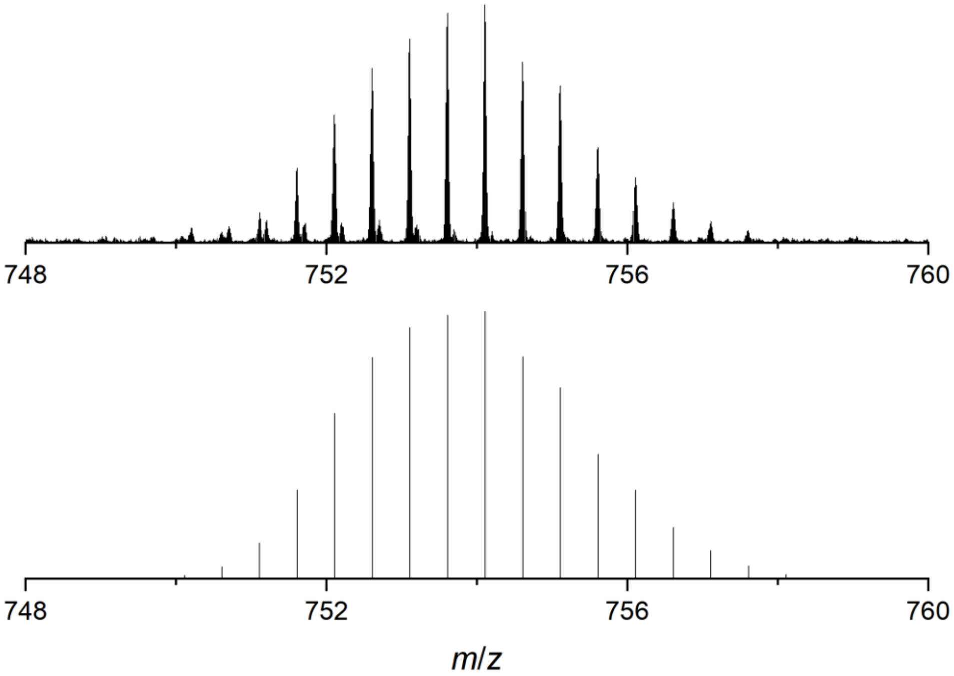


**Figure S241 Observed (top) and calculated (bottom) isotopic patterns for $\{[Pd_2(L2^H)_4](BF_4)_2\}^{2+}$.**

# S3. Density Functional Theory Calculations

## Building block construction

Models of the ligands **L1$^{Q}$**, **L1$^{P}$**, and **L3$^{P}$** were generated manually for use in cage model construction. The models were optimised with UFF,[2] such that their nitrogen donor groups were pointing in the same direction and their backbone was as linear/planar as possible.

## Cage model construction

*stk*[3] (https://github.com/lukasturcani/stk), the Universal Force Field (UFF in the General Utility Lattice Program (GULP)[4])[5] and the GFN2-xTB semiempirical method[6] were used to construct and optimise models of the various isomers of $Pd_2L_4$ and $Pd_3L_6$ cages. This approach is like that applied in our previous work on Pd-based cages.[7]

All code to reproduce this process is freely at https://github.com/andrewtarzia/citable_data/tree/master/molinska_2023.

The following optimisation sequence was used to generate the lowest energy conformer of each cage model (with the exception of the heteroleptic $[Pd_2($**L2$^{H}$**$)_2($**L4$^{Q}$**$)_2]^{4+}$ structures that were built manually):

1. The cage model was constructed with *stk* and MCHammer (https://github.com/andrewtarzia/MCHammer) optimiser used to produce an initial structure.

2. The cage structure was geometry optimised using UFF in GULP. This optimisation was performed in two steps: 1) with the conjugate gradient algorithm ("conj unit" option) and 2) with the second-order Newton-Raphson and BFGS hessian update (default options). The atom typing was handled by a Python implementation of the "ForceFieldHelpers" module in RDKit,[8] except for the metal atoms, which were manually typed to match the target types in UFF (because RDKit does not handle the metal-atom typing). Palladium atoms were assigned the square planar atom type, "Pd4+2". The bonding used within GULP matched the bonding in the *stk* molecule.

3. A conformer search was performed starting from the UFF-optimised cage structure using high-temperature molecular dynamics (MD) with UFF in GULP at 1000K, in the NVT ensemble, using the leapfrog verlet integrator. A short equilibration was run for 1.0 ps with a timestep of 0.25 fs and the production run performed for 200.5 ps with a time step of 0.75 fs. From the production run, conformers were extracted at regular intervals of 5 ps and optimised with UFF in GULP.

4. The lowest energy conformer was then geometry optimised using GFN2-xTB with the "extreme" (energy converged within $5 \times 10^{-8}$ Hartrees and gradient norm converged within $5 \times 10^{-5}$ Hartree bohr$^{-1}$) convergence criteria using the ALPB implicit solvent model of DMSO (with the "tight" grid) and a charge of +2 * the number of Pd atoms.

5. For cages **C1$^{Q}$** and **C1$^{P}$**, $Cl^-$ ions were placed at the optimised cage centroid with *stk* and geometry optimised using GFN2-xTB with the "extreme" convergence criteria using the ALPB implicit solvent model of DMSO and a charge of +3.

For the $Pd_3L_6$ models formed from **L3$^{P}$**, only the all-*cis* and all-*trans* isomers were built (out of 9 potential isomers). The same procedure was applied as above but stopping after step 4 and

applying a manual modification of the cage conformation to obtain a more symmetrical structure. Steps 2 and 4 were then performed on the modified structure (i.e., without the high temperature MD conformer search). No ions were added to these models.

## DFT methodology

DFT calculations were carried out in Gaussian16[9], with the optimisation first carried out with the hybrid functional PBE0D3BJ[10], followed by optimisation with the screened-hybrid functional HSE06[11] and implicit DMSO solvent. The calculations were all-electron with no pseudopotentials used and the Ahlrichs Def2-SVP basis sets[12], which has polarisation on all atoms. Each palladium had a charge of +2 and was assumed to be in the low-spin state. The calculations were carried out with an accurate pruned grid ('superfinegrid' option in Gaussian16). We have successfully used this approach previously for Pd-cages[13] and it has been demonstrated to be appropriate for third-row transition metals.[14]

## Geometry-optimised structures

GFN2-xTB and DFT optimised structures are freely available here: https://github.com/andrewtarzia/citable_data/tree/master/molinska_2023.

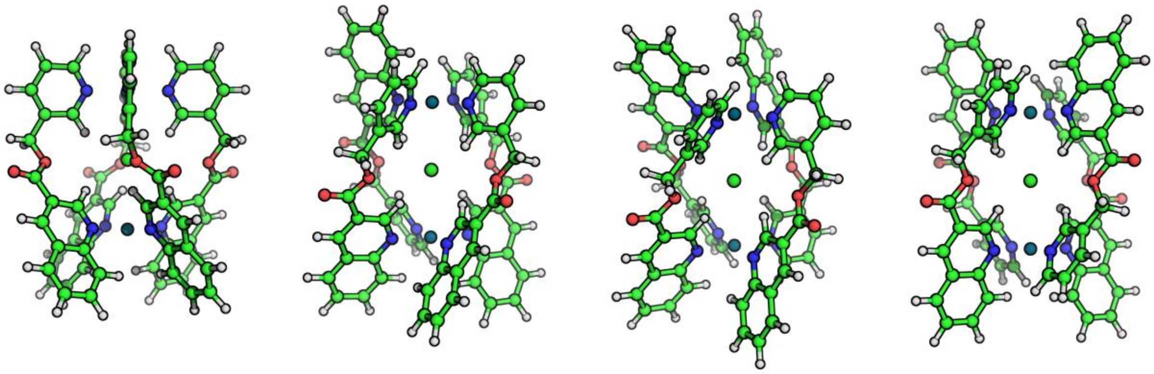

**Figure S242 DFT-optimised (HSE) structures of (from left to right) 'all-up', 'three-up-one-down', *cis* and *trans* isomers of $[Pd_2(L1^Q)_4 \supset Cl]^{3+}$.**

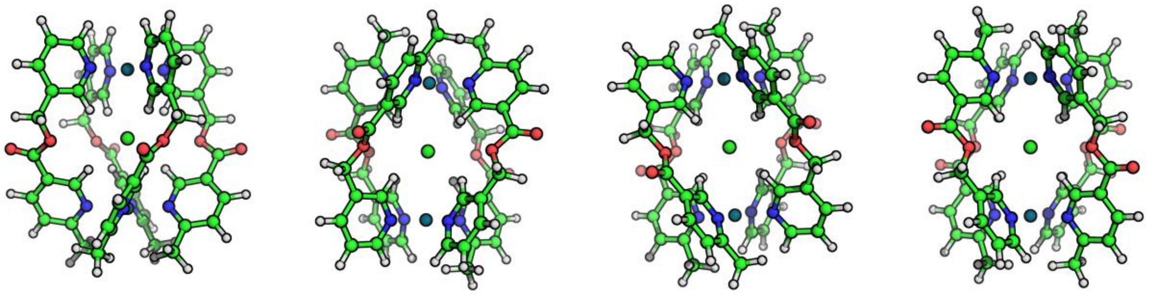

**Figure S243 DFT-optimised (HSE) structures of (from left to right) 'all-up', 'three-up-one-down', *cis* and *trans* isomers of $[Pd_2(L1^P)_4 \supset Cl]^{3+}$.**

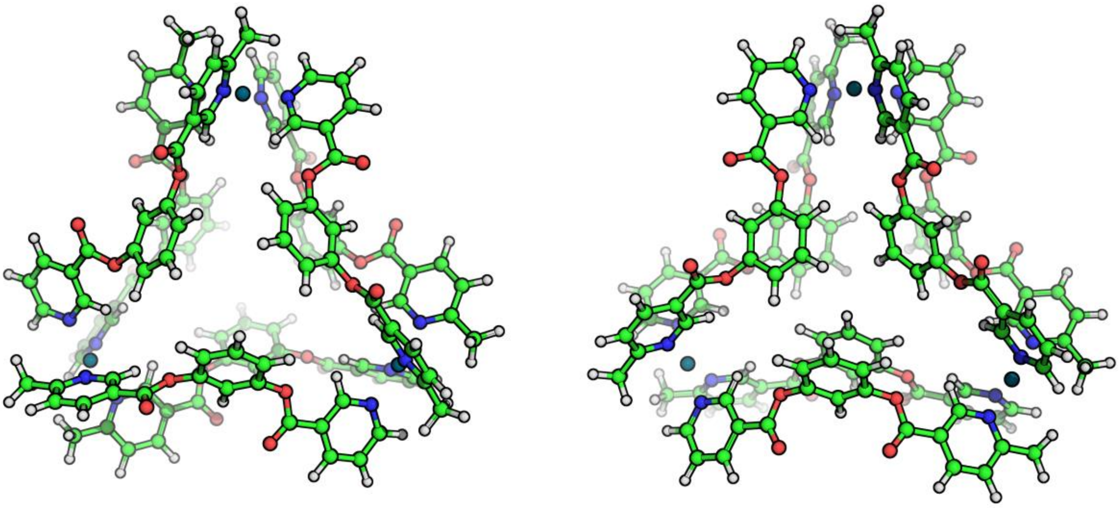

**Figure S244 DFT-optimised (HSE) structures of *cis*- (left) and *trans*-$[Pd_3(L3^P)_6]^{6+}$ (right).**

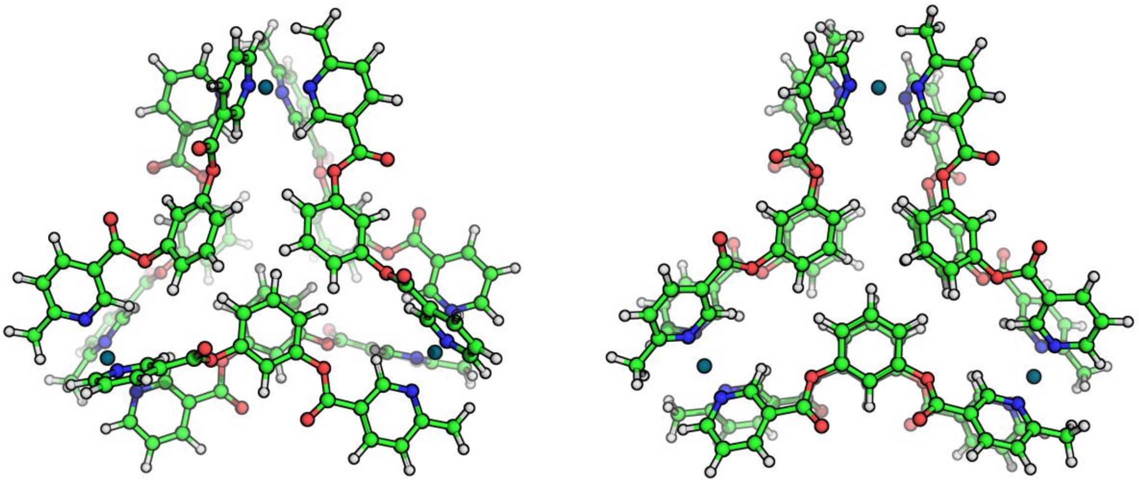

**Figure S245 DFT-optimised (PBE0) structures of *cis*- (left) and *trans*-$[Pd_3(L3^P)_6]^{6+}$ (right).**

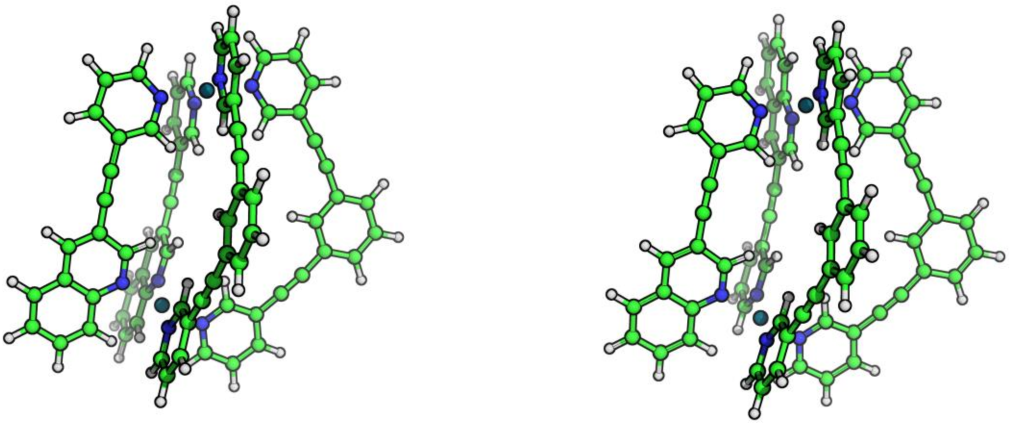

**Figure S246 xTB-optimised structures of *syn* (left) and *anti* (right) isomers of $[Pd_2(L2^H)_2(L4^Q)_2]^{4+}$.**

# Isomer energy comparisons

**Table S1 Calculated relative energies of optimised geometries for isomers of C1$^{Q}$, C1$^{P}$, C3$^{P}$ and [Pd$_2$(L2$^{H}$)$_2$(L4$^{Q}$)$_2$]$^{4+}$. $^{a}$ without implicit DMSO solvation.**

| Cage | Isomer | *E* (xTB, Ha) | Δ*E* (xTB, kJ mol$^{-1}$) | *E* (HSE, Ha) | Δ*E* (HSE, kJ mol$^{-1}$) |
|---|---|---|---|---|---|
| **C1$^{Q}$** | 'All-up' | -230.5388365 | 10.0 | -4216.522361 | 9.3 |
| | '3-u-1-d' | -230.539971 | 7.0 | -4216.522946 | 7.7 |
| | *cis* | -230.5380815 | 12.0 | -4216.521864 | 10.6 |
| | *trans* | -230.5426374 | 0 | -4216.525891 | 0 |
| **C1$^{P}$** | 'All-up' | -204.827224 | 16.8 | -3760.002892 | 10.3 |
| | '3-u-1-d' | -204.8317434 | 4.9 | -3760.00438 | 6.4 |
| | *cis* | -204.8306348 | 7.8 | -3760.003947 | 7.5 |
| | *trans* | -204.8336077 | 0 | -3760.006802 | 0 |
| **C3$^{P}$** | *cis* | -431.880677573905 | 16.2 | -7228.192788 | 0 |
| | *trans* | -431.886834516297 | 0 | -7228.18290577 | 25.9 |
| | | **_E_ (PBE0, Ha)$^{a}$** | **Δ*E* (PBE0, kJ mol$^{-1}$)** | **_E_ (HSE, Ha)$^{a}$** | **Δ*E* (HSE, kJ mol$^{-1}$)** |
| | *cis* | -7226.9011915 | 22.9 | -7227.08509742 | 0 |
| | *trans* | -7226.90991977 | 0 | -7227.0806932 | 11.8 |
| | | **_E_ (xTB, Ha)** | **Δ*E* (xTB, kJ mol$^{-1}$)** | | |
| [Pd$_2$(**L2$^{H}$**)$_2$(**L4$^{Q}$**)$_2$]$^{4+}$ | *syn* | -207.702701910685 | 10.5 | | |
| | *anti* | -207.706685505447 | 0 | | |

# S4. Solvodynamic Radii Calculations

Solvodynamic radii were calculated using a variation of the Stokes-Einstein equation:

$$R_s = \frac{k_B T}{6\pi\eta D}$$

Where $R_s$ is the solvodynamic radius (m)

$k_B$ is the Boltzmann constant ($1.38 \times 10^{-23}$ J $K^{-1}$)

$T$ is the temperature (K)

$\eta$ is the solvent viscosity

$2.180 \times 10^{-3}$ kg $s^{-1}m^{-1}$ for $d_6$-DMSO[15]

$3.57 \times 10^{-4}$ kg $s^{-1}m^{-1}$ for $CD_3CN$[15]

$D$ is the diffusion coefficient ($m^2s^{-1}$)

## S5. X-ray Crystallography

The datasets for ***cis*-C1$^Q$⊃Cl** and ***cis*-C2$^Q$** were measured on a Rigaku XtaLAB Synergy diffractometer using a HyPix detector. The dataset for ***trans*-C1$^P$⊃Cl** was measured on an Agilent SuperNova diffractometer using an Atlas detector. These data collections were driven and processed and absorption corrections were applied using CrysAlisPro.[16] The datasets for ***cis*-C3$^Q$** and [$Pd_2$(**L2$^H$**)$_2$(**L4$^Q$**)$_2$]($BF_4$)$_4$ were measured at the Diamond Light Source, Beamline I19-1, using a Dectris PILATUS 2M detector.[17] These data set were processed and absorption corrections were applied using DIALS 3, XIA2 and AIMLESS.[18]

Using OLEX2,[19] the structures were solved using ShelXT,[20] and were refined by a full-matrix least-squares procedure on $F^2$ in ShelXL.[21] Unless otherwise specified, all non-hydrogen atoms were refined with anisotropic displacement parameters. In all structures the hydrogen atoms were fixed as riding models and the isotropic thermal parameters ($U_{iso}$) were based on the $U_{eq}$ of the parent atom.

### S5.1 *cis*-[$Pd_2$(L1$^Q$)$_4$⊃Cl]($BF_4$)$_3$

**L1$^Q$** (22.8 mg, 0.1 mmol, 4.0 eq.), [Pd($CH_3CN$)$_4$]($BF_4$)$_2$ (22.3 mg, 0.05 mmol, 2.0 eq.) and $Bu_4NCl$ (6.9 mg, 0.025 mmol, 1.0 eq.) were sonicated in $d_6$-DMSO (2 mL) until a homogenous solution was obtained. After heating at 50 °C for 24 h the solution was diluted with DMF (2 mL), filtered through celite and left for vapour diffusion of $Et_2O$.

$C_{77}H_{83}B_3ClF_{12}N_9O_{12}Pd_2S$ (*M* =1867.26 g/mol): triclinic, space group P-1 (no. 2), *a* = 15.18660(10) Å, *b* = 16.20810(10) Å, *c* = 16.57430(10) Å, *α* = 92.9370(10)°, *β* = 103.7560(10)°, *γ* = 92.1780(10)°, *V* = 3952.17(5) Å$^3$, *Z* = 2, *T* = 100.00(10) K, μ(Cu Kα) = 5.055 mm$^{-1}$, *Dcalc* = 1.569 g/cm$^3$, 75015 reflections measured (5.5° ≤ 2Θ ≤ 158.15°), 16145 unique ($R_{int}$ = 0.0350, $R_{sigma}$ = 0.0283) which were used in all calculations. The final $R_1$ was 0.0789 (I > 2σ(I)) and $wR_2$ was 0.2269 (all data). CCDC 2300292.

The structure contains one palladium dimer and one chloride anion in total, arranged as two half dimers located on inversion centres at the site of the chloride anions.

The structure also contains and three $BF_4$ anions, two of which, B(301), F(301)-F(304) / B(31A), F(31A)-F(34A), and B(401), F(401)-F(404) / B(41A), F(41A)-F(44A) being disordered over two positions each at refined percentage occupancy ratios of 58.6 (14) : 41.4 (14) and 50 (2) : 50 (2) respectively. There are also two molecules of diethyl ether, one molecule of dimethyl sulfoxide, and one of dimethyl formamide.

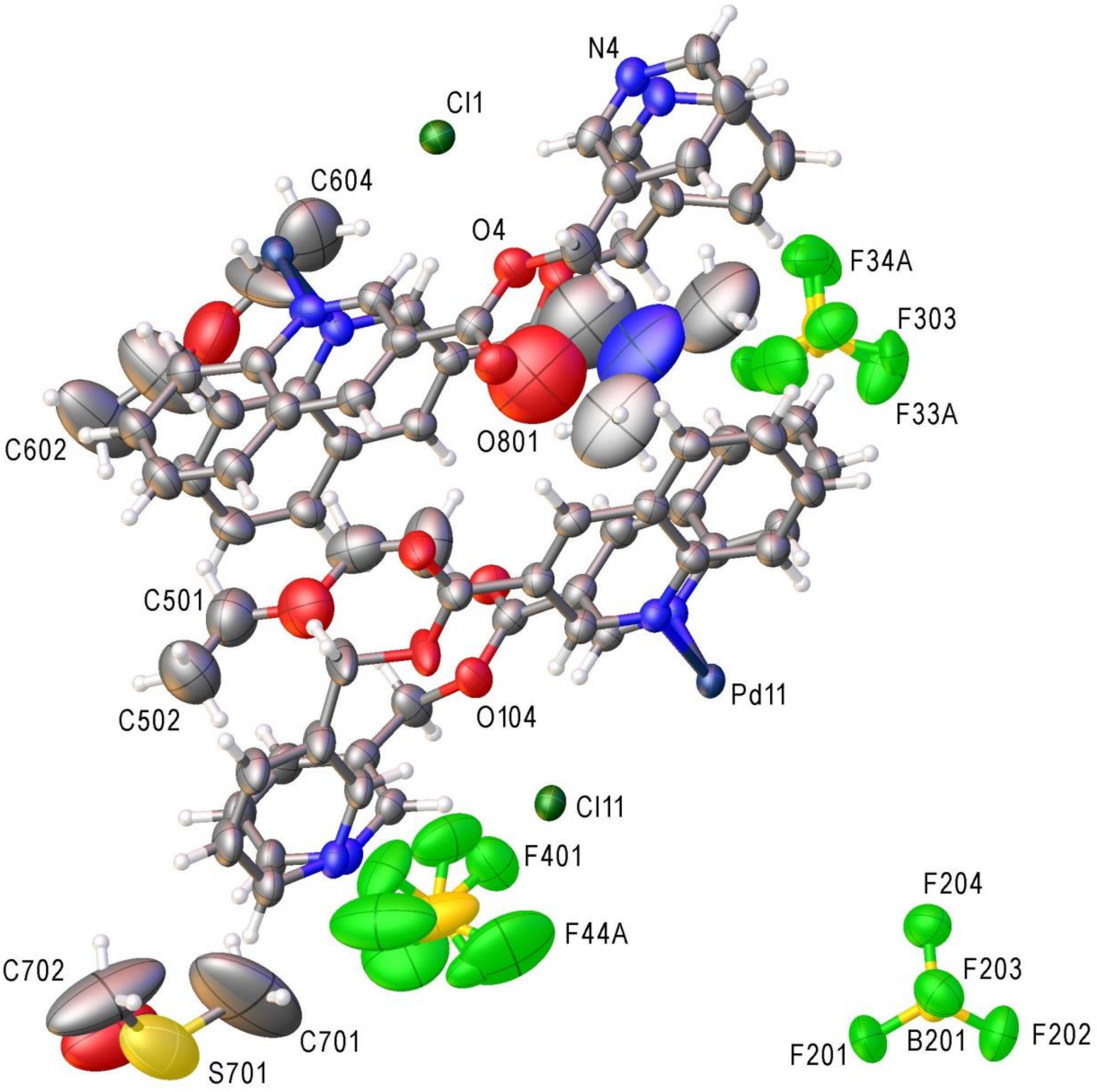


**Figure S247 Crystal structure of *cis*-[Pd$_2$(L1$^Q$)⊃Cl](BF$_4$)$_3$ with ellipsoids drawn at the 50 % probability level. The structure contains one palladium dimer and one chloride anion in total, arranged as two half dimers located on inversion centres at the site of the chloride anions. The structure also contains and three BF$_4$ anions, two of which, B(301), F(301)-F(304) / B(31A), F(31A)-F(34A), and B(401), F(401)-F(404) / B(41A), F(41A)-F(44A) being disordered over two positions each. There are two molecules of diethyl ether, one molecule of dimethyl sulfoxide, and one of dimethyl formamide.**

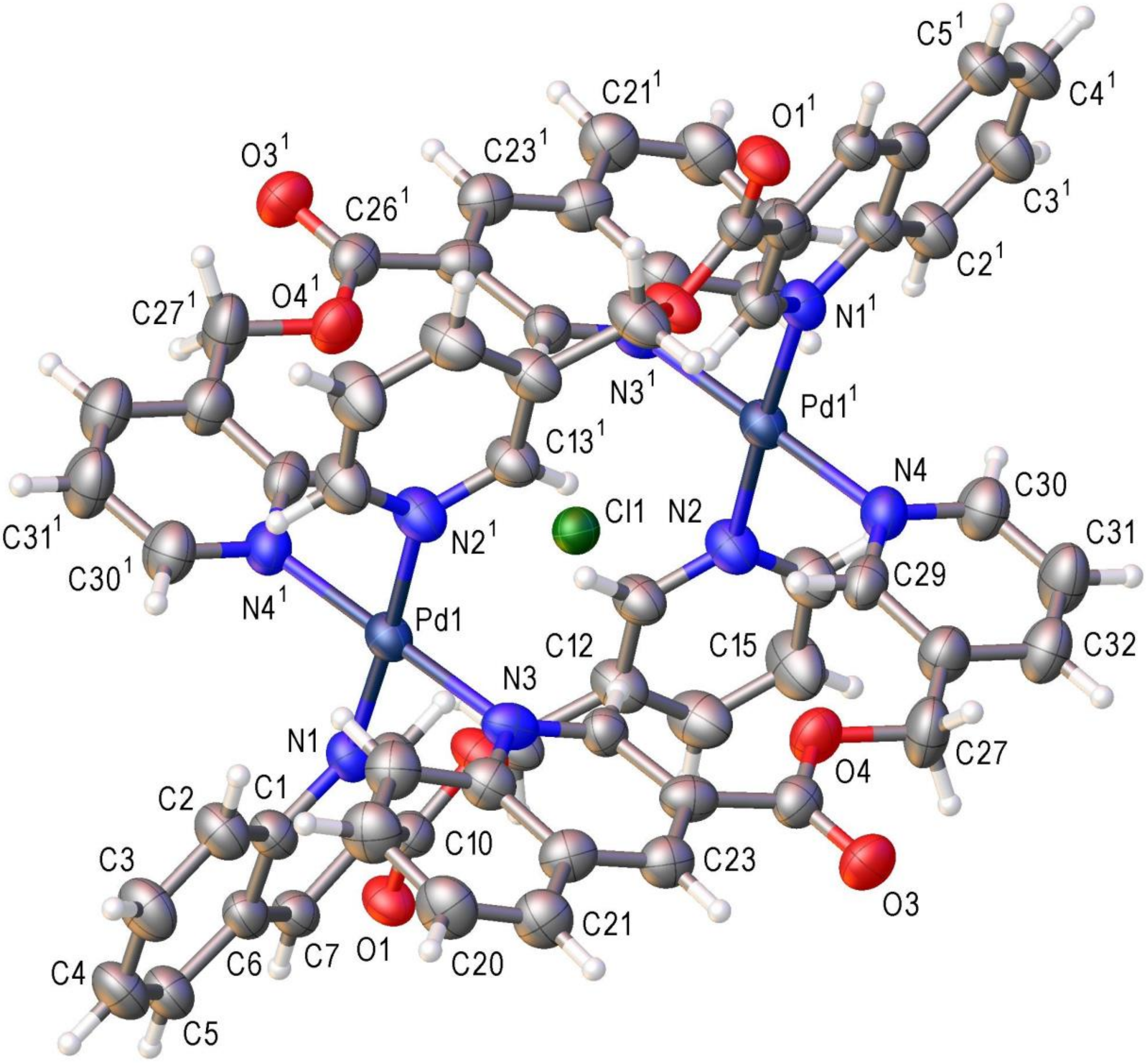


**Figure S248 Crystal structure of *cis*-[$Pd_2(L1^{Q})\supset Cl$]($(BF_4)_3$) with ellipsoids drawn at the 50 % probability level. The structure contains one palladium dimer and one chloride anion in total, arranged as two half dimers located on inversion centres at the site of the chloride anions. The structure also contains and three $BF_4$ anions, two molecules of diethyl ether, one molecule of dimethyl sulfoxide, and one of dimethyl formamide. Only one of the palladium dimers and chloride anions is shown for clarity. Symmetry code used to generate equivalent atoms: $1 1-x, 1-y, -z.**

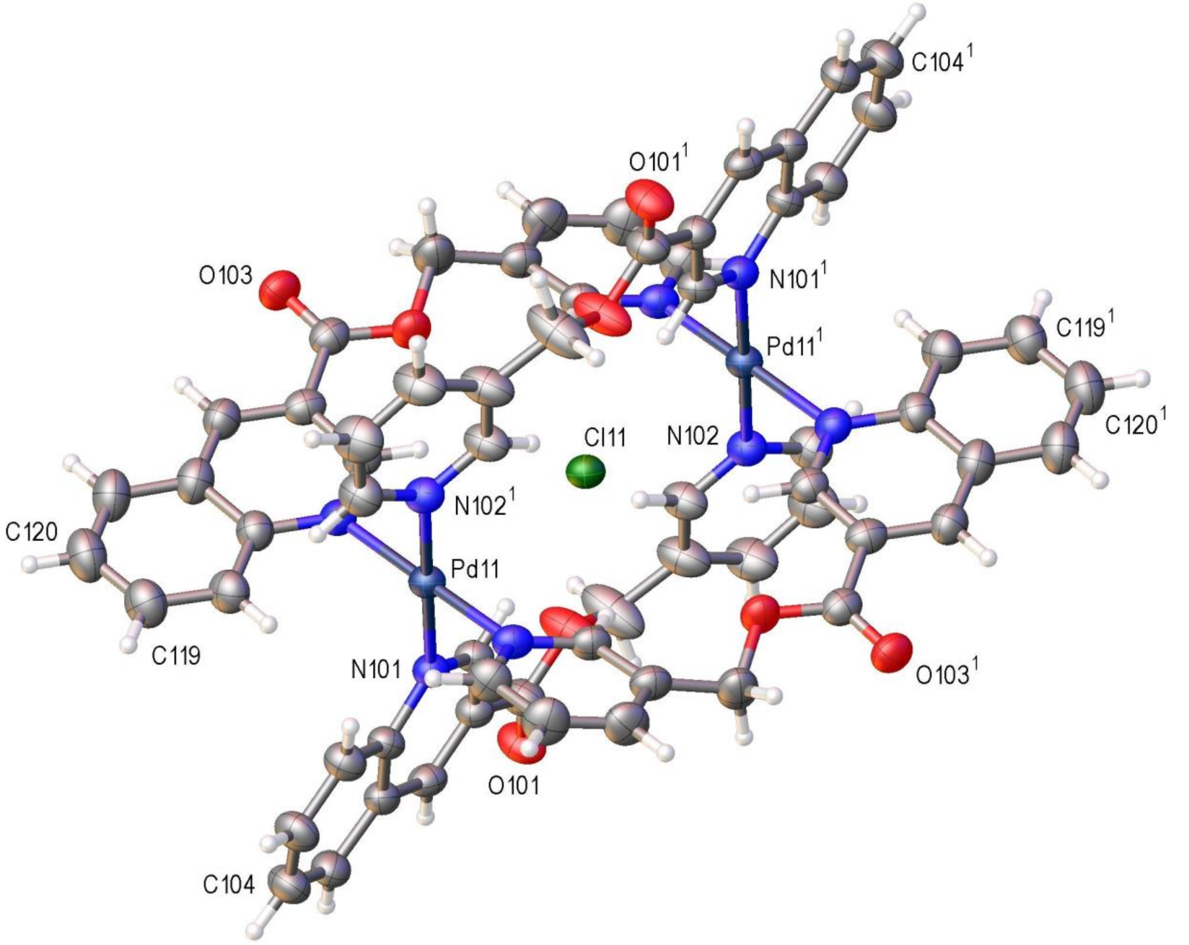


**Figure S249 Crystal structure of *cis*-[$Pd_2(L1^{Q})$⊃Cl]$(BF_4)_3$ with ellipsoids drawn at the 50 % probability level. The structure contains one palladium dimer and one chloride anion in total, arranged as two half dimers located on inversion centres at the site of the chloride anions. The structure also contains and three $BF_4$ anions, two molecules of diethyl ether, one molecule of dimethyl sulfoxide, and one of dimethyl formamide. Only one of the palladium dimers and chloride anions is shown for clarity. Symmetry code used to generate equivalent atoms: $1 1-x, -y, 1-z.**

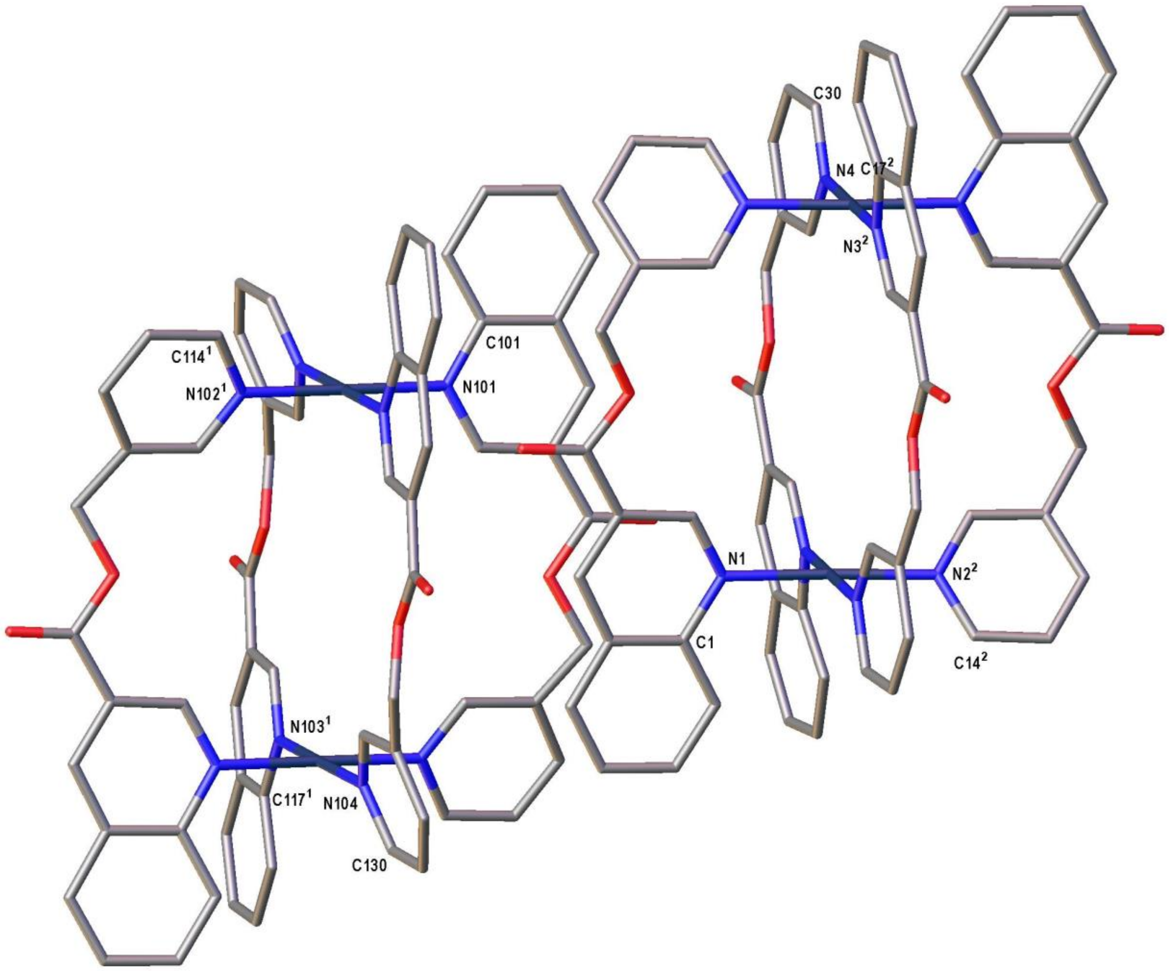


**Figure S250 SCXRD structure of the two crystallographically independent molecules of C1$^{Q}$ with labelled atoms for torsion angle measurements. Symmetry codes used to generate equivalent atoms: $1 1-x, -y, 1-z, $2 1-x, 1-y, -z.**

**Table S2 Torsion angles between pyridine and quinoline donors in SCXRD structure of C1$^{Q}$⊃Cl.**

| | | | | Torsion angle Θ (°) |
|---|---|---|---|---|
| C130 | N104 | N103 | C117 | -16.8(6) |
| C114 | N102 | N101 | C101 | -19.8(6) |
| C30 | N4 | N3 | C17 | 3.1(6) |
| C14 | N2 | N1 | C1 | 27.9(6) |

## S5.2 *trans*-[$Pd_2(L1^P)_4 \supset Cl$]($BF_4$)$_3$

**L1$^P$** (22.8 mg, 0.1 mmol, 4.0 eq.), [$Pd(CH_3CN)_4$]($BF_4$)$_2$ (22.3 mg, 0.05 mmol, 2.0 eq.) and $Bu_4NCl$ (6.9 mg, 0.025 mmol, 1.0 eq.) were sonicated in $d_6$-DMSO (2 mL) until a homogenous solution was obtained. After heating at 50 °C for 24 h the solution was diluted with DMF (2 mL), filtered through celite and left for vapour diffusion of $Et_2O$.

$C_{64}H_{82}B_3ClF_{12}N_8O_{13}Pd_2S_4$ (*M* =1808.29 g/mol): triclinic, space group P-1 (no. 2), *a* = 13.0250(3) Å, *b* = 16.3013(4) Å, *c* = 19.7245(4) Å, *α* = 102.713(2)°, *β* = 90.449(2)°, *γ* = 99.435(2)°, *V* = 4025.86(16) Å$^3$, *Z* = 2, *T* = 120.00(10) K, μ(Cu Kα) = 5.652 mm$^{-1}$, *Dcalc* = 1.492 g/cm$^3$, 40349 reflections measured (8.04° ≤ 2Θ ≤ 145.214°), 15527 unique ($R_{int}$ = 0.0446, $R_{sigma}$ = 0.0486) which were used in all calculations. The final $R_1$ was 0.0557 (I > 2σ(I)) and $wR_2$ was 0.1539 (all data). CCDC 2300288.

The structure contains one chloride and three $BF_4$ anions, one of the latter, B(301), F(301)-F(304) / B(31A), F(31A)-F(34A), being disordered over two positions at a refined percentage occupancy ratio of 67.7 (14) : 32.3 (14).

There are four molecules of dimethyl sulfoxide, one of which (O(701), S(701), C(701), C(702) / O(71A), S(71A), C(71A), C(72A)) is disordered over two positions at a refined occupancy ratio of 70.0 (6) : 30.0 (6).

There is also one molecule of diethyl ether (O801), C(801)-C(804) / O(81A), C(81A)-C(84A)) disordered over two positions at a refined occupancy ratio of 63.6 (6) : 36.4 (6), the minor position being located on an inversion centre.

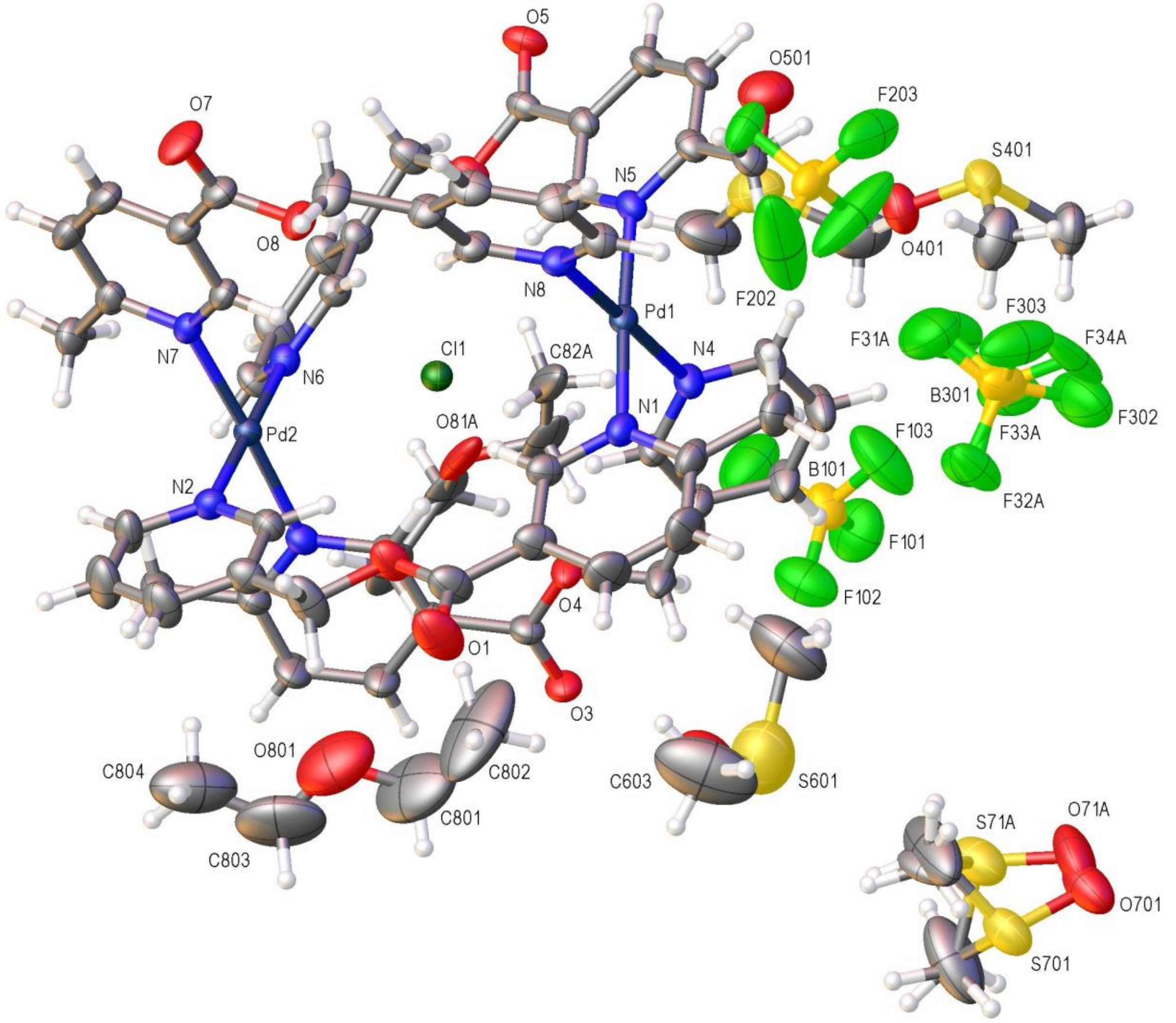


**Figure S251 Crystal structure of *trans*-[Pd$_2$(L1$^P$)⊃Cl](BF$_4$)$_3$ with ellipsoids drawn at the 50% probability level. The structure contains one chloride and three BF$_4$ anions, one of the latter, B(301), F(301)-F(304) / B(31A), F(31A)-F(34A), being disordered over two positions. There are four molecules of dimethyl sulfoxide, one of which (O(701), S(701), C(701), C(702) / O(71A), S(71A), C(71A), C(72A)) being disordered over two positions. There is also one molecule of diethyl ether (O801), C(801)-C(804) / O(81A), C(81A)-C(84A)) disordered over two positions, the minor position being located on an inversion centre.**

## S5.3 *cis*-$[Pd_2(L2^Q)_4\supset DMSO_2](BF_4)_2X_2$

**$L2^Q$** (9.9 mg, 30μ mmol, 1.0 eq.) and $[Pd(CH_3CN)_4](BF_4)_2$ (7.3 mg, 16.5 μmol, 0.55 eq.) were sonicated in $d_6$-DMSO (0.75 mL) until a homogenous solution was obtained. After heating at 50 °C for 19 h the solution was diluted with DMF (1.5 mL) and $Bu_4NOTf$ (11.7 mg, 30 μmol, 1.0 eq.) added. After filtering through celite the solution was left for vapour diffusion of $Et_2O$, yielding orange crystals.

$C_{50}H_{34}BF_4N_4OPdS$ ($M$ =932.08 g/mol): triclinic, space group P-1 (no. 2), $a$ = 12.39640(10) Å, $b$ = 13.65590(10) Å, $c$ = 18.7481(2) Å, $\alpha$ = 102.0890(10)°, $\beta$ = 94.7500(10)°, $\gamma$ = 99.1760(10)°, $V$ = 3041.38(5) $Å^3$, $Z$ = 2, $T$ = 99.98(10) K, μ(Cu Kα) = 3.138 $mm^{-1}$, $Dcalc$ = 1.018 $g/cm^3$, 45680 reflections measured (6.732° ≤ 2Θ ≤ 157.732°), 12454 unique ($R_{int}$ = 0.0419, $R_{sigma}$ = 0.0340) which were used in all calculations. The final $R_1$ was 0.0623 (I > 2σ(I)) and $wR_2$ was 0.2088 (all data). CCDC 2300289.

The structure contains one palladium dimer, located on an inversion centre, such that only half is crystallographically unique.

One $BF_4$ anion and a molecule of DMSO have also been refined. Remaining anions and solvent molecules could not be refined, and a solvent mask has been used.

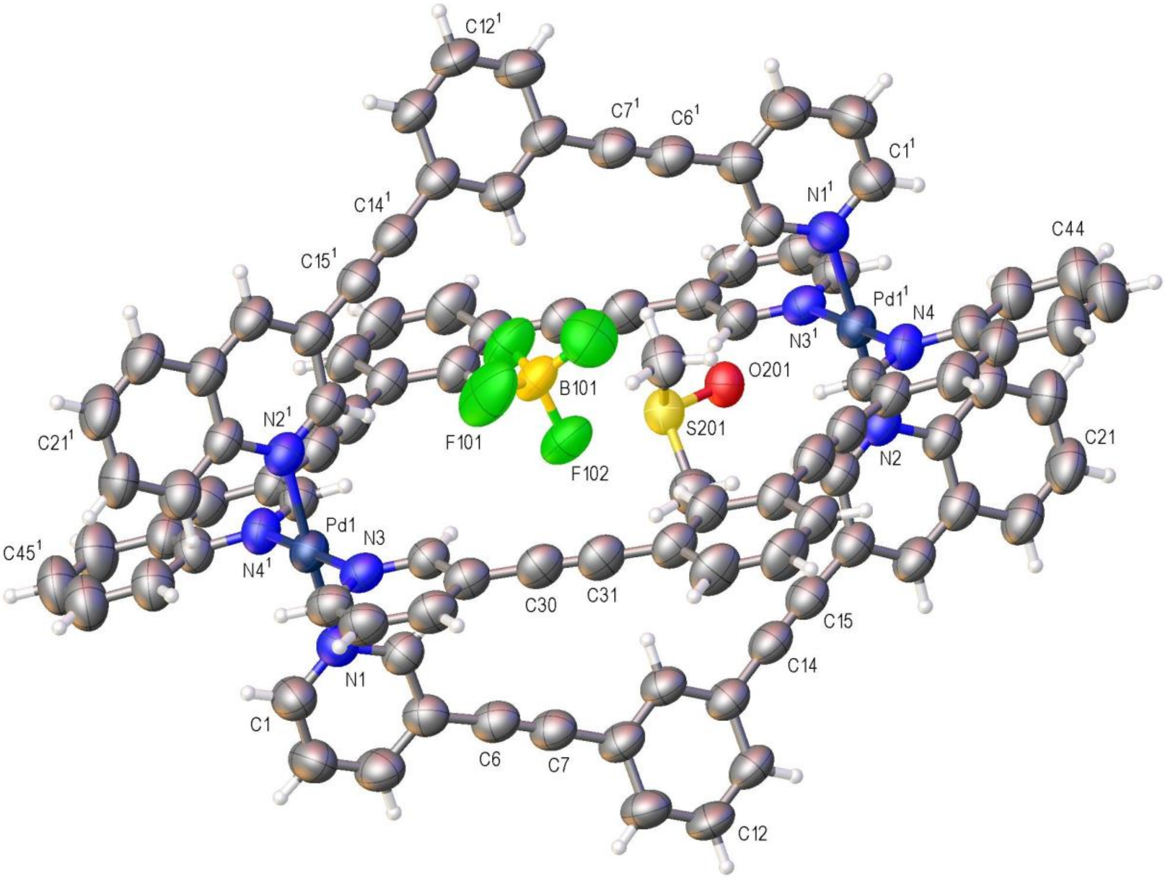


**Figure S252 Crystal structure of *cis*-$[Pd_2(L2^Q)_4\supset DMSO_2](BF_4)_2X_2$ with ellipsoids drawn at the 50 % probability level. The structure contains one palladium dimer, located on an inversion centre, such that only half is crystallographically unique. One $BF_4$ anion and a molecule of DMSO have also been refined. Remaining anions and solvent molecules could not be refined and a solvent mask has been used. Symmetry code used to generate equivalent atoms: $1 1-x, 1-y, 1-z.**

## S5.4 *cis*-[$Pd_3(L3^Q)_6$⊃$DMF_2$DMSO]$(BF_4)_6$

**$L3^Q$** (11.1 mg, 30 µmol, 1 eq.) and [Pd($CH_3CN)_4$]($BF_4)_2$ (6.7 mg, 15 µmol, 0.5 eq.) were sonicated in $d_6$-DMSO (0.75 mL) until a homogenous solution was obtained, and then stood at 50 °C for 2 d. The solution was diluted with DMF (1.0 mL), filtered through celite and left for vapour diffusion of $Et_2O$ from which X-ray quality crystals grew.

$C_{140}H_{104}B_3F_{12}N_{14}O_{27}Pd_3S$ (*M* =3026.06 g/mol): triclinic, space group P-1 (no. 2), *a* = 21.0264(5) Å, *b* = 23.5273(5) Å, *c* = 25.9306(4) Å, *α* = 72.642(2)°, *β* = 70.038(2)°, *γ* = 85.663(2)°, *V* = 11503.0(4) Å$^3$, *Z* = 2, *T* = 100.15 K, μ(?) = 0.275 mm$^{-1}$, *Dcalc* = 0.874 g/cm$^3$, 147390 reflections measured (1.692° ≤ 2Θ ≤ 48.416°), 40361 unique ($R_{int}$ = 0.0559, $R_{sigma}$ = 0.0760) which were used in all calculations. The final $R_1$ was 0.0892 (I > 2σ(I)) and $wR_2$ was 0.2767 (all data). CCDC 2300292.

The data were collected at the Diamond Light Source but the diffraction was very weak. The refinement is the best that could be achieved with the data available.

The structure contains six $BF_4$ anions per molecular triangle, of which only three have been located in the electron density. Two molecules of dimethylformamide and one molecule of dimethyl sulfoxide have been refined. Remaining anions and solvent molecules could not be refined, and a solvent mask has been used.

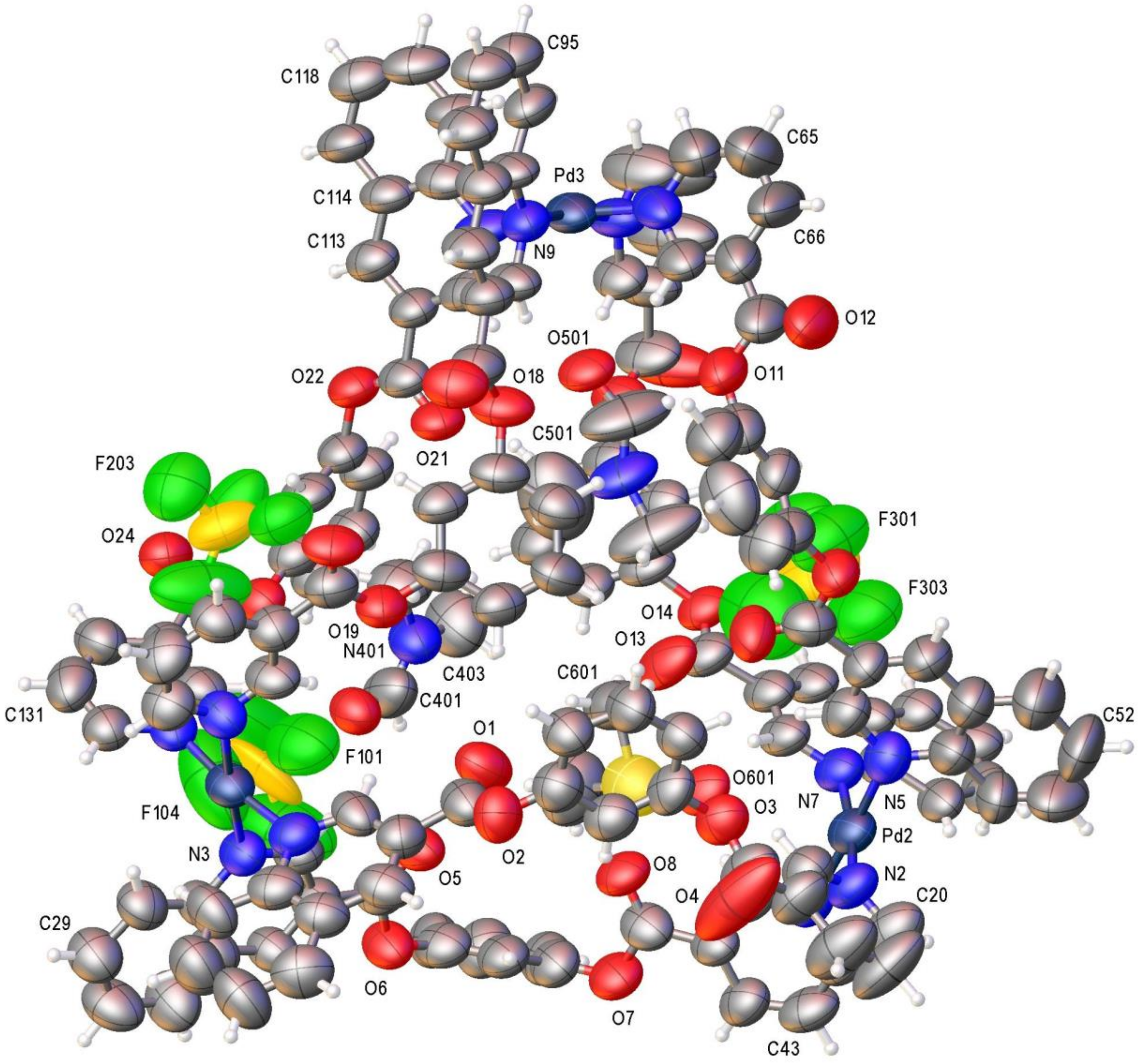


**Figure S253 Crystal structure of *cis*-[$Pd_2(L3^Q)_4 \supset DMF_2DMSO](BF_4)_6$ with ellipsoids drawn at the 50 % probability level.**

## S5.5 $[Pd_2(L2^H)_2(L4^Q)_4 \supset BF_4](BF_4)_3$

**$L2^H$** (8.4 mg, 30 µmol, 1 eq.), **$L4^Q$** (6.9 mg, 30 µmol, 1 eq.) and $[Pd(CH_3CN)_4](BF_4)_2$ (14.7 mg, 33 µmol, 1.1 eq.) were stirred at 70 °C in $CH_3CN$ (3.0 mL) under $N_2$ for 24 h. The cooled reaction mixture was filtered through celite and the solution left for vapour diffusion of $Et_2O$ from which X-ray quality crystals grew.

$C_{75}H_{48.5}B_4F_{16}N_{9.5}Pd_2$ (*M* =1642.77 g/mol): monoclinic, space group $P2_1$ (no. 4), *a* = 20.9081(4) Å, *b* = 10.9321(2) Å, *c* = 36.1901(8) Å, *β* = 91.610(2)°, *V* = 8268.7(3) Å$^3$, *Z* = 4, *T* = 100.15 K, μ(?) = 0.477 mm$^{-1}$, *Dcalc* = 1.320 g/cm$^3$, 101686 reflections measured (2.182° ≤ 2Θ ≤ 49.676°), 30995 unique ($R_{int}$ = 0.2197, $R_{sigma}$ = 0.2671) which were used in all calculations. The final $R_1$ was 0.1763 (I > 2σ(I)) and $wR_2$ was 0.4320 (all data). CCDC 2300290.

The data were collected at the Diamond Light Source but the diffraction was very weak. The refinement is the best that could be achieved with the data available.

The structure occupies a chiral space group and has been refined as an inversion twin, with the refined percentage occupancy of the two domains being 84 (8) : 16 (8).

The structure contains two crystallographically-independent palladium dimers and four $BF_4$ anions per dimer. One of the anions, B(901),F(901)-F(904) / B(91A), F(91A)-F(94A) is disordered over two positions, at a refined percentage occupancy ratio of 74.1 (17) : 25.9 (17). It was not possible to refine the minor disordered component anisotropically.

Two molecules of acetonitrile have been refined. Remaining solvent molecules could not be refined, and a solvent mask has been used.

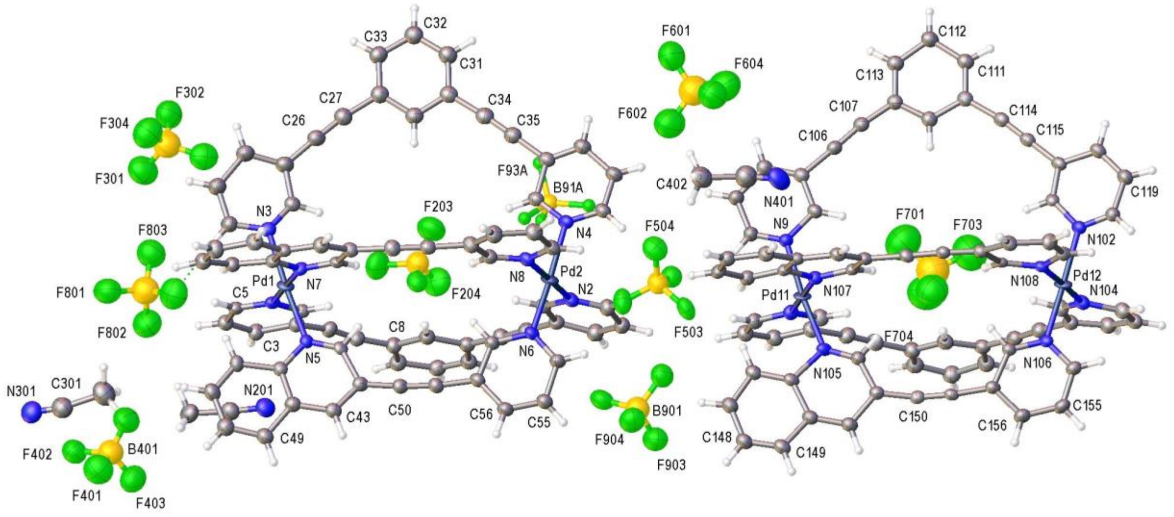


**Figure S254 Crystal structure of $[Pd_2(L2^H)_2(L4^Q)_2 \supset BF_4](BF4)_3$ with ellipsoids drawn at the 50 % probability level.**

## S6. References


[1] M. K. Singh, A. Etcheverry-Berríos, J. Vallejo, S. Sanz, J. Martínez-Lillo, G. S. Nichol, P. J. Lusby and E. K. Brechin, *Dalton Trans.*, 2022, **51**, 8377.

[2] A. K. Rappé, C. J. Casewit, K. S. Colwell, W. A. Goddard III and W. M. Skiff, *J. Am. Chem. Soc.*, 1992, **114**, 10024.

[3] L. Turcani, A. Tarzia, F. T. Szczypiński and K. E. Jelfs, *J. Chem. Phys.*, 2021, **154**, 214102.

[4] J. D. Gale, *J. Chem. Soc., Faraday Trans.*, 1997, **93**, 629-637; J. D. Gale and A. L. Rohl, *Molecular Simulation*, 2003, **29**, 291.

[5] D. E. Coupry, M. A. Addicoat and T. Heine, *J. Chem. Theory Comput.*, 2016, **12**, 5215; M. A. Addicoat, N. Vankova, I. F. Akter and T. Heine, *J. Chem. Theory Comput.*, 2014, **10**, 880.

[6] C. Bannwarth, S. Ehlert and S. Grimme, *J. Chem. Theory Comput.*, 2019, **15**, 1652; M. Bursch, H. Neugebauer and S. Grimme, *Angew. Chem. Int. Ed.*, 2019, **58**, 11078.

[7] A. Tarzia, J. E. M. Lewis and K. E. Jelfs, *Angew. Chem. Int. Ed.*, 2021, **60**, 20879.

[8] RDKit: Open-source cheminformatics. https://www.rdkit.org.

[9] Gaussian 16, Revision C.01, M. J. Frisch, G. W. Trucks, H. B. Schlegel, G. E. Scuseria, M. A. Robb, J. R. Cheeseman, G. Scalmani, V. Barone, G. A. Petersson, H. Nakatsuji, X. Li, M. Caricato, A. V. Marenich, J. Bloino, B. G. Janesko, R. Gomperts, B. Mennucci, H. P. Hratchian, J. V. Ortiz, A. F. Izmaylov, J. L. Sonnenberg, D. Williams-Young, F. Ding, F. Lipparini, F. Egidi, J. Goings, B. Peng, A. Petrone, T. Henderson, D. Ranasinghe, V. G. Zakrzewski, J. Gao, N. Rega, G. Zheng, W. Liang, M. Hada, M. Ehara, K. Toyota, R. Fukuda, J. Hasegawa, M. Ishida, T. Nakajima, Y. Honda, O. Kitao, H. Nakai, T. Vreven, K. Throssell, J. A. Montgomery, Jr., J. E. Peralta, F. Ogliaro, M. J. Bearpark, J. J. Heyd, E. N. Brothers, K. N. Kudin, V. N. Staroverov, T. A. Keith, R. Kobayashi, J. Normand, K. Raghavachari, A. P. Rendell, J. C. Burant, S. S. Iyengar, J. Tomasi, M. Cossi, J. M. Millam, M. Klene, C. Adamo, R. Cammi, J. W. Ochterski, R. L. Martin, K. Morokuma, O. Farkas, J. B. Foresman, and D. J. Fox, Gaussian, Inc., Wallingford CT, 2016.

[10] C. Adamo and V. Barone, *J. Chem. Phys*., 1999, **110**, 6158.

[11] J. Heyd, E. G. Scuseria and M. Ernzerhof, *J. Chem. Phys*., 2003, **118**, 8207; J. Heyd, E. G. Scuseria and M. Ernzerhof, *J. Chem. Phys*., 2003, **124**, 219906.

[12] F. Weigend and R. Ahlrichs, *Phys. Chem. Chem. Phys*., 2005, **7**, 3297.

[13] R.-J. Li, A. Tarzia, V. Posligua, K. E. Jelfs, N. Sanchez, A. Marcus, A. Baksi, G. H. Clever, F. Fadaei-Tirani and K. Severin, *Chem. Sci.*, 2022, **13**, 11912.

[14] L. M. Debefve and C. J. Pollock, *Phys. Chem. Chem. Phys.*, 2021, **23**, 24780.

[15] M. Holz, X. Mao, D. Seiferling and A. Sacco, *J. Chem. Phys.*, 1996, **104**, 669.

[16] CrysAlisPro, Rigaku Oxford Diffraction, 2020 & 2021.

[17] D. R. Allan et al., *Crystals*, 2017, **7**(11), 336.

[18] J. Beilsten-Edmands et al., *Acta Cryst.*, 2020, **D76**, 385-399; P. Evans, *Acta Cryst.*, 2006, **D62**, 72-82; P. R. Evans and G. N. Murshudov, *Acta Cryst.*, 2013, **D69**, 1204-1214; M. D. Winn et al., *Acta Cryst.*, 2011, **D67**, 235-242, G. Winter, *J. Appl. Cryst.*, 2010, **43**, 186-190; G. Winter et al., *Acta Cryst.*, 2018, **D74**, 85-97.

[19] O. V. Dolomanov, L. J. Bourhis, R. J. Gildea, J. A. K. Howard, H. Puschmann, *J. Appl. Crystallogr.*, 2009, **42**, 339-341.

[20] G. M. Sheldrick, *Acta Cryst.*, 2015, **A71**, 3-8.

[21] G. M. Sheldrick, *Acta Cryst.*, 2015, **C71**, 3-8.
